# *Plasmodium vivax* readiness to transmit: implication for malaria eradication

**DOI:** 10.1186/s12918-018-0669-4

**Published:** 2019-01-11

**Authors:** Swamy Rakesh Adapa, Rachel A. Taylor, Chengqi Wang, Richard Thomson-Luque, Leah R. Johnson, Rays H. Y. Jiang

**Affiliations:** 1 Department of Global Health (GH) & Center for Drug Discovery and Innovation (CDDI), College of Public Health, University of South Florida, Tampa, FL 33612 USA; 2 Department of Integrative Biology, University of South Florida, Tampa, FL USA

**Keywords:** Malaria, RNAseq, Mathematical modelling, Disease transmission, *Plasmodium vivax*

## Abstract

**Background:**

The lack of a continuous long-term in vitro culture system for *Plasmodium vivax* severely limits our knowledge of pathophysiology of the most widespread malaria parasite. To gain direct understanding of *P. vivax* human infections, we used Next Generation Sequencing data mining to unravel parasite in vivo expression profiles for *P. vivax,* and *P. falciparum* as comparison.

**Results:**

We performed cloud and local computing to extract parasite transcriptomes from publicly available raw data of human blood samples. We developed a Poisson Modelling (PM) method to confidently identify parasite derived transcripts in mixed RNAseq signals of infected host tissues. We successfully retrieved and reconstructed parasite transcriptomes from infected patient blood as early as the first blood stage cycle; and the same methodology did not recover any significant signal from controls. Surprisingly, these first generation blood parasites already show strong signature of transmission, which indicates the commitment from asexual-to-sexual stages. Further, we place the results within the context of *P. vivax’s* complex life cycle, by developing mathematical models for *P. vivax* and *P. falciparum* and using sensitivity analysis assess the relative epidemiological impact of possible early stage transmission.

**Conclusion:**

The study uncovers the earliest onset of *P. vivax* blood pathogenesis and highlights the challenges of *P. vivax* eradication programs.

**Electronic supplementary material:**

The online version of this article (10.1186/s12918-018-0669-4) contains supplementary material, which is available to authorized users.

## Background

*Plasmodium vivax (P. vivax)* infection has the most widespread distribution across different continents of any malaria parasite, with up to 2.6 billion people estimated to be at risk [1]. It can lead to severe disease and death but, despite the high disease burden [2], there is a lack of in-depth understanding of the distinct pathogenesis of *P. vivax*. This has resulted in a lack of targeted control measures. Thus, as malaria cases decline overall, the proportion of cases attributable to *P. vivax* is on the rise [3].

Human malaria infection starts with the inoculation of sporozoites into the skin dermis through the proboscis of female *Anopheles* mosquitoes; the sporozoites are hosted in her salivary glands. Some part of the inoculum enters the bloodstream and within a few minutes they invade hepatocytes in the liver [4, 5]. During the next five to 8 days (depending on the Plasmodium spp), the parasite transforms into a large exoerythrocytic form, packed with thousands of merozoites inside a parasitophorous vacuolar membrane (PVM). As the parasite matures the membrane breaks down into small packets of vesicles filled with merozoites. These are released into the bloodstream, leading to erythrocytic invasion [6]. In the next 48 h (depending on species) the parasite undergoes mitotic division and cytoplasmic growth inside the erythrocyte. They may develop either directly into a schizont (asexual) or gametocyte (sexual) [7]. For *P. falciparum* the sexual stages are not found in the periphery until after multiple blood stage cycles because gametogenesis, which requires bone marrow sequestration, takes 10 to 12 days, to achieve the fully transmissible stage V gametocyte [8]. In contrast, the appearance of *P. vivax* sexual stages is believed to be much earlier [7]. However, whether sexual commitment in *P. vivax* occurs early still needs to be determined.

*P. vivax* has a complex transmission cycle with distinct biological features compared to other malaria parasites, most notably: the high prevalence of asymptomatic carriers and the potential for disease relapses [7, 9] and gametocytes in circulation at the very beginning of infections [10]. In contrast to the better studied *Plasmodium falciparum*, *P. vivax* has the unique ability to remain as dormant hypnozoites in a hepatocyte in the liver and, in the future, to reactivate a blood stage infection leading to what is termed a clinical relapse [7, 11]. Unlike *P. falciparum*, there are currently no established laboratory methods to perform continuous long-term culture of *P. vivax* in vitro [12]. Furthermore, in *P. vivax*, the merozoites from both exo-erythrocytic and intra-erythrocytic schizogony have strong preferences of infecting reticulocytes [12], which typically comprise about 1 % of red blood cells. This leads to low parasitemia rates in peripheral circulation. The host requirement of human reticulocytes and many other technical challenges hampers studies of this parasite. These unique *P. vivax* life cycle characteristics pose major challenges for the understanding of *P. vivax* pathogenesis and hence the elimination of malaria worldwide [7].

We propose that better understanding of these complex characteristics of *P. vivax* can be achieved by employing a variety of new and established computational and mathematical methods. We utilise cloud computing to achieve this, as we believe that the cloud has the potential to transform future analyses of sequencing data. These tools can uncover traits of *P. vivax* that cannot be found experimentally as well as teasing apart the different roles of these traits in transmission of *P. vivax* at the population level.

**Fig. 1:**
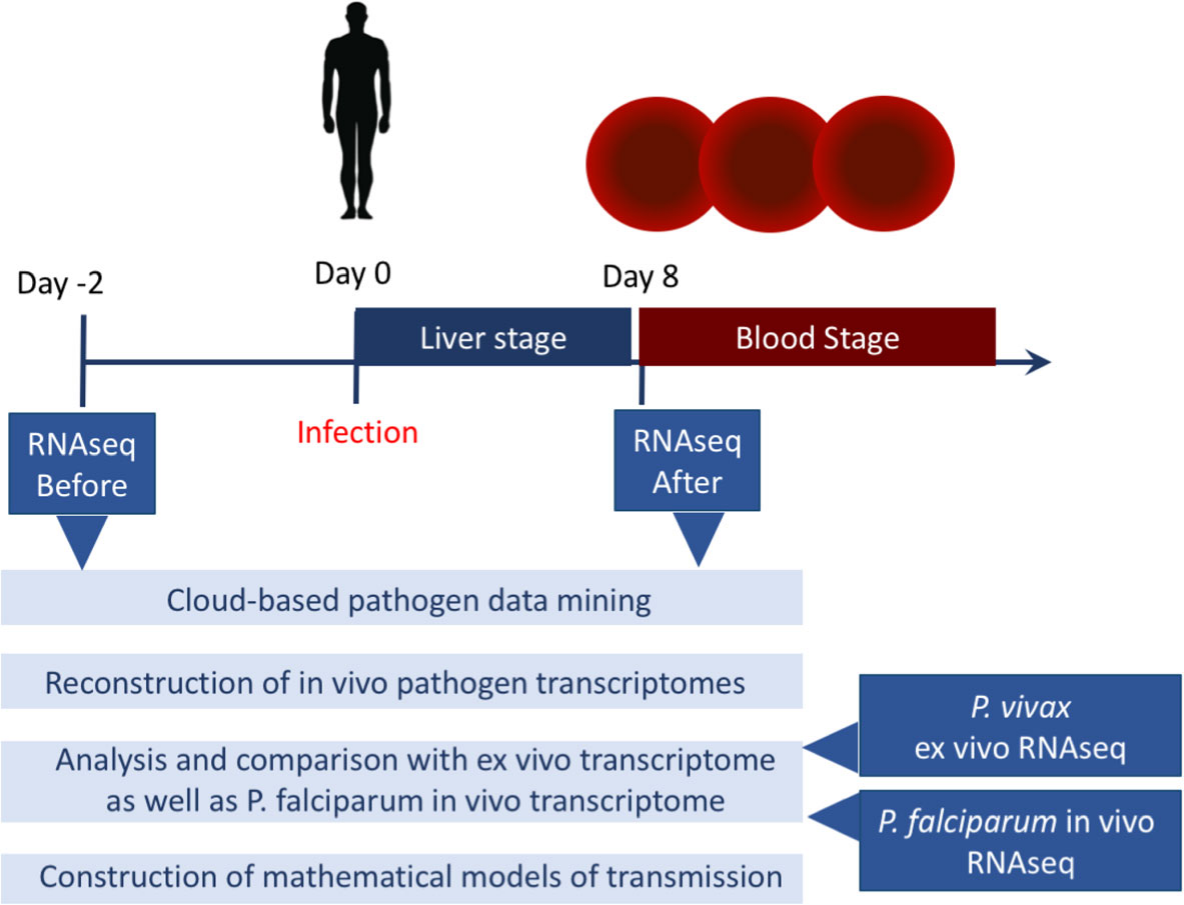
Study design and protocols. We have used two sets of RNAseq raw reads data pre and post sporozoite challenge from Rojas-Peña, et al. The post challenge data are inferred as the first blood stage cycle sequencing data. The early transcriptome signature is compared with publicly available in vivo *P. falciparum* and *ex vivo* *P. vivax* data to cross-validate the gametocyte signature in the early in vivo *P. vivax* infection

In this study (Fig. 1), we examined patient blood sequencing data using data mining techniques to recover *P. vivax* transcripts in the earliest time point possible during the blood stage, i.e. immediately after sporozoite invasion and the liver stage parasite ruptures into the blood stream. We discovered a very early gametogenesis expression signature, indicating the possibility of very early sexual commitment and possible transmission. To evaluate the epidemiological impact of this possible early transmission, we constructed a novel mathematical model of *P. vivax* transmission, which quantifies the effect of relapses, asymptomatic carriers and early transmission. We used sensitivity analysis to compare all of these characteristics of the *P. vivax* life cycle on the spread of the disease at a population level. Combining all of these methods allows us to explain why *P. vivax* transmits so successfully and hence why it may be the most difficult malaria to eradicate.

## Results

### Using cloud-based computational pipelines to mine parasite derived transcript

**Fig. 2:**
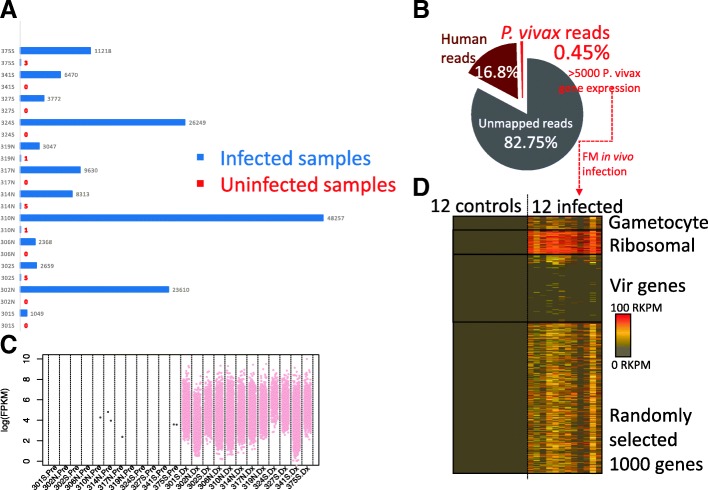
Recovering the earliest in vivo *P. vivax* blood stage transcriptome. Uninfected and post-infection blood samples were derived from the same individual. A total of 12 paired individual genomics data were analysed. **a** Cloud-based sequence mining revealed that only the post-infection RNAseq raw data set contains parasite sequences in all patients. Patient identifiers are from the publication by Rojas et al. The *P. vivax* reads number is generated with stringent criteria and reflects conservative estimation. **b** On average, less than 0.5% of total signal is derived from *P. vivax*. The mapped data of total reads and percentage of alignment in individual patient samples are listed in Table 1. **c** The log(FPKM) distribution of all patients. FPKM represents fragments per kilobase of exon per million fragments mapped. Pre represents uninfected, while Dx means infected. Only the genes with FPKM > 0 are plotted here. The labels on the horizontal axis represent de-identified patient numbers. **d** RNAseq recovered parasite transcriptome in infected samples. RKPM refers to Reads Per Kilobase of transcript per Million mapped reads. Genes expressed in at least two patients are plotted

To understand *P. vivax* in vivo pathogenesis, we first utilized a set of publicly available NGS (Next Generation Sequencing) raw data from Rojas-Pina et al. [13] that examined human immune responses against malaria. We performed computational analysis to extract the low levels of parasite signals from the raw sequencing data, by using pre-infection data as negative controls (Fig. 1). The study by Rojas-Pina et al. performed sporozoite challenge on 12 volunteers with a single source of *P. vivax*, and generated whole blood RNAseq before and after the challenge. The post-infection RNAseq was produced around day 9 (diagnosis day), i.e., the first blood stage cycle after the liver stage infection which usually lasts for about 6–7 days [4, 5]. Due to the very low levels of parasitemia at this time point, we first used a cloud-based data mining pipeline to obtain pathogen sequences (*P. vivax*) in order to investigate the feasibility of our project. We deployed the program PathoScope 2.0 in the Amazon Elastic Compute Cloud (Amazon EC2: aws.amazon.com/ec2), due to the computational scalability that can be achieved within a few minutes. We mapped the entire set of raw sequencing reads to the NCBI NR(Non-Redundant) reference sequences and set *P. vivax* reference (Sal I) as targets. We have also used other pathogens such as viruses and bacteria as non-targets to increase the search specificity. From a total of 12 pairs of pre and post infection RNAseq raw sequencing reads data sets, we successfully detected *P. vivax* sequences from 1000 to almost 50,000 reads in post-infection samples (Table 1) (Fig. 2a). In contrast, none of the pre-infection samples gave a significant amount of reads (> 10).

**Table 1:** Patient specific information from literature and RNAseq data analysis

Patient Number	SRR (Sequence sample number) [11]	Parasite Density on Pre-patent Day (Parasites/μL)	Patient Location [11]	Total Reads	% reads aligned to Parasite Genome	% reads aligned to Human Genome
1	SRR1925783	6	Cali	800,452	0.32	17.52
2	SRR1925785	10	Cali	1,410,398	0.24	16.92
3	SRR1925803	20	Buenaventura	949,274	0.09	16.1
4	SRR1925797	25	Buenaventura	415,781	0.19	19.52
5	SRR1925781	34	Cali	725,123	1.1	18.16
6	SRR1925795	34	Buenaventura	446,940	0.09	15.79
7	SRR1925787	38	Cali	1,055,063	0.1	13.43
8	SRR1925799	55	Buenaventura	587,972	0.29	16.51
9	SRR1925788	95	Cali	1,570,675	0.43	16.81
10	SRR1925790	110	Cali	712,547	1.13	18.48
11	SRR1925798	216	Buenaventura	1,238,628	0.98	15.14
12	SRR1925791	390	Buenaventura	711,395	0.2	17.49

### Reconstruction of *P. vivax* in vivo transcriptome from very early blood stage infection

Next, we used the Tuxedo RNAseq pipeline [14] to reconstruct transcriptomes from the 12 post-infection samples, which is deployed in USF research computing cluster. We aligned the entire sequence data to *P. vivax Sal 1* and Human (*GHRc37*) reference genome and estimated the transcript abundances. The majority of the raw reads cannot be assigned to any references, primarily due to reads quality and possibly a small amount belonging to unknown genomes and the Phix179 control genome generally used during sequencing library construction. Reads originating from Phix were filtered prior to implementation of the Tuxedo RNAseq pipeline. An average of 16.8% of the reads can be mapped to human genome reference *GRCh37*. On average only 0.45% total reads on average mapped to the *P. vivax* reference genome (Fig. 2b). From the 12 post-infection RNAseq pathogen transcriptomes, we can detect over 95% of the 5625 total protein coding genes expressed at > 20 FPKM (fragments per kilobase of exon per million fragments mapped). For each patient, we can identify from 9% to over 50% of the total protein-coding *P. vivax* genes are expressed at > 20 FPKM (Additional file 1: Figure S1A).

To confidently identify parasite derived RNAseq signal from infected host tissues, we developed a Poisson Modelling (PM) method to characterize positive pathogen signals above the background (i.e. parasite expression levels in the pre-infected samples). We modelled background signal with a Poisson distribution and estimated the significance of detected parasite transcriptional levels with a Maximum Likelihood method (Additional file 2: Table S1 and Additional file 3: TableS2).

Subsequently, we performed PM at two levels (Additional file 1: Figure S2A). First, we used PM to evaluate patient level infection signals of before and after infection, taking the entire transcriptomes into account. Second, we used a gene-by-gene PM evaluation approach to identify the significantly expressed parasite genes in mixed sequencing results from human tissues as compared to pre-infection samples (Additional file 1: Figure S2B). To cross-validate our PM method, we have independently built our statistical model based on Negative Binomial Model (NBM) (Additional file 1: Figure S3A B) and obtained very similar results with only 0–2 genes expression in the negative control of pre-infected samples.

To understand the molecular patterns associated with *P. vivax* parasitemia, we designed a computational method to search for parasitemia associated genes. First, we grouped the patients into low (<=25 ul), medium (34–55/ul) and high parasitemia (95–300/ul) groups, based on the reported levels of parasitemia on pre-patent day, i.e. a range of 11–13 days [15], a few days later than the RNAseq samples were collected. We recognize that, in reality, all the patients have very few parasites during this early stage of infection, and the categories are primarily for statistical analysis. Then we performed a non-parametric statistical analysis (Wilcoxon test with *p* values adjusted with multiple hypothesis testing correction) to search for transcripts that are positively and significantly associated with the levels of parasitemia. In the top 20 in vivo parasitemia associated genes (*p* < 0.05), we identified genes with peak expressions in different asexual stages such as ring, trophozoite and schizont. The top markers from parametric analysis (based on Pearson’s r) are consistent with that of non-parametric analysis. We clearly identify a gametocyte expression signature at this early stage of in vivo infection in the top ranking markers.

### *P. vivax* early blood infection is associated with gametocytogenesis

**Fig. 3:**
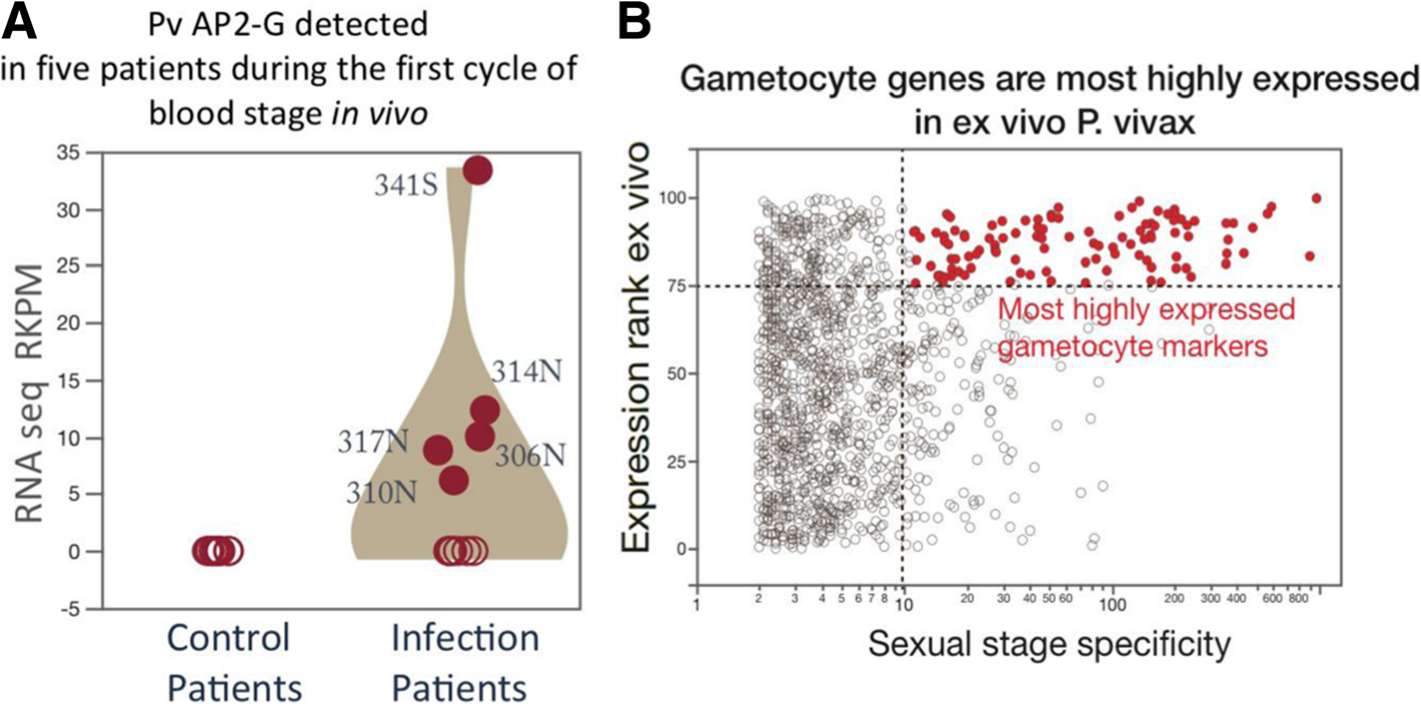
Discovering gametocyte signatures from early *P. vivax* in vivo RNAseq. **a** Five patients showed expression of PvAP2-G, a master regulator of Plasmodium gametocyte production. The numbers on the plot represent de-identified patient numbers. **b** Gametocyte specific genes are the most highly expressed genes in ex vivo *P. vivax* RNAseq transcriptomes. The x axis refers to the ratio of FPKM levels for sexual to asexual stages gene expressions. The top quartile of most highly expressed genes (Normalized rank score > =75) in the ex vivo data consisted of more than 40% of gametocyte specific genes

To understand the extent of gametocytogenesis gene expression and its relationship with parasite abundance, we search for how many known gametocyte specific markers are expressed. We first defined a set of 280 gametocyte specific genes by using *P. falciparum* orthologous gene expression specificity (details in Materials and Methods). We discovered that between 8 to 60% all sexual stage specific genes are expressed in this early blood stage (Additional file 1: Figure S1B). To further investigate the gametocytogenesis transcription pattern, we identified 48 gametocyte related genes from the patient infected transcriptomes. We were able to identify stage specific gametocyte markers with early markers [16] such as tubulin-specific chaperone PVX_081315 and Pvs16 PVX_000930. We also found late markers such as PVX_116610, indicating that there might be mixed stages of gametocyte obligation at this early blood stage. Furthermore, we found gender specific markers, such as female markers like PVX_093600; as well as male markers such as PVX_116610 (Additional file 4: Table S3), indicating that there were mixed genders of gametocytes developed from a single source of sporozoite challenge. The transcriptional factor PvAP2-G is considered a master regulator and a specific marker for early gametocyte production [17]. We are able to clearly identify transcripts of PvAP2-G in 5 patients in the early blood stage (Fig. 3a).

To validate our findings of gametocytogenesis expression signature in vivo, we analyzed an independently generated, publicly available data set of ex vivo RNAseq data from pooled infected patient blood [18]. To search for relationships between expression levels and gametocyte production, we classified the ex vivo *P. vivax* expression based on two transcriptome features in orthologous genes of *P. falciparum*, namely, 1) Level of expression and 2) Specificity of gametocyte stage expression. We first computed the average FPKM for each gene and converted the values into a rank score from 0 to 100, with 100 representing the highest relative expression levels. Then we analyzed the levels of gametocyte expression specificity by calculating the ratio of sexual stage FPKM vs. asexual stage FPKM, the higher values indicate higher levels of sexual stage expression specificity. We found that the gametocyte specific genes in fact are the highest expressed genes in the ex vivo data (Fig. 3b). The top quartile of most highly expressed genes in the ex vivo data consists of more than 40% of gametocyte specific genes. The ex vivo data has even stronger gametocyte expression pattern than that of the early in vivo data (Additional file 1: Figure S4). The ex vivo enhanced gametocyte induction could be due to the abiotic stress of the culture conditions [19]. By analyzing the precise peak expression time in the ex vivo expression data set, we found that gametocyte specific genes are mostly expressed in late schizont/early ring stage, despite the fact that these stages have the lowest number peak expression genes (Additional file 1: Figure S5 A B). *P. falciparum* and *P. vivax* appear to share a pattern in which commitment to gametocyte development occurs in the schizont stage [20]. The ex vivo analysis strongly supports our in vivo analysis, that *P. vivax* parasitemia is associated with commitment to gametocytemia.

**Fig. 4:**
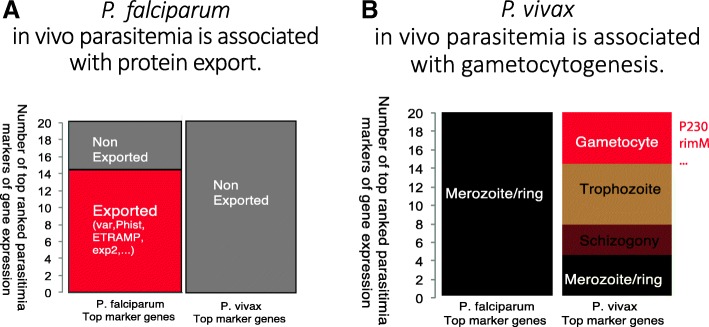
Comparison of *P. falciparum* and *P. vivax* in vivo transcriptomes. Top ranked markers that correlated with the levels of parasitemia are used for plotting. The top ranked parasitemia markers in *P. falciparum* are derived from 116 patients’ in vivo infection data. And the top ranked parasitemia markers in *P. vivax* are from 12 in vivo early infection data. **a** Exported protein proportions in *P. falciparum* and *P. vivax*. Exported proteins are defined as PlamsoDBv27 PEXEL containing proteins; and they are likely be involved in host cell remodelling. **b** Life cycle peak expression markers in *P. falciparum* and *P. vivax*. The peak expression patterns are assigned with all differentially expressed genes in 7 stages when there are more than 2-fold difference between stages

We next compared our in vivo *P. vivax* analysis with that of *P. falciparum*. Similar to *P. vivax* analysis, we have identified the top 20 transcripts associated with parasitemia from 116 whole blood samples of *P. falciparum* infected patients (data deposited in the publication by Yamagishi, et al., [21] (Fig. 4a, b). We defined these markers by searching for the gene expression levels that are most strongly associated in Spearman correlations with the levels of *P. falciparum* parasitemia among over 5000 unique transcripts. We found that in contrast to *P. vivax* parasitemia markers, *P. falciparum* parasitemia driven genes have peak expression only in the merozoite/early ring stages and many of them are associated with protein export as PEXEL containing proteins. None of the top *P. falciparum* parasitemia markers are gametocyte related in terms of peak expression pattern.

The differences between in vivo *P. vivax* and *P. falciparum* expression in relation to gametocytogenesis is twofold as revealed by our analysis. First, *P. vivax* has gametocyte specific gene expressions correlated with parasitemia levels in this in vivo data set. Secondly, *P. vivax*’s most abundant in vivo and ex vivo transcripts include gametocyte specific genes (Additional file 1: Figure S4). For *P. falciparum*, neither parasitemia correlations nor high gene expression levels show strong associations with sexual commitments. Although *P. falciparum* in vivo data do contain some sexual stage gene transcripts, the expression levels are much lower as compared to *P. falciparum* merozoite/ring stage gene expressions levels.

Therefore, we conclude that the two malaria parasites in vivo pathogenesis show distinct patterns. *P. falciparum* parasitemia is likely to be more associated with asexual cycle protein export and host red blood cell remodelling; whereas *P. vivax* shows clear gametocyte expression signatures from the first blood stage cycle.

### Mathematical modelling shows unique *P. vivax* transmission pattern

**Fig. 5:**
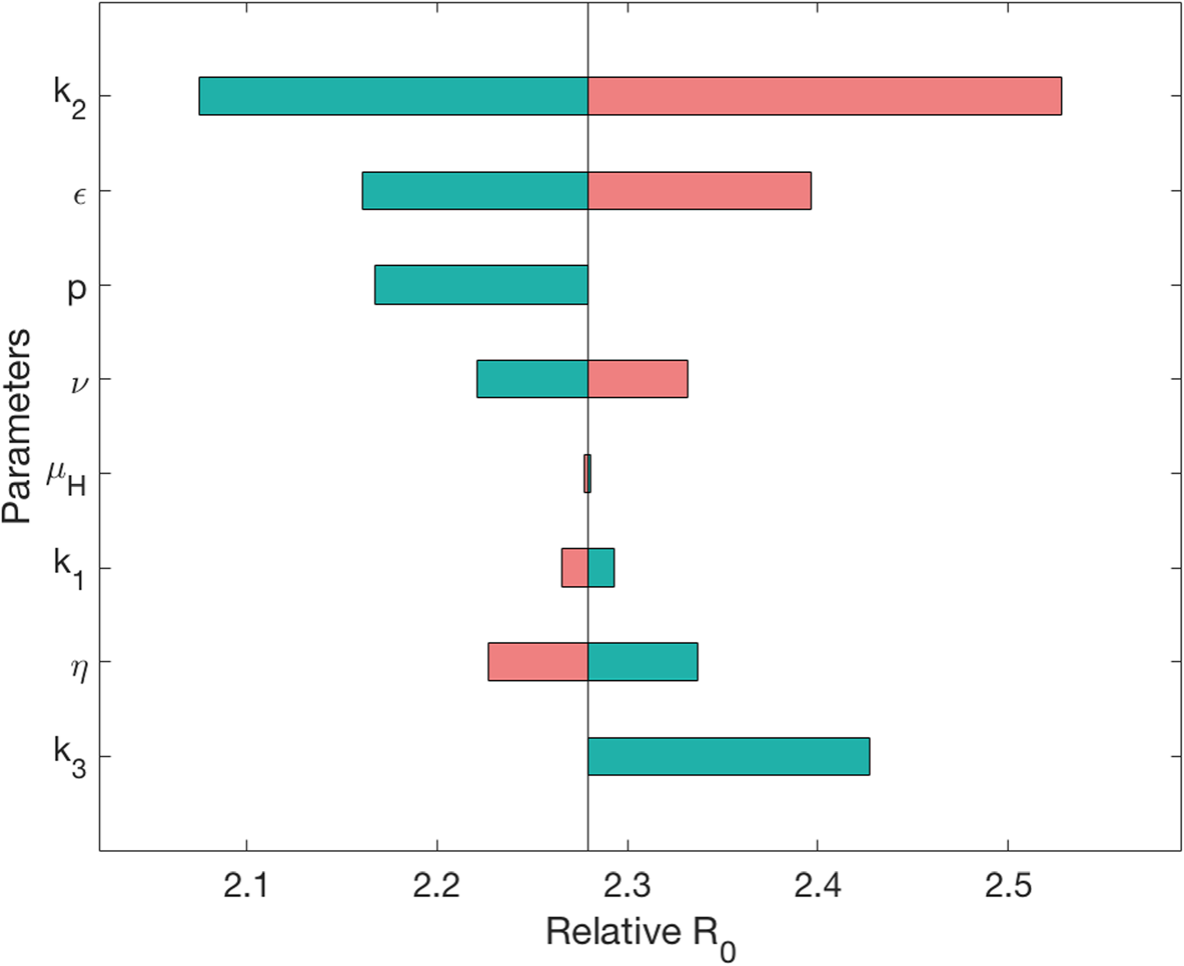
Mathematical model of *P. vivax* exploring the effect of reduced incubation period on spread of disease. A sensitivity analysis is performed on relative *R*_0_ for *P. vivax* (relative to *P. falciparum*). Green indicates when the parameter has been lowered from its baseline value, pink indicates higher than baseline (therefore *R*_0_ is positively correlated with the first four parameters and negatively correlated with the last four parameters). Parameters *ε*, *p* and *k*_3_ are varied between 0 and 7, 0–1, and 0–1 respectively, all other parameters are varied by 10%. Parameters and their baseline values are: proportion of hosts that develop hypnozoites (*k*_2_, 0.68), reduction in incubation time (*ε*, 3.5), proportion of hosts developing symptoms in *P. falciparum* (*p*, 1), rate of relapsing (*ν*, 1/72), host death rate (*μ*_*H*_, 3.84 × 10^−5^), proportional rate of disease-induced death for *P. vivax* (*k*_1_, 0.25), rate of hypnozoite death in liver (*η*, 1/223) and proportion of hosts developing symptoms in *P. vivax* relative to *P. falciparum* (*k*_3_, 1). Parameter values are in Additional file 5: Text S1

*P. vivax* has unique biology with respect to its population dynamics, such as hypnozoite formations and disease relapses. Our finding of potential early transmission adds another important aspect to its population dynamics. To evaluate these different parameters' impact on disease transmission, we performed a sensitivity analysis of the effect of different components of *P. vivax* disease spread (Fig. 5). The most influential parameter on relative *R*_0_, the basic reproductive number of the disease, is *k*_2_, which determines the proportion of human hosts that recover with hypnozoites, and hence the possibility of relapse. It causes *R*_0_ for *P. vivax* to vary from less than 2.1 to over 2.5 times the values for *P. falciparum* (set to 1, when *p* = 1, where *p* is the proportion of *P. falciparum* hosts that are symptomatic). The second most influential parameter on changes in relative *R*_0_ is *ε*, the reduction in the length of the incubation period. When there is no difference between *P. falciparum* and *P. vivax*, that is *ε* is 0 days, *R*_0_ is lowered to less than 2.2. However, when the incubation period is shortened for *P. vivax* by *ε* = 7 days, as we expect from our experimental results, *R*_0_ is 2.4. Therefore, if the reduction in incubation time is not considered, mathematical models could miscalculate *R*_0_, underestimating it by approximately 11%. Parameters *υ* and *η*, the rate of relapse and the rate of hypnozoite death in the liver respectively, are also influential in determining the value of *R*_0_, as are the parameters related to proportion of hosts that show symptoms, *p* and *k*_3_. Varying *p* from 1 to 0 increases the number of asymptomatic hosts in both *P. falciparum* and *P. vivax.* Even though *k*_3_ = 1 in this scenario and, therefore, there are exactly the same proportions of asymptomatic cases in both diseases, this change in *p* leads to a reduction in the relative value of *R*_0_ for *P. vivax.* This is because it increases *R*_0_ for *P. falciparum* proportionally more than it increases *R*_0_ for *P. vivax* (the actual increases are approximately the same but *P. falciparum* has a lower initial value)*.* Therefore, the role of asymptomatic hosts in malaria generally leads to increased *R*_0_ equally for the two diseases. However, the difference in *R*_0_ is larger (i.e. *R*_0_ is more sensitive) when *k*_3_ is varied compared to *p*. This indicates that it is necessary to understand the likelihood of asymptomatic cases in *P. vivax* compared to *P. falciparum* to accurately predict differences in disease spread. The influence of these parameters highlights the importance of understanding the role of the asymptomatic stage correctly.

We further explore the role of the reduction in the incubation period length in Additional file 1: Figure S6, which shows the effect of not including relapses in the model and not accounting for asymptomatic hosts transmitting the infection in *P. vivax*. When we model the asymptomatic class as capable of transmitting infection but are unsure what proportion of hosts are in this category, our uncertainty in *R*_0_ is small (Additional file 1: Figure S6A). On the other hand, when asymptomatic hosts for *P. vivax* exist but the existence of these asymptomatic cases is unknown and hence not modelled as capable of spreading disease, there is a drastic underestimation of *R*_0_, the potential for spread, of *P. vivax* (Additional file 1: Figure S6B). In fact, if more than 40% of infectious hosts are asymptomatic compared to *P. falciparum*, the estimate of *R*_0_ for *P. vivax* would be less than for *P. falciparum* when in reality it is approximately 2.5 times larger. Similarly, when the model does not account for relapses, the estimate for *R*_0_ is halved (Additional file 1: Figure S6B).

## Discussion

Our study has uncovered the earliest possible in vivo infection transcription patterns of blood stage *P. vivax*, a parasite that cannot be cultured in the laboratory. With malaria eradication always in the spotlight of the scientific and public health community, there is an urgent need to understand the unique biological and physiopathological features of *P. vivax*. If, as our data suggest, *P. vivax* transmission to mosquitoes is plausible at the very first blood stage cycle immediately after liver stage development, this would represent a major hurdle towards targeting *P. vivax* reservoirs. Due to ethical and practical limitations to obtain experimentally infected *P. falciparum* data in vivo, our study used *P. falciparum* data without defined infection age. Nevertheless, the major differences we have discovered between *P. vivax* and *P. falciparum*, in terms of in vivo gene expression, suggest that *P. vivax* begins gametocyte production immediately upon entering the blood, whereas more research is needed for early gametocyte production in *P. falciparum*.

Early stage I gametocytes of *P. falciparum* can be initially in peripheral blood and are microscopically indistinguishable from early rings [22, 23]. Yet, we did not find strong transmission expression signatures. It stands to reason that the 1–2 weeks of bone marrow sequestration that *P. falciparum* needs in order to achieve a fully transmissible stage V truly represents an advantage for *P. vivax* transmission over *P. falciparum*. Further, it has been demonstrated [24] that even very few gametocytes in circulation, such as inferred from our study on *P. vivax*, can effectively mount an infection in the mosquito host [24]. Our mathematical model indicates that this advantage in early transmission for *P. vivax* leads to a higher reproductive number relative to *P. falciparum*, signifying a greater ability to spread quickly throughout populations. Hence, without including shorter incubation periods, models may underestimate the work required to reduce transmission of *P. vivax* within a population.

A study in native Amazonian populations [25] found that the proportion of symptomatic and asymptomatic clinical forms was roughly similar for both *P. falciparum* and *P. vivax*. However, others have reported that the relative proportion of submicroscopic *P. vivax* is significantly higher than that of *P. falciparum* [26, 27]. Taking into account that over 89% of *P. vivax* submicroscopic infections are said to be asymptomatic [28], the balance in terms of better asymptomatic transmissibility falls on the side of *P. vivax*. Altogether, this evidence suggests that the differences we have discovered between *P. vivax* and *P. falciparum*, in terms of in vivo gene expression, suggests that *P. vivax* has the ability to spread quickly to multiple hosts before the onset of symptomatic phenotypes. Our mathematical model found that the proportion of hosts that are asymptomatic in *P. vivax* infections has a greater impact on population spread of the disease than the proportion in *P. falciparum*. Further, the model highlights the importance of including the asymptomatic stage within models, even if the exact proportion of hosts that will not show symptoms is unknown (Additional file 1: Figure S6). A further factor to consider is that symptomatic cases of *P. vivax* are much more likely to seek treatment than asymptomatic cases [29]. We assumed the recovery rate was the same across symptomatic and asymptomatic cases. However, if the recovery rate for symptomatic cases is higher, the role of asymptomatic cases in increasing disease transmission will be even more significant.Thus, the results from across the computational methods we use confirm the idea, held widely, that *P. vivax* will be the last parasite standing before the goal of malaria eradication is to be achieved [3].

Our mathematical model highlights the important role of relapses and asymptomatic cases, similar to previous mathematical models of *P. vivax* [30, 31] and epidemiological studies [32, 33]. However, the sensitivity analysis we perform allows a quantitative comparison of each of these traits, including the reduction in incubation time, on the reproductive ratio, relative to *P. falciparum*. It asserts that relapses are the most influential factor on increases in disease spread. And yet, relapses are poorly understood with no consensus on what causes them to occur or on their frequency. Our model can be used for further exploration of *P. vivax* dynamics and can also be adapted to account for the potential evolutionary consequences of reducing the length of the incubation period. A shorter incubation period could indicate lower production of efficient gametocytes, therefore the probability of successful transmission from an infected human to mosquito could be reduced [34]. This could be achieved by introducing a trade-off function between these two parameters in the model. However, the form of this trade-off function is not clear and would need to be investigated experimentally. The potential reduction in incubation period, and hence early transmission, has a substantial impact on disease spread, dependent on this evolutionary trade-off. Other potential differences between *P. falciparum* and *P. vivax* such as a reduced development time in the mosquito or the length of waning immunity, could have an effect on the relative *R*_0_ between the diseases, but we have not included these in our model. This may limit our ability to understand the transmission properties of the two malaria strains. Nor did we include superinfection of multiple diseases within one host. Further studies are required to determine, for example, the interaction of different diseases within a single host. *P. falciparum* may mask the symptoms of *P. vivax* which essentially increases the proportion of asymptomatic *P. vivax* cases [35].Our model focuses instead on some of the differences between *P. falciparum* and *P. vivax* and assesses which are of most importance in determining changes in relative *R*_0_. The fact that our model is relatively simple compared to previous *P. vivax* models [30, 31, 36] allows us to more clearly determine how each parameter affects *R*_0_.

We used a cloud-based mining method as part of our study which we employed at a pricing of $0.13/h. All the computational storage was synced with Amazon Simple Storage Service (Amazon S3), which automatically scales according to the current usage requirements. This facility gave us a cost effective ($0.03 per GB) advantage over the fixed storage on the local computing cluster. Further, this approach does not require local High Performance Computing (HPC) facilities and can accommodate high volumes of data analysis within short time frames. As publicly available genomic data grow in complexity and volume every day, more efficient and more precise analytical tools are needed for future studies. Our study is an example for infectious disease researchers on how to use large raw sequencing data to investigate previously intractable pathogenesis-related features. Infectious disease scientists could use similar approaches in resource-limited research settings.

We use a wide variety of computational tools to uncover the transmission potential of *P. vivax*, such as cloud computing, data mining, mathematical models and cross-platform genomics data comparisons. In our study, we used a more robust method (non-parametric analysis) to give a conservative estimate for *P. vivax* infection data because the *P. vivax* data set is smaller than that of *P. falciparum*. The results of non-parametric and parametric analysis of *P. vivax* parasitemia associated gene expressions are mostly consistent (Pearson’s *r* = 0.784). Overall, the unique transmission of *P. vivax* leads to a much higher likelihood of disease spread compared to *P. falciparum* in similar settings. Although future transmission studies need to be conducted to further verify the transmission window of these malaria parasites, our work highlights the challenge of *P. vivax* eradication and provides evidence for the need for more thorough and earlier transmission intervention measures. Controlled transcriptomic studies comparing *P. falciparum* and *P. vivax* gametocyte gene expression in oocysts and sporozoites are needed in order to understand how soon sexual commitment is decided in the *P. vivax* complex life cycle. Since *P. vivax* commits to gametocytogenesis early in the blood stage, rationally designing a treatment or vaccine targeting the early blood stage will reduce transmission rates. However, targeting treatment at such an early stage is difficult to achieve and once again re-enforces the idea that *P. vivax* may be the most difficult malaria to eliminate.

## Conclusion

In this study, we used cloud and local computation with Poisson modelling to reconstruct the earliest in vivo blood stage transcriptome of *P. vivax* infection. We found that hundreds of sexual stage specific genes are already expressed in the first blood stage cycle. Furthermore, our novel mathematical modelling quantifies the epidemiological impact of the complex life cycle of *P. vivax*; and highlights the important challenges for *P. vivax* control.

## Methods

### Mining parasite data from infected human tissues

We used the blood transcriptome data sets deposited in Gene Expression Omnibus (GEO) under accession numbers GSE67184, GSE61252 associated with the in vivo *P. vivax* sporozoite challenge [13] and ex vivo *P. vivax* asexual stage culture [18] respectively. We also use the in vivo *P. falciparum* infection genomic reads [21] deposited in DNA Data Bank of Japan (DDBJ) under accession number DRA000949 to compare the transcript abundances with the above datasets.

PathoScope 2.0 [37] framework is used to quantify proportions of reads from individual species present in sequencing data from samples from environmental or clinical sources. A spot Elastic Computing Cloud (EC2) instance r3.4xlarge (Virtual CPUs – 16, Memory (GB) – 122, Storage (SSD GB) – 320)) was employed. We used the Patholib module along with National Centre for Biotechnology Information (NCBI) vast nucleotide database to create filter genomes containing host (human), microbes (virus, bacteria), artificially added sequence (PhiX Control v3, Illumina) and target genome library containing *P. vivax Sal-1* sequences using their respective taxonomic identifiers. PathoMap module is used to align the reads to target library using the Bowtie2 algorithm [38] and then filters reads that aligned to the filtered genomes. PathoReport was used to annotate the sequences.

The Tuxedo suite [14] of programs (Bowtie2, TopHat2, and Cufflinks) were used to process and analyze the data. Reference genomes of Human (*GHRc37*) from Ensembl human genome database and *P. vivax Sal-1* from PlasmoDB—a Plasmodium genome resource. Bowtie2 [38] was used to build indexes of the reference genomes. RNASeq reads from each sample were aligned to the *P. vivax Sal-1* genome using TopHat2 (v. 1.4.1) [39]. A maximum of one mismatch per read was allowed. The mapped reads from TopHat were used to assemble known transcripts from the reference, and their abundance FPKM (fragments per kilobase of exon per million fragments mapped) values were calculated for all genes using Cufflinks.

### Gene expression level estimation with Poisson modelling (PM)

Poisson distribution has been widely used to estimate the background level of gene expression [40–42]. In this work, we used Poisson distribution to model the background expression level (x) for each patient.

1 $$ x\sim Pois\left(\lambda \right) $$

2 $$ p\left(x|\lambda \right)=\frac{e^{-\lambda }{\lambda}^x}{x!} $$

It is well known that the unbiased estimator of *λ* is the mean value of *x*, which can be calculated from maximum likelihood estimation.

3 $$ \widehat{\lambda}=\frac{\sum x}{n} $$

where ∑*x* is the sum of gene expression level of specific patient or gene; *n* is the number of genes considered. Finally, we can compare the expression levels between different patients or genes by using the mean value of estimated distribution.

Negative Binomial (NB) distribution has been widely used to estimate the background level of gene expression [43–45]. In this study, we also used NB distribution as an additional method to model the background expression level (*x*) for each patient. The NB distribution also arises as a continuous mixture of Poisson distributions where the mixing distribution of the Poisson rate is a gamma distribution.

1 $$ x\sim Poisson\left(\lambda \right) $$

2 $$ \lambda \sim gamma\left(\gamma, \frac{1-p}{p}\right) $$

By using bayes rule

3 $$ P(x)=\int P\left(x|\lambda \right)P\left(\lambda \right)\  d\lambda =\frac{\Gamma \left(\gamma +x\right)}{x!\Gamma \left(\gamma \right)}{p}^x{\left(1-p\right)}^{\gamma } $$

The parameters can be estimated by maximum likelihood. Finally, we compared the expression levels between different patients (samples) or genes by using the expectation value of estimated distribution. The methods based on NBM and PM gave very similar results.

### Analysis of RNAseq data

The sexual stage specific genes are defined by using the 7 stages RNAseq data [16]. The stage specific RNAseq dataset is from Illumina-based sequencing of *P. falciparum* 3D7 mRNA from gametocyte stage II and gametocyte stage V), and ookinete. The dataset has also four time points of asexual stages representing ring, early trophozoite, late trophozoite, and schizont. The orthologs of *P. vivax* and *P. falciparum* were mapped with OrthoMCL data [46]. Sexual stage specific genes are required to have 20 or more fold expression level FPKM differences in the sexual stage (gametocytes, ookinete) vs the time points in the blood stages. The expression differences between asexual and sexual stages were analyzed with Fisher’s Exact tests, and the *P* values (< 0.001) were adjusted by multiple hypothesis correction with Benjamini-Hochberg method.

### Mathematical modelling of *P. vivax* transmission

We created two mathematical models to represent the population-level spread of disease among humans and mosquitoes for *P. falciparum* and *P. vivax* malaria. We do this to allow comparisons between the two malaria diseases, in order to assess which differences between the two have the most influence in producing the current epidemiological profile of the two diseases. This can inform us whether our genomics research results are an important aspect of *P. vivax* spread within populations. Similar to many models of vector-borne diseases which were developed for malaria, we categorize humans and mosquitoes into compartments based on their infection status, such as Susceptible, Exposed, Asymptomatic and Infected, [47–49] see Additional file 5: Text S1). In both models, we include a time delay in human acquisition of disease, the possibility of asymptomatic hosts, waning immunity, disease-induced death for symptomatic hosts, natural death and frequency-dependent transmission between mosquitoes and hosts. The major structural difference between the two models is the inclusion of hosts with hypnozoites in the *P. vivax* model; other differences are implemented through changes in parameter values. Specifically, the parameters that alter are the proportion of hosts with symptoms, the disease-induced death rate (lower for *P. vivax*), the length of the incubation period and the recovery rate. Accounting for relapses of *P. vivax* has been achieved in models usually by many additional compartments to represent all the various transitions that can occur (e.g. see [30, 31, 36]). Our model is novel for the simple but effective way it introduces relapses in *P. vivax*, by including a single compartment for humans with hepatocytes in which transition into and out of this compartment is parameterised by the average number of relapses each human is expected to have [50, 51]. For both the *P. vivax* and *P. falciparum* model we calculate *R*_0_, the basic reproductive number of the disease. This is a commonly used, fundamental metric of disease transmission potential defined as the number of people one infected person is able to infect in a susceptible population. If *R*_0_ > 1 then the disease is likely to take off and spread widely throughout the population. As the models for *P. vivax* and *P. falciparum* contain many similar components, we assess the relative *R*_0_ for the diseases, i.e. we divide all values of *R*_0_ for *P. vivax* by the value of *R*_0_ for *P. falciparum*. The model structure and resultant calculation of relative *R*_0_ allows us to easily make comparisons between the two diseases as well as ignore potential error in parameter values for those parameters which are shared between the two models.

In order to assess the impact of early transmission in humans on disease spread compared to other differences between *P. falciparum* and *P. vivax*, we perform a sensitivity analysis of *R*_0_ for *P. vivax*. For each parameter, we vary its value and calculate the new value of *R*_0_ to determine the effect of each parameter. We introduce parameter ε to represent the reduction in the length of the incubation period for *P. vivax* in comparison to *P. falciparum*; thus ε varies from 0 to 7 days to indicate a reduction from 14 to 7 days in the incubation period. That is, the larger ε is, the bigger a difference between *P. falciparum* and *P. vivax,* indicating earlier transmission for the latter disease. The parameter *p* represents the proportion of humans that are symptomatic in the *P. falciparum* model, and thus *p* varies from 0 to 1. In comparison, *k*_3_*p* indicates the proportion of symptomatic hosts in the *P. vivax model,* therefore, by focusing on *k*_3_ between 0 and 1, there are less symptomatic cases for *P. vivax* than for *P. falciparum*. Thus, this ensures that there are more asymptomatic cases for *P. vivax*. All other parameters are varied by 10% to create a range from 90 to 110% of the baseline value of each parameter. The more *R*_0_ changes when a parameter is varied, the more influence that parameter has on *R*_0_. In this way we can compare how much effect reducing the length of the intrinsic incubation period has on disease spread versus the role of other differences between *P. vivax* and *P. falciparum*.

Full details of the models created and the parameter values chosen as base values are presented in Additional file 5: Text S1.

## Additional files

Additional file 1: Supplemental figures. **Figure S1-S6. (PPTX 2002 kb)**

Additional file 2: Supplemental table s1. **Table S1. (PDF 1742 kb)**

Additional file 3: Supplemental table s2. **Table S2. (PDF 753 kb)**

Additional file 4: Supplemental table s3. **Table S3. (PDF 35 kb)**

Additional file 5: Supplemental methods. **Text S1.** Full details of the mathematical models of *P. falciparum* and *P. vivax* and the calculation of R_0_. (PDF 365 kb)

## Abbreviations

Amazon S3: Amazon Simple Storage Service
DDBJ: DNA Data Bank of Japan
EC2: Elastic Computing Cloud
FPKM: Fragments per kilobase of exon per million fragments mapped
GEO: Gene Expression Omnibus
HPC: High Performance Computing
NBM: Negative Binomial Model
NCBI: National Centre for Biotechnology Information
NGS: Next Generation Sequencing
*P. falciparum*: *Plasmodium falciparum*
*P. vivax*: *Plasmodium vivax*
PM: Poisson Modelling
PVM: Parasitophorous Vacuolar Membrane
RNAseq: RNA sequencing
USF: University of South Florida

## References

[1] World Health O: World malaria report 2015: World Health Organization; 2016.

[2] Battle KE, Gething PW, Elyazar IR, Moyes CL, Sinka ME, Howes RE, Guerra CA, Price RN, Baird JK, Hay SI: The global public health significance of Plasmodium vivax. 2012. 10.1016/B978-0-12-397900-1.00001-3 23199486

[3] Vogel G (2013). The forgotten malaria. Science.

[4] Sauerwein RW, Roestenberg M, Moorthy VS (2011). Experimental human challenge infections can accelerate clinical malaria vaccine development. Nat Rev Immunol.

[5] Hermsen CC, Telgt DSC, Linders EHP, van de Locht LATF, Eling WMC, Mensink EJBM, Sauerwein RW (2001). Detection of Plasmodium falciparum malaria parasites in vivo by real-time quantitative PCR. Mol Biochem Parasitol.

[6] Mikolajczak SA, Vaughan AM, Kangwanrangsan N, Roobsoong W, Fishbaugher M, Yimamnuaychok N, Rezakhani N, Lakshmanan V, Singh N, Kaushansky A (2015). Plasmodium vivax liver stage development and hypnozoite persistence in human liver-chimeric mice. Cell Host Microbe.

[7] Mueller I, Galinski MR, Baird JK, Carlton JM, Kochar DK, Alonso PL, del Portillo HA (2009). Key gaps in the knowledge of Plasmodium vivax, a neglected human malaria parasite. The Lancet infectious diseases.

[8] Lensen A, Bril A, Van De Vegte M, Van Gemert GJ, Eling W, Sauerwein R (1999). Plasmodium falciparum: infectivity of cultured, synchronized gametocytes to mosquitoes. Experimental parasitology.

[9] Carlton JM, Sina BJ, Adams JH (2011). Why is Plasmodium vivax a neglected tropical disease?. PLoS Negl Trop Dis.

[10] Bassat Q, Velarde M, Mueller I, Lin J, Leslie T, Wongsrichanalai C, Baird JK (2016). Key knowledge gaps for Plasmodium vivax control and elimination. Am. J. Trop. Med. Hyg..

[11] White NJ, Imwong M (2012). Relapse. Adv Parasitol.

[12] Noulin F, Borlon C, Van Den Abbeele J, D’Alessandro U, Erhart A (2013). 1912–2012: a century of research on Plasmodium vivax in vitro culture. Trends Parasitol.

[13] Rojas-Peña ML, Vallejo A, Herrera S, Gibson G, Arévalo-Herrera M (2015). Transcription profiling of malaria-naive and semi-immune Colombian volunteers in a Plasmodium vivax sporozoite challenge. PLoS Negl Trop Dis.

[14] Trapnell C, Roberts A, Goff L, Pertea G, Kim D, Kelley DR, Pimentel H, Salzberg SL, Rinn JL, Pachter L (2012). Differential gene and transcript expression analysis of RNA-seq experiments with TopHat and cufflinks. Nat Protoc.

[15] Arévalo-Herrera M, Forero-Peña DA, Rubiano K, Gómez-Hincapie J, Martínez NL, Lopez-Perez M, Castellanos A, Céspedes N, Palacios R, Oñate JM (2014). Plasmodium vivax sporozoite challenge in malaria-naive and semi-immune Colombian volunteers. PLoS One.

[16] López-Barragán MJ, Lemieux J, Quiñones M, Williamson KC, Molina-Cruz A, Cui K, Barillas-Mury C, Zhao K, Su X-Z (2011). Directional gene expression and antisense transcripts in sexual and asexual stages of Plasmodium falciparum. BMC Genomics.

[17] Kafsack BFC, Rovira-Graells N, Clark TG, Bancells C, Crowley VM, Campino SG, Williams AE, Drought LG, Kwiatkowski DP, Baker DA (2014). A transcriptional switch underlies commitment to sexual development in malaria parasites. Nature.

[18] Zhu L, Mok S, Imwong M, Jaidee A, Russell B, Nosten F, Day NP, White NJ, Preiser PR, Bozdech Z. New insights into the Plasmodium vivax transcriptome using RNA-Seq. Sci Rep. 2016;6(20498):srep20498. 10.1038/srep20498 PMC4746618 26858037

[19] Meibalan E, Marti M (2017). Biology of malaria transmission. Cold Spring Harb. Perspect. Med..

[20] Bruce MC, Alano P, Duthie S, Carter R (1990). Commitment of the malaria parasite Plasmodium falciparum to sexual and asexual development. Parasitology.

[21] Yamagishi J, Natori A, Tolba MEM, Mongan AE, Sugimoto C, Katayama T, Kawashima S, Makalowski W, Maeda R, Eshita Y (2014). Interactive transcriptome analysis of malaria patients and infecting Plasmodium falciparum. Genome Res.

[22] Sinden RE (1982). Gametocytogenesis of Plasmodium falciparum in vitro: an electron microscopic study. Parasitology.

[23] Tibúrcio M, Silvestrini F, Bertuccini L, Sander AF, Turner L, Lavstsen T, Alano P (2013). Early gametocytes of the malaria parasite Plasmodium falciparum specifically remodel the adhesive properties of infected erythrocyte surface. Cell Microbiol.

[24] Bousema T, Drakeley C (2011). Epidemiology and infectivity of Plasmodium falciparum and Plasmodium vivax gametocytes in relation to malaria control and elimination. Clin Microbiol Rev.

[25] Alves FP, Durlacher RR, Menezes MJ, Krieger H, Silva LHP, Camargo EP (2002). High prevalence of asymptomatic Plasmodium vivax and Plasmodium falciparum infections in native Amazonian populations. Am. J. Trop. Med. Hyg..

[26] Cheng Q, Cunningham J, Gatton ML (2015). Systematic review of sub-microscopic P. vivax infections: prevalence and determining factors. PLoS Negl Trop Dis.

[27] Adams JH, Mueller I. The Biology of Plasmodium vivax. Cold Spring Harb Perspect Med. 2017;7(9):a025585. 10.1101/cshperspect.a025585 PMC5580510 28490540

[28] Howes RE, Battle KE, Mendis KN, Smith DL, Cibulskis RE, Baird JK, Hay SI (2016). Global epidemiology of Plasmodium vivax. Am. J. Trop. Med. Hyg..

[29] Karyana M, Devine A, Kenangalem E, Burdarm L, Poespoprodjo JR, Vemuri R, Anstey NM, Tjitra E, Price RN, Yeung S (2016). Treatment-seeking behaviour and associated costs for malaria in Papua, Indonesia. Malaria journal.

[30] Ishikawa H, Ishii A, Nagai N, Ohmae H, Harada M, Suguri S, Leafasia J (2003). A mathematical model for the transmission of Plasmodium vivax malaria. Parasitol Int.

[31] White MT, Shirreff G, Karl S, Ghani AC, Mueller I (2016). Variation in relapse frequency and the transmission potential of Plasmodium vivax malaria. Proc R Soc B.

[32] Barbosa S, Gozze AB, Lima NF, Batista CL, da Silva Bastos M, Nicolete VC, Fontoura PS, Gonçalves RM, Viana SAS, Menezes MJ (2014). Epidemiology of disappearing Plasmodium vivax malaria: a case study in rural Amazonia. PLoS Negl Trop Dis.

[33] Olliaro PL, Barnwell JW, Barry A, Mendis K, Mueller I, Reeder JC, Shanks GD, Snounou G, Wongsrichanalai C (2016). Implications of Plasmodium vivax biology for control, elimination, and research. Am. J. Trop. Med. Hyg..

[34] Koella JC, Antia R (1995). Optimal pattern of replication and transmission for parasites with two stages in their life cycle. Theor Popul Biol.

[35] Mayxay M, Pukrittayakamee S, Newton PN, White NJ (2004). Mixed-species malaria infections in humans. Trends Parasitol.

[36] De Zoysa A, Mendis C, Gamage-Mendis A, Weerasinghe S, Herath P, Mendis KN (1991). A mathematical model for Plasmodium vivax malaria transmission: estimation of the impact of transmission-blocking immunity in an endemic area. Bull World Health Organ.

[37] Hong C, Manimaran S, Shen Y, Perez-Rogers JF, Byrd AL, Castro-Nallar E, Crandall KA, Johnson WE (2014). PathoScope 2.0: a complete computational framework for strain identification in environmental or clinical sequencing samples. Microbiome.

[38] Langmead B, Salzberg SL (2012). Fast gapped-read alignment with bowtie 2. Nat Methods.

[39] Kim D, Pertea G, Trapnell C, Pimentel H, Kelley R, Salzberg SL (2013). TopHat2: accurate alignment of transcriptomes in the presence of insertions, deletions and gene fusions. Genome Biol.

[40] Anjum A, Jaggi S, Varghese E, Lall S, Bhowmik A, Rai A (2016). Identification of differentially expressed genes in RNA-seq data of Arabidopsis thaliana: a compound distribution approach. J Comput Biol.

[41] Hebenstreit D, Fang M, Gu M, Charoensawan V, van Oudenaarden A, Teichmann SA (2011). RNA sequencing reveals two major classes of gene expression levels in metazoan cells. Mol Syst Biol.

[42] Soneson C, Delorenzi M (2013). A comparison of methods for differential expression analysis of RNA-seq data. BMC bioinformatics.

[43] Anders S, Huber W (2010). Differential expression analysis for sequence count data. Genome Biol.

[44] Cumbie JS, Kimbrel JA, Di Y, Schafer DW, Wilhelm LJ, Fox SE, Sullivan CM, Curzon AD, Carrington JC, Mockler TC (2011). GENE-counter: a computational pipeline for the analysis of RNA-Seq data for gene expression differences. PLoS One.

[45] Zhang ZH, Jhaveri DJ, Marshall VM, Bauer DC, Edson J, Narayanan RK, Robinson GJ, Lundberg AE, Bartlett PF, Wray NR (2014). A comparative study of techniques for differential expression analysis on RNA-Seq data. PLoS One.

[46] Chen F, Mackey AJ, Stoeckert CJ, Roos DS (2006). OrthoMCL-DB: querying a comprehensive multi-species collection of ortholog groups. Nucleic Acids Res.

[47] Macdonald G (1952). The analysis of equilibrium in malaria. Tropical diseases bulletin.

[48] Macdonald G (1961). Epidemiologic models in studies of vetor-borne diseases: the re dyer lecture. Public Health Rep.

[49] Ross R (1911). The prevention of malaria.

[50] White MT, Karl S, Battle KE, Hay SI, Mueller I, Ghani AC (2014). Modelling the contribution of the hypnozoite reservoir to Plasmodium vivax transmission. Elife.

[51] White NJ (2011). Determinants of relapse periodicity in Plasmodium vivax malaria. Malar J.

