# Supplementary material for: Plasmodium vivax readiness to transmit: implication for malaria eradication
Source: BMC Syst Biol. 2019 Jan 11;13:5. doi: 10.1186/s12918-018-0669-4 (PMC6330404; doi:10.1186/s12918-018-0669-4)
Supplement: Supplementary file 1 — Supplemental figures. Figure S1-S6. (PPTX 2002 kb) [file 12918_2018_669_MOESM1_ESM.pptx]

## Slide 1
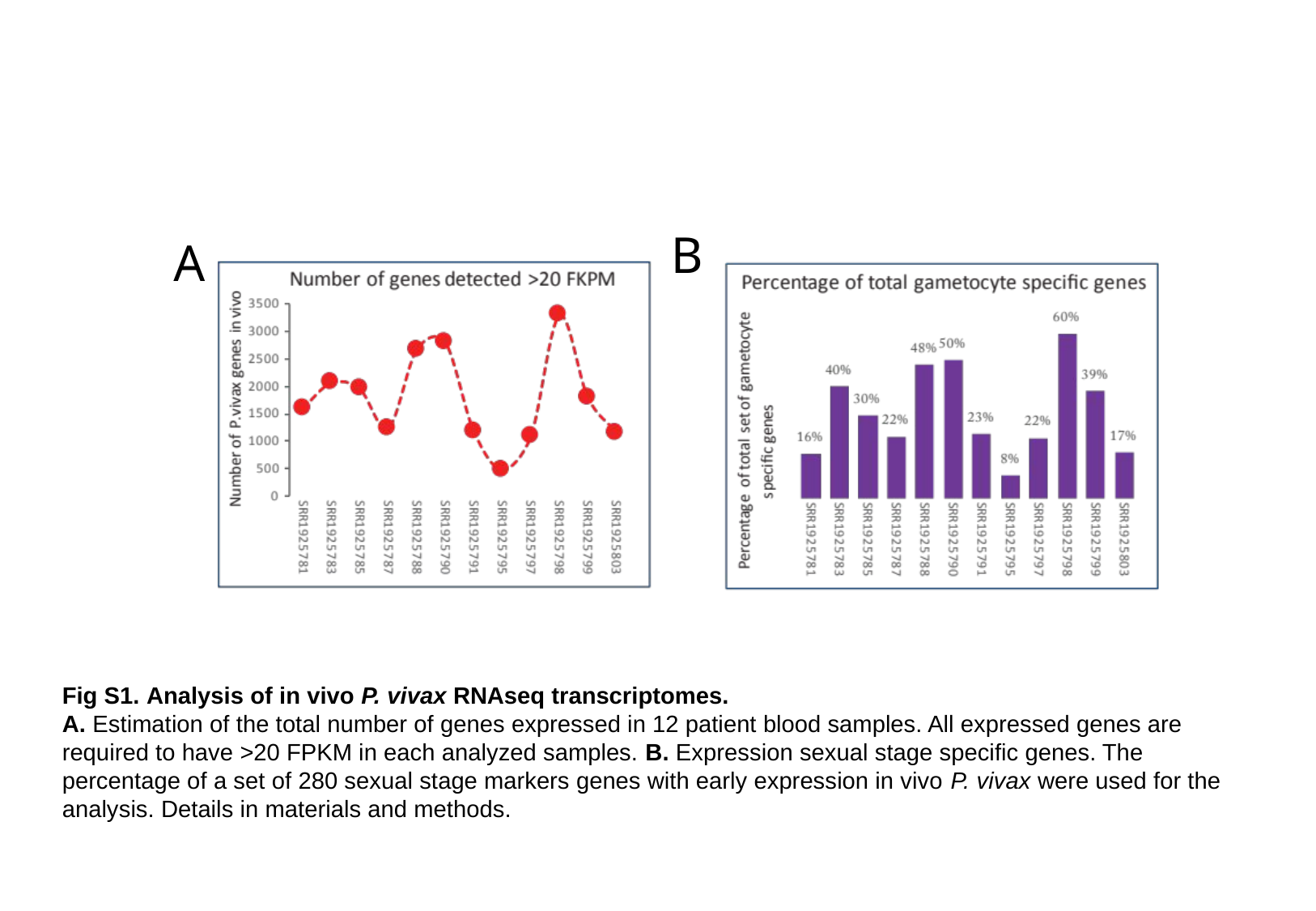

B
A
Fig S1. Analysis of in vivo P. vivax RNAseq transcriptomes.
A. Estimation of the total number of genes expressed in 12 patient blood samples. All expressed genes are required to have >20 FPKM in each analyzed samples. B. Expression sexual stage specific genes. The percentage of a set of 280 sexual stage markers genes with early expression in vivo P. vivax were used for the analysis. Details in materials and methods.

## Slide 2
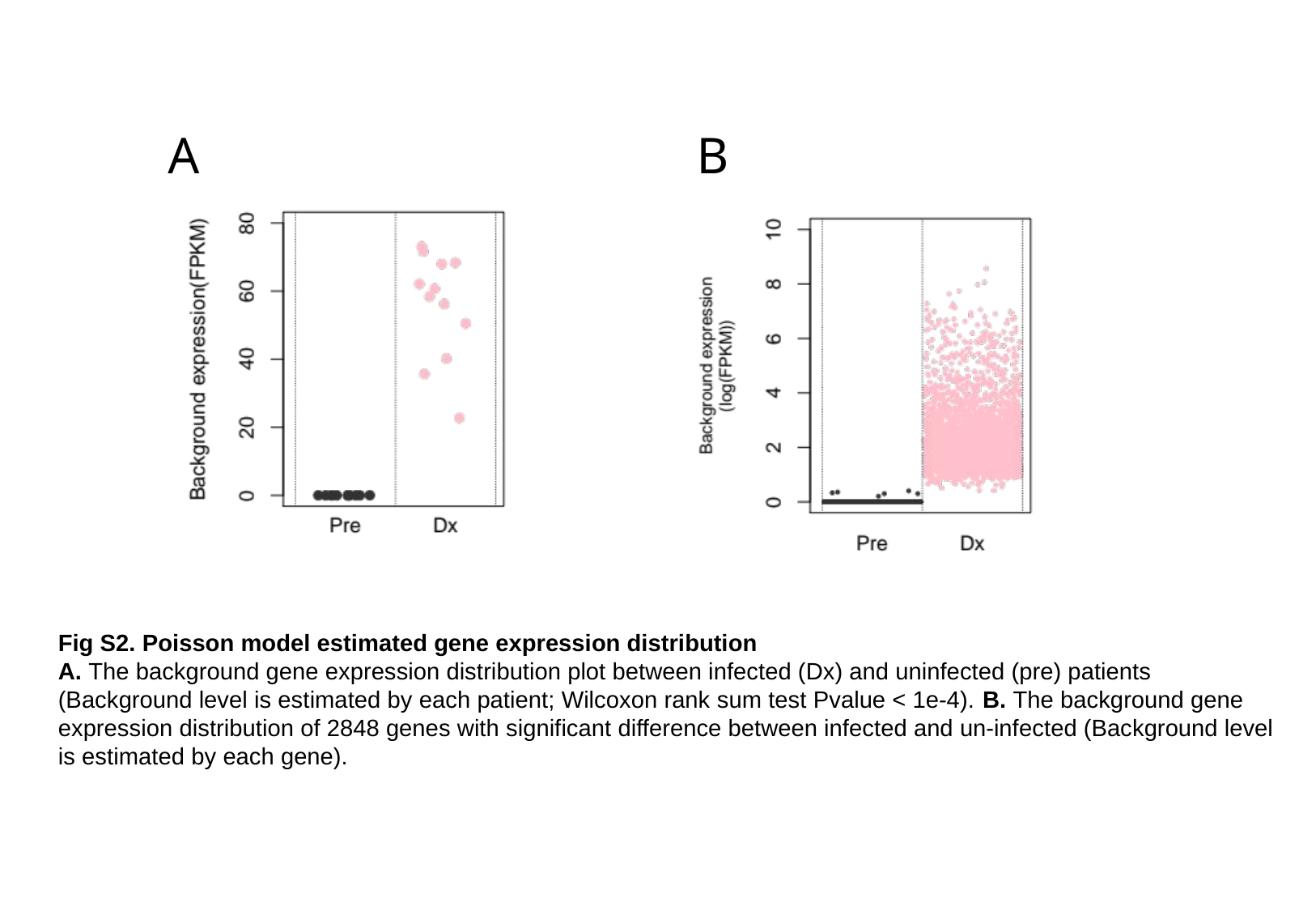

A
B
Fig S2. Poisson model estimated gene expression distribution
A. The background gene expression distribution plot between infected (Dx) and uninfected (pre) patients (Background level is estimated by each patient; Wilcoxon rank sum test Pvalue < 1e-4). B. The background gene expression distribution of 2848 genes with significant difference between infected and un-infected (Background level is estimated by each gene).

## Slide 3
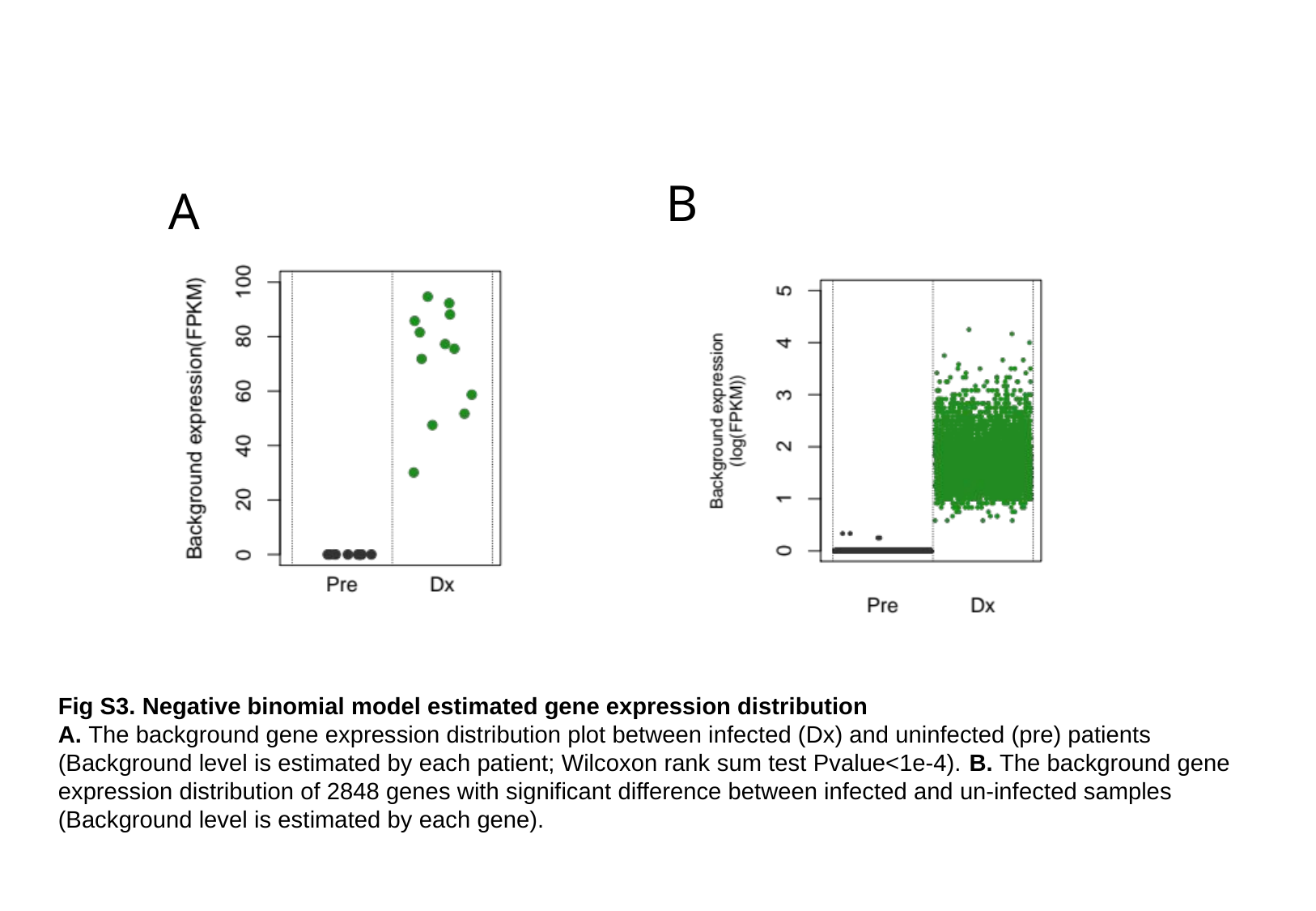

B
A
Fig S3. Negative binomial model estimated gene expression distribution
A. The background gene expression distribution plot between infected (Dx) and uninfected (pre) patients (Background level is estimated by each patient; Wilcoxon rank sum test Pvalue<1e-4). B. The background gene expression distribution of 2848 genes with significant difference between infected and un-infected samples (Background level is estimated by each gene).

## Slide 4
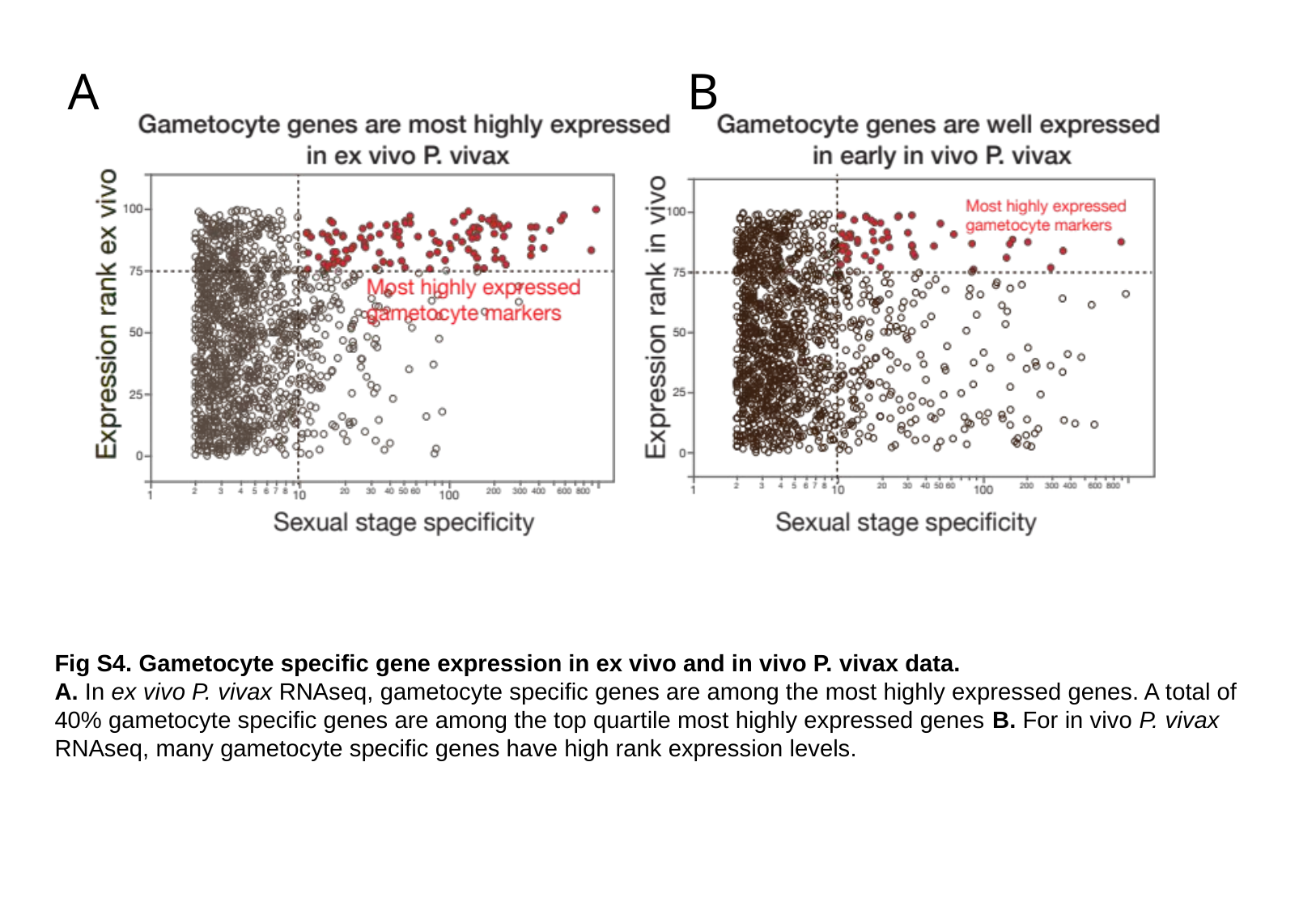

A
B
Fig S4. Gametocyte specific gene expression in ex vivo and in vivo P. vivax data.
A. In ex vivo P. vivax RNAseq, gametocyte specific genes are among the most highly expressed genes. A total of 40% gametocyte specific genes are among the top quartile most highly expressed genes B. For in vivo P. vivax RNAseq, many gametocyte specific genes have high rank expression levels.

## Slide 5
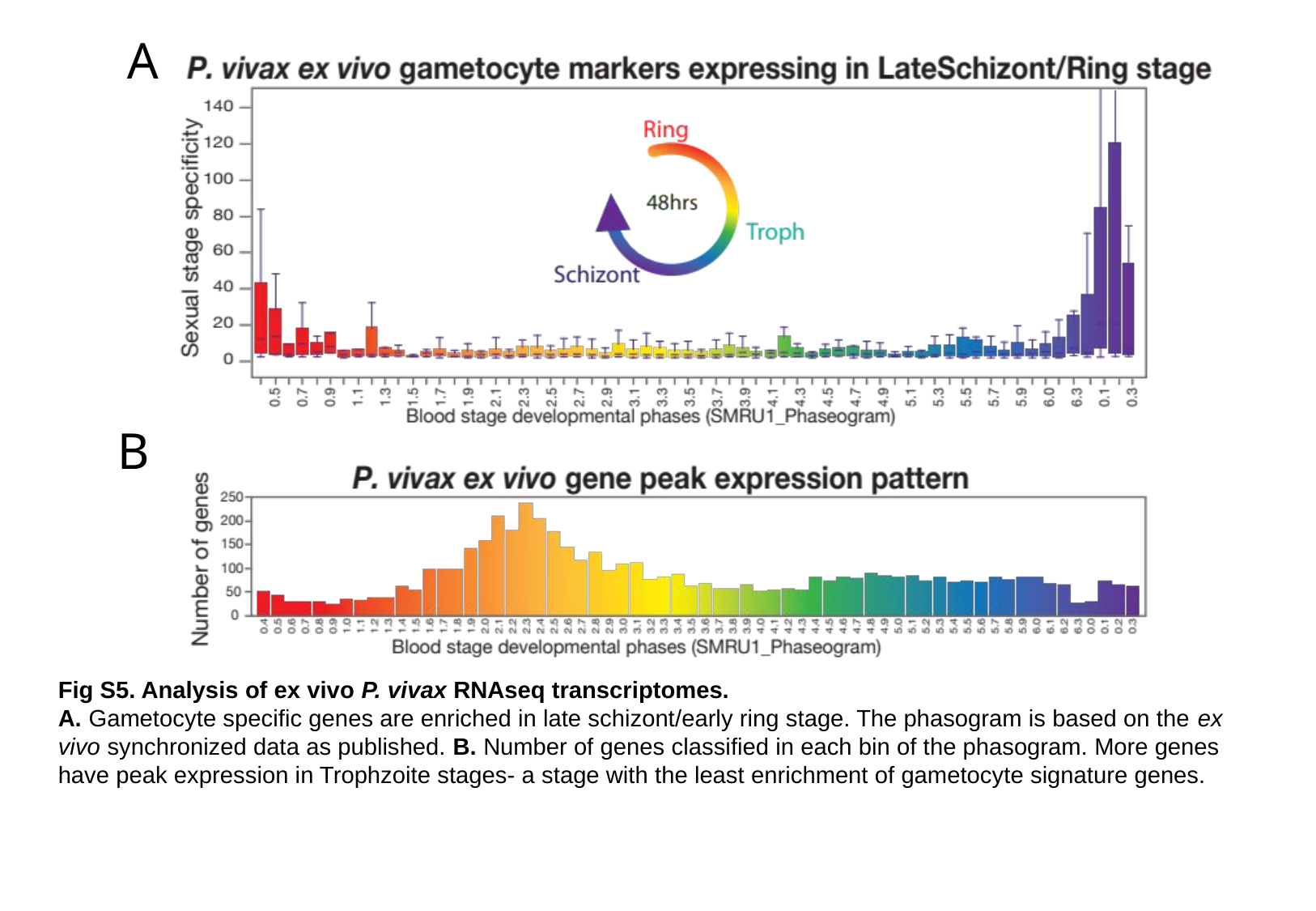

A
B
Fig S5. Analysis of ex vivo P. vivax RNAseq transcriptomes.
A. Gametocyte specific genes are enriched in late schizont/early ring stage. The phasogram is based on the ex vivo synchronized data as published. B. Number of genes classified in each bin of the phasogram. More genes have peak expression in Trophzoite stages- a stage with the least enrichment of gametocyte signature genes.

## Slide 6
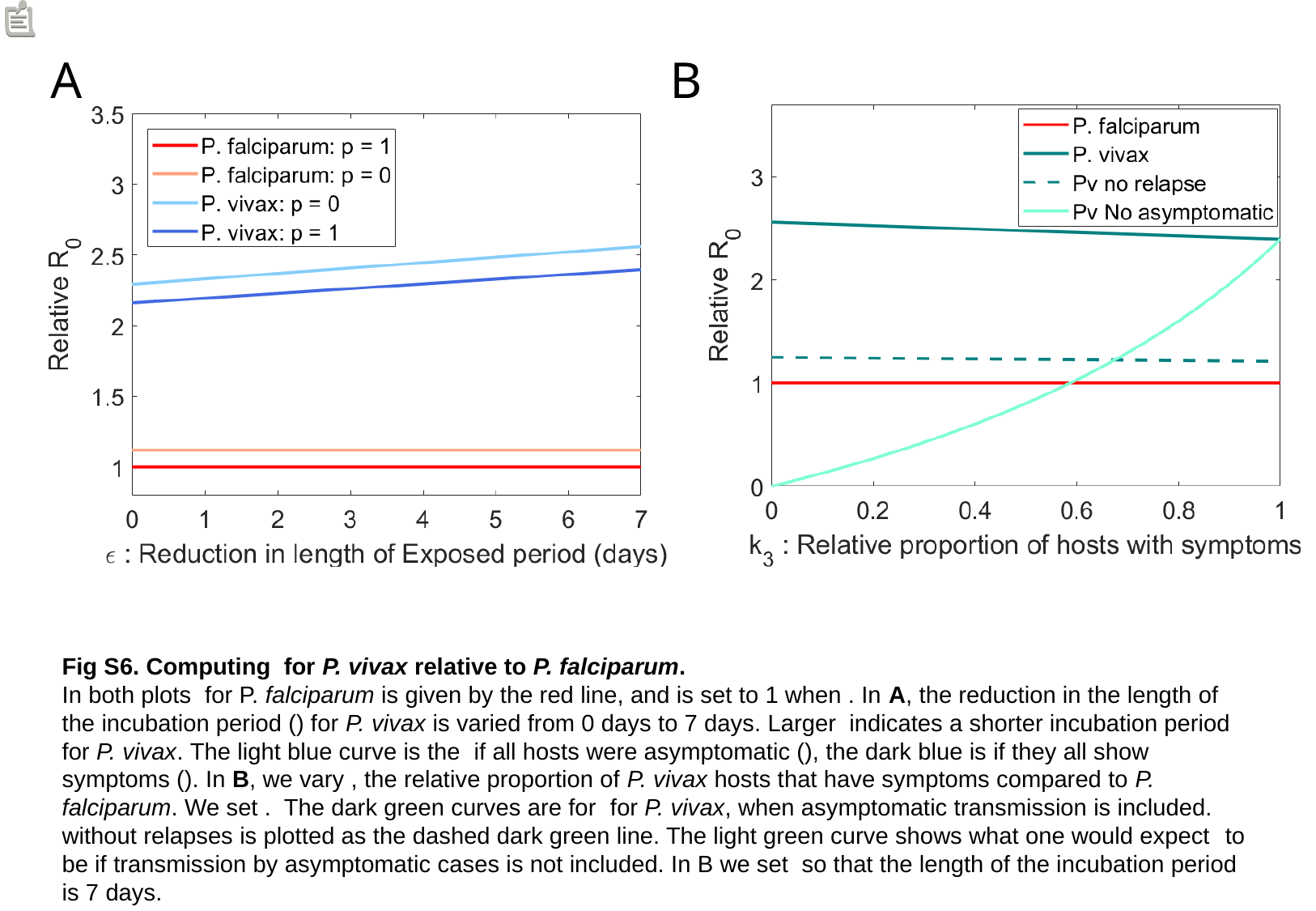

A
B
