# Supplementary material for: Plasmodium vivax readiness to transmit: implication for malaria eradication
Source: BMC Syst Biol. 2019 Jan 11;13:5. doi: 10.1186/s12918-018-0669-4 (PMC6330404; doi:10.1186/s12918-018-0669-4)
Supplement: Supplementary file 2 — Supplemental table s1. Table S1. (PDF 1742 kb) [file 12918_2018_669_MOESM2_ESM.pdf]

S1 Table

SRR Number	SRR1925783	SRR1925785	SRR1925803	SRR1925797	SRR1925781	SRR1925795	SRR1925787	SRR1925799	SRR1925788	SRR1925790	SRR1925798	SRR1925791	
Parasite Density (Parasites/µL)	6	10	20	25	34	34	38	55	95	110	216	390	
Parasitemia Classification	low	low	low	low	medium	medium	medium	medium	high	high	high	high	
Patient Identifier	317N-Dx	314N-Dx	375S-Dx	327S-Dx	302N-Dx	324S-Dx	319N-Dx	301S-Dx	306N-Dx	310N-Dx	341S-Dx	302S-Dx	
Patient Location	Cali	Cali	Buenaventura	Buenaventura	Cali	Buenaventura	Cali	Buenaventura	Cali	Cali	Buenaventura	Buenaventura	
Reads Mapped to Parasite	2597	3316	856	810	7966	386	1091	1689	6821	8020	12165	1406	
Gene ID / Patient	Patient 1	Patient 2	Patient 3	Patient 4	Patient 5	Patient 6	Patient 7	Patient 8	Patient 9	Patient 10	Patient 11	Patient 12	Gene Description
PlvioMp1	0	0	0	0	0	0	0	0	0	0	0	0	unspecified product
PlvioMp2	0	0	0	0	0	0	0	0	0	0	0	0	unspecified product
PlvioMp3	0	0	0	0	0	0	0	0	0	33.2406	0	0	unspecified product
PVX_000000	0	0	0	0	0	0	0	0	0	0	0	0	Plasmodium exported protein, unknown function
PVX_000005	101.036	70.5823	0	0	0	0	0	0	76.5888	35.4911	20.1123	163.824	Plasmodium exported protein, unknown function
PVX_000010	2313.41	1730.52	1369.84	880.362	873.674	1691.34	842.405	1376.96	2066.14	2612.64	1655.54	891.954	Plasmodium exported protein, unknown function
PVX_000015	0	164.8	0	0	0	402.833	0	0	0	0	46.9717	0	Pv-fam-d protein
PVX_000020	0	0	0	0	0	0	0	0	0	0	0	0	variable surface protein Vir9-like,PIR protein
PVX_000025	0	0	0	0	0	0	0	0	0	0	0	0	hypothetical protein
PVX_000525	0	0	0	0	0	0	0	0	0	0	8.75013	0	protein kinase domain containing protein
PVX_000530	0	4.02029	0	0	1.82567	0	8.79847	0	0	0	4.58736	0	hypothetical protein, conserved
PVX_000535	60.4785	42.2273	0	193.355	0	0	0	0	0	42.4974	12.0383	0	thioredoxin, putative
PVX_000540	0	0	0	0	7.59378	0	0	0	0	0	0	0	hypothetical protein, conserved
PVX_000545	93.7714	0	0	149.84	44.612	0	0	260.443	177.639	49.4223	130.654	75.9067	serine--tRNA ligase, putative
PVX_000550	0	23.1284	82.3581	0	0	0	0	0	0	11.6439	26.3823	0	hypothetical protein, conserved
PVX_000555	0	0	160.537	0	0	0	98.7279	0	0	0	25.6913	0	calcium-dependent protein kinase 4, putative (CDPK4)
PVX_000560	0	0	0	0	14.395	0	0	0	17.1961	31.8957	36.137	0	queuine tRNA-ribosyltransferase, putative
PVX_000565	60.3968	42.1703	0	0	0	0	92.386	83.885	22.885	21.22	36.066	0	hypothetical protein, conserved
PVX_000570	0	0	0	0	0	0	0	0	0	0	63.7946	0	hypothetical protein, conserved
PVX_000575	0	28.36	100.999	259.637	0	0	0	0	30.7846	14.2758	32.3472	65.764	hypothetical protein, conserved
PVX_000580	88.7165	0	0	283.86	0	0	135.828	0	201.733	155.828	123.618	143.799	hypothetical protein, conserved
PVX_000585	116.869	81.6597	0	0	0	0	0	0	44.3002	41.0496	69.7938	0	transporter/permease protein, putative
PVX_000590	573.119	320.354	856.628	367.022	36.4155	0	175.638	159.286	651.727	483.136	616.074	371.854	eukaryotic translation initiation factor 3 subunit 2, putative
PVX_000595	0	0	0	0	0	0	0	0	12.0449	0	18.9853	0	hypothetical protein, conserved
PVX_000600	116.774	40.7661	0	186.658	55.5714	0	89.3063	81.0922	88.4931	41.0283	11.622	0	cysteine desulfurase, putative (SufS)
PVX_000604	149.435	208.715	0	0	71.1426	0	228.688	207.579	56.6282	104.998	133.855	121.06	hypothetical protein, conserved
PVX_000606	0	0	0	0	90.4174	0	0	0	0	0	112.865	0	transcription initiation factor IIA subunit 1, putative
PVX_000610	221.278	115.868	550.312	176.836	87.746	0	169.211	230.488	419.208	272.113	418.429	89.582	hypothetical protein, conserved
PVX_000615	0	0	0	0	21.0237	339.184	0	0	50.211	0	13.188	0	hypothetical protein, conserved
PVX_000620	0	43.6381	77.6934	99.863	39.6452	0	47.7744	43.4063	59.2144	54.9253	56.001	0	protein SDA1, putative (SDA1)
PVX_000625	24.9372	52.211	0	159.293	15.8101	0	0	103.869	28.34	26.2892	29.7811	0	RNA-binding protein, putative
PVX_000630	159.702	111.535	0	0	101.387	0	0	0	151.302	112.209	95.3685	129.396	phosphopantothenate--cysteine ligase, putative,phosphopantothenoylcysteine synthetase, putative
PVX_000635	0	0	0	0	0	0	0	0	0	35.733	60.7486	0	hypothetical protein, conserved
PVX_000640	107.985	0	0	0	0	0	0	150.05	122.79	37.9308	64.4875	0	30S ribosomal protein S12, putative
PVX_000645	0	0	0	0	0	0	0	0	0	0	19.5242	0	hypothetical protein, conserved
PVX_000650	17.6385	0	0	56.324	16.7711	0	26.944	97.9511	20.0444	18.5955	35.1075	28.5328	DNA polymerase alpha, putative
PVX_000660	7.86427	0	0	0	0	0	0	0	2.97878	2.76379	10.9569	0	hypothetical protein
PVX_000670	0	0	50.9655	0	0	0	0	0	0	7.20857	24.4975	0	hypothetical protein, conserved
PVX_000675	0	0	0	0	0	0	0	0	0	0	0	0	hypothetical protein
PVX_000677	0	0	0	0	0	0	0	0	0	0	0	0	tRNA Glutamic acid
PVX_000678	0	0	0	0	0	0	0	0	0	0	0	0	tRNA Alanine
PVX_000680	0	0	0	0	16.8532	0	0	0	20.1394	0	10.5816	43.0102	RNA helicase, putative
PVX_000685	53.6288	0	266.734	0	0	0	0	0	0	56.5283	53.3737	0	hypothetical protein, conserved
PVX_000690	29.0209	0	0	0	0	0	0	0	43.9759	10.1979	28.8819	0	hypothetical protein, conserved
PVX_000695	0	0	0	0	0	0	0	0	0	78.5905	44.5709	0	hypothetical protein, conserved
PVX_000700	0	0	0	0	0	0	0	46.1647	0	0	19.8529	0	hypothetical protein, conserved
PVX_000705	39.9211	55.7327	0	0	25.3191	0	61.0255	0	45.3733	42.0824	31.7844	0	hypothetical protein, conserved
PVX_000710	0	15.182	0	0	6.89564	0	0	0	0	7.64484	17.3202	0	hypothetical protein, conserved
PVX_000715	0	29.823	0	0	13.5489	0	0	0	16.1861	30.0233	17.0075	138.317	ribosome biogenesis GTPase A, putative (RbgA)
PVX_000720	172.975	60.4075	0	0	54.9154	443.035	0	120.153	163.884	182.297	120.512	0	hypothetical protein, conserved
PVX_000725	0	0	0	0	80.4804	0	0	0	96.0032	44.4742	75.6211	205.464	hypothetical protein, conserved
PVX_000730	107.209	74.9005	0	0	34.0536	0	164.237	148.971	0	37.6583	106.706	0	exosome complex component RRP4, putative (RRP4)
PVX_000735	33.6599	23.4939	0	0	10.6724	0	51.444	0	25.5035	11.8278	46.8984	0	protein phosphatase 2C, putative

Gene ID / Patient	Patient 1	Patient 2	Patient 3	Patient 4	Patient 5	Patient 6	Patient 7	Patient 8	Patient 9	Patient 10	Patient 11	Patient 12	Gene Description
PVX_000737	0	0	0	0	0	0	0	0	0	0	0	0	tRNA Isoleucine
PVX_000738	0	0	0	0	0	0	0	0	0	0	0	0	tRNA Serine
PVX_000745	38.3309	214.047	0	122.471	0	0	58.5917	0	14.5218	0	22.8887	0	hypothetical protein, conserved
PVX_000750	0	0	0	0	0	0	0	0	0	0	0	0	actin-like protein, putative
PVX_000755	64.6742	180.637	160.885	206.794	0	331.08	0	0	98.026	90.8895	51.494	0	zinc finger protein, putative
PVX_000760	0	0	0	0	0	0	0	0	0	0	0	0	hypothetical protein, conserved
PVX_000765	0	17.6746	0	80.8872	16.0563	258.984	0	0	38.3749	35.5978	45.367	40.9762	replication factor a protein, putative
PVX_000770	0	0	0	0	28.5068	0	0	124.738	68.0545	0	17.8723	0	hypothetical protein, conserved
PVX_000775	0	71.66	0	0	65.1182	262.623	0	0	136.117	108.195	61.2935	83.0997	chaperone DNAJ protein, putative
PVX_000780	36.6813	25.6038	0	234.39	0	0	0	50.9347	41.6901	25.7785	36.506	59.369	hypothetical protein, conserved
PVX_000785	69.0132	48.1918	0	0	21.9007	0	0	95.8604	0	72.7389	13.7373	0	ribosomal processing protein, putative
PVX_000790	50.8556	35.504	0	0	16.1313	0	0	0	0	17.8686	40.4908	0	U4/U6 small nuclear ribonucleoprotein PRP31, putative
PVX_000795	0	0	0	46.8692	4.652	0	0	0	11.1205	0	20.4516	23.7432	hypothetical protein, conserved
PVX_000800	46.8121	0	116.393	149.605	14.8473	0	0	0	17.736	65.793	55.9069	0	tRNA N6-adenosine threonylcarbamoyltransferase, putative (KAE1)
PVX_000805	0	0	0	0	0	0	0	0	0	36.9939	20.9645	0	hypothetical protein, conserved
PVX_000810	0	0	0	0	0	0	0	0	0	0	0	0	perforin-like protein 1 (PLP1)
PVX_000815	0	0	0	0	0	0	0	0	0	0	0	0	sporozoite invasion-associated protein 1, putative (SIAP1)
PVX_000820	0	0	0	0	12.6765	0	0	0	45.4341	14.0463	47.7405	0	flap endonuclease 1, putative (FEN1)
PVX_000825	66.4017	0	0	0	0	0	0	0	50.3228	69.9873	52.8696	0	hypothetical protein, conserved
PVX_000830	106.444	0	0	0	0	0	0	0	40.3455	74.7794	63.5669	0	hypothetical protein, conserved
PVX_000835	0	0	143.717	0	0	0	0	0	0	0	11.5019	0	hypothetical protein, conserved
PVX_000840	0	21.8879	0	0	0	0	0	0	11.8802	0	12.4839	0	hypothetical protein, conserved
PVX_000845	0	0	0	894.713	0	0	0	0	0	0	0	0	hypothetical protein, conserved
PVX_000850	94.2157	0	117.129	0	14.9412	0	0	0	0	16.5521	84.39	0	AAA family ATPase, putative
PVX_000855	0	0	0	0	0	0	0	0	0	0	7.70488	0	hypothetical protein, conserved
PVX_000860	0	0	0	0	0	0	0	0	0	0	4.166	0	hypothetical protein, conserved
PVX_000865	0	0	0	0	0	0	0	0	0	0	0	0	hypothetical protein, conserved
PVX_000870	0	0	0	0	0	0	0	0	36.0925	33.4527	56.8695	0	mitochondrial carrier protein, putative
PVX_000875	0	0	0	0	0	0	0	0	0	0	0	0	hypothetical protein, conserved
PVX_000880	0	127.98	0	0	19.385	0	0	84.8591	23.1507	42.9325	60.8078	0	hypothetical protein, conserved
PVX_000885	0	77.5038	0	0	35.2387	568.659	169.957	0	84.0943	38.9646	66.2463	179.916	peptidyl-tRNA hydrolase, putative
PVX_000890	75.0937	52.4418	186.86	240.18	0	0	0	104.312	56.9134	79.1452	74.7386	0	methyltransferase, putative
PVX_000895	0	94.4159	0	0	0	0	0	93.9039	25.6178	23.7519	53.8282	0	hypothetical protein, conserved
PVX_000900	0	0	0	0	0	0	0	18.4284	0	0	0	21.4708	hypothetical protein, conserved
PVX_000905	0	0	340.704	0	0	0	0	0	51.8174	96.0077	163.256	0	hypothetical protein, conserved
PVX_000910	0	0	0	0	0	0	0	0	0	0	14.9895	0	hypothetical protein, conserved
PVX_000915	386.15	0	0	0	0	0	0	0	0	0	153.793	0	hypothetical protein, conserved
PVX_000920	0	22.215	0	0	0	162.775	0	44.1938	0	22.3685	44.3464	0	hypothetical protein, conserved
PVX_000925	488.53	85.3431	0	0	38.8081	0	0	339.464	92.5939	171.588	72.9375	198.148	hydroxyacyl glutathione hydrolase, putative
PVX_000930	0	0	0	0	0	0	0	0	48.6911	45.1121	51.1377	0	sexual stage antigen s16, putative
PVX_000935	349.104	48.7562	521.141	223.282	22.1574	0	213.667	0	132.288	98.1198	125.082	113.111	vacuolar ATP synthase subunit b, putative
PVX_000940	48.8833	68.2524	0	156.233	0	0	74.7469	0	55.5629	17.1758	126.491	158.29	hypothetical protein, conserved
PVX_000945	0	0	0	135.163	0	0	0	0	0	0	0	0	apical sushi protein, putative (ASP)
PVX_000950	221.311	0	0	354.275	35.1513	0	0	153.765	41.9431	0	44.055	0	hypothetical protein, conserved
PVX_000955	74.8303	73.1045	37.178	47.7867	9.4861	76.5001	0	20.7771	51.0214	36.816	41.7035	24.208	lysine decarboxylase, putative
PVX_000960	240.329	167.932	0	0	0	0	0	0	91.1008	0	95.6827	0	secy-independent transporter protein, putative
PVX_000965	0	0	246.299	0	62.825	0	0	0	74.9793	34.7462	39.3799	0	hypothetical protein, conserved
PVX_000970	40.4182	28.2025	0	64.5188	16.0098	0	46.295	84.1635	30.6192	21.3064	50.2795	32.6842	pre-mRNA-processing-splicing factor 8, putative
PVX_000975	10.503	7.32869	26.0878	0	6.6565	0	0	0	0	0	22.9954	33.9734	liver specific protein 2, putative (LISP2)
PVX_000980	0	0	0	0	0	0	0	0	0	0	0	0	Plasmodium yoelii blood stage membrane protein ag-1, putative
PVX_000985	20.0579	13.9975	49.834	128.108	19.0726	205.086	0	55.6947	60.7844	56.3891	35.9309	32.4487	protein transport protein Sec24B, putative (SEC24B)
PVX_000990	61.9865	86.5621	0	0	0	0	0	86.0942	70.4629	65.3351	24.6769	0	ATP-dependent RNA helicase protein, putative
PVX_000995	0	0	0	0	0	0	0	0	0	0	0	0	6-cysteine protein (P41)
PVX_001000	0	0	0	0	0	0	0	0	0	0	9.87575	0	hypothetical protein, conserved
PVX_001005	0	0	0	0	13.6074	0	0	0	0	0	0	0	dipeptidyl aminopeptidase 3, putative (DPAP3)
PVX_001010	0	0	0	0	0	0	0	0	0	0	0	0	hypothetical protein, conserved
PVX_001015	0	0	0	0	0	0	0	0	0	0	0	0	hypothetical protein, conserved
PVX_001020	0	0	0	0	0	0	0	0	0	0	0	0	6-cysteine protein (P52)
PVX_001025	0	0	0	0	0	0	0	0	0	0	0	0	6-cysteine protein (P36)
PVX_001030	0	59.6744	106.263	0	27.1107	0	196.035	118.711	64.7753	45.0564	144.633	0	hypothetical protein, conserved
PVX_001035	273.048	0	0	0	87.0847	0	0	0	207.224	95.8312	54.3655	0	hypothetical protein, conserved
PVX_001040	0	0	0	0	0	0	0	0	0	0	1.70703	0	transcription factor with AP2 domain(s), putative (ApiAP2)

Gene ID / Patient	Patient 1	Patient 2	Patient 3	Patient 4	Patient 5	Patient 6	Patient 7	Patient 8	Patient 9	Patient 10	Patient 11	Patient 12	Gene Description
PVX_001045	0	0	0	0	0	0	0	0	0	0	78.0146	0	hypothetical protein, conserved
PVX_001050	0	0	0	0	0	0	25.9759	0	19.3244	17.9277	3.38467	27.5076	SET domain protein, putative (SET8)
PVX_001055	0	0	0	0	0	0	0	0	0	39.0034	14.7292	0	alpha/beta hydrolase, putative
PVX_001060	136.163	63.3689	0	0	14.395	0	0	0	34.3922	0	45.1713	0	splicing factor, putative,CGI-201 protein, short form, putative,crooked neck-like protein 1, putative
PVX_001065	0	0	93.2435	0	0	0	0	0	0	0	29.8659	0	hypothetical protein, conserved
PVX_001070	0	0	0	0	0	0	0	0	0	0	0	0	hypothetical protein
PVX_001075	0	0	0	0	0	0	0	0	0	0	0	0	ubiquitin specific protease, putative
PVX_001080	0	0	0	0	0	0	0	22.8334	6.23023	8.6708	1.63691	13.3003	hypothetical protein, conserved
PVX_001085	0	0	0	0	116.883	0	0	0	0	0	145.621	0	hypothetical protein, conserved
PVX_001090	0	44.0443	0	0	20.0145	0	96.4966	0	23.9017	44.324	50.2236	0	hypothetical protein, conserved
PVX_001095	0	0	0	0	13.8062	0	0	0	0	76.4818	60.6556	70.4723	hypothetical protein, conserved
PVX_001097	0	0	0	0	0	0	0	0	0	0	0	0	tRNA Asparagine
PVX_001100	0	0	0	0	0	0	0	0	0	0	0	0	RAD protein (Pv-fam-e)
PVX_001105	0	0	0	0	0	0	0	0	0	31.8601	18.0535	0	Plasmodium exported protein, unknown function, pseudogene
PVX_001110	0	0	0	0	0	0	0	0	0	0	0	0	hypothetical protein
PVX_001610	0	0	0	0	0	0	0	0	0	0	18.4844	0	variable surface protein Vir25-related,PIR protein
PVX_001615	0	0	0	0	0	0	0	0	0	0	0	0	variable surface protein Vir12/24-related,PIR protein
PVX_001620	0	0	0	0	0	0	0	0	0	0	0	0	hypothetical protein
PVX_001625	0	0	0	0	0	0	0	0	0	0	15.0998	0	variable surface protein Vir12-related,PIR protein
PVX_001630	0	0	0	0	0	0	0	0	0	26.3155	14.9102	0	VIR protein, pseudogene,PIR protein, pseudogene
PVX_001635	0	0	0	0	0	0	0	0	0	0	9.84301	0	VIR protein,PIR protein
PVX_001640	0	0	0	0	21.4047	0	0	0	0	0	0	0	VIR protein,PIR protein
PVX_001645	0	17.5228	0	0	15.923	0	0	0	0	0	19.9842	0	variable surface protein Vir12-related,PIR protein
PVX_001650	43.7459	152.688	0	0	27.7475	223.803	66.8804	0	66.2956	61.4844	52.2447	70.8172	Pv-fam-d protein
PVX_001655	0	0	0	0	0	0	0	0	77.3628	143.224	0	0	hypothetical protein
PVX_001660	0	239.085	0	0	0	0	0	0	0	0	0	0	Plasmodium exported protein, unknown function, pseudogene
PVX_001670	252.556	564.214	251.206	484.33	176.241	258.466	154.48	280.592	382.759	532.429	442.382	572.492	Plasmodium exported protein, unknown function
PVX_001675	0	0	0	0	0	0	0	0	0	0	22.4721	0	Phist protein (Pf-fam-b)
PVX_001680	0	0	0	0	0	0	0	0	0	0	0	0	Phist protein (Pf-fam-b)
PVX_001685	346.704	363.101	143.717	184.727	73.3286	591.493	88.3819	401.27	569.26	426.348	149.525	93.5795	Phist protein (Pf-fam-b)
PVX_001690	78.6577	0	0	0	0	0	0	0	0	0	0	0	Phist protein (Pf-fam-b)
PVX_001695	0	0	0	0	0	0	0	0	0	0	0	0	Phist protein (Pf-fam-b)
PVX_001700	0	0	0	0	0	0	0	0	0	0	36.9688	0	Phist protein (Pf-fam-b)
PVX_001705	0	0	0	0	0	0	0	0	34.2334	0	17.9806	0	Phist protein (Pf-fam-b)
PVX_001710	0	99.1691	0	0	0	728.008	0	197.218	0	49.8291	56.4896	0	Phist protein (Pf-fam-b)
PVX_001715	0	0	0	0	0	0	0	0	0	0	0	0	early transcribed membrane protein (ETRAMP) (ETRAMP10.3)
PVX_001720	144.917	0	144.055	0	36.7542	0	0	80.4849	32.9392	40.7388	92.3024	46.8996	DNA polymerase delta catalytic subunit, putative
PVX_001725	0	0	0	0	0	0	0	0	27.6896	0	0	0	rhoptry neck protein 12, putative (RON12)
PVX_001730	67.1491	0	0	0	0	0	0	0	0	0	53.4647	0	enoyl-CoA hydratase-related protein, putative
PVX_001735	0	0	0	0	0	0	0	79.4838	21.6845	0	34.1747	185.361	golgi re-assembly stacking protein, putative (GRASP)
PVX_001740	0	0	0	0	0	0	0	0	0	0	33.1305	0	phosphomannomutase, putative
PVX_001745	0	0	0	0	0	0	0	0	358.201	0	0	0	hypothetical protein, conserved
PVX_001750	104.077	36.3303	129.406	166.332	0	0	159.159	0	19.7168	54.8524	93.2234	0	hypothetical protein, conserved
PVX_001752	0	0	0	0	0	0	0	0	0	0	0	0	hypothetical protein
PVX_001755	0	47.1475	83.9447	0	21.4173	0	0	0	12.7951	11.8679	13.4451	0	hypothetical protein, conserved
PVX_001760	74.5308	52.0484	185.456	0	94.6193	0	0	103.53	141.217	104.736	89.014	0	26S proteasome regulatory subunit p55, putative
PVX_001765	81.2279	0	0	0	0	0	0	0	30.7826	0	0	0	tRNA pseudouridine synthase, putative
PVX_001770	0	0	0	0	0	756.932	0	0	0	0	58.7245	0	hypothetical protein, conserved
PVX_001775	14.9811	0	0	0	0	0	22.8827	0	11.3495	10.5294	8.94545	0	serine/threonine protein phosphatase 8, putative (PPP8)
PVX_001780	130.599	60.7781	0	0	27.6124	0	66.5546	0	32.9865	45.8891	43.3254	0	hypothetical protein, conserved
PVX_001782	0	0	0	0	0	0	0	0	0	0	49.6663	0	hypothetical protein
PVX_001783	0	0	0	0	0	0	0	531.006	0	0	38.0085	0	hypothetical protein
PVX_001785	0	0	0	0	0	0	0	0	0	0	0	0	hypothetical protein, conserved
PVX_001790	0	40.6794	0	62.0503	12.3174	0	0	26.9765	22.0814	20.4848	19.3375	0	hypothetical protein, conserved
PVX_001795	58.4635	0	0	0	0	0	0	81.1983	0	0	34.9115	0	hypothetical protein, conserved
PVX_001800	0	0	0	0	0	0	0	0	36.3858	33.7435	76.4624	0	hypothetical protein, conserved
PVX_001805	44.9094	31.3503	0	0	14.2431	0	68.6617	62.365	34.0297	15.7798	80.4514	0	hypothetical protein, conserved
PVX_001810	26.6439	27.888	33.0922	42.535	12.6655	0	20.3474	18.4942	0	23.4084	15.9094	21.5475	hypothetical protein, conserved
PVX_001820	0	0	0	0	0	0	34.4948	0	0	0	13.4818	0	hypothetical protein
PVX_001825	0	0	0	0	0	0	0	0	0	0	0	0	hypothetical protein, conserved
PVX_001830	0	0	0	0	0	0	0	0	0	0	0	0	hypothetical protein, conserved
PVX_001835	829.108	0	517.705	0	0	1065.65	637.104	576.604	943.427	509.402	165.066	0	60S ribosomal protein L30, putative

Gene ID / Patient	Patient 1	Patient 2	Patient 3	Patient 4	Patient 5	Patient 6	Patient 7	Patient 8	Patient 9	Patient 10	Patient 11	Patient 12	Gene Description
PVX_001840	0	17.3891	0	0	0	0	0	0	0	8.75575	24.7969	0	hypothetical protein, conserved
PVX_001845	62.0028	0	154.094	0	9.82883	0	47.3769	0	23.4888	21.7875	43.1942	50.168	hypothetical protein, conserved
PVX_001850	0	0	0	0	0	0	0	142.796	0	18.0636	10.2332	83.2431	hypothetical protein
PVX_001855	0	0	0	0	0	0	50.9263	0	0	0	13.2648	0	tRNA methyltransferase, putative
PVX_001860	0	0	0	0	0	0	0	0	0	0	103.606	853.505	autophagy-related protein 8, putative (ATG8)
PVX_001865	57.1566	0	0	0	18.1328	0	0	79.3822	43.3136	200.819	68.262	92.5619	RNA-binding protein, putative
PVX_001868	0	0	0	0	0	0	0	0	0	0	0	0	hypothetical protein, conserved
PVX_001872	0	0	0	0	0	0	0	0	0	12.7019	0	0	hypothetical protein, conserved
PVX_001875	0	0	0	0	0	0	0	0	0	0	0	0	cytoplasmic dynein intermediate chain, putative
PVX_001880	182.919	127.718	151.662	194.939	58.036	0	0	84.6856	138.62	128.534	60.6835	0	S-adenosyl-L-methionine-dependent methyltransferase, putative
PVX_001885	81.6727	0	0	0	0	0	0	0	30.9512	57.3842	97.5446	0	hypothetical protein, conserved
PVX_001890	0	0	0	0	6.31506	0	0	55.3226	30.1892	21.0047	31.7252	0	hypothetical protein, conserved
PVX_001895	0	35.703	0	0	0	0	0	35.514	19.3794	8.98846	20.3648	0	histone acetyltransferase, putative
PVX_001900	0	0	0	0	0	0	0	0	0	0	0	0	dihydrolipoamide acetyltransferase, putative
PVX_001905	0	0	1306.25	839.491	0	0	0	363.354	198.139	0	155.956	0	ADP-ribosylation factor, putative (ARF1)
PVX_001910	0	0	0	0	0	0	0	284.629	0	0	81.5544	0	hypothetical protein, conserved
PVX_001920	58.1031	13.5158	0	0	42.9708	0	0	53.7778	66.0291	34.0306	84.8087	31.3316	hypothetical protein, conserved
PVX_001925	0	0	0	182.601	18.1212	0	0	0	0	0	68.2185	0	hypothetical protein, conserved
PVX_001930	77.2322	53.9368	0	0	24.5138	0	0	0	29.2675	54.2655	76.8673	125.144	hypothetical protein, conserved
PVX_001935	0	37.7813	134.579	172.98	0	0	82.7607	0	41.008	57.0409	21.5431	0	endomembrane protein 70, putative
PVX_001940	0	0	266.734	0	0	274.445	246.047	148.96	0	94.2138	96.0727	0	ATP-dependent RNA helicase, putative
PVX_001945	0	106.257	379.06	0	145.006	0	466.387	1056.54	57.6322	266.905	302.601	246.82	deoxyribose-phosphate aldolase, putative
PVX_001950	10.242	0	0	16.3469	3.24512	0	0	21.3261	7.75868	3.59946	9.17338	0	hypothetical protein, conserved
PVX_001955	0	0	44.6656	0	5.69823	0	0	24.9601	0	12.6361	32.2062	29.0834	schizont egress antigen-1, putative (SEA1)
PVX_001960	33.4758	0	0	106.894	0	0	0	0	12.6805	23.5282	33.3149	0	hypothetical protein, conserved,10b antigen, putative
PVX_001965	20.3452	0	50.5482	0	12.8973	0	0	28.2462	0	21.4487	8.099	0	RNA-binding protein, putative
PVX_001970	145.339	0	362.371	0	184.833	0	0	0	220.411	51.042	231.464	235.953	hypothetical protein, conserved
PVX_001975	65.6409	68.7233	0	0	20.812	0	0	45.5719	37.3011	69.1971	84.925	0	hypothetical protein, conserved
PVX_001980	0	167.257	0	0	0	0	0	0	0	0	0	0	zinc transporter, putative
PVX_002480	0	0	0	0	0	0	0	0	0	0	0	0	variable surface protein Vir12/24-related,PIR protein
PVX_002485	0	180.637	0	206.794	41.0431	331.08	0	0	122.533	136.334	64.3675	0	variable surface protein Vir12-related,PIR protein
PVX_002490	0	0	0	0	18.9573	0	0	0	0	0	0	0	variable surface protein Vir12-related,PIR protein
PVX_002495	0	0	0	0	0	0	0	0	0	0	0	0	variable surface protein Vir7-like,PIR protein
PVX_002500	0	0	0	0	0	0	0	0	0	0	0	0	tryptophan-rich antigen (Pv-fam-a)
PVX_002505	38.6788	0	0	0	0	0	0	0	0	13.591	0	0	Plasmodium exported protein, unknown function
PVX_002507	0	40.5806	0	0	0	0	0	0	0	0	0	0	Pv-fam-b protein
PVX_002510	0	0	0	0	0	0	0	0	0	0	0	0	Nucleosomal binding protein 1, putative
PVX_002512	0	39.7538	0	0	0	291.411	0	0	0	0	22.6671	0	Plasmodium exported protein, unknown function
PVX_002515	0	43.9204	0	0	0	0	0	0	23.8345	0	0	0	Pv-fam-b protein
PVX_002520	0	0	0	0	0	0	0	0	0	0	0	0	Pv-fam-b protein
PVX_002525	0	0	0	0	30.3369	0	0	0	0	0	0	0	Pv-fam-b protein
PVX_002530	0	76.8361	0	0	0	0	0	0	0	38.6296	0	0	Pv-fam-b protein
PVX_002535	91.1564	63.6724	0	0	0	467.017	0	0	34.5475	96.0667	0	0	Pv-fam-b protein
PVX_002550	21.9488	45.946	0	0	6.95533	56.0897	50.2817	15.2349	78.9812	38.5674	15.2902	17.7493	hypothetical protein, conserved
PVX_002555	80.1369	335.804	398.875	0	25.4376	0	245.317	333.97	91.1066	112.611	143.565	0	Plasmodium exported protein, unknown function
PVX_002560	0	0	0	0	0	0	0	0	0	0	0	0	hypothetical protein
PVX_002565	0	0	0	0	0	0	0	0	0	0	25.9035	0	RAD protein (Pv-fam-e)
PVX_002570	112.889	0	0	0	0	0	0	0	0	0	0	0	Plasmodium exported protein, unknown function
PVX_002575	58.5162	0	145.422	93.4587	9.27573	149.619	0	162.496	88.6708	61.6875	87.354	47.3447	replication factor C subunit 1, putative
PVX_002580	96.4621	0	0	308.71	61.2639	0	295.446	0	0	33.8852	57.6052	0	pseudouridine synthetase, putative
PVX_002585	0	0	711.971	457.565	45.3945	0	0	0	54.1346	0	56.8502	0	ribosome associated membrane protein RAMP4, putative
PVX_002590	447.874	1885.69	1126.28	0	143.461	2319.39	4161.9	3122.12	2041.89	1413.47	2051.49	1466.72	40S ribosomal protein S30, putative
PVX_002595	0	0	0	0	0	0	0	0	150.551	278.081	78.9401	0	hypothetical protein, conserved
PVX_002600	0	0	0	0	0	0	0	0	0	18.6307	10.5546	85.861	hypothetical protein, conserved
PVX_002605	0	0	0	209.064	41.4936	0	0	90.8147	49.5504	45.9426	65.073	0	nuclear protein SkiP, putative
PVX_002610	0	10.6848	0	48.8909	4.85265	0	0	42.5141	5.8	5.3809	30.4763	0	hypothetical protein, conserved
PVX_002615	127.892	0	0	0	0	0	196.052	0	193.929	179.675	178.215	207.527	small nuclear ribonucleoprotein Sm D2, putative
PVX_002620	63.1678	0	0	0	0	0	0	0	0	22.1933	50.2945	0	DEAD/DEAH box helicase, putative
PVX_002625	0	0	0	324.492	0	0	0	0	38.4246	0	100.904	0	rRNA methylase, putative
PVX_002630	23.0252	0	0	0	0	0	0	31.9679	8.72227	24.2738	4.58295	37.2522	hypothetical protein
PVX_002635	0	0	0	0	0	0	0	0	0	0	0	0	hypothetical protein
PVX_002640	113.318	0	0	0	0	0	0	0	42.9529	119.408	45.115	0	replication factor C subunit 2, putative

Gene ID / Patient	Patient 1	Patient 2	Patient 3	Patient 4	Patient 5	Patient 6	Patient 7	Patient 8	Patient 9	Patient 10	Patient 11	Patient 12	Gene Description
PVX_002645	123.624	0	0	131.674	0	0	62.995	114.445	46.8362	115.836	65.6179	0	hypothetical protein, conserved
PVX_002650	574.122	804.072	0	0	183.171	0	0	799.174	762.581	604.454	571.567	2339.52	40S ribosomal protein S26, putative (RPS26)
PVX_002655	0	0	0	0	0	0	0	0	52.5403	48.6724	27.5886	0	hypothetical protein, conserved
PVX_002660	41.4537	14.4645	0	0	0	0	0	28.7762	23.5545	0	12.3764	0	hypothetical protein, conserved
PVX_002665	64.9559	45.3561	0	0	0	0	99.3742	90.2211	24.6133	0	64.6479	0	calcium-dependent protein kinase 1, putative (CDPK1)
PVX_002670	162.197	0	0	520.046	51.5895	0	0	0	0	113.915	96.8692	263.447	hypothetical protein, conserved
PVX_002675	0	0	0	0	0	0	621.281	0	152.582	140.908	0	0	AP-2 complex subunit sigma, putative
PVX_002680	0	0	58.63	150.72	7.47959	0	0	32.7604	8.93848	16.5836	28.1793	0	hypothetical protein, conserved
PVX_002685	179.976	125.662	149.218	0	95.1683	0	275.296	83.3223	113.658	84.3113	191.063	0	ATP synthase alpha chain, putative
PVX_002690	0	0	0	0	10.6845	0	0	0	0	0	13.4147	0	hypothetical protein, conserved
PVX_002695	0	0	0	0	0	0	0	0	0	23.86	0	0	hypothetical protein, conserved
PVX_002700	0	0	0	0	0	0	0	0	0	13.1809	7.46646	0	hypothetical protein, conserved
PVX_002705	0	0	0	0	0	0	0	0	15.3928	7.13984	0	0	hypothetical protein, conserved
PVX_002710	0	0	0	160.446	15.923	0	0	69.7149	19.0198	88.1907	79.9367	81.2792	hypothetical protein, conserved
PVX_002715	0	29.0907	0	66.5617	0	0	0	0	0	7.3244	16.5941	0	hypothetical protein, conserved
PVX_002720	138.053	0	114.415	147.063	29.1901	0	70.3589	0	87.1747	80.8461	64.1178	74.4998	vacuolar protein sorting-associated protein 45, putative
PVX_002725	0	0	0	0	8.28381	0	0	72.5631	39.5965	27.5479	10.4024	0	hypothetical protein, conserved
PVX_002730	0	0	0	0	0	0	0	0	81.2575	0	42.6499	0	hypothetical protein, conserved
PVX_002735	0	0	0	0	19.1789	0	0	41.9975	34.3756	21.2574	42.143	0	hypothetical protein
PVX_002740	0	0	0	0	0	0	0	0	14.5691	0	26.7932	0	DEAD/DEAH helicase, putative
PVX_002745	0	0	0	0	0	0	0	0	0	0	0	0	palmitoyltransferase, putative (DHHC11)
PVX_002750	0	0	0	0	0	0	0	0	0	0	23.3999	0	origin recognition complex subunit 5, putative (ORC5)
PVX_002755	40.1969	56.1034	0	0	25.4816	102.75	30.7041	27.9036	15.2268	35.3144	12.0012	32.5144	DNA-directed RNA polymerase II 135 kDa polypeptide, putative
PVX_002760	0	140.214	0	321.208	31.8716	0	0	278.878	76.0731	0	159.816	162.719	hypothetical protein, conserved
PVX_002765	51.0587	35.6459	0	0	16.1958	0	0	141.817	58.0364	35.8799	111.794	0	hypothetical protein, conserved
PVX_002770	0	56.3362	0	0	0	0	61.6867	56.0353	0	28.3585	16.0642	0	hypothetical protein, conserved
PVX_002775	0	0	0	0	0	0	275.514	0	0	0	35.7218	0	hypothetical protein, conserved
PVX_002780	0	0	0	0	0	0	0	0	47.4639	0	0	0	RING zinc finger protein, putative
PVX_002785	89.6837	125.213	0	0	0	0	0	62.2712	101.936	63.0245	35.7024	0	ATP-dependent acyl-CoA synthetase, putative
PVX_002790	0	0	0	0	0	0	0	0	0	0	0	0	rhoptry neck protein 6, putative (RON6)
PVX_002795	0	27.9951	49.834	0	0	0	0	0	7.59805	14.0973	7.98464	0	hypothetical protein, conserved
PVX_002800	0	0	0	0	0	0	0	0	0	0	0	0	hypothetical protein, conserved
PVX_002805	0	0	0	0	6.10695	0	0	0	14.5973	0	11.5051	0	calcium-dependent protein kinase 4, putative
PVX_002810	0	0	0	0	0	0	0	176.234	0	89.0744	100.971	0	hypothetical protein, conserved
PVX_002815	41.5137	57.9573	0	132.652	13.1651	0	0	0	62.9118	72.9347	57.8418	0	hypothetical protein, conserved
PVX_002820	21.7547	0	0	0	0	0	0	0	0	15.2897	4.33006	0	hypothetical protein
PVX_002825	0	0	0	0	65.3922	0	0	0	0	72.1044	40.8877	0	50S ribosomal protein L13, putative
PVX_002830	65.264	30.364	0	69.476	13.7913	0	0	60.4071	82.409	53.5139	90.9312	35.1954	protein transport protein SEC31, putative (SEC31)
PVX_002835	284.293	231.543	0	151.429	75.1415	242.433	72.4481	329.003	161.568	249.728	198.057	76.7116	T-complex protein 1, theta subunit, putative
PVX_002840	28.305	19.7549	0	0	17.9467	0	0	0	42.8908	19.8927	56.3388	0	hypothetical protein, conserved
PVX_002845	0	31.3976	0	143.733	0	0	0	62.4591	34.081	15.8036	26.8576	72.8128	hypothetical protein, conserved
PVX_002850	0	0	0	0	0	0	0	0	0	359.858	0	0	hypothetical protein, conserved
PVX_002855	0	0	0	0	0	0	0	0	5.32275	4.93821	5.59375	0	hypothetical protein, conserved
PVX_002860	0	50.7785	180.927	0	0	0	0	101.005	27.5545	0	57.896	0	hypothetical protein, conserved
PVX_002865	0	0	0	0	34.1768	0	0	149.508	40.7824	0	21.4183	0	serine/threonine protein kinase, putative
PVX_002867	0	0	4621.49	0	0	0	0	5015.17	0	0	709.378	0	conserved Plasmodium protein, unknown function
PVX_002870	0	0	0	0	0	0	0	0	187.969	0	394.026	0	hypothetical protein, conserved
PVX_002875	0	0	0	0	0	0	0	0	43.282	80.2147	90.9208	0	heat shock protein, putative
PVX_002880	169.895	0	0	0	0	0	0	0	64.4235	119.317	33.8225	276.01	hypothetical protein, conserved
PVX_002885	85.6595	0	0	0	27.1943	0	0	0	0	0	102.307	0	Leu/Phe-tRNA protein transferase, putative
PVX_002890	37.3706	0	0	119.399	11.85	191.151	0	0	28.3158	39.3943	52.0688	0	hypothetical protein, conserved
PVX_002895	0	69.4054	0	0	31.5524	0	0	0	75.3125	34.9004	19.7774	161.088	hypothetical protein, conserved
PVX_002900	287.872	0	0	0	0	0	0	200.077	0	0	28.6537	0	secreted protein with altered thrombospondin repeat domain, putative (SPATR)
PVX_002905	8.07065	5.63129	20.0449	25.7646	7.67196	0	12.3247	11.2035	15.2848	19.8542	25.7017	0	hypothetical protein, conserved
PVX_002910	8.63607	18.0776	0	27.5701	10.9461	0	13.1884	23.977	6.54228	18.2101	30.9401	41.8996	hypothetical protein, conserved
PVX_002915	165.154	115.347	0	0	26.2137	0	252.806	458.864	187.765	116.039	131.5	267.649	eukaryotic peptide chain release factor subunit 1, putative
PVX_002920	0	0	0	0	22.1401	0	106.75	193.815	52.874	122.554	138.872	0	50S ribosomal protein L7/L12, putative
PVX_002925	0	0	0	0	6.1627	0	29.7029	26.9941	0	6.83273	19.3501	0	hypothetical protein, conserved
PVX_002930	0	0	309.396	0	39.4562	0	0	172.562	94.1369	43.6109	49.4347	0	GDP-fucose transporter, putative
PVX_002935	173.815	121.402	0	278.057	82.7739	0	0	241.473	32.9361	61.0605	69.1982	0	hypothetical protein, conserved
PVX_002940	230.689	281.866	0	368.738	91.4833	0	176.422	80.0988	305.932	263.42	172.195	93.3983	asparagine--tRNA ligase, putative
PVX_002945	60.9264	0	0	0	9.65807	0	0	0	0	10.7046	72.7617	0	tyrosine kinase-like protein, putative (TKL1)

Gene ID / Patient	Patient 1	Patient 2	Patient 3	Patient 4	Patient 5	Patient 6	Patient 7	Patient 8	Patient 9	Patient 10	Patient 11	Patient 12	Gene Description
PVX_002950	0	0	0	0	204.674	0	0	0	0	0	42.6499	0	hypothetical protein, conserved
PVX_002955	0	0	0	0	7.3707	0	0	0	0	0	9.25641	0	hypothetical protein, conserved
PVX_002960	0	0	0	0	0	0	0	0	0	0	0	0	beta-ketoacyl-acyl carrier protein synthase III precursor, putative
PVX_002965	78.9353	0	196.44	0	25.0554	0	0	0	29.9133	0	15.7125	0	hypothetical protein, conserved
PVX_002970	0	106.257	0	0	48.3353	0	233.193	0	115.264	106.762	90.7803	0	ras-related protein Rab-5A, putative (RAB5a)
PVX_002975	28.8432	0	0	0	18.2882	0	88.1513	40.0478	32.7799	30.4064	11.482	46.6727	hypothetical protein, conserved
PVX_003475	0	0	0	0	0	0	0	0	0	0	7.91545	16.0837	hypothetical protein, conserved
PVX_003485	0	0	0	0	0	0	0	0	0	0	41.2017	167.814	variable surface protein Vir4, putative,PIR protein
PVX_003487	214.223	0	0	0	0	0	0	0	0	75.2079	0	0	Plasmodium exported protein, unknown function
PVX_003490	0	26.6872	0	0	0	0	0	0	0	0	0	0	VIR protein,PIR protein
PVX_003495	0	0	0	0	0	0	0	0	0	0	0	0	variable surface protein Vir22/24-related,PIR protein
PVX_003500	0	0	0	0	0	0	0	0	0	0	0	0	VIR protein,PIR protein
PVX_003505	0	26.5849	0	0	0	0	0	0	0	13.3829	0	0	variable surface protein Vir24-related,PIR protein
PVX_003510	0	0	0	0	0	0	0	0	0	0	0	0	variable surface protein Vir24-related,PIR protein
PVX_003515	0	54.9333	0	0	0	0	0	0	0	0	0	0	Phist protein (Pf-fam-b)
PVX_003520	0	0	0	0	0	0	0	0	0	0	0	0	histidine-rich knob protein homolog KPRPC, putative
PVX_003525	0	0	0	0	0	0	0	23.0049	0	0	3.29818	0	Plasmodium exported protein, unknown function
PVX_003530	0	0	0	0	54.1514	0	0	0	0	0	67.7738	0	hypothetical protein
PVX_003535	0	0	0	0	0	0	0	0	0	0	14.1971	0	Phist protein (Pf-fam-b)
PVX_003540	0	52.8412	0	0	0	0	0	0	28.6734	0	15.0615	0	Plasmodium exported protein (PHISTc), unknown function
PVX_003545	187.126	391.975	155.157	0	0	638.578	190.837	173.269	212.715	306.809	148.99	202.056	Plasmodium exported protein, unknown function
PVX_003550	0	0	0	0	0	0	0	0	0	0	0	0	Plasmodium exported protein, unknown function
PVX_003555	0	197.3	70.2513	90.2974	0	289.115	86.3955	39.2507	53.5458	49.669	16.8803	45.7432	Plasmodium exported protein, unknown function
PVX_003560	0	0	0	310.725	30.8318	0	148.688	0	73.5955	34.1056	38.6535	0	hypothetical protein
PVX_003565	2455.2	3661.98	816.626	0	1249.52	3361.62	502.4	0	2296.38	2414.08	1075.31	0	early transcribed membrane protein (ETRAMP)
PVX_003570	0	0	60.9622	78.3576	0	0	0	34.063	9.29385	17.2427	9.76648	0	hypothetical protein, conserved
PVX_003575	0	0	0	0	0	0	0	0	0	0	0	0	octaprenyl pyrophosphate synthase, putative (OPP)
PVX_003578	0	0	0	1684.83	0	0	0	0	0	0	0	0	conserved Plasmodium protein, unknown function
PVX_003580	135.441	0	0	0	0	0	0	0	0	0	0	0	palmitoyltransferase, putative (DHHC12)
PVX_003585	16.6352	0	41.3264	0	0	0	0	0	0	23.3838	23.1774	0	hypothetical protein, conserved
PVX_003590	0	0	0	0	4.59321	0	0	0	0	0	0	0	serine/threonine-specific protein kinase, putative
PVX_003595	111.814	39.0331	0	0	53.2075	0	0	77.6456	42.3662	78.5719	55.6412	0	hypothetical protein, conserved
PVX_003600	0	0	0	0	0	0	282.346	255.667	139.449	0	183.013	0	ERCC1 nucleotide excision repair protein, putative
PVX_003605	0	0	0	563.106	0	0	0	0	0	0	104.852	0	hypothetical protein, conserved
PVX_003607	0	0	0	0	0	0	0	0	0	0	0	0	tRNA Glutamine
PVX_003610	0	0	0	0	42.425	0	0	0	25.3306	0	66.5313	0	hypothetical protein, conserved
PVX_003615	62.3753	43.5528	0	199.43	19.791	0	0	0	23.635	65.7448	62.0793	0	hypothetical protein, conserved
PVX_003620	61.3491	0	152.599	0	19.4649	0	93.8454	170.417	116.23	86.218	24.4232	0	hypothetical protein, conserved
PVX_003625	0	0	0	0	0	0	0	0	0	0	0	0	5'-3' exonuclease, N-terminal resolvase-like domain, putative
PVX_003630	0	0	0	0	13.1896	0	0	0	0	0	24.8354	0	hypothetical protein, conserved
PVX_003635	12.2296	0	0	0	0	0	0	0	0	0	0	0	hypothetical protein, conserved
PVX_003640	0	0	0	0	45.1056	0	217.595	0	0	49.8291	141.224	0	hypothetical protein, conserved
PVX_003645	0	0	0	0	0	0	0	0	0	0	0	0	hypothetical protein, conserved
PVX_003650	0	0	0	0	0	0	0	0	0	0	0	0	dynein light polypeptide 4, axonemal, putative
PVX_003655	0	0	0	0	0	0	101.818	0	25.2176	0	52.9876	0	aspartate aminotransferase, mitochondrial precursor, putative
PVX_003660	0	0	0	0	0	0	0	0	19.4406	0	10.2145	0	5'-3' exoribonuclease, putative
PVX_003665	0	47.8593	0	0	0	0	104.866	0	51.9422	96.317	81.8555	111.028	hexose transporter
PVX_003670	0	0	0	0	0	0	0	0	29.42	0	0	0	3'-5' exonuclease domain containing protein
PVX_003675	0	0	0	0	0	0	0	0	0	0	18.2011	148.218	ubiquinone biosynthesis methyltransferase, putative
PVX_003680	0	0	0	0	29.9772	0	0	0	0	16.6046	9.40638	0	hypothetical protein
PVX_003685	0	0	0	0	19.385	0	0	0	0	0	36.4847	0	hypothetical protein
PVX_003690	0	0	0	0	0	0	0	0	0	0	0	0	hypothetical protein, conserved
PVX_003695	0	0	0	0	13.6009	0	0	0	0	0	17.0727	0	hypothetical protein, conserved
PVX_003700	53.3093	148.873	0	0	50.7321	272.808	81.5262	74.036	60.5956	37.4611	74.278	0	hypothetical protein, conserved
PVX_003705	913.96	0	0	0	0	0	0	0	173.628	0	637.068	0	DNA-directed RNA polymerase II 16 kDa subunit, putative
PVX_003710	341.894	0	857.19	0	0	0	0	0	0	0	0	0	hypothetical protein
PVX_003715	0	0	124.617	0	15.8962	0	0	0	0	17.6085	39.9012	81.1424	hypothetical protein, conserved
PVX_003720	120.651	0	300.607	386.384	38.3358	0	0	167.67	0	42.3769	24.0174	0	hypothetical protein, conserved
PVX_003725	0	0	319.172	0	0	0	0	178.001	0	0	0	0	PH domain containing protein
PVX_003730	24.799	34.6144	308.107	158.41	78.6119	0	75.7815	103.294	93.9431	95.8596	187.569	120.372	26S proteasome regulatory subunit RPN1, putative (RPN1)
PVX_003735	0	14.804	0	0	6.72393	0	0	29.4517	0	7.45459	21.1114	0	DNA repair endonuclease, putative,RAD2 endonuclease, putative
PVX_003740	0	115.709	0	0	0	0	0	0	0	0	32.9467	0	cysteine desulfuration protein SufE, putative (SufE)

Gene ID / Patient	Patient 1	Patient 2	Patient 3	Patient 4	Patient 5	Patient 6	Patient 7	Patient 8	Patient 9	Patient 10	Patient 11	Patient 12	Gene Description
PVX_003745	0	0	0	0	0	0	0	0	0	0	0	0	pantothenate transporter, putative (PAT)
PVX_003750	25.6327	8.94315	0	0	12.1846	0	19.5748	17.7923	67.9654	18.0161	12.7547	0	pentafunctional AROM polypeptide, putative (AROM)
PVX_003755	29.2964	20.4472	0	0	9.28789	149.815	0	40.6773	0	0	52.481	0	hypothetical protein, conserved
PVX_003760	0	0	1893.53	0	0	0	0	0	0	0	0	0	transcription factor, putative
PVX_003765	0	52.4418	0	0	0	0	0	208.625	28.4567	79.1452	59.7909	0	adenylosuccinate lyase (ASL)
PVX_003770	0	0	0	0	0	0	0	0	35.3031	0	0	0	merozoite surface protein 5
PVX_003775	0	0	0	0	0	0	0	0	0	0	0	0	merozoite surface protein 4, putative
PVX_003780	18.7352	13.0743	0	59.8278	5.93811	0	0	26.0106	14.1939	6.58386	22.3743	0	hypothetical protein, conserved
PVX_003785	0	0	0	0	0	0	0	351.033	0	0	0	0	iron-sulfur cluster assembly accessory protein, putative
PVX_003790	0	0	0	0	0	0	0	0	0	0	0	0	serine-repeat antigen (SERA), putative,serine-repeat antigen protein precursor, putative
PVX_003795	0	0	0	0	0	0	0	0	0	0	0	0	serine-repeat antigen (SERA),serine-repeat antigen protein precursor, putative
PVX_003800	31.4923	0	0	0	9.98459	0	0	43.7272	71.5825	11.0662	18.8049	0	serine-repeat antigen (SERA),serine-repeat antigen protein precursor, putative
PVX_003805	0	0	0	0	0	0	0	0	10.5689	0	0	0	serine-repeat antigen (SERA), putative
PVX_003810	65.7615	0	0	0	0	0	0	0	0	0	0	0	serine-repeat antigen 5 (SERA), putative
PVX_003815	0	0	0	0	0	0	0	0	0	0	0	0	serine-repeat antigen (SERA), truncated, putative
PVX_003820	29.1818	0	0	0	0	0	0	0	0	0	0	0	serine-repeat antigen 4 (SERA), putative
PVX_003825	31.7267	0	0	0	0	0	0	0	0	0	0	0	serine-repeat antigen 4 (SERA),cysteine protease, putative
PVX_003830	0	0	0	0	0	0	0	0	0	0	0	0	serine-repeat antigen 5 (SERA)
PVX_003835	29.1533	0	0	0	0	0	0	0	0	0	11.6055	0	serine-repeat antigen 1 (SERA)
PVX_003840	0	0	0	0	0	0	0	0	0	10.6179	12.0287	0	serine-repeat antigen 3 (SERA)
PVX_003845	26.254	18.323	0	0	0	0	0	0	0	36.903	26.1282	0	serine-repeat antigen 4 (SERA)
PVX_003850	0	0	0	0	0	0	0	0	0	0	0	0	serine-repeat antigen 2 (SERA)
PVX_003855	0	14.1114	0	0	0	103.378	0	28.0739	0	7.10597	16.0992	32.7128	hypothetical protein, conserved
PVX_003860	0	0	0	407.904	0	653.136	195.211	0	96.5501	134.182	152.103	0	KRR1 small subunit processome component, putative (KRR1)
PVX_003865	0	14.4144	0	0	6.54693	0	0	57.3532	31.2972	43.5508	28.7783	33.4154	hypothetical protein, conserved
PVX_003870	16.1577	0	40.1395	0	5.12085	0	0	0	12.2409	22.7126	19.296	26.1363	hypothetical protein, conserved
PVX_003875	0	0	0	0	0	0	0	0	0	0	0	0	hypothetical protein
PVX_003880	0	0	0	0	0	0	616.966	279.229	152.293	70.4885	159.881	0	acyl carrier protein, putative (ACP)
PVX_003885	0	87.7174	0	0	19.9301	0	0	0	47.602	132.412	87.5211	0	ribosome-recycling factor, putative (RRF1)
PVX_003890	0	70.0281	0	0	0	0	0	0	75.9878	105.639	59.8639	0	hypothetical protein, conserved
PVX_003895	199.87	0	0	0	0	0	0	0	0	0	79.5832	0	hypothetical protein, conserved
PVX_003900	13.8862	9.68985	0	0	0	0	0	38.5555	0	4.87998	0	0	6-cysteine protein
PVX_003905	11.1258	7.76335	0	0	0	0	0	0	0	0	2.21446	0	6-cysteine protein
PVX_003910	0	0	111.656	0	0	0	0	0	0	31.5597	35.7562	0	phospholipase A2, putative
PVX_003915	106.698	298.171	0	0	33.8908	0	163.451	0	202.209	149.915	212.395	0	hypothetical protein, conserved
PVX_003920	0	0	337.598	0	43.0509	0	0	0	0	0	26.9622	0	2C-methyl-D-erythritol 2,4-cyclodiphosphate synthase, putative
PVX_003925	129.818	0	0	0	0	0	99.3019	0	98.3817	205.243	103.362	0	hypothetical protein, conserved
PVX_003930	0	0	0	0	0	0	0	0	0	0	0	0	hypothetical protein
PVX_003935	0	0	0	0	0	0	0	0	0	0	11.4012	0	amine transporter, putative
PVX_003940	0	211.566	0	0	19.2273	0	0	84.1692	68.8876	21.2919	24.1254	0	RING zinc finger protein, putative
PVX_003945	376.304	300.252	534.752	171.835	136.425	550.211	328.852	522.613	427.737	642.193	363.813	261.147	ATP-dependent RNA helicase UAP56, putative (UAP56)
PVX_003950	0	1082.12	0	0	499.598	0	0	0	582.496	1063.13	911.16	0	transport protein SEC61 gamma subunit, putative
PVX_003955	1932.45	2729.32	7391.9	3167.05	313.281	0	0	4060.88	4419.98	2365.92	3080.8	0	60S ribosomal protein L37a, putative
PVX_003960	0	19.1303	34.0505	43.7668	8.68815	0	0	38.0594	10.3846	14.4516	35.4683	0	hypothetical protein, conserved
PVX_003965	0	0	189.732	0	24.2	0	0	211.827	0	26.7861	91.0618	0	transporter, putative
PVX_003970	0	0	0	694.556	0	0	0	0	0	0	129.187	0	50S ribosomal protein L33, putative
PVX_003975	26.9904	0	0	0	8.55631	0	0	0	51.123	18.9689	26.861	0	hypothetical protein, conserved
PVX_003980	0	0	0	0	0	0	0	0	0	0	15.6572	0	hypothetical protein, conserved
PVX_003985	0	0	0	0	11.7587	0	0	51.4648	14.0398	52.0647	7.37484	120.056	syntaxin, putative
PVX_003990	0	0	0	0	0	0	0	0	67.2444	0	0	0	hypothetical protein, conserved
PVX_003995	0	87.1295	310.694	0	0	0	0	0	189.061	131.379	124.103	0	hypothetical protein, conserved
PVX_004495	0	0	0	0	0	0	0	0	0	0	0	0	VIR protein,PIR protein
PVX_004503	0	0	0	0	0	0	0	0	0	0	7.45509	0	VIR protein,PIR protein
PVX_004505	0	0	0	0	0	0	0	0	0	0	0	0	hypothetical protein
PVX_004510	0	0	0	0	31.6581	0	0	0	0	0	0	0	variable surface protein Vir4/14-related,PIR protein
PVX_004515	188.503	395.503	0	0	0	0	0	0	71.489	66.1869	0	0	RAD protein (Pv-fam-e)
PVX_004520	0	0	0	322.295	0	0	0	0	0	106.114	60.1333	0	variable surface protein Vir4, putative,PIR protein
PVX_004525	0	0	0	0	0	0	0	0	0	36.6064	0	0	variable surface protein Vir16/32-related,PIR protein
PVX_004530	0	0	0	0	0	0	0	0	0	28.3814	0	0	VIR protein,PIR protein
PVX_004535	149.81	104.62	0	0	0	0	0	0	56.7704	131.578	59.6407	0	variable surface protein Vir, putative,PIR protein
PVX_004536	0	0	0	0	0	0	0	0	0	0	0	0	Plasmodium exported protein, unknown function
PVX_004537	0	0	0	0	0	0	0	0	22.7927	0	0	0	VIR protein,PIR protein

Gene ID / Patient	Patient 1	Patient 2	Patient 3	Patient 4	Patient 5	Patient 6	Patient 7	Patient 8	Patient 9	Patient 10	Patient 11	Patient 12	Gene Description
PVX_004539	0	120.002	0	274.846	54.5456	0	0	0	65.1131	60.3574	119.702	0	variable surface protein Vir12-related,PIR protein
PVX_005040	0	0	0	0	0	0	0	0	0	30.2658	17.1496	0	hypothetical protein
PVX_005045	0	0	0	0	0	0	0	0	0	0	11.66	0	variable surface protein Vir12-related,PIR protein
PVX_005050	0	0	0	0	0	0	0	0	0	0	0	0	variable surface protein Vir22/23-related,PIR protein
PVX_005055	0	19.8264	0	0	0	0	0	0	0	19.9545	0	0	VIR protein,PIR protein
PVX_005057	78.8657	0	0	0	0	403.9	0	0	0	0	15.6986	0	VIR protein,PIR protein
PVX_005058	0	0	0	0	0	0	0	0	0	32.7218	0	0	VIR protein,PIR protein
PVX_005060	0	0	193.196	0	0	0	0	0	0	0	0	0	variable surface protein Vir12-like,PIR protein
PVX_005065	0	0	0	0	0	0	0	0	0	0	0	0	VIR protein,PIR protein
PVX_005565	0	0	0	0	0	0	0	0	0	0	0	0	hypothetical protein
PVX_005570	0	0	0	0	0	0	0	0	0	0	0	0	hypothetical protein
PVX_005575	0	0	0	0	0	0	0	0	0	0	0	0	hypothetical protein
PVX_005580	0	0	0	0	0	0	0	0	0	0	0	0	variable surface protein Vir4, putative
PVX_006080	0	0	0	0	0	0	0	0	0	0	11.6372	0	variable surface protein Vir18-related
PVX_006580	0	0	0	0	0	0	0	0	0	0	0	0	variable surface protein Vir14, putative
PVX_007080	0	0	0	0	0	0	0	0	0	0	0	0	variable surface protein Vir 28, putative
PVX_007085	0	0	0	0	0	0	0	0	0	0	0	0	variable surface protein Vir24-related
PVX_007585	0	0	0	0	0	0	0	0	0	0	0	0	variable surface protein Vir28, putative
PVX_008085	266.994	279.653	166.055	0	42.3616	0	102.122	0	0	117.254	66.4321	0	variable surface protein Vir 14-related
PVX_009090	0	0	0	0	0	0	0	0	0	0	0	0	variable surface protein Vir14-like
PVX_009590	0	0	0	0	0	0	0	0	0	0	0	0	variable surface protein Vir21, pseudogene, putative
PVX_009595	0	0	0	0	0	0	0	0	0	0	0	0	variable surface protein Vir22, putative
PVX_009600	0	0	0	0	0	0	0	0	0	0	0	0	variable surface protein Vir5-related
PVX_010100	0	0	0	0	0	0	0	0	0	0	0	0	variable surface protein Vir18, putative
PVX_010105	0	0	0	0	0	0	0	0	0	0	0	0	variable surface protein Vir12-related
PVX_010605	0	0	0	0	0	0	0	0	0	0	0	0	variable surface protein Vir15, putative
PVX_010610	0	0	0	0	0	0	0	0	0	0	0	0	variable surface protein Vir14, putative
PVX_011110	0	0	0	0	0	0	0	0	0	0	0	0	variable surface protein Vir18-like
PVX_011610	0	0	0	0	0	0	0	0	0	0	0	0	variable surface protein Vir17, putative
PVX_011615	0	0	0	0	0	0	0	0	0	0	0	0	variable surface protein vir23, putative
PVX_012115	0	0	0	0	21.5511	0	0	0	0	0	27.0367	0	variable surface protein Vir 28/6-related
PVX_013120	0	0	0	0	0	0	0	0	0	0	0	0	variable surface protein Vir18, putative
PVX_013620	0	44.2943	0	0	0	0	0	0	0	0	12.6271	0	variable surface protein Vir12-like
PVX_013625	0	0	0	0	0	0	0	0	0	0	18.9565	0	variable surface protein Vir18, putative
PVX_014125	0	0	0	0	0	0	0	0	0	0	0	0	variable surface protein Vir6-like, pseudogene
PVX_014625	0	0	0	0	0	0	0	0	0	0	0	0	variable surface protein Vir7, putative
PVX_014630	0	0	0	0	0	0	0	0	0	0	0	0	variable surface protein Vir6, putative
PVX_015130	0	0	0	0	0	0	0	0	0	0	0	0	variable surface protein Vir18-like
PVX_015135	0	0	0	0	0	0	0	0	0	0	0	0	variable surface protein Vir17, truncated, putative
PVX_015635	0	0	0	0	0	0	0	0	0	0	0	0	variable surface protein Vir12-like
PVX_015640	0	0	0	0	0	0	0	0	0	0	0	0	variable surface protein Vir18-related
PVX_016140	0	0	0	0	0	0	0	0	0	0	0	0	variable surface protein Vir4, putative
PVX_016640	0	0	0	0	0	0	0	0	0	0	0	0	variable surface protein Vir18-like
PVX_017140	0	0	0	0	0	0	0	0	0	0	0	0	variable surface protein Vir12-like
PVX_017145	0	0	0	0	0	0	0	0	0	0	0	0	variable surface protein Vir18, putative
PVX_017645	0	0	0	0	0	0	0	0	0	64.1616	0	0	variable surface protein Vir35, putative
PVX_017650	0	0	0	0	0	0	0	0	0	0	0	0	variable surface protein Vir18, putative
PVX_018150	0	0	0	0	0	0	0	0	0	0	0	0	variable surface protein Vir18, putative
PVX_018155	0	0	0	0	0	0	0	0	0	0	0	0	variable surface protein Vir17, putative
PVX_018655	0	0	0	0	0	0	0	0	0	0	0	0	variable surface protein Vir28-like
PVX_018660	0	0	0	0	0	0	0	0	0	0	0	0	variable surface protein Vir24-like
PVX_019160	0	0	0	0	0	0	0	0	0	0	0	0	variable surface protein Vir17, putative
PVX_019165	0	0	0	0	0	350.091	0	0	25.9115	0	0	0	variable surface protein Vir18, putative
PVX_019665	0	0	0	0	0	0	0	0	0	0	0	0	variable surface protein Vir11, putative
PVX_019670	0	0	0	0	0	0	0	0	0	0	0	0	variable surface protein Vir12-related
PVX_020170	0	0	0	0	0	0	0	0	0	0	0	0	hypothetical protein
PVX_020175	0	0	0	0	0	0	0	0	0	0	0	0	Pv-fam-c protein
PVX_020675	0	0	0	0	0	0	0	0	0	0	0	0	variable surface protein Vir18, putative
PVX_020680	0	40.5016	0	0	0	0	0	0	0	0	0	0	variable surface protein Vir12-like
PVX_021180	0	0	0	0	0	0	0	0	0	0	0	0	variable surface protein Vir18, putative
PVX_021680	0	0	0	0	0	0	0	0	0	0	0	0	variable surface protein Vir4, putative
PVX_021685	0	0	0	0	0	0	0	0	0	0	0	0	variable surface protein Vir5, putative

Gene ID / Patient	Patient 1	Patient 2	Patient 3	Patient 4	Patient 5	Patient 6	Patient 7	Patient 8	Patient 9	Patient 10	Patient 11	Patient 12	Gene Description
PVX_022185	59.3553	165.77	0	189.758	37.6628	0	0	0	67.4707	62.5626	129.962	0	variable surface protein Vir12-related
PVX_022685	105.925	18.4879	0	0	0	271.029	0	0	60.2009	9.30433	0	0	variable surface protein Vir24-related
PVX_023185	0	0	0	0	0	0	0	0	0	0	0	0	variable surface protein Vir12-related
PVX_023685	0	0	0	0	0	0	0	0	0	0	0	0	variable surface protein Vir7, putative
PVX_024185	0	0	0	0	0	0	0	0	0	0	0	0	variable surface protein Vir6-like
PVX_024685	84.6868	0	0	0	0	0	0	0	64.1883	59.5011	33.715	0	variable surface protein Vir12-related
PVX_024690	0	0	0	0	0	0	0	0	0	0	0	0	variable surface protein Vir12-related
PVX_025190	0	0	689.606	0	0	0	0	0	0	0	0	0	Pvstp1, truncated, putative
PVX_025690	0	0	0	0	0	0	0	0	0	0	0	0	variable surface protein Vir21, putative
PVX_026190	0	0	0	0	0	0	0	0	0	0	0	0	variable surface protein Vir14, putative
PVX_026690	0	0	0	0	0	0	0	0	0	0	0	0	variable surface protein Vir6, putative
PVX_027190	0	0	0	0	0	0	0	0	0	0	0	0	hypothetical protein
PVX_027690	0	0	0	0	0	0	0	0	0	0	0	0	variable surface protein Vir16, putative
PVX_028190	0	0	0	0	0	0	0	0	0	0	0	0	variable surface protein Vir4, putative
PVX_028690	0	0	0	0	0	0	0	0	0	0	0	0	variable surface protein Vir6, putative
PVX_029190	0	0	0	0	0	0	0	0	0	0	0	0	variable surface protein Vir14, putative
PVX_029690	0	0	0	0	0	0	0	0	0	0	0	0	variable surface protein Vir6, putative
PVX_030190	0	0	0	0	0	0	0	0	0	0	0	0	variable surface protein Vir14, truncated, putative
PVX_030690	0	0	0	0	0	0	0	0	0	0	0	0	variable surface protein Vir6, putative
PVX_031190	0	0	0	0	0	0	0	0	0	0	0	0	variable surface protein Vir6, putative
PVX_031690	0	0	0	0	0	0	0	0	0	0	0	0	variable surface protein Vir30, putative
PVX_032190	0	0	0	0	0	0	0	0	0	0	20.6562	0	variable surface protein Vir28-related
PVX_032690	0	0	0	0	0	0	0	0	0	0	0	0	variable surface protein Vir7, putative
PVX_033190	0	0	0	0	0	0	0	0	0	0	0	0	variable surface protein Vir21, putative
PVX_033690	0	0	0	0	0	0	0	0	0	0	0	0	variable surface protein Vir6, truncated, putative
PVX_034190	0	0	0	0	0	0	0	0	0	0	0	0	variable surface protein Vir14-related
PVX_034690	0	0	0	0	0	0	0	0	0	0	17.6073	0	hypothetical protein
PVX_035190	0	0	0	0	0	0	0	0	0	0	0	0	variable surface protein Vir6, truncated, putative
PVX_035690	0	0	0	0	0	0	0	0	0	0	0	0	hypothetical protein
PVX_036190	0	0	0	0	0	0	0	0	0	0	0	0	variable surface protein Vir34, putative
PVX_036690	0	645.112	0	0	0	0	0	0	0	0	0	0	hypothetical protein
PVX_037190	0	0	0	0	0	0	0	0	0	0	0	0	variable surface protein Vir14/24-related
PVX_037690	0	0	0	0	0	0	0	0	0	0	0	0	hypothetical protein
PVX_038190	0	0	0	0	0	0	0	0	0	0	0	0	hypothetical protein
PVX_038690	0	0	0	0	0	0	0	0	0	0	0	0	variable surface protein Vir35-like
PVX_039190	0	0	0	0	0	0	0	0	0	0	0	0	variable surface protein Vir6, putative
PVX_039690	0	0	0	0	0	0	0	0	0	0	0	0	variable surface protein Vir27, truncated, putative
PVX_040190	0	0	0	0	0	0	0	0	0	0	0	0	variable surface protein Vir6, truncated, putative
PVX_040690	0	0	0	0	0	0	0	0	0	0	0	0	variable surface protein Vir35, putative
PVX_041190	0	0	0	0	0	0	0	0	0	0	0	0	variable surface protein Vir35, truncated, putative
PVX_041690	0	0	0	0	0	0	0	0	0	0	0	0	variable surface protein Vir12-related, truncated
PVX_042190	0	0	0	0	0	0	0	0	0	0	0	0	variable surface protein Vir12-related, truncated
PVX_042690	0	0	0	0	0	0	0	0	0	0	0	0	variable surface protein Vir30, putative
PVX_043190	0	0	0	0	0	0	0	0	44.8275	41.5375	23.5413	0	variable surface protein Vir12, truncated, putative
PVX_043690	0	0	0	0	0	0	0	0	0	0	0	0	variable surface protein Vir32, truncated, putative
PVX_044190	0	0	0	0	0	0	0	0	0	0	0	0	variable surface protein Vir32, putative
PVX_044690	0	0	0	0	0	0	0	0	0	0	0	0	variable surface protein Vir35, truncated, putative
PVX_045190	0	0	0	0	0	0	0	0	0	0	95.3683	0	variable surface protein Vir18-related
PVX_045690	0	0	0	0	0	0	0	0	0	0	0	0	variable surface protein Vir12-related
PVX_046190	0	0	0	0	0	0	0	0	0	0	0	0	variable surface protein Vir32, putative
PVX_046690	0	0	0	0	0	0	0	0	0	0	0	0	Pvstp1, truncated, putative
PVX_047190	0	0	0	0	0	0	0	0	0	0	0	0	variable surface protein Vir28-like
PVX_047690	0	0	0	0	0	0	0	0	0	0	0	0	variable surface protein Vir10, truncated, putative
PVX_048190	0	0	0	0	0	0	0	0	0	0	0	0	variable surface protein Vir27, truncated, putative
PVX_048690	0	0	0	0	0	0	0	0	0	0	0	0	variable surface protein Vir7-like
PVX_049190	0	0	0	0	0	0	0	0	0	0	0	0	Pv-fam-c protein
PVX_049690	0	69.6376	0	0	0	0	0	0	12.5941	70.0339	0	0	hypothetical protein
PVX_050190	0	0	0	0	0	0	0	0	0	38.347	0	0	variable surface protein Vir4, putative
PVX_050690	0	0	0	0	0	0	0	0	0	0	0	0	variable surface protein Vir6, putative
PVX_051190	0	0	0	0	0	0	0	0	0	0	0	0	variable surface protein Vir23, truncated, putative
PVX_051690	0	0	0	0	0	0	0	0	0	0	0	0	variable surface protein Vir32, putative
PVX_052190	0	0	0	0	0	0	0	0	0	0	0	0	variable surface protein Vir4, pseudogene, putative

Gene ID / Patient	Patient 1	Patient 2	Patient 3	Patient 4	Patient 5	Patient 6	Patient 7	Patient 8	Patient 9	Patient 10	Patient 11	Patient 12	Gene Description
PVX_052690	0	0	0	0	0	0	0	0	0	0	27.9347	0	variable surface protein Vir4-related, truncated
PVX_053190	0	0	0	0	0	0	0	0	0	0	0	0	variable surface protein Vir27, putative
PVX_053690	0	0	0	0	0	0	0	0	0	0	0	0	variable surface protein Vir30, putative
PVX_054190	0	0	0	0	0	0	0	0	34.3375	0	0	0	variable surface protein Vir14-related, truncated
PVX_054690	0	0	0	0	0	0	0	0	0	0	0	0	variable surface protein Vir29, truncated, putative
PVX_055190	0	0	0	0	0	0	0	0	0	0	0	0	variable surface protein Vir18, truncated, putative
PVX_055690	0	0	0	0	0	0	0	0	0	83.162	0	0	variable surface protein Vir27, truncated, putative
PVX_056190	87.8457	0	0	0	0	0	0	0	0	61.7195	0	0	variable surface protein Vir6-related
PVX_056690	0	0	0	0	0	0	0	0	0	0	0	0	variable surface protein Vir30, truncated, putative
PVX_057190	0	0	0	0	0	0	0	0	0	0	0	0	variable surface protein Vir6, truncated, putative
PVX_057690	0	0	0	0	0	0	0	0	0	0	0	0	variable surface protein Vir33, putative
PVX_058190	0	0	0	0	0	0	0	0	0	0	0	0	variable surface protein Vir10/35, truncated, putative
PVX_058690	0	0	0	0	0	0	0	0	0	0	0	0	variable surface protein Vir21, truncated, putative
PVX_059190	0	0	0	0	0	0	0	0	0	0	0	0	variable surface protein Vir6, putative
PVX_059690	0	0	0	0	0	0	0	0	0	0	0	0	variable surface protein Vir26, putative
PVX_060190	0	0	0	0	0	0	0	0	0	0	0	0	hypothetical protein
PVX_060690	0	0	0	0	0	0	0	0	0	0	0	0	variable surface protein Vir18-related
PVX_061190	0	0	0	0	0	0	0	0	0	38.0685	0	0	variable surface protein Vir32, putative
PVX_061690	0	0	0	0	0	0	0	0	0	0	0	0	hypothetical protein
PVX_062190	0	0	0	0	0	0	0	0	0	0	0	0	variable surface protein Vir21, truncated, putative
PVX_062690	75.4736	52.7074	0	0	23.9546	0	0	0	28.6008	0	15.0234	0	variable surface protein Vir18, putative
PVX_063190	0	0	0	0	0	0	0	0	0	0	0	0	variable surface protein Vir12-related
PVX_063690	0	0	0	0	0	0	0	0	0	0	0	0	variable surface protein Vir6-like
PVX_064190	0	0	0	0	0	0	0	0	0	0	0	0	variable surface protein Vir7, putative
PVX_064690	0	0	0	0	0	0	0	0	0	0	0	0	variable surface protein Vir27, putative
PVX_065190	0	0	0	0	0	0	0	0	0	0	0	0	variable surface protein Vir18, truncated, putative
PVX_065690	0	63.6724	0	0	0	0	0	0	34.5475	0	0	147.764	hypothetical protein
PVX_066190	0	0	0	0	0	0	0	104.576	0	0	14.9854	0	variable surface protein Vir18-like
PVX_066690	0	0	0	0	0	0	0	0	0	0	0	0	variable surface protein Vir16, pseudogene, putative
PVX_067190	0	0	0	0	0	0	0	0	0	0	0	0	variable surface protein Vir35, putative
PVX_067690	0	0	0	0	0	0	0	0	0	0	0	0	variable surface protein Vir1-like
PVX_068190	0	0	0	0	0	0	0	0	0	0	0	0	variable surface protein Vir18, putative
PVX_068690	0	0	0	0	0	0	0	0	0	0	0	0	variable surface protein Vir18, putative
PVX_069190	0	0	0	0	0	0	0	0	0	0	0	0	variable surface protein Vir27-like, truncated
PVX_069690	0	0	0	0	0	0	0	0	0	0	0	0	variable surface protein Vir23, putative
PVX_070190	0	0	0	0	0	0	0	0	0	0	0	0	variable surface protein Vir28, truncated, putative
PVX_070690	0	0	0	0	0	0	0	0	0	0	0	0	variable surface protein Vir33, truncated, putative
PVX_071190	0	0	0	0	0	0	0	0	0	0	0	0	variable surface protein Vir28-related
PVX_071690	0	0	0	0	0	0	0	0	0	0	0	0	variable surface protein Vir6 (truncated), putative
PVX_072190	0	0	0	0	0	0	0	0	38.2944	0	0	0	variable surface protein Vir7, putative
PVX_072690	0	0	0	0	0	0	0	0	0	0	0	0	variable surface protein Vir4/14-related
PVX_073190	0	48.9857	0	0	0	0	0	0	0	0	0	0	variable surface protein Vir6-like
PVX_073690	0	57.0411	0	0	0	0	0	0	30.9512	28.6921	0	0	variable surface protein Vir12-related
PVX_074190	0	0	0	0	0	0	0	0	0	0	0	0	hypothetical protein
PVX_074690	0	28.9168	0	0	0	0	0	0	15.6905	0	0	0	variable surface protein Vir4-related
PVX_074695	0	0	0	0	0	0	0	0	0	0	0	0	variable surface protein Vir10, putative
PVX_075195	0	0	0	0	0	0	0	0	0	0	0	0	variable surface protein Vir26, putative
PVX_075695	0	0	0	0	0	0	0	0	0	0	0	0	variable surface protein Vir12/22/24-related
PVX_076195	0	53.1109	0	0	0	0	0	0	0	0	0	0	variable surface protein Vir12-related
PVX_076695	0	0	0	0	0	0	0	0	0	0	0	0	variable surface protein Vir14/32-related
PVX_077195	0	0	0	0	0	0	0	0	0	0	0	0	Pvstp1, putative
PVX_077695	0	21.7764	0	0	19.791	0	0	0	23.635	10.9575	12.4159	0	variable surface protein Vir12-like
PVX_078195	0	0	0	0	0	0	0	0	0	0	0	0	variable surface protein Vir12-related
PVX_078695	0	0	0	0	0	0	0	0	0	0	0	0	variable surface protein Vir29-like
PVX_079195	0	0	0	0	26.2866	0	0	0	0	0	0	0	variable surface protein Vir6-like
PVX_079695	25.1545	52.6661	0	80.3412	7.97395	0	38.4343	34.9249	19.058	26.5182	0	0	hypothetical protein, conserved
PVX_079700	0	0	0	0	0	0	0	0	0	29.4166	16.6682	0	hypothetical protein, conserved
PVX_079705	0	0	0	0	0	0	0	234.437	63.9375	59.2094	67.1352	0	30S ribosomal protein S16-related, putative
PVX_079710	0	0	0	0	0	0	0	0	0	0	0	0	hypothetical protein, conserved
PVX_079715	0	29.7661	0	0	13.523	0	0	0	32.3105	29.9661	16.9751	0	hypothetical protein, conserved
PVX_079720	23.2942	0	0	0	0	0	0	16.1689	0	0	0	0	hypothetical protein, conserved
PVX_079725	0	0	321.481	0	0	0	0	0	0	0	25.6795	0	hypothetical protein, conserved

Gene ID / Patient	Patient 1	Patient 2	Patient 3	Patient 4	Patient 5	Patient 6	Patient 7	Patient 8	Patient 9	Patient 10	Patient 11	Patient 12	Gene Description
PVX_079730	28.9084	40.3525	0	0	0	0	0	40.1384	0	10.1584	17.262	0	hypothetical protein, conserved
PVX_079735	0	0	0	0	0	0	0	0	28.5286	0	44.9563	0	XAP-5 DNA binding protein, putative
PVX_079740	0	0	0	0	0	0	0	0	0	0	0	0	hypothetical protein, conserved
PVX_079745	0	0	0	0	0	0	0	28.2462	0	21.4487	12.1485	0	hypothetical protein, conserved
PVX_079750	0	0	63.2887	0	0	0	0	0	9.64834	0	15.2084	0	hypothetical protein, conserved
PVX_079755	0	0	0	0	0	0	0	87.5505	23.8848	110.732	50.1882	102.097	phosphoenolpyruvate/phosphate translocator, putative (PPT)
PVX_079760	0	0	0	0	0	0	0	0	142.978	66.1869	75.0561	0	hypothetical protein, conserved
PVX_079765	122.971	0	0	0	0	0	188.479	170.9	233.077	129.575	73.4387	0	hypothetical protein, conserved
PVX_079770	274.622	191.936	0	0	0	0	0	0	0	144.674	27.3346	0	hypothetical protein, conserved
PVX_079772	0	0	0	0	0	0	0	0	0	0	3.38467	0	conserved Plasmodium protein, unknown function
PVX_079775	30.0843	0	0	0	19.0757	0	0	0	11.3969	0	23.9522	0	hypothetical protein, conserved
PVX_079778	0	0	0	0	0	0	0	0	0	0	0	0	tRNA Arginine
PVX_079780	0	0	0	0	150.924	0	0	0	179.933	277.747	125.962	0	cell cycle regulator protein, putative
PVX_079785	0	0	0	0	6.14933	0	29.6385	26.9356	14.6986	13.6358	15.4465	0	hypothetical protein, conserved
PVX_079790	0	0	0	0	0	0	0	0	0	0	0	0	apicoplast TIC22 protein, putative
PVX_079795	0	166.675	0	0	0	0	0	0	0	167.208	47.4186	0	hypothetical protein, conserved
PVX_079800	0	0	0	0	14.2431	459.527	0	62.365	0	47.3395	0	0	T-cell immunomodulatory protein homolog precursor, putative
PVX_079805	0	0	0	0	0	0	0	0	0	0	0	0	hypothetical protein, conserved
PVX_079810	63.9807	44.6746	0	204.572	20.3011	0	0	177.731	0	44.9576	12.7354	0	hypothetical protein, conserved
PVX_079815	0	61.4781	0	0	27.9448	0	0	0	33.3575	0	17.5208	0	cyclophilin, putative
PVX_079820	104.7	0	0	0	0	0	0	0	39.6842	0	20.8419	0	hypothetical protein, conserved
PVX_079825	0	0	0	0	0	0	0	0	0	0	0	0	F-actin capping protein, alpha subunit, putative
PVX_079830	0	0	0	0	0	0	0	0	0	0	0	0	palmitoyltransferase, putative (DHHC7)
PVX_079835	0	0	0	0	0	0	0	0	0	0	0	0	hypothetical protein, conserved
PVX_079840	267.024	223.709	132.809	0	101.646	0	0	148.337	121.408	262.697	191.343	0	eukaryotic translation initiation factor 3, subunit 6, putative
PVX_079845	0	72.3604	0	110.4	21.9139	0	0	0	65.458	48.5714	27.5132	55.927	AP-1 complex subunit beta, putative
PVX_079850	402.666	0	334.549	0	42.6622	0	0	186.556	101.768	424.264	213.756	0	hypothetical protein, conserved
PVX_079855	40.4992	28.2701	100.679	129.407	12.8431	0	0	0	46.0305	14.2306	16.1224	0	RNA helicase-1, putative
PVX_079857	0	0	0	0	0	0	0	0	0	0	0	0	tRNA Leucine
PVX_079858	0	0	0	0	0	0	0	0	0	0	0	0	tRNA Glutamic acid
PVX_079860	0	0	0	0	0	0	0	0	0	0	0	0	hypothetical protein, conserved
PVX_079865	479.936	209.437	149.218	191.797	57.101	0	91.7655	249.967	454.631	337.245	298.535	0	Hsc70-interacting protein, putative (HIP)
PVX_079870	0	0	0	0	0	0	0	0	504.19	279.82	105.824	0	hypothetical protein, conserved
PVX_079875	0	0	0	0	0	0	356.779	0	176.509	40.8895	0	0	methionine aminopeptidase 1a, putative (METAP1a)
PVX_079880	131.688	30.6426	0	0	0	0	67.1103	0	49.8925	77.1194	26.212	71.0606	ubiquitin carboxyl-terminal hydrolase, putative
PVX_079885	366.208	0	457.017	0	58.2693	0	0	0	138.877	128.585	109.358	297.58	ubiquitin-conjugating enzyme E2 N, putative (UBC13)
PVX_079890	0	23.026	0	0	0	0	50.4189	45.8072	12.4979	0	45.9648	0	DNA replication licensing factor MCM3, putative
PVX_079895	0	0	0	0	64.9429	0	0	0	0	71.6119	81.2161	0	transmembrane emp24 domain-containing protein, putative
PVX_079900	134.097	0	0	0	0	0	0	0	50.8131	70.6688	120.115	0	hypothetical protein, conserved
PVX_079905	94.8146	132.381	0	0	60.1451	0	0	197.506	53.8846	83.2866	75.4902	76.7523	hypothetical protein, conserved
PVX_079910	0	6.32432	22.5121	0	2.87208	0	0	12.5823	3.43315	3.18532	9.02013	0	hypothetical protein, conserved
PVX_079915	0	0	0	0	0	0	0	0	0	0	0	0	hypothetical protein
PVX_079920	50.2524	0	62.4352	0	31.8599	0	0	0	0	35.318	55.0126	0	hypothetical protein, conserved
PVX_079925	100.695	70.3437	0	0	31.9794	0	0	139.91	38.165	106.114	80.1778	163.27	hypothetical protein, conserved
PVX_079930	132.215	92.3218	164.457	0	0	0	101.14	0	25.0498	162.58	26.3176	107.084	WD domain, G-beta repeat domain containing protein
PVX_079935	0	0	0	0	0	0	0	0	73.202	22.6243	51.2717	0	ADP-ribosylation factor GTPase-activating protein, putative (ARF-GAP)
PVX_079940	0	0	0	321.208	31.8716	0	0	139.439	76.0731	35.2524	39.954	0	hypothetical protein, conserved
PVX_079945	0	0	123.991	0	15.8164	0	0	0	18.8926	52.5607	39.7011	0	hypothetical protein, conserved
PVX_079950	0	0	0	0	0	0	0	0	0	0	0	0	serine/threonine-protein kinase Nek1, putative
PVX_079955	0	0	0	0	0	0	0	0	0	0	25.3505	0	inner membrane complex protein 1g, putative (IMC1g)
PVX_079960	0	0	0	0	0	0	0	0	63.1042	58.4393	66.2611	270.338	hypothetical protein, conserved
PVX_079965	0	0	0	0	0	0	0	0	38.0365	0	0	0	RNA methyltransferase, putative
PVX_079970	0	35.8795	0	0	10.8637	0	26.1799	23.7936	12.9841	0	23.8787	0	hypothetical protein, conserved
PVX_079975	0	0	0	0	0	0	113.489	0	28.1028	52.1078	29.5238	0	G-protein coupled receptor, putative
PVX_079980	65.383	0	0	209.064	0	0	0	0	0	0	26.0292	0	hypothetical protein, conserved
PVX_079985	0	0	45.3215	0	0	0	0	0	6.9103	6.41075	7.26197	29.5104	hypothetical protein, conserved
PVX_079990	95.523	166.713	118.756	152.643	45.4463	0	73.0289	0	72.3832	67.127	76.0543	77.3265	long-chain fatty acid CoA ligase, putative
PVX_079995	0	0	0	0	0	0	0	0	0	0	5.5967	0	hypothetical protein, conserved
PVX_080000	0	27.4982	97.9278	0	0	0	0	0	0	41.5268	23.5235	127.529	hypothetical protein, conserved
PVX_080005	0	0	198.902	255.658	0	0	0	0	0	28.0774	47.7269	0	hypothetical protein, conserved
PVX_080010	0	33.6844	0	0	15.3041	0	0	0	54.8437	16.9536	48.0209	0	hypothetical protein, conserved
PVX_080015	0	285.603	0	0	130.308	0	0	0	309.337	142.825	162.189	0	mitochondrial import receptor subunit TOM22, putative

Gene ID / Patient	Patient 1	Patient 2	Patient 3	Patient 4	Patient 5	Patient 6	Patient 7	Patient 8	Patient 9	Patient 10	Patient 11	Patient 12	Gene Description
PVX_080020	166.42	0	0	0	0	0	0	231.381	0	0	66.2611	0	50S ribosomal protein L12, apicoplast, putative
PVX_080025	0	0	0	0	0	0	0	0	451.652	0	157.88	0	hypothetical protein, conserved
PVX_080030	33.3399	69.8714	0	0	0	0	0	0	12.6363	11.7114	66.3667	0	developmentally regulated GTP-binding protein 1, putative
PVX_080035	80.7879	112.845	201.062	0	25.6447	0	0	0	153.079	85.1442	48.2439	0	hypothetical protein, conserved
PVX_080040	0	0	0	0	0	0	0	0	0	0	0	0	hypothetical protein, conserved
PVX_080045	0	19.2911	0	0	8.76258	0	42.2363	0	20.9419	0	0	89.4503	hypothetical protein, conserved
PVX_080050	56.4315	196.926	70.1183	180.253	35.7802	144.284	43.116	78.3528	171.022	148.725	106.706	45.6566	karyopherin beta, putative,importin-beta 3, putative
PVX_080055	0	0	0	0	0	0	0	0	0	0	0	0	hypothetical protein, conserved
PVX_080060	36.017	100.559	89.5247	0	0	0	165.152	50.0119	40.9348	101.247	21.5069	58.2927	metal transporter, putative
PVX_080065	0	0	0	0	0	0	37.975	0	37.6607	26.2016	9.89396	0	hypothetical protein, conserved
PVX_080070	0	0	0	0	0	0	0	0	0	33.2757	37.7124	153.566	hypothetical protein, conserved
PVX_080075	0	0	0	0	0	0	0	0	0	0	0	0	hypothetical protein, conserved
PVX_080080	129.069	45.0615	0	206.345	40.9542	0	0	0	24.4535	22.6732	51.3826	0	hypothetical protein, conserved
PVX_080085	0	0	913.574	0	0	0	0	0	0	0	145.028	594.861	hypothetical protein, conserved
PVX_080090	63.7927	0	79.2732	101.894	0	0	48.746	0	24.167	22.4164	44.4412	0	hypothetical protein, conserved
PVX_080095	127.136	88.7483	0	135.419	40.319	0	129.575	117.698	96.3346	59.5632	92.7882	0	mitochondrial processing peptidase alpha subunit, putative
PVX_080100	42.6997	89.3965	53.0459	0	40.6036	0	32.6172	148.207	72.7878	67.5234	84.9896	34.5401	multidrug resistance protein (MDR1)
PVX_080105	0	11.1424	0	0	5.06052	0	0	0	30.2419	16.8339	25.4251	25.8284	hypothetical protein, conserved
PVX_080110	151.746	0	0	0	96.5061	0	232.796	210.949	57.5344	159.872	151.044	0	G10 protein, putative
PVX_080115	0	0	0	0	0	0	0	726.709	99.0696	0	51.9855	0	iron-sulfur cluster assembly accessory protein, putative (SufA)
PVX_080120	0	0	0	0	0	0	0	0	26.7711	0	56.2505	0	hypothetical protein, conserved
PVX_080125	0	0	0	0	0	0	463.294	0	0	0	59.8451	0	50S ribosomal protein L17, apicoplast, putative (RPL17)
PVX_080130	3.36419	2.34722	0	0	0	0	0	0	0	0	1.33919	0	hypothetical protein, conserved
PVX_080135	101.957	71.2264	0	0	0	0	0	0	38.6436	35.8144	40.5914	0	S-adenosylmethionine-dependent methyltransferase, putative
PVX_080140	0	141.708	0	0	0	0	0	0	0	0	80.6644	0	transcription initiation factor TFIID subunit 10, putative (TAF10)
PVX_080145	0	0	0	0	0	0	0	0	0	0	0	0	hypothetical protein, conserved
PVX_080147	0	0	0	0	0	0	0	0	0	238.959	0	0	conserved Plasmodium protein, unknown function
PVX_080150	0	0	0	0	0	0	0	0	0	0	0	0	hypothetical protein, conserved
PVX_080155	0	0	0	0	15.8962	0	0	0	18.9878	17.6085	19.9506	0	hypothetical protein, conserved
PVX_080160	0	25.7198	0	0	23.3679	0	0	51.1655	27.9193	51.7905	14.6685	0	DEAD/DEAH box ATP-dependent RNA helicase, putative
PVX_080165	0	0	0	0	0	0	0	0	0	0	210.144	0	hypothetical protein, conserved
PVX_080170	68.3805	0	0	0	21.6995	0	0	0	77.7346	0	54.4452	0	ribosomal large subunit pseudouridylate synthase, putative
PVX_080175	0	35.1839	0	0	15.9858	0	0	0	38.1896	17.7077	60.1889	0	hypothetical protein, conserved
PVX_080180	0	14.8216	52.7688	67.8262	6.73191	0	0	88.4599	8.0453	22.3903	25.3638	0	hypothetical protein, conserved
PVX_080185	0	0	0	0	0	0	0	0	28.2198	26.1623	44.47	0	hypothetical protein, conserved
PVX_080190	0	0	0	0	12.7839	0	0	27.998	0	0	12.0417	0	hypothetical protein, conserved
PVX_080195	0	64.7153	115.246	0	0	0	0	0	52.6842	16.2864	27.6783	0	hypothetical protein, conserved
PVX_080200	403.531	28.1681	100.315	0	153.56	0	123.373	168.106	366.915	467.915	409.637	195.956	adenosylhomocysteinase(S-adenosyl-L-homocystein e hydrolase), putative
PVX_080205	0	10.5923	0	0	9.62131	0	0	0	17.2495	5.33436	15.1063	0	hypothetical protein, conserved
PVX_080210	33.0264	0	0	0	0	0	0	137.574	0	58.026	13.1474	0	hypothetical protein, conserved
PVX_080215	0	345.209	0	0	0	0	0	0	62.4071	57.795	65.5298	0	bis(5'-nucleosyl)-tetraphosphatase [asymmetrical], putative
PVX_080220	0	0	0	0	0	0	0	0	0	0	46.7122	0	phosphomethylpyrimidine kinase, putative
PVX_080225	349.239	0	0	372.769	0	0	0	0	88.2544	40.8895	185.39	0	hypothetical protein, conserved
PVX_080230	197.227	0	0	0	62.7856	0	0	0	149.604	207.741	157.061	320.661	U6 snRNA-associated Sm-like protein LSm4, putative
PVX_080235	47.9406	33.4677	119.202	0	30.4112	0	0	66.5764	127.146	134.757	57.2546	155.234	hypothetical protein, conserved
PVX_080240	60.4735	105.519	0	0	0	0	0	0	34.364	53.1255	18.0552	0	protein phosphatase 2C, putative
PVX_080245	1718.94	901.11	357.13	918.071	364.323	735.03	659.084	1991.15	1140.47	2062.6	1026.58	930.16	40S ribosomal protein S9, putative
PVX_080250	0	0	0	0	0	0	0	0	79.9344	98.8134	97.9744	0	hypothetical protein, conserved
PVX_080255	22.7328	0	0	0	14.4117	232.455	0	0	25.8344	7.98852	31.6732	0	hypothetical protein, conserved
PVX_080260	0	609.976	0	558.834	221.81	0	267.402	363.979	364.066	337.471	556.287	283.096	hypothetical protein, conserved
PVX_080265	0	185.862	0	0	0	0	135.687	123.245	50.4369	31.1842	79.4939	71.8368	zinc finger protein, putative
PVX_080270	0	0	0	0	0	0	0	0	29.8337	0	4.47903	0	hypothetical protein, conserved
PVX_080275	523.539	1189.1	652.4	1257.84	83.1962	671.357	401.317	363.823	992.354	1103.26	364.771	212.401	40S ribosomal protein S24, putative (RPS24)
PVX_080280	0	0	0	0	19.7496	0	0	0	0	21.8692	37.1699	0	cytochrome c oxidase assembly protein (heme A: farnesyltransferase), putative
PVX_080285	117.637	246.592	0	0	74.7512	0	0	0	445.916	289.234	210.758	0	V-type proton ATPase 16 kDa proteolipid subunit, putative
PVX_080290	0	0	0	0	0	0	0	0	0	0	210.842	0	50S ribosomal subunit protein L14, putative
PVX_080295	0	0	387.402	248.973	24.7061	0	0	108.126	88.4903	27.3451	61.9752	0	hypothetical protein, conserved
PVX_080300	0	0	0	0	0	0	0	0	0	0	0	0	hypothetical protein, conserved
PVX_080305	0	0	0	0	0	0	0	0	0	0	0	0	hypothetical protein, conserved
PVX_080310	0	0	0	0	0	0	0	0	0	0	0	0	RNA-binding protein, putative
PVX_080315	23.9559	16.7186	0	76.5106	7.59378	0	0	0	36.2996	25.2549	66.7549	0	hypothetical protein, conserved
PVX_080320	0	0	0	0	0	0	0	0	0	11.1605	6.32171	0	ATP-dependent RNA helicase DDX23, putative

Gene ID / Patient	Patient 1	Patient 2	Patient 3	Patient 4	Patient 5	Patient 6	Patient 7	Patient 8	Patient 9	Patient 10	Patient 11	Patient 12	Gene Description
PVX_080325	0	0	0	0	0	0	0	0	0	0	0	0	cyclin, putative
PVX_080330	62.9014	43.9204	0	603.345	59.8745	0	96.2248	262.097	95.3378	265.197	250.412	305.645	proteasome subunit beta type-1, putative
PVX_080335	140.546	0	0	0	0	0	0	0	53.2836	0	111.914	228.14	SWIB/MDM2 domain-containing protein, putative
PVX_080340	0	0	0	0	0	0	39.7539	0	9.85595	9.14261	25.8927	0	hypothetical protein, conserved
PVX_080345	0	0	728.305	0	0	0	224.017	0	0	0	58.1493	0	zinc finger protein, putative
PVX_080350	0	0	0	0	0	0	93.0783	0	0	42.7574	72.6716	98.5509	zinc finger protein, putative
PVX_080355	0	0	0	0	6.98225	0	0	61.1656	0	7.7408	26.3065	0	transcription factor with AP2 domain(s), putative (ApiAP2)
PVX_080360	0	0	0	555.441	0	0	0	0	0	0	68.9544	0	apicortin, putative
PVX_080365	206.543	240.273	513.364	219.951	65.4888	0	105.226	143.397	273.866	254.019	198.703	278.558	Eukaryotic translation initiation factor 3 subunit 9, putative
PVX_080370	0	0	0	0	0	0	0	0	0	0	0	0	f-actin capping protein beta subunit, putative
PVX_080375	0	0	0	0	0	0	0	0	0	14.1984	0	0	UTP--glucose-1-phosphate uridylyltransferase, putative
PVX_080380	30.1552	21.0468	0	96.3268	19.1207	0	46.0825	0	57.1189	31.7894	66.0239	0	FACT complex subunit SPT16, putative (FACT-L)
PVX_080385	723.903	168.612	0	0	38.3358	0	0	0	91.4695	84.7539	168.122	0	pre-mRNA-splicing factor (SR1)
PVX_080390	0	42.0566	0	0	0	0	0	0	0	0	23.9793	0	hypothetical protein, conserved
PVX_080395	0	0	0	0	0	0	0	0	0	0	55.5922	113.111	hypothetical protein, conserved
PVX_080400	1078.76	880.099	448.712	2883.75	171.633	923.583	1380.35	1500.04	818.179	1010.1	1252.77	292.172	60S ribosomal protein L12, putative
PVX_080405	505.751	353.311	503.765	647.513	160.622	0	309.851	702.717	690.078	959.342	604.052	492.03	60S ribosomal protein L2, putative (RPL2)
PVX_080410	12.8072	0	31.8131	0	0	0	0	0	4.8512	0	12.7456	0	transcription factor with AP2 domain(s), putative (ApiAP2)
PVX_080415	23.3192	48.8225	57.9421	74.4757	36.9592	119.228	0	32.3762	61.8356	65.5566	37.1318	0	ubiquitin carboxyl-terminal hydrolase 2, putative
PVX_080420	45.9205	16.0236	0	0	14.556	0	35.0793	31.8777	52.1859	32.2738	22.8501	0	MB2 protein, putative
PVX_080425	0	0	0	0	0	0	0	0	0	0	0	0	transporter, putative
PVX_080430	102.424	71.5529	0	0	97.5894	0	156.882	284.629	0	143.913	81.5544	0	phosducin-like protein, putative (PhLP3)
PVX_080435	0	130.658	0	0	19.791	0	0	0	47.27	43.8299	37.2476	0	tRNA pseudouridine synthase, putative
PVX_080440	664.443	580.699	828.809	1065.31	158.518	0	0	692.851	692.857	816.633	925.933	269.833	40S ribosomal protein S11, putative
PVX_080445	0	24.4603	0	0	11.1116	0	0	0	13.2762	12.314	6.97526	0	RAP protein, putative
PVX_080450	56.1877	39.2293	139.741	179.616	53.4751	287.563	0	78.0358	149.027	78.9664	145.394	0	NLI interacting factor-like phosphatase, putative (NIF2)
PVX_080455	1320.13	0	0	4364.6	0	0	0	0	0	0	263.183	0	BolA-like protein, putative
PVX_080460	0	0	0	0	0	0	114.921	0	0	26.3817	14.9477	0	glideosome-associated protein 40, putative (GAP40)
PVX_080465	0	0	0	0	0	0	0	26.9882	0	0	3.86918	0	hypothetical protein, conserved
PVX_080470	0	0	0	0	0	0	0	0	0	0	0	0	hypothetical protein, conserved
PVX_080475	0	0	0	0	0	0	0	0	0	0	0	0	hypothetical protein, conserved
PVX_080480	0	24.3156	0	55.6328	0	0	0	0	19.7985	12.245	24.2739	0	phosphatidylinositol 3-kinase, putative
PVX_080485	180.155	0	449.62	0	0	0	0	0	0	63.2583	107.597	0	hypothetical protein, conserved
PVX_080490	0	0	0	0	0	0	0	0	0	0	0	0	rhomboid protease ROM9, putative (ROM9)
PVX_080495	0	59.6459	106.212	0	13.5489	0	0	0	32.3722	0	34.015	0	RNA-binding protein, putative
PVX_080500	0	39.8045	141.792	0	36.1731	0	0	79.1798	43.2032	0	56.7401	0	hypothetical protein, conserved
PVX_080505	0	45.6982	0	0	20.7587	0	0	0	12.4018	23.0067	13.0319	0	hypothetical protein, conserved
PVX_080510	0	0	0	0	0	0	0	31.5138	8.59837	0	0	0	hypothetical protein, conserved
PVX_080515	0	0	0	0	0	899.952	269.005	0	0	61.5259	69.7646	0	ribose 5-phosphate epimerase, putative
PVX_080520	0	0	0	0	0	0	0	0	0	0	0	0	hypothetical protein, conserved
PVX_080523	0	0	0	0	0	0	0	0	0	0	0	0	tRNA Phenylalanine
PVX_080525	30.4321	63.7203	0	97.212	28.9446	0	0	126.764	46.1148	53.4686	54.5156	0	aspartate--tRNA ligase, putative
PVX_080530	0	33.414	0	0	0	0	0	0	36.269	0	9.52713	0	hypothetical protein, conserved
PVX_080535	0	0	0	97.1128	0	0	0	42.2116	0	0	18.1533	0	ATP-dependent DNA helicase UvrD, putative (UvrD)
PVX_080540	0	0	0	0	0	0	0	104.312	0	0	59.7909	0	tubulin tyrosine ligase-like protein 1, putative
PVX_080545	323.807	0	0	0	51.4958	0	0	0	122.78	56.8548	96.6939	0	hypothetical protein, conserved
PVX_080550	425.392	0	531.321	682.931	67.7357	0	0	0	0	0	42.3457	0	ras-related protein Rab-1A, putative (RAB1a)
PVX_080555	0	0	0	0	0	0	0	0	0	13.8606	0	0	6-cysteine protein,secreted ookinete protein, putative (PSOP12)
PVX_080560	26.629	0	66.1721	0	8.44167	0	0	0	10.0877	9.35749	47.7024	0	deoxyribodipyrimidine photo-lyase, putative
PVX_080565	0	371.801	0	0	0	0	0	0	201.201	0	316.263	0	mitochondrial import inner membrane translocase subunit TIM16, putative (PAM16)
PVX_080570	0	0	0	0	0	0	0	0	0	0	26.8403	0	GTP-binding protein, putative
PVX_080575	314.838	146.635	261.363	335.942	66.6659	537.888	321.517	583.291	755.77	663.549	292.471	170.183	purine nucleoside phosphorylase, putative (PNP)
PVX_080580	13.0824	18.2576	0	41.7699	4.14588	0	0	54.485	0	22.9875	28.6428	0	hypothetical protein, conserved
PVX_080585	0	0	0	0	0	0	0	0	0	0	31.7148	0	hypothetical protein, conserved
PVX_080590	0	0	0	0	0	0	0	0	0	0	36.0339	0	hypothetical protein, conserved
PVX_080595	0	0	0	0	0	0	0	0	0	0	26.1315	0	hypothetical protein, conserved
PVX_080600	0	0	0	0	0	0	0	0	35.9817	41.7229	51.991	0	hypothetical protein, conserved
PVX_080605	0	0	0	0	0	0	0	0	0	0	31.2146	0	orotate phosphoribosyltransferase, putative (OPRT)
PVX_080610	0	0	0	0	0	0	0	0	0	68.3442	38.7527	316.454	ras-related protein Rab-1B, putative (RAB1b)
PVX_080615	0	0	0	0	0	0	0	0	28.3158	13.1314	29.7536	0	hypothetical protein, conserved
PVX_080617	0	0	0	0	0	0	0	0	0	0	9.68249	0	conserved Plasmodium protein, unknown function
PVX_080620	0	0	0	0	0	0	0	0	0	0	0	0	hypothetical protein, conserved

Gene ID / Patient	Patient 1	Patient 2	Patient 3	Patient 4	Patient 5	Patient 6	Patient 7	Patient 8	Patient 9	Patient 10	Patient 11	Patient 12	Gene Description
PVX_080625	0	0	227.863	0	29.062	0	140.147	254.327	34.6889	64.3064	36.4394	0	CDK-activating kinase assembly factor, putative
PVX_080630	0	0	0	0	0	0	77.0223	0	38.1681	17.6977	30.0775	0	glutathione synthetase, putative
PVX_080635	254.418	0	0	0	81.1058	0	0	0	193.061	89.3011	0	414.313	hypothetical protein, conserved
PVX_080640	551.766	387.698	2783.19	0	531.565	0	1714.78	770.048	209.776	0	329.701	0	prefoldin subunit 6, putative
PVX_080645	49.4505	0	0	0	0	0	0	68.6743	0	0	9.84301	0	hypothetical protein, conserved
PVX_080650	525.93	842.115	538.209	494.132	186.36	791.064	425.506	730.252	808.682	782.722	652.727	400.511	inositol-3-phosphate synthase, putative (INO1)
PVX_080655	0	0	0	0	0	0	0	366.571	199.892	92.4509	52.4447	0	EKC/KEOPS complex subunit CGI121, putative (CGI121)
PVX_080657	0	0	0	0	0	0	0	0	86.218	0	0	0	apical rhoptry neck protein, putative (ARNP)
PVX_080660	2.62108	10.9724	13.0178	25.0986	5.81295	0	32.0157	3.63835	21.8408	13.8175	13.0422	0	RNA pseudouridylate synthase, putative
PVX_080665	0	0	0	0	0	0	0	0	0	21.5545	0	99.363	hypothetical protein, conserved
PVX_080670	22.8372	0	0	0	0	0	0	0	0	0	4.54553	0	hypothetical protein, conserved
PVX_080675	68.6363	71.8607	0	0	10.8813	0	52.4512	47.6522	39.0036	36.1771	95.6314	0	stearoyl-CoA desaturase (acyl-CoA desaturase, faty acid desaturase), putative
PVX_080680	0	0	0	0	0	0	0	0	43.8418	0	23.024	0	hypothetical protein, conserved
PVX_080685	558.805	312.341	0	715.652	35.5035	0	171.235	155.303	84.7249	196.282	200.228	543.806	translationally controlled tumor protein, putative
PVX_080690	0	0	0	0	0	0	0	0	0	0	0	0	WD-repeat potein, putative
PVX_080700	0	0	0	0	0	0	0	0	0	0	0	0	hypothetical protein, conserved
PVX_081200	0	0	0	0	0	0	0	0	0	0	0	0	hypothetical protein, conserved
PVX_081205	0	0	0	0	0	0	319.501	0	26.3753	48.9073	55.4192	112.759	TatD-like deoxyribonuclease, putative
PVX_081210	0	0	0	0	0	0	0	582.007	0	220.355	41.6526	0	hypothetical protein, conserved
PVX_081215	0	34.0147	121.152	0	30.9086	0	74.5023	135.329	36.9209	85.5986	87.2848	0	hypothetical protein, conserved
PVX_081220	0	423.683	0	0	0	0	937.868	0	229.178	0	240.063	0	hypothetical protein, conserved
PVX_081225	0	0	0	0	95.2098	0	229.665	208.122	56.7636	157.734	89.4132	0	hypothetical protein, conserved
PVX_081230	0	0	191.53	0	0	0	0	0	58.3336	27.0394	15.3206	0	UMP-CMP kinase, putative
PVX_081235	44.3749	0	0	141.806	28.1469	0	0	0	16.8123	0	26.498	0	hypothetical protein, conserved
PVX_081240	49.5842	0	61.6044	0	15.718	126.764	0	0	0	17.4242	19.7386	0	replication factor c, putative
PVX_081245	0	0	0	0	70.9566	0	342.509	0	0	78.1997	177.396	0	hypothetical protein, conserved
PVX_081250	0	0	0	0	0	0	0	0	0	0	4.17772	0	kinesin-8, putative
PVX_081255	236.281	496.052	0	0	75.2905	0	0	0	89.638	0	47.0429	0	adenylate kinase-like protein 1, putative (AKLP1)
PVX_081260	0	0	0	0	0	0	152.675	0	113.346	0	19.8435	161.628	transcription initiation factor TFIIB, putative
PVX_081265	182.794	0	303.117	584.417	135.325	0	186.411	169.256	392.49	428.156	351.724	394.741	chromatin assembly factor 1 protein WD40 domain, putative
PVX_081270	41.3962	28.8888	0	66.0996	13.1211	0	31.6208	0	39.203	14.5472	0	0	phosphatidylinositol-4-phosphate 5-kinase, putative (PIP5K)
PVX_081275	0	28.1085	0	0	6.38331	0	30.7664	0	7.62884	0	12.0255	0	bromodomain protein, putative
PVX_081277	80.5148	281.45	0	258.143	76.8251	0	247.111	0	152.645	84.8222	96.1718	0	DNA-directed RNA polymerase 2, putative
PVX_081280	0	0	0	0	133.997	0	0	1166.97	159.015	0	0	0	FAD-linked sulfhydryl oxidase ERV1, putative (ERV1)
PVX_081285	168.24	0	104.563	0	0	0	0	58.4065	47.805	14.7789	16.7437	68.0848	selenocysteine-specific elongation factor selB homologue, putative
PVX_081290	178.36	0	0	0	0	0	0	0	0	0	71.0163	0	hypothetical protein
PVX_081295	0	0	0	0	0	0	0	0	0	0	26.2636	0	hypothetical integral membrane protein, DUF56 family, putative
PVX_081300	237.579	0	147.73	189.884	75.3756	0	90.8502	0	90.0207	104.34	189.16	0	phenylalanyl-tRNA synthetase beta chain, putative
PVX_081305	0	0	323.823	0	0	0	0	0	0	136.907	77.5977	421.706	hypothetical protein, conserved
PVX_081307	0	0	0	0	0	0	0	538.042	0	135.45	153.793	0	cold-shock protein, putative
PVX_081310	0	0	0	0	0	0	498.813	900.562	368.244	113.462	128.776	0	N-acetyltransferase, putative
PVX_081315	0	0	421.478	0	0	0	0	0	0	0	67.2619	0	tubulin-specific chaperone a, putative
PVX_081320	119.204	83.2937	296.991	0	0	0	0	0	90.3721	125.608	355.942	193.382	elongation of very long chain fatty acids protein 3, putative
PVX_081325	148.968	0	371.457	0	0	0	0	207.081	169.44	52.3155	207.589	241.869	mRNA cleavage factor-like protein, putative
PVX_081330	0	0	0	0	9.26967	0	0	0	0	0	34.9188	0	LCCL domain-containing protein (CCp5)
PVX_081335	0	0	241.482	0	0	0	0	0	0	0	0	0	hypothetical protein, conserved
PVX_081340	93.052	0	0	0	29.5466	0	0	0	141.065	32.6878	111.137	0	hypothetical protein, conserved
PVX_081345	0	0	0	0	0	0	0	0	11.0766	0	23.2792	0	secreted ookinete protein, putative (PSOP24)
PVX_081350	123.651	86.406	308.109	0	0	0	0	0	93.7463	0	196.919	0	hypothetical protein, conserved
PVX_081355	397.08	277.743	0	0	63.2055	0	0	0	451.803	69.7074	0	0	hypothetical protein
PVX_081360	0	0	0	0	0	0	0	0	0	0	111.937	0	hypothetical protein, conserved
PVX_081365	29.3577	40.9722	36.4643	93.7385	27.912	75.0314	89.6834	81.5132	83.4036	51.5851	73.0414	23.7432	hypothetical protein, conserved
PVX_081370	46.3755	0	0	0	0	0	70.9066	0	70.282	16.2949	83.078	0	hypothetical protein, conserved
PVX_081375	106.065	74.1005	0	339.536	101.068	0	162.479	589.52	241.212	335.311	253.364	0	proteasome subunit beta type-3, putative
PVX_081380	0	131.004	0	0	0	0	0	520.985	71.0395	0	111.877	0	hypothetical protein, conserved
PVX_081385	85.0494	0	0	181.11	0	0	43.321	39.3626	32.2191	29.8864	33.8568	0	double-strand break repair protein MRE11, putative
PVX_081390	0	0	164.457	211.385	0	0	0	91.8216	25.0498	23.2257	26.3176	0	hypothetical protein, conserved
PVX_081395	0	0	0	0	0	0	0	0	3.48504	0	7.32516	0	serine/threonine protein kinase, putative
PVX_081400	0	59.0062	52.5193	0	20.1003	0	0	88.0419	40.0365	37.1409	37.8659	34.1972	patched family protein, putative
PVX_081405	0	0	0	403.867	80.1392	0	193.278	0	47.7985	88.5727	276.105	204.592	hypothetical protein, conserved
PVX_081410	0	0	0	0	0	0	0	0	0	39.0613	22.137	0	carbon catabolite repressor protein 4, putative
PVX_081415	0	0	0	0	0	0	0	101.005	0	0	43.422	0	hypothetical protein, conserved

Gene ID / Patient	Patient 1	Patient 2	Patient 3	Patient 4	Patient 5	Patient 6	Patient 7	Patient 8	Patient 9	Patient 10	Patient 11	Patient 12	Gene Description
PVX_081420	0	0	0	948.174	0	0	0	0	0	0	410.638	0	centrin-1, putative (CEN1)
PVX_081425	0	0	0	0	0	0	0	0	0	0	0	0	hypothetical protein, conserved
PVX_081430	0	0	0	0	0	0	0	0	0	0	40.3322	0	ras-related protein Rab-5C, putative (RAB5c)
PVX_081435	25.2538	0	0	161.318	0	0	38.5863	70.1258	0	17.7487	20.1062	0	ribosome biogenesis protein BMS1, putative
PVX_081440	0	0	0	0	0	0	0	0	0	0	0	0	hypothetical protein, conserved
PVX_081445	0	0	0	0	0	0	0	0	0	0	0	0	hypothetical protein, conserved
PVX_081450	0	0	0	0	0	0	198.906	0	98.3714	45.5696	51.6568	0	pre-rRNA-processing protein TSR2, putative
PVX_081455	0	36.8732	0	84.3758	8.37434	0	0	36.6779	20.0145	27.8488	78.8704	0	calcium-transporting ATPase, putative
PVX_081460	0	0	0	0	0	0	309.181	0	0	0	0	0	hypothetical protein, conserved
PVX_081465	0	0	0	0	0	0	0	207.926	0	0	14.8977	0	vacuolar ATP synthase subunit c, putative
PVX_081470	0	33.739	120.169	308.918	0	0	0	67.116	0	0	19.2395	0	hypothetical protein, conserved
PVX_081475	72.012	150.863	179.176	0	68.562	0	0	0	136.442	25.2995	100.34	116.668	DNA binding protein, putative
PVX_081480	38.4297	26.8248	0	0	0	0	0	0	0	0	61.1938	0	hypothetical protein, conserved
PVX_081485	12.3358	0	30.6419	39.3854	0	0	18.8406	34.2502	18.6906	13.0055	27.0083	0	hypothetical protein
PVX_081490	49.6667	34.6624	0	0	7.8721	0	0	103.437	47.0367	8.7266	34.6	40.1797	hypothetical protein, conserved
PVX_081495	0	0	0	321.208	0	0	0	0	76.0731	141.01	119.862	0	hypothetical protein, conserved
PVX_081498	0	285.603	0	0	0	0	0	0	0	0	81.0943	0	hypothetical protein, conserved
PVX_081502	0	0	0	0	0	0	482.945	0	0	0	62.3593	0	hypothetical protein, conserved
PVX_081505	57.2297	79.9146	0	0	18.1561	0	87.5326	79.4838	86.7381	100.538	148.09	185.361	hypothetical protein, conserved
PVX_081510	0	0	0	0	120.356	0	0	1573.25	142.935	0	74.955	0	hypothetical protein, conserved
PVX_081515	0	41.3054	0	0	18.769	0	0	0	44.8317	20.7853	11.7756	0	transporter, putative
PVX_081520	0	0	0	0	0	0	0	0	0	0	0	0	transporter, putative
PVX_081525	0	19.2376	0	0	0	0	42.1191	0	10.4419	0	5.48638	0	hypothetical protein, conserved
PVX_081530	67.7079	0	336.893	216.512	21.4858	0	103.594	94.0461	25.6566	47.5757	53.9097	0	hypothetical protein, conserved
PVX_081535	166.073	0	206.671	0	26.36	0	127.109	0	94.4053	87.5133	181.819	0	4-hydroxy-3-methylbut-2-enyl diphosphate reductase, putative
PVX_081540	8.21285	5.73053	20.3981	0	0	0	12.5419	45.6039	18.665	11.5452	14.7119	13.282	ubiquitin carboxyl-terminal hydrolase 1, putative (UBP1)
PVX_081550	0	0	0	0	0	0	0	0	26.3958	0	0	0	StAR-related lipid transfer protein, putative
PVX_081555	0	0	0	0	0	0	0	0	15.6481	0	0	0	hypothetical protein, conserved
PVX_081560	0	0	0	0	0	0	0	0	0	0	0	0	hypothetical protein, conserved
PVX_081565	68.5376	143.578	0	0	21.7495	350.897	104.866	0	51.9422	96.317	122.783	0	hypothetical protein, conserved
PVX_081570	0	0	0	0	0	0	146.299	0	0	0	19.0172	0	actin-related protein (ARP1)
PVX_081572	0	0	0	0	18.6211	0	0	0	88.9575	20.6218	58.4148	0	L-seryl-tRNA(Sec) kinase, putative (PSTK)
PVX_081575	0	0	0	0	7.21135	0	0	0	8.61805	7.99462	4.5282	0	hypothetical protein, conserved
PVX_081580	0	0	0	0	0	0	0	0	0	0	11.0142	0	hypothetical protein, conserved
PVX_081585	20.9451	131.552	52.0397	0	13.2778	0	95.9954	29.0794	47.6052	36.802	66.7027	67.7698	hypothetical protein, conserved
PVX_081590	0	31.9275	0	0	14.5055	0	0	0	0	16.0701	63.7246	74.0423	hypothetical protein, conserved
PVX_081595	237.224	0	196.788	0	50.1996	0	121.029	329.541	29.9661	194.459	188.883	128.136	nucleoside transporter 4 (NT4)
PVX_081600	0	0	0	0	7.09229	0	0	0	0	15.7254	26.7208	0	vacuolar protein sorting-associated protein 51, putative (VPS51)
PVX_081605	0	0	0	0	0	0	0	0	0	0	0	0	vacuolar protein sorting-associated protein VTA1, putative
PVX_081610	85.9375	119.98	106.825	0	40.881	0	131.381	238.676	146.514	166.079	102.633	139.115	aspartate--tRNA ligase, putative
PVX_081615	0	0	0	0	20.8844	0	100.692	0	0	0	52.403	0	hypothetical protein, conserved
PVX_081620	0	0	61.9134	0	0	0	0	0	18.8776	17.5115	19.8375	0	DNA mismatch repair protein PMS1, putative (PMS1)
PVX_081625	13.4005	4.67501	0	21.3889	2.12301	0	10.2315	0	15.2272	16.483	18.6703	0	hypothetical protein, conserved
PVX_081630	20.34	14.1926	0	64.9368	6.4454	0	15.5317	14.1181	26.9654	25.0184	30.3631	32.8959	ubiquitin carboxyl-terminal hydrolase family 2, putative
PVX_081635	31.0659	0	0	99.2385	9.84931	0	0	0	0	21.8328	49.4676	0	hypothetical protein, conserved
PVX_081640	0	0	0	938.825	93.0893	0	0	0	0	204.764	174.266	475.593	hypothetical protein, conserved
PVX_081645	0	0	0	0	470.615	0	0	1020.02	0	0	0	0	defender against cell death 2, putative
PVX_081650	0	163.568	0	0	59.4514	0	0	195.23	106.527	49.3964	111.931	0	mitochondrial import inner membrane translocase subunit TIM50, putative (TIM50)
PVX_081655	31.885	0	79.2449	0	0	0	0	44.2726	12.0792	11.2042	25.3859	0	vacuolar protein sorting-associated protein 53, putative (VPS53)
PVX_081660	35.887	25.0491	0	114.655	11.3791	0	0	0	0	37.8306	64.2878	0	hypothetical protein, conserved
PVX_081665	0	0	0	0	0	0	0	0	0	0	0	0	cysteine desulfurase, putative
PVX_081670	40.2986	0	0	0	12.7794	0	0	0	0	14.1601	16.0425	0	DNA (cytosine-5)-methyltransferase, putative (DNMT)
PVX_081675	90.511	126.442	225.322	0	57.4759	0	0	0	137.211	0	180.17	146.715	proteasome subunit alpha type-5, putative
PVX_081680	0	0	0	146.16	0	0	0	0	0	16.0701	0	0	hypothetical protein, conserved
PVX_081685	31.693	0	78.7674	0	0	0	0	0	12.0065	11.1367	25.2331	51.2883	hypothetical protein, conserved
PVX_081690	0	0	75.3233	0	9.60897	0	46.3169	0	22.9637	21.3006	24.1307	0	hypothetical protein, conserved
PVX_081695	37.0285	0	0	59.122	23.4722	0	28.2826	25.7038	42.0795	26.0249	47.9059	0	cation transporting ATPase, putative
PVX_081700	0	0	39.4974	0	0	0	0	0	18.0678	5.58737	15.8229	0	hypothetical protein, conserved
PVX_081705	160.561	280.339	199.796	256.807	50.9667	0	0	0	91.2702	112.813	159.803	0	eukaryotic translation initiation factor 2 alpha subunit, putative
PVX_081707	6.07775	0	0	0	0	0	0	0	0	0	2.41939	0	conserved Plasmodium protein, unknown function
PVX_081710	0	0	0	0	0	0	0	0	0	0	0	0	actin-like protein, putative (ALP3)
PVX_081715	0	0	0	0	54.7563	441.751	0	0	0	0	137.329	139.771	hypothetical protein, conserved

Gene ID / Patient	Patient 1	Patient 2	Patient 3	Patient 4	Patient 5	Patient 6	Patient 7	Patient 8	Patient 9	Patient 10	Patient 11	Patient 12	Gene Description
PVX_081720	0	0	0	0	0	0	0	0	0	106.544	181.363	0	hypothetical protein, conserved
PVX_081722	0	0	0	0	0	0	0	0	0	0	0	0	conserved Plasmodium protein, unknown function
PVX_081725	17.2509	0	42.8566	0	16.4024	0	26.3516	23.9496	52.277	24.2492	27.4688	27.9055	hypothetical protein, conserved
PVX_081730	0	0	0	0	0	0	0	0	0	19.9926	0	0	alpha/beta hydrolase, putative
PVX_081740	0	0	0	0	0	0	0	105.913	28.8933	53.5723	45.5309	123.541	hypothetical protein, conserved
PVX_081745	107.839	75.2892	0	0	17.1045	0	82.4611	0	20.4298	132.614	64.3958	174.623	RNA-binding protein, putative
PVX_081750	0	328.095	0	0	74.6954	0	0	0	355.73	82.2917	140.019	0	signal recognition particle subunit SRP9, putative (SRP9)
PVX_081755	0	0	0	0	3.60502	0	0	0	0	0	4.52856	0	hypothetical protein, conserved
PVX_081760	0	0	0	0	0	0	0	0	199.332	61.5259	69.7646	284.699	peroxiredoxin, putative
PVX_081765	36.1479	75.6939	0	115.489	0	0	55.2509	0	68.4728	63.5094	93.5353	0	60S ribosomal export protein NMD3, putative (NMD3)
PVX_081770	112.446	196.27	139.829	0	35.6725	0	0	0	149.12	98.77	33.5735	0	hypothetical protein, conserved
PVX_081775	39.8678	27.8291	99.107	0	0	203.939	60.9438	0	0	28.0174	23.8064	0	hypothetical protein, conserved
PVX_081780	0	127.007	0	0	0	0	278.888	0	0	63.7713	108.471	0	hypothetical protein, conserved
PVX_081785	43.2804	30.2126	0	0	0	0	0	60.102	32.795	30.4152	51.6888	0	hypothetical protein, conserved
PVX_081790	0	0	0	0	0	0	0	0	0	0	0	0	hypothetical protein, conserved
PVX_081792	0	0	0	0	0	0	0	8.3417	4.5522	0	2.39209	0	hypothetical protein
PVX_081795	0	0	0	0	0	0	0	0	0	0	0	0	hypothetical protein, conserved
PVX_081800	35.7579	0	0	0	0	0	0	49.6521	13.5468	12.5649	0	0	hypothetical protein, conserved
PVX_081805	0	0	0	0	11.3246	0	54.5887	49.5926	0	25.0997	35.5444	57.8037	AP-4 complex subunit beta, putative
PVX_081810	119.876	50.1963	0	76.5721	37.9995	0	0	66.5743	127.151	109.526	109.757	38.7902	transcription factor with AP2 domain(s), putative (AP2-L)
PVX_081815	0	0	0	0	0	0	0	0	0	0	0	0	hypothetical protein, conserved
PVX_081820	9.77349	0	0	0	6.19405	49.9501	0	13.5676	7.40399	3.43472	7.78115	0	hypothetical protein, conserved
PVX_081822	0	0	0	0	0	0	0	0	0	0	0	0	tRNA Valine
PVX_081824	0	0	0	0	0	0	0	0	0	0	0	0	tRNA Threonine
PVX_081825	0	0	0	0	0	0	0	0	0	0	0	0	hypothetical protein
PVX_081830	507.822	1447.5	526.033	270.454	348.935	1515.44	388.172	235.063	432.89	565.048	480.129	274.015	Plasmodium exported protein, unknown function
PVX_081832	0	121.919	0	0	0	178.676	0	0	13.2347	98.2043	104.302	0	Plasmodium exported protein, unknown function
PVX_081835	44.7745	0	0	0	0	0	0	0	0	0	0	0	knob-associated histidine-rich protein, putative
PVX_081840	84.4471	0	0	0	0	0	0	0	128.013	88.999	67.2391	0	heat shock protein, putative,DnaJ domain containing protein
PVX_081845	79.0747	55.2248	0	0	0	0	0	0	0	27.7798	31.4805	0	Plasmodium exported protein, unknown function
PVX_081847	412.527	72.0483	0	330.118	32.7553	528.561	157.971	143.3	234.536	72.4538	307.944	334.464	Plasmodium exported protein, unknown function
PVX_081850	0	0	0	0	0	0	0	0	0	0	0	0	variable surface protein Vir22/23-related,PIR protein
PVX_082350	0	9.40522	0	0	4.27143	0	0	0	0	0	26.8272	0	hypothetical protein, conserved
PVX_082355	492.734	57.3555	408.78	0	78.2073	0	125.705	456.335	248.974	317.35	474.062	133.086	proteasome subunit beta type-7, putative
PVX_082360	0	0	0	0	0	0	0	0	8.8743	0	13.9894	0	hypothetical protein, conserved
PVX_082365	0	0	0	313.798	0	0	0	0	74.3217	0	58.552	0	hypothetical protein, conserved
PVX_082370	241.709	0	0	0	0	0	0	111.925	30.533	56.6096	16.0379	0	hypothetical protein, conserved
PVX_082375	0	0	79.2732	0	0	0	0	44.2884	12.0835	11.2082	50.79	0	PST-A protein
PVX_082380	0	0	0	0	0	0	0	0	0	0	0	0	hypothetical protein, conserved
PVX_082385	124.685	87.1295	0	399.349	39.6216	0	0	0	47.2653	0	49.6413	0	NAD-dependent deacetylase, putative
PVX_082390	274.307	63.8678	0	0	87.0967	0	0	127.034	69.3068	64.2407	182.011	0	hypothetical protein, conserved
PVX_082395	15.8739	33.2315	0	0	15.0927	0	24.2472	22.0377	18.0389	5.57845	18.9572	0	DNA-directed RNA polymerase III largest subunit, putative
PVX_082400	0	0	0	0	19.7865	0	0	21.6686	23.6492	21.9402	12.4265	0	myosin C, putative
PVX_082405	0	0	0	0	0	0	0	0	0	0	0	0	hypothetical protein, conserved
PVX_082410	63.9807	0	0	0	40.6023	0	0	88.8657	48.4872	22.4788	63.6772	0	chromatin assembly factor 1 P55 subunit, putative
PVX_082415	0	0	0	0	0	0	0	0	0	0	0	0	adenosine/AMP deaminase, putative
PVX_082420	12.2901	17.1517	0	0	3.89472	0	18.7708	34.1232	23.2766	4.31909	14.6772	0	hypothetical protein, conserved
PVX_082425	0	101.31	180.486	231.987	23.021	0	0	100.759	27.4875	76.4519	101.072	0	hypothetical protein
PVX_082430	0	0	0	0	0	0	0	0	0	0	0	0	apicoplast calcium binding protein 1, putative (ACBP1)
PVX_082435	0	0	0	0	0	0	43.6253	0	10.8151	0	0	0	hypothetical protein, conserved
PVX_082437	0	0	0	155.468	0	0	0	0	0	0	0	0	conserved Plasmodium protein, unknown function
PVX_082440	128.259	0	0	0	0	0	0	356.514	0	90.095	0	0	hypothetical protein, conserved
PVX_082445	187.32	0	0	601.022	0	0	575.394	260.492	71.0395	65.7716	111.877	0	hypothetical protein, conserved
PVX_082450	163.68	114.437	0	0	104.126	0	753.6	227.566	186.193	57.4782	228.095	0	hypothetical protein, conserved
PVX_082455	318.619	0	0	0	50.6676	0	0	0	0	0	31.7148	0	DnaJ domain containing protein
PVX_082460	0	0	0	0	0	0	0	0	52.0561	0	0	0	ER lumen protein retaining receptor 1, putative
PVX_082465	88.4866	0	0	282.77	14.0317	0	67.6419	61.4396	50.2873	31.0917	132.097	143.247	hypothetical protein, conserved
PVX_082470	229.911	0	190.709	0	48.6491	0	117.288	106.458	145.209	53.8474	198.314	124.177	elongation factor Tu, mitochondrial precursor, putative
PVX_082475	35.5308	24.8004	88.315	0	22.5322	0	54.3066	0	26.9214	37.4552	28.2888	0	hypothetical protein, conserved
PVX_082480	50.0311	69.856	124.407	159.907	31.739	256.006	153.009	0	113.736	87.895	109.544	0	hypothetical protein, conserved
PVX_082485	0	0	0	0	0	0	0	0	0	0	13.0432	0	hypothetical protein, conserved
PVX_082490	0	0	0	0	0	0	0	0	10.1057	0	15.9292	0	helicase, putative

Gene ID / Patient	Patient 1	Patient 2	Patient 3	Patient 4	Patient 5	Patient 6	Patient 7	Patient 8	Patient 9	Patient 10	Patient 11	Patient 12	Gene Description
PVX_082495	93.7342	0	0	0	0	0	0	0	142.1	98.7822	55.976	0	hypothetical protein, conserved
PVX_082500	254.418	0	0	0	0	0	0	0	96.5304	0	101.31	0	signal peptidase 21 kDa subunit, putative
PVX_082505	0	0	0	0	0	0	0	0	0	0	0	0	CPW-WPC family protein, putative
PVX_082510	0	0	0	0	0	0	78.2127	71.0304	0	17.9707	20.3611	0	hypothetical protein
PVX_082515	0	106.439	0	0	0	0	0	0	57.7304	160.415	121.246	0	protein tyrosine phosphatase-like protein, putative (PTPLA)
PVX_082520	262.581	26.1836	93.2435	359.551	47.579	0	57.3377	104.176	227.381	276.8	104.531	60.7142	glutamine--tRNA ligase, putative
PVX_082525	1835.7	257.361	0	1183.99	352.1	0	567.14	511.461	1115.26	901.56	438.655	599.792	60S ribosomal protein L23, putative
PVX_082530	139.234	0	0	0	0	0	0	0	52.7857	97.7989	83.1522	0	syntaxin 5, putative
PVX_082535	0	0	0	0	0	0	0	0	0	0	0	0	hypothetical protein, conserved
PVX_082540	0	0	0	0	0	0	0	0	0	0	0	0	hypothetical protein, conserved
PVX_082545	0	0	0	0	0	0	0	394.511	107.557	0	112.865	0	trafficking protein particle complex subunit 2, putative (TRAPPC2)
PVX_082550	40.2442	56.1841	100.044	128.591	12.7621	0	123.04	55.8841	106.728	42.4229	72.0938	195.427	hypothetical protein, conserved
PVX_082555	184.996	129.376	0	0	58.8743	0	0	0	140.315	64.9566	110.489	0	SAM-dependent RNA methyltransferase, putative
PVX_082560	70.1493	48.9857	0	448.669	0	0	107.337	194.879	79.7464	24.6453	55.8537	0	apurinic/apyrimidinic endonuclease Apn1, putative
PVX_082565	0	49.3341	0	0	22.4202	0	0	0	0	24.8203	28.1253	0	DEAD/DEAH box helicase domain containing protein
PVX_082570	0	231.419	0	0	0	0	508.004	0	188.261	58.1154	164.733	268.831	eukaryotic translation initiation factor 6, putative
PVX_082575	52.3697	18.2747	65.0677	0	24.9024	0	0	0	69.4358	36.8058	78.178	84.7359	isoleucine--tRNA ligase, putative
PVX_082580	84.5268	118.073	0	0	0	0	129.396	0	128.134	29.6944	0	0	20 kDa chaperonin, putative (CPN20)
PVX_082585	0	0	0	0	0	0	0	0	0	0	0	0	hypothetical protein, conserved
PVX_082590	0	0	0	0	0	0	0	0	14.2836	13.2509	3.75261	0	ubiquitin-activating enzyme, putative
PVX_082595	0	0	505.919	650.281	0	0	0	0	76.8367	0	161.329	0	hypothetical protein, conserved
PVX_082600	176.252	369.74	0	0	0	0	270.603	0	200.511	185.667	105.265	0	hypothetical protein, conserved
PVX_082605	0	0	272.918	0	0	0	0	152.257	83.0639	0	43.6234	0	hypothetical protein, conserved
PVX_082610	53.8346	37.5722	66.8892	0	8.53313	0	0	37.3731	40.7877	9.4588	64.2919	0	hypothetical protein, conserved
PVX_082615	0	0	0	0	0	0	0	0	0	0	0	0	histone H3-like centromeric protein CSE4, putative (CenH3)
PVX_082620	0	88.6133	0	0	0	0	0	176.234	48.0697	0	0	0	hypothetical protein, conserved
PVX_082625	25.084	0	0	0	15.9032	0	0	34.827	0	17.6293	29.9564	40.5854	hypothetical protein, conserved
PVX_082630	0	0	0	0	0	0	0	0	0	26.9237	0	0	hypothetical protein, conserved
PVX_082635	63.3188	0	0	0	10.0376	0	0	43.9593	0	33.3748	25.2063	0	hypothetical protein, conserved
PVX_082640	47.4073	0	0	0	45.1088	0	72.4865	197.506	233.5	99.9439	56.6177	76.7523	chaperone binding protein, putative
PVX_082645	0	134.331	0	0	0	492.681	0	133.591	109.325	101.328	191.398	311.764	merozoite surface protein 7 (MSP7), putative
PVX_082650	0	0	0	0	0	0	0	0	0	0	0	0	merozoite surface protein 7 (MSP7), putative
PVX_082655	0	0	0	0	0	0	0	0	0	0	0	0	merozoite surface protein 7 (MSP7), putative
PVX_082660	0	0	0	0	174.169	0	0	0	0	0	108.731	0	hypothetical protein
PVX_082665	0	0	0	0	0	0	0	0	0	0	0	0	merozoite surface protein 7 (MSP7)
PVX_082670	0	0	0	0	0	0	0	0	0	0	51.7478	0	merozoite surface protein 7 (MSP7), putative
PVX_082675	0	0	0	0	0	0	0	0	0	0	0	0	merozoite surface protein 7 (MSP7)
PVX_082680	0	0	0	0	0	0	0	0	0	0	0	0	merozoite surface protein 7 (MSP7), putative
PVX_082685	0	0	0	0	0	0	0	0	0	0	0	0	merozoite surface protein 7 (MSP7)
PVX_082690	0	0	0	0	0	0	0	0	0	0	0	0	merozoite surface protein 7 (MSP7)
PVX_082695	0	0	0	0	0	0	0	0	0	0	0	0	merozoite surface protein 7 (MSP7), putative
PVX_082700	84.4471	0	0	0	0	0	0	0	0	0	0	0	merozoite surface protein 7 (MSP7)
PVX_082710	186.298	130.132	0	0	0	0	0	0	70.6061	130.887	0	0	hypothetical protein
PVX_082715	0	0	0	0	0	0	0	0	0	0	0	0	hypothetical protein, conserved
PVX_082720	0	117.297	209.004	0	0	0	0	116.655	0	58.9988	117.006	0	hypothetical protein, conserved
PVX_082725	42.8223	0	53.1983	0	0	0	32.7109	0	16.2215	15.0483	42.6168	0	hypothetical protein, conserved
PVX_082730	0	34.5225	122.962	158.049	15.6851	0	0	0	37.4719	0	19.686	0	hypothetical protein, conserved
PVX_082735	0	0	0	0	0	0	0	0	0	0	0	0	thrombospondin-related anonymous protein,sporozoite surface protein 2 (TRAP)
PVX_082740	0	53.1109	0	0	24.1382	0	0	0	0	0	0	0	hypothetical protein, conserved
PVX_082742	0	0	0	1030.25	102.144	0	0	0	0	0	63.6973	0	conserved Plasmodium protein, unknown function
PVX_082745	0	81.8528	0	374.786	18.5967	0	89.6579	81.4111	88.841	102.974	140.012	0	hypothetical protein
PVX_082750	0	66.9495	0	0	30.4346	0	0	0	0	33.6676	19.0783	0	hypothetical protein, conserved
PVX_082755	143.332	50.0459	0	0	0	0	0	0	54.3143	151.068	156.919	0	RanBPM and CLTH-like protein, putative
PVX_082760	0	0	0	0	79.9621	0	0	0	190.35	0	99.8896	0	hypothetical protein, conserved
PVX_082765	0	154.982	184.074	0	0	0	0	0	56.0662	155.936	14.7253	0	hypothetical protein
PVX_082770	0	148.589	0	0	22.5092	0	0	197.043	161.263	199.349	211.776	0	nuclear movement protein, putative
PVX_082775	0	0	128.286	0	16.3641	0	0	0	19.5463	108.756	71.8801	0	tryptophan--tRNA ligase, putative
PVX_082780	32.3811	0	0	0	0	0	0	0	12.2672	45.5139	6.44523	0	mitochondrial intermediate peptidase, putative
PVX_082785	125.208	0	0	0	0	0	0	0	47.4639	175.907	49.8497	0	CDK-related protein kinase 6, putative
PVX_082790	0	19.9379	70.992	91.2493	0	0	0	79.3285	21.644	50.1921	39.8022	0	1-deoxy-D-xylulose 5-phosphate synthase, putative (DXS)
PVX_082795	28.0214	58.6708	0	0	17.7668	0	128.456	0	84.922	68.9271	55.7743	90.6836	exonuclease, putative
PVX_082800	0	36.6502	0	0	0	0	0	0	19.8904	36.8899	52.2465	0	hypothetical protein, conserved

Gene ID / Patient	Patient 1	Patient 2	Patient 3	Patient 4	Patient 5	Patient 6	Patient 7	Patient 8	Patient 9	Patient 10	Patient 11	Patient 12	Gene Description
PVX_082810	14.4094	0	0	0	4.56658	0	0	0	10.9163	0	8.60409	0	hypothetical protein
PVX_082813	0	0	0	0	0	0	0	0	0	0	0	0	tRNA Serine
PVX_082815	0	0	0	0	0	0	0	0	0	0	0	0	hypothetical protein, conserved
PVX_082820	60.1128	0	0	0	0	0	0	0	0	0	0	0	calcium-dependent protein kinase, putative
PVX_082825	0	0	0	0	49.6038	0	0	0	0	54.7755	31.0514	0	hypothetical protein, conserved
PVX_082830	0	44.8672	159.844	0	0	0	0	0	0	0	38.3709	0	hypothetical protein, conserved
PVX_082835	66.8481	0	0	0	42.425	0	0	92.8511	126.653	117.429	172.981	324.859	26S proteasome regulatory subunit RPN3, putative (RPN3)
PVX_082840	961.316	1007.59	1197.57	0	381.809	1232.34	1289.12	834.971	774.356	1181.78	574.096	0	60S ribosomal protein L6, putative
PVX_082845	544.444	266.079	406.196	348.068	138.17	557.252	166.53	226.84	453.833	439.973	422.647	0	elongation factor 1-gamma, putative
PVX_082850	0	0	0	0	0	0	0	0	0	0	0	0	hypothetical protein, conserved
PVX_082855	91.6116	0	0	0	14.5278	0	70.0343	0	52.0638	16.0947	100.292	74.1562	hypothetical protein, conserved
PVX_082860	37.7489	0	281.502	0	0	0	0	104.835	28.6025	39.793	15.0274	61.0988	hypothetical protein, conserved
PVX_082865	125.238	0	103.782	0	13.2389	0	0	0	0	44.0057	33.2375	0	hypothetical protein, conserved
PVX_082870	0	0	0	0	0	0	0	0	0	0	0	0	hypothetical protein
PVX_082875	0	0	0	0	0	0	0	0	0	0	0	0	CPW-WPC family protein, putative
PVX_082880	35.9302	25.0793	0	0	0	0	0	0	13.6121	0	42.9102	0	protein kinase, putative
PVX_082882	0	0	0	0	0	0	0	0	0	0	0	0	tRNA Methionine
PVX_082884	0	0	0	0	0	0	0	0	0	0	0	0	tRNA Proline
PVX_082885	0	0	0	0	0	0	0	0	0	94.5644	107.2	0	hypothetical protein, conserved
PVX_082890	341.894	0	0	1101.79	0	0	0	0	389.396	119.953	340.397	0	hypothetical protein, conserved
PVX_082895	0	0	0	0	93.3959	0	0	0	111.082	205.433	0	0	60S ribosomal protein, mitochondrial precursor, putative
PVX_082900	0	0	0	0	0	0	0	89.0568	48.5914	0	63.8141	103.856	hypothetical protein, conserved
PVX_082905	18.5488	0	0	0	0	0	0	0	0	13.0367	11.0758	0	hypothetical protein, conserved
PVX_082910	0	91.069	0	0	41.4158	0	0	0	0	0	51.8825	0	mitotic-spindle organizing protein 1, putative
PVX_082915	0	0	0	0	0	0	0	0	0	28.0174	31.7419	0	ABC transporter, putative
PVX_082920	0	0	0	0	0	0	0	0	0	0	0	0	hypothetical protein, conserved
PVX_082925	139.234	194.629	0	0	88.5213	0	0	0	52.7857	48.8994	166.304	0	exosome complex component RRP42, putative (RRP42)
PVX_082930	0	0	0	0	0	0	0	0	0	0	0	0	hypothetical protein
PVX_082935	27.2534	152.166	0	0	43.1987	0	0	0	72.2698	38.3075	54.2456	0	nucleolar complex protein 2, putative
PVX_082937	0	0	0	0	0	0	0	138.971	37.9089	0	0	0	hypothetical protein, conserved
PVX_082938	0	0	0	0	0	0	0	0	10.142	0	0	0	hypothetical protein, conserved
PVX_082945	0	83.4388	0	0	0	0	0	331.953	45.2809	41.9868	107.042	96.7713	Ser/Thr protein phosphatase family protein
PVX_082950	0	0	0	0	0	0	0	0	0	0	0	0	ras-related protein Rab-11B, putative (RAB11b)
PVX_082955	0	0	0	0	0	0	0	0	0	0	152.346	0	mitochondrial pyruvate carrier protein 1, putative (MPC1)
PVX_082960	0	0	0	0	16.9208	272.971	0	0	20.2106	56.2251	53.0874	0	sodium-dependent phosphate transporter, putative (PiT)
PVX_082962	0	0	0	0	0	0	0	0	0	0	0	0	tRNA Arginine
PVX_082964	0	0	0	0	0	0	0	0	0	0	0	0	U6 spliceosomal RNA
PVX_082965	240.007	167.706	0	384.305	190.648	1230.68	367.825	166.77	363.914	505.797	262.775	389.365	60S ribosomal protein L18a, putative
PVX_082970	405.059	212.227	0	0	0	0	0	281.405	575.716	462.428	423.315	164.196	60S ribosomal protein L18, putative
PVX_082975	0	0	0	0	0	0	0	0	0	0	0	0	hypothetical protein, conserved
PVX_082980	0	60.867	0	0	0	0	0	0	16.5174	15.3187	8.67775	0	GPI mannosyltransferase 3, putative (GPI10)
PVX_082985	0	0	0	0	0	0	0	0	242.857	0	0	0	hypothetical protein, conserved
PVX_082990	0	0	0	0	0	0	0	0	0	0	15.8242	0	hypothetical protein, conserved
PVX_082995	0	0	0	0	0	845.065	0	0	62.4071	0	0	0	vacuolar ATP synthase subunit d, putative
PVX_083000	1685.06	1250.8	786.864	337.131	234.157	1619.38	322.656	731.691	997.947	1035.83	985.333	853.926	40S ribosomal protein S6, putative
PVX_083005	0	0	0	226.851	0	0	54.2635	98.5949	13.45	0	56.5326	0	aconitate hydratase I, putative,IRP-like protein (iron regulatory protein-like), putative
PVX_083010	104.844	24.3934	0	0	0	0	0	0	39.7197	0	6.95619	0	hypothetical protein, conserved
PVX_083015	86.4874	0	0	0	0	443.035	0	0	32.7769	121.531	68.8638	0	hsp70 interacting protein, putative
PVX_083020	94.0297	131.364	0	0	0	0	0	0	71.2742	33.0311	37.435	152.434	casein kinase II beta chain, putative
PVX_083025	0	0	0	0	0	0	0	0	0	0	0	0	sporozoite protein essential for cell traversal, putative (SPECT1)
PVX_083030	168.236	23.4851	83.6288	107.492	21.3367	0	51.4246	233.601	50.9879	35.47	80.3671	54.4537	myosin A, putative
PVX_083032	0	0	0	0	0	0	0	0	1864.57	0	0	0	transcription activator, putative
PVX_083035	56.047	0	0	0	0	286.841	0	0	42.4724	19.6922	89.2489	0	phosphoenolpyruvate carboxykinase, putative (PEPCK)
PVX_083040	15.7743	5.50324	0	0	2.49916	0	12.0444	0	11.9498	19.4028	17.2681	25.5102	transcription factor with AP2 domain(s), putative (ApiAP2)
PVX_083045	1556.77	483.26	1291.68	830.13	686.442	0	397.216	1081.38	753.869	638.038	2496.31	981.238	phosphoethanolamine N-methyltransferase, putative (PMT)
PVX_083050	0	0	0	0	26.5078	0	0	0	31.6447	29.3343	33.2431	0	hypothetical protein, conserved
PVX_083055	138.347	0	172.099	0	21.9516	0	0	0	26.2121	24.3025	68.8457	112.06	hypothetical protein, conserved
PVX_083060	0	0	88.1399	113.29	33.7313	0	54.1989	0	40.3021	12.4603	28.2327	0	hypothetical protein, conserved
PVX_083065	18.1317	0	0	0	0	0	0	0	13.7366	19.1154	14.4357	0	DNA repair protein RAD5, putative (RAD5)
PVX_083070	0	59.6571	0	273.269	0	0	0	0	161.85	0	119.016	0	hypothetical protein, conserved
PVX_083075	0	0	0	0	0	0	0	0	24.0885	0	63.2701	205.938	UDP-N-acteylglucosamine pyrophosphorylase 1, putative
PVX_083080	46.2317	32.274	0	147.748	0	0	0	0	0	16.2444	18.4046	0	kelch protein K13, putative

Gene ID / Patient	Patient 1	Patient 2	Patient 3	Patient 4	Patient 5	Patient 6	Patient 7	Patient 8	Patient 9	Patient 10	Patient 11	Patient 12	Gene Description
PVX_083085	0	0	0	0	0	0	0	0	0	0	1.65199	0	hypothetical protein, conserved
PVX_083090	0	0	0	0	0	0	79.027	0	0	36.3149	0	0	U4/U6 small nuclear ribonucleoprotein PRP4, putative
PVX_083095	29.5187	0	0	0	9.3584	0	45.1088	0	22.3652	51.864	23.5019	0	aminomethyl transferase, putative
PVX_083100	0	43.5772	0	0	9.89745	0	47.7077	0	0	0	12.4273	0	hypothetical protein, conserved
PVX_083105	34.6369	48.3524	86.0911	110.657	32.9473	0	52.9389	96.19	118.098	97.3683	62.0484	56.057	heat shock protein 110, putative (HSP110)
PVX_083110	0	0	43.081	55.374	0	0	0	24.0749	19.7064	12.188	13.8063	56.1031	hypothetical protein, conserved
PVX_083115	47.2321	0	0	0	0	0	0	0	35.7903	33.1916	56.4085	0	hypothetical protein, conserved
PVX_083120	226.349	0	281.92	0	0	0	0	0	0	39.7525	157.703	0	hypothetical protein, conserved
PVX_083125	0	0	0	0	0	0	0	0	0	22.0997	25.0412	0	lipoyl synthase, putative
PVX_083130	27.2368	19.0091	0	0	0	0	41.6188	0	20.6359	38.2842	75.8976	0	hypothetical protein, conserved
PVX_083135	0	46.0928	0	0	0	337.931	0	0	75.0386	23.1915	0	0	aspartate carbamoyltransferase, putative
PVX_083140	17.0895	59.6278	0	0	10.8326	0	0	0	19.4204	24.0223	17.0074	0	hypothetical protein, conserved
PVX_083150	0	0	0	0	0	0	0	196.264	0	0	14.0626	0	hypothetical protein, conserved
PVX_083155	0	0	341.752	0	0	703.371	0	0	103.953	48.1508	0	222.527	thioredoxin 2, putative (TRX2)
PVX_083160	0	42.4859	0	0	0	0	0	0	0	0	12.1119	0	rhomboid protease ROM6, putative
PVX_083165	220.559	154.317	0	0	70.2533	0	0	306.816	167.329	0	43.9116	358.831	hypothetical protein
PVX_083170	0	0	0	0	11.1763	0	0	0	0	6.19606	7.01874	0	hypothetical protein, conserved
PVX_083175	152.782	0	0	489.73	0	0	0	0	0	53.6542	152.076	248.089	ubiquitin-conjugating enzyme E2, putative
PVX_083180	0	0	0	0	0	0	0	0	0	0	0	0	hypothetical protein, conserved
PVX_083185	0	0	184.532	0	47.074	0	113.489	412.06	112.411	26.0539	280.476	120.156	isocitrate dehydrogenase [NADP], mitochondrial precursor, putative
PVX_083190	176.124	40.9748	0	93.7655	37.2247	0	0	40.7574	155.683	82.5197	122.697	95.0001	hypothetical protein, conserved
PVX_083195	0	0	0	0	0	0	0	0	75.8057	0	119.375	0	hypothetical protein, conserved
PVX_083200	0	0	0	0	0	0	0	0	0	0	0	0	dynactin subunit 2, putative
PVX_083205	294.445	308.433	183.164	706.286	46.7249	0	0	102.252	306.841	129.306	161.179	0	protein transport protein SEC61 subunit alpha, putative
PVX_083210	0	0	0	0	0	0	0	0	111.815	206.783	58.6626	0	nuclear import protein MOG1, putative
PVX_083215	212.696	892.801	0	0	135.471	0	0	0	161.355	149.344	42.3457	0	DNA/RNA-binding protein Alba 2, putative (ALBA2)
PVX_083220	27.1872	7.58768	0	0	3.44566	0	0	0	18.5358	11.4656	17.3159	17.5856	hypothetical protein, conserved
PVX_083225	85.4452	0	0	136.52	40.6466	0	0	0	32.3722	60.0465	85.0375	0	hypothetical protein, conserved
PVX_083230	193.392	0	0	0	61.5587	0	0	0	146.69	67.9015	77.0029	0	hypothetical protein, conserved
PVX_083235	0	0	0	0	0	0	0	0	19.1704	0	0	0	6-cysteine protein
PVX_083240	0	39.1555	0	0	17.7915	0	0	77.889	0	0	11.1631	0	6-cysteine protein
PVX_083245	227.28	0	0	0	0	0	0	0	86.218	79.7867	45.2502	0	hypothetical protein, conserved
PVX_083250	0	0	0	0	31.9794	0	0	0	0	0	0	0	G-beta repeat protein, putative
PVX_083255	41.8046	0	103.927	0	0	0	0	0	15.8382	0	8.32101	0	DNA topoisomerase 3, putative (TOP3)
PVX_083260	0	41.4425	0	0	56.4941	0	0	164.875	157.432	41.7084	35.4441	0	nucleoside transporter 1, putative (NT1)
PVX_083262	0	0	0	0	0	0	0	0	0	0	0	0	conserved Plasmodium membrane protein, unknown function
PVX_083265	319.896	0	0	0	0	0	0	0	0	112.247	0	0	hypothetical protein, conserved
PVX_083270	909.714	882.082	502.696	484.604	272.527	258.612	463.702	1193.19	497.867	1047.7	523.11	245.492	DNA/RNA-binding protein Alba 4, putative (ALBA4)
PVX_083275	0	0	0	0	0	0	0	0	0	0	25.9413	0	hypothetical protein
PVX_083280	141.323	98.658	234.258	0	0	0	216.084	65.4194	35.6961	49.6564	75.0134	0	ethanolamine-phosphate cytidylyltransferase, putative
PVX_083285	0	85.6945	0	0	0	0	0	0	46.4874	43.0734	0	0	leucine-rich repeat protein
PVX_083290	53.2302	12.382	0	0	11.2473	0	0	0	20.1636	37.4122	45.9113	0	hypothetical protein, conserved
PVX_083295	0	0	0	0	0	0	0	0	0	0	0	0	hypothetical protein, conserved
PVX_083300	0	0	0	0	0	0	0	0	0	0	0	0	GTPase, putative
PVX_083305	0	0	0	0	0	0	0	0	77.6752	0	54.4036	0	step II splicing factor, putative
PVX_083310	45.1217	0	0	0	0	0	0	31.3231	17.0927	15.8562	17.9621	36.5005	translation elongation factor, putative
PVX_083315	0	5.98485	0	0	5.43579	0	0	0	3.24888	3.01436	5.1216	27.743	hypothetical protein, conserved
PVX_083320	318.196	0	0	0	0	0	0	0	80.4039	74.5136	126.682	0	TBC domain protein, putative
PVX_083325	0	0	0	0	0	0	0	0	0	33.5598	19.0172	0	hypothetical protein, conserved
PVX_083330	0	0	0	0	12.3507	0	0	0	0	13.6857	23.2573	0	hypothetical protein, conserved
PVX_083335	0	0	0	0	0	0	0	0	7.05264	0	3.70577	0	plasma membrane Ca2+ ATPase, putative
PVX_083340	0	94.6588	0	0	43.0509	0	207.673	0	51.3464	95.1365	53.9244	0	hypothetical protein, conserved
PVX_083345	0	0	0	0	0	0	0	88.0469	0	0	0	0	hypothetical protein, conserved
PVX_083350	875.76	306.362	0	703.104	69.7349	0	673.204	913.677	747.444	538.034	523.071	356.181	nucleoside diphosphate hydrolase, putative
PVX_083355	0	57.8231	102.964	132.345	13.1346	0	63.3162	57.5141	62.7662	29.1064	107.172	0	glutamate--tRNA ligase, putative
PVX_083360	0	12.471	88.7957	0	0	0	0	24.8106	0	6.28019	39.1273	28.909	tyrosine kinase-like protein, putative (TKL3)
PVX_083365	0	0	0	0	0	0	0	0	101.768	94.281	80.1586	0	cytidine and deoxycytidylate deaminase, putative
PVX_083370	22.3601	7.80118	0	35.6941	0	0	0	15.5204	16.9392	23.574	33.3787	18.082	hypothetical protein, conserved
PVX_083375	0	0	194.89	0	0	0	0	0	29.6775	27.5125	31.1774	0	hypothetical protein, conserved
PVX_083380	0	0	0	0	0	0	0	165.879	0	0	23.7611	0	hypothetical protein, conserved
PVX_083385	0	59.4866	0	0	27.0387	0	130.384	0	32.2775	0	50.8613	138.037	hypothetical protein, conserved
PVX_083390	0	0	0	0	0	0	0	0	0	0	0	0	hypothetical protein, conserved

Gene ID / Patient	Patient 1	Patient 2	Patient 3	Patient 4	Patient 5	Patient 6	Patient 7	Patient 8	Patient 9	Patient 10	Patient 11	Patient 12	Gene Description
PVX_083395	0	0	0	0	0	0	0	0	0	0	0	0	hypothetical protein, conserved
PVX_083400	159.358	74.1712	0	169.793	16.8504	0	81.2354	73.7722	100.633	55.9916	148.027	0	lysine--tRNA ligase, putative
PVX_083405	0	723.305	0	0	332.293	0	0	0	780.659	0	1019.96	0	small nuclear ribonucleoprotein E, putative
PVX_083410	327.512	305.092	0	0	69.3568	0	167.253	303.399	124.139	38.347	173.855	0	hypothetical protein, conserved
PVX_083415	0	0	0	0	0	0	0	79.9441	21.8101	0	22.915	0	ubiquitin-activating enzyme E1, putative
PVX_083420	0	0	0	0	0	0	0	0	27.8281	0	9.74777	39.6186	hypothetical protein, conserved
PVX_083425	0	110.196	0	0	0	0	0	219.136	59.7662	110.708	125.518	0	hypothetical protein, conserved
PVX_083430	86.9073	0	0	0	0	0	0	0	32.9361	0	34.5991	0	hypothetical protein, conserved
PVX_083435	0	0	229.51	0	0	0	0	0	34.9392	0	91.7554	0	hypothetical protein, conserved
PVX_083440	0	0	0	0	0	0	0	0	0	0	0	0	transcription factor with AP2 domain(s), putative (ApiAP2)
PVX_083445	103.43	24.0644	0	110.145	21.8633	176.334	0	0	26.1226	12.1148	0	0	phosphatidylinositol transfer protein, putative
PVX_083450	0	287.463	0	0	43.5803	0	420.459	0	363.834	288.905	245.634	0	hypothetical protein, conserved
PVX_083455	22.2413	0	0	0	0	0	0	30.8793	8.42525	0	8.85382	0	hypothetical protein, conserved
PVX_083460	0	0	0	0	23.537	0	0	0	28.1028	78.1617	0	0	hypothetical protein, conserved
PVX_083465	490.774	822.804	1710.82	314.143	280.537	0	751.617	545.51	892.839	861.988	586.162	318.279	60S ribosomal protein L17, putative
PVX_083470	0	0	0	0	14.4243	0	0	0	17.2311	0	27.1579	0	glycerol kinase, putative
PVX_083475	0	0	0	0	0	0	0	0	0	0	0	0	inner membrane complex protein 1f, putative (IMC1f)
PVX_083480	70.9839	247.845	353.226	0	45.0543	363.449	0	197.199	295.884	199.507	240.203	229.998	hypothetical protein, conserved
PVX_083485	0	0	0	0	0	0	0	0	26.2527	48.6802	0	0	hypothetical protein, conserved
PVX_083490	17.1912	0	0	54.8951	5.44855	0	0	0	6.51202	0	17.1087	27.809	hypothetical protein, conserved
PVX_083495	0	0	0	0	0	0	0	44.4787	24.2709	11.2563	19.1281	0	ABC transporter, putative
PVX_083500	51.0878	35.6662	0	0	16.2051	0	0	0	58.0695	35.9004	10.1689	0	hypothetical protein, conserved
PVX_083505	0	0	0	0	16.031	0	0	0	57.4462	0	20.1196	0	hypothetical protein, conserved
PVX_083510	0	28.6897	51.071	65.6439	0	0	0	28.5383	15.5731	28.8939	45.0049	0	hypothetical protein, conserved
PVX_083515	147.01	102.76	0	0	46.7416	0	0	0	55.7369	258.143	58.5315	0	protein disulfide-isomerase, putative
PVX_083520	0	0	0	0	26.5824	0	0	0	0	0	16.6682	0	calcium-dependent protein kinase, putative
PVX_083530	12.4943	8.71837	31.0356	0	3.95945	0	0	52.0353	33.1286	21.9542	22.3815	20.2084	hypothetical protein, conserved
PVX_083535	0	0	0	0	0	0	115.402	0	14.3012	0	22.5411	61.0988	vacuolar fusion protein MON1, putative
PVX_083545	0	121.034	0	0	0	295.746	0	0	21.8946	0	0	0	Plasmodium exported protein, unknown function,fam-f protein
PVX_083550	0	82.6766	0	0	0	201.957	0	0	14.9576	27.7456	7.85846	0	tryptophan-rich antigen (Pv-fam-a)
PVX_083555	845.93	788.345	351.481	903.551	224.102	0	648.654	783.893	748.323	693.214	842.003	457.724	hypothetical protein
PVX_083560	841.132	832.105	348.79	1120.79	355.91	1076.65	107.253	389.452	823.4	1132.79	823.196	567.775	Plasmodium exported protein, unknown function
PVX_083565	0	0	0	0	0	0	0	0	0	0	0	0	Plasmodium exported protein, unknown function
PVX_083570	94.7264	397.014	0	0	0	0	0	0	0	0	18.8562	0	Plasmodium exported protein, unknown function
PVX_083575	0	0	0	0	0	0	0	0	0	0	0	0	variable surface protein Vir24-related,PIR protein
PVX_083580	0	0	0	0	0	0	0	0	0	0	0	0	variable surface protein Vir24-related,PIR protein
PVX_083585	0	0	0	0	0	0	0	0	0	0	24.6769	0	variable surface protein Vir18-related,PIR protein
PVX_083590	0	51.7464	0	0	0	0	0	0	0	0	0	120.056	variable surface protein Vir12, putative,PIR protein
PVX_084090	0	415.454	0	0	0	0	0	0	0	0	0	0	liver stage associated protein 1, putative (LSAP1)
PVX_084095	0	0	0	0	0	0	0	0	0	0	0	0	hypothetical protein
PVX_084100	56.4714	78.8549	0	0	0	0	86.3706	78.4299	0	19.8412	22.4812	0	XPA binding protein 1, putative
PVX_084105	0	0	0	0	0	0	0	0	0	0	39.4234	0	serine/threonine-protein kinase Nek1, putative
PVX_084110	0	0	0	0	0	0	0	0	0	0	0	0	hypothetical protein, conserved
PVX_084115	0	0	0	0	0	0	0	0	0	45.1769	0	0	cytochrome c oxidase assembly protein COX19, putative (COX19)
PVX_084120	161.904	113.192	0	0	0	0	0	0	61.3899	170.564	64.4626	0	hypothetical protein, conserved
PVX_084125	38.3803	26.7903	190.811	122.629	12.1705	0	0	0	58.162	0	22.9182	124.244	ATP dependent RNA helicase, putative
PVX_084130	0	0	0	304.758	0	0	145.831	0	0	33.4527	75.8261	0	conserved protein, unknown function
PVX_084135	116.413	0	0	0	36.9856	0	178.39	161.775	44.1272	40.8895	69.5214	0	mitochondrial carrier protein, putative
PVX_084160	10.707	0	0	0	0	0	0	0	2.02774	3.76289	3.19663	0	dynein heavy chain, putative
PVX_084165	68.907	0	171.435	0	21.8669	0	0	0	0	24.209	41.1483	0	cyclophilin, putative
PVX_084170	0	0	0	0	0	0	0	0	0	21.5634	0	0	hypothetical protein, conserved
PVX_084175	45.8411	31.9886	0	0	4.8427	0	46.6801	127.281	23.1524	16.1096	42.5794	0	hypothetical protein, conserved
PVX_084180	208.487	0	172.903	0	0	355.812	106.335	193.062	26.3343	24.4157	41.4999	112.583	hypothetical protein, conserved
PVX_084185	0	1637.15	0	0	0	0	1829.84	0	0	0	230.552	0	hypothetical protein, conserved
PVX_084190	428.633	1202.82	0	1384.71	137.232	0	0	597.486	488.477	300.639	682.888	0	high mobility group protein B1, putative (HMGB1)
PVX_084195	0	0	0	0	0	0	0	0	0	0	5.42126	0	origin recognition complex subunit 1, putative (ORC1)
PVX_084200	0	72.5506	0	0	32.9839	0	0	0	118.085	36.479	82.6902	0	hypothetical protein, conserved
PVX_084205	0	0	0	0	73.1536	0	706.263	0	87.1042	322.418	182.859	373.657	signal recognition particle subunit SRP14, putative (SRP14)
PVX_084210	80.8156	0	100.451	0	0	0	0	112.223	30.6177	0	56.3008	0	hypothetical protein, conserved
PVX_084215	33.8509	23.6273	0	108.143	21.466	0	0	94.0062	76.9448	35.6846	60.6401	0	hypothetical protein, conserved
PVX_084220	60.4785	84.4546	0	193.355	19.1882	0	0	83.9984	45.8319	42.4974	48.1531	0	threonylcarbamoyl-AMP synthase, putative (SUA5)
PVX_084225	165.803	115.924	0	0	52.7412	0	0	0	0	116.446	33.0078	0	cytochrome c1 heme lyase, putative

Gene ID / Patient	Patient 1	Patient 2	Patient 3	Patient 4	Patient 5	Patient 6	Patient 7	Patient 8	Patient 9	Patient 10	Patient 11	Patient 12	Gene Description
PVX_084230	108.928	25.344	90.2519	0	23.0263	185.716	0	100.836	123.801	102.068	101.18	58.7662	nucleosome assembly protein, putative (NAPL)
PVX_084235	255.143	0	0	0	0	0	0	0	193.612	89.5553	152.398	0	ubiquitin-conjugating enzyme E2 4, putative
PVX_084240	0	0	0	0	0	0	0	0	0	0	0	0	hypothetical protein, conserved
PVX_084245	0	0	0	0	0	0	0	0	0	0	30.3622	0	hypothetical protein, conserved
PVX_084250	0	42.7477	0	0	38.8497	0	0	85.0334	0	43.0205	48.7461	0	hypothetical protein, conserved
PVX_084255	354.866	123.932	220.843	283.86	56.3339	0	0	246.505	268.978	311.655	194.257	143.799	eukaryotic translation initiation factor 5A
PVX_084260	0	0	0	0	0	0	0	0	0	29.7505	0	0	hypothetical protein, conserved
PVX_084265	184.95	0	0	0	0	0	0	0	35.0476	32.4853	18.408	0	hypothetical protein, conserved
PVX_084270	0	0	0	0	0	0	0	0	35.4137	32.8243	18.6002	0	cytidine deaminase, putative
PVX_084275	0	0	418.305	0	0	0	0	0	0	117.754	33.3789	272.373	hypothetical protein, conserved
PVX_084277	0	0	0	0	0	0	0	0	0	0	0	0	conserved Plasmodium protein, unknown function
PVX_084280	31.2395	43.6076	0	99.7932	29.7131	0	47.7411	173.504	11.8346	32.9321	18.654	0	hypothetical protein, conserved
PVX_084285	87.7061	0	0	0	13.9078	0	0	0	0	46.2263	34.9151	0	O-phosphoseryl-tRNA(Sec) selenium transferase, putative (SEPSECS)
PVX_084290	0	0	0	0	0	0	0	0	0	0	0	0	HAD domain ookinete protein, putative (HADO)
PVX_084295	0	0	0	0	17.7137	0	0	0	21.1567	0	11.1143	0	hypothetical protein, conserved
PVX_084300	35.3763	0	0	0	0	0	0	0	0	24.8616	7.04144	0	hypothetical protein, conserved
PVX_084305	53.0409	0	0	0	5.60362	0	0	24.5458	46.8811	12.4264	70.3815	0	zinc finger protein, putative
PVX_084310	186.298	65.0658	231.905	894.237	88.7322	0	142.634	388.247	141.212	229.053	352.3	0	hypothetical protein, conserved
PVX_084315	0	0	0	0	0	0	0	0	0	0	45.287	0	glyoxalase II, putative
PVX_084320	0	0	0	0	0	0	0	0	0	0	0	0	hypothetical protein
PVX_084325	10.5178	7.33903	52.2492	0	13.3318	0	16.063	29.202	7.9679	22.1777	16.7475	34.0213	high mobility group protein B3, putative (HMGB3)
PVX_084330	79.4613	55.4572	65.8193	0	8.39667	135.437	0	0	40.1357	27.9229	52.7203	85.7147	hypothetical protein, conserved
PVX_084335	0	0	0	407.904	40.4699	0	0	0	96.5501	134.182	177.454	206.637	shewanella-like protein phosphatase 2, putative (SHLP2)
PVX_084340	0	0	0	0	0	0	0	0	0	0	38.7325	0	IMP-specific 5'-nucleotidase, putative
PVX_084345	55.2641	134.996	68.6667	0	70.079	141.297	42.2232	268.56	146.548	126.229	159.498	44.7113	eukaryotic translation initiation factor 3 subunit 8, putative
PVX_084350	11.7665	0	0	0	0	0	0	0	0	0	4.68397	0	hypothetical protein, conserved
PVX_084355	110.382	0	0	0	70.1288	0	0	0	83.6791	0	43.9463	0	rhodanese like protein, putative
PVX_084360	0	0	0	0	0	0	0	0	0	0	0	0	Tat binding protein 1(TBP-1)-interacting protein, putative
PVX_084365	18.9816	0	0	0	0	0	0	0	64.7125	26.6817	26.4466	0	DNA-directed RNA polymerase III subunit, putative
PVX_084370	134.906	94.2022	0	0	64.214	345.332	103.202	93.6914	178.919	71.0947	214.826	0	eukaryotic translation initiation factor 5, putative
PVX_084375	0	0	0	0	0	0	0	0	0	0	47.0319	0	hypothetical protein, conserved
PVX_084380	0	22.0347	0	0	10.0093	0	0	0	0	0	6.28379	0	hypothetical protein, conserved
PVX_084385	0	0	0	0	0	0	0	0	0	0	2.39489	0	hypothetical protein, conserved
PVX_084390	53.5127	74.6949	66.4889	0	33.9283	0	0	74.2991	40.5437	65.8158	53.2562	0	small subunit rRNA processing factor, putative
PVX_084395	0	0	0	0	6.96168	0	0	0	0	0	17.486	0	hypothetical protein
PVX_084400	0	0	0	0	0	0	0	0	0	0	0	0	hypothetical protein, conserved
PVX_084405	0	0	0	0	0	0	0	0	0	0	0	0	hypothetical protein
PVX_084410	0	0	0	0	0	0	0	0	0	34.5553	0	0	hypothetical protein, conserved
PVX_084415	0	0	0	0	0	0	0	0	0	90.8487	51.5343	0	RNA-binding protein, putative
PVX_084417	0	0	0	0	18.548	0	89.4232	81.1983	22.1522	82.1639	104.734	0	tRNA delta(2)-isopentenylpyrophosphate transferase, putative
PVX_084420	0	0	0	0	0	0	0	0	0	0	0	0	41K blood stage antigen precursor 41-3, putative
PVX_084425	0	0	0	0	0	0	51.328	0	0	0	0	0	hypothetical protein, conserved
PVX_084430	0	0	0	0	23.1715	0	0	0	0	51.3004	14.5331	0	hypothetical protein, conserved
PVX_084435	0	0	0	0	0	0	0	0	0	106.544	60.4545	0	50S ribosomal protein L29, putative
PVX_084440	0	30.0986	17.856	0	6.83422	0	0	19.9606	5.44639	10.1067	12.8788	0	hypothetical protein, conserved
PVX_084445	0	0	0	0	0	0	0	12.8319	3.50124	3.24848	9.19901	0	cysteine repeat modular protein 3, putative (CRMP3)
PVX_084450	0	105.897	0	0	0	0	232.4	0	172.311	319.203	241.261	0	hypothetical protein, conserved
PVX_084455	20.2623	0	50.3421	0	0	0	0	0	0	0	0	0	amino acid transporter, putative
PVX_084460	0	0	0	0	0	0	0	0	16.5179	0	8.6791	0	hypothetical protein, conserved
PVX_084462	0	0	0	0	666.789	0	0	0	772.252	0	803.662	0	mitochondrial import inner membrane translocase subunit TIM10, putative (TIM10)
PVX_084465	78.9353	0	196.44	0	0	404.257	120.815	109.653	59.8265	55.4616	47.1374	0	hypothetical protein, conserved
PVX_084470	29.9233	20.8849	0	0	9.48679	0	0	0	0	10.515	5.95601	0	zinc finger protein, putative
PVX_084475	0	0	0	0	6.75759	0	0	0	24.2279	0	12.7302	0	hypothetical protein, conserved
PVX_084480	546.095	0	683.246	0	87.0847	0	0	380.028	103.612	0	54.3655	0	hypothetical protein, conserved
PVX_084485	0	0	0	0	8.91971	0	0	0	0	0	0	0	hypothetical protein, conserved
PVX_084490	152.782	0	0	0	48.5838	0	0	0	57.9277	53.6542	212.906	0	U6 snRNA-associated Sm-like protein LSm7, putative
PVX_084495	0	0	0	0	0	0	0	27.7169	15.1249	7.01562	7.94723	0	zinc finger transcription factor, putative (KROX1)
PVX_084500	0	0	118.251	151.993	0	0	0	0	0	0	9.46637	0	cytosolic iron-sulfur protein assembly protein 1, putative (CIA1)
PVX_084505	0	0	0	0	10.0804	0	0	0	0	0	6.32845	0	3',5'-cyclic nucleotide phosphodiesterase, putative
PVX_084510	0	0	0	0	0	0	0	0	0	0	0	0	porphobilinogen deaminase, putative (PBGD)
PVX_084515	0	0	0	0	35.3706	0	170.594	154.722	0	78.2198	66.4936	0	hypothetical protein, conserved
PVX_084519	0	0	0	0	155.537	0	0	0	185.152	0	194.33	397.251	ATP synthase mitochondrial F1 complex assembly factor 1, putative (ATP11)

Gene ID / Patient	Patient 1	Patient 2	Patient 3	Patient 4	Patient 5	Patient 6	Patient 7	Patient 8	Patient 9	Patient 10	Patient 11	Patient 12	Gene Description
PVX_084521	0	19.2791	68.6455	0	0	0	0	0	10.4645	19.4139	21.9929	0	ABC transporter, putative
PVX_084525	60.7247	0	0	194.144	0	0	0	0	23.0093	21.3352	72.5237	0	50S ribosomal protein L1, apicoplast, putative (RPL1)
PVX_084530	0	0	0	0	0	0	0	0	0	0	2.92541	0	hypothetical protein
PVX_084535	0	82.6323	0	0	37.5737	0	0	0	44.8275	0	0	0	syntaxin 5, putative
PVX_084540	0	0	0	0	0	0	0	0	0	8.55801	4.84736	0	hypothetical protein, conserved
PVX_084545	151.489	105.897	0	0	0	0	0	421.183	114.874	212.802	30.1576	0	hypothetical protein, conserved
PVX_084550	0	0	0	0	0	0	0	0	0	0	0	0	hypothetical protein
PVX_084555	30.5568	0	0	0	9.68777	0	0	0	0	0	36.4926	0	general transcription factor 3C polypeptide 5, putative
PVX_084560	152.881	0	0	0	24.2621	0	0	0	115.87	26.8547	91.2952	0	hypothetical protein, conserved
PVX_084565	22.1916	30.974	0	0	0	0	0	30.8103	25.2193	15.5968	35.3362	0	hypothetical protein, conserved
PVX_084570	0	0	0	0	22.4736	0	0	0	0	24.8792	14.096	0	hypothetical protein, conserved
PVX_084575	0	0	0	0	0	0	0	0	0	0	0	0	mannosyltransferase, putative
PVX_084580	0	0	0	0	0	0	0	0	0	0	0	0	kinesin-7, putative
PVX_084585	0	0	0	0	0	0	0	0	0	0	0	0	heat shock protein, class I, putative
PVX_084590	40.497	28.2612	0	0	0	0	30.9335	56.224	46.0216	14.2312	28.2118	0	hypothetical protein, conserved
PVX_084595	0	16.3252	0	0	0	0	0	32.4778	0	8.22027	13.9681	0	DNA replication licensing factor MCM8, putative (MCM8)
PVX_084600	167.353	234.021	417.519	536.656	106.473	0	0	0	63.4587	176.301	33.3164	543.723	DnaJ domain containing protein,heat shock protein DNAJ homologue Pfj4, putative
PVX_084605	0	0	146.079	0	55.9001	0	0	81.5715	0	41.2706	35.0719	95.1174	hypothetical protein, conserved
PVX_084610	0	0	0	0	0	0	16.3279	0	8.09927	7.51444	0	0	lysine-specific histone demethylase 1, putative (LSD1)
PVX_084615	65.9311	0	0	105.312	10.4521	0	0	0	0	34.7515	52.4925	0	DNA replication licensing factor MCM5, putative
PVX_084620	394.314	137.668	326.979	420.281	62.5609	336.439	301.633	273.845	298.831	507.966	431.69	319.362	polyubiquitin 5, putative
PVX_084625	0	57.2479	50.9538	0	6.50039	0	31.3308	0	15.5374	21.6207	20.4099	0	P-type ATPase4, putative
PVX_084630	429.048	99.963	356.557	458.299	45.4673	0	0	198.796	162.664	0	56.9411	0	glutathione peroxidase-like thioredoxin peroxidase, putative
PVX_084635	0	29.8658	106.365	0	13.5683	0	0	0	113.465	75.1658	51.0957	0	hypothetical protein, conserved
PVX_084640	0	0	0	0	54.1514	0	0	0	322.731	239.088	67.7738	0	hypothetical protein
PVX_084645	179.188	62.5437	222.752	0	56.8298	0	0	124.418	67.889	188.884	107	0	hypothetical protein, conserved
PVX_084650	347.884	173.476	0	476.519	47.2908	0	0	207.053	282.444	366.7	178.06	80.4656	DnaJ domain containing protein
PVX_084655	0	0	0	0	43.986	0	0	192.332	104.918	48.5972	55.0919	224.599	trafficking protein particle complex subunit 5, putative
PVX_084660	0	22.4547	159.916	102.773	10.2001	0	0	44.6706	0	45.2195	57.6317	52.0634	succinyl-CoA ligase, putative
PVX_084665	54.7107	0	136.062	0	34.7117	279.991	0	0	0	38.4456	43.5604	0	hypothetical protein, conserved
PVX_084670	0	210.367	0	0	143.538	0	0	0	0	52.8429	119.819	0	hypothetical protein, conserved
PVX_084675	146.171	0	121.152	155.722	30.9086	0	74.5023	135.329	55.3813	119.838	77.5864	0	hypothetical protein, conserved
PVX_084680	0	0	0	0	0	0	0	0	0	0	0	0	hypothetical protein, conserved
PVX_084685	65.9554	23.0175	0	0	20.9118	0	100.801	91.5806	74.9596	69.5285	78.7678	0	ribonucleoside-diphosphate reductase large chain, putative
PVX_084687	0	0	0	0	0	0	0	427.061	0	215.275	61.0764	0	conserved Plasmodium protein, unknown function
PVX_084690	37.8447	0	0	0	0	0	0	52.5507	14.3375	13.298	22.5983	0	N-acetyltransferase, putative
PVX_084695	0	0	555.216	0	0	0	0	0	0	156.011	88.4776	0	histidine triad protein, putative
PVX_084698	0	0	0	0	0	0	0	0	0	57.4782	0	0	ATP-dependent Clp protease proteolytic subunit, putative (ClpR)
PVX_084702	0	25.8262	0	0	0	0	0	0	42.0521	52.0046	22.0938	0	ATP-dependent Clp protease proteolytic subunit, putative
PVX_084705	37.8927	0	0	0	0	0	0	0	0	0	22.627	0	cGMP-dependent protein kinase, putative (PKG)
PVX_084710	0	0	0	0	27.0387	0	0	118.322	64.555	0	50.8613	0	GTP-binding protein, putative
PVX_084715	32.1023	0	0	0	10.1781	0	0	44.5744	24.3231	11.2805	44.7282	0	hypothetical protein
PVX_084720	70.9208	0	0	0	22.4875	0	0	49.2388	80.6042	62.3017	35.2909	0	translocon component PTEX150, putative (PTEX150)
PVX_084725	0	0	0	0	0	0	0	0	4.34389	0	4.56512	0	hypothetical protein, conserved
PVX_084730	0	12.7148	45.2661	58.1826	11.5497	0	0	0	20.7056	12.8058	7.25309	0	hypothetical protein, conserved
PVX_084735	56.4714	157.71	0	0	0	0	86.3706	0	85.5882	119.047	44.9624	91.4504	glucose-6-phosphate isomerase, putative
PVX_084740	122.634	0	0	0	0	0	375.921	0	46.4874	0	24.4125	0	hypothetical protein, conserved
PVX_084745	138.026	48.1918	0	0	21.9007	0	0	0	52.3029	24.2463	27.4745	0	GTPase Era, putative (ERA)
PVX_084750	0	78.1575	92.7764	0	0	0	0	51.8272	56.5607	26.23	66.8619	0	ataxin-2 like protein, putative
PVX_084755	0	0	0	0	0	0	0	0	0	6.23908	0	0	hypothetical protein, conserved
PVX_084760	0	0	422.279	0	0	0	0	0	128.359	59.4332	101.084	0	hypothetical protein, conserved
PVX_084765	0	55.668	0	0	0	0	0	0	0	0	31.7329	129.165	conserved protein, unknown function
PVX_084770	29.8984	6.95404	49.5079	0	3.15808	0	15.2202	0	26.4248	21.0145	33.7217	16.1182	NAD(P)H-dependent glutamate synthase, putative
PVX_084775	0	0	0	0	0	0	0	0	0	0	11.66	0	hypothetical protein, conserved
PVX_084780	0	60.466	215.489	0	27.4843	0	0	0	0	60.8243	51.6978	0	ribonuclease H2 subunit B, putative
PVX_084785	0	0	0	264.17	26.2137	0	0	114.716	93.8823	58.0193	65.7499	0	cytochrome c oxidase assembly protein, putative
PVX_084790	0	0	0	0	0	0	0	0	0	0	15.6161	0	hypothetical protein, conserved
PVX_084795	205.455	143.468	170.384	0	108.664	0	314.356	380.505	233.561	240.608	272.643	0	mitochondrial acidic protein MAM33, putative
PVX_084800	0	0	0	0	0	0	0	0	0	0	0	0	mitochondrial import inner membrane translocase subunit TIM17, putative (TIM17)
PVX_084805	183.293	42.6601	151.973	0	38.7701	312.738	0	0	0	150.264	72.9693	0	methionine aminopeptidase 2, putative (METAP2)
PVX_084810	8.2188	5.73437	10.2053	13.1173	6.51003	0	0	0	9.33897	11.5537	10.6329	0	dynein-related AAA-type ATPase, putative
PVX_084815	0	0	0	0	0	0	0	0	0	0	0	0	hypothetical protein, conserved

Gene ID / Patient	Patient 1	Patient 2	Patient 3	Patient 4	Patient 5	Patient 6	Patient 7	Patient 8	Patient 9	Patient 10	Patient 11	Patient 12	Gene Description
PVX_084820	399.578	278.956	0	0	79.2144	0	305.506	971.104	246.015	333.442	159.07	0	Hsp70/Hsp90 organizing protein, putative (HOP)
PVX_084825	181.25	0	452.365	0	0	0	0	252.036	68.7346	63.6423	108.251	0	calmodulin, putative
PVX_084830	0	0	0	0	0	0	0	0	65.3012	30.2658	68.5985	0	queuine tRNA-ribosyltransferase, putative
PVX_084835	0	0	0	0	0	0	0	0	59.4517	0	31.2146	0	ABC transporter, putative
PVX_084837	27.39	0	0	0	4.34009	0	0	0	10.375	0	2.72583	0	protein kinase, putative
PVX_084840	0	0	0	0	0	0	0	0	5.19229	0	5.45666	0	hypothetical protein, conserved
PVX_084845	0	0	0	0	0	0	0	0	12.6098	40.9445	33.129	80.7724	hypothetical protein, conserved
PVX_084850	345.03	0	0	553.289	0	0	529.667	719.608	130.836	242.312	274.754	0	microsomal signal peptidase 12 kDa subunit, putative
PVX_084855	0	14.5048	0	0	26.352	106.26	63.5062	0	7.87338	14.608	53.7806	33.6251	DNA topoisomerase II, putative
PVX_084860	26.6957	3.72526	13.2597	0	6.76674	27.2836	0	14.823	22.2453	11.2584	21.2536	8.63385	hypothetical protein, conserved
PVX_084870	0	47.1011	0	0	0	0	0	0	0	23.6982	13.4266	0	chromatin assembly factor 1 P55 subunit, putative
PVX_084875	0	0	0	0	0	0	0	0	0	0	0	0	hypothetical protein, conserved
PVX_084885	30.0641	62.9494	0	96.0354	9.53144	0	45.9431	41.7435	22.7785	21.1288	41.8882	0	hypothetical protein, conserved
PVX_084895	0	461.463	0	0	105.176	0	0	458.593	250.023	577.73	196.72	0	H/ACA ribonucleoprotein complex subunit 3, putative (NOP10)
PVX_084900	0	0	0	0	0	0	0	0	0	0	0	0	SF-assemblin/beta giardin domain containing protein
PVX_084905	52.1293	0	0	166.623	16.5359	0	159.437	144.793	39.5025	0	62.2575	0	hypothetical protein, conserved
PVX_084910	0	0	0	0	0	0	291.378	0	0	66.6074	37.7667	0	protein-L-isoaspartate O-methyltransferase beta-aspartate methyltransferase, putative
PVX_084915	25.8446	36.0743	0	0	0	0	0	0	9.79047	0	5.14414	0	hypothetical protein, conserved
PVX_084920	71.4942	49.9258	0	0	0	0	0	0	81.2761	25.1176	28.4623	0	RNA methyltransferase, putative
PVX_084925	0	0	0	0	0	0	0	0	0	0	0	0	hypothetical protein, conserved
PVX_084930	0	13.249	47.1685	0	0	0	0	0	7.19181	0	15.1155	0	hypothetical protein, conserved
PVX_084935	0	0	0	0	0	0	0	0	7.11482	0	14.9537	30.3842	hypothetical protein, conserved
PVX_084940	208.811	97.2088	0	0	44.1767	0	213.001	96.6811	52.7506	97.8146	221.677	0	voltage-dependent anion-selective channel protein, putative
PVX_084945	0	0	0	0	0	0	120.389	0	29.808	82.9	125.258	254.917	syntaxin, putative
PVX_084950	0	29.6953	0	0	0	0	0	0	0	0	8.46737	0	hypothetical protein, conserved
PVX_084955	605.957	953.073	1133.31	485.568	144.513	0	697.2	421.183	1608.23	1383.21	1085.67	1229.9	60S ribosomal protein L14, putative
PVX_084960	0	0	0	0	0	0	0	0	0	26.5152	0	0	ATP-specific succinyl-CoA synthetase beta subunit, putative
PVX_084965	0	0	0	0	13.766	0	0	0	0	0	8.63987	0	mitogen-activated protein kinase 1, putative (MAPK1)
PVX_084970	34.3182	0	0	0	0	0	52.4512	0	0	24.1181	20.4924	55.5406	hypothetical protein, conserved
PVX_084975	0	24.0088	85.4949	0	10.9064	0	0	0	26.0624	36.2605	27.3863	0	hypothetical protein, conserved
PVX_084980	0	0	0	0	0	0	0	0	0	0	0	0	hypothetical protein, conserved
PVX_084985	0	0	0	0	0	0	0	0	0	72.7051	20.6008	0	hypothetical protein, conserved
PVX_084990	0	212.877	0	0	96.8358	0	233.592	211.668	57.7304	0	30.3116	247.242	50S ribosomal protein L17, putative
PVX_084995	0	0	709.42	911.851	0	0	0	0	0	0	112.865	0	cytochrome c oxidase subunit 2, putative
PVX_085000	0	43.4014	0	0	0	0	0	0	0	21.8389	12.3727	0	hypothetical protein, conserved
PVX_085005	47.1326	32.9033	234.381	0	0	0	0	65.4539	35.7149	33.1217	159.487	76.307	glutamate dehydrogenase (NADP+), putative
PVX_085010	0	0	101.414	0	12.9368	0	0	0	15.4554	14.3343	32.4799	66.0341	exodeoxyribonuclease III, putative
PVX_085015	0	64.2622	0	0	0	0	140.869	127.818	139.469	96.9551	54.9401	0	hypothetical protein, conserved
PVX_085020	0	0	0	0	0	0	0	0	0	18.6749	21.1593	0	hypothetical protein, conserved
PVX_085025	41.3699	28.8679	0	0	13.1106	0	0	0	15.6705	48.4614	21.9578	22.3048	hypothetical protein, conserved
PVX_085030	0	0	0	0	0	0	0	0	0	0	0	0	aspartyl protease, putative
PVX_085035	0	0	0	0	0	0	0	0	0	0	21.3413	0	serine/threonine protein phosphatase 2A activator, putative (PTPA)
PVX_085040	0	0	0	0	0	0	0	0	0	0	0	0	hypothetical protein, conserved
PVX_085045	24.5473	0	0	0	0	0	0	0	0	0	4.88592	0	ATP-dependent DNA helicase, putative
PVX_085050	95.9006	66.9283	59.5731	76.5721	30.3996	0	73.2622	166.436	72.6575	92.6756	114.529	0	coatamer protein, beta subunit, putative
PVX_085055	0	0	0	0	31.4474	0	151.658	0	37.5312	34.7846	59.1352	160.552	ribosomal protein L15, putative
PVX_085060	209.705	146.702	0	0	66.7784	0	322.309	0	318.165	0	125.251	0	hypothetical protein, conserved
PVX_085065	0	33.414	0	0	15.1812	0	73.1854	132.939	90.6726	33.6353	38.1085	0	diphthamide synthesis protein, putative
PVX_085070	13.2627	0	0	0	0	0	0	0	5.02376	4.66086	5.27956	0	rRNA (adenosine-2'-O-)-methyltransferase, putative
PVX_085080	0	0	0	0	0	0	0	0	0	0	0	0	CPW-WPC family protein, putative
PVX_085085	0	0	0	0	0	0	0	0	0	0	7.55194	0	transcription factor with AP2 domain(s), putative (ApiAP2)
PVX_085090	0	0	0	0	35.6949	0	86.0443	78.1339	21.3163	59.2993	55.991	0	50S ribosomal protein L15, putative
PVX_085095	0	0	0	0	0	0	0	0	0	0	0	0	hypothetical protein, conserved
PVX_085100	0	19.7674	0	0	0	0	0	39.3252	10.7295	9.95266	45.0996	0	hypothetical protein, conserved
PVX_085105	223.243	0	0	0	0	0	0	0	84.6191	0	88.8793	0	transcription initiation TFIID-like, putative
PVX_085110	0	0	0	348.228	0	0	0	0	41.2288	38.2072	0	0	hypothetical protein, conserved
PVX_085115	127.892	0	0	0	0	0	0	0	48.4822	44.9188	76.3777	0	peptide chain release factor 1, putative
PVX_085120	0	0	0	0	0	0	0	0	20.5146	4.75817	8.08469	0	protein kinase, putative
PVX_085125	286.332	159.932	0	366.138	163.509	0	175.177	238.604	368.873	442.651	250.775	278.219	proliferation-associated protein 2g4, putative
PVX_085130	0	0	0	117.629	0	0	0	0	69.7408	12.937	21.9847	0	transporter, putative
PVX_085135	38.9651	0	0	0	0	0	0	0	0	0	0	0	WW domain-binding protein 11, putative
PVX_085140	0	0	0	250.282	0	0	0	0	29.6516	0	77.8755	0	hypothetical protein, conserved

Gene ID / Patient	Patient 1	Patient 2	Patient 3	Patient 4	Patient 5	Patient 6	Patient 7	Patient 8	Patient 9	Patient 10	Patient 11	Patient 12	Gene Description
PVX_085145	279.156	129.996	231.664	0	236.373	476.756	427.455	775.686	564.262	555.693	537.162	150.845	hypothetical protein, conserved
PVX_085150	0	0	0	0	32.4925	0	0	0	38.7763	35.9372	81.4612	0	exosome complex exonuclease RRP41, putative (RRP41)
PVX_085152	0	0	0	0	0	0	0	0	0	0	0	0	conserved Plasmodium protein, unknown function
PVX_085155	155.663	0	0	165.849	16.4591	0	79.3483	0	39.3192	127.618	30.9844	0	CorA-like Mg2+ transporter protein, putative
PVX_085160	40.136	0	99.7745	128.245	0	205.313	0	111.467	15.2058	42.3088	31.9555	0	DNA mismatch repair enzyme, putative
PVX_085165	0	45.3561	161.587	207.696	61.8332	0	99.3742	0	49.2266	68.4639	25.8591	0	hypothetical protein, conserved
PVX_085170	0	29.4614	0	134.82	0	0	0	29.3058	0	7.41767	29.4096	0	hypothetical protein
PVX_085175	118.416	82.7418	295.02	0	0	0	181.47	329.121	0	124.777	141.435	0	selenoprotein, putative (Sel4)
PVX_085180	0	10.9952	0	0	0	0	0	0	11.937	33.2232	9.4085	0	hypothetical protein, conserved
PVX_085185	0	0	0	88.9476	8.82806	0	0	0	0	0	33.2563	0	hypothetical protein, conserved
PVX_085190	387.621	0	483.886	0	61.6927	0	297.728	269.536	73.5043	204.145	347.264	315.076	ubiquinol-cytochrome c reductase hinge protein, putative
PVX_085195	0	20.4258	0	0	9.2766	0	0	0	11.0877	10.2866	29.1306	0	hypothetical protein, conserved
PVX_085200	53.1157	0	0	84.8268	16.8382	0	0	0	40.2429	27.9975	68.7195	42.9719	phosphoenolpyruvate carboxylase, putative (PEPC)
PVX_085203	22.8898	0	113.749	0	0	0	34.9715	0	8.67096	8.04369	4.55599	0	conserved Plasmodium protein, unknown function
PVX_085205	94.9655	33.1479	0	0	15.0602	0	0	0	17.9901	50.0515	56.7078	0	ATP-binding cassette sub-family G member 2, putative (ABCG2)
PVX_085209	0	0	0	0	0	0	0	0	0	0	0	0	dynein-associated protein, putative
PVX_085211	0	0	0	0	0	0	0	0	20.806	19.2935	10.9302	0	dynein-associated protein, putative
PVX_085215	89.5151	187.574	222.836	286.422	85.2632	458.587	137.054	248.726	135.7	314.46	124.731	145.097	Probable protein arginine N-methyltransferase, putative
PVX_085220	329.773	806.975	0	0	209.794	846.629	759.183	687.737	875.311	868.521	426.728	267.836	basic transcription factor 3b, putative
PVX_085225	614.275	912.505	765.96	0	122.087	394.125	471.212	640.46	669.602	539.302	397.432	748.116	60S ribosomal protein L21, putative (RPL21)
PVX_085230	187.468	0	0	0	0	0	143.533	0	71.0501	32.9274	130.611	0	hypothetical protein, conserved
PVX_085235	37.9088	52.922	0	0	12.0208	0	57.9456	0	14.3618	26.641	22.6366	122.716	hypothetical protein
PVX_085240	174.322	60.8787	0	0	0	0	133.44	121.09	132.13	91.8582	34.7002	0	hypothetical protein, conserved
PVX_085245	0	0	0	0	0	0	0	0	0	0	0	0	hypothetical protein, conserved
PVX_085250	0	0	0	0	0	0	0	0	0	0	49.8051	407.316	hypothetical protein, conserved
PVX_085255	0	0	0	0	0	0	0	0	0	0	0	0	DEAD/DEAH box helicase, putative
PVX_085260	242.281	0	0	0	0	0	0	0	0	85.0979	24.115	0	hypothetical protein, conserved
PVX_085265	0	0	206.671	0	0	0	0	115.355	31.4684	87.5133	33.058	0	enoyl-CoA hydratase, putative
PVX_085270	183.67	641.215	456.857	0	174.824	0	374.61	340.134	324.776	537.757	499.647	99.1587	ribosomal protein L7A, putative
PVX_085275	1066.83	812.983	724.445	0	461.975	1490.89	148.526	673.751	1065.98	885.786	868.761	628.949	60S ribosomal protein L5, putative
PVX_085290	21.6285	30.1878	0	0	20.5669	0	33.0432	0	16.3862	22.8015	30.1346	0	vacuolar protein sorting-associated protein 3, putative (VPS3)
PVX_085295	21.0486	58.7564	0	0	13.3434	107.611	32.1566	87.6691	39.8669	88.7609	62.8427	34.0524	hypothetical protein, conserved
PVX_085300	0	0	0	0	0	0	0	81.3578	0	20.5813	0	0	calcium-dependent protein kinase, putative
PVX_085305	0	0	0	0	0	0	0	0	0	10.0001	0	0	hypothetical protein
PVX_085310	0	0	52.5193	0	0	0	0	0	8.00729	0	4.20732	0	hypothetical protein, conserved
PVX_085315	33.5463	0	0	0	0	0	0	0	0	0	0	0	serine/threonine protein phosphatase 7, putative (PP7)
PVX_085320	0	0	0	0	24.3246	0	0	0	58.0837	53.8474	45.7649	124.177	cyclophilin, putative
PVX_085325	0	0	0	0	0	0	0	64.6029	0	16.3457	55.5581	0	hypothetical protein, conserved
PVX_085330	180.908	94.7168	0	0	28.688	231.391	0	62.8064	51.4058	63.5658	63.016	73.2181	GTPase, putative
PVX_085335	0	0	0	0	0	0	0	0	0	0	13.2469	0	14-3-3 protein, putative
PVX_085340	69.9847	0	174.122	0	0	0	0	0	106.079	24.5875	125.376	113.377	actin, putative
PVX_085345	95.4849	0	0	101.676	30.2736	0	48.6419	44.1938	120.577	44.7371	82.3576	0	hypothetical protein
PVX_085350	0	0	0	0	0	0	0	0	26.8347	0	56.3841	0	hypothetical protein, conserved
PVX_085355	65.8157	0	0	0	41.7687	0	0	0	24.9392	46.2466	52.403	0	ERAD-associated E3 ubiquitin-protein ligase HRD1, putative (HRD1)
PVX_085360	76.3799	66.627	47.4404	0	18.1566	97.6173	0	79.5306	72.3324	60.3925	60.8105	30.8901	hypothetical protein, conserved
PVX_085365	0	0	0	0	0	0	144.443	131.054	107.25	0	18.7767	0	DnaJ domain containing protein
PVX_085370	0	0	0	0	38.8081	0	0	0	0	0	0	0	hypothetical protein, conserved
PVX_085375	193.55	0	0	0	61.4631	0	0	0	73.3566	203.97	57.7921	313.793	hypothetical protein, conserved
PVX_085380	0	0	0	0	0	0	0	0	118.874	219.788	0	0	hypothetical protein, conserved
PVX_085385	0	0	0	797.311	79.0701	0	381.744	0	376.473	261.228	49.3908	0	copper transporter, putative
PVX_085390	0	0	0	0	0	0	0	0	0	0	36.5141	0	hypothetical protein, conserved
PVX_085395	93.919	0	0	0	0	0	0	195.64	53.3755	33.0001	9.34715	0	hypothetical protein, conserved
PVX_085405	0	480.154	1726.83	0	0	0	0	0	259.604	0	0	0	mitochondrial import inner membrane translocase subunit TIM8, putative (TIM8)
PVX_085410	0	92.554	0	0	0	0	0	0	100.411	46.5131	0	0	DNA-directed RNA polymerases III 39 kDa polypeptide, putative
PVX_085415	0	0	0	0	0	0	0	50.6233	0	0	0	0	hypothetical protein, conserved
PVX_085420	1529.32	2154.84	1939.77	2493.28	493.6	3996.97	3588.21	2138.63	2329.01	2141.82	1828.15	2526.11	40S ribosomal protein S25, putative
PVX_085425	73.3626	51.2076	273.533	0	46.5249	0	56.0671	50.9347	97.2768	38.6678	167.928	118.738	hypothetical protein, conserved
PVX_085430	0	196.588	0	0	89.5606	0	432.496	0	0	0	279.505	457.547	hypothetical protein, conserved
PVX_085435	0	0	0	0	24.5776	0	0	0	0	27.2031	15.4134	0	mannose-1-phosphate guanyltransferase, putative
PVX_085440	0	0	0	0	0	0	0	0	0	0	43.6234	0	dynein-associated protein, putative
PVX_085445	0	0	0	0	0	0	0	0	11.5759	0	18.2463	0	surface protein P113, putative (P113)
PVX_085450	62.3753	0	0	0	0	0	0	0	23.635	43.8299	111.743	101.028	pantothenate kinase, putative (PANK)

Gene ID / Patient	Patient 1	Patient 2	Patient 3	Patient 4	Patient 5	Patient 6	Patient 7	Patient 8	Patient 9	Patient 10	Patient 11	Patient 12	Gene Description
PVX_085455	31.2613	21.8191	0	0	19.8226	0	95.5489	130.219	35.5286	21.9701	18.667	0	hypothetical protein, conserved
PVX_085460	38.4793	0	191.303	0	24.4038	0	117.637	0	58.3121	27.0419	45.9546	0	glycine--tRNA ligase, putative
PVX_085465	0	0	0	0	0	0	0	0	46.6153	0	0	0	hypothetical protein, conserved
PVX_085470	26.3934	55.2609	0	0	8.36693	0	40.3289	0	9.9984	9.27471	10.5067	0	hypothetical protein, conserved
PVX_085475	0	0	0	0	0	0	0	0	0	0	0	0	hypothetical protein, conserved
PVX_085480	72.3613	50.5319	0	231.423	0	0	0	201.029	164.525	76.2665	100.827	0	spliceosome-associated protein 49, putative
PVX_085485	44.004	92.1539	0	0	13.9557	0	0	122.214	66.6869	92.7707	87.5882	0	YTH domain-containing protein, putative
PVX_085490	123.453	143.627	0	131.492	39.1499	421.023	125.816	114.287	233.858	216.893	204.773	0	glutathione reductase, putative
PVX_085495	70.4807	98.4349	175.359	225.397	67.1016	0	107.845	293.701	267.078	321.902	154.323	114.182	conserved Plasmodium protein, unknown function
PVX_085500	68.2241	0	0	0	21.6498	0	0	94.7635	0	0	27.1603	0	hypothetical protein, conserved
PVX_085505	0	0	0	0	0	0	0	0	0	0	0	0	hypothetical protein, conserved
PVX_085510	29.6201	62.0076	0	0	18.7743	0	22.6213	0	22.4397	26.023	26.5299	0	hypothetical protein, conserved
PVX_085515	0	0	0	0	0	0	0	192.035	52.3779	0	82.5102	224.251	glutathione S-transferase, putative
PVX_085520	50.1995	0	124.827	0	31.8459	0	0	69.7149	57.0594	141.105	89.9288	0	thioredoxin-like protein, putative
PVX_085525	31.3269	21.8649	0	0	0	160.208	0	0	11.8678	0	18.7062	0	ATP-dependent RNA helicase, putative
PVX_085530	0	0	65.0106	0	0	0	39.9749	0	9.9107	0	20.8292	0	hypothetical protein, conserved
PVX_085535	0	0	0	0	0	0	43.1841	0	10.7058	0	22.4999	0	DEAD/DEAH box ATP-dependent RNA helicase, putative
PVX_085540	0	0	0	0	0	0	0	0	0	0	85.771	0	calmodulin, putative
PVX_085545	0	0	0	0	0	0	0	0	0	0	21.3158	0	hypothetical protein, conserved
PVX_085550	0	0	0	0	0	0	0	0	0	0	5.11963	0	liver specific protein 1, putative (LISP1)
PVX_085555	0	99.6922	0	0	45.3063	0	0	0	54.0976	75.2327	99.4592	0	ubiquitin fusion degradation protein, putative
PVX_085560	0	0	0	0	0	0	157.243	0	38.9098	108.182	81.7415	166.463	hypothetical protein
PVX_085565	58.8239	20.5278	0	93.9505	9.32455	0	44.9456	40.8378	0	0	87.8134	47.5938	DNA replication licensing factor MCM2, putative (MCM2)
PVX_085567	0	0	0	0	0	0	0	0	0	0	36.8297	0	conserved Plasmodium protein, unknown function
PVX_085570	0	13.7744	0	21.0067	0	0	0	27.4045	9.97004	0	7.85857	0	hypothetical protein, conserved
PVX_085575	50.2558	140.34	0	0	0	0	0	0	57.1235	88.2898	60.0199	81.3706	H/ACA ribonucleoprotein complex subunit 4, putative (CBF5)
PVX_085580	0	0	0	0	0	0	0	0	0	0	5.45016	0	hypothetical protein, conserved
PVX_085585	0	0	0	0	0	0	0	0	12.1094	0	19.0871	0	cysteine protease ATG4, putative (ATG4)
PVX_085590	6.39707	17.8538	15.8875	20.4209	2.02694	0	0	8.88017	14.5381	13.4891	20.372	10.3449	hypothetical protein, conserved
PVX_085595	119.682	0	0	0	0	0	0	0	45.3675	0	47.6494	0	hypothetical protein, conserved
PVX_085600	0	0	0	0	32.8692	0	0	0	39.225	0	0	0	hypothetical protein, conserved
PVX_085605	0	0	0	0	57.4429	0	0	251.018	68.457	63.3858	107.814	0	prefoldin subunit 2, putative
PVX_085610	47.736	33.3248	237.386	0	15.1407	0	0	0	36.1723	16.7728	9.50172	0	lysine--tRNA ligase, putative
PVX_085615	0	0	0	0	0	0	0	0	0	0	0	0	hypothetical protein, conserved
PVX_085620	0	0	0	0	0	0	0	0	0	0	0	0	hypothetical protein, conserved
PVX_085625	443.858	0	184.074	236.599	46.9571	0	113.207	411.037	140.165	311.872	235.605	119.857	glutamate dehydrogenase (NADP+), putative
PVX_085630	0	0	121.02	0	15.4374	0	0	0	18.4404	0	9.68776	0	hypothetical protein, conserved
PVX_085635	0	0	0	0	0	0	0	0	0	22.6569	0	0	hypothetical protein, conserved
PVX_085640	0	0	0	0	0	0	23.7045	0	0	10.9074	12.3554	0	hypothetical protein, conserved
PVX_085645	0	25.3234	0	0	11.5038	0	0	0	54.9782	25.4964	64.9915	58.7185	hypothetical protein, conserved
PVX_085650	0	0	0	0	0	0	0	0	0	0	0	0	hypothetical protein, conserved
PVX_085655	0	219.382	0	0	0	0	0	0	118.874	0	187.078	0	zinc finger protein, putative
PVX_085660	0	118.059	0	0	0	0	0	0	21.3566	0	44.8774	0	dimethyladenosine transferase, putative
PVX_085665	236.637	41.3054	294.286	189.13	56.3071	302.797	0	164.33	89.6635	41.5706	117.756	0	serine C-palmitoyltransferase, putative
PVX_085670	0	0	0	0	0	0	0	0	11.0766	0	5.81979	0	hypothetical protein, conserved
PVX_085675	0	19.9379	0	0	18.113	0	0	0	0	10.0384	5.68603	0	hypothetical protein, conserved
PVX_085680	58.5593	13.6219	48.4964	62.3347	12.3738	0	0	0	51.7593	61.736	58.2782	0	hypothetical protein, conserved
PVX_085685	111.205	0	0	0	35.3265	0	0	154.53	126.455	39.0613	177.096	360.73	RNA-binding protein Nova-1, putative
PVX_085690	0	422.437	0	0	0	0	0	419.844	114.458	105.827	60.0469	0	RNA polymerase small subunit, putative
PVX_085695	0	0	0	0	0	0	92.6994	0	0	0	12.0627	0	hypothetical protein, conserved
PVX_085700	102.19	0	254.479	327.093	0	0	0	0	0	35.8962	0	0	uracil-DNA glycosylase, putative (UDG)
PVX_085705	31.3929	0	0	0	0	0	0	0	35.6782	44.1251	24.9941	0	ATP-dependent protease, putative
PVX_085710	0	111.574	0	0	0	0	244.897	0	121.026	56.0443	190.628	259.2	small nuclear ribonucleoprotein-associated protein B, putative
PVX_085715	0	0	0	0	8.27173	0	0	0	39.5387	9.16926	20.7745	0	hypothetical protein, conserved
PVX_085720	0	0	170.515	219.17	108.747	0	0	0	51.9422	96.317	40.9277	222.056	mRNA capping enzyme, putative
PVX_085725	12.7415	0	0	40.6812	8.07566	0	0	0	0	4.47773	10.1442	0	hypothetical protein, conserved
PVX_085730	478.09	250.55	297.787	382.76	151.906	0	183.173	498.303	271.841	503.772	404.479	0	serine/threonine protein phosphatase PP1, putative (PP1)
PVX_085735	1026.59	1219.19	255.65	1314.39	456.465	1578.38	1572.43	855.849	1478.57	865.459	899.157	166.463	60S ribosomal protein L10, putative
PVX_085740	0	73.8376	263.22	0	0	0	0	146.857	120.178	111.375	63.1164	0	hypothetical protein, conserved
PVX_085745	0	25.6564	0	0	11.6551	0	0	51.0393	27.8505	0	21.9486	0	hypothetical protein, conserved
PVX_085750	108.773	0	0	0	0	0	166.641	302.291	123.687	76.4145	151.568	0	26S proteasome regulatory subunit RPN13, putative (RPN13)
PVX_085755	0	100.349	0	0	0	245.162	0	66.5407	108.924	50.5069	19.0747	0	DnaJ domain containing protein

Gene ID / Patient	Patient 1	Patient 2	Patient 3	Patient 4	Patient 5	Patient 6	Patient 7	Patient 8	Patient 9	Patient 10	Patient 11	Patient 12	Gene Description
PVX_085760	0	101.064	0	0	22.9651	0	110.73	0	54.8416	0	28.8076	0	diphthamide synthesis protein, putative
PVX_085765	0	0	0	0	0	0	0	0	0	0	4.89397	0	hypothetical protein, conserved
PVX_085770	0	35.6459	0	0	0	0	0	0	0	0	0	0	hypothetical protein
PVX_085775	0	0	0	0	0	0	0	0	0	0	62.6723	0	FeS assembly ATPase SufC, putative (SufC)
PVX_085780	0	31.3503	0	0	14.2431	0	0	0	0	15.7798	35.7562	72.703	30S ribosomal protein S9, putative
PVX_085785	0	0	0	0	171.908	0	0	0	0	0	0	0	hypothetical protein
PVX_085790	0	0	0	0	0	0	0	0	0	0	0	0	Sperm-specific protein Don juan, putative
PVX_085795	0	0	0	0	0	0	0	0	0	0	0	0	hypothetical protein, conserved
PVX_085800	0	0	0	0	0	0	0	0	0	0	0	0	hypothetical protein
PVX_085810	0	0	0	0	40.4699	0	0	0	0	0	25.3505	0	ubiquitin-conjugating enzyme, putative
PVX_085815	0	203.432	0	186.293	0	0	0	0	44.1601	40.9482	57.9963	0	glycylpeptide N-tetradecanoyltransferase, putative (NMT)
PVX_085820	0	18.6014	0	0	0	0	0	37.0057	20.1934	28.0976	10.61	0	AAA family ATPase, putative
PVX_085825	47.7615	33.3426	0	0	0	0	0	66.3276	36.1916	67.127	9.50679	0	deoxyhypusine synthase, putative
PVX_085830	0	208.867	0	0	31.6327	0	0	0	132.248	35.0405	69.4769	0	actin II
PVX_085835	33.4774	7.78658	27.718	0	7.07245	0	0	15.4914	0	15.6866	19.9898	0	hypothetical protein, conserved
PVX_085840	151.746	212.154	1135.24	486.394	96.5061	0	0	0	345.206	266.453	241.67	0	nuclear transport factor 2, putative (NTF2)
PVX_085845	0	0	0	130.174	0	0	0	0	0	0	0	0	hypothetical protein, conserved
PVX_085850	57.538	0	71.4944	91.8952	45.6029	0	0	0	10.8985	20.2188	51.5362	46.5526	hypothetical protein, conserved
PVX_085855	0	0	0	0	0	0	0	0	0	10.4064	5.89561	0	hypothetical protein, conserved
PVX_085860	0	0	0	0	0	0	0	0	48.0697	44.5372	0	0	S1/P1 nuclease, putative
PVX_085865	161.467	0	0	0	17.0736	0	82.312	0	0	0	107.133	0	hypothetical protein, conserved
PVX_085870	0	43.2811	154.188	0	0	0	0	86.0942	46.9753	21.7784	61.6923	100.397	hypothetical protein, conserved
PVX_085875	0	0	0	0	18.8064	0	0	0	0	0	11.799	0	GTP-binding protein, putative
PVX_085877	35.5167	24.7905	0	0	45.0465	0	0	49.317	0	12.4801	21.2081	0	conserved Plasmodium protein, unknown function
PVX_085880	0	0	0	0	5.27226	0	0	0	18.9041	5.84596	16.5553	26.9091	plastid replication-repair enzyme, putative (PREX)
PVX_085885	0	138.176	0	0	0	0	0	0	49.9888	0	131.297	0	hypothetical protein, conserved
PVX_085890	39.4982	27.571	0	0	0	0	0	54.8478	0	0	23.5858	0	rhomboid protease ROM8, putative (ROM8)
PVX_085895	97.407	0	0	0	61.8654	0	0	0	36.918	68.4339	19.3899	0	hypothetical protein, conserved
PVX_085900	14.2921	0	35.503	45.6337	0	0	0	0	0	5.02259	0	0	hypothetical protein, conserved
PVX_085905	0	71.9075	0	164.607	16.3358	0	0	71.5211	0	18.0948	102.509	83.387	ribosome maturation protein SBDS, putative
PVX_085910	0	0	0	0	0	0	0	0	0	0	0	0	hypothetical protein, conserved
PVX_085915	0	0	0	0	34.3008	0	0	0	40.9301	37.9308	42.9916	0	hypothetical protein, conserved
PVX_085920	143.284	133.37	237.513	152.643	60.595	733.129	0	198.983	253.341	234.944	199.643	77.3265	eukaryotic translation initiation factor 2 gamma subunit, putative
PVX_085925	0	0	0	0	0	0	0	0	0	0	0	0	hypothetical protein, conserved
PVX_085930	52.0384	0	0	0	0	0	0	0	0	0	0	0	rhoptry-associated protein 1, putative (RAP1)
PVX_085935	0	15.4515	0	0	4.67793	0	22.5446	30.7411	2.79598	5.18838	10.2845	11.9375	hypothetical protein, conserved
PVX_085940	0	26.3827	93.9532	0	23.9705	0	0	52.4841	42.9581	53.1245	60.1858	0	CTP synthase, putative
PVX_085945	0	0	0	0	0	0	0	0	0	0	0	0	hypothetical protein, conserved
PVX_085950	0	92.1442	0	0	0	676.322	0	0	99.9671	46.3076	131.235	213.971	hypothetical protein, conserved
PVX_085955	52.3122	36.5216	0	0	16.594	0	79.9987	0	19.8206	91.9014	41.6506	0	cytidine diphosphate-diacylglycerol synthase, putative
PVX_085960	0	0	0	0	86.0082	0	0	0	154.056	214.253	80.9258	0	RNA-binding protein, putative
PVX_085965	0	28.7518	0	0	0	0	0	0	46.8146	28.9457	16.3969	66.6731	hypothetical protein, conserved
PVX_085970	49.7911	208.495	0	79.5137	7.89182	127.293	38.0384	34.5653	103.74	52.4907	104.06	80.5607	hypothetical protein
PVX_085975	0	30.1688	0	0	0	0	0	0	65.495	30.3712	34.4093	0	hypothetical protein, conserved
PVX_085977	0	33.8176	0	77.3811	7.68018	0	0	0	18.3562	0	14.4673	0	conserved Plasmodium protein, unknown function
PVX_085980	0	0	0	0	0	0	0	0	0	0	91.2453	0	hypothetical protein, conserved
PVX_085985	90.6026	63.2852	0	289.91	57.5342	0	138.724	0	0	63.6556	126.246	293.728	DNA damage-inducible protein 1, putative (DDI1)
PVX_085990	131.269	0	0	841.03	166.882	0	201.25	0	0	92.208	78.3946	0	hypothetical protein, conserved
PVX_085995	113.077	0	0	90.2974	8.96201	0	0	0	21.4183	19.8676	22.507	0	oxidoreductase, aldo/keto reductase domain containing protein
PVX_086000	148.024	0	0	0	15.6505	0	0	0	56.0838	156.031	49.1065	0	hypothetical protein, conserved
PVX_086005	0	0	0	0	0	0	0	0	0	0	41.5379	169.188	tRNA-dihydrouridine synthase, putative
PVX_086010	0	0	0	0	0	0	0	0	0	0	0	0	hypothetical protein, conserved
PVX_086015	13.5982	3.16258	11.2567	28.9377	2.87231	0	20.7636	18.8762	13.7348	9.55799	24.3588	14.6593	hypothetical protein, conserved
PVX_086020	1581.25	1444.91	606.134	779.092	270.546	0	1304.95	1352.31	1567.67	1965.23	1234.88	197.338	40S ribosomal protein S8e, putative
PVX_086022	0	631.518	0	0	0	0	0	0	0	210.944	59.8451	0	conserved Plasmodium protein, unknown function
PVX_086025	104.862	36.5923	0	83.7327	8.31052	0	0	0	19.862	64.4856	46.9618	42.4176	DNA repair helicase, putative
PVX_086030	0	0	0	0	0	0	0	0	0	0	0	0	hypothetical protein
PVX_086035	0	13.932	0	0	0	0	0	0	45.3747	14.0312	23.8417	0	transcription factor with AP2 domain(s), putative (AP2-G2)
PVX_086040	0	0	0	0	0	0	0	0	58.9935	0	123.95	0	plasmepsin IV, putative (PM4)
PVX_086045	0	0	0	0	0	0	0	0	0	0	0	0	hypothetical protein, conserved
PVX_086050	0	0	0	0	0	0	0	0	5.65769	5.2489	0	0	hypothetical protein
PVX_086055	486.602	0	0	0	312.028	0	0	0	0	0	193.826	0	hypothetical protein, conserved

Gene ID / Patient	Patient 1	Patient 2	Patient 3	Patient 4	Patient 5	Patient 6	Patient 7	Patient 8	Patient 9	Patient 10	Patient 11	Patient 12	Gene Description
PVX_086060	0	142.68	0	654.753	0	0	0	0	0	0	0	0	hypothetical protein, conserved
PVX_086065	0	104.784	0	479.72	0	0	76.5047	277.924	151.648	140.632	89.6271	81.0062	pre-mRNA-splicing factor 38B, putative (PRP38B)
PVX_086070	0	0	0	0	0	0	0	0	0	0	28.7843	0	hypothetical protein, conserved
PVX_086075	64.0723	492.125	0	204.865	0	327.992	0	0	48.5566	270.132	89.2757	103.782	fibrillarin, putative
PVX_086080	0	16.7996	0	0	0	0	0	0	0	0	9.58258	0	multidomain scavenger receptor, putative
PVX_086085	100.695	0	0	0	31.9794	0	0	0	0	35.3714	40.0889	0	Fe-S-cluster redox enzyme, putative
PVX_086090	0	0	0	0	0	0	140.724	0	0	0	18.2945	148.981	glideosome associated protein with multiple membrane spans 3, putative (GAPM3)
PVX_086095	139.234	0	0	0	0	0	0	193.531	105.571	0	55.4348	0	vacuolar protein sorting-associated protein 29, putative (VPS29)
PVX_086100	25.8222	0	0	0	8.18575	0	0	0	9.78199	27.2221	5.13968	41.7807	ATP-dependent Clp protease, putative
PVX_086105	0	0	0	0	0	0	0	28.2527	30.8346	21.4536	24.3025	0	hypothetical protein, conserved
PVX_086110	55.115	0	0	176.181	17.4843	0	84.2925	0	62.6488	58.0945	120.677	0	hypothetical protein, conserved
PVX_086115	57.2297	0	0	182.952	0	0	0	0	21.6845	0	11.3916	0	glycerophosphodiester phosphodiesterase, putative (GDPD)
PVX_086120	43.5451	60.7694	27.0401	69.5118	6.89948	55.6392	33.2519	75.5629	45.3588	49.7351	47.6691	52.8204	hypothetical protein, conserved
PVX_086125	82.0099	0	0	0	8.66615	0	0	37.9554	10.3558	38.4245	16.3234	0	hypothetical protein, conserved
PVX_086130	158.745	110.979	0	0	50.4871	0	0	0	120.381	55.7463	31.6023	0	RNA-binding protein, putative
PVX_086135	0	0	0	0	0	0	0	0	0	0	7.5469	0	hypothetical protein
PVX_086140	125.176	87.3678	0	0	29.7651	0	0	0	47.4212	21.9931	31.1443	0	RNA-binding protein, putative
PVX_086145	27.6748	77.2597	0	0	8.77342	0	0	153.7	52.4196	97.2494	38.559	0	hypothetical protein, conserved
PVX_086150	0	0	0	0	0	0	0	0	0	15.5921	0	0	circumsporozoite protein, putative
PVX_086155	66.7484	0	332.11	0	63.5424	0	102.122	278.137	202.343	117.254	146.151	0	ribonucleoside-diphosphate reductase small chain, putative
PVX_086160	132.509	92.527	164.823	0	21.0237	0	101.365	0	25.1055	23.2773	52.7522	107.322	COBW domain-containing protein 1, putative (CBWD1)
PVX_086165	0	14.6684	104.446	0	6.66231	0	0	0	39.8107	44.3179	16.7344	0	DNA mismatch repair protein, putative
PVX_086170	33.471	23.362	0	0	0	0	51.1551	46.4756	0	35.2843	46.6353	54.1683	hypothetical protein, conserved
PVX_086175	0	0	0	0	0	0	0	0	0	0	0	0	trafficking protein particle complex subunit 1, putative (BET5)
PVX_086180	64.9502	0	0	0	10.2964	0	0	0	12.3028	45.646	32.3197	52.555	GTPase-activating protein, putative
PVX_086185	86.5609	60.4252	215.202	276.609	0	0	0	60.102	32.795	30.4152	60.3036	0	hypothetical protein, conserved
PVX_086190	0	81.9814	0	0	0	0	0	326.098	88.9492	0	93.4244	0	hypothetical protein, conserved
PVX_086195	149.482	26.0851	0	0	0	191.151	114.244	51.8922	56.6315	52.5257	52.0688	0	hypothetical protein, conserved
PVX_086200	0	0	0	0	0	0	0	0	0	0	0	0	hypothetical protein, conserved
PVX_086205	0	0	0	0	0	0	0	0	0	0	0	58.7185	adenylyl cyclase alpha, putative
PVX_086210	115.717	201.984	0	0	0	0	88.4964	0	21.9229	60.9856	46.0671	93.7007	rRNA biogenesis protein RRP5, putative (RRP5)
PVX_086215	0	0	0	0	84.7421	0	0	0	0	0	52.912	0	50S ribosomal protein L16, putative
PVX_086220	0	0	0	0	0	0	0	0	0	106.544	60.4545	0	secreted ookinete adhesive protein, putative (SOAP)
PVX_086225	0	0	0	0	0	0	0	0	0	0	0	0	hypothetical protein, conserved
PVX_086230	426.405	0	0	0	67.8979	0	0	0	0	224.55	127.34	0	cytochrome c, putative
PVX_086235	189.855	132.619	0	0	0	0	0	131.889	107.933	66.6926	18.8962	0	DNA-directed RNA polymerase II subunit RPB4, putative
PVX_086240	152.361	266.007	0	487.326	120.897	0	116.588	423.292	288.687	240.87	318.445	123.436	acid phosphatase, putative
PVX_086245	0	0	0	0	0	0	0	0	0	0	3.98696	0	nuclear formin-like protein, putative (MISFIT)
PVX_086250	0	0	0	0	32.088	0	0	0	0	0	20.1123	0	hypothetical protein, conserved
PVX_086255	0	0	0	0	0	0	0	0	0	0	0	0	selenoprotein, putative (Sel1)
PVX_086260	0	0	0	0	0	0	0	0	0	0	58.4355	0	hypothetical protein
PVX_086265	0	0	0	0	0	0	0	0	0	0	9.7514	0	hypothetical protein, conserved
PVX_086268	0	0	0	0	0	0	0	0	0	0	0	0	hypothetical protein
PVX_086272	0	0	0	0	0	0	0	0	0	0	20.3886	0	hypothetical protein
PVX_086275	0	0	0	0	0	0	0	0	0	0	26.4885	0	hypothetical protein, conserved
PVX_086280	0	0	0	0	0	0	0	0	0	0	0	0	hypothetical protein, conserved
PVX_086285	0	0	0	0	0	0	0	0	17.9901	0	18.9026	0	hypothetical protein
PVX_086290	0	38.1678	0	0	5.77836	0	0	25.3111	20.7182	6.40683	14.5151	0	hypothetical protein, conserved
PVX_086295	40.7389	85.3125	0	0	0	0	0	0	77.1717	100.203	40.5444	0	U2 snRNP-associated SURP motif-containing protein, putative (SR140)
PVX_086305	732.643	204.845	730.7	469.601	0	0	899.017	814.741	777.759	926.273	670.907	475.785	ubiquitin-40S ribosomal protein S27a, putative
PVX_086310	0	0	0	0	0	0	0	0	0	0	0	0	hypothetical protein, conserved
PVX_086315	151.957	26.5172	94.4323	0	36.1391	0	116.138	52.7516	57.5693	66.7439	136.108	0	26S proteasome regulatory subunit RPN6, putative (RPN6)
PVX_086320	0	0	0	0	0	0	0	0	0	0	0	0	hypothetical protein, conserved
PVX_086325	0	0	0	0	13.1164	0	0	0	15.6698	43.5991	8.23256	0	hypothetical protein, conserved
PVX_086330	0	95.0628	169.345	0	21.6004	0	104.146	94.5471	128.966	47.8289	162.59	220.533	exopolyphosphatase, putative
PVX_086335	0	0	0	0	0	0	0	0	0	0	13.7056	111.542	hypothetical protein, conserved
PVX_086340	79.7086	111.336	0	0	50.6028	0	0	110.728	30.2065	112.01	79.3322	0	choline kinase, putative
PVX_086345	0	0	0	0	0	0	0	0	0	0	0	0	hypothetical protein, conserved
PVX_086350	0	0	0	0	0	0	0	0	0	0	0	0	variable surface protein Vir21, putative,PIR protein
PVX_086845	0	0	0	0	0	0	0	0	0	0	0	0	VIR protein,PIR protein
PVX_086850	0	0	0	0	0	0	0	0	0	0	0	0	variable surface protein Vir35, putative,PIR protein
PVX_086855	0	54.185	0	0	12.3078	0	0	0	0	27.2764	7.72553	0	variable surface protein Vir24-like,PIR protein

Gene ID / Patient	Patient 1	Patient 2	Patient 3	Patient 4	Patient 5	Patient 6	Patient 7	Patient 8	Patient 9	Patient 10	Patient 11	Patient 12	Gene Description
PVX_086860	0	0	0	0	0	0	0	0	0	0	0	0	variable surface protein Vir24-related,PIR protein
PVX_086863	0	0	0	0	0	0	0	0	0	0	0	0	VIR protein,PIR protein
PVX_086865	0	0	0	0	0	0	0	0	0	0	0	0	VIR protein,PIR protein
PVX_086870	0	0	0	0	0	0	0	0	0	0	0	0	VIR protein, pseudogene,PIR protein, pseudogene
PVX_086875	0	0	0	0	0	0	0	0	0	0	0	0	variable surface protein Vir27-related,PIR protein
PVX_086880	0	0	0	0	0	0	0	0	0	0	0	0	variable surface protein Vir23-like,PIR protein
PVX_086890	112.252	0	0	0	0	0	0	51.9573	42.5269	39.4437	29.7909	0	variable surface protein Vir8-like,PIR protein
PVX_086893	0	0	0	0	0	0	0	0	0	0	16.529	0	VIR protein,PIR protein
PVX_086895	0	0	0	0	0	0	0	0	0	0	0	0	VIR protein,PIR protein
PVX_086900	0	186.347	0	0	25.393	0	0	37.0719	70.8033	121.974	85.032	0	Plasmodium exported protein, unknown function
PVX_086903	0	0	0	0	0	0	0	0	0	0	0	0	Plasmodium exported protein, unknown function
PVX_086905	520.729	848.333	287.807	369.932	165.203	2072.91	265.489	723.221	635.765	569.199	437.637	93.7007	Plasmodium exported protein, unknown function
PVX_086910	0	0	0	0	0	0	0	111.624	0	56.4571	0	0	Phist protein (Pf-fam-b)
PVX_086915	496.141	0	620.308	0	79.0701	0	381.744	0	94.1181	0	49.3908	0	early transcribed membrane protein (ETRAMP)
PVX_086920	0	0	0	0	0	0	0	0	0	0	0	0	hypothetical protein
PVX_086925	59.1591	0	0	0	0	0	0	0	0	0	35.3269	0	Plasmodium exported protein, unknown function
PVX_086930	23.374	0	0	0	0	0	0	0	0	0	0	0	cytoadherence linked asexual protein (CLAG), putative
PVX_086935	0	139.804	0	0	0	0	0	0	0	0	0	0	Plasmodium exported protein, unknown function
PVX_086940	128.686	134.728	0	0	20.4002	0	0	44.6706	12.1878	33.9146	51.2282	0	hypothetical protein, conserved
PVX_086945	0	0	0	0	0	0	0	0	0	0	0	0	Plasmodium exported protein, unknown function
PVX_086950	0	0	0	0	0	0	0	0	0	0	0	0	gametocyte development protein 1, putative (GDV1)
PVX_086955	0	0	0	0	0	0	0	0	0	384.446	109.238	0	phosphatidylinositol N-acetylglucosaminyltransferase subunit P, putative
PVX_086960	0	0	0	0	0	0	0	0	12.032	11.1605	25.2868	0	vacuolar protein sorting-associated protein 33, putative (VPS33)
PVX_086962	0	0	0	0	0	0	0	0	0	0	458.795	0	conserved protein, unknown function
PVX_086965	0	0	595.379	0	0	0	732.778	0	271.065	83.6041	142.256	0	U2 small nuclear ribonucleoprotein B'', putative
PVX_086970	0	0	0	0	0	0	0	0	24.7752	0	0	0	hypothetical protein, conserved
PVX_086975	0	0	0	0	0	0	0	0	0	0	21.6526	0	cAMP-dependent protein kinase catalytic subunit, putative
PVX_086980	0	0	191.201	0	24.3873	393.473	0	106.732	58.2334	80.9791	183.531	0	hypothetical protein, conserved
PVX_086985	0	0	0	0	0	0	0	0	0	0	86.5431	0	hypothetical protein, conserved
PVX_086990	212.131	296.402	0	0	0	0	162.479	294.76	40.202	298.055	168.909	0	vacuolar ATP synthase subunit e, putative
PVX_086995	195.932	0	489.212	0	62.3712	0	0	0	74.31	206.378	234.044	0	transcription factor with AP2 domain(s), putative (ApiAP2)
PVX_087000	0	0	0	0	0	0	890.358	0	219.29	0	115.052	0	hypothetical protein, conserved
PVX_087005	0	0	0	0	114.522	0	0	0	0	251.411	71.352	0	hypothetical protein, conserved
PVX_087010	61.2391	0	0	0	29.123	0	0	85.0299	57.9985	53.7979	79.2297	49.5496	DNA repair helicase RAD3, putative (RAD3)
PVX_087015	53.9196	0	0	0	0	0	0	0	20.4298	0	21.4653	0	histidine--tRNA ligase, putative
PVX_087020	0	0	0	0	71.9987	0	0	0	42.9529	159.211	135.345	0	hypothetical protein, conserved
PVX_087025	49.615	0	0	0	0	0	0	137.806	37.5966	87.1643	79.006	0	delta tubulin, putative
PVX_087030	0	142.68	509.398	654.753	64.9429	0	0	0	77.3628	71.6119	162.432	0	hypothetical protein, conserved
PVX_087035	149.062	104.097	0	476.752	70.9645	0	114.057	0	141.217	130.92	133.521	120.757	mitochondrial-processing peptidase subunit beta, putative (MAS1)
PVX_087040	0	16.8677	0	0	7.6615	0	0	0	0	25.4799	14.4321	39.1047	gamma-tubulin complex component, putative
PVX_087042	0	0	0	0	0	0	175.638	0	173.794	0	91.2702	0	conserved Plasmodium protein, unknown function
PVX_087044	0	0	0	0	0	0	0	0	28.0696	0	22.1212	0	conserved Plasmodium protein, unknown function
PVX_087045	0	0	362.963	0	46.2838	0	0	202.358	110.385	51.125	202.86	0	SGS domain containing protein
PVX_087050	0	0	0	0	14.3513	0	0	0	17.1439	0	27.0205	0	hypothetical protein, conserved
PVX_087055	0	31.8298	0	0	0	0	0	0	34.55	0	18.1514	0	mRNA processing protein, putative
PVX_087060	0	0	0	0	0	0	0	0	0	0	0	0	hypothetical protein, conserved
PVX_087065	90.4487	47.3417	112.368	0	50.1732	0	34.5471	31.3943	102.789	23.8384	103.517	0	hypothetical protein, conserved
PVX_087070	32.8084	45.7987	0	0	0	0	0	0	24.8583	11.5286	13.0606	0	hypothetical protein, conserved
PVX_087075	0	0	0	0	53.8429	0	0	0	0	59.4332	134.778	274.961	30S ribosomal protein S6, putative (RPS6)
PVX_087080	0	0	0	208.15	0	0	0	0	0	0	12.9578	0	palmitoyltransferase, putative (DHHC6)
PVX_087085	0	54.9333	0	0	24.9672	0	0	0	29.808	27.6333	31.3145	0	peptide release factor, putative
PVX_087090	61.2232	42.7477	152.286	0	58.2746	0	374.61	85.0334	208.785	64.5308	97.4921	0	M18 aspartyl aminopeptidase, putative (M18AAP)
PVX_087095	246.281	86.0488	920.499	394.387	78.2589	0	566.218	513.405	326.757	302.757	294.159	399.58	profilin, putative (PFN)
PVX_087105	0	0	0	0	0	0	0	36.8739	20.1214	0	15.8583	0	protein MAM3, putative
PVX_087107	0	0	0	0	0	0	0	0	0	0	159.945	0	conserved Plasmodium protein, unknown function
PVX_087110	0	0	0	0	0	0	0	0	0	0	29.2177	0	adenylate kinase-like protein 2, putative (AKLP2)
PVX_087115	345.282	241.163	0	276.175	0	0	264.299	119.921	65.4273	272.918	103.096	139.906	proteasome subunit beta type-6, putative
PVX_087120	48.8033	34.0704	242.701	0	15.4796	0	0	0	18.4906	17.1477	58.285	79.0156	hypothetical protein, conserved
PVX_087125	0	0	0	0	0	0	0	0	0	0	0	0	conserved protein, unknown function
PVX_087130	150.313	0	0	0	23.8539	0	0	0	56.9612	105.615	14.9603	0	hypothetical protein, conserved
PVX_087135	146.74	102.474	182.562	0	46.5714	0	112.277	305.749	55.6063	206.211	233.673	0	hypothetical protein, conserved
PVX_087140	214.826	0	0	229.011	22.7258	0	109.575	0	108.542	25.1578	71.2697	0	hypothetical protein

Gene ID / Patient	Patient 1	Patient 2	Patient 3	Patient 4	Patient 5	Patient 6	Patient 7	Patient 8	Patient 9	Patient 10	Patient 11	Patient 12	Gene Description
PVX_087143	181.618	0	0	0	0	0	836.665	0	68.8743	127.543	180.784	0	selenoprotein, putative (Sel2)
PVX_087145	36.2357	25.2926	0	0	11.4898	0	110.771	150.947	82.3671	25.4655	79.3376	0	nucleolar protein Nop52, putative
PVX_087150	116.262	81.2347	0	0	0	0	0	0	0	40.8364	0	0	hypothetical protein, conserved
PVX_087155	0	0	0	0	12.3453	0	0	0	14.7493	13.6797	46.4944	0	elongation factor Tu, putative
PVX_087160	0	0	0	0	0	0	124.446	0	0	0	32.3672	0	NifU-like protein, putative
PVX_087165	0	0	0	0	0	0	0	0	0	0	0	0	hypothetical protein
PVX_087665	92.1778	257.394	0	0	14.6176	707.419	70.4677	128.007	122.233	97.1651	91.7384	0	hypothetical protein
PVX_087670	316.579	497.463	0	0	0	810.665	0	109.944	149.963	194.63	63.0167	0	hypothetical protein, conserved
PVX_087675	253.028	574.197	157.354	202.254	60.2136	0	96.77	87.8601	143.816	200.022	100.731	102.459	hypothetical protein, conserved
PVX_087680	0	0	0	0	0	0	0	0	0	0	0	0	chitinase (CHT1)
PVX_087682	0	0	0	0	0	0	0	0	0	0	0	0	tRNA Alanine
PVX_087684	0	0	0	0	0	0	0	0	0	0	0	0	tRNA Tyrosine
PVX_087685	243.905	56.7817	0	0	0	0	124.446	0	30.8105	57.1236	97.1015	0	centrin, putative
PVX_087690	0	0	0	0	0	0	0	0	6.40134	0	16.8179	0	hypothetical protein, conserved
PVX_087695	0	187.722	0	0	0	0	0	0	0	0	0	0	hypothetical protein, conserved
PVX_087700	0	16.5196	0	75.5993	7.50335	0	0	0	0	0	23.5572	0	hypothetical protein, conserved
PVX_087705	0	0	0	0	0	0	0	545.123	0	137.222	77.9049	0	hypothetical protein, conserved
PVX_087710	48.1469	67.2237	239.432	0	15.2711	0	0	0	0	84.5857	28.7505	0	hypothetical protein, conserved
PVX_087715	0	0	0	0	0	0	0	0	4.99489	0	5.24922	0	hypothetical protein, conserved
PVX_087720	0	0	0	0	0	0	0	0	0	0	0	0	hypothetical protein, conserved
PVX_087725	238.408	83.2937	296.991	0	0	611.228	0	0	45.186	0	0	0	hypothetical protein, conserved
PVX_087730	0	0	0	0	0	0	0	36.3665	9.9223	0	5.2134	0	hypothetical protein, conserved
PVX_087735	15.0387	5.24659	37.3506	24.0043	0	0	0	20.8764	19.9369	29.0683	11.973	12.1602	hypothetical protein, conserved
PVX_087740	0	6.61583	23.5499	0	0	48.4573	0	0	21.5483	6.66422	13.2102	0	hypothetical protein, conserved
PVX_087745	0	0	0	0	0	0	0	0	3.10228	0	3.26034	0	hypothetical protein, conserved
PVX_087750	0	19.8428	0	0	0	145.385	0	39.4752	10.7704	29.9718	39.6126	0	hypothetical protein, conserved
PVX_087755	0	0	0	0	0	0	0	21.3778	11.6659	0	12.2597	0	hypothetical protein, conserved
PVX_087760	0	49.6007	0	0	22.5322	0	0	0	13.4607	12.4851	35.3609	0	WD domain, G-beta repeat domain containing protein
PVX_087765	25.1899	0	0	0	7.98518	0	0	0	0	0	0	0	calcium-dependent protein kinase 3, putative
PVX_087775	7.93751	11.0768	0	0	5.03025	0	12.1213	0	3.00653	11.1581	22.118	12.8366	E3 ubiquitin-protein ligase, putative
PVX_087780	0	0	0	0	0	0	0	33.0217	9.00976	16.7158	9.46798	0	phosphopantetheine adenylyltransferase, putative (PPAT)
PVX_087785	0	0	0	0	0	0	0	34.4216	9.3917	0	4.93465	0	hypothetical protein, conserved
PVX_087790	111.622	0	0	0	35.4591	0	171.021	155.109	0	39.2074	66.6595	0	peptide chain release factor, putative
PVX_087795	52.2932	18.248	0	0	8.28866	133.695	0	36.3027	29.7147	27.564	15.6128	42.306	methyltransferase, putative
PVX_087800	0	0	0	0	0	0	0	55.9596	0	14.1601	8.02124	0	hypothetical protein, conserved
PVX_087802	22.0821	0	0	0	0	0	0	0	33.4598	0	26.3714	0	hypothetical protein, conserved
PVX_087805	0	0	0	0	0	0	0	52.088	0	13.1809	22.3994	0	origin recognition complex subunit 2, putative (ORC2)
PVX_087810	32.1023	0	0	0	0	0	0	89.1489	24.3231	22.5611	63.8974	0	DNA replication licensing factor MCM7, putative (MCM7)
PVX_087815	11.101	0	27.5736	0	0	56.7369	0	0	4.20486	7.80245	6.62854	17.9541	inositol-phosphate phosphatase, putative
PVX_087820	30.3084	0	75.3233	0	9.60897	0	0	0	57.4092	53.2514	36.196	0	RNA helicase, putative
PVX_087825	0	7639.78	0	0	0	0	0	0	2027.45	5400.69	5218.13	0	40S ribosomal protein S29, putative
PVX_087830	0	0	0	0	0	0	0	0	0	0	0	0	cysteine-rich secretory protein, putative
PVX_087835	409.695	0	255.063	0	195.179	0	156.882	0	232.924	143.913	387.383	332.161	ATP synthase lipid-binding protein, mitochondrial precursor, putative
PVX_087840	56.1585	78.38	93.0142	0	41.532	95.6969	28.5963	77.9666	99.274	52.6268	89.4221	30.2824	importin-7, putative
PVX_087845	0	0	101.861	0	6.49741	0	31.3164	56.9196	23.2955	14.4072	24.4807	0	hypothetical protein, conserved
PVX_087851	0	0	0	0	0	0	0	864.1	0	0	123.255	0	hypothetical protein
PVX_087855	0	0	0	0	0	0	0	0	118.874	0	0	0	hypothetical protein
PVX_087860	2173.59	2305.58	0	0	353.3	0	1715.96	1524.13	414.595	2659.75	2382.69	1810.04	60S ribosomal protein L37, putative
PVX_087865	0	0	74.5056	0	0	0	0	41.6377	22.7218	5.27	0	0	hypothetical protein, conserved
PVX_087870	0	0	279.834	0	0	0	0	0	21.3163	0	33.5946	0	hypothetical protein, conserved
PVX_087875	0	0	0	0	0	0	0	0	0	0	0	0	DNA mismatch repair enzyme, putative
PVX_087876	0	0	0	0	0	0	0	0	0	0	0	0	tRNA Threonine
PVX_087877	0	0	0	0	0	0	0	0	0	0	0	0	tRNA Histidine
PVX_087878	0	0	0	0	0	0	0	0	0	0	0	0	tRNA Lysine
PVX_087879	0	0	0	0	0	0	0	0	0	0	0	0	tRNA Lysine
PVX_087880	0	0	36.9125	0	9.41835	0	0	0	5.62856	5.22188	26.618	0	hypothetical protein, conserved
PVX_087885	0	0	0	0	0	0	0	0	0	15.8036	0	0	rhoptry-associated membrane antigen, putative
PVX_087895	0	34.8112	0	159.371	31.6327	0	0	0	37.7852	35.0405	109.178	80.735	AAA family ATPase, putative
PVX_087900	0	14.6237	0	0	0	0	0	29.093	7.93791	0	8.34175	0	hypothetical protein, conserved
PVX_087905	0	0	0	0	36.6983	0	0	160.52	0	0	114.971	0	hypothetical protein, conserved
PVX_087910	86.7813	0	71.8876	0	0	0	0	0	10.9584	40.6598	28.7886	0	E3 ubiquitin-protein ligase, putative
PVX_087915	30.1858	0	75.0182	96.4244	19.1401	0	0	0	34.306	21.2144	42.0577	0	RAP protein, putative

Gene ID / Patient	Patient 1	Patient 2	Patient 3	Patient 4	Patient 5	Patient 6	Patient 7	Patient 8	Patient 9	Patient 10	Patient 11	Patient 12	Gene Description
PVX_087920	310.333	108.473	0	0	49.3448	0	238.069	431.422	411.828	326.945	401.568	0	ribosomal protein S8e, putative
PVX_087925	40.7946	0	0	0	0	0	0	0	0	0	24.3599	0	cytoskeleton associated protein, putative
PVX_087930	0	90.4097	0	0	123.347	0	0	179.805	49.0434	90.8759	77.2612	0	hypothetical protein
PVX_087935	0	1899.75	0	0	0	0	0	0	0	0	528.213	0	DNA-directed RNA polymerase II 8.2 kDa polypeptide, putative
PVX_087940	0	0	0	0	0	0	0	0	0	11.6698	6.61027	0	hypothetical protein, conserved
PVX_087945	169.214	59.0925	0	0	26.8594	433.377	129.519	117.538	96.1913	118.89	50.5247	0	EKC/KEOPS complex subunit BUD32, putative (BUD32)
PVX_087950	1853.56	1807.04	584.941	657.87	541.002	2256.84	1079.05	2205.96	1683.02	1736.9	1587.02	714.142	heat shock protein 86, putative
PVX_087955	0	39.7741	70.811	0	18.0668	0	0	39.5632	32.3832	80.1029	28.3577	0	O1, putative
PVX_087960	0	0	0	0	0	0	0	0	0	0	26.1055	0	inner membrane complex protein 1d, putative (IMC1d)
PVX_087965	0	0	0	0	60.1284	0	0	0	47.8709	44.3866	138.31	102.314	cloroquine resistance associated protein Cg8, putative
PVX_087970	436.419	456.933	271.196	116.194	46.1275	186.018	111.176	101	427.12	460.054	332.989	176.586	heat shock protein 110, putative (HSP110c)
PVX_087975	0	0	0	380.212	37.7237	0	0	164.998	0	83.4054	0	192.609	cloroquine resistance associated protein Cg3, putative,SCO1 protein homolog, mitochondrial, putative
PVX_087980	0	105.374	0	0	0	0	0	139.744	57.1878	123.745	10.0146	0	chloroquine resistance transporter, putative (CRT)
PVX_087985	59.1153	0	0	0	18.7415	0	0	82.0803	44.7894	20.773	17.6497	47.8298	cloroquine resistance associated protein cg1, putative
PVX_087990	0	0	0	0	0	0	0	0	0	39.3546	22.3033	0	glutaredoxin-like protein
PVX_087995	12.5539	26.2799	0	0	11.935	0	0	17.4278	9.51047	13.2353	17.4909	0	cloroquine resistance associated protein Cg2, putative
PVX_088000	21.2788	0	52.8692	67.9553	20.2342	0	0	29.5427	8.06061	22.4329	33.8827	68.8501	cloroquine resistance asscociatd protein Cg7, putative
PVX_088005	0	0	0	0	0	0	0	0	0	127.231	72.2199	0	hypothetical protein, conserved
PVX_088007	0	57.8732	0	0	8.76258	0	0	38.3776	52.3548	38.8517	27.5081	44.7252	ribonucleases P/MRP protein subunit POP1, putative (POP1)
PVX_088010	0	30.3006	0	138.707	13.766	0	66.3606	0	65.781	45.7556	34.5595	0	lysophospholipase, putative
PVX_088015	0	65.6818	0	0	0	0	0	130.641	0	132.125	18.7175	0	lysophospholipase, putative
PVX_088020	0	0	0	0	0	0	0	0	0	0	111.247	0	hypothetical protein, conserved
PVX_088025	0	0	0	42.4213	4.21053	0	0	0	0	4.66917	7.93345	0	hypothetical protein, conserved
PVX_088035	29.6704	0	24.5651	0	0	0	0	0	3.74618	0	5.90552	0	hypothetical protein, conserved
PVX_088040	59.5925	0	0	0	0	0	0	0	22.5801	20.9375	59.3095	0	hypothetical protein, conserved
PVX_088045	0	0	0	0	0	0	0	0	0	0	2.93659	0	hypothetical protein, conserved
PVX_088050	105.815	147.85	0	0	0	0	0	0	80.2136	0	84.2548	0	hypothetical protein, conserved
PVX_088055	66.5499	0	0	0	0	0	203.636	0	50.4351	0	39.7407	0	DNA repair protein, putative
PVX_088060	0	0	235.843	0	0	0	0	131.61	71.8027	0	75.4247	153.566	hypothetical protein, conserved
PVX_088065	651.053	454.859	540.526	1042.14	206.806	1112.42	1163.65	301.557	370.159	343.032	583.202	703.911	60S ribosomal protein L34, putative (RPL34)
PVX_088070	110.382	0	549.888	0	70.1288	0	0	153.385	41.8395	193.862	65.9194	0	hypothetical protein, conserved
PVX_088075	173.815	60.7011	0	0	27.5913	0	133.05	120.737	32.9361	0	17.2995	0	hypothetical protein, conserved
PVX_088080	130.124	0	0	0	0	0	0	0	0	0	155.421	0	50S ribosomal protein L1, mitochondrial, putative (RPL1)
PVX_088085	0	20.3671	0	0	9.25152	0	0	40.5181	44.2197	20.5089	5.80841	0	cell division cycle ATPase, putative
PVX_088090	0	0	647.416	0	0	0	0	360.194	98.2085	90.8487	206.137	421.556	hypothetical protein, conserved
PVX_088095	0	0	0	0	11.8649	0	57.1937	0	0	0	7.44773	0	hypothetical protein, conserved
PVX_088100	0	0	0	0	32.4553	0	0	141.989	38.732	71.7923	0	0	hypothetical protein, conserved
PVX_088105	0	0	315.023	269.943	26.7905	0	64.573	58.6547	64.0108	0	67.2594	136.749	hypothetical protein, conserved
PVX_088110	0	33.6299	0	0	0	0	0	0	73.0068	67.705	67.1207	0	regulator of chromosome condensation, putative
PVX_088115	267.241	186.769	0	0	0	0	409.741	371.438	253.279	469.295	372.397	0	coatomer epsilon subunit, putative
PVX_088120	52.6198	36.7365	0	168.193	83.4583	0	0	73.0779	39.8743	18.4883	94.2649	0	NSFL1 cofactor p47, putative
PVX_088125	0	0	0	0	0	0	0	0	0	0	0	0	aspartyl protease, putative
PVX_088130	44.023	0	0	70.2967	13.9542	0	0	0	33.3528	7.73509	30.6683	0	AP-3 complex subunit delta, putative
PVX_088140	0	0	0	0	17.9581	0	0	0	53.6475	69.6686	56.3744	0	hypothetical protein, conserved
PVX_088145	285.691	0	0	0	30.2399	0	145.831	0	108.277	301.074	189.565	154.385	tyrosine--tRNA ligase, putative
PVX_088150	271.891	0	135.234	347.645	17.2502	0	0	0	123.622	114.636	151.535	0	26S proteasome regulatory subunit RPN10, putative (RPN10)
PVX_088155	0	0	0	0	0	0	0	0	0	0	12.6839	0	serine protease DegP, putative (DegP)
PVX_088165	0	62.8365	0	71.889	14.2703	115.086	34.3906	62.5043	0	47.461	44.8036	0	hypothetical protein, conserved
PVX_088170	0	0	384.97	0	0	0	236.831	214.593	117.056	216.836	215.11	250.668	proteasome subunit alpha type-6, putative
PVX_088175	0	68.8695	0	631.062	93.9254	0	150.988	136.979	186.828	0	176.624	0	hypothetical protein, conserved
PVX_088180	0	466.408	0	0	141.559	0	0	618.212	84.2884	156.011	309.672	0	ras-related protein Rab-18, putative (RAB18)
PVX_088185	62.5934	43.7052	0	0	0	0	191.505	86.9377	118.589	65.9747	62.2964	0	hypothetical protein, conserved
PVX_088190	49.7496	17.3601	0	0	7.88524	0	0	0	9.42304	17.4823	9.90221	0	helicase, putative
PVX_088195	0	0	0	0	38.5968	0	186.168	0	0	85.3287	48.3609	0	hypothetical protein, conserved
PVX_088200	0	91.9141	0	210.45	20.8844	0	0	0	74.8177	23.1233	26.2015	0	hypothetical protein, conserved
PVX_088205	32.5934	90.9967	0	0	0	0	0	90.513	98.781	68.7184	123.262	52.7466	vacuolar proton translocating ATPase subunit A, putative
PVX_088210	0	0	0	0	0	0	0	0	28.1729	0	59.1948	0	protein tyrosine phosphatase, putative (PTP2)
PVX_088215	0	31.0192	0	0	4.69592	0	22.6325	0	22.4509	31.2431	35.3908	23.9674	hypothetical protein, conserved
PVX_088220	0	28.6331	101.972	0	13.008	0	0	113.921	46.6214	43.2394	57.1525	0	kinesin, putative
PVX_088225	0	53.6496	95.5283	0	24.3723	0	0	53.3634	29.1185	67.5176	45.8954	0	DnaJ domain containing protein
PVX_088230	0	0	0	0	0	0	0	0	94.9087	0	0	0	glycosyltransferase, putative
PVX_088235	18.3097	0	0	58.4684	17.4096	0	0	0	0	19.3031	32.7992	0	ferlin, putative

Gene ID / Patient	Patient 1	Patient 2	Patient 3	Patient 4	Patient 5	Patient 6	Patient 7	Patient 8	Patient 9	Patient 10	Patient 11	Patient 12	Gene Description
PVX_088240	0	0	0	0	0	0	0	0	0	0	20.2089	0	hypothetical protein, conserved
PVX_088245	0	0	0	0	0	0	0	0	0	48.605	0	0	hypothetical protein, conserved
PVX_088250	0	0	160.958	0	0	0	0	44.9616	12.2672	22.757	12.8905	0	AAA family ATPase, putative
PVX_088254	74.5308	52.0484	0	0	0	0	0	103.53	84.7299	26.1841	14.8357	0	hypothetical protein, conserved
PVX_088256	0	0	0	0	0	0	0	0	0	0	0	0	hypothetical protein, conserved
PVX_088265	43.6473	0	0	0	0	0	0	0	0	0	21.7189	0	serine/threonine protein kinase, FIKK family
PVX_088270	136.865	47.786	0	218.835	43.4323	0	0	0	0	96.1697	13.6217	0	PAP2-like protein, putative
PVX_088275	0	19.2198	0	0	0	0	0	38.2358	10.4323	9.67707	5.48131	0	hypothetical protein, conserved
PVX_088280	0	0	0	0	0	0	0	197.846	53.9622	99.9749	28.3347	0	hypothetical protein, conserved
PVX_088775	0	0	0	0	0	0	0	0	0	0	0	0	VIR protein,PIR protein
PVX_088780	0	0	0	0	0	0	0	0	0	0	27.9927	0	variable surface protein Vir24-related,PIR protein
PVX_088790	0	0	0	0	0	0	0	0	0	0	0	65.764	variable surface protein Vir21-like,PIR protein
PVX_088795	0	0	0	0	0	0	0	0	29.574	27.4166	0	0	VIR protein,PIR protein
PVX_088797	0	0	0	0	0	0	0	0	0	0	0	0	VIR protein,PIR protein
PVX_088798	0	0	0	0	0	0	0	0	0	0	0	0	VIR protein,PIR protein
PVX_088800	0	0	0	0	0	0	0	0	0	0	0	0	variable surface protein Vir4-related,PIR protein
PVX_088805	0	0	0	0	0	0	0	0	0	0	0	0	variable surface protein Vir22/24-like,PIR protein
PVX_088810	0	0	0	0	0	0	0	0	0	0	0	0	tryptophan-rich antigen (Pv-fam-a)
PVX_088815	96.775	135.204	0	0	0	0	0	0	0	33.9951	19.264	0	PST-A protein
PVX_088820	92.4751	129.189	0	0	0	0	283.198	0	35.0476	64.9705	55.2239	0	tryptophan-rich antigen (Pv-fam-a)
PVX_088825	0	0	0	0	0	0	0	0	0	0	0	0	tryptophan-rich antigen (Pv-fam-a)
PVX_088830	143.132	599.526	0	0	75.6629	488.231	0	265.027	271.147	335.277	189.933	77.244	Phist protein (Pf-fam-b)
PVX_088835	0	0	0	0	0	0	0	0	0	160.797	0	0	hypothetical protein, conserved
PVX_088840	0	0	0	0	0	0	0	0	68.6551	63.6601	84.1541	0	Phist protein (Pf-fam-b)
PVX_088845	0	0	0	0	0	0	0	0	0	0	0	0	Plasmodium exported protein, unknown function
PVX_088850	0	49.7666	0	0	0	0	0	0	0	25.0376	0	0	tryptophan-rich antigen (Pv-fam-a)
PVX_088855	375.425	262.56	0	1204.58	59.7427	964.495	0	0	284.755	131.819	186.853	0	hypothetical protein, conserved
PVX_088860	0	60.3491	0	0	0	0	0	0	0	0	0	0	sporozoite invasion-associated protein 2, putative (SIAP2)
PVX_088865	464.051	651.401	0	0	2230.52	0	1438.15	647.08	528.968	162.712	277.256	0	Senescence-associated protein, putative
PVX_088870	0	0	0	0	0	0	0	0	0	0	0	0	early transcribed membrane protein (ETRAMP)
PVX_088875	0	0	0	0	0	0	0	0	0	0	0	0	hypothetical protein
PVX_088880	73.2498	0	0	0	23.2475	0	0	0	111.03	0	72.9032	0	prolyl 4-hydroxylase subunit alpha, putative
PVX_088885	0	127.007	0	0	0	0	0	0	0	0	72.3137	0	U6 snRNA-associated Sm-like protein LSm8, putative
PVX_088890	0	0	0	0	0	0	0	250.229	68.2601	94.9072	53.7789	0	prohibitin, putative
PVX_088895	599.611	0	0	0	0	0	0	277.98	75.8057	140.348	39.7916	0	hypothetical protein, conserved
PVX_088905	18.9977	0	0	0	12.0427	0	29.0215	0	7.19639	26.7044	7.56258	0	hypothetical protein, conserved
PVX_088907	0	0	0	0	0	0	0	0	0	0	0	0	hypothetical protein
PVX_088910	0	0	0	0	0	0	0	0	0	0	0	0	GPI-anchored micronemal antigen, putative (GAMA)
PVX_088915	0	0	0	0	0	0	0	0	0	0	0	0	hypothetical protein, conserved
PVX_088920	0	0	0	0	0	0	0	0	0	0	0	0	folate transporter 1, putative (FT1)
PVX_088930	108.377	0	0	0	68.8515	0	0	0	41.0789	0	43.1479	0	translation initiation factor EIF-2b alpha subunit, putative
PVX_088935	0	0	192.193	0	24.5138	0	0	0	29.2675	27.1327	15.3735	125.144	mitochondrial inner membrane protein OXA1, putative (OXA1)
PVX_088940	0	147.844	0	0	13.4333	0	0	0	32.0963	14.8838	8.4313	0	hypothetical protein, conserved
PVX_088945	23.9752	0	0	0	0	0	36.6311	0	18.1644	25.2752	28.6322	0	leucine--tRNA ligase, putative
PVX_088950	0	0	0	0	0	0	0	50.3158	41.1835	25.4655	28.85	58.6471	hypothetical protein, conserved
PVX_088955	0	0	0	0	0	0	0	0	0	0	0	0	rhomboid protease ROM3, putative (ROM3)
PVX_088960	502.87	250.832	357.487	229.748	113.994	0	329.784	399.15	571.674	454.294	629.187	116.386	protein disulfide isomerase, putative
PVX_088965	45.5147	52.9347	0	96.8863	14.4246	0	23.1738	63.1872	17.2407	21.3266	48.3157	24.5405	SET domain protein, putative
PVX_088970	0	0	0	0	0	0	0	0	0	0	0	0	hypothetical protein, conserved
PVX_088975	59.0617	0	48.9127	0	43.6801	0	30.0756	27.3326	14.9153	55.3472	58.7782	0	hypothetical protein, conserved
PVX_088985	136.865	143.358	340.507	218.835	43.4323	0	0	95.0536	25.9314	72.1273	136.217	0	plastid 50S ribosomal protein L21, putative
PVX_088990	0	0	0	0	0	523.119	0	0	0	0	0	0	hypothetical protein, conserved
PVX_088995	0	24.0644	0	0	0	176.334	0	0	26.1226	0	6.86239	0	hypothetical protein, conserved
PVX_089000	0	0	0	0	0	0	0	0	0	0	10.4789	0	hypothetical protein
PVX_089010	0	0	0	0	8.15984	0	0	0	9.75104	9.04533	15.3703	0	translation initiation factor IF-2, putative
PVX_089015	26.7723	37.3698	0	0	0	0	0	37.1718	20.284	18.8157	21.3152	43.3192	ATP-dependent RNA helicase DBP10, putative
PVX_089020	0	0	0	0	0	0	0	0	0	0	0	0	hypothetical protein, conserved
PVX_089025	1429.38	1497.59	667.17	1143.39	340.37	915.337	410.339	1489.37	1591.29	1129.8	960.289	868.837	receptor for activated c kinase, putative
PVX_089030	0	158.39	188.125	0	23.9952	0	0	105.018	229.193	159.36	120.39	0	hypothetical protein, conserved
PVX_089035	74.0685	34.4616	61.3493	78.8552	0	0	0	0	56.117	86.7605	78.6276	0	ubiquitin conjugation factor E4 B, putative (UBE4B)
PVX_089040	120.165	83.9658	299.392	0	0	0	0	166.994	182.202	84.4127	239.207	0	dehydrodolichyl diphosphate synthetase, putative
PVX_089045	0	0	0	0	15.7199	0	0	0	0	17.4135	39.4592	0	hypothetical protein, conserved

Gene ID / Patient	Patient 1	Patient 2	Patient 3	Patient 4	Patient 5	Patient 6	Patient 7	Patient 8	Patient 9	Patient 10	Patient 11	Patient 12	Gene Description
PVX_089050	0	0	115.485	0	0	0	0	64.5023	17.5979	0	0	75.1967	alpha/beta hydrolase, putative
PVX_089055	10.4843	14.63	0	0	16.6088	17.8577	10.6722	9.70236	31.7686	17.1951	28.519	22.6043	E3 ubiquitin-protein ligase, putative
PVX_089060	0	23.7893	0	0	0	0	0	47.3254	12.9121	11.9764	13.568	0	hypothetical protein, conserved
PVX_089065	0	28.0794	0	0	0	0	61.4924	0	45.7201	56.5385	24.0205	0	hypothetical protein, conserved
PVX_089070	0	0	0	0	0	0	0	0	21.5604	0	0	0	hypothetical protein, conserved
PVX_089075	0	0	0	0	0	0	0	0	0	0	0	0	hypothetical protein, conserved
PVX_089080	0	0	0	0	0	0	0	0	0	0	272.491	0	cytochrome c oxidase assembly protein, putative
PVX_089085	0	29.1954	0	0	26.5273	213.959	63.9384	0	31.6912	14.696	49.9494	0	protein KRI1, putative (KRI1)
PVX_089087	0	0	0	0	0	0	0	0	0	199.393	226.655	0	conserved Plasmodium protein, unknown function
PVX_089090	179.793	0	448.712	576.75	0	0	0	0	68.1816	0	71.5871	0	hypothetical protein, conserved
PVX_089095	0	0	0	0	0	0	0	0	0	94.1107	0	0	translation initiation factor IF-3, putative
PVX_089105	0	0	0	0	0	0	0	0	0	0	0	0	hypothetical protein, conserved
PVX_089110	47.8398	0	0	38.1849	3.79007	0	0	0	4.53025	4.20307	19.0439	58.0316	hypothetical protein, conserved
PVX_089115	0	42.9182	0	0	0	0	0	0	46.5902	32.4118	6.11972	0	hypothetical protein, conserved
PVX_089120	60.718	0	0	0	6.41509	0	0	28.0993	7.6668	7.11239	16.1137	0	hypothetical protein, conserved
PVX_089125	0	37.9189	0	173.611	0	0	0	0	41.1573	57.2485	21.6216	0	hypothetical protein, conserved
PVX_089130	0	64.1961	0	0	0	0	0	0	34.8315	0	36.5891	0	Fe-S cluster assembly protein DRE2, putative (DRE2)
PVX_089135	97.407	0	0	0	0	0	149.175	0	36.918	0	77.5594	0	hypothetical protein, conserved
PVX_089140	0	0	157.801	202.829	20.1282	0	0	0	48.0746	44.5753	63.1355	0	nucleoside transporter 2, putative (NT2)
PVX_089145	0	0	0	140.137	13.9078	0	0	0	16.6145	15.4088	17.4576	0	GTP-binding protein, putative
PVX_089150	49.4063	0	122.766	0	31.3232	0	37.7442	68.5962	28.074	8.68084	108.173	39.9688	hypothetical protein, conserved
PVX_089155	0	0	0	0	0	0	0	0	26.4783	49.0982	27.8178	113.2	3'-5' exonuclease domain containing protein
PVX_089160	0	0	0	0	0	0	0	0	0	14.1984	0	0	hypothetical protein, conserved
PVX_089165	232.524	0	0	0	73.8747	0	0	161.564	88.1397	81.6728	115.718	0	dicarboxylate/tricarboxylate carrier, putative (DTC)
PVX_089170	153.851	80.5436	95.6105	0	0	0	58.7934	106.819	131.146	148.666	122.493	0	DnaJ protein, putative
PVX_089175	755.991	265.007	0	0	0	0	584.112	0	143.54	265.19	225.815	0	hypothetical protein, conserved
PVX_089180	79.4962	0	0	0	0	0	0	0	0	27.9278	0	0	lipoate-protein ligase B, putative
PVX_089185	0	0	0	0	0	0	0	0	0	0	0	0	hypothetical protein, conserved
PVX_089195	0	0	0	214.239	0	0	0	0	0	0	0	0	alpha/beta hydrolase, putative
PVX_089200	77.8076	40.7236	0	0	6.16538	0	0	27.0058	44.2108	20.5071	58.0755	0	histone acetyltransferase GCN5, putative
PVX_089205	0	92.554	0	0	0	0	203.044	0	50.2057	186.052	131.818	0	clustered-asparagine-rich protein, putative
PVX_089210	113.318	0	0	0	35.9994	0	0	0	0	119.408	112.787	0	hypothetical protein, conserved
PVX_089215	0	18.0999	0	0	0	0	0	0	0	18.2269	20.6481	0	hypothetical protein, conserved
PVX_089220	0	21.1108	0	0	0	0	0	0	11.4585	0	0	0	hypothetical protein, conserved
PVX_089225	115.961	81.0239	288.884	0	36.8414	0	177.694	322.29	219.778	407.306	69.2511	188.102	U5 snRNP-specific 40 kDa protein, putative
PVX_089230	0	0	0	0	0	0	0	0	0	0	0	0	hypothetical protein, conserved
PVX_089235	0	0	107.134	0	40.9993	220.458	65.8807	59.8414	48.9793	90.8503	60.0422	0	protein transport protein SEC23, putative
PVX_089240	0	0	0	0	0	0	0	0	0	0	0	0	dynein light chain 1, putative (DLC1)
PVX_089245	0	0	0	0	22.2095	0	0	0	0	0	27.8613	0	hypothetical protein, conserved
PVX_089250	244.012	170.778	0	0	0	0	0	339.52	185.152	171.306	145.748	397.251	small nuclear ribonucleoprotein G, putative,SmG, putative
PVX_089255	0	0	0	0	13.7212	0	0	0	0	7.60602	21.5403	0	hypothetical protein, conserved
PVX_089260	0	0	0	0	102.619	0	247.559	0	0	56.65	96.3452	0	mediator of RNA polymerase II transcription subunit 7, putative (MED7)
PVX_089265	0	0	0	0	20.2143	0	0	88.4859	48.28	89.5312	12.681	0	hypothetical protein, conserved
PVX_089270	305.565	0	0	0	0	0	234.393	212.392	57.9277	214.617	121.66	0	hypothetical protein, conserved
PVX_089275	197.227	0	0	0	0	1013.67	0	0	224.406	0	39.2653	0	protein transport protein SEC61 subunit beta, putative
PVX_089280	1608.44	749.84	1784.04	573.278	56.8668	0	1097.63	1242.53	881.044	1004.06	676.002	1161.65	60S ribosomal protein L22, putative
PVX_089285	51.7975	36.162	0	0	32.861	0	0	0	19.6255	18.1995	20.6204	0	hypothetical protein, conserved
PVX_089290	0	0	0	0	32.7932	0	0	0	0	0	20.5533	0	ribosomal RNA small subunit methyltransferase NEP1, putative (NEP1)
PVX_089292	0	0	0	0	0	0	0	0	358.201	0	187.742	0	conserved Plasmodium protein, unknown function
PVX_089295	0	0	0	0	38.2347	0	61.4371	111.618	45.679	42.3658	23.9989	0	ATP-dependent RNA helicase prh1, putative
PVX_089300	0	0	0	0	0	0	0	0	0	0	157.061	0	hypothetical protein, conserved
PVX_089305	0	28.3987	0	0	0	0	0	0	61.6531	0	0	0	protein kinase, putative
PVX_089310	0	0	0	0	0	0	0	0	0	23.1744	0	0	hypothetical protein, conserved
PVX_089315	20.8281	0	0	0	33.009	0	0	28.9169	0	14.6386	29.0194	0	hypothetical protein, conserved
PVX_089320	59.2523	20.6774	0	94.6354	9.3925	0	45.2733	123.406	0	31.2317	58.9686	0	hypothetical protein, conserved
PVX_089325	0	0	0	96.0354	9.53144	0	45.9431	0	34.1677	31.6933	77.7924	48.6499	2-oxoglutarate dehydrogenase E1 component, mitochondrial precursor, putative
PVX_089330	0	0	0	0	0	0	0	0	0	0	13.5595	0	hypothetical protein, conserved
PVX_089335	37.355	52.1485	0	0	0	0	57.0981	51.8705	14.152	26.2519	7.4353	0	hypothetical protein, conserved
PVX_089340	0	0	0	0	23.4785	0	0	0	0	25.9893	0	0	hypothetical protein, conserved
PVX_089345	63.3018	44.2002	0	0	20.0854	0	0	0	0	22.2404	0	0	hypothetical protein, conserved
PVX_089355	0	0	120.364	0	15.3538	0	0	67.2249	0	17.0086	28.9061	0	phosphatidylglycerophosphate synthase, putative
PVX_089360	110.792	0	0	0	35.195	0	0	0	41.995	77.8327	44.1095	0	hypothetical protein, conserved

Gene ID / Patient	Patient 1	Patient 2	Patient 3	Patient 4	Patient 5	Patient 6	Patient 7	Patient 8	Patient 9	Patient 10	Patient 11	Patient 12	Gene Description
PVX_089365	0	11.1028	39.5255	0	0	0	0	22.0886	24.1075	16.774	53.8362	0	helicase, putative
PVX_089370	0	269.356	0	0	61.2926	0	0	0	146.058	135.219	76.6714	313.031	U6 snRNA-associated Sm-like protein LSm3, putative
PVX_089375	0	0	0	0	0	0	0	0	0	0	4.27088	0	hypothetical protein, conserved
PVX_089380	0	0	0	0	0	0	0	0	0	0	0	0	hypothetical protein, conserved
PVX_089385	0	0	0	0	0	0	0	0	0	0	0	0	hypothetical protein, conserved
PVX_089390	90.6026	0	0	0	0	0	0	125.875	0	0	36.0704	0	hypothetical protein, conserved
PVX_089395	0	0	0	0	0	0	0	0	0	0	0	0	perforin-like protein 4 (PLP4)
PVX_089400	140.837	24.5758	0	0	0	0	0	48.89	13.3389	12.3721	14.0164	0	hypothetical protein, conserved
PVX_089405	0	0	0	0	0	0	0	0	0	0	9.9753	0	perforin-like protein 5 (PLP5)
PVX_089410	0	0	0	0	0	0	0	0	50.4297	0	0	0	hypothetical protein, conserved
PVX_089415	21.2991	0	0	0	6.75115	0	0	29.5709	16.1366	7.48475	38.1543	0	hypothetical protein, conserved
PVX_089425	1280.7	1379.21	1637.17	935.257	603.339	0	727.083	965.384	1580.33	1504.35	1638.74	1066.02	heat shock 70 kDa protein, putative
PVX_089430	0	57.7801	0	0	26.2623	0	126.638	0	62.704	87.1898	49.4036	0	U3 small nucleolar ribonucleoprotein protein, putative
PVX_089435	0	44.3888	0	0	40.3423	0	0	88.2972	120.443	22.3351	88.5781	0	RAD protein (Pv-fam-e)
PVX_089440	0	0	0	0	0	0	0	0	0	0	0	0	hypothetical protein
PVX_089445	94.0297	65.6818	0	300.905	0	0	0	0	35.6371	0	37.435	0	RAD protein (Pv-fam-e)
PVX_089450	0	0	0	0	0	0	0	0	0	0	0	0	RAD protein (Pv-fam-e)
PVX_089455	0	0	0	0	0	0	131.894	0	32.6506	0	0	0	RAD protein (Pv-fam-e)
PVX_089460	0	0	0	0	0	0	0	0	0	0	0	0	RAD protein (Pv-fam-e)
PVX_089465	77.0328	0	0	0	0	0	0	0	0	0	15.3338	0	RAD protein (Pv-fam-e)
PVX_089467	0	0	0	0	0	0	0	0	0	0	0	0	RAD protein (Pv-fam-e)
PVX_089470	0	81.0239	0	0	0	0	0	0	0	0	23.0837	0	RAD protein (Pv-fam-e)
PVX_089473	0	86.406	0	0	0	0	0	0	0	0	0	0	RAD protein (Pv-fam-e)
PVX_089475	0	0	0	0	0	0	0	0	0	0	0	0	RAD protein (Pv-fam-e)
PVX_089480	28.8153	20.1113	0	0	0	0	44.0331	0	21.8322	70.8798	57.3547	93.2552	hypothetical protein, conserved
PVX_089485	67.5546	94.3446	0	0	0	0	0	93.833	25.5984	47.468	80.6814	0	hypothetical protein, conserved
PVX_089490	0	114.858	409.819	0	52.2551	0	252.127	0	62.2924	57.689	0	0	hypothetical protein, conserved
PVX_089495	0	0	0	910.753	0	0	0	197.531	0	0	113.159	0	hypothetical protein, conserved
PVX_089500	0	0	0	0	0	0	0	0	0	0	0	0	dynactin subunit 6, putative
PVX_089505	89.7846	62.7131	0	0	171.041	0	0	0	442.354	599.271	428.936	145.534	14-3-3 protein, putative (14-3-3I)
PVX_089510	0	0	0	0	0	0	0	0	0	0	0	0	D13 protein, putative
PVX_089515	0	57.3555	0	0	0	0	0	0	0	28.85	49.0409	0	conserved protein, unknown function
PVX_089520	0	0	0	0	0	0	0	0	0	0	177.296	0	high mobility group protein B2, putative (HMGB2)
PVX_089525	0	0	0	0	0	0	0	0	0	0	0	0	hypothetical protein, conserved
PVX_089530	0	0	0	0	0	0	0	0	0	9.60613	0	0	rhoptry neck protein 5, putative (RON5)
PVX_089535	0	0	0	0	0	0	0	0	0	10.7928	12.2268	0	hypothetical protein, conserved
PVX_089540	347.037	0	432.975	0	110.412	0	266.382	0	197.398	182.79	414.529	0	protein kinase C inhibitor, putative,14 kDa zinc-binding protein, putative
PVX_089542	0	0	0	0	0	0	96.0219	0	23.7843	0	37.4827	101.667	conserved Plasmodium protein, unknown function
PVX_089545	34.437	0	0	0	0	0	0	0	0	0	0	0	hypothetical protein
PVX_089550	0	32.5262	0	0	0	0	0	0	0	49.1136	18.5483	0	hypothetical protein, conserved
PVX_089555	0	29.9949	0	0	27.254	0	0	0	16.2794	15.0981	76.9747	0	tRNA modification GTPase trmE, putative
PVX_089560	87.932	61.4176	218.885	0	27.9173	0	0	122.162	33.3247	0	0	0	ubiquitin-activating enzyme E1C, putative
PVX_089565	102.073	0	0	0	32.4182	0	0	0	0	35.8552	101.594	0	adenylate kinase 2, putative (AK2)
PVX_089570	0	0	0	0	0	0	0	0	0	0	21.189	0	meiotic recombination protein DMC1, putative (DMC1)
PVX_089575	0	0	0	0	0	0	0	0	159.015	0	83.368	0	trafficking protein particle complex subunit 2-like protein, putative (TRAPPC2L)
PVX_089580	60.6169	126.923	75.3233	0	67.2628	0	0	210.414	206.673	170.404	205.111	0	chaperone protein ClpB1, putative (ClpB1)
PVX_089585	0	0	0	0	0	0	0	0	0	0	0	466.395	heat shock protein, putative
PVX_089590	0	0	0	0	45.5403	0	219.695	0	54.3081	0	142.581	0	hypothetical protein, conserved
PVX_089592	0	0	0	0	0	0	0	0	0	0	7.44773	0	hypothetical protein, conserved
PVX_089595	0	0	0	0	65.3922	0	0	0	0	72.1044	163.551	333.984	vacuolar protein sorting-associated protein 2, putative (VPS2)
PVX_089600	0	0	0	0	72.4063	0	0	0	172.436	0	45.2502	0	phosphopantothenoylcysteine decarboxylase, putative
PVX_089605	76.2453	106.494	0	0	0	390.449	0	105.913	202.253	53.5723	30.3539	0	WD domain, G-beta repeat domain containing protein
PVX_089610	0	0	0	0	0	0	0	0	0	0	10.0428	0	dihydrolipoamide dehydrogenase, putative
PVX_089615	22.2579	15.5333	0	0	0	0	0	30.9023	8.43154	0	13.2906	0	vacuolar protein sorting-associated protein 9, putative (VPS9)
PVX_089620	0	0	0	0	29.1217	0	140.435	0	0	32.2191	73.0282	0	ubiquitin, putative
PVX_089625	94.6262	330.495	235.593	0	60.0952	0	144.903	0	179.317	398.887	339.052	460.21	eukaryotic translation initiation factor 3 subunit 4, putative
PVX_089630	0	0	0	0	0	0	0	0	0	0	13.3963	109.02	hypothetical protein, conserved
PVX_089632	0	0	752.671	0	0	0	0	418.43	114.073	210.944	59.8451	0	conserved Plasmodium protein, unknown function
PVX_089635	89.1584	0	0	285.278	0	0	0	0	304.108	93.9621	159.729	0	phosphoglycerate mutase, putative
PVX_089640	312.707	109.194	194.549	500.125	124.071	0	119.651	325.795	296.257	164.787	217.861	0	26S proteasome AAA-ATPase subunit RPT3, putative
PVX_089645	45.0677	0	0	0	14.2934	0	0	0	17.0748	31.6709	71.7645	0	hypothetical protein, conserved
PVX_089650	0	0	0	0	0	0	0	0	100.859	46.7203	26.4812	0	50S ribosomal protein L10, putative

Gene ID / Patient	Patient 1	Patient 2	Patient 3	Patient 4	Patient 5	Patient 6	Patient 7	Patient 8	Patient 9	Patient 10	Patient 11	Patient 12	Gene Description
PVX_089655	0	25.3234	0	0	11.5038	0	0	50.377	68.7227	89.2375	72.2128	117.437	ubiquitin carboxyl-terminal hydrolase 13, putative (USP13)
PVX_089660	0	0	0	151.279	0	0	0	32.8818	0	8.32251	56.5673	76.6353	chromosome associated protein, putative
PVX_089665	0	0	0	0	6.7001	0	0	29.3473	8.00729	0	0	0	hypothetical protein, conserved
PVX_089667	0	0	0	0	0	0	0	0	0	0	0	0	calmodulin-like protein
PVX_089670	0	0	0	0	0	0	0	0	0	0	0	0	Rab5-interacting protein, putative
PVX_089675	88.1919	0	0	0	0	0	0	0	33.4233	61.9627	35.1106	0	hypothetical protein, conserved
PVX_089680	0	0	0	0	0	0	144.291	0	0	0	18.7569	0	RNA-binding protein, putative
PVX_089685	0	0	0	0	0	0	0	0	0	0	0	0	hypothetical protein, conserved
PVX_089690	0	36.5858	0	0	0	0	80.1394	0	0	36.8251	31.2928	0	GTP-binding protein, putative
PVX_089695	0	0	0	0	0	0	0	0	0	0	0	0	hypothetical protein, conserved
PVX_089700	0	0	0	0	0	0	0	0	0	0	0	0	armadillo-domain containing rhoptry protein, putative (ARO)
PVX_089705	97.09	339.111	0	0	30.8318	0	0	0	73.5955	170.528	96.6337	0	arsenical pump-driving ATPase, putative
PVX_089710	0	0	177.459	0	22.635	0	0	0	0	50.1152	56.7886	0	hypothetical protein, conserved
PVX_089715	0	0	0	0	0	0	25.5389	0	12.6663	17.6263	19.9666	27.0449	hypothetical protein, conserved
PVX_089720	0	0	0	0	0	0	0	0	0	0	19.7098	32.0392	protein kinase domain containing protein
PVX_089725	211.19	147.744	0	0	67.2536	0	0	0	160.211	148.288	210.229	0	coatomer subunit zeta, putative
PVX_089730	0	179.262	0	0	0	0	0	0	97.2426	45.0475	127.661	0	nuclear cap-binding protein, putative
PVX_089735	416.851	72.8044	0	0	33.0995	0	159.632	144.803	78.9985	36.6064	62.2343	168.99	adenylate kinase 1, putative
PVX_089740	147.223	0	0	0	0	0	0	102.252	83.684	77.5833	102.568	0	hypothetical protein, conserved
PVX_089745	44.9771	0	0	0	0	0	0	0	0	0	8.95252	0	hypothetical protein, conserved
PVX_089750	928.935	648.651	825.341	636.51	147.385	339.688	203.031	829.46	578.283	676.043	594.342	0	60S ribosomal protein L15-1, putative
PVX_089755	0	0	0	0	0	0	0	0	54.7088	25.3607	0	116.951	RNA-binding protein, putative
PVX_089760	0	0	0	0	0	0	0	0	0	14.084	0	64.8794	hypothetical protein
PVX_089765	99.0653	172.899	0	0	0	0	151.483	206.365	206.438	191.443	157.75	80.198	RAD protein (Pv-fam-e)
PVX_089770	0	0	0	0	33.8908	0	0	0	0	0	0	0	hypothetical protein
PVX_089775	0	0	0	0	37.8749	611.228	0	0	90.3721	83.7385	47.459	0	RAD protein (Pv-fam-e)
PVX_089780	0	0	0	0	0	0	0	0	162.826	0	170.722	701.473	hypothetical protein
PVX_089785	0	0	0	0	0	0	0	0	0	0	0	0	RAD protein (Pv-fam-e)
PVX_089790	0	68.4918	0	0	0	0	0	0	37.1608	34.4418	19.5173	0	RAD protein (Pv-fam-e)
PVX_089795	0	0	0	0	0	0	0	0	44.6503	0	0	0	RAD protein (Pv-fam-e)
PVX_089800	0	0	0	0	0	0	0	0	0	0	0	0	RAD protein (Pv-fam-e)
PVX_089805	0	527.074	0	0	0	386.489	0	0	114.403	26.5152	0	0	RAD protein (Pv-fam-e)
PVX_089810	0	234.549	0	0	0	573.653	0	0	127.246	235.832	111.377	181.496	RAD protein (Pv-fam-e)
PVX_089815	0	0	0	0	0	0	0	0	0	0	0	0	RAD protein (Pv-fam-e)
PVX_089820	0	0	0	0	0	0	0	0	0	0	0	0	RAD protein (Pv-fam-e)
PVX_089825	0	0	0	0	0	0	0	0	0	40.573	0	0	RAD protein (Pv-fam-e)
PVX_089830	0	0	0	0	0	0	0	267.216	72.8722	0	0	0	RAD protein (Pv-fam-e)
PVX_089835	0	0	0	0	0	0	0	0	0	0	0	0	RAD protein (Pv-fam-e)
PVX_089840	108.509	151.62	0	0	0	0	0	0	575.803	304.917	43.2002	0	RAD protein (Pv-fam-e)
PVX_089845	0	0	0	0	0	0	0	0	0	61.5259	34.8823	284.699	RAD protein (Pv-fam-e)
PVX_089850	0	226.946	0	0	20.6261	332.766	99.4465	0	49.2623	91.3515	25.8779	0	RAD protein (Pv-fam-e)
PVX_089852	0	0	0	0	0	0	0	0	0	0	0	0	RAD protein (Pv-fam-e)
PVX_089855	0	0	0	0	0	0	0	0	0	0	0	0	RAD protein (Pv-fam-e)
PVX_089860	0	0	0	0	0	0	0	0	0	63.3858	0	0	RAD protein (Pv-fam-e)
PVX_089863	0	126.237	0	0	0	0	0	0	0	0	0	0	RAD protein (Pv-fam-e)
PVX_089865	92.6666	0	0	0	0	0	0	0	70.2404	65.105	92.2305	0	RAD protein (Pv-fam-e)
PVX_089867	0	0	0	0	0	0	0	0	0	63.3858	0	0	RAD protein (Pv-fam-e)
PVX_089870	0	126.237	0	0	0	0	0	0	0	0	0	0	RAD protein (Pv-fam-e)
PVX_089875	0	0	0	0	0	0	0	0	0	0	0	0	RAD protein (Pv-fam-e)
PVX_089880	0	45.9571	0	0	0	0	0	0	0	46.2466	13.1007	0	RAD protein (Pv-fam-e)
PVX_089885	0	0	0	0	0	0	0	0	0	127.543	0	0	RAD protein (Pv-fam-e)
PVX_089890	0	0	0	0	0	0	0	0	0	0	0	0	RAD protein (Pv-fam-e)
PVX_089895	0	35.5242	126.533	0	16.1405	0	0	0	38.5589	17.8788	70.8991	0	glutamyl-tRNA(Gln) amidotransferase subunit A, putative
PVX_089900	0	137.951	0	0	0	0	0	0	74.802	138.494	39.2653	0	hypothetical protein, conserved
PVX_089905	0	0	0	76.6337	0	0	0	0	0	0	38.207	0	DNA helicase MCM9, putative (MCM9)
PVX_089910	24.2875	33.9002	0	77.5703	30.7958	0	0	33.7209	18.401	17.0696	29.0052	0	histone acetyltransferase, putative
PVX_089915	0	0	0	0	43.4467	0	0	189.979	0	48.0039	136.047	0	repressor of RNA polymerase III transcription MAF1, putative
PVX_089920	0	66.6636	0	0	30.3045	0	0	0	72.339	33.524	132.979	0	prohibitin-like protein, putative
PVX_089925	0	0	0	0	0	0	0	1792.25	0	445.746	0	0	hypothetical protein, conserved
PVX_089930	240.085	0	0	0	76.5095	0	369.36	0	0	84.2762	430.203	0	small GTP-binding protein sar1, putative
PVX_089935	56.4649	9.85036	0	0	4.47363	0	0	19.5971	21.3884	34.7256	56.1934	22.8328	hypothetical protein, conserved
PVX_089940	0	61.0573	0	0	0	0	0	0	0	0	0	0	hypothetical protein, conserved

Gene ID / Patient	Patient 1	Patient 2	Patient 3	Patient 4	Patient 5	Patient 6	Patient 7	Patient 8	Patient 9	Patient 10	Patient 11	Patient 12	Gene Description
PVX_089945	0	0	0	0	0	0	0	0	0	0	0	0	mRNA-binding protein PUF2, putative (PUF2)
PVX_089950	53.5326	37.3742	133.127	0	33.9632	0	81.8681	0	141.982	112.854	127.867	0	bifunctional dihydrofolate reductase-thymidylate synthase, putative (DHFR-TS)
PVX_089955	0	32.4249	115.485	0	44.1947	0	71.0171	129.005	35.1957	114.242	64.7169	0	hypothetical protein, conserved
PVX_089960	16.6616	0	0	0	3.51945	0	0	0	2.10362	0	8.84334	0	hypothetical protein, conserved
PVX_089970	89.7846	62.7131	447.017	0	114.027	0	137.468	0	34.0272	94.6217	89.3617	145.534	hypothetical protein, conserved
PVX_089972	0	0	0	0	0	0	0	0	0	0	7.0498	0	conserved Plasmodium protein, unknown function
PVX_089975	0	0	0	226.469	0	0	0	0	0	0	0	0	hypothetical protein, conserved
PVX_089980	0	38.1973	0	0	17.3558	0	0	0	41.4594	96.114	32.6703	0	cdc2-related protein kinase 1, putative
PVX_089985	39.5681	0	0	0	12.5475	0	0	0	59.9626	13.9034	15.7517	0	hypothetical protein, conserved
PVX_089990	13.6613	0	0	21.8053	2.16434	0	0	0	5.17451	4.8011	5.43819	0	hypothetical protein, conserved
PVX_089995	0	0	0	0	0	0	0	35.4835	29.0442	8.98076	10.1737	0	hypothetical protein, conserved
PVX_090000	166.473	77.4849	138.005	177.385	88.0184	0	84.8684	77.0675	252.305	467.925	209.863	0	hypothetical protein, conserved
PVX_090005	169.194	59.0536	0	0	13.4142	0	0	0	32.0508	89.176	50.5161	0	hypothetical protein
PVX_090010	0	0	0	0	0	0	0	0	65.2922	0	102.834	0	hypothetical protein, conserved
PVX_090015	0	306.765	0	937.661	186.046	0	0	203.352	221.853	256.878	378.589	0	trafficking protein particle complex subunit 3, putative (BET3)
PVX_090020	0	0	0	0	0	0	0	0	0	0	3.63766	0	hypothetical protein, conserved
PVX_090025	0	50.7785	0	0	23.0772	0	0	0	82.6636	25.546	72.3701	117.808	RNA-binding protein NOB1, putative
PVX_090030	0	0	0	0	0	0	0	0	0	39.6024	0	0	hypothetical protein, conserved
PVX_090035	0	0	0	0	0	0	0	0	0	0	12.4072	0	hypothetical protein, conserved
PVX_090040	0	0	0	0	25.1666	0	0	0	0	0	0	0	hypothetical protein, conserved
PVX_090045	0	66.7348	0	0	60.6739	0	0	0	0	134.239	76.0689	0	hypothetical protein, conserved
PVX_090050	0	302.156	0	0	551.593	0	0	0	490.828	151.039	343.084	0	Got1 domain containing protein
PVX_090055	0	0	0	0	35.5926	0	0	0	0	39.3546	22.3033	0	hypothetical protein, conserved
PVX_090060	13.5028	9.42225	0	0	8.55833	0	0	18.7454	0	14.2357	29.5634	0	hypothetical protein, conserved
PVX_090065	0	0	0	0	0	0	0	0	0	0	11.7523	0	hypothetical protein, conserved
PVX_090070	159.453	111.323	528.709	509.681	33.7208	271.996	325.135	295.264	120.831	149.4	285.65	172.131	ran binding protein 1, putative
PVX_090075	0	0	0	0	0	0	0	0	0	35.4911	20.1123	0	hypothetical protein, conserved
PVX_090080	0	300.006	0	274.846	0	0	0	0	65.1131	60.3574	119.702	0	ribosomal protein L7Ae-related protein, putative
PVX_090085	6.93707	4.84027	0	22.1451	2.19807	0	10.5932	19.2597	7.8827	4.8759	13.8073	0	phosphatidylinositol 4-kinase, putative
PVX_090090	0	6.22595	0	28.4857	0	0	0	0	23.6583	0	7.10386	0	CW-type zinc finger domain-containing protein
PVX_090095	51.7675	108.423	128.732	496.394	16.421	0	158.329	71.8936	58.8424	36.3779	82.4338	83.8217	serine/threonine protein kinase RIO2, putative (RIO2)
PVX_090100	0	0	0	67.4578	0	0	0	0	0	0	0	0	hypothetical protein, conserved
PVX_090105	67.1491	0	0	0	0	0	102.737	0	0	0	0	0	holo-(acyl-carrier protein) synthase, putative
PVX_090110	18.1441	12.6602	45.0652	0	5.7494	0	0	0	20.6177	12.7529	28.8907	0	transcription factor with AP2 domain(s), putative (ApiAP2)
PVX_090115	143.936	0	358.86	0	0	0	0	200.077	109.141	101.1	28.6537	233.667	ribosome-recycling factor, putative (RRF2)
PVX_090120	0	0	0	0	0	0	0	0	0	0	0	0	hypothetical protein, conserved
PVX_090125	0	0	0	0	22.9651	0	0	0	54.8416	25.4222	100.827	0	hypothetical protein
PVX_090130	0	0	0	0	0	0	0	0	0	0	12.3554	0	hypothetical protein, conserved
PVX_090135	0	0	0	0	0	0	0	0	0	0	0	0	hypothetical protein, conserved
PVX_090137	0	0	0	0	0	0	0	0	0	0	0	0	conserved Plasmodium protein, unknown function
PVX_090140	0	0	0	0	0	0	179.091	0	0	0	116.323	0	steroid dehydrogenase, putative
PVX_090145	51.8876	0	0	0	0	0	0	0	0	18.2311	51.6406	0	hypothetical protein, conserved
PVX_090150	34.3736	43.1709	17.074	0	10.8915	70.2642	0	47.7164	28.6435	12.0802	27.3664	0	erythrocyte membrane-associated antigen, putative
PVX_090155	311.347	54.3594	0	248.973	0	0	238.258	540.629	29.4968	54.6901	340.864	126.126	tubulin alpha chain, putative
PVX_090160	1392.88	1150.34	631.094	811.174	80.4804	2597.7	582.306	527.952	432.014	845.01	983.074	616.391	40S ribosomal protein S19, putative (RPS19)
PVX_090165	21.2202	22.2103	26.3539	0	13.4488	0	64.816	14.7291	32.1512	22.3723	14.7826	0	pre-mRNA-splicing helicase BRR2, putative
PVX_090170	0	0	0	0	137.898	0	0	0	0	0	171.542	0	hypothetical protein, conserved
PVX_090175	0	64.863	0	0	29.4851	0	142.189	0	175.966	260.96	221.813	0	eukaryotic initiation factor, putative
PVX_090180	0	0	0	0	0	0	0	0	0	0	30.2765	0	hypothetical protein, conserved
PVX_090185	0	0	0	0	52.3516	0	0	0	124.814	115.59	0	0	hypothetical protein, conserved
PVX_090190	0	0	0	0	0	0	0	113.875	62.1293	28.7972	81.585	265.683	hypothetical protein, conserved
PVX_090195	0	0	0	0	134.189	0	0	0	79.9164	0	0	0	AP-4 complex subunit sigma, putative
PVX_090200	0	0	0	0	0	0	0	0	148.572	137.222	77.9049	0	hypothetical protein, conserved
PVX_090205	0	0	0	0	0	0	0	0	0	0	0	0	hypothetical protein, conserved
PVX_090210	0	0	0	0	0	0	0	0	0	0	0	0	asparagine-rich protein (ARP)
PVX_090215	0	0	0	0	0	0	0	0	0	0	0	0	glideosome associated protein with multiple membrane spans 2, putative (GAPM2)
PVX_090220	20.6503	100.878	51.3068	65.947	6.54542	0	0	0	15.645	43.5408	16.441	0	hypothetical protein, conserved
PVX_090225	134.222	0	0	0	0	0	0	0	0	0	53.439	0	hypothetical protein, conserved
PVX_090230	1295.44	775.297	1380.98	1183.36	606.744	315.761	1132.37	1970.61	1495.95	1668.85	1719.05	799.295	early transcribed membrane protein (ETRAMP)
PVX_090235	0	0	0	0	0	0	0	0	0	0	58.0887	0	hypothetical protein
PVX_090240	0	0	0	0	0	0	0	0	0	0	0	0	hypothetical protein, conserved
PVX_090245	0	0	0	0	0	0	0	0	0	0	0	0	Phist protein (Pf-fam-b)

Gene ID / Patient	Patient 1	Patient 2	Patient 3	Patient 4	Patient 5	Patient 6	Patient 7	Patient 8	Patient 9	Patient 10	Patient 11	Patient 12	Gene Description
PVX_090250	0	326.639	291.155	0	37.1309	0	0	324.818	88.6004	41.0496	93.0584	0	tryptophan-rich antigen (Pv-fam-a)
PVX_090255	364.625	636.724	226.932	0	144.717	467.017	279.148	253.29	310.927	128.089	254.036	0	tryptophan-rich antigen (Pv-fam-a)
PVX_090260	106.825	0	0	0	0	0	0	0	0	0	0	0	tryptophan-rich antigen (Pv-fam-a)
PVX_090265	317.703	332.821	0	0	50.4228	813.55	121.567	110.335	60.1984	167.418	15.8101	0	tryptophan-rich antigen (Pv-fam-a)
PVX_090270	0	58.6485	209.004	0	0	0	0	0	0	0	0	0	tryptophan-rich antigen (Pv-fam-a)
PVX_090275	0	108.908	0	0	24.7492	0	0	0	29.5482	0	0	0	tryptophan-rich antigen (Pv-fam-a)
PVX_090280	85.6595	0	0	0	0	0	0	0	32.463	0	0	138.832	PST-A protein
PVX_090285	0	0	0	0	0	0	0	43.4975	0	11.0081	6.2354	0	Pvstp1, putative
PVX_090290	0	0	0	0	0	0	0	0	0	0	7.32823	0	variable surface protein Vir12, putative,PIR protein
PVX_090293	0	77.9878	0	0	0	0	0	0	0	0	0	0	VIR protein, pseudogene,PIR protein, pseudogene
PVX_090295	0	0	0	0	0	0	0	0	0	0	0	0	VIR protein,PIR protein
PVX_090300	0	0	0	0	0	0	0	0	0	73.4693	0	0	Plasmodium exported protein, unknown function
PVX_090305	75.4736	52.7074	0	0	0	0	0	0	57.2015	26.5152	15.0234	0	variable surface protein Vir12-related,PIR protein
PVX_090310	0	0	0	0	0	0	0	0	0	0	0	0	VIR protein,PIR protein
PVX_090315	0	49.5691	0	0	0	0	0	0	0	0	0	0	variable surface protein Vir12-like,PIR protein
PVX_090320	0	0	0	0	0	0	0	0	0	0	18.7175	0	VIR protein, pseudogene,PIR protein
PVX_090325	0	7.54933	0	0	0	0	0	0	0	0	4.30684	0	reticulocyte binding protein 2c (RBP2c)
PVX_090330	0	0	0	0	0	0	0	0	0	0	0	0	reticulocyte binding protein 2 precursor (PvRBP-2), putative
PVX_090335	78.8657	0	0	0	0	0	0	0	0	0	0	0	variable surface protein Vir12-like,PIR protein
PVX_090830	130.124	0	0	0	0	0	0	0	49.3289	0	0	0	hypothetical protein
PVX_090835	0	0	0	0	0	0	0	0	0	0	0	0	hypothetical protein
PVX_090840	0	0	0	0	0	0	0	0	0	0	0	0	hypothetical protein
PVX_090845	622.377	507.265	0	1660.18	98.8368	0	1112.22	432.393	825.636	728.735	392.323	336.408	60S acidic ribosomal protein P1, putative
PVX_090847	0	0	0	0	0	0	0	0	0	0	0	0	tRNA Glycine
PVX_090848	0	0	0	0	0	0	0	0	0	0	0	0	tRNA Leucine
PVX_090850	0	0	0	0	0	0	0	32.9691	0	25.0338	18.9058	0	FeS cluster assembly protein SufD, putative (SufD)
PVX_090860	0	0	0	0	0	0	0	0	0	0	0	0	CPW-WPC family protein, putative
PVX_090865	0	37.1961	0	0	16.9006	0	0	0	40.373	131.036	63.629	86.2706	actin, putative
PVX_090870	107.386	74.9728	0	0	0	274.775	0	149.139	122.064	150.923	117.563	86.9444	casein kinase II beta chain, putative
PVX_090876	26.5697	6.17973	0	28.2742	11.2256	0	0	0	13.4187	24.9	21.1534	0	CCR4-NOT transcription complex subunit 1, putative
PVX_090878	0	2923.75	0	0	0	0	0	0	0	0	0	0	hypothetical protein, conserved
PVX_090880	118.622	41.3959	0	0	18.8037	0	45.3183	82.3524	44.9379	41.6837	70.8327	47.9884	phenylalanyl-tRNA synthetase beta chain, putative
PVX_090885	0	0	0	0	32.088	0	0	0	76.5888	70.9822	60.337	0	hypothetical protein, conserved
PVX_090890	22.3915	15.6266	0	71.5111	0	114.481	0	31.0879	0	15.7372	13.3705	0	helicase, putative,PfSNF2L, putative
PVX_090895	0	0	0	0	0	0	0	0	0	10.4278	11.8133	0	hypothetical protein, conserved
PVX_090900	319.096	111.389	661.279	1019.97	50.6113	0	406.66	369.3	241.806	224.232	264.648	258.35	hypothetical protein, conserved
PVX_090905	0	0	0	0	0	0	0	0	0	0	10.8108	0	WD domain, G-beta repeat domain containing protein
PVX_090910	0	0	0	0	0	0	0	0	0	0	0	0	hypothetical protein, conserved
PVX_090915	500.302	0	0	0	0	1287.73	0	348.082	0	87.8053	149.415	0	RNA polymerase Rpb7, N-terminal domain containing protein
PVX_090920	0	0	0	0	0	0	0	0	32.2775	0	33.9075	138.037	metabolite/drug transporter, putative
PVX_090925	0	0	0	0	0	0	0	0	0	0	0	0	protein kinase domain containing protein
PVX_090930	0	0	0	0	102.886	0	0	0	0	339.162	64.1561	0	histone H4, putative
PVX_090935	257.344	360.279	0	0	246.134	0	396.132	0	390.571	180.654	461.14	419.114	histone 2B
PVX_090940	0	0	0	0	0	0	0	0	0	0	0	0	hypothetical protein, conserved
PVX_090945	0	0	0	0	0	0	0	0	0	0	28.5356	0	hypothetical protein, conserved
PVX_090950	889.246	966.305	984.106	632.459	345.155	1518.96	605.289	1921.95	1423.03	1180.07	904.737	320.393	40S ribosomal protein S4, putative
PVX_090955	122.634	0	0	0	0	0	0	0	0	0	73.2374	0	centrin-4, putative (CEN4)
PVX_090960	0	179.595	91.3651	0	0	0	0	0	27.8505	51.6629	65.8457	0	translocon component PTEX88, putative
PVX_090965	134.298	46.8889	0	0	42.6163	0	102.737	93.2694	203.558	235.916	173.76	0	hypothetical protein, conserved
PVX_090970	171.47	199.531	0	0	0	0	0	0	21.6568	20.0819	91.0161	0	hypothetical protein, conserved
PVX_090972	0	0	0	0	0	0	0	0	0	0	182.019	0	conserved Plasmodium membrane protein, unknown function
PVX_090975	186.606	130.295	0	0	19.7359	0	0	259.181	70.7079	43.7081	86.6694	0	RuvB-like helicase 2, putative (RUVB2)
PVX_090980	0	0	0	480.764	23.8539	0	0	0	0	26.4039	14.9603	121.774	30S ribosomal protein S15, putative
PVX_090985	50.4542	0	0	483.783	16.0039	0	231.458	0	172.047	124.093	90.3851	0	hypothetical protein, conserved
PVX_090990	0	0	0	0	0	0	0	0	19.1487	0	0	81.8308	hypothetical protein, conserved
PVX_090995	0	0	0	0	0	0	0	0	0	0	49.1184	0	hypothetical protein, conserved
PVX_091000	38.9123	0	0	62.1313	6.16672	0	29.7223	54.0234	7.37007	27.3487	19.3627	0	hypothetical protein, conserved
PVX_091005	23.4475	0	0	0	22.2976	0	71.6486	97.6631	53.2936	32.9586	74.6722	37.9359	DEAD/DEAH box helicase, putative
PVX_091010	29.1818	0	0	0	18.503	0	0	0	0	30.7633	23.2336	0	hypothetical protein, conserved
PVX_091015	0	14.996	106.78	0	0	0	0	0	8.13997	15.1025	12.8311	0	protein kinase, putative
PVX_091020	0	0	0	0	0	0	0	0	0	0	0	0	hypothetical protein, conserved
PVX_091025	494.599	0	0	0	0	0	0	0	375.938	693.586	394.026	0	U6 snRNA-associated Sm-like protein LSm4, putative

Gene ID / Patient	Patient 1	Patient 2	Patient 3	Patient 4	Patient 5	Patient 6	Patient 7	Patient 8	Patient 9	Patient 10	Patient 11	Patient 12	Gene Description
PVX_091030	127.438	118.612	0	0	13.4716	0	64.9413	117.978	48.2816	104.483	76.0978	68.7643	nucleic acid binding protein, putative
PVX_091035	0	0	0	0	0	0	161.702	73.4234	0	55.727	52.6169	0	hypothetical protein, conserved
PVX_091040	54.9485	76.6804	77.9847	100.237	22.3859	80.2324	23.9747	65.3815	83.2523	63.45	101.556	38.084	polyadenylate-binding protein-interacting protein 1, putative (PAIP1)
PVX_091045	0	0	0	674.262	100.353	0	0	0	0	110.982	83.8581	0	DNA repair protein RAD51, putative (RAD51)
PVX_091050	0	99.0119	0	453.929	90.068	0	0	0	268.529	199.001	0	0	prefoldin, putative
PVX_091055	29.8535	0	74.1917	0	0	0	0	41.451	0	0	11.8842	0	hypothetical protein, conserved
PVX_091060	54.5108	38.0576	0	0	0	0	0	151.411	103.269	38.3051	43.4012	88.2704	pescadillo N-terminus domain containing protein
PVX_091065	0	0	37.7656	0	4.81801	77.7092	0	0	11.5172	16.0275	30.2588	0	transcription factor with AP2 domain(s), putative (ApiAP2)
PVX_091075	0	0	0	0	0	0	0	0	0	0	0	0	hypothetical protein, conserved
PVX_091080	45.5494	31.7974	0	145.564	0	0	0	63.2542	17.2574	48.014	63.4651	73.7405	hypothetical protein, conserved
PVX_091085	0	0	0	0	0	0	0	0	0	0	7.13451	0	hypothetical protein, conserved
PVX_091090	0	0	0	0	0	0	201.546	0	99.6731	138.515	26.1699	213.34	D-tyrosyl-tRNA(Tyr) deacylase, putative
PVX_091092	640.137	0	0	0	206.005	0	0	0	0	224.26	0	0	pterin-4a-carbinolamine dehydratase, putative
PVX_091095	111.205	194.242	0	0	35.3265	0	85.1906	231.795	189.683	117.184	121.753	0	casein kinase 2, alpha subunit, putative
PVX_091100	257.966	0	214.036	0	0	0	0	238.919	32.5878	90.6231	222.517	0	succinyl-CoA synthetase alpha subunit, putative
PVX_091105	746.552	104.271	185.766	238.774	189.555	0	114.248	311.109	311.195	340.96	564.696	241.918	endoplasmic reticulum-resident calcium binding protein, putative (ERC)
PVX_091110	123.716	86.3825	307.735	0	58.8797	316.636	0	0	70.3167	130.399	160.067	0	DnaJ domain containing protein,heat shock protein DnaJ homologue Pfj2, putative
PVX_091115	0	0	0	0	0	0	0	0	136.05	0	0	0	hypothetical protein, conserved
PVX_091120	0	51.111	0	0	0	0	0	0	0	25.7131	116.55	118.58	hypothetical protein, conserved
PVX_091136	0	0	0	0	0	0	0	0	0	0	9.16827	37.2618	hypothetical protein, conserved
PVX_091137	0	0	0	0	29.7637	0	0	0	0	0	0	0	pre-mRNA splicing factor, putative
PVX_091140	0	52.0181	0	119.051	47.2617	190.593	0	155.223	0	26.1863	29.6669	0	hypothetical protein, conserved
PVX_091145	232.597	325.529	1162.72	747.25	444.658	1196.73	0	970.8	705.904	1388.06	833.563	378.545	60S ribosomal protein L36, putative
PVX_091150	0	0	0	0	0	0	0	0	187.969	0	98.5065	0	hypothetical protein
PVX_091155	0	0	0	0	0	0	0	0	0	0	18.8426	0	hypothetical protein, conserved
PVX_091160	20.7077	28.9022	0	66.1302	13.1272	0	31.6354	57.4992	15.6884	36.3847	20.6083	0	pre-mRNA-processing factor 6, putative
PVX_091165	0	123.981	0	0	0	0	0	0	67.2349	62.2566	0	0	hypothetical protein, conserved
PVX_091170	137.538	95.9817	48.816	0	37.366	100.448	60.0322	54.5573	89.3147	89.7615	109.502	63.5716	asparagine-rich antigen, putative
PVX_091175	30.3393	42.3506	0	0	9.61875	0	0	84.2514	34.4805	21.3222	36.2328	0	vacuolar protein sorting-associated protein 35, putative (VPS35)
PVX_091180	0	0	0	0	0	0	0	0	47.0684	43.6109	74.152	0	50S ribosomal protein L11, putative
PVX_091185	0	0	0	0	0	0	0	0	0	0	0	0	actin, putative
PVX_091190	0	0	0	0	0	0	0	0	0	0	0	0	hypothetical protein, conserved
PVX_091195	0	0	0	200.269	0	0	0	173.997	23.7343	44.0139	24.936	0	ES2 protein, putative
PVX_091200	0	47.5314	0	0	0	0	0	0	77.3797	23.9145	27.0984	0	hypothetical protein, conserved
PVX_091205	105.69	0	0	1014.99	0	0	0	587.429	120.178	111.375	63.1164	0	replication factor C subunit 5, putative
PVX_091210	0	0	0	0	0	0	0	81.0393	44.2177	0	46.4576	0	hypothetical protein, conserved
PVX_091215	0	0	0	0	0	0	0	0	94.9278	0	99.6994	0	hypothetical protein, conserved
PVX_091220	0	0	0	790.69	0	0	0	0	0	86.3587	195.933	0	hypothetical protein, conserved
PVX_091225	0	0	0	0	0	0	0	0	0	0	0	0	acylphosphatase, putative
PVX_091230	0	0	0	0	0	0	0	0	0	0	0	0	endonuclease/exonuclease/phosphatase domain containing protein
PVX_091235	0	28.2829	0	0	12.8489	0	0	0	30.7009	0	16.1297	65.5851	hypothetical protein, conserved
PVX_091240	0	57.9948	413.342	0	0	425.315	0	346.065	31.4684	0	99.1739	0	peptidyl-prolyl cis-trans isomerase, putative
PVX_091245	0	0	0	0	0	0	0	0	0	43.9767	0	0	hypothetical protein, conserved
PVX_091250	0	0	0	0	0	0	0	0	227.681	209.776	119.25	0	hypothetical protein, conserved
PVX_091255	0	0	0	0	0	0	0	0	0	0	12.5093	0	hypothetical protein, conserved
PVX_091260	0	72.2986	0	0	0	0	0	0	0	36.3525	61.8025	167.814	ubiquinone biosynthesis protein COQ4, putative (COQ4)
PVX_091265	34.8392	0	0	0	33.1399	0	0	0	26.3973	12.2421	48.5417	0	hypothetical protein, conserved
PVX_091270	0	0	0	0	0	0	0	0	183.647	84.9591	192.753	0	hypothetical protein, conserved
PVX_091275	251.208	175.451	208.416	535.773	53.1648	0	128.182	232.655	253.869	117.667	200.018	135.707	hypothetical protein, conserved
PVX_091280	0	31.2249	0	0	28.3722	0	0	0	16.9468	15.7168	44.5165	0	hypothetical protein, conserved
PVX_091285	0	0	0	0	0	0	0	39.0287	10.6486	0	27.9747	0	hypothetical protein, conserved
PVX_091290	0	0	0	0	27.8351	0	0	0	0	0	34.9042	0	hypothetical protein, conserved
PVX_091295	0	0	0	0	0	0	0	0	0	0	20.0444	0	hypothetical protein, conserved
PVX_091300	0	0	0	0	0	0	0	0	0	0	1.07267	0	hypothetical protein, conserved
PVX_091305	0	0	0	0	0	0	0	232.681	63.4587	58.7669	0	0	protein tyrosine phosphatase, putative (PRL)
PVX_091307	0	0	0	1714.75	0	0	821.881	0	0	0	0	0	conserved Plasmodium protein, unknown function
PVX_091310	107.596	0	535.963	0	0	0	0	0	40.7824	75.5881	235.601	174.492	UDP-galactose transporter, putative
PVX_091315	67.1491	46.8889	0	0	127.849	0	0	186.539	279.892	212.324	200.493	0	ubiquitin domain-containing protein DSK2, putative (DSK2)
PVX_091320	0	0	0	0	29.2806	0	0	64.1028	52.4667	32.4385	82.6923	0	hypothetical protein, conserved
PVX_091325	0	0	0	0	0	0	0	0	36.0925	0	37.913	0	hypothetical protein, conserved
PVX_091330	146.26	51.0692	545.891	0	69.6284	0	111.909	203.166	193.986	205.537	145.568	0	glyoxalase I, putative
PVX_091335	43.9824	0	0	0	0	0	0	0	0	15.4542	26.2636	0	hypothetical protein, conserved

Gene ID / Patient	Patient 1	Patient 2	Patient 3	Patient 4	Patient 5	Patient 6	Patient 7	Patient 8	Patient 9	Patient 10	Patient 11	Patient 12	Gene Description
PVX_091340	0	0	0	0	0	0	0	0	0	41.2435	35.0489	0	mitogen-activated protein kinase 2, putative
PVX_091345	0	0	0	0	0	0	0	0	0	0	0	0	dynein light chain type 2, putative
PVX_091350	0	0	0	0	0	0	0	0	0	0	33.4616	0	rhomboid protease ROM1, putative (ROM1)
PVX_091355	61.8579	43.1912	0	0	19.6266	0	94.6254	0	46.8778	65.1997	36.9386	0	hypothetical protein, conserved
PVX_091360	0	67.8077	0	0	0	0	0	0	55.2009	34.128	9.66673	0	hypothetical protein, conserved
PVX_091365	0	0	0	0	0	0	0	0	0	0	29.1374	118.58	hypothetical protein, conserved
PVX_091370	0	0	0	0	0	0	0	0	0	0	0	0	hypothetical protein, conserved
PVX_091375	87.8784	92.0181	0	0	13.9351	224.793	0	0	99.883	123.512	96.2052	142.261	serine/threonine-protein kinase PRP4K, putative (CLK3)
PVX_091380	0	0	0	0	0	0	0	0	0	0	36.2909	0	glycerol-3-phosphate dehydrogenase, putative (G3PDH)
PVX_091385	0	0	0	0	0	0	0	0	0	0	9.89484	0	hypothetical protein, conserved
PVX_091390	155.166	0	0	497.406	98.6897	0	0	0	0	163.472	123.559	0	hypothetical protein, conserved
PVX_091395	0	0	0	0	0	0	0	0	0	0	0	0	hypothetical protein
PVX_091400	0	0	0	0	0	0	0	0	0	0	0	0	SET domain protein, putative (SET7)
PVX_091405	214.483	99.8516	0	0	45.3788	0	0	0	108.368	75.3529	71.1558	0	vivapain-2
PVX_091410	139.316	291.854	0	222.76	66.3168	356.645	106.584	96.7564	211.167	146.836	124.79	0	vivapain-3 (VP3)
PVX_091415	141.968	198.276	0	0	67.5814	1090.35	108.617	197.199	134.493	274.322	127.166	114.999	vivapain-2
PVX_091420	0	0	0	0	0	0	0	0	0	120.923	34.2782	0	transcription factor with AP2 domain(s), putative (ApiAP2)
PVX_091425	514.569	0	427.972	0	163.705	0	263.302	476.976	130.083	421.609	443.908	278.668	peptidyl-prolyl cis-trans isomerase 11, putative
PVX_091430	0	0	0	0	0	0	0	0	0	0	26.1315	0	palmitoyltransferase, putative (DHHC9)
PVX_091434	0	0	0	0	0	0	0	0	16.3029	0	0	0	rhoptry neck protein 4 (RON4)
PVX_091436	21.4728	14.9852	0	0	27.225	0	0	29.8121	32.5364	22.6374	42.7395	69.4782	hypothetical protein, conserved
PVX_091440	0	0	0	0	0	0	301.007	0	222.93	343.963	117.022	0	hypothetical protein, conserved
PVX_091445	0	0	0	0	0	0	0	0	225.866	417.686	296.24	0	cyclophilin, putative
PVX_091450	160.886	0	133.367	0	51.0365	274.445	82.0156	148.96	142.237	75.3711	128.097	0	guanine nucleotide-exchange factor SEC12, putative (SEC12)
PVX_091455	77.6342	0	0	496.647	0	0	0	0	0	27.2739	61.8139	0	folate transporter 2, putative (FT2)
PVX_091460	0	0	0	0	12.4102	0	0	0	44.4802	27.503	46.7383	0	oligosaccharyl transferase STT3 subunit, putative
PVX_091465	143.591	66.8279	0	0	0	244.9	0	0	54.4035	33.6353	66.6899	0	dipeptidyl aminopeptidase 1, putative (DPAP1)
PVX_091470	0	99.3593	0	0	45.1362	364.039	0	0	26.9643	125.049	56.6675	0	heat shock protein 101, putative (HSP101)
PVX_091475	0	0	0	0	0	0	0	0	0	0	0	0	hypothetical protein
PVX_091480	30.6195	0	0	97.8112	38.8307	0	0	0	11.5997	53.7979	79.2297	0	hypothetical protein, conserved
PVX_091485	95.6762	33.3961	118.947	152.888	15.1731	0	219.439	66.434	163.123	117.661	199.963	77.4507	ubiquitin C-terminal hydrolase, family 1, putative
PVX_091490	0	0	0	0	0	0	0	27.6244	15.0745	0	31.6829	0	hypothetical protein, conserved
PVX_091495	1459.81	0	0	1574.56	468.043	0	0	0	554.759	511.799	290.738	797.646	hypothetical protein, conserved
PVX_091500	298.061	69.4054	0	317.99	0	0	0	276.089	188.281	69.8008	138.442	0	hypothetical protein, conserved
PVX_091505	67.1491	46.8889	0	0	21.3081	0	0	0	25.4447	70.7748	53.4647	0	tyrosine--tRNA ligase, putative
PVX_091510	0	0	0	0	0	0	0	0	0	0	60.0469	0	Immature colon carcinoma transcript 1, putative
PVX_091515	1012.92	566.832	0	650.281	0	0	0	1127.05	691.531	640.135	524.318	658.844	GTP-binding nuclear protein RAN/TC4, putative
PVX_091520	0	0	0	0	0	0	0	0	0	0	0	0	hypothetical protein
PVX_091525	34.8256	0	86.5606	0	0	0	0	0	0	0	41.591	56.3627	DNA mismatch repair protein MLH, putative
PVX_091530	22.7675	0	0	0	0	0	0	31.61	8.62462	8.00072	4.53165	0	hypothetical protein, conserved
PVX_091535	0	0	0	0	0	0	0	0	0	79.1841	0	0	hypothetical protein, conserved
PVX_091540	0	785.167	0	0	0	0	0	0	170.27	0	44.6827	0	AP-1 complex subunit sigma, putative
PVX_091545	173.993	72.8671	0	0	66.2024	0	159.559	193.278	92.2827	73.3669	103.897	56.3188	heat shock protein 90, putative
PVX_091550	0	0	101.838	0	25.9837	0	62.6184	85.3598	46.5803	64.8177	85.6627	0	hypothetical protein, conserved
PVX_091555	0	0	0	0	0	0	387.104	0	47.866	0	75.4077	0	haloacid dehalogenase-like hydrolase, putative
PVX_091560	204.235	106.938	0	0	97.175	0	156.157	70.9087	174.109	143.52	71.1419	82.6723	nucleolar protein NOP56, putative,SIK1 protein, putative
PVX_091565	53.0565	0	0	0	16.8304	0	0	147.369	20.1027	0	21.1216	0	histone acetyltransferase, putative (MYST)
PVX_091570	0	0	0	0	0	0	0	0	0	0	0	0	hypothetical protein, conserved
PVX_091575	0	0	0	0	0	0	0	0	0	0	0	0	WD domain, G-beta repeat domain containing protein
PVX_091580	0	0	0	0	0	0	0	0	0	0	0	0	hypothetical protein, conserved
PVX_091585	0	46.5047	0	212.961	42.2669	0	0	0	75.709	46.7971	66.2837	0	acyl-CoA-binding protein, putative
PVX_091590	0	39.2539	139.829	179.729	0	287.743	0	0	21.3029	0	22.3823	0	tRNA methyltransferase, putative
PVX_091595	0	191.17	0	0	0	0	0	0	0	95.8312	54.3655	0	conserved protein, unknown function
PVX_091600	119.682	334.514	0	0	38.0273	0	183.419	0	45.3675	126.111	95.2988	388.32	splicing factor U2AF small subunit, putative (U2AF1)
PVX_091605	15.5027	10.8181	77.0235	99.0019	39.3055	0	23.6799	43.0444	105.702	103.512	160.453	25.0764	ubiquitin-protein ligase, putative
PVX_091610	145.075	50.6549	180.486	0	46.042	0	0	201.518	109.95	50.9679	158.827	0	AP-4 complex subunit mu, putative
PVX_091615	0	23.5737	0	0	32.126	0	51.6189	0	12.7951	23.7359	47.0577	54.6594	ATP-dependent zinc metalloprotease FTSH, putative
PVX_091620	0	0	0	0	0	0	0	0	0	0	0	0	hypothetical protein
PVX_091625	0	0	0	0	67.2536	0	0	0	80.1053	0	210.229	0	alternative splicing factor ASF-1, putative
PVX_091630	0	13.4459	0	0	6.10695	0	0	0	7.29866	6.77095	15.3401	0	hypothetical protein, conserved
PVX_091635	32.917	114.876	0	0	0	0	50.3075	91.4121	112.233	57.8339	78.623	0	hypothetical protein, conserved
PVX_091640	1704.15	770.309	249.619	1925.08	477.537	0	1842.39	2228.53	2089.66	1866.29	1336.96	975.216	2,3-bisphosphoglycerate-dependent phosphoglycerate mutase, putative,phosphoglycerate mutase, putative

Gene ID / Patient	Patient 1	Patient 2	Patient 3	Patient 4	Patient 5	Patient 6	Patient 7	Patient 8	Patient 9	Patient 10	Patient 11	Patient 12	Gene Description
PVX_091645	0	0	0	0	0	0	0	0	0	0	0	0	hypothetical protein, conserved
PVX_091650	0	0	0	0	0	0	0	0	0	0	44.6185	121.06	RNA splicing protein MRS2, mitochondrial precursor, putative
PVX_091652	99.3535	0	247.396	0	0	0	152.165	138.045	0	34.9004	59.3321	0	hypothetical protein, conserved
PVX_091655	78.6839	54.9238	97.7985	251.41	12.4757	201.246	60.1391	54.6307	74.5248	82.944	93.9698	0	tRNA nucleotidyltransferase, putative
PVX_091660	0	0	0	0	0	0	0	0	0	0	3.72812	0	hypothetical protein
PVX_091662	0	18.5186	0	0	0	0	0	0	0	0	5.28141	0	hypothetical protein, conserved
PVX_091664	0	0	0	0	0	0	0	0	0	0	0	0	conserved Plasmodium protein, unknown function
PVX_091665	0	0	0	0	0	0	0	0	0	0	0	0	heat shock factor-binding protein 1, putative (HSBP)
PVX_091670	0	93.8055	0	0	0	0	0	0	0	0	0	0	palmitoyltransferase, putative (DHHC3)
PVX_091675	39.566	13.8057	49.1507	0	6.27037	0	0	0	0	13.9041	23.6256	0	liver stage antigen, putative
PVX_091680	0	0	0	0	75.491	0	0	0	89.8757	0	0	0	hypothetical protein, conserved
PVX_091685	0	0	0	0	0	0	0	0	0	0	0	0	tyrosine kinase-like protein, putative (TKL2)
PVX_091690	0	0	0	0	0	0	0	0	0	0	0	0	WD domain, G-beta repeat domain containing protein
PVX_091695	0	0	0	0	0	0	0	0	0	0	25.5806	0	hypothetical protein, conserved
PVX_091700	165.04	115.226	410.447	0	69.8079	563.085	84.1368	76.4039	187.6	135.304	153.305	0	circumsporozoite-related antigen, putative (EXP1)
PVX_091705	138.924	24.2419	172.65	0	22.0246	0	53.0829	192.903	65.7883	85.4286	82.9559	56.2094	ABC transporter, putative,ATP-binding cassette, sub-family F, member 2, putative
PVX_091710	0	0	0	0	0	0	0	0	0	0	0	0	hypothetical protein, conserved
PVX_091715	0	42.1418	0	0	0	0	0	0	0	0	0	0	3-phosphoinositide dependent protein kinase-1, putative
PVX_091720	0	0	0	0	22.6894	0	0	0	0	50.2353	0	0	conserved Plasmodium protein, unknown function
PVX_091725	0	0	0	0	0	0	0	0	0	0	26.4348	0	GPI transamidase component GPI16, putative (GPI16)
PVX_091730	44.6405	0	0	0	14.1578	0	0	0	0	47.0562	8.88553	0	cupin-like protein, putative
PVX_091735	0	89.6057	0	0	0	0	0	0	0	22.5432	12.772	0	hypothetical protein, conserved
PVX_091740	0	0	0	0	9.67125	0	0	0	11.5562	0	6.07174	0	hypothetical protein, conserved
PVX_091745	16.2723	0	0	0	0	0	0	0	0	0	6.47762	0	hypothetical protein, conserved
PVX_091750	0	0	0	0	0	0	0	0	0	0	13.2664	0	hypothetical protein, conserved
PVX_091752	0	0	0	0	49.0885	0	0	0	58.5279	0	92.1898	0	conserved Plasmodium protein, unknown function
PVX_091755	0	0	0	0	0	0	0	26.8889	0	0	11.5648	62.6631	calcium-dependent protein kinase 6, putative (CDPK6)
PVX_091760	0	0	0	0	1.7955	0	0	0	2.14638	1.99152	4.51154	0	dynein heavy chain, putative
PVX_091765	0	39.9306	35.5371	45.6775	18.1349	0	0	0	16.2567	35.1918	19.9318	0	hypothetical protein, conserved
PVX_091770	0	0	0	0	0	0	0	0	11.7569	10.9074	0	25.1025	calcium-dependent protein kinase 7, putative (CDPK7)
PVX_091775	0	0	0	0	13.5101	0	0	0	0	0	16.9589	0	leucine-rich repeat protein (LRR11)
PVX_091780	105.737	184.551	131.473	168.989	33.5413	0	0	0	100.157	74.3027	52.6169	0	RING zinc finger protein, putative
PVX_091785	40.2986	168.78	0	128.765	12.7794	0	61.6033	0	137.407	113.281	104.276	0	translation elongation factor EF-1, subunit alpha, putative
PVX_091790	26.0704	54.5845	0	0	0	0	0	0	19.7521	18.3225	57.08	42.1827	hypothetical protein, conserved
PVX_091795	79.7711	55.683	99.1512	127.444	0	0	0	55.3858	45.3328	28.0299	63.5121	0	hypothetical protein, conserved
PVX_091800	0	0	0	0	0	1806.73	540.231	0	0	0	0	0	hypothetical protein, conserved
PVX_091805	51.914	0	0	0	0	0	39.6616	0	9.83307	27.3642	0	41.999	SMC family, C-terminal domain containing protein
PVX_091810	196.741	0	0	0	62.4789	0	0	0	74.5669	69.1106	97.9085	0	ribosomal protein L7A, putative
PVX_091815	74.7175	208.715	0	0	0	0	0	103.79	0	26.2496	104.11	242.121	endoplasmic reticulum oxidoreductin, putative
PVX_091820	0	14.5555	0	0	6.61104	0	0	0	31.6036	14.659	16.6057	0	hypothetical protein, conserved
PVX_091825	0	0	0	0	0	0	0	110.335	90.2976	27.903	31.6202	128.706	hypothetical protein, conserved
PVX_091830	114.68	0	0	0	18.191	0	0	0	0	0	0	0	hypothetical protein, conserved
PVX_091835	362.677	0	0	0	0	0	560.165	0	0	0	0	0	LSM domain containing protein
PVX_091840	0	0	0	0	0	0	0	0	0	0	0	0	pyruvate dehydrogenase E1 component subunit alpha, putative
PVX_091845	0	0	0	0	46.6097	0	0	0	27.826	77.3924	58.4662	0	ethanolamine kinase, putative
PVX_091850	124.685	87.1295	0	0	0	0	0	173.284	141.796	0	148.924	0	GrpE protein homolog, mitochondrial, putative (MGE1)
PVX_091855	127.71	0	0	0	40.5858	0	0	177.493	96.8259	134.564	25.4229	0	ribosome biogenesis regulatory protein (RRS1), putative
PVX_091860	43.446	30.3197	53.9735	0	20.6568	0	33.1877	60.3191	65.8312	45.8023	112.418	175.72	hypothetical protein
PVX_091865	628.291	549.04	0	503.544	349.674	806.319	482.018	436.73	714.674	606.757	719.196	1020.35	60S ribosomal protein L35, putative,15kD antigen, putative
PVX_091870	0	0	0	0	0	0	0	0	51.4243	0	0	0	hypothetical protein, conserved
PVX_091875	69.9847	0	0	0	66.6285	0	0	0	106.079	24.5875	111.445	113.377	hypothetical protein, conserved
PVX_091880	332.839	0	0	0	0	0	0	0	0	116.879	33.1305	0	hypothetical protein, conserved
PVX_091885	23.5215	16.4154	0	75.1221	22.368	0	0	65.3142	62.372	24.7969	23.4086	38.0556	DNA-dependent RNA polymerase, putative
PVX_091890	50.8556	71.008	0	0	0	0	0	0	57.8054	0	50.6135	82.343	mitochondrial import inner membrane translocase subunit TIM44, putative (TIM44)
PVX_091895	0	0	0	0	40.5278	0	0	0	145.032	0	76.16	0	small nuclear ribonucleoprotein Sm D1, putative
PVX_091900	0	0	0	0	0	0	0	0	0	512.454	0	0	hypothetical protein
PVX_091905	0	0	0	0	18.1561	0	0	0	0	0	11.3916	92.6804	kelch domain-containing protein
PVX_091910	0	0	0	0	0	0	0	0	0	0	11.8936	0	kelch domain-containing protein
PVX_091915	174.875	244.562	0	0	0	0	0	0	265.256	184.217	104.442	0	hypothetical protein, conserved
PVX_091920	35.2926	123.17	175.445	338.261	67.1431	180.51	53.9421	245.029	120.334	62.0069	168.595	0	threonine--tRNA ligase, putative
PVX_091922	0	0	0	0	16.457	0	0	72.0789	0	9.1214	15.4996	0	autophagy-related protein 7, putative (ATG7)
PVX_091925	836.703	1754.39	1390.53	2234.14	354.639	715.478	1710.79	775.331	1480.31	1175.41	1082.66	905.424	40S ribosomal protein S18, putative

Gene ID / Patient	Patient 1	Patient 2	Patient 3	Patient 4	Patient 5	Patient 6	Patient 7	Patient 8	Patient 9	Patient 10	Patient 11	Patient 12	Gene Description
PVX_091930	0	31.1626	0	0	0	0	0	61.9915	0	0	0	0	DnaJ domain containing protein
PVX_091935	0	64.3284	229.274	589.392	0	0	0	0	104.71	32.3516	54.9966	0	hypothetical protein, conserved
PVX_091940	59.9453	97.6108	0	0	12.6668	0	0	55.4832	22.7076	56.1751	47.726	32.3255	hypothetical protein, conserved
PVX_091945	0	0	0	0	0	0	0	0	0	0	0	0	hypothetical protein, conserved
PVX_091950	0	0	0	0	10.7574	0	0	0	0	0	20.2594	0	hypothetical protein, conserved
PVX_091955	0	0	0	0	0	0	0	41.7856	0	0	11.9801	0	RNA-binding protein, putative
PVX_091960	0	935.91	0	0	862.511	0	0	0	0	0	0	0	U6 snRNA-associated Sm-like protein LSm6, putative
PVX_091965	138.587	193.723	0	0	0	711.027	0	0	210.161	97.3449	137.943	0	hypothetical protein, conserved
PVX_091970	119.682	83.6284	298.187	0	76.0546	0	0	498.97	45.3675	126.111	238.247	194.16	deoxyuridine 5'-triphosphate nucleotidohydrolase, putative
PVX_091975	0	0	0	0	211.924	0	511.895	0	0	0	66.0596	0	hypothetical protein, conserved
PVX_091980	0	131.088	0	0	0	0	0	0	35.5623	32.9619	37.3564	0	tRNA (guanine-N(7)-)-methyltransferase, putative
PVX_091985	0	0	0	0	0	0	0	0	34.2334	63.463	17.9806	0	hypothetical protein, conserved
PVX_091990	83.5016	0	207.831	0	26.5078	0	0	0	31.6447	0	49.8646	0	hypothetical protein, conserved
PVX_091992	0	0	0	422.38	0	0	0	0	99.9671	46.3076	209.976	0	hypothetical protein
PVX_091995	0	0	0	0	0	0	0	0	0	0	0	0	hypothetical protein
PVX_092000	61.7089	100.483	0	0	6.51983	0	0	0	38.9596	7.22845	40.9418	0	hypothetical protein, conserved
PVX_092005	0	0	0	0	0	0	0	0	31.4684	0	0	0	hypothetical protein, conserved
PVX_092010	0	28.3953	33.6943	43.3089	4.29863	0	0	37.6613	15.4139	14.3005	24.2982	0	hypothetical protein, conserved
PVX_092015	0	0	0	0	0	0	0	0	0	0	0	0	conserved Plasmodium protein, unknown function
PVX_092025	330.993	115.709	825.729	0	52.6432	0	0	230.095	62.7537	0	230.627	0	prefoldin subunit 5, putative
PVX_092030	0	0	0	0	0	2229.24	0	1200.73	0	302.079	171.542	0	multiprotein bridging factor type 1, putative
PVX_092035	0	0	0	0	6.88058	0	0	0	8.22291	0	4.3206	0	6-phosphofructokinase, putative
PVX_092040	96.775	67.6019	0	0	0	495.887	148.204	268.917	110.035	33.9951	77.0561	0	geranylgeranyl pyrophosphate synthase (GGPPS)
PVX_092045	38.9991	27.2224	0	0	37.1006	199.49	0	0	14.775	13.7035	54.338	0	hypothetical protein, conserved
PVX_092050	0	0	0	0	0	0	0	0	21.8946	0	23.0038	0	hypothetical protein, conserved
PVX_092055	0	0	0	0	0	0	0	0	0	0	27.2018	0	GPI8p transamidase, putative
PVX_092060	0	0	0	0	0	0	0	0	0	0	0	0	hypothetical protein, conserved
PVX_092065	259.823	90.7123	161.587	0	61.8332	0	397.497	451.105	344.586	365.141	349.098	0	spermidine synthase, putative
PVX_092070	578.587	565.555	431.71	369.932	201.915	592.259	176.993	482.147	350.767	487.885	368.537	93.7007	parasitophorous vacuolar protein 1, putative (PV1)
PVX_092075	449.431	52.31	186.39	239.575	118.869	0	0	312.151	141.926	131.578	208.743	0	26S proteasome regulatory subunit RPN7, putative (RPN7)
PVX_092080	0	0	0	0	0	0	0	0	0	0	0	0	hypothetical protein, conserved
PVX_092085	163.234	75.9763	0	173.928	0	0	0	75.5673	0	57.3529	75.8136	0	proliferating-cell nucleolar antigen p120, putative
PVX_092090	0	31.5563	0	0	14.3368	0	0	0	17.1266	15.8834	44.9887	0	A/G-specific adenine glycosylase, putative
PVX_092095	0	0	79.0195	0	0	0	0	0	12.0449	0	0	0	phosphatidylinositol-4-phosphate 5-kinase, putative
PVX_092100	0	93.8055	0	0	0	0	0	0	0	0	53.439	0	hypothetical protein, conserved
PVX_092105	59.1591	123.916	0	189.13	37.5381	0	0	0	44.8317	62.3559	105.981	0	transporter, putative
PVX_092110	123.396	0	0	0	39.1319	0	0	0	93.5001	28.9058	81.8715	0	phosphoacetylglucosamine mutase, putative
PVX_092115	2108.81	3726.75	2694.8	0	0	0	1663.3	4434.88	2815.13	2949.53	2942.02	1754.68	60S ribosomal protein L38, putative (RPL38)
PVX_092120	853.328	1251.6	424.8	1911.05	216.723	437.106	522.532	1066.93	549.765	959.276	713.416	138.301	60S acidic ribosomal protein P0, putative,ribosomal phosphoprotein P0, putative
PVX_092125	159.417	0	0	254.973	25.3014	0	366.004	0	151.032	168.014	269.729	0	26S protease regulatory subunit 6a, putative (RPT5)
PVX_092130	0	0	0	0	0	0	0	41.1489	0	10.414	17.6964	0	hypothetical protein, conserved
PVX_092135	0	0	93.0094	0	0	0	0	0	42.5269	0	22.3432	0	hypothetical protein, conserved
PVX_092140	19.2925	13.4633	47.9315	0	0	0	0	0	7.3081	6.77971	34.5598	31.2099	structural maintenance of chromosome protein, putative
PVX_092145	0	0	0	0	0	0	0	0	0	0	0	0	PF16 protein, putative
PVX_092150	0	0	0	0	9.0047	0	0	0	10.7601	9.98109	22.6142	0	hypothetical protein, conserved
PVX_092155	153.833	0	0	0	0	0	0	0	116.653	0	91.8728	0	RNA-binding protein, putative
PVX_092160	0	0	0	0	0	0	0	0	0	0	13.3244	0	hypothetical protein, conserved
PVX_092165	0	0	0	0	0	0	0	0	0	0	36.6267	149.134	hypothetical protein, conserved
PVX_092170	0	0	0	0	42.1412	0	0	0	0	0	13.2174	0	hypothetical protein, conserved
PVX_092175	20.6694	0	0	0	6.55147	0	0	0	39.1486	7.2635	16.4561	0	hypothetical protein, conserved
PVX_092180	0	0	0	0	0	0	0	0	0	0	0	0	hypothetical protein, conserved
PVX_092185	0	0	0	0	0	0	0	0	4.88687	0	15.4071	0	hypothetical protein, conserved
PVX_092190	0	0	0	0	0	0	0	0	0	0	0	0	hypothetical protein
PVX_092195	59.3506	0	0	0	0	0	45.3484	0	33.7258	31.2835	17.7199	0	oxysterol-binding protein/PH domain containing protein
PVX_092200	0	0	0	0	0	0	72.1421	131.046	0	0	37.566	0	hypothetical protein, conserved
PVX_092205	53.3214	37.2139	66.251	0	8.45173	0	0	0	30.2991	37.4746	37.1461	0	ubiquitin domain containing protein
PVX_092210	0	0	0	0	0	0	0	0	0	0	0	0	hypothetical protein, conserved
PVX_092215	139.415	97.3252	115.545	445.548	73.6962	0	71.0541	193.607	193.677	228.602	138.751	75.2358	T-complex protein 1, alpha subunit, putative
PVX_092220	0	0	0	0	0	0	0	0	109.141	50.5498	143.269	0	hypothetical protein, conserved
PVX_092225	25.0069	0	62.1384	0	15.8543	0	0	34.7199	37.8923	26.3627	44.7965	40.4606	hypothetical protein, conserved
PVX_092230	0	0	0	0	0	0	0	31.2522	0	0	13.4411	0	hypothetical protein, conserved
PVX_092235	0	61.7822	0	0	28.0832	0	0	0	33.5225	31.0732	52.822	0	pre-mRNA-splicing factor 38A, putative (PRP38A)

Gene ID / Patient	Patient 1	Patient 2	Patient 3	Patient 4	Patient 5	Patient 6	Patient 7	Patient 8	Patient 9	Patient 10	Patient 11	Patient 12	Gene Description
PVX_092240	121.14	0	301.832	0	0	0	0	0	0	85.0979	48.23	196.534	50S ribosomal protein L2, putative
PVX_092245	0	0	0	0	99.2077	0	0	0	0	0	31.0514	0	aquaglyceroporin, putative (AQP)
PVX_092250	117.637	0	0	0	0	0	0	0	44.5916	41.3192	0	0	glycine cleavage system H protein, putative
PVX_092260	24.1303	50.5212	0	0	7.6491	123.378	36.8683	67.0052	27.4229	8.47957	48.0292	0	hypothetical protein, conserved
PVX_092265	21.8343	30.4751	0	69.7304	6.92089	0	0	0	8.27106	7.67282	43.459	0	hypothetical protein, conserved
PVX_092270	0	0	0	0	0	0	0	0	0	0	14.2768	0	LEM3/CDC50 family protein, putative
PVX_092275	0	0	0	0	0	0	0	0	0	0	0	0	apical membrane antigen 1 (AMA1)
PVX_092280	0	0	0	0	15.3455	0	0	67.1886	109.984	84.9972	77.0411	78.3312	hypothetical protein, conserved
PVX_092285	56.4714	0	0	0	0	0	0	0	21.3971	0	33.7218	0	hypothetical protein, conserved
PVX_092290	0	24.939	0	0	11.3291	0	0	49.6124	13.536	37.6645	49.782	0	hypothetical protein, conserved
PVX_092300	32.3811	0	0	0	10.2666	0	0	0	49.0688	22.757	32.2262	52.4028	hypothetical protein, conserved
PVX_092305	0	28.5129	0	0	6.47516	0	0	28.3623	7.73856	7.17895	20.3307	0	hypothetical protein, conserved
PVX_092310	62.3049	173.945	77.4227	99.5151	49.3838	0	47.608	129.765	165.223	142.309	136.415	151.238	heat shock protein 70, putative
PVX_092315	83.5016	58.3198	207.831	0	26.5078	0	0	116.001	63.2894	176.006	166.215	0	protein disulfide isomerase, putative
PVX_092320	14.5856	10.1779	36.2324	0	9.24483	74.5543	0	0	16.5747	20.5029	29.0309	0	hypothetical protein, conserved
PVX_092325	0	0	0	0	0	0	0	0	5.27224	4.89136	2.77034	0	hypothetical protein, conserved
PVX_092330	0	0	0	0	0	0	0	0	0	0	55.3796	0	hypothetical protein, conserved
PVX_092335	0	0	0	0	0	0	0	0	6.82679	6.3333	17.9355	29.1537	hypothetical protein, conserved
PVX_092340	0	0	0	0	0	0	0	0	0	0	0	0	zinc finger protein, putative
PVX_092345	0	69.6132	0	0	12.647	0	0	0	52.9018	21.0328	19.8548	0	DNA-directed RNA polymerase I subunit RPA2, putative (RPA2)
PVX_092350	132.215	230.805	0	211.385	20.9771	0	101.14	91.8216	25.0498	0	65.7941	0	coatomer delta subunit, putative
PVX_092355	0	0	0	0	0	0	0	0	0	0	0	0	hypothetical protein, conserved
PVX_092360	0	0	0	0	0	0	0	0	0	0	0	0	PQ loop repeat family protein
PVX_092365	0	185.62	0	0	36.1391	582.956	58.0688	105.503	100.746	186.883	105.862	0	protein phosphatase 2C domain containing protein
PVX_092370	0	0	0	0	0	0	0	0	0	0	6.64727	0	hypothetical protein, conserved
PVX_092375	87.1612	60.8787	0	0	0	0	133.44	0	33.0324	61.2388	34.7002	0	hypothetical protein, conserved
PVX_092380	0	0	0	0	0	0	0	0	0	0	0	0	hypothetical protein
PVX_092385	171.648	0	427.25	0	81.7398	0	131.387	238.46	227.677	180.899	239.174	139.099	hypothetical protein, conserved
PVX_092390	64.721	0	0	206.943	0	0	0	179.789	73.5727	90.9552	38.6484	104.834	hypothetical protein, conserved
PVX_092395	0	0	44.2917	0	5.65053	0	0	49.5025	13.5067	18.7955	17.7426	28.8399	hypothetical protein, conserved
PVX_092400	0	0	0	0	0	0	3407.62	0	806.712	0	0	0	hypothetical protein
PVX_092405	0	0	0	0	0	0	0	0	128.324	0	0	0	hypothetical protein, conserved
PVX_092410	130.124	181.873	0	0	41.3554	0	199.487	180.852	98.6578	91.4041	77.7106	0	3-oxo-5-alpha-steroid 4-dehydrogenase, putative
PVX_092415	0	0	0	0	0	0	0	0	3.62015	0	1.90228	0	hypothetical protein, conserved
PVX_092420	0	34.4653	0	0	0	0	0	0	0	17.3463	0	0	hypothetical protein, conserved
PVX_092425	0	0	0	0	0	0	0	0	0	0	0	0	hypothetical protein, conserved
PVX_092430	74.1708	17.2546	0	0	23.5119	0	75.551	0	84.2918	69.5043	34.4471	0	tudor staphylococcal nuclease, putative (TSN)
PVX_092435	75.5934	26.3827	93.9532	0	11.9852	193.333	57.7741	52.4841	157.513	79.6868	120.372	61.1763	signal recognition particle subunit SRP72, putative (SRP72)
PVX_092440	133.855	31.147	0	0	28.3014	0	68.216	123.921	50.7136	62.7103	8.8811	0	casein kinase 1, putative (CK1)
PVX_092445	0	34.8268	0	0	0	0	0	0	9.45194	17.5359	4.96629	0	hypothetical protein, conserved
PVX_092450	0	0	0	0	0	0	0	0	0	0	17.2493	0	hypothetical protein, conserved
PVX_092455	0	0	0	0	0	0	0	0	71.9551	0	0	0	DnaJ domain containing protein
PVX_092460	0	0	0	0	5.84989	0	28.195	0	0	0	3.67367	0	subtilisin-like protease 2, putative
PVX_092465	146.74	0	0	0	23.2857	0	0	0	55.6063	77.329	87.6273	0	30S ribosomal protein S9, putative
PVX_092470	0	0	0	0	0	0	123.658	0	0	28.3814	0	0	hypothetical protein, conserved
PVX_092475	0	0	0	0	0	0	0	0	0	0	0	0	hypothetical protein
PVX_092480	102.292	107.121	127.184	326.952	0	0	78.2127	71.0304	38.7573	35.9414	91.6249	82.8143	hypothetical protein, conserved
PVX_092485	0	160.562	0	0	0	0	117.288	106.458	29.0419	53.8474	45.7649	0	hypothetical protein, conserved
PVX_092490	0	0	0	0	92.1813	0	0	402.174	0	0	0	0	30S ribosomal protein S14, putative
PVX_092495	0	0	0	0	0	0	0	0	0	0	0	0	hypothetical protein, conserved
PVX_092500	0	0	0	0	0	0	0	0	0	0	0	0	hypothetical protein, conserved
PVX_092505	0	0	0	0	0	0	0	0	0	0	0	0	hypothetical protein, conserved
PVX_092510	0	0	0	0	0	0	0	0	0	0	8.08679	0	hypothetical protein, conserved
PVX_092515	0	0	0	0	0	0	0	0	0	0	2.05717	0	hypothetical protein, conserved
PVX_092520	0	0	0	0	0	901.727	0	244.112	199.724	61.6465	139.803	0	ubiquitin-related modifier 1, putative (URM1)
PVX_092525	0	0	0	0	0	0	0	0	0	0	0	0	hypothetical protein, conserved
PVX_092530	76.3103	0	0	0	0	0	0	0	0	26.809	30.3798	0	hypothetical protein, conserved
PVX_092535	0	0	0	0	0	0	0	0	0	0	3.03533	0	Adenylate and Guanylate cyclase catalytic domain containing protein
PVX_092540	76.4736	124.533	63.3428	244.253	40.4038	0	77.8986	106.178	106.222	89.5774	208.026	0	protein phosphatase 2C, putative
PVX_092545	57.2664	39.9829	0	0	0	0	0	0	0	0	68.3931	0	beta-catenin-like protein 1, putative
PVX_092550	0	10.8237	38.5318	0	0	0	0	21.5334	0	10.9017	12.349	0	hypothetical protein, conserved
PVX_092555	0	46.0487	0	0	5.2285	0	0	45.8062	15.6227	13.5274	17.5125	0	WD domain, G-beta repeat domain containing protein

Gene ID / Patient	Patient 1	Patient 2	Patient 3	Patient 4	Patient 5	Patient 6	Patient 7	Patient 8	Patient 9	Patient 10	Patient 11	Patient 12	Gene Description
PVX_092560	0	0	0	0	0	0	0	0	0	0	0	0	hypothetical protein, conserved
PVX_092565	0	0	0	0	0	0	134.226	0	0	92.3979	52.3563	0	hypothetical protein, conserved
PVX_092570	10.8035	15.0768	0	34.4915	13.694	0	0	0	0	11.39	17.2024	34.9457	transcription factor with AP2 domain(s), putative (ApiAP2)
PVX_092575	0	0	0	0	0	0	0	0	0	0	21.197	0	AAA family ATPase, putative
PVX_092580	0	0	0	0	0	0	0	0	0	0	9.90871	0	hypothetical protein
PVX_092585	16.9784	0	0	54.2927	10.7755	0	0	0	15.0118	0	16.8979	0	adrenodoxin reductase, putative
PVX_092590	29.3145	0	0	0	4.64514	0	0	20.3482	5.55204	0	17.5041	0	hypothetical protein, conserved
PVX_092595	0	0	0	0	144.813	0	0	0	172.436	79.7867	45.2502	0	conserved protein, unknown function
PVX_092600	0	357.053	0	0	54.1514	0	0	0	0	119.544	135.548	276.537	vacuolar ATP synthase subunit f, putative
PVX_092605	115.419	40.2925	0	0	36.617	0	353.071	80.1505	196.797	81.1045	114.871	93.4586	hypothetical protein, conserved
PVX_092610	0	0	0	264.66	0	0	0	0	94.056	58.1265	98.8072	0	hypothetical protein, conserved
PVX_092615	0	0	0	0	0	0	0	0	0	0	0	0	hypothetical protein, conserved
PVX_092620	0	0	0	0	0	0	0	0	0	0	0	0	myosin heavy chain subunit, putative
PVX_092625	0	0	0	0	0	0	0	0	0	0	13.3713	0	hypothetical protein
PVX_092630	22.5545	39.3451	28.0115	36.0045	10.721	0	0	46.9662	17.0865	27.7421	65.0932	18.2393	hypothetical protein, conserved
PVX_092635	136.801	23.8712	85.0044	0	0	0	0	47.4883	38.8694	72.1054	34.0366	0	hypothetical protein, conserved
PVX_092640	17.7329	0	0	0	5.62029	0	0	0	0	0	0	0	hypothetical protein, conserved
PVX_092650	0	0	0	0	0	0	0	0	0	0	0	0	hypothetical protein, conserved
PVX_092655	0	0	0	0	0	0	0	0	0	0	2.2574	0	hypothetical protein
PVX_092660	0	0	389.014	0	0	0	0	0	0	0	155.257	0	hypothetical protein, conserved
PVX_092665	0	0	0	0	0	0	0	0	0	0	0	0	hypothetical protein
PVX_092670	0	0	0	0	0	0	49.3267	0	0	0	0	0	hypothetical protein, conserved
PVX_092675	0	0	0	1032.57	0	0	247.111	0	61.0581	56.5481	64.1145	261.542	phosphatidylinositol N-acetylglucosaminyltransferase subunit H, putative (PIGH)
PVX_092680	0	0	0	0	0	0	0	0	0	0	25.791	0	hypothetical protein, conserved
PVX_092685	152.782	0	0	0	0	0	0	0	0	53.6542	60.8302	0	dolichol-phosphate mannosyltransferase, putative (DPM1)
PVX_092690	78.3136	109.385	0	0	0	0	0	108.789	29.6775	82.5375	77.9436	253.8	hypothetical protein, conserved
PVX_092695	0	0	0	0	0	0	0	0	0	0	0	0	hypothetical protein, conserved
PVX_092700	0	39.1309	0	0	0	0	0	0	0	0	0	0	inner membrane complex protein 1b, putative (IMC1b)
PVX_092705	0	0	0	0	0	0	0	0	0	0	0	0	hypothetical protein, conserved
PVX_092710	30.7793	28.6358	50.967	32.7551	9.75346	0	15.6688	28.4855	11.6586	14.4224	34.7153	0	hypothetical protein, conserved
PVX_092715	0	113.192	0	0	0	0	0	0	0	0	0	0	hypothetical protein, conserved
PVX_092720	0	0	0	0	0	0	0	0	6.69334	0	3.51699	0	hypothetical protein, conserved
PVX_092725	66.9983	93.567	0	0	0	0	102.505	0	76.1626	70.6159	53.3446	0	coproporphyrinogen-III oxidase, putative
PVX_092730	808.056	225.975	806.256	0	154.207	829.73	248.008	449.369	612.789	567.522	579.115	0	60S ribosomal protein L28, putative
PVX_092735	560.785	915.11	466.613	0	118.984	960.443	0	519.894	1134.25	1115.78	483.787	303.829	60S ribosomal protein L35ae, putative
PVX_092740	0	40.2665	0	0	0	0	0	0	21.8523	20.2631	22.9593	0	methyltransferase, putative
PVX_092745	0	48.7562	0	223.282	44.3148	0	0	96.983	79.3729	98.1198	97.2864	113.111	hypothetical protein, conserved
PVX_092750	0	0	0	0	0	0	0	65.0086	0	0	0	0	hypothetical protein
PVX_092755	83.5016	58.3198	0	0	26.5078	0	0	0	0	29.3343	116.351	135.326	alpha/beta hydrolase, putative,alpha/beta hydrolase, putative
PVX_092760	0	0	0	0	0	0	0	0	0	0	3.21079	0	transcription factor with AP2 domain(s), putative (AP2-O)
PVX_092765	84.0505	58.7036	0	0	0	0	128.665	0	63.7057	147.635	66.9233	0	DnaJ protein, putative
PVX_092770	0	0	0	0	0	0	0	155.692	42.4686	78.7092	44.6066	181.724	DNA-directed RNA polymerase I, putative
PVX_092775	73.2498	306.917	0	702.81	46.495	0	112.092	0	305.334	231.607	174.968	0	translation initiation factor eIF-1A, putative
PVX_092780	0	0	0	0	0	0	0	0	0	0	25.8652	0	hypothetical protein, conserved
PVX_092785	0	0	0	0	0	0	0	0	0	0	18.0718	0	hypothetical protein, conserved
PVX_092790	0	0	0	0	0	0	0	0	0	0	8.07579	0	hypothetical protein, conserved
PVX_092795	0	189.294	0	0	0	154.107	0	0	34.2483	52.9466	35.9888	0	hypothetical protein, conserved
PVX_092800	0	50.4502	0	0	22.9279	0	0	0	0	50.7623	50.3318	0	carbohydrate kinase, putative
PVX_092805	664.954	465.25	553.832	711.866	353.016	0	681.6	616.678	1261.19	855.94	750.196	721.239	40S ribosomal protein S21, putative
PVX_092810	0	0	0	0	178.557	0	0	0	0	98.2254	167.178	0	mitochondrial large subunit ribosomal protein, putative
PVX_092815	0	0	0	0	0	0	0	0	0	0	0	0	hypothetical protein, conserved
PVX_092820	5705.59	1506.42	1806.73	4644.54	919.656	7444.97	2227.65	996.871	2985.87	4246.07	1420.84	1176.42	60S ribosomal protein L41, putative
PVX_092825	0	0	0	0	0	0	0	30.3586	8.28319	7.68407	13.0568	0	hypothetical protein, conserved
PVX_092830	59.0031	0	0	188.63	18.7195	0	0	163.896	22.3567	62.1915	0	0	ubiquitin activating enzyme, putative
PVX_092835	0	134.331	0	0	0	0	0	0	109.325	101.328	19.1398	0	hypothetical protein, conserved
PVX_092840	0	0	0	0	0	0	0	0	65.2384	30.2367	119.932	0	apicoplast import protein Tic20, putative (TIC20)
PVX_092845	0	0	0	0	0	0	0	0	0	17.0918	0	0	hypothetical protein, conserved
PVX_092850	212.696	0	0	0	0	0	0	0	80.6773	74.6722	296.42	0	ras-related protein Rab-6, putative (RAB6)
PVX_092855	0	0	0	0	0	0	0	0	0	0	0	0	hypothetical protein, conserved
PVX_092860	94.8775	110.368	0	101.029	50.1348	0	0	43.9126	71.8861	111.131	75.5388	51.1795	coatomer subunit gamma, putative
PVX_092865	0	0	0	0	0	0	0	0	0	0	0	0	protein kinase domain containing protein
PVX_092870	0	216.341	0	0	0	0	0	430.018	0	0	61.4981	0	cysteine-rich PDZ-binding protein, putative (CRIPT)

Gene ID / Patient	Patient 1	Patient 2	Patient 3	Patient 4	Patient 5	Patient 6	Patient 7	Patient 8	Patient 9	Patient 10	Patient 11	Patient 12	Gene Description
PVX_092875	119.763	27.8664	99.2398	0	0	0	0	110.87	45.3733	42.0824	87.4071	0	dynamin-like protein, putative
PVX_092880	83.2979	0	0	0	13.208	0	0	115.671	15.7792	73.1723	49.7402	0	ABC transporter, putative
PVX_092885	0	0	0	0	14.9886	0	0	65.6268	0	0	9.40638	0	conserved protein, unknown function
PVX_092890	0	0	0	0	0	0	0	0	0	0	31.9893	130.211	bicoid-interacting protein BIN3, putative
PVX_092895	0	21.7734	0	0	0	159.538	0	0	0	21.9242	18.628	50.4831	hypothetical protein, conserved
PVX_092897	0	0	0	0	0	0	0	0	0	0	0	0	conserved Plasmodium protein, unknown function
PVX_092900	0	0	125.037	0	15.9498	0	0	0	76.2075	0	40.0357	162.833	WD domain, G-beta repeat domain containing protein
PVX_092905	0	0	0	0	0	0	0	0	0	0	0	0	hypothetical protein
PVX_092910	0	210.506	0	0	0	0	0	418.43	0	105.472	119.69	0	hypothetical protein, conserved
PVX_092915	0	0	0	0	0	0	0	0	0	0	0	0	hypothetical protein
PVX_092920	0	0	0	0	0	0	0	0	0	0	0	0	leucine-rich repeat protein (LRR10)
PVX_092925	0	0	0	0	0	0	0	41.9125	0	21.2144	12.0165	0	CCAAT-box DNA binding protein subunit B, putative
PVX_092930	0	0	0	0	0	0	0	0	0	0	0	0	kinesin-like protein, putative
PVX_092935	0	0	0	0	0	0	0	0	0	0	2.1128	0	hypothetical protein, conserved
PVX_092940	149.062	0	0	0	23.6548	0	114.057	0	0	0	29.6713	120.757	hypothetical protein
PVX_092945	0	0	0	0	0	0	0	0	0	0	1.83355	0	sporozoite asparagine-rich protein,sporozoite and liver stage asparagine-rich protein (SLARP)
PVX_092947	0	0	0	0	0	0	0	0	0	0	0	0	conserved Plasmodium protein, unknown function
PVX_092950	0	0	0	0	0	0	0	0	0	0	0	16.8561	tubulin--tyrosine ligase, putative
PVX_092955	0	0	0	0	0	0	0	0	0	0	4.20434	0	hypothetical protein, conserved
PVX_092958	0	1320.15	4821.05	0	0	0	0	2613.15	0	0	0	0	conserved Plasmodium protein, unknown function
PVX_092962	0	0	0	96.3268	19.1207	0	0	0	0	10.5965	12.0044	48.7976	farnesyltransferase beta subunit, putative
PVX_092965	0	0	0	0	19.6266	0	94.6254	0	23.4389	43.4664	110.816	0	hypothetical protein, conserved
PVX_092970	0	0	0	0	34.0447	0	0	0	81.3272	56.5622	74.768	0	hypothetical protein, conserved
PVX_092975	0	0	0	0	0	0	0	0	0	0	3.2079	0	merozoite adhesive erythrocytic binding protein (MAEBL)
PVX_092980	0	137.951	0	0	0	0	0	0	0	0	39.2653	0	hypothetical protein, conserved
PVX_092985	0	0	0	64.1692	0	0	0	27.8974	22.8351	7.0613	19.9975	0	serine/threonine protein kinase, putative
PVX_092990	20.3221	340.367	100.982	0	12.8826	207.789	62.092	56.4283	123.17	92.839	32.3592	0	tryptophan-rich antigen (Pv-fam-a)
PVX_092995	0	885.948	0	0	50.3333	1218.16	0	110.139	120.183	55.7072	142.039	0	tryptophan-rich antigen (Pv-fam-a)
PVX_093495	0	0	0	0	0	0	0	0	0	0	0	0	hypothetical protein, conserved
PVX_093500	0	0	0	0	0	0	0	0	0	0	0	0	gamete release protein, putative (GAMER)
PVX_093505	0	0	0	0	0	0	0	0	0	0	0	0	hypothetical protein, conserved
PVX_093510	45.0904	31.4768	0	288.191	42.9018	0	137.878	62.6165	68.3338	126.747	80.7757	72.9965	hypothetical protein, conserved
PVX_093515	74.9051	0	0	0	47.5477	0	0	208.101	56.7704	78.9465	89.4611	0	GTPase-activating protein, putative
PVX_093520	0	0	381.01	0	0	784.193	0	0	173.783	107.308	60.8302	248.089	cyclophilin, putative,peptidyl-prolyl cis-trans isomerase precursor, putative
PVX_093525	0	0	0	0	0	0	0	0	5.44852	0	2.86296	0	hypothetical protein
PVX_093530	53.2459	37.1739	0	0	16.8905	0	0	0	100.872	56.1248	42.3941	0	pseudouridylate synthase 1, putative
PVX_093535	0	8.25097	0	0	7.49378	0	0	0	8.95823	8.31185	24.7137	0	hypothetical protein, conserved
PVX_093540	47.787	0	0	0	15.1569	0	219.204	0	18.1055	16.7907	76.0949	0	methionine aminopeptidase 1c, putative (METAP1c)
PVX_093545	0	0	0	155.468	0	0	74.3806	0	36.8606	17.0918	58.0949	78.7575	hypothetical protein, conserved
PVX_093550	0	138.038	0	0	12.5419	0	0	0	44.9521	55.5892	55.1065	0	cactin homolog, putative
PVX_093555	630.504	110.196	393.143	505.325	100.26	0	241.862	219.136	179.299	332.123	313.796	767.969	proteasome subunit beta type-4, putative
PVX_093560	77.4327	0	192.693	0	24.5776	0	0	0	58.6871	0	107.894	125.469	tubulin gamma chain, putative
PVX_093565	17.6281	0	0	0	0	0	0	24.4733	6.67751	18.5845	28.0694	0	hypothetical protein, conserved
PVX_093570	0	0	0	0	0	0	0	0	0	0	0	0	AAA family ATPase, putative
PVX_093575	0	0	74.3406	0	9.48362	0	0	0	11.3321	0	17.8621	0	DNA repair protein RAD54, putative (RAD54)
PVX_093580	0	69.8714	0	0	31.7645	0	153.189	0	37.9089	70.2686	59.7301	0	mitogen-activated protein kinase organizer 1, putative
PVX_093585	0	0	0	0	0	0	79.7186	0	0	0	10.3763	0	SF-assemblin, putative
PVX_093590	0	24.4891	87.206	112.09	33.3739	0	0	48.7174	79.7508	36.9854	69.8346	113.566	hypothetical protein, conserved
PVX_093595	0	122.218	145.125	0	37.0234	0	0	0	44.2177	82.0032	116.144	0	peptidyl-prolyl cis-trans isomerase, putative
PVX_093600	0	0	0	0	0	0	0	0	0	0	30.8898	0	hypothetical protein, conserved
PVX_093605	0	0	0	0	38.0673	0	91.7655	0	90.9262	42.1556	47.7657	0	serine/threonine protein phosphatase 2B catalytic subunit A, putative (CNA)
PVX_093607	0	0	0	0	0	0	0	166.323	0	0	23.8247	0	conserved Plasmodium protein, unknown function
PVX_093610	16.3078	0	0	0	0	0	0	0	0	0	0	0	adenylyl cyclase beta, putative
PVX_093615	24.2677	0	0	0	0	0	0	0	27.5791	17.0557	24.1513	0	inositol 5-phosphatase, putative
PVX_093620	0	98.4196	0	0	0	0	161.635	0	26.7093	49.5469	0	0	hypothetical protein, conserved
PVX_093625	29.7938	41.5889	0	190.343	0	0	45.5297	82.7363	33.8605	31.4084	11.8605	0	hypothetical protein, conserved
PVX_093630	480.658	0	299.392	384.823	114.543	0	0	333.988	409.953	295.444	310.969	0	1-cys peroxiredoxin (1-CysPrx)
PVX_093635	11.8507	0	0	0	3.75543	0	18.0994	32.9031	4.48886	0	7.0762	0	transcription factor with AP2 domain(s), putative (ApiAP2)
PVX_093640	0	53.054	62.9659	0	8.03269	0	0	0	57.595	17.809	35.3054	0	NAD-specific glutamate dehydrogenase, putative
PVX_093645	0	0	0	27.1379	5.38725	0	12.9816	11.8006	0	8.96235	10.1517	13.7476	hypothetical protein, conserved
PVX_093650	0	52.1703	0	0	0	0	0	0	42.4737	26.2628	14.8768	0	mannose-6-phosphate isomerase, putative
PVX_093655	19.095	0	0	0	0	0	0	0	7.23324	0	7.60131	0	sentrin-specific protease 2, putative (SENP2)

Gene ID / Patient	Patient 1	Patient 2	Patient 3	Patient 4	Patient 5	Patient 6	Patient 7	Patient 8	Patient 9	Patient 10	Patient 11	Patient 12	Gene Description
PVX_093660	0	100.284	0	0	45.6136	0	0	398.868	108.79	50.3879	57.1237	0	hypothetical protein, conserved
PVX_093665	0	41.061	0	376.019	18.6579	0	89.953	0	133.699	41.3248	35.118	0	hypothetical protein, conserved
PVX_093670	0	0	0	0	0	0	0	0	0	0	0	0	protein kinase, putative
PVX_093675	0	0	0	0	0	0	0	0	0	0	0	0	von Willebrand factor A domain-related protein, putative (WARP)
PVX_093680	1256.74	2424.86	367.386	629.624	359.323	2393.91	376.506	1129.03	1568.25	1844.44	774.951	438.567	Phist protein (Pf-fam-b)
PVX_093682	0	0	0	0	0	0	0	0	0	0	0	0	Plasmodium exported protein, unknown function
PVX_093685	339.512	681.372	105.506	135.612	94.2117	434.217	194.639	648.262	418.044	402.624	464.601	412.194	Plasmodium exported protein, unknown function
PVX_093695	0	401.697	0	0	0	0	0	0	0	67.3351	76.3133	0	ring-exported protein 3, putative
PVX_093700	0	131.004	0	0	59.6171	0	0	0	0	65.7716	0	0	Plasmodium exported protein, unknown function
PVX_093705	0	55.7674	0	0	0	0	122.22	0	0	28.0523	63.579	0	variable surface protein Vir18, putative, pseudogene,PIR protein, pseudogene
PVX_093710	0	0	0	0	0	0	0	99.7876	0	0	0	0	variable surface protein Vir24-related,PIR protein
PVX_093715	0	0	0	0	0	0	0	0	0	0	42.0882	0	variable surface protein Vir5-like,PIR protein
PVX_093720	0	0	0	0	0	0	0	0	0	0	0	0	variable surface protein Vir24-like,PIR protein
PVX_093725	0	0	0	0	0	0	0	0	0	0	0	0	variable surface protein Vir24-related,PIR protein
PVX_093730	0	43.0127	0	0	0	0	0	0	0	0	0	0	VIR protein,PIR protein
PVX_093735	0	0	0	0	0	0	0	0	0	0	0	0	VIR protein,PIR protein
PVX_094230	0	0	0	0	0	0	0	0	0	3.62974	2.05575	8.35216	hypothetical protein
PVX_094235	0	0	0	0	0	0	0	0	0	0	0	0	hypothetical protein
PVX_094240	0	0	0	0	0	0	0	0	0	0	0	0	variable surface protein Vir12/24-related,PIR protein
PVX_094243	0	0	0	0	0	0	0	0	0	0	0	0	VIR protein, pseudogene,PIR protein, pseudogene
PVX_094245	0	0	0	0	0	0	0	0	0	17.2228	0	0	variable surface protein Vir12-like,PIR protein
PVX_094247	0	0	0	0	0	0	0	0	0	0	0	0	Plasmodium exported protein, unknown function
PVX_094250	0	0	0	0	0	0	0	0	0	0	0	0	VIR protein,PIR protein
PVX_094255	0	0	0	0	0	0	0	0	4.12504	0	0	0	reticulocyte binding protein 2b (RBP2b)
PVX_094260	0	67.1656	0	0	0	0	0	0	0	0	0	0	VIR protein, pseudogene,PIR protein, pseudogene
PVX_094265	0	0	0	0	7.53926	0	0	0	9.00976	0	4.73399	0	cytoadherence-linked asexual protein (CLAG), putative
PVX_094270	0	0	0	0	0	0	0	0	0	81.0198	0	0	Plasmodium exported protein, unknown function
PVX_094275	138.695	258.192	229.896	0	14.663	236.538	282.746	449.416	227.708	259.91	119.63	0	hypothetical protein
PVX_094277	0	218.688	0	0	18.061	145.663	0	79.1011	43.1639	50.0484	113.395	0	Plasmodium exported protein, unknown function
PVX_094280	147.223	257.028	0	0	23.3625	0	0	0	55.7893	77.5833	87.9157	0	Plasmodium exported protein, unknown function
PVX_094285	120.326	252.237	0	0	0	0	184.409	0	91.2234	126.789	23.9529	0	Pv-fam-h protein
PVX_094290	0	0	0	0	0	579.469	0	157.07	42.8443	79.4047	22.5005	0	Pv-fam-h protein
PVX_094295	0	0	0	0	0	0	0	0	0	84.0742	71.4741	194.16	Pv-fam-h protein
PVX_094300	60.1281	209.831	0	96.0354	9.53144	153.744	45.9431	83.487	34.1677	84.5154	53.8562	97.2999	Plasmodium exported protein, unknown function
PVX_094303	312.98	273.224	584.157	250.282	99.3438	0	239.511	326.08	148.258	219.908	311.502	126.789	Pvstp1, putative
PVX_094305	0	0	0	0	0	0	0	0	28.1746	0	0	0	tryptophan-rich antigen
PVX_094310	0	0	0	0	0	0	0	0	0	0	0	0	hypothetical protein, conserved
PVX_094315	0	489.958	0	374.235	37.1309	599.213	0	162.409	88.6004	369.446	162.852	0	RNA-binding protein, putative
PVX_094325	0	0	0	0	0	0	0	0	151.611	70.174	0	0	hypothetical protein, conserved
PVX_094330	0	0	0	0	20.8383	0	100.47	0	24.8843	69.2172	65.3595	0	hypothetical protein, conserved
PVX_094335	0	0	84.9069	109.135	0	0	0	0	12.9416	12.0038	47.5966	0	hypothetical protein, conserved
PVX_094340	0	12.0592	42.9312	0	0	0	0	23.9912	13.092	18.2185	27.5166	0	DnaJ protein, putative
PVX_094345	177.652	0	0	0	0	0	0	0	134.737	249.52	106.101	0	hypothetical protein, conserved
PVX_094350	0	18.0528	0	0	0	0	0	0	9.79896	9.08977	10.2972	0	hypothetical protein, conserved
PVX_094355	0	0	0	0	0	0	0	0	0	0	0	0	conserved Plasmodium protein, unknown function
PVX_094360	194.653	0	0	0	0	0	299.031	0	0	68.3442	0	0	hypothetical protein, conserved
PVX_094365	0	0	0	0	0	0	0	0	56.1059	0	0	0	N-acetyltransferase, putative
PVX_094370	0	12.6838	0	0	0	0	0	0	0	0	0	0	hypothetical protein, conserved
PVX_094375	439.228	736.287	0	0	139.449	0	268.981	488.166	332.92	617.195	297.268	0	40S ribosomal protein S20e, putative
PVX_094380	0	31.6971	0	0	0	0	0	0	0	0	0	0	inner membrane complex protein 1c, putative (IMC1c)
PVX_094385	24.0913	16.8132	0	76.9434	15.2735	0	0	0	36.5048	33.8635	28.771	0	hypothetical protein, conserved
PVX_094390	22.4815	47.0681	0	0	21.3785	0	34.3473	0	34.0651	23.7007	40.2726	36.372	U5 small nuclear ribonuclear protein, putative
PVX_094395	0	0	0	0	0	0	0	0	0	0	31.5463	0	hypothetical protein, conserved
PVX_094400	517.484	813.687	0	0	205.578	0	594.985	719.219	490.434	499.817	489.321	209.935	60S ribosomal protein L13a, putative
PVX_094405	11.2179	15.6552	0	0	3.55484	0	17.1326	0	12.7474	19.7115	13.3967	0	hypothetical protein, conserved
PVX_094410	0	0	0	0	0	0	0	0	0	0	5.43615	0	E3 ubiquitin-protein ligase, putative
PVX_094415	112.676	131.083	0	120.001	95.2778	0	229.64	260.768	142.292	118.778	112.138	60.7907	RNA-binding protein, putative
PVX_094420	0	0	0	0	0	0	0	109.171	0	0	15.6435	0	hypothetical protein, conserved
PVX_094425	30.1248	0	0	0	0	0	0	0	0	0	0	0	hypothetical protein, conserved
PVX_094430	0	0	0	0	0	0	0	0	0	0	29.5082	0	hypothetical protein, conserved
PVX_094435	0	0	0	0	0	0	0	0	41.2288	76.4145	21.6526	0	ADP/ATP carrier protein, putative
PVX_094440	0	0	0	0	0	0	0	0	43.9555	81.4612	46.1674	0	hypothetical protein, conserved

Gene ID / Patient	Patient 1	Patient 2	Patient 3	Patient 4	Patient 5	Patient 6	Patient 7	Patient 8	Patient 9	Patient 10	Patient 11	Patient 12	Gene Description
PVX_094445	43.7887	91.7028	0	139.931	13.8873	0	0	0	49.7704	15.3861	78.4437	0	methionine--tRNA ligase, putative
PVX_094450	26.8849	75.054	0	0	0	0	0	0	20.3693	47.2372	16.0537	0	hypothetical protein, conserved
PVX_094455	0	0	0	0	0	0	0	0	0	0	61.9257	0	hypothetical protein
PVX_094460	0	0	0	0	0	0	0	0	0	0	0	0	hypothetical protein, conserved
PVX_094462	0	0	0	0	0	0	0	0	0	0	0	0	conserved Plasmodium protein, unknown function
PVX_094465	40.5245	0	50.3421	0	0	0	0	0	15.351	7.12044	24.198	0	regulator of nonsense transcripts, putative
PVX_094470	0	31.7327	0	0	0	0	0	0	86.1116	0	45.2401	0	hypothetical protein
PVX_094480	0	0	0	0	0	0	0	0	16.6881	30.954	0	0	Plasmodium exported protein, unknown function
PVX_094485	0	0	0	0	0	0	0	0	0	0	0	0	hypothetical protein, conserved
PVX_094490	0	0	155.809	0	0	0	0	0	0	22.0069	99.7441	0	hypothetical protein, conserved
PVX_094495	0	0	0	0	0	0	0	0	0	0	0	0	hypothetical protein, conserved
PVX_094500	0	0	0	0	0	0	0	0	0	0	0	0	CCR4-NOT transcription complex subunit 5, putative
PVX_094505	253.61	0	315.995	0	80.5948	0	0	0	144.209	89.0744	100.971	0	DNA/RNA-binding protein Alba 3, putative (ALBA3)
PVX_094510	0	0	0	0	0	0	0	0	0	0	0	0	hypothetical protein
PVX_094515	65.4011	0	0	0	0	0	0	0	0	0	6.50882	0	hypothetical protein, conserved
PVX_094520	0	0	337.598	0	0	0	0	0	0	0	0	0	hypothetical protein, conserved
PVX_094525	0	0	0	0	75.2905	0	0	657.476	179.276	248.828	94.0857	0	phosducin-like protein, putative (PhLP1)
PVX_094530	61.1136	21.3272	0	0	9.68777	0	46.6968	84.8557	11.5759	32.2126	30.4105	49.448	hypothetical protein
PVX_094535	345.39	826.711	122.69	630.799	156.505	757.417	301.794	616.705	579.533	485.429	392.852	559.216	RNA-binding protein, putative
PVX_094540	0	0	0	0	0	0	0	0	0	0	40.608	0	PPPDE peptidase, putative
PVX_094545	0	0	0	0	25.9024	0	0	113.355	0	28.6659	0	0	conserved membrane protein, unknown function
PVX_094550	0	0	0	0	0	0	0	0	0	67.7553	0	0	dynein light chain 2B, cytoplasmic, putative
PVX_094555	0	0	0	0	0	0	0	0	0	0	0	0	Rho-GTPase-activating protein 1, putative
PVX_094560	0	0	0	0	0	0	0	0	0	0	0	0	RING zinc finger protein, putative
PVX_094565	99.6855	0	496.45	0	0	0	0	0	37.7822	35.0169	79.3739	161.628	hypothetical protein, conserved
PVX_094570	0	224.106	0	0	0	0	0	0	121.429	0	318.486	0	hypothetical protein
PVX_094575	697.405	490.924	0	0	0	0	0	0	265.404	244.256	416.803	0	hypothetical protein, conserved
PVX_094580	0	63.3625	0	0	7.19488	116.05	0	0	8.59837	7.97637	13.5536	0	transcription factor with AP2 domain(s), putative (ApiAP2)
PVX_094585	0	0	0	0	0	0	0	49.2974	0	0	14.1332	0	hypothetical protein, conserved
PVX_094590	162.438	170.083	0	0	0	0	62.0791	112.783	138.468	128.424	137.413	0	eukaryotic translation initiation factor 3 subunit 7, putative
PVX_094595	13.9447	19.4612	0	0	0	0	0	19.3589	15.8463	14.7015	13.8776	0	histone deacetylase 2, putative (HDA2)
PVX_094600	16.8907	29.4639	0	0	13.3804	0	25.7942	35.1713	9.5967	26.712	20.1713	27.3161	hypothetical protein, conserved
PVX_094605	0	0	0	0	0	0	0	0	166.134	92.3979	139.617	142.104	endonuclease, putative
PVX_094610	0	0	0	0	0	0	0	0	71.9442	0	0	0	hypothetical protein
PVX_094615	144.519	100.89	479.122	153.96	61.1175	246.484	0	133.798	146.014	169.262	191.773	0	26S protease regulatory subunit 4, putative
PVX_094620	0	0	0	0	21.6628	0	0	0	0	24.0076	13.599	0	hypothetical protein, conserved
PVX_094625	0	0	0	0	0	0	0	0	0	0	0	0	hypothetical protein, conserved
PVX_094635	78.8657	55.0787	785.068	504.542	50.0666	0	0	328.669	89.6607	83.1191	408.164	127.796	tubulin beta chain, putative
PVX_094640	0	186.871	0	0	0	0	0	74.3462	81.1325	75.2358	42.6223	86.684	nucleolar protein 5, putative (NOP5)
PVX_094645	173.24	483.822	430.873	0	91.6015	591.11	0	401.011	262.566	243.47	91.9558	187.038	RAD protein (Pv-fam-e)
PVX_094650	0	0	0	0	0	0	0	0	0	0	0	0	RAD protein (Pv-fam-e)
PVX_094655	0	111.375	0	0	0	0	0	0	60.4053	0	31.7148	0	RAD protein (Pv-fam-e)
PVX_094660	170.218	119.018	0	0	54.1514	0	261.288	0	129.092	179.316	101.661	0	adenylate kinase 2, putative
PVX_094665	0	159.727	0	0	0	0	0	79.4329	21.6707	60.2843	11.3843	0	diphthine synthase, putative
PVX_094670	0	0	0	0	0	0	0	0	0	0	0	0	hypothetical protein, conserved
PVX_094675	36.7265	51.2706	91.29	0	11.6456	0	56.1363	0	55.6552	51.6205	58.4815	0	hypothetical protein, conserved
PVX_094680	115.568	0	0	184.727	0	0	0	80.254	43.7892	0	23.0038	0	hypothetical protein, conserved
PVX_094685	0	46.8537	166.928	0	42.5843	0	0	0	177.979	117.87	53.4246	0	zinc finger protein, putative
PVX_094690	0	65.751	0	0	0	0	0	130.778	107.024	99.1976	37.4743	0	hypothetical protein, conserved
PVX_094695	0	0	0	0	0	0	0	0	0	22.7882	51.6433	0	protein phosphatase 2C, putative
PVX_094700	0	0	0	0	0	0	0	0	0	0	0	0	tubulin--tyrosine ligase, putative
PVX_094705	0	0	0	0	0	0	0	0	8.53773	0	17.944	36.4637	hypothetical protein, conserved
PVX_094710	0	6.50825	0	0	0	0	0	0	0	0	7.42594	0	kinesin-4, putative
PVX_094715	84.12	0	0	134.4	13.3385	0	0	116.813	47.805	73.8943	33.4874	0	hypothetical protein, conserved
PVX_094720	0	0	0	0	0	0	0	154.722	84.4084	39.1099	66.4936	0	ribosomal protein L43, mitochondrial, putative
PVX_094725	0	0	0	0	0	0	0	0	49.5452	0	26.017	0	hypothetical protein, conserved
PVX_094730	31.5353	11.003	0	0	14.9916	0	0	21.8901	59.7271	22.1644	28.2454	0	hypothetical protein, conserved
PVX_094735	176.252	246.493	0	0	0	905.297	0	0	66.8372	185.667	0	0	hypothetical protein, conserved
PVX_094740	0	0	0	0	0	0	0	0	0	0	0	0	hypothetical protein, conserved
PVX_094745	0	0	0	0	0	0	0	0	0	0	0	0	hypothetical protein, conserved
PVX_094750	73.9689	154.893	275.796	118.165	23.4549	0	169.593	0	238.197	220.928	88.3386	59.8602	eukaryotic translation initiation factor 2 beta subunit, putative
PVX_094755	0	64.998	0	0	29.5466	0	0	129.281	35.2663	32.6878	0	150.845	hypothetical protein, conserved

Gene ID / Patient	Patient 1	Patient 2	Patient 3	Patient 4	Patient 5	Patient 6	Patient 7	Patient 8	Patient 9	Patient 10	Patient 11	Patient 12	Gene Description
PVX_094760	131.269	0	327.161	0	41.7206	0	0	0	149.29	92.208	78.3946	213.026	50S ribosomal protein L22, mitochondrial, putative
PVX_094765	107.596	0	0	0	0	0	329.663	0	40.7824	0	42.8366	0	hypothetical protein, conserved
PVX_094770	0	0	0	0	0	0	0	0	0	0	0	0	inner membrane complex sub-compartment protein 1, putative (ISP1)
PVX_094775	0	0	0	0	0	0	0	0	29.8493	0	39.2058	0	hypothetical protein, conserved
PVX_094780	0	0	0	0	0	0	0	0	39.316	109.31	41.2972	0	SNARE domain containing protein
PVX_094785	0	39.2046	139.653	0	0	0	0	0	0	0	44.7085	0	hypothetical protein, conserved
PVX_094790	289.268	0	360.607	2317.53	137.951	0	887.339	201.048	274.176	203.179	374.305	0	proteasome subunit beta type-5, putative
PVX_094795	0	0	0	0	0	0	0	0	0	96.7152	164.604	0	hypothetical protein, conserved
PVX_094800	0	0	0	0	0	0	0	0	47.4686	22.0069	12.468	0	hypothetical protein, conserved
PVX_094805	63.7074	44.4837	158.476	0	0	0	0	88.4859	168.98	22.3828	76.0862	0	DNA repair protein RAD23, putative
PVX_094810	65.7918	153.048	0	0	41.7086	112.123	33.5051	30.4479	124.613	61.6533	139.682	35.4802	PRE-binding protein, putative (PREBP)
PVX_094815	109.841	76.7417	0	0	0	0	0	0	0	38.5822	0	0	heme oxygenase, putative (HO)
PVX_094820	0	0	0	0	23.4065	0	0	51.2499	0	0	7.34637	0	hypothetical protein, conserved
PVX_094825	0	0	210.788	0	0	0	0	0	0	29.7505	33.715	0	hypothetical protein, conserved
PVX_094830	0	0	0	0	0	0	0	0	0	0	0	0	hypothetical protein, conserved
PVX_094835	62.3319	43.5224	155.048	199.291	79.1087	0	95.3518	86.5742	118.093	87.5987	136.479	100.958	ubiquinol-cytochrome c reductase complex subunit, putative
PVX_094840	1370.73	441.98	787.794	1350.12	200.943	0	484.557	732.554	879.229	1703.72	440.774	170.987	hypoxanthine-guanine phosphoribosyltransferase, putative
PVX_094845	0	0	0	0	16.4115	0	0	71.8521	58.8083	18.1784	30.8948	0	phosphoglucomutase, putative
PVX_094850	49.4505	34.5225	0	0	31.3702	0	75.6156	137.349	206.095	191.126	88.587	0	GMP synthetase, putative
PVX_094855	21.3041	29.735	0	0	0	0	32.5473	29.5779	16.1404	14.9731	12.7211	0	NLI interacting factor-like phosphatase, putative (NIF4)
PVX_094860	42.7226	0	0	0	13.5489	0	0	118.654	80.9305	45.0349	85.0375	0	hypothetical protein, conserved
PVX_094865	67.5546	94.3446	168.064	0	64.3111	345.855	103.358	93.833	51.1969	71.202	13.4469	109.433	autophagy-related protein 18, putative (ATG18)
PVX_094870	0	0	0	0	0	0	0	0	48.9069	0	12.8457	0	hypothetical protein, conserved
PVX_094875	25.2325	17.6098	0	0	15.9974	0	0	105.1	19.1171	44.3341	15.0669	0	hypothetical protein, conserved
PVX_094880	0	0	0	0	17.248	0	0	37.7708	0	9.55942	10.8293	0	hypothetical protein, conserved
PVX_094885	84.7975	0	105.406	135.484	40.3382	216.902	64.818	58.8771	64.2535	59.5915	42.1964	0	hypothetical protein, conserved
PVX_094890	0	0	0	0	0	0	0	0	0	0	0	0	hypothetical protein, conserved
PVX_094895	43.7006	15.2487	0	0	0	0	0	30.3363	8.27712	15.3569	21.7454	0	phospholipase C-like, putative
PVX_094900	0	0	0	0	0	0	0	0	5.72941	0	18.0633	0	hypothetical protein, conserved
PVX_094902	0	0	0	0	0	0	0	0	0	0	85.2998	0	hypothetical protein, conserved
PVX_094905	0	0	0	0	0	0	0	0	0	0	0	0	hypothetical protein, conserved
PVX_094910	29.9133	20.8779	0	0	0	0	0	0	33.9964	10.5115	23.8161	0	initiation factor 2 subunit family, putative
PVX_094915	0	0	0	0	0	0	0	0	0	0	0	0	GDP-L-fucose synthase, putative (FS)
PVX_094920	58.2544	0	0	0	0	0	0	0	0	0	0	31.4132	hypothetical protein, conserved
PVX_094925	0	0	0	0	0	0	0	0	14.2113	13.1809	0	0	male gamete fusion factor HAP2, putative (HAP2)
PVX_094930	0	8.56176	0	39.1747	15.5533	0	0	51.1006	32.5336	17.2479	48.8434	0	hypothetical protein, conserved
PVX_094935	0	0	0	0	20.3595	0	0	178.241	0	0	76.6318	0	cyclin dependent kinase 7 (cdk7), putative
PVX_094940	0	0	0	0	0	0	0	0	7.9009	21.9886	4.15143	0	hypothetical protein, conserved
PVX_094945	33.7552	39.256	55.8962	35.923	14.2623	0	17.1843	0	46.8816	31.6335	49.2693	18.198	transcriptional coactivator ADA2, putative (ADA2)
PVX_094950	77.8368	0	193.701	248.973	0	0	238.258	108.126	88.4903	136.725	77.469	0	prohibitin, putative
PVX_094955	0	0	0	0	0	0	0	0	0	0	0	0	hypothetical protein, conserved
PVX_094965	0	0	0	0	0	0	0	0	0	0	0	0	hypothetical protein, conserved
PVX_094970	111.622	0	0	0	0	0	0	0	0	0	44.4396	181.042	FAD synthetase, putative
PVX_094975	56.9023	0	0	0	0	0	0	0	0	0	45.3055	0	hypothetical protein, conserved
PVX_094980	211.193	88.4549	0	0	26.7905	216.082	0	58.6547	96.0162	89.0497	126.111	0	cysteine--tRNA ligase, putative (CysRS)
PVX_094985	383.185	107.041	0	0	24.3246	0	234.577	0	87.1256	134.619	106.785	0	methionine aminopeptidase 1b, putative (METAP1b)
PVX_094990	47.2571	32.9902	0	0	14.9886	0	72.2565	0	17.9046	66.4182	94.0638	0	hypothetical protein, conserved
PVX_094995	0	0	0	0	0	0	0	0	0	0	35.6094	96.5776	hypothetical protein, conserved
PVX_095000	116.244	40.5806	0	0	73.7578	0	0	0	154.159	306.314	80.9839	0	heat shock protein 60, putative
PVX_095005	0	0	0	0	0	0	0	0	42.1517	39.0613	44.274	0	hypothetical protein
PVX_095010	111.205	0	0	0	0	0	0	0	42.1517	39.0613	110.685	180.365	ribonucleotide reductase small subunit, putative
PVX_095015	4184.98	2267.91	3573.59	3195.34	1387.31	3197.36	2866.56	4251.02	4118.24	5640	3431.56	2225.72	enolase, putative
PVX_095020	0	0	0	0	0	0	0	0	0	0	0	0	hypothetical protein, conserved
PVX_095025	0	0	0	0	0	0	90.8502	82.4924	22.5052	0	0	0	hypothetical protein, conserved
PVX_095030	259.438	144.9	129.031	165.849	32.9182	0	158.697	0	117.958	72.9245	103.281	0	hypothetical protein, conserved
PVX_095035	0	0	0	0	0	0	0	0	11.8429	0	12.4447	0	hypothetical protein, conserved
PVX_095040	32.7724	22.8742	0	0	10.3908	0	50.0862	45.5051	0	34.5479	26.0925	0	hypothetical protein, conserved
PVX_095045	0	52.1921	0	119.449	23.7099	382.461	114.292	103.828	14.1638	39.4107	96.7396	60.5111	hypothetical protein, conserved
PVX_095050	108.427	113.55	134.824	0	17.1979	277.442	82.9114	0	102.706	114.289	97.1203	0	survival motor neuron-like protein, putative (SMN)
PVX_095055	0	0	0	0	9.38007	0	0	0	0	0	5.88906	0	Rh5 interacting protein, putative (RIPR)
PVX_095060	0	65.6818	0	0	0	0	0	0	35.6371	33.0311	18.7175	0	conserved protein, unknown function
PVX_095065	0	0	0	0	0	0	0	0	28.3377	0	14.8853	0	hypothetical protein, conserved

Gene ID / Patient	Patient 1	Patient 2	Patient 3	Patient 4	Patient 5	Patient 6	Patient 7	Patient 8	Patient 9	Patient 10	Patient 11	Patient 12	Gene Description
PVX_095070	0	15.5681	0	0	0	114.053	0	0	0	31.3568	44.4015	0	hypothetical protein, conserved
PVX_095075	0	178.594	638.12	0	0	0	0	355.046	0	0	101.599	415.503	translation machinery-associated protein 7, putative (TMA7)
PVX_095080	1378.58	1219.73	1372.81	1176.36	262.635	1412.59	1547.97	1532.24	1602.25	1872.54	1372.09	595.923	40S ribosomal protein S3A, putative
PVX_095085	0	0	0	0	0	0	0	0	0	0	0	0	hypothetical protein
PVX_095095	0	15.546	0	0	3.53006	0	0	0	0	3.91482	6.65164	0	hypothetical protein, conserved
PVX_095100	0	0	0	0	0	0	0	0	0	0	8.03144	0	hypothetical protein, conserved
PVX_095105	0	0	268.954	0	0	0	0	150.05	40.9301	37.9308	107.479	0	iron-sulfur assembly protein, putative
PVX_095115	0	59.9898	0	0	13.627	0	65.6906	0	0	15.0981	17.1055	0	D123 (regulator of eIF2), putative
PVX_095120	0	0	0	0	37.6878	0	0	0	22.5052	62.6042	94.5802	96.1923	diacylglycerol O-acyltransferase, putative (DGAT)
PVX_095125	0	0	0	0	0	0	0	0	0	0	0	0	hypothetical protein, conserved
PVX_095130	0	42.3993	0	0	0	0	0	84.3406	46.0186	0	60.4364	0	mRNA capping enzyme, putative
PVX_095135	326.167	285.047	813.636	0	285.3	418.664	500.56	1360.41	1082.16	658.592	616.859	397.341	cyclophilin, putative
PVX_095140	25.6226	17.8822	0	0	16.2449	0	0	0	29.1191	18.0078	20.3998	0	cyclic amine resistance locus protein, putative (CARL)
PVX_095145	0	16.5582	0	0	7.51983	0	18.121	0	13.4826	8.33928	16.5308	0	hypothetical protein, conserved
PVX_095150	16.4306	11.4657	0	104.93	0	0	0	45.6211	6.22386	11.5481	42.5143	0	hypothetical protein, conserved
PVX_095155	42.9687	0	0	0	13.627	0	0	0	16.2794	15.0981	8.55275	0	ATP-dependent RNA helicase, putative
PVX_095160	186.853	26.0851	0	0	23.7	0	57.122	259.461	84.9473	65.6571	200.837	60.4858	hypothetical protein, conserved
PVX_095165	0	0	0	0	0	0	0	0	0	0	45.6351	0	protein kinase, putative
PVX_095170	0	0	71.1967	0	0	0	0	39.7785	21.7063	30.202	45.6193	46.3587	hypothetical protein, conserved
PVX_095175	0	49.1787	0	0	0	0	107.76	0	53.3735	24.7422	0	0	N-acetylglucosamine-1-phosphate transferase, putative
PVX_095180	0	39.4209	0	0	23.8724	0	57.5298	0	14.2656	0	11.2436	0	hypothetical protein, conserved
PVX_095185	0	0	0	0	0	0	0	0	22.0657	81.8432	23.1834	0	hypothetical protein, conserved
PVX_095190	1021.31	714.107	424.7	0	216.606	874.145	0	236.67	580.916	597.72	474.416	276.537	histone H2A, putative
PVX_095195	0	56.8852	0	0	25.8552	0	0	0	0	57.2276	48.6391	0	ATP-dependent RNA helicase DDX6, putative (DOZI)
PVX_095200	0	0	0	0	20.823	0	200.793	0	74.5982	92.222	26.1246	0	signal peptidase complex subunit 2, putative
PVX_095205	137.378	191.776	170.727	0	65.3378	0	157.474	143.066	169.145	181.024	211.918	55.5833	hypothetical protein, conserved
PVX_095210	117.233	0	291.583	0	74.3868	0	0	244.233	111.051	144.163	93.3412	0	nicotinamidase, putative
PVX_095215	0	0	0	0	2.83929	0	13.6837	0	0	0	0	0	oocyst capsule protein, putative (Cap380)
PVX_095220	127.415	44.4837	158.476	203.697	60.6429	652.242	97.4603	88.4859	241.4	156.68	126.81	0	T-complex protein 1, epsilon subunit, putative
PVX_095225	0	0	0	0	0	0	0	0	0	0	0	0	CPW-WPC family protein, putative
PVX_095230	0	0	0	301.222	29.8892	0	0	130.778	35.6746	33.0659	0	152.594	vesicle transport protein Sec22, putative
PVX_095235	320.858	0	532.751	0	101.916	0	163.843	148.614	162.154	225.41	170.322	173.446	protein phosphatase inhibitor 2, putative
PVX_095240	0	0	0	0	0	0	0	236.193	32.2162	0	33.8431	137.774	hypothetical protein, conserved
PVX_095245	0	0	0	0	0	0	0	104.05	0	26.3155	14.9102	0	hypothetical protein, conserved
PVX_095250	0	40.4934	0	0	7.35595	0	17.726	16.1123	13.1889	16.3152	27.7211	0	ABC transporter, putative
PVX_095255	668.466	468.387	0	0	0	0	0	0	1141.95	938.155	532.426	0	elongation factor 1, putative
PVX_095260	70.7594	0	0	0	22.4558	0	108.273	294.862	0	24.8595	28.1698	0	RNA-binding protein, putative
PVX_095265	48.9737	17.0893	60.8454	0	7.76219	0	0	67.9954	9.27605	8.60484	29.2433	0	kinesin-8, putative
PVX_095270	0	0	0	0	0	0	0	0	47.0684	43.6109	24.7173	0	ubiquitin-conjugating enzyme E2, putative
PVX_095275	0	27.6076	98.3178	0	0	0	0	109.841	74.9201	83.3838	39.3618	64.0182	endonuclease/exonuclease/phosphatase domain containing protein
PVX_095280	0	122.281	436.376	0	55.6392	0	0	0	0	0	69.6283	0	E3 ubiquitin-protein ligase RBX1, putative (RBX1)
PVX_095285	0	0	0	0	0	0	0	0	0	0	10.8597	0	potential phospholipid-transporting ATPase, putative
PVX_095290	0	116.314	207.249	0	52.8674	0	127.464	115.677	63.1126	0	116.026	0	hypothetical protein, conserved
PVX_095295	92.5707	0	0	0	0	0	141.746	0	0	32.5189	128.989	0	triosephosphate isomerase, putative
PVX_095300	0	49.3264	0	0	11.2038	0	0	0	53.5452	0	21.0993	0	RNA-metabolising metallo-beta-lactamase domain containing protein
PVX_095305	0	0	0	0	1.98493	0	0	0	0	0	2.49373	0	hypothetical protein, conserved
PVX_095310	0	0	0	0	0	0	0	0	0	0	0	0	hypothetical protein, conserved
PVX_095315	31.9647	44.6205	0	0	0	0	0	44.3833	24.2188	33.6966	31.8118	51.7283	hypothetical protein, conserved
PVX_095320	38.704	18.0049	64.0941	41.1916	16.354	0	19.7047	35.8206	39.0949	22.6694	51.3571	20.867	DNA-directed RNA polymerase II subunit RPB1, putative (RPB1)
PVX_095325	102.424	143.106	0	327.844	32.5298	0	0	0	155.283	71.9566	0	0	stomatin-like protein
PVX_095330	0	0	0	0	43.8499	0	0	0	0	0	27.461	0	hypothetical protein, conserved
PVX_095335	0	36.7149	0	0	0	0	0	0	0	0	10.4677	0	hypothetical protein, conserved
PVX_095340	0	0	0	0	101.189	0	0	147.555	120.749	74.602	126.832	0	26S proteasome regulatory subunit p27, putative
PVX_095345	0	8.35553	0	76.4619	15.1786	0	18.2884	0	22.6785	8.41627	26.2168	0	hypothetical protein, conserved
PVX_095350	352.505	1109.22	439.831	1696.01	224.318	0	270.603	1225.37	1136.23	990.225	807.034	286.39	40S ribosomal protein S11, putative
PVX_095355	0	0	0	72.7125	0	0	0	0	0	0	9.06331	0	kinesin-5 (EG5)
PVX_095360	0	45.755	0	0	0	0	0	0	0	0	65.2159	0	hypothetical protein, conserved
PVX_095365	10.307	7.19194	0	32.9061	3.26614	0	15.741	42.9251	23.4246	32.6	18.4634	0	hypothetical protein, conserved
PVX_095370	0	0	0	143.887	0	0	34.4166	0	17.0669	7.91614	17.935	0	protein kinase domain containing protein
PVX_095375	0	0	0	0	0	0	0	0	55.8616	25.9059	22.0119	0	6-cysteine protein
PVX_095380	174.833	61.0573	0	279.691	111.013	0	401.496	364.335	331.293	245.673	348.019	566.747	proteasome subunit alpha type-3, putative
PVX_095385	66.4017	46.3666	0	0	0	0	0	92.2306	50.3228	0	52.8696	0	E3 ubiquitin-protein ligase, putative

Gene ID / Patient	Patient 1	Patient 2	Patient 3	Patient 4	Patient 5	Patient 6	Patient 7	Patient 8	Patient 9	Patient 10	Patient 11	Patient 12	Gene Description
PVX_095390	669.932	2187.46	0	1434.46	640.212	0	1373.48	931.956	1185.94	1411.1	1333.79	726.673	40S ribosomal protein S15Aa, putative
PVX_095395	0	0	0	0	0	0	0	0	0	0	0	0	hypothetical protein, conserved
PVX_095400	0	130.185	0	597.252	59.2433	0	0	0	211.787	0	37.0596	0	hypothetical protein, conserved
PVX_095405	88.3661	185.164	0	282.736	28.0554	0	0	0	100.468	217.297	105.54	0	transporter, putative
PVX_095410	70.3699	0	175.082	450.083	66.9958	0	0	195.492	26.6658	0	84.0441	114.002	hypothetical protein, conserved
PVX_095415	0	0	0	0	0	0	55.1172	100.145	27.3229	0	64.5987	58.3633	hypothetical protein, conserved
PVX_095420	181.19	42.1703	150.227	193.094	38.3246	0	92.386	167.77	228.85	190.98	96.1761	293.454	inorganic pyrophosphatase, putative
PVX_095425	0	0	0	99.7801	0	0	0	0	0	16.4724	34.2087	25.2735	hypothetical protein, conserved
PVX_095430	115.961	0	0	0	0	0	177.694	0	131.867	122.192	92.3348	376.205	hypothetical protein, conserved
PVX_095435	0	0	0	0	0	0	0	0	0	0	0	0	microneme associated antigen, putative (MA)
PVX_095440	0	0	0	0	0	0	0	0	52.2971	0	82.383	0	hypothetical protein, conserved
PVX_095445	0	82.3056	0	0	37.425	0	0	163.693	44.6503	0	23.4483	0	zinc finger protein, putative
PVX_095450	0	0	0	0	0	0	0	0	10.8116	0	0	0	hypothetical protein, conserved
PVX_095452	0	14.4846	0	0	13.1577	0	31.7089	0	23.5873	7.29382	78.493	0	hypothetical protein
PVX_095455	0	0	0	0	0	0	0	0	201.705	62.2566	35.297	288.1	39s ribosomal protein L47, mitochondrial precursor, putative
PVX_095460	0	0	0	446.13	44.2606	0	213.515	0	0	0	83.1522	226.002	hypothetical protein, conserved
PVX_095470	33.3946	0	0	0	0	0	0	23.1809	31.6245	11.7355	26.5873	27.0096	hypothetical protein, conserved
PVX_095475	0	0	0	0	0	0	0	0	0	0	0	0	circumsporozoite- and TRAP-related protein, putative (CTRP)
PVX_095480	186.151	390.555	0	0	118.487	0	1143.56	0	141.191	326.808	370.596	302.558	translation initiation factor 4E, putative
PVX_095485	279.336	0	1392.71	0	177.597	0	642.553	776.543	635.408	343.364	611.686	0	zinc finger protein, putative
PVX_095490	0	0	0	0	0	0	0	0	20.1745	0	10.5985	0	hypothetical protein, conserved
PVX_095495	0	0	0	0	0	0	0	0	0	0	0	0	hypothetical protein, conserved
PVX_095990	0	0	0	0	0	0	0	0	0	0	45.6351	0	VIR protein,PIR protein
PVX_095995	0	0	0	0	0	0	0	0	0	0	0	0	variable surface protein Vir16/32-related,PIR protein
PVX_095997	0	45.9571	0	0	0	0	0	0	24.9392	46.2466	13.1007	0	VIR protein, pseudogene,PIR protein, pseudogene
PVX_096000	0	0	0	0	0	0	0	0	0	0	0	0	variable surface protein Vir4, putative,PIR protein
PVX_096001	0	0	0	0	0	0	0	0	0	0	0	0	VIR protein,PIR protein
PVX_096001a	0	0	0	0	31.6581	0	0	0	0	0	19.8435	0	VIR protein,PIR protein
PVX_096003	0	0	0	0	0	0	0	0	0	0	0	0	VIR protein,PIR protein
PVX_096004	0	0	0	0	0	0	0	0	0	0	0	0	VIR protein,PIR protein
PVX_096005	0	69.4054	0	0	0	0	0	0	0	0	19.7774	0	variable surface protein Vir15-related,PIR protein
PVX_096007	0	0	0	0	0	0	0	0	0	0	0	0	VIR protein, pseudogene,PIR protein, pseudogene
PVX_096010	0	150.342	0	0	0	0	164.831	0	40.7824	37.794	85.6731	0	Plasmodium exported protein, unknown function
PVX_096015	199.87	0	499.101	0	127.262	0	0	0	0	0	79.5832	0	Plasmodium exported protein, unknown function
PVX_096020	0	440.241	0	0	50.0225	0	0	218.919	0	110.728	47.0544	0	Plasmodium exported protein, unknown function
PVX_096030	147.01	976.218	0	0	93.4832	0	0	204.355	0	335.585	117.063	0	hypothetical protein
PVX_096035	0	47.1151	0	0	0	0	0	0	0	23.6767	0	0	hypothetical protein
PVX_096040	0	0	0	0	0	0	0	0	0	0	0	0	hypothetical protein
PVX_096045	244.012	1537	1220.18	0	0	2511.8	375.45	0	185.152	171.306	388.66	0	hypothetical protein
PVX_096050	0	233.38	0	0	35.3706	570.789	0	0	42.2042	156.44	22.1645	0	hypothetical protein
PVX_096055	0	0	0	0	0	0	0	0	31.1217	144.25	49.0409	0	hypothetical protein
PVX_096060	239.365	0	0	0	0	0	0	0	0	0	47.6494	194.16	hypothetical protein
PVX_096065	0	0	318.715	0	0	0	0	177.747	48.4822	89.8376	76.3777	0	Plasmodium exported protein, unknown function
PVX_096070	76.3754	426.702	0	0	121.207	391.117	0	530.471	231.541	536.637	182.435	123.752	early transcribed membrane protein (ETRAMP)
PVX_096071	0	0	0	0	0	0	0	0	0	0	0	0	conserved Plasmodium protein, unknown function
PVX_096075	0	0	0	0	0	188.705	0	0	27.9538	12.9636	22.03	0	hypothetical protein, conserved
PVX_096080	183.104	0	0	0	58.2693	0	0	0	347.193	128.585	145.811	297.58	mago nashi domain containing protein
PVX_096085	0	0	0	0	0	0	0	0	0	23.6591	8.93375	0	hypothetical protein, conserved
PVX_096090	0	0	0	0	0	0	0	0	8.2249	0	25.9299	0	exonuclease I, putative
PVX_096095	0	0	0	0	0	0	0	0	0	0	0	0	kinesin-19, putative
PVX_096105	0	0	0	0	0	0	0	0	0	0	0	0	kelch domain-containing protein
PVX_096110	0	5.91847	0	0	0	0	0	0	3.21285	8.94281	8.44133	13.7176	hypothetical protein, conserved
PVX_096115	0	10.4435	0	0	0	0	0	0	0	5.25943	20.8518	0	protein kinase, putative
PVX_096120	0	0	0	0	0	0	0	0	0	0	0	0	hypothetical protein, conserved
PVX_096125	0	0	0	0	0	0	0	0	233.78	215.355	122.434	0	mitochondrial import inner membrane translocase subunit TIM14, putative (PAM18)
PVX_096130	73.9763	0	0	236.599	0	0	0	0	28.0331	25.9893	14.7253	0	3-demethylubiquinone-9 3-methyltransferase, putative
PVX_096135	0	0	0	0	28	0	0	0	100.27	123.925	35.1106	0	type 2A phosphatase-associated protein 42, putative (TAP42)
PVX_096140	0	40.9345	0	0	9.29703	0	0	0	44.4371	20.6097	40.8587	0	hypothetical protein, conserved
PVX_096145	0	13.472	0	0	6.11881	0	0	0	0	27.1364	19.2123	0	Rab GTPase activator and protein kinase, putative
PVX_096150	45.8852	48.0337	0	0	7.27239	0	0	31.8532	34.7639	8.06224	27.399	74.2369	hypothetical protein, conserved
PVX_096155	17.3917	0	0	55.5355	5.51211	0	0	24.1451	0	0	10.3849	28.1334	hypothetical protein, conserved
PVX_096165	38.1838	0	94.9163	0	0	0	0	0	43.3981	40.2513	22.8008	61.8034	metallo-hydrolase/oxidoreductase, putative

Gene ID / Patient	Patient 1	Patient 2	Patient 3	Patient 4	Patient 5	Patient 6	Patient 7	Patient 8	Patient 9	Patient 10	Patient 11	Patient 12	Gene Description
PVX_096170	653.815	0	0	1053.04	0	0	504.337	0	0	229.407	130.188	0	hypothetical protein, conserved
PVX_096175	0	0	0	0	0	0	0	0	0	0	67.3892	0	dynactin subunit 5, putative
PVX_096180	21.2586	29.6714	0	0	0	0	0	29.5146	32.2118	37.3526	29.6192	34.3923	hypothetical protein
PVX_096185	0	0	0	0	0	0	0	0	0	0	0	0	hypothetical protein, conserved
PVX_096195	0	0	0	0	0	0	0	0	0	0	0	0	hypothetical protein, conserved
PVX_096200	0	0	0	0	0	0	0	0	0	0	17.5553	0	hypothetical protein, conserved
PVX_096205	0	0	0	0	0	0	0	0	38.0365	0	19.977	0	hypothetical protein, conserved
PVX_096210	0	0	0	0	0	0	188.739	0	0	43.2511	0	0	hypothetical protein, conserved
PVX_096215	0	0	0	0	0	0	0	0	23.2939	0	0	0	hypothetical protein, conserved
PVX_096220	240.085	0	0	0	0	0	0	668.084	273.25	337.105	143.401	0	hypothetical protein, conserved
PVX_096225	0	83.5618	0	0	0	204.121	121.996	110.821	120.941	42.0636	79.4256	0	hypothetical protein, conserved
PVX_096230	0	0	0	0	0	0	210.554	0	104.112	96.4493	54.6691	0	hypothetical protein, conserved
PVX_096235	91.7169	0	0	293.485	29.1217	0	140.435	127.424	139.04	64.4381	73.0282	148.675	GTP-binding protein, putative
PVX_096240	36.1042	0	89.7416	230.698	11.4481	0	0	50.133	13.678	50.746	35.9316	58.434	hypothetical protein, conserved
PVX_096245	0	0	0	0	0	0	74.993	0	0	0	19.5242	0	rhoptry-associated leucine zipper-like protein 1, putative (RALP1)
PVX_096250	0	0	196.788	252.941	25.0998	0	121.029	0	89.8984	27.7798	94.4415	128.136	PelOta protein homologue, putative
PVX_096252	172.183	120.395	429.627	0	0	0	264.321	0	65.2922	120.923	171.391	279.745	conserved Plasmodium protein, unknown function
PVX_096253	0	0	0	0	0	0	0	0	680.992	0	0	0	conserved Plasmodium protein, unknown function
PVX_096255	0	107.169	0	491.415	48.7509	0	235.2	426.241	348.758	269.189	396.751	248.943	hypothetical protein, conserved
PVX_096260	0	0	0	0	0	0	0	0	0	0	0	0	hypothetical protein, conserved
PVX_096265	1366.82	1146.33	0	875.845	477.914	0	1047.92	759.917	1139.98	912.073	1170	443.689	40S ribosomal protein S5, putative
PVX_096268	44.7745	0	0	0	0	0	0	0	0	15.7325	53.4733	144.969	conserved Plasmodium membrane protein, unknown function
PVX_096271	389.307	272.292	0	0	0	0	0	270.71	73.8245	68.3442	116.258	0	conserved Plasmodium protein, unknown function
PVX_096273	0	0	86.3588	0	33.0497	0	53.1035	0	26.3255	24.4177	27.6627	56.2313	DEAD/DEAH box ATP-dependent RNA helicase, putative
PVX_096275	38.9821	0	0	0	0	0	0	54.1308	29.5372	13.6976	31.0367	0	hypothetical protein, conserved
PVX_096280	100.132	0	498.678	320.487	190.801	0	613.442	278.254	493.368	246.215	378.714	487.061	hypothetical protein, conserved
PVX_096285	22.761	0	28.2681	36.3343	7.21279	0	0	31.5975	8.62149	15.9978	33.9772	0	hypothetical protein, conserved
PVX_096289	0	0	0	0	61.5587	0	0	0	0	0	269.51	314.391	hypothetical protein
PVX_096292	0	0	0	660.812	65.5434	0	0	286.309	312.303	72.2701	40.9817	0	HAM1 domain containing protein
PVX_096295	0	41.795	0	0	0	0	0	41.575	17.0157	15.7862	11.9213	0	hypothetical protein, conserved
PVX_096300	0	0	445.117	572.129	283.765	0	273.857	0	67.6374	0	177.541	289.831	hypothetical protein, conserved
PVX_096302	0	311.161	0	0	142.029	0	0	0	336.945	777.527	264.943	0	conserved Plasmodium protein, unknown function
PVX_096305	153.324	0	95.2825	0	24.3096	0	117.183	0	101.653	26.9376	106.814	124.084	ferrodoxin reductase, putative
PVX_096307	0	0	0	0	0	0	0	0	0	0	0	0	conserved Plasmodium protein, unknown function
PVX_096310	0	0	0	0	0	0	0	0	0	0	62.3593	0	hypothetical protein, conserved
PVX_096315	0	0	0	0	0	0	187.96	0	92.9749	129.22	73.2374	0	hypothetical protein, conserved
PVX_096320	294.02	0	366.555	0	46.7416	0	0	0	111.474	51.6285	117.063	716.032	exosome complex component CSL4, putative (CSL4)
PVX_096325	33.6916	7.83643	0	0	3.55886	0	0	31.1811	8.50788	19.7338	8.9412	0	hypothetical protein, conserved
PVX_096330	139.316	48.6423	173.307	0	0	0	0	0	26.3958	24.4727	55.4624	0	hypothetical protein, conserved
PVX_096335	980.289	1256.5	1222.69	523.861	207.871	0	0	227.15	743.411	573.733	585.46	265.38	40S ribosomal protein S10, putative
PVX_096340	1231.9	860.607	766.949	985.796	391.256	526.128	1415.19	1854.34	1439.66	1045.76	1287.43	1664.63	60S ribosomal protein L11, putative
PVX_096345	0	40.0856	0	0	36.4288	293.846	0	79.7389	21.7541	40.3442	57.1405	0	hypothetical protein, conserved
PVX_096350	48.378	0	60.1048	0	15.3354	0	36.9581	0	36.6528	59.5014	48.146	0	hypothetical protein, conserved
PVX_096355	75.6893	0	0	0	24.0009	0	0	0	43.0126	119.682	45.1966	0	actin-related protein, putative (ARP6)
PVX_096360	0	0	0	0	0	0	0	0	0	0	48.8249	0	serine/threonine-protein kinase NEK4, putative
PVX_096365	0	0	0	0	86.2896	0	0	753.144	0	0	161.617	0	hypothetical protein
PVX_096370	0	83.9658	0	384.823	152.724	0	368.321	500.982	0	253.238	239.207	194.945	hypothetical protein, conserved
PVX_096380	0	0	0	1055.47	0	0	0	228.823	62.4071	0	131.06	267.341	conserved Plasmodium protein, unknown function
PVX_096385	0	0	0	0	0	0	0	0	0	0	28.7927	0	hypothetical protein, conserved
PVX_096390	0	0	0	0	0	0	0	0	0	0	0	0	hypothetical protein, conserved
PVX_096395	0	0	0	0	0	0	0	0	0	0	13.2353	0	hypothetical protein, conserved
PVX_096400	0	0	154.832	0	0	0	0	0	0	87.4769	37.1699	0	prefoldin subunit 3, putative
PVX_096405	0	0	0	0	74.1097	0	0	0	176.476	0	92.6181	0	30S ribosomal protein S8, putative
PVX_096410	0	0	0	0	0	0	0	0	0	0	0	0	cysteine repeat modular protein 2, putative (CRMP2)
PVX_096910	0	0	0	0	0	0	0	0	0	0	0	0	variable surface protein Vir23-related,PIR protein
PVX_096920	0	0	0	0	0	0	0	0	24.14	0	0	0	variable surface protein Vir12-related,PIR protein
PVX_096925	0	0	0	0	0	0	0	0	0	0	0	0	variable surface protein Vir15-like,PIR protein
PVX_096930	0	95.9682	0	0	0	0	0	0	0	0	0	0	variable surface protein Vir1-related,PIR protein
PVX_096935	0	0	0	0	0	0	0	0	0	0	0	0	variable surface protein Vir9-related,PIR protein
PVX_096937	0	0	0	0	0	0	0	0	0	0	28.6077	0	VIR protein, pseudogene,PIR protein, pseudogene
PVX_096938	0	0	0	0	0	0	0	0	0	0	0	0	VIR protein, pseudogene,PIR protein, pseudogene
PVX_096940	0	0	0	0	0	0	0	0	0	0	0	0	variable surface protein Vir24-related,PIR protein

Gene ID / Patient	Patient 1	Patient 2	Patient 3	Patient 4	Patient 5	Patient 6	Patient 7	Patient 8	Patient 9	Patient 10	Patient 11	Patient 12	Gene Description
PVX_096945	0	0	0	0	0	437.524	0	0	0	0	0	0	variable surface protein Vir4-related,PIR protein
PVX_096950	0	399.407	0	0	68.0682	366.067	109.4	99.309	135.46	226.059	56.9247	115.827	tryptophan-rich antigen (Pv-fam-a)
PVX_096955	160.502	373.518	0	342.025	84.8571	547.575	0	74.3017	405.42	319.561	181.037	173.264	Plasmodium exported protein, unknown function
PVX_096960	0	0	0	0	0	0	0	0	0	0	0	0	PST-A protein
PVX_096965	0	0	0	0	0	0	0	0	0	0	0	0	VIR protein,PIR protein
PVX_096970	0	196.405	0	0	22.3144	0	0	0	0	49.4067	139.963	0	variable surface protein Vir8-related,PIR protein
PVX_096975	182.919	298.01	303.324	584.816	58.036	0	186.538	169.371	231.034	107.112	254.871	98.7527	VIR protein,PIR protein
PVX_096980	181.205	442.996	0	289.91	86.3013	0	0	0	34.3375	190.967	144.282	0	variable surface protein Vir, putative,PIR protein
PVX_096985	0	0	0	298.079	0	0	0	0	35.3031	0	18.5421	0	variable surface protein Vir, putative,PIR protein
PVX_096987	152.004	425.03	758.121	0	145.006	0	0	0	345.793	53.381	90.7803	246.82	VIR protein,PIR protein
PVX_096990	0	0	0	0	0	0	0	0	0	35.9414	10.1805	0	Pv-fam-d protein
PVX_096992	0	0	0	375.711	0	0	179.798	163.049	0	82.4219	70.0683	0	Plasmodium exported protein, unknown function
PVX_096995	216.559	403.276	0	0	91.6376	369.618	110.461	200.542	109.418	101.443	172.427	116.951	tryptophan-rich antigen (Pv-fam-a)
PVX_097000	0	26.551	0	0	0	0	58.1429	0	0	26.7316	7.57119	0	Plasmodium exported protein, unknown function
PVX_097002	0	0	0	0	0	0	0	0	0	0	0	0	Plasmodium exported protein, unknown function
PVX_097005	0	0	0	0	0	0	0	0	0	111.295	63.0926	0	Plasmodium exported protein, unknown function
PVX_097010	0	0	0	0	0	0	0	0	99.3601	91.9106	52.1377	0	hypothetical protein
PVX_097015	0	21.2185	0	0	0	0	46.4586	0	46.0678	21.3657	18.1533	0	Plasmodium exported protein, unknown function
PVX_097025	35.9573	12.5462	0	0	11.3965	0	0	0	6.81033	25.2721	10.7354	0	ABC transporter, putative
PVX_097525	0	0	0	0	0	0	0	0	0	0	0	0	variable surface protein Vir 12, putative,PIR protein
PVX_097530	0	0	0	0	0	0	0	0	0	0	0	0	variable surface protein Vir22/12-related,PIR protein
PVX_097540	0	0	0	154.71	0	0	0	0	18.3406	17.0086	9.63535	0	variable surface protein Vir24-related,PIR protein
PVX_097542	0	0	0	0	0	0	0	0	0	0	0	0	VIR protein,PIR protein
PVX_097545	0	46.6786	0	0	0	0	0	0	25.3306	0	0	0	variable surface protein Vir12-related,PIR protein
PVX_097550	0	0	0	0	0	0	0	0	42.5791	19.7416	11.1841	0	VIR protein, pseudogene,PIR protein, pseudogene
PVX_097555	0	0	0	0	0	0	0	0	0	0	0	0	variable surface protein Vir12/16-related,PIR protein
PVX_097557	97.6628	327.14	194.113	124.751	37.1457	99.8557	0	27.1178	73.9903	109.825	62.2041	0	Plasmodium exported protein, unknown function
PVX_097560	0	33.3674	237.863	0	0	0	0	0	0	33.5598	19.0172	0	lysophospholipase, putative
PVX_097565	80.7879	338.535	0	0	25.6447	0	0	0	153.079	141.907	112.569	130.918	Plasmodium exported protein, unknown function
PVX_097567	0	101.096	0	0	0	0	0	201.048	54.8352	152.384	115.171	0	Plasmodium exported protein, unknown function
PVX_097570	0	0	0	0	0	0	0	0	0	50.6153	9.5578	0	Phist protein (Pf-fam-b)
PVX_097575	20.881	116.561	0	33.3323	0	53.3601	0	0	15.8187	55.0368	8.3122	16.8856	tryptophan-rich antigen (Pv-fam-a)
PVX_097577	0	0	0	0	38.1809	0	0	0	0	42.2063	95.6827	0	tryptophan-rich antigen (Pv-fam-a)
PVX_097580	102.424	71.5529	0	0	0	0	0	0	0	0	0	0	hypothetical protein
PVX_097583	1762.52	5422.85	0	2261.34	224.318	2715.89	541.206	1960.6	1938.28	2413.67	1017.57	0	skeleton-binding protein 1, putative (SBP1)
PVX_097585	0	0	0	0	0	0	0	201.048	0	50.7948	0	0	Plasmodium exported protein, unknown function
PVX_097590	0	0	0	0	0	0	0	0	0	0	0	0	rhoptry-associated protein 2, putative
PVX_097592	0	0	0	0	0	0	0	0	0	0	0	0	hypothetical protein
PVX_097595	0	0	0	0	0	0	0	47.2175	0	0	0	0	hypothetical protein, conserved
PVX_097600	0	0	0	446.564	0	0	106.834	96.983	105.831	73.5898	180.675	0	hypothetical protein, conserved
PVX_097605	0	38.7257	68.9436	88.6165	0	0	0	0	0	19.4981	5.5221	0	vacuolar protein sorting-associated protein 11, putative (VPS11)
PVX_097610	93.919	32.7824	0	150.077	0	0	0	0	106.751	66.0001	130.86	0	hypothetical protein, conserved
PVX_097615	0	56.1183	0	0	0	0	0	0	0	28.2286	15.9947	0	hypothetical protein, conserved
PVX_097620	0	0	0	0	0	0	0	0	0	0	0	0	hypothetical protein, conserved
PVX_097625	438.49	459.21	545.249	175.208	69.551	280.506	83.827	304.492	124.607	365.904	185.472	88.7577	merozoite surface protein 8, putative
PVX_097630	0	0	0	0	0	0	0	0	0	0	0	0	hypothetical protein, conserved
PVX_097635	0	0	0	0	0	0	0	0	0	0	0	0	hypothetical protein, conserved
PVX_097640	0	0	0	0	32.6422	0	0	142.805	77.909	36.1022	40.9177	0	hypothetical protein, conserved
PVX_097645	236.206	0	294.239	0	0	0	180.989	164.126	134.305	0	141.061	0	hypothetical protein, conserved
PVX_097650	0	0	0	0	0	0	0	0	0	0	0	0	hypothetical protein, conserved
PVX_097655	0	0	0	0	49.6038	0	0	0	0	0	62.1027	0	50S ribosomal protein L28, apicoplast, putative
PVX_097660	109.355	0	0	0	34.6904	0	83.6218	75.9369	20.717	38.4221	87.0676	88.5405	4-diphosphocytidyl-2-C-methyl-D-erythritol kinase, putative (IspE)
PVX_097665	37.2772	0	0	0	0	0	0	51.7624	0	13.0986	14.8396	0	hypothetical protein, conserved
PVX_097670	0	26.7558	0	0	0	0	0	0	0	0	0	0	merozoite surface protein 3 (MSP3.1)
PVX_097675	35.7151	24.9291	0	0	0	0	0	0	13.5306	0	0	0	merozoite surface protein 3 (MSP3.2)
PVX_097680	0	0	0	0	0	0	0	0	0	0	0	0	merozoite surface protein 3 (MSP3.3)
PVX_097685	0	0	0	0	0	0	0	0	16.233	0	0	0	merozoite surface protein 3 (MSP3.4)
PVX_097690	0	0	0	0	17.8563	0	0	0	5.33435	0	0	0	merozoite surface protein 3 (MSP3.5)
PVX_097695	0	0	0	0	0	0	0	0	0	0	3.56725	0	merozoite surface protein 3 (MSP3.6)
PVX_097700	0	0	0	0	0	0	0	0	0	0	0	0	merozoite surface protein 3 (MSP3.7)
PVX_097705	0	0	0	0	0	0	0	0	0	0	3.53329	0	merozoite surface protein 3 (MSP3.8)
PVX_097710	0	0	0	0	0	0	38.7505	0	0	0	0	0	merozoite surface protein 3 (MSP3.9)

Gene ID / Patient	Patient 1	Patient 2	Patient 3	Patient 4	Patient 5	Patient 6	Patient 7	Patient 8	Patient 9	Patient 10	Patient 11	Patient 12	Gene Description
PVX_097715	0	217.438	0	0	0	398.619	0	0	0	0	15.4938	0	hypothetical protein
PVX_097720	0	0	0	0	9.10592	0	0	0	0	0	0	0	merozoite surface protein 3 (MSP3.10)
PVX_097725	0	0	0	0	0	0	0	0	0	0	18.8362	0	merozoite surface protein 3 (MSP3.11)
PVX_097730	0	54.5017	0	0	0	399.664	0	0	0	54.8331	31.0687	0	hypothetical protein
PVX_097735	128.443	179.52	0	0	0	0	0	178.513	97.3823	90.2242	102.275	0	hypothetical protein
PVX_097740	97.2355	203.644	362.665	310.767	77.1033	497.527	74.3402	202.552	368.406	273.32	203.222	157.429	serine/arginine-rich splicing factor 12, putative (SRSF12)
PVX_097745	0	0	0	0	172.94	0	0	0	0	188.819	107.297	0	actin-depolymerizing factor 1, putative (ADF1)
PVX_097750	0	28.7121	0	0	0	0	0	0	0	0	0	0	protein kinase, putative
PVX_097755	0	0	0	0	0	0	0	0	0	0	8.09782	0	myosin B, putative
PVX_097760	373.078	782.741	1396.91	1795.51	118.735	0	859.472	778.213	707.43	851.47	557.052	0	60S ribosomal protein L31, putative (RPL31)
PVX_097765	0	42.5728	0	0	19.3453	0	0	169.371	69.3102	64.2671	60.6835	0	hypothetical protein, conserved
PVX_097770	32.2979	11.2691	80.2356	0	15.3542	0	74.0023	44.8389	18.3515	56.7508	19.2856	0	P-type ATPase, putative
PVX_097772	0	0	0	0	0	0	0	0	0	133.722	0	0	AN1-like zinc finger family protein
PVX_097775	41.5909	0	0	0	13.1896	0	0	0	15.7572	29.2281	24.8354	0	ATP-dependent helicase, putative
PVX_097780	0	0	0	0	0	0	0	0	0	0	0	0	hypothetical protein, conserved
PVX_097785	0	31.8786	0	0	0	0	0	63.4156	34.6029	0	9.08958	0	ATP-dependent helicase, putative
PVX_097787	0	0	0	0	0	0	0	0	0	0	0	0	conserved Plasmodium protein, unknown function
PVX_097790	0	0	0	0	0	0	0	0	0	0	109.058	0	2-oxoisovalerate dehydrogenase subunit beta, mitochondrial, putative (BCKDHB)
PVX_097795	18.9735	0	47.1385	0	12.0274	0	0	0	28.7489	26.6704	45.3178	0	hypothetical protein
PVX_097800	6.51157	4.54336	0	0	4.12644	0	9.94335	18.0782	7.39918	6.86525	10.3683	0	hypothetical protein, conserved
PVX_097805	0	0	0	0	0	0	0	0	0	0	18.7372	0	hypothetical protein, conserved
PVX_097810	0	0	0	0	0	0	0	17.7187	9.66918	4.48539	10.1616	0	hypothetical protein, conserved
PVX_097815	24.5397	8.56176	0	0	7.77663	0	0	0	4.64765	17.2479	14.653	0	trafficking protein particle complex subunit 8, putative (TRS85)
PVX_097820	0	0	0	0	0	0	0	0	0	0	0	0	actin-related protein, putative
PVX_097825	0	0	0	0	0	0	0	86.4537	23.5857	87.4769	12.39	0	hypothetical protein, conserved
PVX_097830	82.2642	28.7121	0	262.863	52.1758	0	0	114.235	62.3334	28.9058	122.807	66.581	hypothetical protein, conserved
PVX_097835	24.4067	17.0333	0	77.9513	7.73677	0	0	67.7729	36.9827	0	63.1531	0	DNA mismatch repair protein MSH6, putative (MSH6)
PVX_097840	0	0	0	0	0	0	0	0	87.2751	26.9699	45.8434	0	hypothetical protein, conserved
PVX_097845	0	43.1912	153.867	0	19.6266	0	0	0	0	0	24.6257	0	hypothetical protein, conserved
PVX_097850	87.4166	0	0	0	0	0	0	0	33.1293	122.836	69.6038	0	small ubiquitin-related modifier, putative (SUMO)
PVX_097852	0	0	0	0	161.289	0	1560.34	0	0	0	200.303	0	conserved Plasmodium protein, unknown function
PVX_097855	0	0	0	0	0	874.145	0	0	0	59.772	67.7738	0	hypothetical protein, conserved
PVX_097860	96.1512	134.331	239.4	307.713	61.0659	0	147.245	400.773	145.767	202.656	229.678	0	60S ribosomal subunit protein L24, putative
PVX_097865	325.574	0	0	521.947	51.778	0	0	0	123.451	114.33	226.851	264.41	TATA-box binding protein, putative (TBP)
PVX_097870	0	0	0	0	35.7272	0	0	0	0	0	22.3874	0	hypothetical protein, conserved
PVX_097875	0	0	0	0	0	0	0	0	0	0	0	0	hypothetical protein, conserved
PVX_097885	0	11.8763	0	27.1688	0	0	0	11.8141	3.22354	8.97257	8.46943	0	hypothetical protein, conserved
PVX_097890	0	0	0	0	0	0	0	0	0	0	0	0	histone deacetylase, putative
PVX_097895	0	0	0	0	13.8873	0	0	0	16.5901	15.3861	0	0	TBC domain containing protein
PVX_097900	34.4768	24.0644	0	0	10.9316	0	0	95.7452	39.184	36.3443	0	55.7976	transcription factor 25, putative (TCF25)
PVX_097905	56.686	0	0	0	0	0	0	78.7282	42.9568	39.8332	45.1333	0	rhomboid protease ROM4, putative (ROM4)
PVX_097910	0	0	0	0	82.5222	0	0	0	0	0	0	0	hypothetical protein, conserved
PVX_097915	854.348	596.563	163.489	210.141	187.683	0	201.088	638.973	896.494	392.519	523.26	212.908	60S ribosomal protein L4, putative (RPL4)
PVX_097920	0	0	0	0	0	0	0	0	0	0	0	0	subtilisin-like serine protease, putative
PVX_097925	0	0	0	0	0	0	0	0	0	0	0	0	hypothetical protein, conserved
PVX_097930	0	0	0	0	0	0	0	0	0	0	51.582	0	hypothetical protein, conserved
PVX_097935	0	0	0	0	0	0	0	0	0	0	0	0	subtilisin-like protease 1 (SUB1)
PVX_097940	0	0	0	0	25.1172	0	0	0	15.0039	0	31.5312	0	cell differentiation protein rcd1, putative
PVX_097945	64.2563	44.8672	319.688	205.455	101.944	0	196.603	89.2488	194.785	270.907	217.435	104.08	hypothetical protein, conserved
PVX_097950	52.1865	54.6323	64.8399	0	16.5435	0	0	36.2286	29.6541	36.6771	31.1618	0	hypothetical protein, conserved
PVX_097955	183.104	128.049	0	0	116.539	0	0	254.619	138.877	128.585	145.811	0	hypothetical protein, conserved
PVX_097960	206.979	0	0	0	32.8692	0	0	143.797	0	0	103.004	167.814	6-cysteine protein
PVX_097965	0	0	0	0	0	0	0	0	0	0	0	0	hypothetical protein, conserved
PVX_097970	0	0	0	0	0	0	157.971	0	0	108.681	102.648	0	hypothetical protein, conserved
PVX_097975	0	0	0	0	0	0	0	448.525	40.7824	37.794	85.6731	174.492	triose/hexose phosphate phosphate translocator, putative
PVX_097980	0	0	0	0	0	0	0	0	0	0	0	0	transcription factor IIb, putative
PVX_097985	0	0	0	0	0	0	0	0	0	0	0	0	guanidine nucleotide exchange factor, putative
PVX_097990	0	0	365.952	0	46.6647	0	0	0	0	0	116.871	238.284	hypothetical protein, conserved
PVX_097995	0	14.9208	0	68.2802	0	109.309	0	0	0	15.0267	8.51115	0	ATP-dependent RNA helicase, putative
PVX_098000	0	0	0	0	44.4689	0	0	0	0	49.1286	83.5422	0	single-strand binding protein, putative
PVX_098005	0	0	0	0	0	0	0	0	0	0	0	0	hypothetical protein
PVX_098010	0	0	0	0	10.4677	0	0	15.2855	4.17072	7.73909	8.76628	0	hypothetical protein

Gene ID / Patient	Patient 1	Patient 2	Patient 3	Patient 4	Patient 5	Patient 6	Patient 7	Patient 8	Patient 9	Patient 10	Patient 11	Patient 12	Gene Description
PVX_098015	0	76.7417	0	0	0	0	0	0	166.536	38.5822	109.326	0	soluble NSF attachment protein (SNAP), putative
PVX_098020	0	29.9921	0	0	0	0	0	0	0	0	12.8311	0	chromosome condensation protein, putative
PVX_098022	0	0	0	326.719	0	0	0	0	0	0	0	0	conserved Plasmodium protein, unknown function
PVX_098023	0	0	0	0	125.136	0	0	0	0	0	467.429	0	conserved Plasmodium protein, unknown function
PVX_098025	22.7004	47.5195	28.1928	0	21.5807	0	34.6694	15.7567	38.6934	27.9216	40.664	55.0719	DNA-directed RNA polymerase I 190 kDa polypeptide, putative
PVX_098030	0	0	0	0	0	0	0	0	0	0	0	0	hypothetical protein
PVX_098035	80.5697	0	0	0	25.5753	0	0	0	0	0	16.0379	130.564	hypothetical protein, conserved
PVX_098040	129.279	0	0	137.704	13.6664	0	263.523	179.524	48.9793	151.417	94.3521	0	asparagine--tRNA ligase, putative
PVX_098045	0	0	0	0	0	0	0	0	65.8083	0	0	0	hypothetical protein, conserved
PVX_098050	0	28.552	0	0	19.4521	0	0	56.8025	61.9934	43.1328	48.8606	33.0944	phosphatidylinositol 4-kinase, putative (PI4K)
PVX_098055	88.4534	0	0	0	28.0832	0	0	0	67.0449	62.1463	70.4294	143.371	hypothetical protein, conserved
PVX_098060	0	0	0	0	0	0	0	0	0	0	24.5728	0	hypothetical protein, conserved
PVX_098065	0	0	30.4029	39.0783	0	0	18.6937	0	0	4.30137	14.6169	19.7964	hypothetical protein, conserved
PVX_098070	0	31.0695	110.655	0	14.1155	0	0	123.613	16.8625	46.9158	79.7312	0	cyclophilin, putative
PVX_098075	0	0	0	0	0	0	0	129.551	0	0	18.5614	0	stripes inner membrane complex protein, putative (SIP)
PVX_098080	0	0	0	0	20.7925	0	0	0	49.6593	92.087	65.2159	0	hypothetical protein, conserved
PVX_098085	0	0	0	0	0	0	0	0	39.0936	48.3474	27.3863	0	topoisomerase I, putative
PVX_098582	0	0	0	0	0	0	0	0	0	0	0	0	reticulocyte binding protein 1b (RBP1b)
PVX_098585	0	0	0	0	0	0	0	0	0	0	2.14615	0	reticulocyte binding protein 1a (RBP1a)
PVX_098590	0	0	0	0	0	0	0	0	0	0	0	0	hypothetical protein, conserved
PVX_098595	0	0	282.278	0	0	0	173.629	0	171.812	0	135.345	183.802	hypothetical protein, conserved
PVX_098600	0	0	0	0	141.559	0	0	0	84.2884	0	44.2388	0	RER1 protein, putative
PVX_098605	0	129.726	0	0	0	0	0	129.013	70.3862	97.8601	147.875	0	ras-related protein RAB7, putative (RAB7)
PVX_098610	0	0	0	0	0	0	0	12.8518	0	0	1.84266	0	hypothetical protein, conserved
PVX_098615	50.1521	17.4978	0	20.0156	11.92	0	38.2992	17.4059	9.49848	41.859	43.672	20.2792	DEAD/DEAH box helicase, putative
PVX_098620	0	58.1003	0	66.4688	13.1944	0	31.7974	0	23.6531	7.31418	33.1419	0	hypothetical protein, conserved
PVX_098625	0	0	0	0	0	0	0	0	0	0	0	0	hypothetical protein, conserved
PVX_098630	0	0	0	0	14.1014	0	135.957	123.49	16.8457	31.2461	115.053	0	tubulin alpha chain, putative
PVX_098635	0	0	0	0	0	0	0	0	0	0	0	0	LCCL domain-containing protein
PVX_098640	452.46	263.315	187.649	723.582	167.541	386.163	346.218	419.009	428.65	423.885	390.278	366.555	60S ribosomal protein L32, putative (RPL32)
PVX_098645	0	221.487	0	253.615	0	0	242.702	0	210.321	83.5608	157.821	0	GTPase-activating protein, putative
PVX_098650	40.1608	0	0	0	12.7294	0	0	0	22.8197	28.2262	11.9904	32.4852	AP-4 complex subunit epsilon, putative
PVX_098655	0	0	0	0	0	0	0	91.686	0	23.1915	0	0	hypothetical protein, conserved
PVX_098660	0	0	0	41.7333	0	0	0	0	0	0	0	0	hypothetical protein, conserved
PVX_098665	252.266	0	0	0	0	0	0	0	0	88.5468	50.2264	0	signal peptidase complex subunit 3, putative
PVX_098670	0	0	291.919	0	0	0	179.562	0	0	0	139.953	0	prefoldin subunit, putative
PVX_098675	35.8278	0	0	0	5.67769	0	27.3649	74.6106	13.5716	12.5905	21.3934	0	ubiquitin carboxyl-terminal hydrolase, putative
PVX_098680	0	0	0	0	0	0	0	0	0	0	97.9667	0	bacterial histone-like protein, putative (HU)
PVX_098685	58.3111	40.7129	145.03	0	36.9992	0	178.379	80.9865	66.2833	81.9498	116.068	0	replication protein A1, small fragment, putative
PVX_098690	14.8691	0	36.937	0	18.8492	0	0	41.2848	22.5292	41.8028	59.1904	0	copper-transporting ATPase, putative (CuTP)
PVX_098695	0	96.8615	0	444.05	0	0	0	0	52.5403	48.6724	193.12	0	hypothetical protein, conserved
PVX_098700	29.5614	41.2564	0	0	14.0528	0	0	20.5197	16.7964	0	17.6515	0	nucleoporin NUP100/NSP100, putative (NUP100)
PVX_098705	0	19.7862	70.4518	0	0	0	0	0	0	9.96212	33.8568	0	mitochondrial carrier protein, putative
PVX_098710	0	0	0	0	0	0	0	0	0	0	0	0	dynein heavy chain, putative
PVX_098712	72.4715	0	0	0	0	0	0	0	0	0	0	0	high molecular weight rhoptry protein 3, putative (RhopH3)
PVX_098715	0	0	0	0	0	0	0	0	0	0	0	0	hypothetical protein, conserved
PVX_098720	0	0	0	0	0	0	0	0	0	0	0	0	hypothetical protein, conserved
PVX_098725	0	38.291	0	175.316	17.3984	0	0	76.1697	0	19.2699	21.8336	0	autophagy-related protein 3, putative (ATG3)
PVX_098730	0	31.1518	0	0	0	0	0	30.9871	25.364	23.5293	13.3271	0	hypothetical protein, conserved
PVX_098735	0	88.0144	0	201.416	9.99515	0	0	0	0	77.5454	50.1995	0	coatomer complex beta subunit, putative (SEC27)
PVX_098740	0	0	0	0	0	0	0	0	4.82221	4.4739	7.60165	0	endonuclease/exonuclease/phosphatase domain containing protein
PVX_098745	0	11.263	0	0	5.1153	0	0	22.4073	18.3415	5.67201	25.7002	0	exoribonuclease II, putative (RNaseII)
PVX_098750	0	0	0	0	0	0	0	0	0	0	46.5292	0	vacuolar protein sorting-associated protein 46, putative (VPS46)
PVX_098755	0	0	0	0	0	0	0	0	0	0	24.4735	99.5681	hypothetical protein, conserved
PVX_098760	69.9847	0	0	0	44.419	0	0	0	106.079	49.175	153.237	0	Maf-like protein, putative
PVX_098765	0	0	0	0	0	0	0	0	0	0	0	0	dynein light intermediate chain 2, putative
PVX_098770	0	0	0	0	0	0	0	0	0	0	0	0	arginase, putative
PVX_098775	38.7794	81.207	0	0	36.8915	0	0	53.8492	88.1506	81.7582	69.4695	0	zinc finger protein, putative
PVX_098780	64.2563	44.8672	0	0	40.7775	0	0	89.2488	73.0442	90.3024	63.9515	0	leucine-rich repeat protein
PVX_098784	0	0	0	2171.28	215	0	0	0	0	0	399.006	0	hypothetical protein
PVX_098786	0	104.131	0	0	0	0	0	414.161	0	104.631	29.6555	0	tubulin-specific chaperone, putative
PVX_098795	0	0	0	0	125.136	0	0	0	148.572	137.222	0	639.553	hypothetical protein

Gene ID / Patient	Patient 1	Patient 2	Patient 3	Patient 4	Patient 5	Patient 6	Patient 7	Patient 8	Patient 9	Patient 10	Patient 11	Patient 12	Gene Description
PVX_098800	23.0668	0	0	0	7.31178	0	70.4843	32.0256	8.73801	0	36.7298	0	hypothetical protein, conserved
PVX_098805	0	0	0	0	0	0	0	35.9144	9.79896	9.08977	20.5944	0	TBC domain containing protein
PVX_098810	243.892	0	0	0	0	0	0	169.371	23.1034	0	121.367	0	hypothetical protein, conserved
PVX_098815	118.182	82.4945	97.9278	0	24.9844	201.512	0	109.406	14.9246	55.369	70.5705	0	ATP-dependent heat shock protein, putative
PVX_098820	0	0	0	0	0	0	0	0	0	0	58.6626	0	hypothetical protein
PVX_098825	262.911	0	0	120.001	11.9097	0	57.41	52.1536	56.9168	105.58	82.2345	0	hypothetical protein, conserved
PVX_098830	129.03	60.0475	106.928	0	95.4808	0	131.508	119.453	228.13	196.464	196.902	278.498	proteasome activator complex subunit 3, putative,subunit of proteaseome activator complex, putative
PVX_098835	0	0	0	0	199.966	0	0	436.056	0	109.894	62.3593	0	ribosomal protein L35, putative
PVX_098840	0	0	0	0	0	0	0	0	64.9171	60.1148	102.244	0	peptide deformylase, putative (PDF)
PVX_098845	0	0	0	0	0	0	0	0	30.9512	28.6921	81.2872	0	S1/P1nuclease, putative
PVX_098847	0	0	0	0	21.0237	0	0	0	25.1055	0	26.3761	0	conserved Plasmodium membrane protein, unknown function
PVX_098850	0	0	0	0	5.86441	0	0	0	14.0178	0	11.0483	0	hypothetical protein, conserved
PVX_098855	0	51.5329	0	0	70.2611	0	0	102.505	139.818	25.9251	102.822	119.56	hypothetical protein, conserved
PVX_098860	92.9553	0	0	0	0	0	0	0	35.2297	0	0	0	hypothetical protein, conserved
PVX_098865	0	0	0	0	0	0	0	0	0	0	3.07128	0	hypothetical protein, conserved
PVX_098870	0	0	219.886	0	0	0	67.6084	61.4093	0	0	17.6042	0	ribosomal RNA methyltransferase, putative
PVX_098875	0	0	310.694	0	0	0	0	0	47.2653	0	49.6413	0	tRNA pseudouridine synthase, putative
PVX_098880	0	46.4701	0	212.802	0	0	0	92.4365	25.2176	46.7623	39.7407	0	transporter, putative
PVX_098885	34.3445	0	0	0	10.8896	0	0	0	13.0112	0	34.1803	0	hypothetical protein, conserved
PVX_098890	0	0	0	130.174	12.9191	0	0	113.142	15.4343	42.9443	0	65.9438	hypothetical protein, conserved
PVX_098895	0	0	0	0	0	0	0	0	0	0	10.694	0	hypothetical protein, conserved
PVX_098900	0	0	0	0	0	0	0	0	0	0	0	0	GDP-fucose protein O-fucosyltransferase 2, putative (POFUT2)
PVX_098905	464.051	325.7	0	0	0	0	0	0	0	162.712	184.837	0	apoptosis-related protein, putative (ARP)
PVX_098910	46.3846	48.5567	0	0	7.35159	0	0	0	17.5712	24.45	27.6973	0	exoribonuclease, putative
PVX_098915	0	0	0	143.444	0	0	0	0	0	0	0	0	subpellicular microtubule protein 1, putative (SPM1)
PVX_098920	0	41.3328	0	0	0	0	0	82.2194	22.4307	20.7991	23.5669	0	hypothetical protein, conserved
PVX_098925	0	0	0	0	0	0	0	42.3844	11.5641	10.7266	12.1518	0	hypothetical protein, conserved
PVX_098930	0	0	0	0	0	0	0	0	0	211.145	0	0	small nuclear ribonucleoprotein (snRNP), putative
PVX_098935	22.4928	0	0	0	7.12974	0	0	0	25.5617	31.6168	17.9079	36.3903	DEAD/DEAH box helicase, putative
PVX_098940	0	0	0	0	9.40809	0	0	0	0	0	0	0	SET domain protein, putative (SET4)
PVX_098945	26.4401	129.171	131.405	0	16.7635	0	40.4004	36.7104	30.0484	37.1646	99.991	42.7813	Ran-binding protein, putative
PVX_098950	19.8197	13.8295	24.6141	31.6377	3.14024	202.589	0	27.5139	30.0292	10.4479	13.807	0	hypothetical protein, conserved
PVX_098955	0	0	0	0	23.537	0	113.489	0	28.1028	26.0539	44.2857	0	hypothetical protein, conserved
PVX_098960	0	0	0	0	17.1281	0	0	0	10.2339	113.917	43.0166	0	selenophosphate synthetase, putative
PVX_098965	0	0	0	0	0	0	0	26.681	21.8395	6.75351	19.1257	0	DNA repair protein REV1, putative
PVX_098970	0	0	0	0	71.8558	0	0	313.791	171.13	79.1841	224.54	0	prenylated protein, putative
PVX_098975	0	0	0	0	0	0	0	77.8402	0	0	0	0	actin-like protein, putative
PVX_098980	0	54.9333	0	0	24.9672	0	0	0	0	55.2667	0	0	cytosolic Fe-S cluster assembly factor NBP35, putative (NBP35)
PVX_098985	0	86.7425	154.509	0	0	0	95.0202	0	0	43.6474	86.5491	0	DNA primase large subunit, putative
PVX_098990	0	0	0	0	0	0	0	0	0	0	0	0	phosphatidylinositol N-acetylglucosaminyltransferase, putative
PVX_098995	0	0	0	0	0	0	0	0	0	0	0	0	hypothetical protein, conserved
PVX_099000	0	0	0	0	0	0	0	0	0	0	28.4705	0	hypothetical protein, conserved
PVX_099005	0	0	0	0	0	0	0	0	0	0	1.85127	0	cysteine repeat modular protein 1, putative (CRMP1)
PVX_099010	0	31.0048	0	0	0	0	0	30.8409	16.8296	31.2245	17.6857	0	hypothetical protein, conserved
PVX_099015	0	0	0	0	0	0	0	0	0	0	39.5267	0	hypothetical protein, conserved
PVX_099020	0	0	173.307	0	22.1056	0	106.584	0	26.3958	73.4181	97.0592	0	hypothetical protein, conserved
PVX_099025	0	0	0	113.021	11.2171	0	54.0702	98.244	53.6087	62.154	105.622	0	GTP-binding protein, putative
PVX_099030	0	0	0	0	0	0	0	0	0	33.9217	19.2225	0	hypothetical protein, conserved
PVX_099035	434.519	354.01	360.386	0	114.918	0	110.82	100.596	82.3289	279.87	201.816	0	inhibitor of cysteine proteases, putative (ICP)
PVX_099040	0	14.9529	0	136.855	0	0	0	29.7479	0	0	21.3237	0	hypothetical protein, conserved
PVX_099045	0	0	0	0	19.5051	0	0	0	0	21.5989	36.7102	99.5681	hypothetical protein, conserved
PVX_099050	0	0	0	0	0	0	0	0	0	0	0	0	hypothetical protein, conserved
PVX_099055	0	0	0	0	25.0333	0	0	0	29.8869	83.1191	78.4932	0	hypothetical protein, conserved
PVX_099060	0	41.5528	0	0	18.8815	0	0	0	45.1002	41.8193	47.3845	0	hypothetical protein, conserved
PVX_099065	0	0	0	0	0	0	0	0	0	0	5.44113	0	hypothetical protein, conserved
PVX_099070	0	0	3063.01	0	0	0	0	0	0	0	238.079	0	hypothetical protein, conserved
PVX_099075	0	33.0426	117.687	0	0	0	72.3713	0	35.8661	0	18.8426	0	tRNA (adenine(58)-N(1))-methyltransferase non-catalytic subunit TRM6, putative (GCD10)
PVX_099080	140.409	49.0242	0	0	111.397	0	0	0	266.03	172.652	307.436	227.467	26S proteasome regulatory subunit RPN8, putative (RPN8)
PVX_099085	169.895	118.791	0	0	108.096	0	521.579	236.22	64.4235	119.317	67.6451	0	hypothetical protein, conserved
PVX_099090	0	0	0	0	0	0	0	0	0	0	0	0	hypothetical protein, conserved
PVX_099095	429.944	180.176	0	275.111	136.495	440.474	263.28	597.298	456.23	332.285	427.917	278.734	elongation factor 1B, putative
PVX_099100	106.302	0	0	0	0	0	0	0	20.1385	18.6749	42.3185	0	hypothetical protein, conserved

Gene ID / Patient	Patient 1	Patient 2	Patient 3	Patient 4	Patient 5	Patient 6	Patient 7	Patient 8	Patient 9	Patient 10	Patient 11	Patient 12	Gene Description
PVX_099105	0	0	0	0	0	0	0	0	0	0	74.5659	0	hypothetical protein, conserved
PVX_099107	0	0	0	0	24.836	0	0	0	59.3032	0	31.1502	0	protease, putative
PVX_099110	91.2963	0	0	0	9.64821	0	0	0	11.5287	21.3875	24.2291	0	hypothetical protein, conserved
PVX_099115	43.7245	30.5228	0	0	27.7339	0	0	0	16.5658	46.0908	95.7351	0	hypothetical protein, conserved
PVX_099117	0	0	0	0	0	0	0	0	0	0	0	0	conserved Plasmodium protein, unknown function
PVX_099120	0	45.7484	0	0	10.3908	0	0	0	12.4155	34.5479	26.0925	0	arginine--tRNA ligase, putative
PVX_099125	80.8156	0	0	0	12.814	0	123.541	56.1113	61.2354	28.3969	16.086	0	pseudouridylate synthase, putative
PVX_099130	0	0	0	0	0	0	0	0	11.4585	10.6287	12.0409	0	hypothetical protein, conserved
PVX_099135	84.6868	0	210.788	0	26.8849	0	0	0	32.0941	59.5011	16.8575	0	phospholipid or glycerol acyltransferase, putative
PVX_099140	0	31.5404	0	0	14.3295	0	69.0783	0	0	15.8754	17.9864	0	Met-10+ domain containing protein
PVX_099145	0	0	0	0	0	0	0	227.566	62.0643	57.4782	0	0	hypothetical protein, conserved
PVX_099150	0	0	0	0	5.64041	0	0	0	0	0	21.253	0	hypothetical protein, conserved
PVX_099155	0	0	0	0	0	0	0	0	0	0	327.997	0	zinc binding protein, putative
PVX_099160	0	0	0	0	0	0	0	0	0	0	28.4623	115.827	transporter, putative
PVX_099165	0	0	0	0	0	0	0	0	0	0	0	0	GINS complex subunit Psf3, putative
PVX_099170	0	0	0	0	0	0	0	0	0	0	0	0	hypothetical protein
PVX_099175	30.6825	42.8299	76.2538	0	0	0	0	42.6024	58.1179	10.7817	61.0713	49.6516	BSD-domain protein, putative
PVX_099180	0	0	0	0	0	0	0	0	0	44.9576	38.2063	0	type II NADH:ubiquinone oxidoreductase, putative
PVX_099185	0	0	810.305	0	0	0	0	0	122.748	0	257.552	0	SUMO-conjugating enzyme UBC9, putative (UBC9)
PVX_099190	0	0	0	0	32.8692	0	0	287.595	39.225	0	144.206	0	ribonuclease H2 subunit C, putative
PVX_099195	91.4712	0	0	0	0	0	0	63.5129	69.3119	48.2102	27.3105	0	hypothetical protein, conserved
PVX_099200	110.139	92.2358	0	0	20.9468	112.62	134.614	30.5828	83.4437	100.63	87.6883	35.6375	6-phosphofructokinase, putative
PVX_099205	0	0	0	0	15.8164	0	0	0	0	17.5202	0	0	hypothetical protein, conserved
PVX_099210	0	0	379.06	0	0	0	233.193	0	0	0	121.04	246.82	hypothetical protein, conserved
PVX_099215	0	0	0	0	31.4125	0	0	0	0	34.7462	78.7597	0	hypothetical protein, conserved
PVX_099220	0	0	0	0	0	0	0	0	0	0	0	0	glycolipid transfer protein, putative
PVX_099225	0	0	0	0	0	0	0	0	0	73.3836	62.3796	0	hypothetical protein, conserved
PVX_099230	0	0	0	0	6.38763	0	0	0	0	0	0	0	sugar transporter, putative
PVX_099235	129.935	0	0	0	0	0	0	0	0	0	25.8659	0	thioredoxin 3, putative (TRX3)
PVX_099240	29.6458	0	0	94.6981	0	0	0	0	33.6922	10.4175	41.3054	0	hypothetical protein, conserved
PVX_099245	0	0	0	0	0	0	0	0	0	0	0	0	hypothetical protein, conserved
PVX_099247	31.7817	0	0	0	5.03625	0	24.273	44.1223	42.1357	11.1688	31.629	0	conserved Plasmodium protein, unknown function
PVX_099250	0	0	0	0	0	408.958	0	110.926	121.042	84.157	31.7895	0	ubiquitin fusion degradation protein 1, putative (UFD1)
PVX_099255	34.9071	24.3649	0	0	0	0	53.3524	0	26.4488	36.7979	6.94805	0	methyltransferase, putative
PVX_099257	0	76.3662	0	0	0	0	0	0	0	0	0	0	RNA-binding protein musashi, putative (HoMu)
PVX_099263	0	0	0	0	0	0	0	0	3742.76	0	0	0	conserved Plasmodium protein, unknown function
PVX_099265	0	0	0	0	0	0	0	0	0	0	69.3571	0	hypothetical protein, conserved
PVX_099270	15.9503	0	0	0	5.05511	0	0	0	12.0838	0	28.5726	0	hypothetical protein, conserved
PVX_099275	0	34.6374	0	158.575	15.7374	0	227.603	0	112.79	34.8657	19.7515	0	N-glycosylase/DNA lyase, putative (OGG1)
PVX_099280	0	0	0	0	0	0	0	0	0	0	0	0	hypothetical protein, conserved
PVX_099285	0	0	0	0	0	0	0	0	0	0	0	0	hypothetical protein, conserved
PVX_099290	0	0	0	0	0	0	0	0	0	0	0	0	hypothetical protein, conserved
PVX_099295	0	35.2236	125.461	0	16.0039	0	77.1527	0	38.2327	17.7276	120.513	81.6922	hypothetical protein, conserved
PVX_099300	115.736	0	0	0	0	0	0	53.5705	58.463	54.2236	30.7156	62.4435	pre-mRNA-splicing factor ATP-dependent RNA helicase PRP43, putative
PVX_099305	0	0	0	0	0	0	0	0	38.9545	36.1022	0	0	XPA binding protein 1, putative
PVX_099310	0	0	0	785.968	0	0	125.359	0	124.145	0	81.5104	132.72	hypothetical protein, conserved
PVX_099315	409.527	540.006	565.668	581.664	187.611	698.415	347.853	884.657	672.354	687.501	697.406	368.327	78 kDa glucose-regulated protein precursor (GRP 78), putative,heat shock protein, putative,DnaK domain containing protein
PVX_099320	0	0	156.578	0	0	0	0	87.4272	0	0	12.5294	101.953	glideosome-associated protein 50, putative (GAP50)
PVX_099325	0	0	470.566	604.84	0	0	0	0	0	198.561	112.584	306.402	cytochrome b5-like Heme/Steroid binding domain containing protein
PVX_099330	107.855	0	0	0	0	0	0	0	122.642	0	85.8797	0	50S ribosomal protein L3, apicoplast, putative
PVX_099335	0	246.917	0	377.2	0	603.962	180.511	0	491.154	165.494	187.587	764.333	eukaryotic translation initiation factor 3 subunit 5, putative
PVX_099340	0	0	0	0	0	0	28.0043	0	6.94428	0	14.5953	0	hypothetical protein, conserved
PVX_099345	0	0	0	142.018	14.0944	0	0	0	50.5121	15.6153	61.9207	0	ATP-dependent DNA helicase Q1, putative (RECQ1)
PVX_099350	0	0	0	0	0	0	0	0	0	0	0	0	hypothetical protein
PVX_099355	0	148.607	0	0	0	272.32	81.3805	0	20.1625	18.6971	21.1844	0	tRNA-dihydrouridine synthase A, putative
PVX_099360	30.905	43.1406	153.615	0	19.5965	158.049	47.2295	85.823	46.8315	86.8791	79.9685	0	gamma-glutamylcysteine synthetase, putative
PVX_099365	697.022	139.12	495.896	318.699	221.359	510.275	762.523	276.704	603.841	594.626	554.999	322.896	nucleosome assembly protein 1, putative
PVX_099370	128.976	135.087	0	0	20.4623	0	0	89.5705	122.179	67.9707	102.691	0	DNAJ-like molecular chaperone protein, putative
PVX_099375	0	0	0	0	0	0	0	0	0	0	0	0	hypothetical protein, conserved
PVX_099380	0	0	0	0	0	0	0	0	0	0	0	0	thioredoxin-like protein 1, putative (TrxL1)
PVX_099385	0	0	0	0	20.9771	0	101.14	0	25.0498	46.4515	39.4764	107.084	protein disulfide isomerase, putative
PVX_099390	0	52.1788	185.922	238.974	47.4284	0	0	0	56.6282	26.2496	29.7457	242.121	sugar transporter, putative

Gene ID / Patient	Patient 1	Patient 2	Patient 3	Patient 4	Patient 5	Patient 6	Patient 7	Patient 8	Patient 9	Patient 10	Patient 11	Patient 12	Gene Description
PVX_099395	67.1491	0	0	214.722	63.9244	0	0	186.539	25.4447	70.7748	66.8309	0	dolichyl-diphosphooligosaccharide--protein glycosyltransferase, putative
PVX_099400	0	119.018	0	0	0	0	0	0	64.5462	119.544	33.8869	0	hypothetical protein, conserved
PVX_099405	33.063	0	0	0	0	0	50.5308	0	25.0512	11.6181	19.7429	0	TLD domain-containing protein
PVX_099410	0	0	0	0	0	0	0	0	0	4.18964	0	0	hypothetical protein, conserved
PVX_099415	0	42.924	0	196.548	0	0	0	0	0	0	12.2367	0	GNS1/SUR4 domain containing protein
PVX_099420	0	106.257	0	0	48.3353	0	0	0	172.897	53.381	151.3	0	co-chaperone Hsc20, putative
PVX_099425	33.2102	0	82.5416	0	21.0594	0	0	46.1133	25.1627	46.6791	66.1027	0	hypothetical protein, conserved
PVX_099430	0	0	0	0	0	0	0	0	0	0	0	0	hypothetical protein, conserved
PVX_099435	18.9735	13.2406	0	60.5893	0	0	28.9846	26.3416	14.3745	13.3352	26.4354	0	hypothetical protein, conserved
PVX_099440	0	0	0	0	0	0	0	0	0	0	143.401	0	ADP-ribosylation factor-like protein, putative
PVX_099445	0	17.7753	63.2887	0	0	0	0	0	19.2967	17.9002	25.3474	41.2096	hypothetical protein, conserved
PVX_099450	0	0	0	0	0	0	0	0	0	0	0	0	hypothetical protein, conserved
PVX_099455	146.49	204.534	0	0	61.9522	249.851	223.996	135.624	222.009	205.885	194.389	79.0587	inosine-5'-monophosphate dehydrogenase, putative
PVX_099460	146.141	0	0	0	0	0	0	101.5	110.758	77.0134	72.7246	0	RNA-binding protein, putative
PVX_099465	180.519	378.71	0	579.089	114.886	927.329	554.382	251.018	205.371	380.315	35.938	0	ubiquitin-conjugating enzyme E2, putative
PVX_099470	0	0	0	0	0	0	0	0	0	0	6.63984	0	WD domain, G-beta repeat domain containing protein
PVX_099475	0	0	0	0	0	0	0	0	66.0556	122.334	208.071	0	hypothetical protein, conserved
PVX_099480	0	39.1605	0	44.7964	4.44626	71.7129	0	38.9545	21.2576	29.5828	25.1324	22.6931	hypothetical protein, conserved
PVX_099485	0	0	0	0	0	0	0	0	75.3165	46.5546	0	0	hypothetical protein, conserved
PVX_099490	0	0	0	0	0	0	0	418.346	57.0502	105.686	29.9548	0	NifU-like scaffold protein, putative
PVX_099495	0	0	408.78	0	26.0691	0	125.705	0	31.1217	28.85	81.7348	0	hypothetical protein, conserved
PVX_099500	0	13.757	0	0	12.4965	0	0	27.3688	7.46749	6.92754	19.6186	0	hypothetical protein, conserved
PVX_099505	0	0	0	0	15.0522	0	145.127	0	35.9612	50.025	56.6777	0	hypothetical protein, conserved
PVX_099507	0	39.9318	0	182.835	0	0	0	0	21.6707	0	34.1528	0	hypothetical protein, conserved
PVX_099510	73.6719	102.896	183.315	0	23.3817	0	225.48	102.336	0	77.6472	102.653	0	50S ribosomal protein L29, putative
PVX_099515	0	0	0	0	0	0	0	0	0	0	7.61978	0	WD domain, G-beta repeat domain containing protein
PVX_099520	55.9829	91.1572	46.3615	0	17.7437	0	57.0136	155.445	148.445	104.924	85.4282	30.1877	ubiquitin-like protein, putative
PVX_099525	665.954	464.886	883.041	425.63	352.011	0	814.526	554.878	504.619	514.794	892.541	503.107	S-adenosylmethionine synthetase, putative
PVX_099528	0	0	0	0	0	0	0	0	0	0	0	0	conserved Plasmodium protein, unknown function
PVX_099530	31.4591	0	0	100.495	0	0	0	0	0	11.0545	6.2617	0	para-aminobenzoic acid synthetase, putative
PVX_099535	1887.5	912.528	1445.06	3018.28	437.754	371.722	2443.98	2319.34	2365.84	2652.48	1632.91	1058.55	phosphoglycerate kinase, putative
PVX_099540	125.275	43.7358	0	0	19.8742	320.632	0	0	71.203	66.0208	37.404	0	glutamine synthetase, putative
PVX_099545	80.9341	113.049	0	258.902	25.6912	0	0	224.863	0	28.4327	80.552	0	pre-mRNA splicing factor, putative
PVX_099550	7.9354	11.0738	0	25.3327	10.0578	0	0	22.0316	15.0286	5.57757	9.47661	0	hypothetical protein, conserved
PVX_099555	0	0	0	0	0	0	0	0	0	0	0	0	3-oxoacyl-[acyl-carrier-protein] reductase, putative (FabG)
PVX_099560	0	0	0	0	0	0	0	107.657	58.738	81.6801	77.1336	0	DNA-directed RNA polymerase II, putative
PVX_099565	0	139.804	0	0	0	0	0	0	0	70.174	119.375	0	OTU-like cysteine protease, putative
PVX_099570	0	0	0	0	13.9283	0	67.1433	0	16.639	15.4314	26.2249	0	NADPH-cytochrome p450 reductase, putative (CPR)
PVX_099575	0	0	0	0	0	0	0	0	0	0	8.29003	0	perforin-like protein 3 (PLP3)
PVX_099580	0	0	0	70.6269	0	113.066	0	0	16.7547	23.3142	26.4105	35.7785	hypothetical protein, conserved
PVX_099585	0	0	0	0	0	0	0	0	0	0	0	0	cyclin-dependent kinases regulatory subunit, putative
PVX_099590	0	0	216.962	0	0	0	0	121.09	66.0649	30.6194	138.801	141.272	lipoate-protein ligase 2, putative (LipL2)
PVX_099595	0	0	0	0	44.4689	0	0	0	0	0	0	0	conserved protein, unknown function
PVX_099600	62.2452	0	309.665	0	39.4992	0	95.2189	86.4537	141.514	131.215	161.069	201.634	thioredoxin reductase 2, putative
PVX_099605	220.559	308.633	0	708.335	0	0	0	0	83.6643	77.4296	131.735	0	RNA-binding protein, putative
PVX_099610	0	0	0	1833.71	0	0	0	0	0	0	0	0	hypothetical protein
PVX_099615	0	0	0	0	9.01329	0	0	0	0	9.9906	0	46.005	patatin-like phospholipase, putative
PVX_099620	0	0	0	0	35.7625	0	0	0	21.3566	39.6075	44.8774	0	hypothetical protein, conserved
PVX_099625	0	23.2317	0	0	10.5532	0	0	0	12.6094	0	6.62502	0	hypothetical protein, conserved
PVX_099630	93.4406	0	0	0	29.6702	0	0	0	70.8275	98.4729	55.8006	0	thiamine pyrophosphokinase, putative
PVX_099635	30.1858	0	0	192.849	9.57005	0	0	0	45.7414	21.2144	42.0577	48.847	conserved Plasmodium protein, unknown function
PVX_099640	0	0	63.2347	0	0	0	0	0	0	0	0	0	hypothetical protein, conserved
PVX_099645	14.2898	0	0	0	4.52865	0	0	0	0	0	2.84422	0	hypothetical protein, conserved
PVX_099650	0	36.9102	131.473	0	0	0	80.8508	73.4234	40.0628	37.1514	84.1871	85.607	splicing factor 3A subunit 3, putative
PVX_099655	0	0	0	0	0	0	0	0	0	0	0	0	telomeric repeat binding factor 1, putative
PVX_099660	0	0	0	0	0	0	0	0	0	0	0	0	alpha-tubulin N-acetyltransferase, putative
PVX_099665	0	0	0	0	0	0	0	0	0	0	0	0	hypothetical protein, conserved
PVX_099670	0	0	0	0	0	0	0	0	0	0	0	0	hypothetical protein, conserved
PVX_099675	0	149.181	0	0	0	0	0	98.9136	0	25.0177	14.1745	0	hypothetical protein, conserved
PVX_099680	0	0	0	0	19.9161	0	0	87.1818	23.7843	88.2129	124.942	0	proline--tRNA ligase, putative
PVX_099685	0	21.7431	0	0	19.7535	0	0	43.2551	47.2066	21.8936	12.4013	0	protein phosphatase-beta, putative
PVX_099690	0	0	0	0	0	0	697.175	1255.13	171.012	157.837	89.6421	0	thioredoxin-like protein 2, putative (TLP2)

Gene ID / Patient	Patient 1	Patient 2	Patient 3	Patient 4	Patient 5	Patient 6	Patient 7	Patient 8	Patient 9	Patient 10	Patient 11	Patient 12	Gene Description
PVX_099695	0	0	0	0	0	0	0	373.672	0	47.2113	53.5193	0	zinc binding protein (Yippee), putative
PVX_099700	59.6322	166.544	0	0	0	0	91.2141	165.645	45.1904	20.9515	59.349	0	histone deacetylase, putative (HDAC1)
PVX_099705	22.1587	0	0	0	14.0475	0	0	0	8.39396	23.3604	13.2314	0	hypothetical protein, conserved
PVX_099710	106.317	594.214	0	340.345	33.7697	0	162.867	295.461	282.083	336.108	105.819	0	hypothetical protein, conserved
PVX_099715	26.7964	0	0	0	8.49478	0	40.9453	0	30.4533	28.249	69.3368	43.3582	protein kinase, putative
PVX_099725	0	13.0902	0	29.946	5.94469	0	0	13.0215	24.8709	13.186	28.0049	45.5105	protein kinase, putative
PVX_099735	0	0	0	0	0	0	0	0	0	0	130.206	0	hypothetical protein
PVX_099740	67.5484	0	0	0	10.7087	0	0	0	12.7951	0	20.1676	0	protein kinase, putative
PVX_099745	0	62.9853	0	0	14.3078	0	0	62.6481	0	0	35.9184	0	transporter, putative
PVX_099750	60.7455	14.1306	0	0	0	0	0	28.112	0	14.2312	32.2421	0	hypothetical protein, conserved
PVX_099755	0	0	0	0	0	0	0	0	0	0	0	0	hypothetical protein
PVX_099760	0	0	0	0	0	0	0	0	10.2617	0	0	0	hypothetical protein, conserved
PVX_099765	37.2772	26.0199	185.321	119.101	47.2814	0	56.979	51.7624	84.735	39.2958	111.297	120.669	glutamine-dependent NAD(+) synthetase, putative (NADSYN)
PVX_099770	55.5902	19.3989	69.0722	0	8.8116	0	0	0	0	0	5.53239	0	hypothetical protein
PVX_099780	0	0	0	0	0	0	0	0	54.4826	100.937	171.646	0	replication termination factor, putative
PVX_099785	0	0	0	0	106.76	0	515.759	0	0	117.269	199.66	0	nuclear movement protein, putative
PVX_099790	0	165.351	0	1518.33	75.2905	0	363.465	657.476	179.276	0	658.6	384.581	hypothetical protein, conserved
PVX_099800	0	0	0	0	0	0	0	0	0	8.94246	20.2606	0	zinc finger protein, putative
PVX_099805	0	0	115.306	0	0	0	0	0	52.7115	65.1795	36.9236	0	fumarate hydratase, putative
PVX_099810	0	0	0	0	0	0	0	0	0	0	7.53277	0	hypothetical protein, conserved
PVX_099815	0	0	0	0	0	0	0	0	0	0	0	0	cytoplasmic dynein light chain, putative
PVX_099820	0	39.8807	0	182.601	36.2425	0	87.3647	0	0	40.1382	22.7395	92.5027	hypothetical protein, conserved
PVX_099825	124.166	0	309.396	0	78.9124	636.764	380.634	0	141.205	43.6109	123.587	0	serine/threonine protein phosphatase 4, putative (PPP4)
PVX_099830	0	0	252.742	0	32.2339	0	155.454	0	76.9364	35.652	60.6108	0	cytochrome c oxidase subunit 5B, putative (COX5B)
PVX_099835	0	0	0	0	0	0	0	0	628.83	1146.42	0	0	hypothetical protein
PVX_099840	103.85	217.652	0	0	65.9679	0	159.074	0	0	109.437	20.6726	0	phosphatidylserine decarboxylase, putative (PSD)
PVX_099845	0	161.501	0	0	0	0	0	0	0	81.0198	137.852	0	cytochrome c oxidase subunit 6B, putative (COX6B)
PVX_099850	0	0	0	0	0	0	0	0	0	0	36.0704	0	hypothetical protein, conserved
PVX_099855	0	0	0	0	0	0	0	0	0	17.818	50.4701	0	hypothetical protein, conserved
PVX_099860	0	0	0	0	0	0	0	0	87.5542	0	45.9506	0	hypothetical protein
PVX_099870	126.268	0	314.653	0	0	0	0	0	47.866	44.3488	0	0	hypothetical protein, conserved
PVX_099875	0	0	0	0	0	0	0	0	0	0	0	0	hypothetical protein, conserved
PVX_099880	0	0	0	0	0	0	0	0	0	0	0	0	hypothetical protein, conserved
PVX_099885	0	0	36.7903	0	4.69358	0	0	20.5604	5.60993	10.4092	38.321	0	hypothetical protein
PVX_099890	0	39.8045	0	0	18.0866	0	0	158.36	43.2032	0	102.132	0	serine/threonine protein kinase, putative
PVX_099895	230.797	161.501	0	0	0	0	0	0	0	0	137.852	0	guanylate kinase, putative
PVX_099900	0	0	0	0	0	0	147.563	0	0	33.8487	0	0	unspecified product
PVX_099905	0	0	0	264.17	0	0	0	0	31.2941	29.0097	49.3124	0	transcription initiation factor TFIID subunit 7, putative (TAF7)
PVX_099910	0	0	0	0	0	0	0	0	0	33.4527	37.913	0	hypothetical protein, conserved
PVX_099915	135.441	189.318	337.598	0	86.1018	694.821	0	188.252	51.3464	47.5683	107.849	219.822	RNA-binding protein, putative
PVX_099920	51.6778	0	0	0	0	0	0	0	0	18.1575	0	0	hypothetical protein, conserved
PVX_099930	0	31.9733	0	0	0	0	0	31.8043	17.3552	8.04986	13.6785	74.1228	high molecular weight rhoptry protein 2 (RhopH2)
PVX_099935	0	0	0	0	0	0	0	0	0	0	0	0	conserved protein, unknown function
PVX_099940	144.634	0	0	0	0	0	0	0	0	0	0	0	G2 protein, putative
PVX_099944	96.775	0	0	0	61.4631	0	0	268.917	73.3566	135.98	57.7921	0	hypothetical protein, conserved
PVX_099946	0	0	0	0	0	0	0	0	0	0	182.146	0	hypothetical protein, conserved
PVX_099955	0	0	0	0	0	0	0	0	0	0	0	0	hypothetical protein, conserved
PVX_099960	0	31.8948	0	0	0	0	0	126.896	17.3103	16.0537	72.7537	0	hypothetical protein, conserved
PVX_099965	27.5639	0	0	0	17.4765	0	0	0	31.3258	29.058	38.4046	0	hypothetical protein, conserved
PVX_099970	0	0	0	0	0	0	0	0	0	0	0	0	hypothetical protein, conserved
PVX_099975	0	0	0	0	0	0	0	0	0	0	0	0	merozoite surface protein 1 paralog (MSP1P)
PVX_099980	17.7118	0	0	0	0	0	0	0	0	0	7.05069	0	merozoite surface protein 1 (MSP1)
PVX_099985	63.4535	22.1441	78.8513	0	0	0	96.973	44.0528	48.077	22.2972	44.2049	51.3429	hypothetical protein, conserved
PVX_099990	21.8024	15.2153	0	0	6.91077	0	0	30.2698	8.25897	0	30.3769	0	diacylglycerol kinase, putative
PVX_099995	50.9714	0	0	0	0	0	0	141.575	19.3124	17.9093	40.583	82.5309	cyclophilin, putative,rotamase, putative,peptidyl-prolyl cis-trans isomerase 4, putative
PVX_100000	0	0	0	0	0	0	186.411	0	69.263	0	12.1284	0	hypothetical protein, conserved
PVX_100005	0	4.98878	17.7575	0	0	0	0	0	0	2.51274	4.26927	11.5626	hypothetical protein, conserved
PVX_100010	0	0	0	0	0	0	0	0	0	0	37.2923	0	hypothetical protein
PVX_100510	0	0	0	0	0	0	0	0	0	0	0	0	hypothetical protein, conserved
PVX_100515	0	0	0	0	0	0	0	0	5.44502	0	2.86112	0	amino acid transporter, putative
PVX_100520	0	194.748	0	0	0	0	0	0	0	0	0	0	mitosis protein dim1, putative
PVX_100525	0	0	0	0	17.4658	0	0	0	10.4355	0	5.483	0	pre-mRNA splicing factor RNA helicase, putative

Gene ID / Patient	Patient 1	Patient 2	Patient 3	Patient 4	Patient 5	Patient 6	Patient 7	Patient 8	Patient 9	Patient 10	Patient 11	Patient 12	Gene Description
PVX_100530	213.203	0	0	0	0	0	0	0	0	149.7	42.4466	346.791	hypothetical protein
PVX_100535	0	0	0	0	12.6541	0	81.3168	18.4777	5.04169	0	13.246	0	hypothetical protein, conserved
PVX_100540	50.5647	17.6447	0	80.75	0	129.273	0	35.1025	19.1549	17.7687	35.2256	0	hypothetical protein, conserved
PVX_100545	126.336	0	0	201.968	0	0	0	0	47.8709	22.1933	62.8681	0	phenylalanyl-tRNA synthetase alpha chain, putative
PVX_100550	46.3755	0	115.306	0	29.4173	0	141.813	257.608	228.416	114.064	110.771	75.0796	chaperonin CPN60, mitochondrial precursor, putative
PVX_100555	66.9983	93.567	166.678	0	42.5204	0	0	93.0597	50.7751	47.0772	106.689	0	dihydrolipoyl dehydrogenase, mitochondrial (LPD1)
PVX_100560	131.462	91.8731	327.643	0	125.346	0	201.546	365.428	49.8366	46.1717	78.5098	0	cytochrome b5, putative
PVX_100565	0	0	0	0	0	0	0	0	0	0	13.5492	0	hypothetical protein, conserved
PVX_100570	0	0	0	0	0	0	0	0	0	0	7.36459	0	F-box domain containing protein
PVX_100575	0	138.349	0	0	0	0	0	0	0	0	0	0	hypothetical protein, conserved
PVX_100580	0	25.5723	0	0	0	0	0	0	13.8796	38.6203	43.7534	0	hypothetical protein, conserved
PVX_100590	0	0	0	0	0	0	419.226	0	0	0	0	0	hypothetical protein, conserved
PVX_100595	63.1678	0	0	0	0	0	96.6331	0	47.8709	22.1933	37.7209	102.314	hypothetical protein, conserved
PVX_100600	42.1194	0	104.711	0	26.7147	0	0	175.467	95.745	44.3992	50.3021	68.181	elongation factor G, putative
PVX_100610	102.541	143.27	0	328.221	0	0	157.063	284.956	233.191	108.058	142.884	0	hypothetical protein, conserved
PVX_100620	0	0	0	0	0	0	0	0	0	0	6.86881	0	hypothetical protein
PVX_100625	52.4004	18.2854	0	0	0	0	0	0	9.92521	9.20684	31.2895	0	hypothetical protein, conserved
PVX_100630	0	0	0	0	36.4522	294.035	87.8704	0	87.0723	80.7402	45.7418	0	hypothetical protein, conserved
PVX_100635	13.4549	14.082	33.4163	21.4757	12.7898	0	10.2731	28.0164	40.7706	35.4642	44.1871	10.8793	hypothetical protein, conserved
PVX_100640	0	0	0	0	0	0	310.31	0	51.2356	71.2558	40.3712	0	homocysteine S-methyltransferase, putative
PVX_100645	201.218	140.75	0	0	0	0	618.362	279.858	152.635	70.6468	40.0601	327.187	hypothetical protein, conserved
PVX_100650	0	20.753	0	0	9.42686	0	45.4389	0	22.5287	20.8972	23.6737	0	sentrin-specific protease 1, putative (SENP1)
PVX_100655	0	0	0	0	0	0	0	275.172	37.5312	0	19.7117	0	hypothetical protein, conserved
PVX_100657	0	12.5344	22.3087	0	0	45.9034	0	0	6.80428	6.31309	10.7264	14.526	conserved Plasmodium protein, unknown function
PVX_100660	14.9987	0	0	0	4.75339	0	0	0	0	0	17.9119	24.2607	hypothetical protein, conserved
PVX_100665	0	6.02503	0	137.904	5.47448	0	13.1952	71.9136	19.6201	18.1963	26.1991	13.972	hypothetical protein, conserved
PVX_100670	0	0	0	0	0	0	0	0	0	0	0	0	aspartyl protease, putative
PVX_100675	227.38	52.9308	0	0	48.1125	0	115.994	0	143.61	106.51	165.957	0	hypothetical protein, conserved
PVX_100680	0	0	0	209.159	10.3794	0	50.0312	0	37.2055	11.5033	45.6117	0	hypothetical protein, conserved
PVX_100685	0	0	0	0	0	0	0	38.1302	0	0	27.3309	0	hypothetical protein, conserved
PVX_100690	51.2892	35.7951	191.173	81.9078	8.12942	0	39.1839	71.2112	38.8588	54.0698	86.7737	82.9863	splicing factor 3B subunit 3, putative
PVX_100695	326.763	0	0	523.861	103.936	0	250.739	227.15	0	114.747	325.256	265.38	CHCH domain containing protein
PVX_100700	0	0	0	0	0	0	0	0	0	0	0	0	PIH1 domain-containing protein, putative (PIH1)
PVX_100705	577.829	0	1446.55	0	184.363	0	0	402.174	219.29	202.784	402.681	470.949	hypothetical protein, conserved
PVX_100710	0	0	0	0	0	0	0	0	0	10.3591	0	47.7037	vacuolar-type H+ pumping pyrophosphatase, putative (VP2)
PVX_100715	40.497	56.5224	0	0	6.41799	103.518	30.9335	56.224	61.3621	49.8093	72.5447	32.7573	hypothetical protein, conserved
PVX_100720	86.5296	0	71.6789	0	0	0	0	0	76.4865	20.2709	34.4461	46.6727	tetQ family GTPase, putative
PVX_100725	0	70.1649	0	0	21.2488	0	0	0	12.6945	0	26.6787	54.2292	hypothetical protein, conserved
PVX_100730	161.661	37.6219	268.021	0	68.3769	0	329.645	299.356	81.6701	75.734	139.44	87.259	serine hydroxymethyltransferase, putative (SHMT)
PVX_100735	135.544	0	112.333	0	57.3181	0	138.157	125.486	102.708	47.6262	143.891	0	ATP synthase subunit beta, mitochondrial, putative
PVX_100740	0	0	0	0	0	0	25.0198	22.7396	0	5.75609	3.26015	0	hypothetical protein, conserved
PVX_100745	0	72.6129	86.1913	0	21.9904	0	53.0005	0	39.4118	36.5555	55.2182	112.244	pre-mRNA-splicing factor SYF1, putative
PVX_100750	90.0556	0	0	0	0	461.363	0	0	34.13	0	71.7052	0	vesicle transport v-SNARE protein VTI1, putative
PVX_100755	0	47.5314	169.345	0	0	0	104.146	0	51.5865	47.8289	0	0	clustered-asparagine-rich protein, putative
PVX_100760	0	0	0	0	0	0	0	0	0	0	22.2039	0	hypothetical protein, conserved
PVX_100765	89.7846	62.7131	0	0	28.5068	0	0	0	0	31.5406	0	291.069	hypothetical protein, conserved
PVX_100770	106.113	37.0416	0	0	0	271.512	0	0	60.3081	37.2835	105.608	0	hypothetical protein, conserved
PVX_100775	0	0	0	0	0	0	0	0	0	0	0	0	hypothetical protein, conserved
PVX_100780	0	0	0	0	0	0	0	0	0	0	0	0	p25-alpha family protein, putative
PVX_100785	0	48.416	0	0	22.0027	0	0	0	52.5461	24.359	41.4034	0	S-adenosyl-methyltransferase mraW, putative
PVX_100790	0	0	0	0	54.7839	0	0	0	0	60.6719	34.3725	0	protein-S-isoprenylcysteine O-methyltransferase, putative
PVX_100795	0	0	232.634	0	0	0	0	0	141.655	65.6486	55.8006	0	hypothetical protein, conserved
PVX_100800	52.8684	0	0	0	16.7706	0	0	73.4234	40.0628	18.5757	94.7104	0	SUMO-activating enzyme subunit 2, putative (UBA2)
PVX_100805	16.1402	0	40.0961	0	5.1153	82.5047	24.6541	22.4073	12.2277	34.0321	28.9127	26.108	hypothetical protein, conserved
PVX_100810	55.2002	38.521	0	0	0	94.0629	0	25.5453	48.7899	25.8644	21.974	0	hypothetical protein, conserved
PVX_100815	0	0	0	0	0	0	0	0	44.8275	0	23.5413	0	hypothetical protein, conserved
PVX_100820	113.516	158.511	0	0	18.0062	290.486	0	0	107.528	59.8256	56.4882	0	hypothetical protein, conserved
PVX_100825	0	23.5987	42.006	0	16.0769	0	0	0	32.0248	5.94204	23.5584	0	hypothetical protein, conserved
PVX_100830	45.4107	126.802	225.807	0	28.8046	0	277.717	63.0615	34.4097	0	45.1942	0	hypothetical protein, conserved
PVX_100835	142.334	198.97	354.849	456.104	90.4992	0	0	395.692	53.9622	49.9875	198.343	0	hypothetical protein, conserved
PVX_100840	0	0	0	0	0	0	0	0	77.7176	71.9395	122.382	0	ubiquitin-like modifier HUB1, putative (HUB1)
PVX_100845	57.5936	60.2949	0	0	18.2588	0	0	0	54.5453	101.192	120.367	0	hypothetical protein, conserved

Gene ID / Patient	Patient 1	Patient 2	Patient 3	Patient 4	Patient 5	Patient 6	Patient 7	Patient 8	Patient 9	Patient 10	Patient 11	Patient 12	Gene Description
PVX_100850	0	0	0	0	0	0	0	0	0	0	0	0	COPI associated protein, putative
PVX_100855	173.142	60.466	0	0	27.4843	0	132.534	120.269	131.234	182.473	206.791	140.313	calcyclin binding protein, putative
PVX_100860	0	0	0	0	0	0	0	0	0	0	0	0	hypothetical protein, conserved
PVX_100865	24.0654	33.5902	0	0	7.62852	0	0	33.4125	27.3491	25.3703	28.74	0	cell cycle control protein, putative
PVX_100870	0	0	0	0	0	0	0	0	0	0	25.0299	0	hypothetical protein, conserved
PVX_100875	36.0515	0	0	0	0	0	0	25.0255	6.82817	0	3.58783	0	hypothetical protein, conserved
PVX_100880	0	60.6895	0	0	0	0	0	0	16.4692	0	43.2623	0	sphingomyelin phosphodiesterase, putative
PVX_100885	148.928	34.6566	0	158.663	0	0	0	68.9411	56.4262	17.4425	98.8123	80.3762	K+ channel tetramerisation domain containing protein
PVX_100890	87.0662	60.7781	108.23	0	41.4185	0	133.109	0	65.973	152.964	103.981	140.945	long-chain-fatty-acid--CoA ligase, putative
PVX_100895	0	0	0	0	0	0	0	0	0	0	0	0	serine/threonine-protein kinase 2, putative
PVX_100900	40.9065	28.5545	0	0	12.9723	0	0	0	0	0	16.2845	0	HD domain containing protein
PVX_100905	282.386	65.751	0	301.222	0	0	0	0	107.024	165.329	18.7372	305.189	50S ribosomal protein L23, putative
PVX_100910	24.9816	0	155.134	39.8803	11.875	63.8428	0	0	14.194	8.77922	17.403	60.6082	transcription factor with AP2 domain(s), putative (ApiAP2)
PVX_100915	0	0	0	0	30.5989	0	0	0	36.5202	0	0	0	hypothetical protein, conserved
PVX_100920	0	0	0	0	0	0	0	0	0	0	0	0	hypothetical protein, conserved
PVX_100925	64.4126	0	0	102.885	0	0	0	44.7188	24.4019	22.6341	38.4626	52.1196	DNA gyrase subunit B, putative
PVX_100930	73.6719	0	0	0	23.3817	0	112.74	0	83.7529	25.8824	87.988	0	hydroxyethylthiazole kinase, putative
PVX_100935	64.552	0	0	103.107	10.2333	165.066	49.3267	0	73.3642	68.0493	77.0917	0	ATP-dependent zinc metalloprotease FTSH 1, putative (FTSH1)
PVX_100940	0	0	13.6506	0	0	0	0	0	0	0	7.65808	8.88843	hypothetical protein, conserved
PVX_100945	0	0	0	0	0	0	0	0	0	0	12.8905	0	vacuolar protein sorting-associated protein 16, putative (VPS16)
PVX_100950	0	76.4882	0	0	17.3771	0	0	0	0	0	21.8069	0	3-hydroxyisobutyryl-coenzyme A hydrolase, putative
PVX_100955	73.6113	51.4056	0	0	0	0	0	102.252	0	103.444	161.179	238.529	hypothetical protein, conserved
PVX_100960	27.5163	0	0	0	0	0	0	19.0999	5.21145	9.66994	5.4768	0	hypothetical protein, conserved
PVX_100965	0	0	0	172.042	0	0	82.312	74.7488	40.7859	18.9107	42.853	87.1539	hypothetical protein, conserved
PVX_100970	0	0	0	0	0	0	0	0	0	0	0	0	hypothetical protein, conserved
PVX_100975	0	0	0	0	18.4996	0	0	80.9865	44.1888	61.4623	81.2478	0	hypothetical protein, conserved
PVX_100980	61.9007	86.4423	0	197.91	19.6402	0	0	171.95	140.731	43.4965	98.5711	100.258	mitochondrial carrier protein, putative
PVX_100985	157.177	54.885	0	0	149.671	0	0	218.342	148.909	55.2182	93.8609	0	replication factor C subunit 4, putative
PVX_100990	0	26.1836	0	0	11.8947	191.872	57.3377	0	14.2113	26.3619	14.9329	0	DEAD/DEAH box ATP-dependent RNA helicase, putative
PVX_100995	59.1591	82.6108	0	0	0	0	0	0	44.8317	62.3559	94.2051	0	TPR domain containing protein
PVX_101000	0	0	0	0	0	0	0	0	0	0	12.2116	0	hypothetical protein, conserved
PVX_101005	0	0	5762.39	0	0	0	0	0	0	0	0	0	hypothetical protein, conserved
PVX_101010	262.481	73.3004	0	0	16.6524	268.64	160.561	72.9063	59.6711	110.67	114.942	85.0035	queuine tRNA-ribosyltransferase, putative
PVX_101015	0	197.669	0	0	29.9524	0	0	262.108	107.25	0	112.66	0	hypothetical protein, conserved
PVX_101020	0	38.1739	0	174.78	0	0	0	151.874	0	0	43.5338	88.5405	hypothetical protein, conserved
PVX_101025	78.4509	0	195.232	0	0	0	120.071	0	59.4592	137.804	93.6963	0	hypothetical protein, conserved
PVX_101030	0	50.2877	0	0	0	0	0	0	27.2883	0	28.6685	0	prenyltransferase alpha subunit, putative
PVX_101035	1485.87	778.732	925.611	396.577	432.813	634.995	569.364	344.167	1173.45	869.805	739.468	401.799	40S ribosomal protein S17, putative
PVX_101040	190.444	88.6515	315.826	0	100.712	0	0	440.859	264.598	289.945	265.357	0	rab GDP dissociation inhibitor beta, putative
PVX_101045	0	0	0	0	96.2495	0	0	1259.53	343.374	105.827	180.141	0	mitochondrial import inner membrane translocase subunit TIM13, putative (TIM13)
PVX_101050	113.318	0	0	0	35.9994	0	0	0	128.859	79.6056	112.787	0	t-SNARE, putative,syntaxin-like
PVX_101055	0	69.0982	0	0	0	0	0	0	37.4896	34.7462	39.3799	0	hypothetical protein, conserved
PVX_101060	127.71	89.2466	0	0	0	0	195.771	354.985	48.413	89.7095	50.8458	0	hypothetical protein
PVX_101065	0	0	0	0	0	0	0	0	0	0	0	0	hypothetical protein, conserved
PVX_101070	0	0	0	0	0	0	0	0	0	0	15.9947	0	hypothetical protein, conserved
PVX_101075	0	0	0	0	0	0	0	0	0	0	73.6593	0	vacuolar-sorting protein SNF7, putative
PVX_101080	350.008	305.585	0	0	27.7805	0	0	121.564	132.647	92.2173	156.762	0	translation initiation factor SUI1, putative
PVX_101085	0	0	0	0	0	0	0	0	0	0	0	0	ubiquitin-conjugating enzyme E2, putative
PVX_101090	0	0	240.177	0	61.2639	0	147.723	268.047	109.679	101.656	76.8069	0	hypothetical protein, conserved
PVX_101095	0	12.0174	0	0	0	0	0	0	0	0	0	0	double C2-like domain-containing protein, putative (DOC2)
PVX_101100	37.4174	78.3536	0	0	0	0	0	0	28.3513	26.2958	37.2387	0	glucose inhibited division protein a homologue, putative
PVX_101105	20.9402	43.8404	0	66.8734	13.2747	0	63.982	58.1451	63.4587	88.304	41.6794	0	hypothetical protein, conserved
PVX_101110	81.5119	37.9259	67.5192	260.357	34.454	0	0	0	72.0503	66.8347	75.7132	87.9283	hypothetical protein, conserved
PVX_101115	0	34.4046	0	0	0	0	37.661	0	28.0121	60.632	58.8732	0	hypothetical protein, conserved
PVX_101120	0	0	98.8425	127.047	0	0	0	0	0	0	0	0	hypothetical protein, conserved
PVX_101125	0	0	0	0	0	0	0	0	0	0	0	141.134	GTP-ase activating protein for Arf containing protein
PVX_101130	0	0	0	665.43	0	0	318.552	0	0	72.7718	165.066	0	hypothetical protein, conserved
PVX_101135	210.944	49.1013	0	224.864	22.3144	360.015	215.181	97.6693	159.869	148.22	111.971	0	hypothetical protein, conserved
PVX_101140	0	0	0	0	0	0	0	0	0	12.2947	13.9287	0	hypothetical protein, conserved
PVX_101145	0	42.6019	0	195.072	0	0	0	169.487	46.2383	0	24.2899	0	hypothetical protein, conserved
PVX_101150	0	0	71.5405	0	0	0	0	0	0	0	5.72993	0	kinesin-13, putative
PVX_101155	0	0	0	0	0	0	0	75.4269	20.5796	38.1797	21.6258	0	hypothetical protein, conserved

Gene ID / Patient	Patient 1	Patient 2	Patient 3	Patient 4	Patient 5	Patient 6	Patient 7	Patient 8	Patient 9	Patient 10	Patient 11	Patient 12	Gene Description
PVX_101160	358.321	0	0	0	0	0	0	0	136.05	0	142.704	0	NEDD8-conjugating enzyme UBC12, putative (UBC12)
PVX_101165	0	0	179.176	0	45.708	0	0	0	109.153	75.8984	86.0054	0	50S ribosomal protein L3, putative
PVX_101170	85.4959	0	425.613	273.53	27.1423	0	0	0	162.005	90.1039	51.0557	0	hypothetical protein, conserved
PVX_101175	0	0	0	0	0	0	0	0	0	7.37074	4.17478	0	kinesin motor domain containing protein
PVX_101180	87.4166	0	217.599	0	27.7534	0	0	0	0	0	0	0	ENTH domain containing protein
PVX_101185	123.141	0	0	0	39.1295	0	0	0	46.6795	0	0	0	hypothetical protein, conserved
PVX_101190	66.6987	93.1482	165.931	213.279	0	0	0	0	0	23.4334	39.8295	0	hypothetical protein, conserved
PVX_101195	0	0	0	0	0	0	0	70.5463	0	0	20.2224	0	5-aminolevulinic acid synthase, putative (ALAS)
PVX_101200	289.386	0	720.53	0	61.2639	0	147.723	268.047	36.5596	67.7704	268.824	0	actin
PVX_101205	0	0	0	0	0	0	0	0	0	0	7.86304	0	hypothetical protein
PVX_101210	0	0	0	0	10.6603	0	0	0	0	0	0	0	hypothetical protein, conserved
PVX_101215	0	0	227.164	0	0	0	0	0	0	0	54.4919	0	myosin A tail domain interacting protein MTIP, putative
PVX_101220	0	0	0	0	0	0	0	0	82.0632	0	100.582	0	hypothetical protein, conserved
PVX_101225	0	47.208	0	0	0	0	103.437	0	51.2356	118.76	26.9141	0	pre-mRNA-splicing factor, putative
PVX_101230	0	0	0	0	0	0	0	142.805	38.9545	0	40.9177	0	hypothetical protein, conserved
PVX_101235	121.634	0	0	0	38.6494	0	0	0	0	128.167	48.4267	0	hypothetical protein, conserved
PVX_101240	0	0	0	0	13.7459	0	0	0	0	15.2297	25.8819	0	rac-beta serine/threonine protein kinase, putative
PVX_101245	0	70.5823	0	0	0	0	0	0	0	0	40.2247	0	hypothetical protein, conserved
PVX_101250	0	0	0	0	39.4169	0	0	0	23.5366	43.6474	49.4566	0	hypothetical protein, conserved
PVX_101255	48.8025	0	0	0	0	0	0	0	18.5152	17.1306	9.71651	0	hypothetical protein, conserved
PVX_101257	0	44.1066	0	0	0	0	0	0	0	22.1933	37.7209	0	hypothetical protein, conserved
PVX_101260	137.391	383.757	0	219.676	21.7997	351.707	210.215	190.837	208.247	217.212	259.806	222.569	70 kDa peptidylprolyl isomerase, putative
PVX_101265	44.9771	0	0	143.733	14.2646	0	0	62.4591	0	31.6072	35.8101	0	cyclin g-associated kinase, putative
PVX_101269	0	0	0	0	24.9014	0	0	0	29.7296	27.5607	15.6161	127.123	hypothetical protein, conserved
PVX_101271	0	0	0	0	0	0	0	0	0	0	0	0	hypothetical protein, conserved
PVX_101275	0	0	0	0	0	0	0	0	0	0	0	0	hypothetical protein
PVX_101280	0	0	0	0	0	0	0	0	0	0	0	0	dipeptidyl aminopeptidase 2, putative (DPAP2)
PVX_101285	0	0	0	0	0	0	243.568	0	30.1527	0	79.1911	128.935	U3 small nucleolar RNA-associated protein 11, putative
PVX_101290	0	0	0	0	159.474	0	0	0	0	0	0	407.316	tRNA intron endonuclease, catalytic C-terminal domain containing protein
PVX_101295	100.02	139.743	0	0	0	0	0	0	0	35.1343	79.6401	162.172	methyltransferase, putative
PVX_101300	0	64.4612	0	0	0	0	0	0	0	64.8366	18.37	0	RNA-binding protein, putative
PVX_101305	0	0	0	0	0	541.07	0	0	80.0243	0	21.014	0	hypothetical protein, conserved
PVX_101310	0	0	0	0	0	0	0	0	0	0	0	0	hypothetical protein, conserved
PVX_101315	606.584	665.77	0	0	192.577	443.895	132.663	120.386	328.404	578.39	362.235	140.449	bax inhibitor 1, putative
PVX_101320	44.1778	30.8394	0	141.176	14.0109	0	270.166	0	16.7376	0	123.108	71.5172	hypothetical protein, conserved
PVX_101325	0	0	0	0	0	0	0	18.3549	0	4.6464	5.26318	0	hypothetical protein, conserved
PVX_101330	0	0	0	0	0	0	0	0	0	0	0	0	hypothetical protein, conserved
PVX_101335	167.003	116.64	0	534.27	132.539	0	127.822	232.003	316.447	117.337	465.403	270.652	26S protease regulatory subunit 8, putative (RPT6)
PVX_101340	0	0	0	0	0	0	0	73.9479	20.1745	56.1248	31.7956	0	hypothetical protein, conserved
PVX_101345	179.03	0	0	0	28.4211	0	137.054	124.363	135.7	188.676	0	0	hypothetical protein, conserved
PVX_101350	0	0	0	0	0	0	0	0	0	0	0	0	hypothetical protein, conserved
PVX_101355	0	0	71.3338	0	0	0	0	39.8551	10.8741	40.3469	34.2803	46.448	protein phosphatase 2C domain containing protein
PVX_101360	0	0	944.642	0	0	0	0	524.415	0	0	74.955	0	hypothetical protein, conserved
PVX_101362	0	0	0	0	91.2908	0	0	796.611	108.591	0	56.9739	0	cutA, putative
PVX_101365	0	0	0	0	0	0	0	0	0	0	9.36188	0	hypothetical protein, conserved
PVX_101370	0	0	0	0	0	0	0	0	0	0	0	0	hypothetical protein, conserved
PVX_101375	0	0	0	0	0	0	0	0	0	0	0	0	hypothetical protein
PVX_101380	0	480.154	0	0	0	0	0	0	0	238.959	0	0	unspecified product
PVX_101385	0	26.1917	0	39.9473	3.96499	127.9	19.1094	0	9.47856	8.79395	9.96124	0	THO complex subunit 2, putative (THO2)
PVX_101390	0	45.755	0	0	0	0	0	91.0143	24.8296	23.0217	26.0864	0	dimethyladenosine transferase, putative
PVX_101395	143.936	100.607	0	461.26	45.7608	738.592	220.76	200.077	163.711	202.199	85.9612	0	hypothetical protein, conserved
PVX_101400	0	0	0	0	0	0	0	0	0	0	4.72497	19.1976	hypothetical protein, conserved
PVX_101405	32.0678	44.7644	0	0	0	0	0	44.5265	48.5939	22.5368	19.1486	0	hypothetical protein, conserved
PVX_101410	0	90.5408	0	0	0	0	0	0	0	0	51.582	210.24	vacuolar protein sorting-associated protein 26, putative (VPS26)
PVX_101415	0	0	0	0	0	0	0	0	0	0	16.5262	0	hypothetical protein, conserved
PVX_101420	392.654	0	0	419.28	124.794	0	200.658	0	99.2354	45.9692	78.1653	0	AP-3 complex subunit sigma, putative
PVX_101425	0	152.708	0	233.123	0	0	0	0	82.8657	25.6084	58.0375	118.096	eukaryotic translation initiation factor 2b, subunit 2, putative
PVX_101430	0	0	0	0	87.0847	0	0	0	103.612	0	54.3655	0	hypothetical protein, conserved
PVX_101435	19.5967	27.3513	48.6876	0	0	0	0	27.2069	0	34.4329	15.6021	0	DNA repair protein rhp16, putative
PVX_101440	0	0	0	0	0	0	0	0	12.8777	23.8891	27.0638	0	hypothetical protein, conserved
PVX_101442	0	0	0	0	0	0	0	0	0	0	0	0	conserved Plasmodium protein, unknown function
PVX_101445	0	0	0	0	21.437	0	0	0	25.5984	47.468	67.2345	0	hypothetical protein, conserved

Gene ID / Patient	Patient 1	Patient 2	Patient 3	Patient 4	Patient 5	Patient 6	Patient 7	Patient 8	Patient 9	Patient 10	Patient 11	Patient 12	Gene Description
PVX_101450	0	0	0	0	0	0	0	0	0	0	0	0	coronin, putative
PVX_101455	0	0	0	394.387	78.2589	0	188.739	0	0	43.2511	147.079	199.79	thymidylate kinase, putative
PVX_101460	0	0	0	0	0	0	0	0	0	0	15.177	0	hypothetical protein, conserved
PVX_101465	44.5073	62.1391	0	0	0	0	0	0	0	31.2772	26.5771	0	DEAD/DEAH box ATP-dependent RNA helicase, putative
PVX_101470	36.9845	51.631	0	0	11.7275	0	0	0	14.0116	51.9831	51.5308	0	hypothetical protein
PVX_101475	47.992	0	119.33	0	30.4439	0	0	66.6479	18.1832	0	19.1053	0	tryptophan--tRNA ligase, putative
PVX_101480	0	0	0	0	14.1578	228.386	0	0	16.913	15.6854	44.4277	0	hypothetical protein, conserved
PVX_101482	0	0	0	0	0	0	0	0	0	0	0	0	tRNA Valine
PVX_101484	0	0	0	0	0	0	0	0	0	0	0	0	tRNA Glutamic acid
PVX_101485	0	0	0	0	4.31236	0	0	0	0	4.78204	0	0	rhoptry neck protein 3, putative (RON3)
PVX_101490	0	991.957	505.919	650.281	0	1041.38	0	0	230.51	284.504	0	0	hypothetical protein, conserved
PVX_101495	0	0	0	0	0	0	16.6808	0	0	0	2.17392	0	reticulocyte binding protein 3, pseudogene (RBP3)
PVX_101500	29.4459	75.3365	0	0	9.33082	0	14.9898	13.6257	29.7427	58.64	29.3041	15.8742	hypothetical protein
PVX_101503	0	0	0	0	0	0	164.237	0	0	0	0	0	VIR protein,PIR protein
PVX_101505	0	0	0	0	0	0	0	0	0	0	0	0	Pv-fam-d protein
PVX_101510	46.0178	64.2492	0	0	14.5951	0	0	63.905	87.1747	32.3384	0	0	tryptophan-rich antigen (Pv-fam-a)
PVX_101515	0	209.146	497.005	0	126.774	0	152.846	138.661	226.946	245.392	397.312	323.618	tryptophan-rich antigen (Pv-fam-a)
PVX_101520	244.224	1577.34	0	585.615	77.4871	937.572	186.793	508.807	508.969	429.032	352.444	197.775	Pv-fam-d protein
PVX_101525	0	94.6588	0	0	0	0	0	0	0	47.5683	0	219.822	tryptophan-rich antigen (Pv-fam-a)
PVX_101530	72.2445	554.953	0	0	0	0	0	100.352	27.3765	25.3811	14.3805	234.091	Plasmodium exported protein, unknown function
PVX_101535	0	58.8142	0	0	0	0	0	0	0	29.5826	0	0	Phist protein (Pf-fam-b)
PVX_101540	0	121.049	0	0	0	0	0	0	0	60.8832	0	0	Pv-fam-d protein
PVX_101545	0	0	0	0	0	0	0	0	0	42.7223	0	0	Plasmodium exported protein, unknown function
PVX_101550	285.121	0	0	0	0	0	0	0	0	0	0	0	Plasmodium exported protein, unknown function
PVX_101555	31.5034	0	0	0	19.9762	161.112	0	0	23.8692	33.2103	12.541	0	hypothetical protein
PVX_101560	0	215.747	0	0	24.5138	0	0	0	29.2675	0	0	0	variable surface protein Vir4-related,PIR protein
PVX_101562	0	0	0	0	0	0	0	0	0	0	0	0	VIR protein, pseudogene,PIR protein, pseudogene
PVX_101565	0	0	0	0	0	0	0	0	0	0	0	0	variable surface protein Vir24-related,PIR protein
PVX_101570	0	0	0	0	0	0	0	0	0	0	0	0	variable surface protein Vir5-related,PIR protein
PVX_101575	0	104.358	0	0	23.7142	0	0	0	28.3141	0	14.8728	0	hypothetical protein, conserved
PVX_101580	106.065	148.201	0	339.536	33.6894	543.643	0	0	120.606	0	63.3409	0	Pv-fam-d protein
PVX_101585	0	0	0	0	0	0	0	0	0	0	0	0	reticulocyte binding protein 2 precursor, pseudogene, putative
PVX_101590	0	0	0	0	0	0	0	0	0	0	0	0	reticulocyte-binding protein 2 (RBP2), like
PVX_101592	0	631.518	0	0	95.9238	0	0	0	228.146	210.944	0	0	Plasmodium exported protein, unknown function
PVX_101595	0	1690.85	317.349	0	80.9399	2612.54	0	0	0	178.909	0	0	Plasmodium exported protein, unknown function
PVX_101600	0	0	0	0	0	0	0	0	35.8634	33.2406	18.8362	0	variable surface protein Vir16/32-related,PIR protein
PVX_101605	0	0	242.801	0	0	0	0	0	0	0	0	0	Plasmodium exported protein, unknown function
PVX_101610	325.799	113.77	202.712	521.111	0	0	124.673	113.149	123.467	257.524	178.343	131.993	RAD protein (Pv-fam-e)
PVX_101615	0	0	0	0	0	0	0	0	0	0	0	0	variable surface protein Vir14-related,PIR protein
PVX_101617	0	0	0	0	0	0	0	0	28.3852	0	0	0	VIR protein,PIR protein
PVX_101620	0	39.2786	0	0	8.92374	0	0	0	28.4218	0	0	0	variable surface protein Vir16-related,PIR protein
PVX_101625	0	0	0	0	0	0	0	0	0	0	0	0	variable surface protein Vir18-like,PIR protein
PVX_101630	0	0	0	0	0	0	0	0	0	0	0	0	VIR protein,PIR protein
PVX_102125	0	0	0	0	0	0	0	0	0	0	0	0	variable surface protein Vir32, truncated, putative
PVX_102130	0	0	0	0	0	0	0	0	0	0	0	0	hypothetical protein
PVX_102630	0	0	0	0	0	0	0	0	0	0	0	0	variable surface protein Vir27, putative
PVX_102635	0	159.033	0	0	0	0	0	0	0	0	0	0	variable surface protein Vir10-related
PVX_102640	0	0	0	0	0	0	0	0	0	0	0	0	variable surface protein Vir12-related
PVX_102645	0	0	0	0	0	0	0	0	0	0	0	0	hypothetical protein
PVX_103145	0	0	0	0	0	0	0	0	0	0	0	0	variable surface protein Vir10, putative,PIR protein
PVX_103150	0	0	0	0	22.4736	0	0	0	0	24.8792	28.192	0	variable surface protein Vir18, putative,PIR protein
PVX_103155	0	83.2792	0	0	0	0	0	0	15.063	0	0	0	variable surface protein Vir12-related,PIR protein
PVX_103160	0	0	0	0	0	0	0	0	0	0	0	0	variable surface protein Vir10-like,PIR protein
PVX_103660	101.725	213.193	0	0	32.3074	0	0	282.686	0	107.199	60.7486	0	variable surface protein Vir16/32-related
PVX_103665	0	0	0	0	0	0	0	0	0	0	0	0	variable surface protein Vir4/32-like
PVX_103670	0	0	0	0	0	0	0	0	0	0	0	0	variable surface protein Vir6-like
PVX_103675	0	0	0	0	0	0	0	0	0	0	0	0	variable surface protein Vir23, pseudogene,putative
PVX_103680	0	0	0	0	0	0	0	0	0	0	0	0	variable surface protein Vir21, putative
PVX_104180	0	0	0	0	0	0	0	0	24.6133	0	0	0	variable surface protein Vir12, putative
PVX_104185	0	0	0	0	0	0	0	0	0	0	0	0	variable surface protein Vir16-related
PVX_104190	0	0	0	0	0	0	0	0	0	0	0	0	variable surface protein Vir12-like
PVX_104690	0	0	0	0	0	0	0	0	0	0	0	0	Pv-fam-c protein

Gene ID / Patient	Patient 1	Patient 2	Patient 3	Patient 4	Patient 5	Patient 6	Patient 7	Patient 8	Patient 9	Patient 10	Patient 11	Patient 12	Gene Description
PVX_104695	0	0	0	0	0	0	0	0	0	0	0	0	variable surface protein Vir6-like
PVX_104700	0	0	0	0	0	0	0	0	0	0	14.5449	0	variable surface protein Vir18, putative
PVX_105200	0	0	0	0	0	0	0	0	0	0	21.8117	0	Pv-fam-c protein
PVX_105205	0	0	0	0	0	0	0	0	0	0	0	0	VIR protein,PIR protein
PVX_105700	0	0	0	0	0	0	0	0	0	0	0	0	variable surface protein Vir32/4, putative
PVX_105705	0	0	0	0	0	0	0	0	0	0	0	0	hypothetical protein
PVX_105710	0	0	0	0	0	0	0	0	0	0	0	0	variable surface protein Vir1/9, putative
PVX_106210	0	0	0	0	0	0	0	0	0	0	0	0	variable surface protein Vir6, putative,PIR protein
PVX_106215	0	0	0	0	0	0	0	0	0	0	0	0	VIR protein, pseudogene,PIR protein, pseudogene
PVX_106220	0	0	0	0	0	0	0	0	0	0	0	0	variable surface protein Vir12, putative,PIR protein
PVX_106720	0	0	0	0	0	0	0	0	0	0	0	0	variable surface protein Vir33 (truncated), putative
PVX_106725	0	0	0	0	0	0	0	0	0	0	0	0	variable surface protein Vir12, putative
PVX_106730	0	0	0	0	0	0	0	0	0	0	0	0	hypothetical protein
PVX_107230	0	0	0	0	0	0	0	0	0	0	0	0	hypothetical protein
PVX_107235	0	0	0	0	0	0	0	0	0	0	0	0	variable surface protein Vir12-related
PVX_107735	0	109.674	0	0	0	0	0	0	59.5114	0	31.2595	0	hypothetical protein
PVX_107740	0	87.8651	0	0	0	0	0	0	95.3282	44.162	0	0	hypothetical protein
PVX_107745	0	0	0	0	33.333	0	0	0	39.7774	36.8638	41.7815	0	variable surface protein Vir 12-like
PVX_107750	0	0	0	0	0	0	0	0	0	0	0	0	variable surface protein Vir2/15-like
PVX_107755	0	0	0	0	0	0	0	0	0	0	0	0	variable surface protein Vir12/24-related
PVX_108255	0	0	0	0	0	0	0	0	0	0	0	0	hypothetical protein
PVX_108260	0	0	0	0	0	0	0	0	0	0	0	0	Pv-fam-c protein
PVX_108760	0	0	0	0	0	0	0	0	0	0	0	0	variable surface protein Vir35, putative
PVX_108765	0	0	0	0	0	0	0	0	0	0	0	0	hypothetical protein
PVX_108770	0	0	0	0	21.5675	0	0	0	0	23.8782	40.5858	0	variable surface protein Vir 14, putative
PVX_108775	0	0	0	0	0	0	0	0	0	0	0	0	variable surface protein Vir26, truncated, putative
PVX_109275	0	0	0	0	0	0	0	0	0	45.307	0	0	Pv-fam-d protein
PVX_109280	0	96.4128	0	441.988	0	0	0	0	52.2971	96.895	82.383	0	tryptophan-rich antigen (Pv-fam-a)
PVX_109778	0	0	0	0	0	0	0	0	0	0	0	0	variable surface protein Vir, putative
PVX_109780	0	0	0	0	0	0	0	0	0	0	0	0	variable surface protein Vir35, putative
PVX_109785	0	0	0	0	0	0	0	0	0	0	0	0	variable surface protein Vir35, putative
PVX_109790	0	0	0	0	0	0	0	0	0	51.2918	0	0	variable surface protein Vir34, putative
PVX_109795	0	0	0	0	0	0	0	0	0	0	0	0	variable surface protein Vir33, putative
PVX_110295	0	0	0	0	0	0	0	0	0	0	0	0	variable surface protein Vir22-like,PIR protein
PVX_110300	0	0	0	0	0	0	0	0	0	0	0	0	variable surface protein Vir21, putative,PIR protein
PVX_110805	0	0	0	0	0	0	0	0	0	0	0	0	ring-exported protein 4, putative
PVX_110810	0	0	0	0	0	0	0	0	0	0	0	0	Duffy receptor precursor (DBP)
PVX_110815	0	0	0	0	0	0	0	0	0	0	0	0	hypothetical protein
PVX_110820	0	0	0	0	0	0	0	0	0	0	0	0	Phist protein (Pf-fam-b)
PVX_110822	0	57.1978	0	0	0	0	0	0	0	0	0	0	VIR protein,PIR protein
PVX_110825	16.3078	125.18	0	0	31.0107	83.362	0	0	49.4188	45.8474	12.9836	0	Pv-fam-d protein
PVX_110830	0	436.164	0	0	0	0	0	0	59.1406	164.326	31.0514	0	Plasmodium exported protein, unknown function
PVX_110832	0	210.367	0	0	0	0	230.83	0	114.1	264.215	29.9548	0	Plasmodium exported protein, unknown function
PVX_110834	0	0	0	0	0	0	0	0	49.3289	0	0	0	Plasmodium exported protein, unknown function
PVX_110835	544.319	189.931	364.107	334.289	172.531	0	223.884	465.052	325.15	308.992	566.708	203.214	Plasmodium exported protein, unknown function
PVX_110840	0	209.309	0	0	0	0	0	0	0	0	0	0	Plasmodium exported protein, unknown function
PVX_110845	0	0	0	0	0	0	0	575.392	0	144.795	164.431	0	hypothetical protein
PVX_110845a	0	0	0	0	0	0	0	0	0	0	0	0	conserved Plasmodium protein, unknown function
PVX_110850	0	0	0	0	0	0	95.9515	0	59.4637	22.0626	18.7456	0	GDP dissociation inhibitor, putative
PVX_110855	117.31	0	437.662	562.548	55.8267	0	0	244.394	22.2248	41.2165	116.753	94.9925	hypothetical protein, conserved
PVX_110860	0	0	0	0	0	0	0	0	0	0	74.1191	0	hypothetical protein
PVX_110865	0	0	0	0	0	0	0	47.0031	0	0	0	0	hypothetical protein, conserved
PVX_110870	0	0	0	0	175.789	0	0	383.548	104.571	483.576	164.604	0	enhancer of rudimentary domain containing protein
PVX_110880	0	24.989	0	0	0	0	0	0	0	75.4797	28.5038	0	DNA repair helicase RAD25, putative
PVX_110885	0	0	0	0	0	0	0	0	0	0	8.69045	0	dynamin protein, putative
PVX_110890	0	0	0	98.6217	0	0	0	0	0	0	0	0	hypothetical protein, conserved
PVX_110895	63.673	0	0	0	0	0	97.6057	0	0	0	38.0258	103.319	ADP/ATP transporter on adenylate translocase, putative
PVX_110900	0	0	0	0	0	0	0	0	0	0	9.24526	0	hypothetical protein, conserved
PVX_110905	37.1688	0	0	118.754	11.786	0	56.8132	0	42.2443	39.1816	44.3894	0	pyruvate kinase, putative
PVX_110910	32.9035	11.4804	40.8704	0	10.4282	0	25.1302	0	18.6956	11.563	3.27454	0	DNA polymerase zeta catalytic subunit, putative
PVX_110915	30.405	21.217	0	0	14.454	0	0	21.1054	34.5517	21.37	24.2071	0	hypothetical protein, conserved
PVX_110920	0	0	0	0	0	0	0	0	0	0	35.8964	0	acetyl-CoA transporter, putative

Gene ID / Patient	Patient 1	Patient 2	Patient 3	Patient 4	Patient 5	Patient 6	Patient 7	Patient 8	Patient 9	Patient 10	Patient 11	Patient 12	Gene Description
PVX_110925	156.798	109.615	782.135	502.658	49.8656	0	0	0	118.903	55.0632	124.858	0	phosducin-like protein, putative (PhLP2)
PVX_110930	0	0	0	403.867	40.0696	0	0	0	95.5971	0	125.502	0	hypothetical protein, conserved
PVX_110935	0	0	0	0	0	0	0	0	0	0	0	0	hypothetical protein
PVX_110940	131.559	30.6125	218.053	0	83.4466	0	67.0445	121.795	249.218	123.27	253.135	70.9909	hypothetical protein, conserved
PVX_110945	0	0	0	0	0	0	0	0	0	0	0	0	hypothetical protein
PVX_110950	0	0	0	0	0	0	0	0	0	0	0	0	hypothetical protein, conserved
PVX_110955	0	0	0	0	0	0	0	0	0	0	0	0	hypothetical protein, conserved
PVX_110960	49.4505	0	0	0	0	0	0	0	0	17.3751	9.84301	0	hypothetical protein
PVX_110965	0	0	0	0	0	0	0	0	0	0	0	0	hypothetical protein
PVX_110970	0	0	0	0	0	0	0	0	0	0	0	0	hypothetical protein
PVX_110975	0	16.7008	59.4615	0	15.1713	0	0	0	9.06518	16.8186	19.0524	0	hypothetical protein, conserved
PVX_110980	254.889	101.665	0	349.007	23.0919	0	55.656	101.123	206.923	127.949	144.954	58.9338	methionine--tRNA ligase, putative
PVX_110985	0	0	0	0	35.5926	0	0	0	169.874	0	66.9099	0	hypothetical protein, conserved
PVX_110990	0	0	0	0	0	0	0	0	0	0	0	0	ADP-ribosylation factor-like protein, putative
PVX_110995	0	0	232.148	0	0	0	0	0	35.3399	0	18.5614	151.16	hypothetical protein, conserved
PVX_111000	67.7338	0	0	0	7.15674	0	0	31.3468	17.1056	47.6047	26.9635	0	hypothetical protein, conserved
PVX_111005	40.3531	0	0	0	25.5934	0	0	0	15.2881	28.3585	48.1926	65.3187	flavoprotein subunit of succinate dehydrogenase, putative
PVX_111010	0	0	0	0	0	0	0	0	0	21.4224	0	0	thioredoxin-like associated protein 2, putative (TLAP2)
PVX_111015	0	0	0	0	0	0	320.044	0	0	0	0	0	50S ribosomal protein L27, putative
PVX_111020	0	0	0	0	0	0	0	0	0	76.3026	0	0	hypothetical protein
PVX_111025	0	0	125.674	0	0	0	0	0	19.1487	71.0306	40.2392	81.8308	vesicle transport-related protein, putative
PVX_111030	212.696	0	531.321	0	0	0	0	591.71	0	373.361	127.037	0	ubiquitin-conjugating enzyme, putative
PVX_111035	0	0	0	0	0	0	0	0	0	0	0	0	aspartyl protease, putative
PVX_111040	221.929	154.982	0	0	70.4356	378.805	0	0	56.0662	77.968	44.1759	0	hypothetical protein, conserved
PVX_111045	81.8099	38.0646	67.7663	87.1032	17.29	0	41.6695	75.7258	51.6527	67.079	70.5622	0	DNA binding protein Myb2, putative
PVX_111050	46.6657	65.1543	116.028	0	44.4024	0	0	64.8051	88.4024	114.778	120.753	0	WD domain, G-beta repeat domain containing protein
PVX_111055	132.434	185.108	330.076	0	84.1844	0	609.132	368.135	502.057	418.618	184.545	429.849	haloacid dehalogenase-like hydrolase, putative (HAD1)
PVX_111060	0	0	0	0	54.8853	0	0	0	130.836	0	0	0	hypothetical protein, conserved
PVX_111065	0	0	0	0	0	0	0	0	0	0	13.3945	0	early transcribed membrane protein (ETRAMP)
PVX_111070	238.605	272.481	215.58	0	144.387	110.899	33.1394	150.578	361.545	282.037	185.651	35.093	S-adenosylmethionine decarboxylase-ornithine decarboxylase, putative
PVX_111075	0	46.7835	0	0	21.2602	0	0	0	25.3875	70.6159	40.0085	0	hypothetical protein, conserved
PVX_111080	21.3958	29.863	0	0	0	0	0	89.1156	48.6296	37.5937	68.138	34.6144	hypothetical protein, conserved
PVX_111085	164.698	115.028	0	0	52.2824	0	0	0	62.4154	86.7888	16.3921	0	protein phosphatases PP1 regulatory subunit sds22, putative
PVX_111090	54.4113	0	0	0	0	278.456	0	0	20.6162	19.1176	21.661	0	hypothetical protein, conserved
PVX_111095	131.923	0	164.093	0	0	0	0	91.6184	49.9888	46.3488	105.038	0	hypothetical protein, conserved
PVX_111100	0	0	0	0	0	0	0	0	0	107.308	0	0	hypothetical protein, conserved
PVX_111105	0	0	0	0	0	0	0	0	0	0	15.2159	0	phosphatidylinositol N-acetylglucosaminyltransferase subunit A, putative (PIGA)
PVX_111110	41.1132	28.6989	0	131.371	0	0	62.8502	0	31.1524	43.3387	32.7336	0	hypothetical protein, conserved
PVX_111115	0	0	0	0	0	0	0	0	0	46.1717	26.1699	0	hypothetical protein
PVX_111120	0	20.1697	0	0	9.16183	0	0	40.1255	21.8956	10.1551	23.0085	0	asparagine-rich antigen, putative
PVX_111125	0	20.2417	0	0	9.19452	0	0	40.2685	0	20.3826	17.3179	0	ribosome maturation factor RimM, putative (RimM)
PVX_111130	0	0	0	0	0	0	0	0	0	0	0	0	hypothetical protein, conserved
PVX_111135	0	0	0	0	0	0	0	0	29.3436	0	30.8267	0	hypothetical protein, conserved
PVX_111140	0	0	0	0	0	0	0	0	0	0	0	0	protein phosphatase 1, regulatory (inhibitor) subunit, putative
PVX_111145	0	0	0	0	0	0	0	0	0	43.003	0	0	hypothetical protein, conserved
PVX_111150	0	43.9143	0	100.495	9.97405	0	0	0	0	0	18.7851	0	RNA helicase, putative
PVX_111155	0	0	0	0	10.518	0	0	0	25.1347	0	13.2058	53.686	hypothetical protein, conserved
PVX_111160	0	0	0	0	0	0	0	0	0	0	0	0	hypothetical protein, conserved
PVX_111165	0	0	0	0	0	0	0	0	0	0	0	0	MORN repeat-containing protein 1, putative (MORN1)
PVX_111170	0	0	0	0	0	0	0	0	0	0	0	0	hypothetical protein, conserved
PVX_111175	0	0	0	0	0	0	0	0	0	0	20.9645	170.785	ookinete surface protein Pvs25
PVX_111180	0	0	0	0	0	0	0	0	0	0	0	0	sexual stage surface protein Pvs28
PVX_111185	0	67.7486	0	0	0	0	0	134.75	147.031	34.0687	57.9174	0	calmodulin, putative
PVX_111190	0	141.708	0	0	0	1041.38	0	0	153.673	71.1261	80.6644	0	RNA methyltransferase, putative
PVX_111195	127.377	29.6389	0	135.677	13.4652	0	0	176.883	112.604	44.7572	76.0617	68.7316	tRNA N6-adenosine threonylcarbamoyltransferase, putative (KAE1)
PVX_111200	313.186	0	311.619	200.269	39.7484	0	0	0	94.9373	198.062	274.296	0	26S proteasome regulatory subunit RPN9, putative (RPN9)
PVX_111205	0	0	0	0	0	0	0	0	0	0	0	0	hypothetical protein, conserved
PVX_111210	0	0	0	0	8.51008	0	0	0	20.3388	0	0	0	hypothetical protein, conserved
PVX_111215	0	0	0	0	0	0	0	0	28.0331	0	14.7253	0	hypothetical protein, conserved
PVX_111220	48.84	51.1279	0	0	7.74099	0	0	33.9049	9.25073	8.58136	14.5818	39.5104	RNA helicase, putative
PVX_111225	0	0	0	0	60.7671	0	0	0	0	67.0332	114.026	0	transcription elongation factor SPT4, putative (SPT4)
PVX_111230	0	0	45.2016	0	0	0	0	0	0	0	3.62139	0	hypothetical protein, conserved

Gene ID / Patient	Patient 1	Patient 2	Patient 3	Patient 4	Patient 5	Patient 6	Patient 7	Patient 8	Patient 9	Patient 10	Patient 11	Patient 12	Gene Description
PVX_111235	79.7086	55.668	0	0	25.3014	0	122.001	0	60.413	28.0024	126.932	0	Secretory protein, putative
PVX_111240	0	0	0	0	0	0	0	0	0	0	0	0	hypothetical protein, conserved
PVX_111245	1172.65	0	194.549	0	421.842	0	239.302	868.788	977.647	1757.72	840.32	506.71	adenosine deaminase, putative
PVX_111250	0	75.718	0	0	0	0	0	0	0	0	21.5739	175.764	hypothetical protein, conserved
PVX_111255	59.8715	83.6064	148.918	0	37.9908	0	91.5809	83.155	22.6859	42.0711	119.174	96.9659	hypothetical protein, conserved
PVX_111260	0	0	0	0	0	0	0	0	0	35.8186	10.1458	0	hypothetical protein, conserved
PVX_111265	0	0	0	0	0	0	82.9114	0	61.6238	57.1445	53.9557	0	WD domain, G-beta repeat domain containing protein
PVX_111270	22.2302	0	0	0	7.04646	0	0	0	0	23.4358	17.6988	35.9652	hypothetical protein, conserved
PVX_111275	0	0	0	0	11.4759	0	0	0	0	0	0	0	hypothetical protein
PVX_111280	0	0	0	0	0	0	0	0	0	0	0	0	hypothetical protein, conserved
PVX_111285	0	0	0	0	153.433	0	0	0	0	0	95.8579	0	hypothetical protein, conserved
PVX_111290	0	0	0	0	0	0	0	0	0	0	18.5421	0	merozoite TRAP-like protein, putative (MTRAP)
PVX_111292	0	0	0	0	0	0	0	0	0	0	0	0	conserved Plasmodium protein, unknown function
PVX_111295	37.7012	0	93.7154	0	0	0	0	104.703	42.8495	39.7427	22.5126	0	hypothetical protein, conserved
PVX_111300	112.889	157.749	0	361.448	71.7255	0	172.97	156.872	42.7903	0	22.4721	0	brix domain containing protein
PVX_111305	0	0	0	0	0	0	138.303	0	0	63.463	89.903	0	rRNA-processing protein EBP2, putative (EBP2)
PVX_111310	0	0	0	0	0	0	0	0	0	0	0	0	hypothetical protein, conserved
PVX_111315	0	0	0	0	29.326	0	70.6865	64.2022	140.128	64.9775	128.832	0	protoporphyrinogen oxidase, putative (PPO)
PVX_111320	0	37.4414	0	171.423	34.0243	0	0	74.4799	40.6392	18.8428	10.6747	0	hypothetical protein, conserved
PVX_111325	0	0	0	0	0	0	0	0	0	0	0	0	palmitoyltransferase, putative (DHHC10)
PVX_111330	1699.13	711.887	845.437	434.672	301.945	347.96	519.939	472.017	1081.67	907.37	771.13	330.297	60S ribosomal protein L3, putative (RPL3)
PVX_111335	0	0	0	0	0	0	0	0	0	0	105.824	0	centrin-3, putative (CEN3)
PVX_111340	0	0	0	0	0	0	0	147.205	40.1543	74.4255	42.1773	0	hypothetical protein, conserved
PVX_111345	0	0	0	1566	0	0	0	0	0	169.687	385.57	0	hypothetical protein, conserved
PVX_111350	192.096	134.187	0	0	0	0	0	0	36.4026	134.959	76.4772	0	DNA-directed RNA polymerase II, putative
PVX_111355	70.7594	0	176.053	0	44.9115	0	108.273	98.2875	53.6268	24.8595	183.103	0	merozoite capping protein 1, putative
PVX_111360	0	0	0	0	0	0	0	0	0	0	0	0	hypothetical protein, conserved
PVX_111365	0	97.8846	0	0	14.824	0	0	64.9067	35.4164	49.2675	46.516	75.6686	U3 small nucleolar ribonucleoprotein protein MPP10, putative
PVX_111370	0	0	0	0	0	0	0	0	0	5.8427	0	0	hypothetical protein
PVX_111375	0	0	0	0	0	0	0	0	0	0	0	0	biotin--[acetyl-CoA-carboxylase] synthetase, putative
PVX_111380	768.45	716.02	638.345	1640.99	284.916	0	589	1958.01	1019.58	944.644	1198.3	831.299	40S ribosomal protein S2, putative (RPS2)
PVX_111385	0	0	0	0	0	0	0	0	0	0	0	0	hypothetical protein
PVX_111387	0	0	0	0	0	0	0	0	0	0	0	0	conserved Plasmodium protein, unknown function
PVX_111390	0	0	0	0	0	0	0	0	0	0	0	0	cell division cycle protein 20 homolog, putative
PVX_111395	0	0	0	0	0	0	0	0	13.4821	0	7.08344	0	hypothetical protein, conserved
PVX_111400	0	0	0	0	0	0	0	0	22.1088	20.5008	58.072	0	hypothetical protein, conserved
PVX_111405	0	0	0	0	0	0	0	0	0	0	0	0	hypothetical protein, conserved
PVX_111410	0	72.0483	0	0	65.5106	0	0	143.3	78.1786	36.2269	123.178	0	hypothetical protein, conserved
PVX_111415	0	0	0	0	0	0	0	0	0	0	4.68912	0	conserved Plasmodium protein, unknown function
PVX_111420	0	0	0	0	0	0	0	0	0	0	0	0	hypothetical protein
PVX_111425	0	0	0	0	71.8558	0	346.856	313.791	0	79.1841	89.816	0	hypothetical protein
PVX_111430	0	0	0	0	0	0	0	0	0	0	613.585	0	cytochrome c oxidase copper chaperone, putative (COX17)
PVX_111435	0	4.08099	14.526	0	1.85324	0	0	16.2385	4.43081	4.11111	10.4774	9.4584	hypothetical protein, conserved
PVX_111440	0	0	0	0	0	0	39.835	0	0	0	10.3782	0	hypothetical protein, conserved
PVX_111445	35.2509	24.6049	0	112.62	0	0	0	146.843	53.4186	74.3204	91.2141	0	hypothetical protein, conserved
PVX_111450	0	0	0	0	0	0	0	0	42.7903	0	67.4164	0	hypothetical protein
PVX_111455	43.0307	30.0382	0	275.011	27.2934	0	0	59.7551	48.9086	15.1199	94.216	0	glutamine--fructose-6-phosphate aminotransferase [isomerizing], putative
PVX_111460	0	0	0	0	0	0	0	0	0	0	13.6303	0	hypothetical protein, conserved
PVX_111465	0	0	0	0	19.7192	0	47.5252	43.18	11.7811	54.639	18.5697	0	hypothetical protein, conserved
PVX_111470	224.031	234.536	139.183	178.898	88.7782	286.399	85.5838	155.528	201.563	98.4056	211.81	90.6268	hypothetical protein, conserved
PVX_111475	64.1182	0	0	0	0	0	0	0	24.2957	45.0542	63.8141	0	hypothetical protein, conserved
PVX_111480	0	54.9333	0	0	0	0	0	0	29.808	27.6333	62.6289	127.459	RAP protein, putative
PVX_111485	166.42	0	0	533.648	0	0	255.427	0	126.208	116.879	265.044	0	hypothetical protein, conserved
PVX_111490	0	0	203.268	0	0	0	0	0	30.9512	28.6921	32.5149	132.355	hypothetical protein, conserved
PVX_111495	0	85.9303	306.41	0	78.151	0	0	683.599	93.2306	86.3833	293.755	0	hypothetical protein, conserved
PVX_111500	0	0	0	0	0	0	0	0	0	181.384	0	0	RNA-binding protein, putative
PVX_111510	0	0	0	0	0	0	0	0	0	7.83334	0	0	hypothetical protein
PVX_111515	48.9368	68.3271	0	156.404	15.522	0	0	0	0	34.3892	38.9629	0	hypothetical protein, conserved
PVX_111520	65.785	39.3448	116.71	60.0053	17.8678	48.0298	28.7042	52.1845	60.515	42.9353	102.879	45.5966	hypothetical protein, conserved
PVX_111525	0	0	0	0	0	0	0	0	0	0	0	0	hypothetical protein, conserved
PVX_111530	0	0	0	0	0	0	0	0	0	0	0	0	hypothetical protein, conserved
PVX_111535	0	0	0	0	0	0	0	0	0	0	0	0	hypothetical protein, conserved

Gene ID / Patient	Patient 1	Patient 2	Patient 3	Patient 4	Patient 5	Patient 6	Patient 7	Patient 8	Patient 9	Patient 10	Patient 11	Patient 12	Gene Description
PVX_111540	1023.57	0	2613.99	0	664.586	0	0	1434.69	390.329	715.917	407.985	1702.06	hypothetical protein
PVX_111545	0	0	559.408	0	71.3135	0	0	0	84.9219	78.5905	222.854	0	mitotic spindle assembly checkpoint protein, putative
PVX_111550	0	0	0	0	0	0	0	0	654.128	1787.75	340.797	0	hypothetical protein, conserved
PVX_111555	96.1512	67.1656	0	0	30.533	0	0	133.591	145.767	101.328	38.2797	0	orotidine 5'-phosphate decarboxylase, putative (OMPDC)
PVX_111560	5.72877	0	0	0	1.81515	0	8.74777	7.95241	8.67952	4.02664	0	0	dynein heavy chain, putative
PVX_111565	0	0	186.86	0	0	0	0	0	0	0	29.8954	0	hypothetical protein, conserved
PVX_111570	0	0	0	0	53.0372	0	0	231.812	63.2219	117.096	33.1923	0	hypothetical protein, conserved
PVX_111575	39.0842	0	0	0	37.1817	0	0	108.545	44.4219	41.2004	54.4566	63.2625	4-hydroxy-3-methylbut-2-en-1-yl diphosphate synthase, putative (GcpE)
PVX_111580	0	0	0	0	0	0	0	0	0	0	28.5577	0	phospholipid scramblase, putative
PVX_111590	69.7599	73.0376	86.6957	0	11.0596	0	0	0	13.2141	49.0256	34.7132	0	kelch domain-containing protein
PVX_111595	21.6717	0	0	0	0	0	0	0	3.28427	3.04594	16.8233	35.0824	citrate synthase, mitochondrial precursor, putative
PVX_111600	49.5326	69.1596	246.333	0	0	0	0	68.7884	56.3012	104.423	69.0155	0	serine/arginine-rich splicing factor 4, putative (SRSF4)
PVX_112100	0	0	0	0	0	0	0	0	0	0	0	0	hypothetical protein
PVX_112105	120.468	0	149.822	0	0	308.309	0	0	45.6468	21.1629	23.9793	0	Phist protein (Pf-fam-b)
PVX_112110	189.228	528.402	117.625	0	15.0044	0	72.333	131.392	233.006	216.087	103.58	0	Phist protein (Pf-fam-b)
PVX_112115	0	0	0	0	0	0	0	0	0	0	0	0	variable surface protein Vir6, putative
PVX_112120	0	0	0	0	0	0	0	0	0	0	0	0	Pv-fam-c protein
PVX_112125	0	0	0	0	0	0	0	0	0	0	0	0	variable surface protein Vir18, putative
PVX_112625	0	0	0	0	77.9826	0	0	0	0	0	0	0	Pvstp1, truncated, putative
PVX_112630	0	0	0	163.476	0	0	0	71.0304	0	0	40.7222	0	variable surface protein Vir12, putative
PVX_112635	0	0	0	0	0	0	0	0	0	74.6722	0	0	hypothetical protein
PVX_112640	0	0	0	0	0	0	0	0	0	0	0	0	hypothetical protein
PVX_112645	0	0	0	0	0	0	0	0	0	0	0	0	variable surface protein Vir17-like
PVX_112650	0	0	0	0	0	0	0	627.565	0	157.837	89.6421	0	hypothetical protein
PVX_112655	0	63.3046	0	144.9	14.3804	0	0	0	0	47.7951	36.1004	0	tryptophan-rich antigen (Pv-fam-a)
PVX_112660	142.108	99.3269	0	0	0	0	0	0	53.8765	0	28.2897	0	tryptophan-rich antigen (Pv-fam-a)
PVX_112665	0	857.801	0	0	73.0991	393.135	0	0	0	80.9097	61.1245	124.391	tryptophan-rich antigen (Pv-fam-a)
PVX_112670	252.984	1448.25	251.632	0	128.393	1035.62	154.742	140.534	191.704	337.777	151.068	0	tryptophan-rich antigen (Pv-fam-a)
PVX_112675	0	86.3505	0	131.841	0	0	0	0	46.8547	14.4781	8.20359	66.7886	tryptophan-rich antigen (Pv-fam-a)
PVX_112680	234.674	327.652	116.698	0	44.6588	240.141	215.289	130.358	248.955	247.371	158.818	75.9865	tryptophan/threonine-rich antigen
PVX_112685	0	209.415	0	0	23.7938	0	0	104.138	142.045	52.6751	14.9227	0	tryptophan-rich antigen (Pv-fam-a)
PVX_112690	971.663	937.796	142.154	91.3589	54.3985	585.06	87.4206	635.057	942.071	873.564	142.213	138.843	tryptophan-rich antigen (Pv-fam-a)
PVX_112695	0	66.7348	0	0	0	0	0	0	0	0	0	0	Pv-fam-d protein
PVX_112700	0	123.932	0	0	0	0	0	0	33.6222	124.662	17.6597	0	PST-A protein
PVX_112705	0	91.7381	0	0	0	0	0	0	49.7634	92.208	52.2631	0	tryptophan-rich antigen (Pv-fam-a)
PVX_112710	0	0	0	0	0	0	0	0	40.6357	37.6583	0	0	Pv-fam-d protein
PVX_112715	0	0	0	0	0	0	0	0	0	0	0	0	Pv-fam-c protein
PVX_112720	0	0	0	0	0	0	0	0	0	0	0	0	variable surface protein Vir12-related
PVX_113220	0	0	0	0	0	0	0	0	34.5475	0	0	0	VIR protein,PIR protein
PVX_113225	0	78.5767	0	0	0	576.546	0	0	0	79.0058	0	0	Plasmodium exported protein, unknown function
PVX_113230	0	0	0	0	0	0	0	127.424	0	32.2191	36.5141	0	variable surface protein Vir14-related,PIR protein
PVX_113235	393.214	1578.43	244.436	785.463	171.493	503	0	68.2592	558.681	379.943	352.207	159.161	Pv-fam-d protein
PVX_113240	177.609	0	0	0	28.1949	0	0	0	67.3112	218.375	53.0317	143.942	hypothetical protein
PVX_113245	210.138	734.036	0	336.337	100.117	538.521	0	291.988	637.186	369.071	355.56	170.383	hypothetical protein
PVX_113250	0	170.221	0	0	0	0	0	338.539	138.513	213.902	24.2463	0	hypothetical protein
PVX_113255	181.25	126.749	0	0	0	0	0	0	137.469	0	0	0	hypothetical protein, conserved
PVX_113260	0	0	0	0	0	0	0	0	0	18.7083	10.5985	0	hypothetical protein
PVX_113265	0	0	0	0	0	0	0	31.6342	8.63121	8.00683	27.2107	0	hypothetical protein, conserved
PVX_113270	22.595	15.7686	0	0	14.3243	0	0	0	0	23.8204	22.4866	36.5558	ATP-dependent RNA helicase, putative
PVX_113275	0	0	0	0	45.3219	0	0	0	162.145	150.201	141.899	0	hypothetical protein, conserved
PVX_113280	0	0	0	0	0	0	0	0	0	0	0	0	liver merozoite formation protein, putative
PVX_113285	0	0	0	105.157	0	0	0	45.7061	12.4703	0	19.6557	0	elongation factor G, putative
PVX_113290	0	0	0	0	33.0995	0	0	0	157.997	73.2128	0	0	geranylgeranyltransferase, putative
PVX_113295	0	35.3093	62.8591	0	8.01905	0	0	0	19.1658	0	20.1403	0	hypothetical protein, conserved
PVX_113300	0	0	0	0	0	0	0	116.985	63.8257	0	16.7623	0	hypothetical protein, conserved
PVX_113305	0	107.49	0	0	0	0	0	0	19.4453	0	30.6467	0	hypothetical protein, conserved
PVX_113310	144.024	0	179.176	230.303	22.854	0	0	100.029	27.2883	25.2995	28.6685	0	hypothetical protein, conserved
PVX_113315	0	0	0	0	0	0	0	0	0	52.8965	29.9709	0	hypothetical protein, conserved
PVX_113320	142.647	0	354.918	228.096	45.2701	0	0	0	27.0272	75.1728	127.774	115.55	hypothetical protein, conserved
PVX_113325	0	50.0459	0	0	22.744	0	0	0	27.1571	25.1779	99.8575	0	mitochondrial chaperone BCS1, putative
PVX_113330	177.126	41.2236	0	188.755	56.1955	0	0	0	89.4859	41.4884	105.771	0	dihydroorotate dehydrogenase, mitochondrial precursor, putative (DHODH)
PVX_113335	0	71.2864	84.616	0	53.9714	348.236	52.0317	0	77.3839	47.8509	33.8812	0	trophozoite exported protein 1, putative (TEX1)

Gene ID / Patient	Patient 1	Patient 2	Patient 3	Patient 4	Patient 5	Patient 6	Patient 7	Patient 8	Patient 9	Patient 10	Patient 11	Patient 12	Gene Description
PVX_113340	79.7086	0	198.369	0	25.3014	0	0	221.456	60.413	56.0048	47.5993	0	cation/H+ antiporter, putative
PVX_113345	12.4249	17.3399	0	0	3.93745	0	0	17.2488	18.8255	17.4658	24.7302	20.0961	ARID/BRIGHT DNA binding domain containing protein
PVX_113350	0	0	0	0	66.3168	0	0	0	52.7916	24.4727	41.5968	0	phenylalanine--tRNA ligase, putative
PVX_113355	0	0	0	0	0	0	0	0	6.43048	5.96571	20.2733	27.4606	hypothetical protein, conserved
PVX_113360	0	0	0	0	0	0	0	0	0	0	0	0	hypothetical protein, conserved
PVX_113365	39.8745	0	0	0	12.6386	0	0	27.6798	15.1047	14.0125	15.8732	0	hypothetical protein, conserved
PVX_113370	0	0	0	0	0	0	0	0	0	0	6.34269	0	transcription factor with AP2 domain(s), putative (ApiAP2)
PVX_113375	0	0	0	0	56.4142	0	0	0	0	62.2566	0	0	mitochondrial ribosomal protein L41, putative
PVX_113380	92.8954	0	115.485	148.439	29.4632	0	0	64.5023	70.3915	65.2809	92.4526	0	hypothetical protein, conserved
PVX_113385	0	0	0	0	53.3365	0	0	0	0	58.8769	33.3789	0	hypothetical protein, conserved
PVX_113390	32.0277	22.3481	106.07	34.084	6.76611	0	32.6091	14.8205	60.6575	48.774	50.9977	34.5328	hypothetical protein, conserved
PVX_113395	0	0	0	0	0	0	0	28.4598	0	0	12.2403	0	DNA helicase, putative
PVX_113400	0	0	0	0	0	0	0	0	0	0	23.7295	193.382	glyoxalase I, putative
PVX_113405	45.5697	0	0	0	0	0	0	0	8.63121	32.0273	13.6053	36.8631	RAP protein, putative
PVX_113410	0	0	0	0	0	0	0	0	0	122.285	0	0	hypothetical protein, conserved
PVX_113415	0	108.661	0	0	0	0	0	216.086	0	54.5854	0	0	50S ribosomal protein L24, putative
PVX_113420	120.408	84.0492	199.549	0	0	0	61.3543	111.467	30.4117	112.824	79.8887	64.9667	RNA-binding protein, putative
PVX_113430	0	0	0	0	0	0	0	0	0	0	164.685	0	hypothetical protein, conserved
PVX_113435	0	0	0	0	20.4623	0	0	0	0	0	51.3456	0	serine/threonine protein kinase, putative (ARK1)
PVX_113440	0	0	819.268	0	0	0	504.337	0	0	0	65.094	0	calcium-binding protein, putative
PVX_113445	0	0	0	196.548	0	0	94.0392	0	23.2939	0	24.4735	0	cyclin dependent kinase binding protein, putative
PVX_113450	0	0	0	0	5.84266	0	0	0	0	0	7.33826	0	nucleoside diphosphate kinase, putative
PVX_113455	0	0	0	0	0	0	0	0	0	0	0	0	hypothetical protein, conserved
PVX_113460	0	0	86.2623	0	0	0	0	0	6.57646	6.10111	20.7335	0	DNA repair protein RAD50, putative (RAD50)
PVX_113465	133.025	92.9674	0	426.162	0	0	203.953	924.448	100.859	140.161	52.9624	0	long chain polyunsaturated fatty acid elongation enzyme, putative
PVX_113470	0	18.7861	0	0	0	0	0	0	50.9846	28.3764	16.073	43.5539	hypothetical protein, conserved
PVX_113475	109.622	114.803	136.312	0	17.3878	0	0	0	20.7678	115.549	87.281	0	RNA-binding protein, putative
PVX_113480	0	0	0	0	0	0	0	487.381	0	0	0	0	ubiquitin-conjugating enzyme E2 I, putative
PVX_113485	0	0	397.361	0	0	0	0	0	0	0	0	0	hypothetical protein, conserved
PVX_113490	0	0	0	0	0	0	0	0	0	0	101.851	0	hypothetical protein, conserved
PVX_113495	87.7061	336.738	218.053	280.273	41.7233	0	67.0445	60.8975	99.6871	123.27	157.118	0	polypyrimidine tract binding protein, putative
PVX_113500	0	22.7227	40.4464	0	15.48	0	0	0	24.6689	28.6077	16.2029	0	hypothetical protein, conserved
PVX_113505	67.225	31.2766	55.6774	0	0	0	102.706	93.3338	84.8855	31.4981	40.1415	0	coatomer alpha subunit, putative
PVX_113510	194.653	0	0	0	123.925	0	0	0	147.649	0	193.764	0	glutaredoxin-like protein, putative
PVX_113515	69.8302	64.9781	231.346	74.34	14.7568	0	35.5632	193.903	132.264	81.7966	97.2934	37.6595	translation initiation factor IF-2, putative
PVX_113520	0	0	0	0	0	0	0	0	0	0	0	0	MYND finger protein, putative
PVX_113525	0	0	0	0	0	0	0	0	33.6222	0	17.6597	143.799	RING zinc finger protein, putative
PVX_113530	0	0	0	0	0	0	0	116.655	31.823	0	66.8605	0	uroporphyrinogen III decarboxylase, putative (UROD)
PVX_113535	52.4963	36.6502	130.547	167.798	0	0	80.2807	72.9063	59.6711	73.7798	62.6958	0	hypothetical protein, conserved
PVX_113540	68.0684	0	0	0	0	0	0	94.5471	51.5865	23.9145	40.6475	110.267	para-hydroxybenzoate--polyprenyltransferase, putative (COQ2)
PVX_113545	0	0	0	0	0	0	0	0	0	0	0	0	spindle assembly abnormal protein 6, putative (SAS6)
PVX_113550	0	0	0	0	0	0	0	0	0	0	0	0	hypothetical protein
PVX_113555	56.686	0	0	0	17.9833	0	0	157.456	42.9568	79.6665	78.9832	0	GTP-binding protein, putative
PVX_113560	74.5543	0	0	0	23.6407	0	56.979	103.525	14.1225	52.3944	66.7783	0	hypothetical protein, conserved
PVX_113565	0	0	0	0	32.1973	0	0	0	0	0	100.904	0	WD domain, G-beta repeat domain containing protein
PVX_113567	26.6527	0	0	0	0	0	0	37.0057	0	28.0976	37.135	43.1256	conserved Plasmodium protein, unknown function
PVX_113570	188.503	0	0	604.84	0	0	0	262.141	71.489	66.1869	187.64	0	hypothetical protein, conserved
PVX_113574	0	0	0	0	16.4714	0	0	0	0	18.2587	5.17102	0	hypothetical protein, conserved
PVX_113576	73.7934	0	0	0	23.4204	0	0	102.505	27.9637	103.7	132.2	0	sorting assembly machinery 50 kDa subunit, putative (SAM50)
PVX_113580	0	0	707.162	0	0	0	0	0	0	99.1544	337.525	0	hypothetical protein, conserved
PVX_113585	0	123.981	442.458	1706.14	169.243	0	272.221	0	470.644	435.796	564.753	864.3	proteasome subunit alpha type-2, putative
PVX_113590	0	0	0	0	0	0	0	0	0	0	0	0	hypothetical protein, conserved
PVX_113595	450.784	174.836	249.094	480.258	158.873	0	76.5905	208.676	588.293	351.973	378.849	162.194	T-complex protein 1, zeta subunit, putative
PVX_113600	0	42.6019	0	0	0	0	93.3326	0	46.2383	21.437	24.2899	197.64	ornithine aminotransferase, putative
PVX_113605	76.6619	0	0	0	0	0	117.183	53.2262	29.0436	40.4064	38.1478	0	hypothetical protein, conserved
PVX_113610	5.19684	21.7557	25.8124	16.589	4.93977	0	15.8708	21.6419	17.7155	10.9583	21.7215	0	hypothetical protein, conserved
PVX_113615	0	0	0	0	0	0	157.971	0	39.0893	0	41.0592	0	transporter protein, putative
PVX_113617	0	48.5289	0	0	22.054	0	0	96.5308	52.6686	73.2471	27.6666	0	citrate synthase-like protein, putative
PVX_113620	0	35.1048	0	0	31.8997	0	0	0	38.1037	17.6679	0	0	hypothetical protein, conserved
PVX_113625	0	0	0	0	0	0	0	0	17.275	0	0	0	cardiolipin synthetase, putative (CLS)
PVX_113630	0	0	0	0	0	0	0	0	0	0	0	0	hypothetical protein, conserved
PVX_113635	19.7917	13.8118	0	0	0	0	0	0	7.49722	6.95512	11.818	64.0359	hypothetical protein, conserved

Gene ID / Patient	Patient 1	Patient 2	Patient 3	Patient 4	Patient 5	Patient 6	Patient 7	Patient 8	Patient 9	Patient 10	Patient 11	Patient 12	Gene Description
PVX_113640	0	0	0	0	0	0	0	0	0	24.8989	0	0	palmitoyltransferase, putative (DHHC2)
PVX_113645	33.0127	0	0	0	5.2314	0	0	22.9158	12.5051	23.2027	29.5687	0	hypothetical protein, conserved
PVX_113650	0	0	0	324.124	64.3216	0	0	0	115.143	35.5714	80.6315	0	50S ribosomal protein L19, putative
PVX_113655	153.274	0	0	0	48.6491	0	117.288	106.458	116.167	107.695	213.569	124.177	step II splicing factor, putative
PVX_113660	0	84.2836	0	192.963	0	0	92.3235	335.314	22.8696	106.029	48.0556	97.752	hypothetical protein, conserved
PVX_113665	653.622	152.433	0	0	138.787	0	669.903	606.146	165.289	76.4881	563.9	0	histone H3, putative
PVX_113670	0	0	0	0	52.159	0	0	227.983	186.535	57.5834	32.6448	0	hypothetical protein, conserved
PVX_113675	112.724	78.6827	93.4003	0	47.6589	192.195	172.302	156.526	128.117	66.0154	97.2271	0	transketolase, putative (TK)
PVX_113680	52.0499	18.1631	129.34	0	24.7502	0	0	0	69.0117	36.5811	56.9804	0	transcription elongation factor SPT5, putative (SPT5)
PVX_113685	0	0	0	0	0	0	0	0	35.3768	32.7901	0	0	SNARE associated Golgi protein, putative
PVX_113690	0	0	0	0	0	0	0	0	0	0	0	0	hypothetical protein, conserved
PVX_113695	85.6595	59.8286	0	0	27.1943	0	0	119.002	0	30.0921	68.2045	0	transcription factor with AP2 domain(s), putative (ApiAP2)
PVX_113705	0	0	0	0	0	0	0	229.432	62.5882	87.029	115.062	0	hypothetical protein, conserved
PVX_113710	0	0	0	0	0	0	0	0	13.3389	0	21.0246	0	hypothetical protein, conserved
PVX_113720	0	0	0	0	0	0	0	0	0	0	0	0	hypothetical protein, conserved
PVX_113725	0	0	0	0	0	0	54.962	0	0	0	0	0	hypothetical protein, conserved
PVX_113725a	0	5660.63	21771.7	0	0	0	13649	11092.1	2982.93	2612.32	4576.46	0	60S ribosomal protein L39, putative
PVX_113731	19.7672	6.89643	24.5489	0	6.26384	0	15.0941	0	37.437	10.4202	13.7704	15.9846	hypothetical protein
PVX_113735	0	0	0	0	88.7198	0	0	0	0	0	0	0	hypothetical protein
PVX_113740	176.21	123.077	219.317	281.899	83.9172	0	134.889	0	33.3904	185.705	157.842	0	hypothetical protein, conserved
PVX_113745	0	0	0	0	0	0	0	0	0	0	133.106	0	hypothetical protein, conserved
PVX_113750	445.863	116.734	138.608	178.159	17.6805	0	170.478	232.211	126.703	313.309	266.246	90.2525	eukaryotic translation initiation factor 3 subunit 6 interacting protein, putative
PVX_113755	0	0	0	0	6.20315	0	0	0	0	0	0	0	hypothetical protein, conserved
PVX_113757	0	0	0	0	0	0	0	0	0	199.393	0	0	conserved protein, unknown function
PVX_113760	0	0	0	0	113.506	0	0	0	202.912	125.257	142.033	0	hypothetical protein, conserved
PVX_113765	0	0	0	0	0	0	0	0	0	0	0	0	hypothetical protein, conserved
PVX_113770	0	0	0	253.615	25.1666	0	0	0	0	0	0	0	hypothetical protein, conserved
PVX_113775	101.036	0	0	0	0	0	0	0	0	0	0	0	6-cysteine protein (P12)
PVX_113780	0	0	0	0	0	0	0	113.77	31.0362	57.5417	48.9062	0	6-cysteine protein
PVX_113785	181.827	158.664	0	290.536	28.8339	465.136	69.4999	0	120.556	95.8329	117.624	73.5905	nucleolar GTP-binding protein 1, putative
PVX_113790	122.299	85.4599	304.729	0	38.8613	627.157	187.445	339.929	46.3603	171.823	97.3829	198.42	hypothetical protein, conserved
PVX_113795	0	164.914	0	0	0	0	0	0	0	0	0	0	hypothetical protein, conserved
PVX_113797	0	0	0	0	0	0	0	0	0	0	0	0	conserved Plasmodium protein, unknown function
PVX_113800	0	0	0	0	0	0	0	0	11.0658	10.2645	5.8141	0	rhoptry protein, putative (ROP14)
PVX_113805	0	0	0	0	0	0	0	0	44.0126	0	0	0	50S ribosomal protein L18, putative
PVX_113810	63.6566	22.215	79.1039	0	0	0	0	0	60.2887	22.3685	57.0168	0	AP-3 complex subunit beta, putative
PVX_113815	0	0	0	0	0	0	0	0	0	0	0	0	hypothetical protein, conserved
PVX_113820	0	35.4637	252.634	0	64.452	0	0	70.5463	96.233	35.6967	91.0008	82.2494	syntaxin binding protein, putative
PVX_113825	60.7365	10.5947	37.7119	0	2.40565	38.7988	23.1874	10.5391	23.0054	8.00439	21.1554	24.5556	transcription factor with AP2 domain(s), putative (ApiAP2)
PVX_113830	0	0	0	0	0	0	0	21.9326	5.98431	5.55187	3.14447	0	myosin-like protein, putative
PVX_113835	0	0	0	0	0	0	0	82.3283	0	20.8266	23.5981	0	thiamin-phosphate pyrophosphorylase, putative
PVX_113844	0	0	0	0	0	0	0	0	13.5468	0	0	0	hypothetical protein, conserved
PVX_113846	32.276	22.5276	0	0	0	0	0	0	36.6821	11.3416	25.6972	52.2325	cytosolic Fe-S cluster assembly factor NAR1, putative (NAR1)
PVX_113850	70.4186	73.7275	0	0	11.1641	0	0	48.89	66.6944	61.8606	14.0164	0	transporter, putative
PVX_113855	0	0	0	0	0	0	0	226.322	0	0	97.2216	0	hypothetical protein, conserved
PVX_113860	1299.93	1249.64	2837.34	520.995	206.734	0	498.732	1129.55	1047.42	1255.34	1002.8	527.855	60S ribosomal protein L19, putative
PVX_113865	44.596	0	0	0	0	0	0	0	33.7922	0	8.87668	0	hypothetical protein, conserved
PVX_113870	0	0	0	0	0	0	0	0	0	0	0	0	hypothetical protein, conserved
PVX_113875	0	0	0	0	27.0387	0	0	0	225.943	119.681	101.723	0	endonuclease III homologue, putative
PVX_113880	29.1248	20.3273	0	0	18.4669	0	0	0	33.1	20.4688	34.7824	0	hypothetical protein, conserved
PVX_113885	0	108.851	0	0	0	0	0	0	0	0	0	0	hypothetical protein, conserved
PVX_113890	0	0	0	0	0	0	0	0	0	0	0	0	enoyl-acyl carrier protein reductase
PVX_113895	0	0	0	0	0	0	0	0	0	0	18.5421	0	hypothetical protein, conserved
PVX_113900	0	0	0	0	16.1958	261.272	78.0786	70.9087	77.3818	35.8799	40.6525	0	GPI-anchored wall transfer protein 1, putative (GWT1)
PVX_113905	21.2757	14.8456	0	0	3.37099	0	16.2464	29.5353	20.1471	22.4308	25.4079	0	hypothetical protein, conserved
PVX_113910	0	0	0	0	0	0	0	0	0	0	0	0	cdc2-like protein kinase, putative
PVX_113915	13.0939	54.821	32.5255	0	24.8971	0	19.9989	36.3552	39.6784	9.20308	26.0618	21.1785	hypothetical protein, conserved
PVX_113920	0	215.594	0	0	98.2507	0	0	0	0	324.022	122.573	501.993	RNA and export factor binding protein, putative
PVX_113925	27.8991	0	69.3308	0	0	0	0	38.7366	21.1378	9.80375	16.6593	45.1438	hypothetical protein, conserved
PVX_113930	0	0	0	0	7.09584	0	0	0	8.48006	15.7333	4.4557	36.2173	hypothetical protein, conserved
PVX_113935	0	0	0	0	0	0	0	0	38.0365	0	0	0	pyridoxal kinase, putative (PDXK)
PVX_113940	0	0	0	0	0	0	0	0	0	127.231	72.2199	0	hypothetical protein, conserved

Gene ID / Patient	Patient 1	Patient 2	Patient 3	Patient 4	Patient 5	Patient 6	Patient 7	Patient 8	Patient 9	Patient 10	Patient 11	Patient 12	Gene Description
PVX_113945	42.2187	0	0	134.908	13.3889	215.98	0	0	31.9903	14.8346	16.8069	0	hypothetical protein, conserved
PVX_113950	201.218	0	0	0	0	0	309.181	0	76.3177	70.6468	80.1201	0	hypothetical protein, conserved
PVX_113955	49.543	0	61.5532	0	0	0	0	0	9.3839	8.70486	29.5833	0	hypothetical protein, conserved
PVX_113960	0	0	0	0	0	0	0	0	0	0	0	0	hypothetical protein
PVX_113965	0	0	0	0	0	0	0	0	0	0	0	0	hypothetical protein, conserved
PVX_113970	0	35.0456	124.827	0	15.923	0	0	0	57.0594	0	59.9525	0	hypothetical protein, conserved
PVX_113975	0	0	0	0	0	0	0	0	0	0	15.4134	0	ras GTPAse, putative
PVX_113980	0	0	0	194.54	0	0	0	0	46.1125	21.3787	36.3358	197.102	malate:quinone oxidoreductase, putative
PVX_113985	0	0	0	0	0	0	0	0	0	0	9.41543	0	hypothetical protein, conserved
PVX_113990	0	189.28	0	0	28.6798	0	276.605	125.494	102.7	95.1945	107.884	146.418	mitochondrial import receptor subunit TOM40, putative
PVX_113995	0	0	0	78.4652	0	0	37.5368	0	0	8.63318	14.6698	0	AP-2 complex subunit alpha, putative
PVX_114000	227.572	211.903	0	0	72.23	0	0	105.374	114.985	186.55	241.596	0	hypothetical protein, conserved
PVX_114005	0	378.459	0	0	0	0	0	0	0	0	0	0	hypothetical protein
PVX_114010	0	0	0	820.206	0	0	392.717	0	0	0	50.7994	0	hypothetical protein, conserved
PVX_114015	0	0	0	0	133.368	0	644.7	0	0	876.829	663.843	0	histone H2A, putative
PVX_114020	0	0	0	0	0	0	0	0	63.2219	58.5481	0	0	histone H3 variant, putative
PVX_114025	0	0	91.9701	0	0	0	0	0	28.0347	26.0023	58.9167	0	hypothetical protein, conserved
PVX_114030	0	0	0	0	17.1979	0	0	75.2925	0	19.0482	21.5823	0	hypothetical protein, conserved
PVX_114035	269.657	94.2302	336.066	0	171.423	691.667	413.461	936.999	306.685	426.181	268.403	656.475	hypothetical protein, conserved
PVX_114040	902.823	1367.38	375.22	964.576	430.613	1544.55	1384.98	1045.86	1083.95	1796.66	1258.1	977.277	60S ribosomal protein L27a, putative
PVX_114045	0	0	0	0	17.1045	0	0	0	0	18.9449	0	87.3117	hypothetical protein, conserved
PVX_114050	181.757	63.4782	0	290.796	0	0	0	252.518	68.8843	63.8495	361.803	147.312	malate dehydrogenase, putative
PVX_114055	144.634	101.096	0	463.505	45.9835	0	221.835	201.048	274.176	50.7948	86.378	234.804	rhomboid protease ROM10, putative
PVX_114060	0	0	0	0	145.006	0	466.387	211.308	115.264	266.905	242.081	0	trafficking protein particle complex subunit 6A, putative
PVX_114065	0	0	0	0	20.5779	0	49.5953	90.119	12.2939	11.4032	58.1333	0	hypothetical protein, conserved
PVX_114070	0	0	131.942	0	0	0	0	0	0	37.2835	42.2433	0	N-acetylglucosamine transferase, putative
PVX_114075	66.4266	15.4525	0	0	7.01855	113.206	0	30.7417	16.7755	23.343	57.2935	0	hypothetical protein, conserved
PVX_114080	0	0	0	0	0	0	0	0	121.037	0	0	0	hypothetical protein, conserved
PVX_114085	62.1967	21.7053	0	0	0	0	0	43.18	0	21.8556	24.7596	0	hypothetical protein, conserved
PVX_114090	9.13781	0	0	0	2.89554	0	0	0	3.4612	0	9.09381	0	hypothetical protein, conserved
PVX_114095	118.495	110.284	196.375	378.615	125.253	202.047	181.135	383.934	284.319	319.215	416.682	0	cell division cycle protein 48 homologue, putative
PVX_114100	119.587	16.6918	59.4297	76.3878	37.908	0	0	33.2071	81.543	100.858	76.1689	38.6968	acyl-CoA synthetase, putative
PVX_114105	0	30.7937	0	0	0	0	0	0	66.8513	15.4999	35.1217	0	hypothetical protein, conserved
PVX_114110	0	0	0	0	11.3929	0	0	0	0	12.6254	28.6068	0	hypothetical protein, conserved
PVX_114115	0	9.15291	0	0	12.4705	0	0	0	24.8426	0	13.0538	0	hypothetical protein, conserved
PVX_114120	0	0	0	0	0	0	0	0	0	140.348	79.5832	0	splicing factor 3a subunit, putative
PVX_114125	0	112.54	0	257.734	76.7258	0	0	0	0	28.3048	64.1515	0	hypothetical protein, conserved
PVX_114130	0	0	0	0	0	0	244.897	221.875	60.5131	0	31.7714	0	hypothetical protein, conserved
PVX_114135	0	0	0	0	0	0	0	0	0	0	0	0	hypothetical protein, conserved
PVX_114140	43.2804	0	0	0	0	0	0	0	0	0	17.2296	0	hypothetical protein, conserved
PVX_114145	0	0	0	0	0	0	0	0	0	0	0	0	merozoite surface protein 10, putative
PVX_114150	63.1678	44.1066	0	0	60.1284	0	0	0	23.9354	0	50.2945	0	cleavage stimulation factor subunit 1, putative
PVX_114155	0	0	0	0	0	0	0	0	58.2262	0	91.7151	0	hypothetical protein, conserved
PVX_114160	0	0	255.063	0	32.5298	0	156.882	0	38.8207	35.9783	81.5544	0	DnaJ domain containing protein
PVX_114163	0	0	0	0	0	0	0	0	0	0	0	0	tRNA Alanine
PVX_114165	0	0	0	0	0	0	0	0	0	0	0	0	hypothetical protein
PVX_114167	0	0	0	0	0	0	0	0	0	0	0	0	tRNA Leucine
PVX_114170	0	60.6011	0	0	0	0	0	60.277	49.3357	30.5037	34.5595	0	hypothetical protein, conserved
PVX_114175	0	0	0	0	6.53938	0	63.0374	28.6435	7.81528	0	0	0	hypothetical protein, conserved
PVX_114180	126.805	443.067	0	0	120.892	0	0	528.701	288.418	356.297	151.457	0	pyridoxine biosynthesis protein PDX1, putative
PVX_114185	46.5201	162.377	115.666	0	14.7546	0	0	0	88.1266	130.765	92.5968	150.628	hypothetical protein, conserved
PVX_114190	0	0	0	0	0	0	0	0	0	0	0	0	Pfs77, putative
PVX_114195	0	0	0	414.488	20.5662	0	0	0	0	113.859	25.803	104.986	hypothetical protein, conserved
PVX_114197	0	0	0	0	0	0	0	0	0	0	0	0	tRNA Serine
PVX_114200	36.785	0	0	0	17.4883	0	0	0	27.8685	25.8538	21.965	0	hypothetical protein, conserved
PVX_114205	305.523	142.291	253.607	0	194.066	0	155.987	566.016	308.797	357.737	263.544	165.133	nascent polypeptide associated complex alpha chain, putative
PVX_114210	111.045	77.5106	92.0081	0	58.6858	189.33	169.734	51.3983	210.348	130.065	169.456	59.9098	signal recognition particle subunit SRP68, putative (SRP68)
PVX_114215	0	0	0	0	0	0	0	0	0	0	0	0	hypothetical protein, conserved
PVX_114220	0	0	0	0	0	0	0	0	0	7.72939	0	0	hypothetical protein, conserved
PVX_114225	0	28.4375	0	0	0	0	124.554	56.5712	30.8687	0	16.2178	0	tRNA modifying enzyme, putative
PVX_114230	41.2458	0	0	0	0	0	0	0	15.6264	28.9857	41.049	66.7653	vacuolar transporter chaperone, putative
PVX_114235	565.059	0	707.162	0	0	0	0	0	107.217	99.1544	112.508	0	hypothetical protein, conserved

Gene ID / Patient	Patient 1	Patient 2	Patient 3	Patient 4	Patient 5	Patient 6	Patient 7	Patient 8	Patient 9	Patient 10	Patient 11	Patient 12	Gene Description
PVX_114240	0	0	0	0	25.1666	0	0	110.139	0	0	0	0	hypothetical protein, conserved
PVX_114245	0	0	0	0	0	0	0	0	0	57.3733	32.5256	0	hypothetical protein
PVX_114250	0	0	0	0	27.4311	0	0	120.037	0	60.7069	34.3986	0	hypothetical protein, conserved
PVX_114255	46.0623	32.1461	0	0	21.9014	0	35.1877	0	113.419	40.4668	100.851	0	hypothetical protein, conserved
PVX_114260	0	13.5636	0	0	0	0	0	0	7.36298	3.4157	3.86902	0	transcription factor with AP2 domain(s), putative (ApiAP2)
PVX_114265	0	0	0	0	0	0	0	0	0	21.8237	37.0925	0	chorismate synthase
PVX_114270	114.698	53.3744	0	244.314	36.3709	0	0	53.0897	43.4538	80.6058	30.44	61.8827	hypothetical protein, conserved
PVX_114275	0	0	0	0	31.5524	0	0	0	37.6562	34.9004	138.442	0	ferredoxin--NADP reductase, putative
PVX_114280	80.4249	0	0	0	25.5292	0	123.1	0	30.4781	56.5079	32.0181	0	hypothetical protein, conserved
PVX_114285	0	0	0	0	0	0	0	0	0	0	0	0	RNA-binding protein mei2 homologue, putative
PVX_114290	234.35	0	0	0	0	0	179.562	0	88.8326	82.314	93.3021	190.079	superoxide dismutase [Fe], putative (SOD2)
PVX_114295	0	70.2095	125.037	0	15.9498	0	230.677	69.8325	38.1037	88.3394	50.0447	81.4164	hypothetical protein, conserved
PVX_114300	0	0	0	0	11.2171	0	0	49.122	53.6087	24.8616	35.2072	0	ATP-dependent DEAD box helicase, putative
PVX_114305	41.6975	19.3978	0	0	8.80964	0	0	0	21.0595	53.7298	22.1317	0	tyrosine kinase-like protein, putative (TKL4)
PVX_114310	0	0	0	0	85.7114	0	0	0	51.1142	0	134.202	0	ribonuclease H2 subunit A, putative
PVX_114315	112.164	234.933	0	0	88.9574	0	171.548	0	169.996	295.568	133.957	272.457	hexokinase, putative
PVX_114320	0	0	0	0	0	0	0	0	0	0	59.0163	0	hypothetical protein, conserved
PVX_114325	0	0	192.526	0	0	0	0	107.471	29.3182	27.1796	107.8	125.361	hypothetical protein, conserved
PVX_114330	0	0	0	0	0	0	0	0	0	52.0558	0	0	Plasmodium falciparum CPW-WPC domain containing protein
PVX_114335	45.503	31.765	0	0	0	0	0	0	0	63.9536	18.1144	73.6654	hypothetical protein, conserved
PVX_114337	0	0	0	0	0	0	0	0	0	417.385	0	0	anaphase promoting complex subunit, putative
PVX_114340	0	7.22941	0	33.0775	0	0	0	14.3829	0	14.5644	4.12434	0	SNF2 family N-terminal domain containing protein
PVX_114344	0	0	0	0	0	0	0	0	0	0	0	0	N-acetylglucosaminylphosphatidylinositol deacetylase, putative
PVX_114346	0	0	0	0	0	0	0	0	0	0	7.68885	0	hypothetical protein, conserved
PVX_114350	0	0	0	0	0	0	0	0	0	0	15.8242	0	hypothetical protein, conserved
PVX_114355	73.4301	51.2789	0	0	0	0	112.369	0	27.826	0	29.2331	0	sphingomyelin synthase 1, putative (SMS1)
PVX_114360	0	0	0	0	0	0	0	0	0	0	0	0	sphingomyelin synthase 2, putative (SMS2)
PVX_114365	0	0	0	0	0	0	0	0	0	30.8901	0	0	hypothetical protein, conserved
PVX_114370	25.8446	0	0	0	0	0	39.4896	71.7666	0	18.1638	15.4324	0	DNA polymerase 1, putative
PVX_114375	0	59.7714	0	0	0	0	0	0	0	0	0	0	hypothetical protein, conserved
PVX_114380	0	0	0	0	36.4155	0	0	0	0	40.2614	45.6351	0	hypothetical protein, conserved
PVX_114385	0	0	0	163.757	0	0	0	0	0	0	30.5941	0	poly(A) polymerase PAP, putative
PVX_114390	0	0	0	0	220.152	0	0	0	261.601	0	205.812	0	hypothetical protein, conserved
PVX_114395	0	30.0237	0	0	13.6401	0	0	0	32.5901	30.2252	0	0	hypothetical protein, conserved
PVX_114400	0	0	0	0	0	0	0	0	0	0	0	0	hypothetical protein, conserved
PVX_114405	80.6655	14.0733	100.207	64.4007	25.5678	0	92.424	27.998	91.6698	42.5205	36.1252	0	hypothetical protein, conserved
PVX_114410	0	0	0	0	26.9104	0	0	0	32.1246	29.7787	16.8735	0	oxidoreductase, short-chain dehydrogenase family, putative
PVX_114415	112.943	118.282	0	180.524	0	0	0	78.4299	106.985	59.5237	89.9248	0	hypothetical protein, conserved
PVX_114420	0	0	0	0	0	0	0	0	0	25.7342	29.1613	0	3-oxoacyl-[acyl-carrier-protein] synthase i/ii, putative
PVX_114425	0	0	0	0	0	0	0	0	0	0	0	0	hypothetical protein, conserved
PVX_114430	0	0	0	0	0	0	0	0	13.4821	0	7.08344	0	hypothetical protein, conserved
PVX_114435	0	127.266	454.215	0	57.9122	0	0	506.126	276.058	0	108.691	0	hypothetical protein, conserved
PVX_114440	0	100.092	0	0	0	0	219.326	0	54.3143	75.5338	28.5307	0	hypothetical protein, conserved
PVX_114445	884.246	617.309	488.603	471.018	296.052	0	525.817	1159.77	930.624	1121.94	557.355	238.61	pyruvate kinase, putative
PVX_114450	66.0586	46.1268	0	0	0	0	101.065	0	0	46.4172	39.4473	0	hypothetical protein, conserved
PVX_114455	0	0	0	0	0	0	0	0	0	0	0	0	hypothetical protein, conserved
PVX_114460	0	0	0	0	0	0	25.5963	0	12.6948	11.7773	10.0057	0	hypothetical protein, conserved
PVX_114465	0	0	0	0	0	0	0	0	0	0	162.189	0	troponin c-like protein, putative
PVX_114470	44.7522	31.2405	0	0	14.1932	0	68.421	124.293	50.8658	15.7246	71.262	0	E3 ubiquitin-protein ligase RNF5, putative (RNF5)
PVX_114475	0	0	0	0	0	0	0	0	0	0	51.0908	0	mitochondrial import inner membrane translocase subunit TIM22, putative (TIM22)
PVX_114480	342.966	239.544	853.679	548.637	326.645	0	656.303	833.811	519.906	481.935	682.699	555.861	protein DJ-1, putative (DJ1)
PVX_114485	0	0	0	0	55.5067	0	133.832	0	0	0	0	0	hypothetical protein, conserved
PVX_114490	83.541	19.4352	69.2013	88.9476	17.6561	0	0	77.3285	42.1967	19.5709	44.3417	45.0594	transportin, putative
PVX_114495	0	0	0	0	9.58947	0	0	0	0	21.2574	0	0	acetyl-CoA synthetase, putative (ACS)
PVX_114500	0	35.6052	126.821	0	16.1774	0	0	0	38.6468	35.839	50.7577	82.578	ribonuclease P protein subunit p29, putative (POP4)
PVX_114505	283.424	0	0	0	0	0	0	0	0	0	0	0	6-pyruvoyltetrahydropterin synthase, putative
PVX_114510	0	2.2389	0	0	0	0	0	0	0	0	0.638694	0	hypothetical protein, conserved
PVX_114512	0	16.5122	0	37.7759	3.74947	0	0	0	4.48174	12.4742	16.4849	0	eukaryotic translation initiation factor 2-alpha kinase, putative (PK4)
PVX_114515	285.749	99.7718	0	228.461	0	0	437.249	297.689	189.493	401.561	312.836	115.735	ethanolaminephosphotransferase, putative
PVX_114520	0	0	0	0	0	0	0	0	0	0	0	0	hypothetical protein, conserved
PVX_114525	0	0	0	0	0	0	0	0	0	45.1121	76.7066	0	hypothetical protein, conserved
PVX_114530	0	0	0	0	0	0	0	0	33.6222	0	0	0	hypothetical protein, conserved

Gene ID / Patient	Patient 1	Patient 2	Patient 3	Patient 4	Patient 5	Patient 6	Patient 7	Patient 8	Patient 9	Patient 10	Patient 11	Patient 12	Gene Description
PVX_114535	63.3018	44.2002	0	202.398	20.0854	0	193.677	0	0	0	25.2006	102.531	hypothetical protein, conserved
PVX_114540	0	0	120.954	0	0	0	0	67.5539	0	34.1836	48.4125	0	glutamyl-tRNA(Gln) amidotransferase subunit B, putative
PVX_114545	0	17.2023	0	0	7.81353	0	0	0	28.0121	25.9851	14.7183	0	hypothetical protein, conserved
PVX_114550	0	0	0	0	0	0	0	0	104.112	0	27.3346	0	N-acetyltransferase, putative
PVX_114555	120.408	28.0164	99.7745	128.245	25.4555	205.313	0	0	15.2058	28.2059	103.855	0	nicotinate phosphoribosyltransferase, putative (NAPRT)
PVX_114560	0	66.5215	474.203	0	30.2399	0	0	132.31	0	100.358	56.8695	0	DNAJ domain protein, putative
PVX_114565	81.1453	141.607	0	0	0	622.647	0	0	61.4854	42.7691	24.2274	65.6744	phosphatidylcholine-sterol acyltransferase precursor, putative
PVX_114570	0	34.5225	0	0	0	0	75.6156	68.6743	74.9438	86.8753	19.686	0	RNA binding function, putative
PVX_114575	34.8799	48.6917	0	111.434	0	0	53.3107	96.865	66.0704	24.5128	69.4263	0	transmembrane amino acid transporter protein, putative
PVX_114580	54.0825	37.7584	134.497	0	0	0	0	75.1104	40.9832	38.0043	32.2952	0	hypothetical protein, conserved
PVX_114585	0	15.9608	0	0	1.44959	23.379	13.9719	25.4038	5.19874	4.8237	7.28495	7.39825	SET domain containing protein
PVX_114590	84.8881	19.7487	0	0	17.941	0	86.4775	0	21.4386	39.7729	61.9531	0	hypothetical protein, conserved
PVX_114595	121.14	0	0	0	0	0	0	0	45.9206	0	0	0	sec14-like cytosolic factor or phosphatidylinositol/phosphatidylcholine transfer protein, putative
PVX_114600	0	0	0	0	0	0	0	0	0	0	24.6149	0	Plasmodium falciparum CPW-WPC domain containing protein
PVX_114605	0	0	0	0	0	0	0	0	0	0	0	0	hypothetical protein, conserved
PVX_114610	0	147.048	0	0	0	0	0	0	0	0	0	0	hypothetical protein, conserved
PVX_114615	0	0	0	0	3.34592	0	0	0	0	11.132	8.40635	0	DNA polymerase epsilon, catalytic subunit a, putative
PVX_114620	0	63.2852	0	0	0	0	0	0	0	0	0	0	hypothetical protein, conserved
PVX_114625	66.8481	0	0	0	42.425	0	0	0	50.6613	23.4859	13.3063	0	microtubule-associated protein ytm1 homologue, putative
PVX_114630	34.437	0	85.5937	0	10.919	0	52.633	0	52.185	0	68.5447	0	hypothetical protein, conserved
PVX_114635	54.5108	0	135.564	0	0	0	0	0	41.3078	0	21.7006	0	bifunctional methylenetetrahydrofolate dehydrogenase/cyclohydrolase, putative
PVX_114640	0	0	0	0	0	0	0	0	0	0	0	0	hypothetical protein, conserved
PVX_114645	0	65.0524	0	0	14.7776	0	0	0	88.2643	81.856	37.0966	0	ATP-dependent RNA helicase HAS1, putative (HAS1)
PVX_114650	0	0	167.811	0	0	0	0	0	25.5598	0	26.8532	0	hypothetical protein, conserved
PVX_114655	15.1896	0	0	0	4.81392	0	0	0	5.75372	10.676	18.1399	0	hypothetical protein, conserved
PVX_114660	53.786	0	66.8288	0	0	0	41.093	0	71.314	47.2514	53.5283	0	Ran-binding protein, putative
PVX_114665	72.4199	0	180.193	463.221	22.9837	0	110.82	0	82.3289	50.8854	43.2463	0	RNA polymerase II transcription factor B subunit 4, putative (TFB4)
PVX_114670	94.1286	131.502	0	0	0	0	0	130.778	107.024	66.1317	56.2115	152.594	ER lumen protein retaining receptor, putative (ERD2)
PVX_114675	297.935	208.262	0	0	47.3663	0	0	207.081	56.4799	261.578	59.3111	0	hypothetical protein, conserved
PVX_114680	82.1223	114.711	613.171	262.712	78.2073	0	377.116	114.084	124.487	201.95	163.47	266.172	proteasome subunit alpha type-4, putative
PVX_114685	437.75	152.92	272.583	350.364	34.7634	0	167.663	456.212	248.887	192.204	304.99	177.489	proteasome subunit alpha type-7, putative
PVX_114690	0	0	0	0	0	0	0	0	0	29.9488	50.9097	0	hypothetical protein, conserved
PVX_114695	0	0	0	0	0	0	0	0	12.3702	22.9479	25.9973	0	phosphoinositide phosphatase SAC1, putative
PVX_114700	0	109.745	65.1249	0	16.6162	0	0	72.7756	119.137	73.6763	62.5974	42.4052	FtsJ-like methyltransferase, putative
PVX_114705	0	62.966	0	0	85.8657	0	0	0	136.658	31.6676	89.7218	0	V-type proton ATPase 21 kDa proteolipid subunit, putative
PVX_114710	56.8661	198.516	141.431	181.787	0	0	86.9754	315.914	258.56	79.9195	135.83	0	adenylosuccinate synthetase, putative
PVX_114715	95.6374	133.612	0	612.128	30.3694	490.041	0	132.877	72.4937	67.1913	57.1127	0	60S ribosomal protein L7, putative
PVX_114720	0	0	0	0	0	0	0	0	0	0	0	0	hypothetical protein, conserved
PVX_114725	0	0	0	0	0	0	0	0	0	0	0	0	metacaspase 1, putative
PVX_114730	0	0	0	0	0	0	0	0	0	28.4097	32.1864	0	hypothetical protein, conserved
PVX_114735	53.8832	18.8031	0	86.0535	8.54084	0	0	0	10.2061	9.46734	69.7125	43.5933	DNA replication licensing factor MCM6, putative (MCM6)
PVX_114740	0	0	0	0	0	0	0	0	12.1878	0	19.2106	0	hypothetical protein, conserved
PVX_114745	44.3529	30.9617	0	0	0	0	0	61.592	0	15.5844	26.4848	0	histone-lysine N-methyltransferase, putative (SET6)
PVX_114747	0	0	0	0	0	0	0	0	0	0	0	0	tRNA Threonine
PVX_114750	0	66.614	118.63	0	60.5303	0	72.9509	66.2568	72.306	100.583	199.43	0	serine/threonine protein phosphatase 5, putative (PP5)
PVX_114755	0	29.8658	0	0	0	0	0	0	0	0	0	0	hypothetical protein, conserved
PVX_114760	0	0	0	0	0	0	0	28.0359	22.9485	7.09635	28.1354	0	NLI interacting factor-like phosphatase, putative (NIF3)
PVX_114765	0	0	0	0	0	0	0	0	0	0	0	0	hypothetical protein
PVX_114770	0	0	0	0	0	0	0	679.04	0	171.306	48.5826	0	splicing factor, putative
PVX_114775	80.1369	0	0	0	0	410.426	0	0	0	56.3055	15.9517	0	hypothetical protein, conserved
PVX_114780	0	0	0	61.2257	0	0	0	0	0	0	0	0	hypothetical protein, conserved
PVX_114785	0	23.4674	0	0	0	0	0	0	0	0	0	0	hypothetical protein, conserved
PVX_114790	0	57.9948	0	0	0	0	0	115.355	62.9369	116.684	49.587	0	mitochondrial import inner membrane translocase subunit TIM23, putative (TIM23)
PVX_114795	0	0	0	0	0	0	0	0	0	0	0	0	ubiquitin-conjugating enzyme, putative
PVX_114800	0	0	0	0	0	0	0	0	11.6596	0	6.12603	0	hypothetical protein, conserved
PVX_114805	0	0	0	0	0	0	0	0	0	0	0	0	hypothetical protein, conserved
PVX_114810	0	0	0	0	22.9651	0	0	0	0	0	0	0	hypothetical protein, conserved
PVX_114815	0	0	0	0	0	0	0	0	0	78.0057	0	0	DnaJ domain containing protein
PVX_114820	0	0	0	0	0	0	0	0	0	0	1.5065	0	protein kinase domain containing protein
PVX_114825	134.828	0	336.066	0	0	0	0	0	0	94.7068	53.6806	0	protein kinase Crk2
PVX_114830	1311.85	1468.65	350.616	554.662	319.937	777.02	481.008	1264.93	1536.46	1603.62	1005.66	579.527	elongation factor 1-alpha, putative
PVX_114832	1375.07	1257.09	467.501	885.539	404.875	405.07	438.818	1195.35	1491.85	1400.71	1047.57	432.578	elongation factor 1-alpha, putative

Gene ID / Patient	Patient 1	Patient 2	Patient 3	Patient 4	Patient 5	Patient 6	Patient 7	Patient 8	Patient 9	Patient 10	Patient 11	Patient 12	Gene Description
PVX_114835	0	0	0	0	18.3559	0	0	0	65.7688	20.3285	11.5168	0	glutamate--tRNA ligase, putative
PVX_114840	0	0	0	0	0	0	0	0	0	0	0	0	hypothetical protein, conserved
PVX_114845	31.693	44.2411	78.7674	101.243	30.1449	162.082	0	44.006	48.0259	66.8205	63.0827	51.2883	hypothetical protein, conserved
PVX_114850	0	25.6986	0	0	11.6743	0	0	0	13.9482	12.937	7.32823	0	helicase, putative
PVX_114855	234.425	0	0	0	0	0	0	0	0	0	46.6731	0	hypothetical protein, conserved
PVX_114860	23.652	16.5065	58.7696	75.5393	22.4922	0	0	0	53.7585	16.623	23.5386	38.267	hypothetical protein, conserved
PVX_114865	387.206	90.1231	0	206.345	20.4771	0	98.7279	179.27	244.535	113.366	205.53	0	T-complex protein 1, delta subunit, putative
PVX_114870	345.198	241.194	0	0	36.5564	0	0	0	43.6162	161.666	91.6223	0	pyrroline carboxylate reductase, putative
PVX_114875	0	9.68083	34.4624	0	0	0	0	0	21.0203	9.75088	16.5679	0	hypothetical protein, conserved
PVX_114880	0	121.379	0	0	13.786	0	66.4575	120.73	16.4692	30.5481	69.2197	0	hypothetical protein, conserved
PVX_114885	21.1831	0	0	0	0	0	0	29.4098	24.0731	14.888	16.8651	0	hypothetical protein, conserved
PVX_114890	0	0	155.919	0	19.8882	0	0	0	23.751	22.0223	12.4767	0	rhomboid protease ROM7, putative (ROM7)
PVX_114892	0	0	0	0	0	0	0	0	0	0	0	0	conserved Plasmodium protein, unknown function
PVX_114895	0	32.3912	0	0	14.7163	237.398	0	64.4353	17.5796	32.6066	27.707	0	zinc-finger domain containing protein
PVX_114900	0	0	0	82.0019	0	0	0	0	0	0	5.11019	41.5408	hypothetical protein, conserved
PVX_114905	0	0	0	0	0	0	0	0	0	0	68.8691	0	hypothetical protein, conserved
PVX_114910	1151.7	512.019	782.248	670.306	332.548	0	962.288	727.407	952.421	1250.44	812.833	1188.48	40S ribosomal protein S15, putative (RPS15)
PVX_114920	99.3535	0	247.396	317.99	31.5524	0	0	0	75.3125	139.602	39.5548	161.088	GTP-binding protein, putative
PVX_114922	10.17	42.5778	25.2604	0	6.4454	51.9772	0	28.2362	11.5566	17.8703	16.1937	16.448	hypothetical protein
PVX_114925	0	0	0	0	0	0	0	0	18.4303	0	9.68249	157.515	hypothetical protein, conserved
PVX_114930	0	0	305.988	0	39.0217	0	0	0	0	0	24.446	0	high mobility group protein B4, putative (HMGB4)
PVX_114935	0	22.3102	79.4432	0	10.1345	0	0	0	72.6565	78.6254	69.9859	0	exosome complex exonuclease RRP44, putative (DIS3)
PVX_114940	208.517	36.3939	129.633	0	66.1436	0	0	144.793	158.01	109.896	72.6338	0	RNA-binding protein, putative
PVX_114945	0	85.4599	0	0	0	0	0	0	0	0	0	0	hypothetical protein, conserved
PVX_114950	15.3987	0	0	49.1686	4.88021	0	23.5209	0	11.6659	27.0573	33.7143	0	hypothetical protein, conserved
PVX_114955	9.34486	0	0	0	0	0	0	0	0	0	1.85997	0	hypothetical protein, conserved
PVX_114960	218.94	0	0	0	0	0	0	0	0	0	0	0	ADP-ribosylation factor-like protein, putative
PVX_114965	0	0	0	50.6329	0	0	0	0	12.0132	16.7176	12.6247	51.2996	hypothetical protein, conserved
PVX_114970	0	0	0	0	86.2896	0	0	0	102.671	0	161.617	0	hypothetical protein, conserved
PVX_114975	0	121.095	0	0	0	0	0	240.799	131.343	60.8121	34.4772	0	RNA-binding protein, putative
PVX_114977	0	0	0	0	0	0	240.584	217.982	59.4517	55.0632	0	0	hypothetical protein
PVX_114980	97.7261	68.2671	0	0	0	0	0	0	0	68.658	38.9068	0	hypothetical protein, conserved
PVX_114985	0	57.8336	0	0	52.5733	0	0	0	94.1431	0	16.4831	134.197	hypothetical protein, conserved
PVX_114990	0	0	0	0	0	0	0	0	0	0	0	0	guanylyl cyclase, putative
PVX_114995	0	0	0	0	0	0	0	0	34.1333	10.5538	29.89	0	E3 SUMO-protein ligase PIAS, putative (PIAS)
PVX_115000	192.237	95.8335	68.2449	175.437	69.6486	0	41.9639	152.521	301.701	202.657	218.647	133.31	falcilysin, putative
PVX_115005	173.646	121.284	216.118	0	82.6934	0	0	0	164.521	30.5006	172.828	0	poly(A)-binding protein, putative
PVX_115010	47.9406	33.4677	0	0	0	0	0	66.5764	18.1637	33.6893	0	0	protein arginine N-methyltransferase 5, putative (PRMT5)
PVX_115015	68.7154	23.9812	85.3963	0	21.7876	0	52.5117	143.121	104.129	60.3646	102.58	0	protein transport protein Sec24A, putative (SEC24A)
PVX_115020	44.8757	10.4383	0	47.7627	0	0	22.8483	62.2999	11.3324	0	8.93199	0	hypothetical protein, conserved
PVX_115025	0	0	0	0	0	0	0	0	0	0	0	0	hypothetical protein, conserved
PVX_115030	0	0	0	0	0	0	0	0	0	0	0	0	actin-depolymerizing factor 2, putative (ADF2)
PVX_115035	0	0	0	0	0	0	0	0	0	0	0	0	conserved Plasmodium protein, unknown function
PVX_115040	450.117	0	0	0	0	0	0	0	0	157.837	0	0	hypothetical protein, conserved
PVX_115045	192.146	0	479.708	1233.18	0	0	0	267.216	218.617	67.4646	267.774	0	cytochrome c oxidase subunit 2a, putative
PVX_115050	0	0	0	0	0	0	0	0	4.44996	0	4.67659	0	hypothetical protein, conserved
PVX_115055	92.3796	0	229.985	0	58.6652	0	141.453	385.038	70.0227	194.711	165.501	0	proliferating cell nuclear antigen, putative
PVX_115060	0	0	0	0	0	0	0	0	0	158.368	134.724	0	hypothetical protein, conserved
PVX_115063	0	0	0	0	0	0	0	0	0	0	0	0	14-3-3 protein, putative (14-3-3II)
PVX_115065	0	93.2086	110.655	0	28.231	0	68.0462	0	0	93.8316	35.4361	0	RuvB-like helicase 3, putative (RUVB3)
PVX_115070	0	0	0	0	0	0	84.9212	0	0	0	33.1568	0	hypothetical protein, conserved
PVX_115075	14.4513	90.758	0	0	13.7396	147.736	0	20.0623	49.2663	25.3927	23.0109	46.75	hypothetical protein, conserved
PVX_115080	0	0	0	0	0	0	0	0	0	14.1856	24.1072	0	3'-5' exonuclease domain containing protein
PVX_115085	0	0	377.13	0	0	0	0	0	0	0	90.32	0	hypothetical protein, conserved
PVX_115090	0	0	0	70.8723	7.03422	0	0	0	0	15.5968	0	0	hypothetical protein
PVX_115095	0	0	0	0	0	0	0	0	0	0	2.81901	0	hypothetical protein, conserved
PVX_115100	0	0	0	0	0	0	0	0	8.38151	0	0	0	hypothetical protein, conserved
PVX_115105	29.6458	0	0	0	9.39873	0	0	0	0	0	17.7023	0	hypothetical protein, conserved
PVX_115110	63.6566	22.215	0	0	10.0912	0	0	44.1938	0	11.1843	25.3408	0	hypothetical protein, conserved
PVX_115115	0	0	0	0	0	0	0	0	81.865	126.497	43.0027	116.668	bifunctional polynucleotide phosphatase/kinase, putative
PVX_115120	66.1563	0	0	0	20.9926	0	0	0	25.0684	23.2429	0	0	ribosomal protein L9, putative
PVX_115125	72.7365	0	90.3987	0	0	0	0	151.5	13.7781	38.3379	115.822	0	polyubiquitin binding protein, putative (DOA1)

Gene ID / Patient	Patient 1	Patient 2	Patient 3	Patient 4	Patient 5	Patient 6	Patient 7	Patient 8	Patient 9	Patient 10	Patient 11	Patient 12	Gene Description
PVX_115130	0	0	0	162.085	16.0856	0	0	70.4263	0	106.908	40.376	0	endonuclease/exonuclease/phosphatase domain containing protein
PVX_115135	140.767	98.3878	0	0	44.7497	0	0	195.665	53.3675	197.75	140.113	0	hypothetical protein, conserved
PVX_115140	0	28.9383	103.06	0	0	0	0	0	47.1183	29.1334	49.5098	0	hypothetical protein, conserved
PVX_115145	0	0	0	0	0	0	0	0	0	0	18.6356	0	nuclear fusion protein, putative (GEX1)
PVX_115155	511.997	417.201	637.199	273.007	81.2712	0	130.633	118.548	258.713	299.774	390.68	138.301	hypothetical protein
PVX_115160	38.1349	26.6189	0	0	12.0926	0	0	0	14.4475	13.4	22.7717	0	hypothetical protein, conserved
PVX_115165	0	0	0	0	0	0	0	0	0	0	0	0	6-cysteine protein (P92)
PVX_115170	50.5541	11.7594	0	0	0	0	25.7409	0	6.38326	5.92191	20.1245	0	hypothetical protein, conserved
PVX_115175	28.1359	19.6369	0	0	0	0	0	39.0655	21.3173	29.6609	11.2004	45.5273	pre-mRNA-splicing factor ATP-dependent RNA helicase PRP16, putative (PRP16)
PVX_115180	26.8165	6.23714	0	28.5369	5.66496	45.6833	0	0	30.4725	12.5657	28.4665	0	hypothetical protein, conserved
PVX_115185	0	0	0	0	0	0	0	0	0	20.211	45.8006	0	exosome complex component RRP45, putative (RRP45)
PVX_115190	0	0	0	120.762	0	0	0	0	0	0	7.52323	0	aldehyde reductase, putative
PVX_115195	0	0	0	0	0	0	0	0	0	0	38.1567	0	hypothetical protein, conserved
PVX_115200	95.5353	0	0	0	121.348	489.516	146.299	0	108.624	33.5598	0	0	DNA-directed RNA polymerase II 23 kDa polypeptide, putative
PVX_115205	0	220.73	0	0	0	0	0	146.338	0	36.9939	0	0	ferrochelatase, putative
PVX_115210	0	57.514	0	0	26.1412	0	0	0	31.2077	0	16.3921	133.454	hypothetical protein, conserved
PVX_115215	0	91.2021	0	0	82.9528	0	0	0	148.419	183.341	25.9791	423.558	50S ribosomal protein L17, putative
PVX_115220	412.847	0	0	0	0	2135.73	0	0	0	144.795	411.078	675.337	hypothetical protein, conserved
PVX_115225	0	61.1352	0	0	0	224.022	0	0	0	15.3861	8.71597	0	hypothetical protein, conserved
PVX_115230	0	0	0	0	20.2576	0	0	0	48.3834	44.8614	12.7082	0	ubiquitin-activating enzyme, putative
PVX_115235	80.2806	56.0679	199.796	0	25.4833	0	0	0	121.694	56.4065	127.843	130.094	aminomethyltransferase, mitochondrial precursor, putative
PVX_115240	0	0	0	0	0	0	0	0	23.7843	22.0532	0	101.667	hypothetical protein, conserved
PVX_115245	0	52.9757	0	485.256	24.0767	0	0	0	0	0	45.2993	0	SNARE associated Golgi protein, putative
PVX_115250	0	0	0	0	0	0	0	0	0	31.0732	0	0	hypothetical protein, conserved
PVX_115255	131.655	184.017	0	0	251.062	0	1009.22	731.932	549.01	416.156	314.501	213.655	ubiquitin-60S ribosomal protein L40, putative
PVX_115260	0	0	0	0	0	0	0	1676.52	455.924	0	0	0	hypothetical protein, conserved
PVX_115265	0	0	0	0	0	0	0	0	0	0	0	0	hypothetical protein, conserved
PVX_115270	0	135.261	0	0	0	0	0	0	73.3452	203.705	192.507	0	hypothetical protein, conserved
PVX_115275	15.7493	0	19.558	0	4.9904	0	12.0253	21.8629	23.8617	16.6046	23.5102	12.7349	hypothetical protein, conserved
PVX_115280	28.3498	0	70.4518	90.555	26.9627	0	0	157.45	53.6985	59.7727	73.3564	0	hypothetical protein, conserved
PVX_115285	146.529	102.423	0	0	186.352	0	449.508	0	111.108	205.838	291.699	475.785	nucleoside diphosphate kinase A, putative
PVX_115290	55.7329	116.734	0	0	0	0	0	154.807	42.2342	19.5818	44.3743	0	signal recognition particle receptor, putative
PVX_115295	61.0145	0	0	390.143	19.3586	0	93.3326	169.487	69.3575	21.437	85.0148	0	hypothetical protein, conserved
PVX_115300	0	0	0	0	0	0	0	0	0	35.4911	20.1123	0	phosphatidylserine synthase, putative
PVX_115305	34.7445	121.256	86.3588	0	11.0166	0	53.1035	96.4888	78.9766	48.8353	76.0725	56.2313	hypothetical protein, conserved
PVX_115310	313.179	0	784.558	0	99.9831	0	0	0	237.747	0	62.3593	0	suppressor of kinetochore protein 1, putative (SKP1)
PVX_115315	0	102.323	0	0	0	0	0	67.8491	92.5542	17.1664	68.0735	0	U1 small nuclear ribonucleoprotein 70 kDa, putative
PVX_115320	0	0	0	0	0	0	0	0	0	0	0	0	hypothetical protein, conserved
PVX_115325	0	0	0	0	0	0	0	0	0	0	14.1408	115.09	hypothetical protein, conserved
PVX_115330	0	21.4591	0	0	9.74772	0	0	42.6903	0	0	24.4789	0	hypothetical protein, conserved
PVX_115335	0	0	0	0	0	0	0	0	0	24.1532	13.6845	0	oxidoreductase NAD-binding domain containing protein
PVX_115340	0	0	0	0	0	0	0	0	79.5547	221.183	83.563	0	ribosome biogenesis protein MRT4, putative
PVX_115345	25.204	123.13	62.6288	0	15.9794	0	38.5102	69.9875	47.7389	141.709	130.433	40.7798	alanine--tRNA ligase, putative
PVX_115350	0	0	0	0	0	0	0	0	0	0	0	0	hypothetical protein, conserved
PVX_115355	0	0	0	0	0	0	0	0	0	0	64.8144	264.41	hypothetical protein, conserved
PVX_115360	0	0	0	0	0	0	0	0	0	0	0	0	hypothetical protein
PVX_115365	0	50.9442	0	0	0	0	223.269	506.671	55.2888	102.517	145.212	118.193	26S proteasome regulatory subunit RPN11, putative
PVX_115370	0	75.4256	134.335	0	51.4066	0	0	0	61.4005	75.9168	64.5124	87.47	RNase L inhibitor, putative
PVX_115375	0	0	297.389	0	75.8512	0	548.782	165.879	0	41.9251	47.5223	580.921	hypothetical protein, conserved
PVX_115380	173.142	120.932	0	0	54.9686	0	0	120.269	98.4259	30.4122	17.2326	0	hypothetical protein, conserved
PVX_115385	0	0	0	127.217	0	0	0	110.574	30.1681	27.9801	55.4742	0	hypothetical protein, conserved
PVX_115390	1086.8	0	0	0	353.3	0	0	0	0	0	216.608	0	mitochondrial import inner membrane translocase subunit TIM9, putative (TIM9)
PVX_115395	47.8381	0	0	0	0	0	0	0	18.1248	16.8087	19.0441	77.4507	mitochondrial carrier protein, putative
PVX_115400	0	0	0	0	0	0	0	52.9865	7.22861	13.412	41.7804	30.8703	DNA repair endonuclease, putative
PVX_115405	73.6113	102.811	0	0	23.3625	0	0	0	83.684	25.8611	43.9578	0	hypothetical protein, conserved
PVX_115410	0	30.1251	0	0	0	0	0	119.856	16.3501	0	25.7696	0	glycosylphosphatidylinositol anchor attachment 1 protein, putative (GPAA1)
PVX_115415	0	54.0769	0	0	0	396.544	0	0	29.3436	27.2031	61.6534	0	hypothetical protein, conserved
PVX_115420	0	0	0	0	0	0	0	0	10.0208	0	15.7965	0	hypothetical protein, conserved
PVX_115425	0	0	0	0	0	0	0	0	0	0	0	132.355	hypothetical protein, conserved
PVX_115430	11.8743	8.28567	0	0	3.76291	0	18.1354	0	4.49779	4.17296	16.544	0	hypothetical protein, conserved
PVX_115435	0	58.6891	69.6568	0	17.7723	0	0	0	31.8557	39.3992	44.6334	0	hypothetical protein, conserved
PVX_115440	0	78.5228	0	0	0	143.83	0	39.0532	10.6553	39.5354	27.9923	0	hypothetical protein, conserved

Gene ID / Patient	Patient 1	Patient 2	Patient 3	Patient 4	Patient 5	Patient 6	Patient 7	Patient 8	Patient 9	Patient 10	Patient 11	Patient 12	Gene Description
PVX_115445	0	0	0	0	87.0847	0	0	0	0	0	0	0	U2 small nuclear ribonucleoprotein A', putative
PVX_115446	0	0	0	0	0	0	0	0	0	0	0	0	tRNA Arginine
PVX_115447	0	0	0	0	0	0	0	0	0	0	0	0	tRNA Tryptophan
PVX_115448	0	0	0	0	0	0	0	0	0	0	0	0	tRNA Arginine
PVX_115449	0	0	0	0	0	0	0	0	0	0	0	0	tRNA Glycine
PVX_115449a	0	0	0	0	0	0	0	0	0	0	0	0	tRNA Cysteine
PVX_115450	90.8787	1142.61	0	290.796	259.694	1862.36	417.443	126.259	585.517	702.344	578.885	294.625	hypothetical protein, conserved
PVX_115455	0	0	0	0	0	0	0	0	53.1166	0	0	0	Plasmodium exported protein, unknown function
PVX_115460	89.5151	62.5247	0	286.422	56.8421	458.587	0	0	33.9251	220.122	89.0936	0	Plasmodium exported protein, unknown function
PVX_115465	0	104.434	123.991	0	31.6327	0	0	0	94.4629	52.5607	39.7011	0	tryptophan-rich antigen (Pv-fam-a)
PVX_115470	109.774	664.065	90.9537	0	23.2053	374.32	167.788	152.429	152.488	128.576	80.1161	0	Pv-fam-d protein
PVX_115475	0	0	0	0	31.5524	0	0	0	0	0	0	0	variable surface protein Vir14, putative,PIR protein
PVX_115480	0	0	0	0	0	0	0	0	0	0	0	0	variable surface protein Vir21, putative,PIR protein
PVX_115490	0	0	0	0	0	0	0	0	0	0	0	0	VIR protein,PIR protein
PVX_115985	0	0	0	0	0	0	0	0	0	20.6624	0	0	variable surface protein Vir, putative
PVX_115990	0	0	0	0	0	0	0	0	0	0	0	0	hypothetical protein
PVX_116485	0	0	0	0	0	0	0	0	0	0	0	0	conserved Plasmodium protein, unknown function
PVX_116490	0	63.9858	0	0	29.0704	0	70.0702	0	34.727	128.824	45.6108	0	ribose-phosphate pyrophosphokinase, putative
PVX_116495	0	0	0	0	0	0	0	0	0	28.85	0	0	mRNA decay protein, putative
PVX_116500	0	0	0	0	0	0	0	295.855	80.6773	224.017	169.383	0	nicotinate-nucleotide adenylyltransferase, putative (NMNAT)
PVX_116505	0	0	0	0	11.6599	0	0	0	0	0	0	0	hypothetical protein, conserved
PVX_116510	0	0	0	0	0	0	108.016	196.109	133.75	49.6015	56.2062	0	hypothetical protein, conserved
PVX_116515	0	0	0	0	0	0	0	0	0	0	3.97273	0	hypothetical protein, conserved
PVX_116520	0	0	0	0	0	0	0	0	35.8092	16.6046	18.8128	0	ribonuclease P protein subunit RPR2, putative
PVX_116525	0	0	0	0	0	0	0	138.045	0	0	0	0	hypothetical protein, conserved
PVX_116530	30.1248	0	0	96.2295	9.5507	0	0	0	22.8245	63.5145	23.9845	0	hypothetical protein, conserved
PVX_116535	0	0	0	0	0	0	35.0163	0	17.3641	16.108	9.12366	0	hypothetical protein, conserved
PVX_116540	0	126.187	224.866	0	28.6798	0	138.303	0	0	0	35.9612	0	hypothetical protein, conserved
PVX_116545	0	0	0	0	39.3348	0	0	0	0	0	24.6769	0	hypothetical protein, conserved
PVX_116550	0	0	0	0	2.31945	0	0	10.1615	11.0906	2.57255	13.1127	11.8378	hypothetical protein, conserved
PVX_116555	0	0	0	0	0	0	0	0	0	0	0	0	hypothetical protein, conserved
PVX_116557	217.084	0	0	0	91.8602	0	110.73	100.514	109.683	50.8443	129.634	0	translation initiation factor EIF-2B gamma subunit, putative
PVX_116560	56.9023	79.457	141.521	181.903	18.052	0	0	0	43.1208	39.9852	67.9582	0	RNA-binding protein, putative
PVX_116565	0	0	0	0	0	0	0	0	0	0	0	0	hypothetical protein, conserved
PVX_116570	0	0	0	0	0	0	0	0	0	0	0	0	TBC domain containing protein
PVX_116575	0	0	0	0	0	0	0	0	0	0	0	0	WD domain, G-beta repeat domain containing protein
PVX_116580	0	0	0	0	0	0	0	0	99.5268	46.104	104.526	0	hypothetical protein, conserved
PVX_116582	0	0	0	0	0	0	0	0	14.9642	0	62.8954	127.867	conserved Plasmodium protein, unknown function
PVX_116585	0	9.32515	0	0	0	0	0	0	0	0	0	0	hypothetical protein, conserved
PVX_116590	0	0	0	0	0	0	0	0	6.75335	0	0	0	hypothetical protein, conserved
PVX_116595	0	0	0	0	108.393	0	0	0	128.812	357.134	67.563	0	hypothetical protein
PVX_116600	158.463	0	0	0	50.3974	0	0	0	120.168	0	0	0	mitochondrial fission 1 protein, putative (FIS1)
PVX_116603	0	42.1418	0	0	0	0	0	0	22.8696	21.2057	84.0973	0	hypothetical protein, conserved
PVX_116604	0	0	0	33.0202	0	0	0	0	0	7.26955	6.17579	16.7275	hypothetical protein, conserved
PVX_116610	264.868	92.554	330.076	0	84.1844	0	0	184.068	150.617	604.67	263.636	214.925	hypothetical protein
PVX_116615	0	0	0	0	0	0	0	0	83.9901	155.665	308.767	0	lactate/malate dehydrogenase, putative
PVX_116620	295.905	206.643	0	473.198	117.393	378.805	566.034	308.278	560.662	519.787	323.956	0	phosphoribosylpyrophosphate synthetase, putative
PVX_116625	295.594	0	0	0	0	0	0	0	112.185	207.465	0	0	U6 snRNA-associated Sm-like protein LSm6, putative
PVX_116630	3710.15	2007.08	4472.8	2299.64	1292.93	1227.38	3118.12	5488.67	4083.07	4497.97	3692.83	2524.08	lactate dehydrogenase
PVX_116635	203.77	106.694	126.677	162.824	48.4768	0	155.801	141.494	115.808	214.79	223.08	0	dihydrofolate synthase/folylpolyglutamate synthase, putative
PVX_116640	0	0	0	0	0	0	0	270.71	0	0	0	0	VAMP-like protein YKT62, putative
PVX_116645	30.9692	0	0	0	0	0	0	0	0	0	6.16419	0	hypothetical protein, conserved
PVX_116650	26.6766	18.618	0	0	0	0	0	0	10.1057	9.37423	37.1682	0	DEAD box helicase, putative
PVX_116652	0	0	0	0	0	0	0	0	91.8235	254.877	144.565	0	MSF1-like protein, putative
PVX_116655	0	0	0	0	4.32274	0	0	9.46907	0	4.79451	13.5768	0	hypothetical protein, conserved
PVX_116660	0	50.6549	0	0	0	0	111	100.759	54.975	25.484	231.021	117.521	Micro-fibrillar-associated protein 1 C-terminus domain containing protein
PVX_116665	69.334	0	0	0	0	0	0	0	0	0	69.0057	0	hypothetical protein, conserved
PVX_116670	74.9368	0	0	79.7802	7.91827	0	38.1659	104.043	0	26.3332	44.7465	40.4154	hypothetical protein, conserved
PVX_116675	90.0556	125.805	0	0	0	0	137.884	125.114	68.2601	126.543	161.337	0	hypothetical protein, conserved
PVX_116680	29.0115	0	0	0	0	0	0	80.5632	32.9712	40.7784	69.2942	46.9453	vacuolar protein sorting-associated protein 52, putative (VPS52)
PVX_116685	0	0	0	0	0	0	0	0	0	0	0	0	glideosome associated protein with multiple membrane spans 1, putative (GAPM1)
PVX_116690	192.355	44.7707	0	205.013	20.3448	0	98.09	0	97.1829	45.0542	38.2884	0	hypothetical protein, conserved

Gene ID / Patient	Patient 1	Patient 2	Patient 3	Patient 4	Patient 5	Patient 6	Patient 7	Patient 8	Patient 9	Patient 10	Patient 11	Patient 12	Gene Description
PVX_116695	127.143	44.3888	0	0	0	0	0	0	24.0885	0	37.962	0	plasmepsin V, putative (PMV)
PVX_116700	1285.34	749.358	2140.64	2063.6	409.348	1101.59	1646.5	1191.94	1056.35	977.702	1364.8	1045.38	60S ribosomal protein L23, putative (RPL23)
PVX_116705	0	127.086	0	0	0	0	0	0	0	31.9572	54.3257	0	N-acetyltransferase, putative
PVX_116710	277.603	97.012	0	0	44.1229	0	425.699	0	0	194.991	138.157	225.299	vacuolar ATP synthase subunit g, putative
PVX_116715	1452.2	677.552	0	777.771	385.67	1245.63	372.38	1010.28	1652.82	1189.43	1156.52	394.006	60S ribosomal protein L9, putative
PVX_116720	0	0	0	0	0	0	0	238.031	0	0	0	0	beta-hydroxyacyl-ACP dehydratase precursor, putative (FabZ)
PVX_116725	0	0	0	0	0	0	0	0	0	0	0	0	hypothetical protein, conserved
PVX_116730	0	0	0	0	0	0	0	0	0	0	0	0	hypothetical protein, conserved
PVX_116735	0	0	0	0	0	0	0	0	0	0	20.0444	0	hypothetical protein, conserved
PVX_116740	0	0	0	0	0	0	0	0	0	0	0	0	hypothetical protein, conserved
PVX_116745	155.843	36.267	0	0	16.4782	0	79.4405	0	19.6825	91.2614	51.7006	0	palmitoyltransferase, putative (DHHC5)
PVX_116750	0	0	0	0	0	0	0	0	0	0	0	0	hypothetical protein
PVX_116755	0	14.6409	0	0	0	0	0	0	0	0	8.35152	0	hypothetical protein, conserved
PVX_116760	9.23779	0	0	0	8.7817	0	14.1076	0	0	6.49294	22.0639	14.9399	hypothetical protein, conserved
PVX_116765	0	14.0917	0	0	0	0	0	0	0	3.54866	0	0	variant-silencing SET protein, putative (SETvs)
PVX_116770	0	0	0	0	0	0	91.0318	0	45.1002	62.729	94.7689	0	nucleoside-diphosphatase mig-23, putative
PVX_116775	122.614	42.8063	152.495	0	19.4515	0	93.781	0	116.15	43.0795	24.4064	99.2948	hypothetical protein, conserved
PVX_116780	154.364	0	0	0	98.177	0	0	214.593	58.5279	54.209	92.1898	0	protein transport protein SFT2, putative
PVX_116785	24.8472	17.3408	0	0	0	0	0	68.9962	75.3005	43.6572	54.4017	0	splicing factor 1, putative (SF1)
PVX_116790	0	79.7615	142.064	0	0	0	0	0	0	20.0691	22.7395	0	hypothetical protein, conserved
PVX_116795	0	0	0	0	0	0	0	0	0	0	0	0	hypothetical protein
PVX_116800	0	194.748	0	0	0	0	0	387.134	0	0	55.3796	0	hypothetical protein, conserved
PVX_116805	51.0587	0	126.966	0	0	0	0	70.9087	38.6909	35.8799	30.4894	0	hypothetical protein, conserved
PVX_116810	0	0	0	0	0	0	0	0	0	12.8734	0	0	hypothetical protein, conserved
PVX_116815	0	0	0	0	0	0	0	0	0	0	0	0	hypothetical protein, conserved
PVX_116820	109.572	0	0	0	0	561.674	0	152.257	0	76.9755	109.058	177.707	DnaJ domain containing protein
PVX_116825	0	0	0	139.181	0	0	0	0	66.0051	15.3038	86.693	0	hypothetical protein, conserved
PVX_116830	73.7326	0	0	0	0	0	0	0	27.9406	25.9037	29.3535	119.461	dihydroorotase, putative
PVX_116835	303.617	212.657	0	0	193.815	0	936.11	422.702	115.236	426.175	241.818	0	hypothetical protein, conserved
PVX_116840	0	0	158.025	0	20.1568	0	0	0	0	22.3193	37.9351	0	hypothetical protein, conserved
PVX_116845	90.1014	0	0	0	28.5655	0	45.8968	41.7014	0	21.1076	53.802	48.6009	DNA-directed RNA polymerase, alpha subunit, putative
PVX_116850	0	0	155.809	0	19.8742	0	0	0	23.7343	0	49.872	0	protein disulfide isomerase, putative
PVX_116855	0	0	0	0	0	0	0	0	74.31	0	0	0	hypothetical protein, conserved
PVX_116860	0	0	0	95.5535	0	0	0	0	0	0	11.908	0	M1-family alanyl aminopeptidase, putative
PVX_116865	64.395	44.9642	160.19	0	40.8656	0	0	0	73.202	45.2486	25.6358	208.61	hypothetical protein, conserved
PVX_116870	19.6743	13.7298	0	0	0	0	0	27.3146	0	6.91384	19.5798	0	histone deacetylase, putative (HDA1)
PVX_116875	120.651	168.612	300.607	0	0	618.671	0	0	0	0	48.0349	195.736	hypothetical protein, conserved
PVX_116880	0	449.615	0	0	0	0	329.301	297.984	0	0	42.6499	0	pre-mRNA-splicing factor ISY1, putative
PVX_116885	0	0	0	0	0	0	23.5452	0	0	0	0	0	hypothetical protein, conserved
PVX_116890	0	0	0	0	0	0	0	0	0	35.3714	0	0	hypothetical protein
PVX_116892	0	27.9412	0	0	0	0	0	0	0	0	0	0	conserved Plasmodium protein, unknown function
PVX_116894	0	0	0	0	0	0	0	0	0	0	0	0	conserved Plasmodium protein, unknown function
PVX_116895	0	0	0	0	0	0	0	0	0	0	0	0	hypothetical protein, conserved
PVX_116900	0	0	0	0	0	0	0	0	0	49.5234	42.0882	0	diacylglycerol kinase, putative
PVX_116905	0	0	160.19	0	20.4328	0	0	89.4416	0	0	38.4538	0	hypothetical protein, conserved
PVX_116910	0	0	0	0	0	0	0	0	18.8926	0	19.8506	0	sulfate transporter, putative
PVX_116915	0	0	0	0	70.2156	0	0	307.15	0	77.6406	0	0	exported protein 2, putative (EXP2)
PVX_116920	0	0	124.687	0	0	0	76.6765	0	0	35.2368	19.9618	0	RNA 3'-terminal phosphate cyclase-like protein, putative
PVX_116925	131.849	0	0	844.761	0	0	202.143	183.253	149.951	324.153	551.188	0	proteasome subunit beta type-2, putative
PVX_116930	0	0	127.558	0	16.2728	0	0	0	19.4461	36.0776	35.7611	0	conserved Plasmodium protein, unknown function
PVX_116935	138.267	32.1742	0	147.291	29.2353	0	70.4677	64.0037	34.9238	16.1942	36.6954	0	hypothetical protein, conserved
PVX_116940	0	0	0	0	0	0	0	82.9329	135.752	0	47.5426	0	hypothetical protein, conserved
PVX_116945	61.281	0	0	0	0	0	0	0	0	0	6.09876	0	cyclic nucleotide phosphodiesterase, putative
PVX_116950	0	0	0	0	0	0	0	0	52.1362	0	0	0	mitochondrial pyruvate carrier protein 2, putative (MPC2)
PVX_116955	0	0	0	0	0	0	0	0	0	66.622	18.8762	0	hypothetical protein, conserved
PVX_116960	261.098	182.776	0	0	0	0	0	0	99.0696	0	103.971	0	hypothetical protein, conserved
PVX_116965	0	19.2809	0	88.223	0	0	0	0	15.6995	14.5654	10.9992	0	hypothetical protein, conserved
PVX_116967	0	0	0	0	210.414	0	0	0	0	457.943	260.412	0	autophagy-related protein 12, putative (ATG12)
PVX_116970	0	45.2575	0	0	0	0	0	0	0	22.7717	12.9015	0	hypothetical protein, conserved
PVX_116975	0	400.746	0	0	182.582	0	0	0	217.182	100.421	113.948	0	hypothetical protein, conserved
PVX_116980	0	0	0	268.642	0	0	0	0	127.292	29.4994	66.8605	0	hypothetical protein, conserved
PVX_116985	0	0	0	0	0	0	0	0	0	3.50125	1.98297	0	biotin carboxylase subunit of acetyl CoA carboxylase, putative (ACC)

Gene ID / Patient	Patient 1	Patient 2	Patient 3	Patient 4	Patient 5	Patient 6	Patient 7	Patient 8	Patient 9	Patient 10	Patient 11	Patient 12	Gene Description
PVX_116990	0	0	0	0	0	0	0	0	19.0841	17.6977	0	0	hypothetical protein, conserved
PVX_116995	0	0	0	0	0	0	0	0	0	0	0	0	hypothetical protein, conserved
PVX_117000	89.6388	0	111.432	0	0	0	0	0	118.865	78.7411	80.2902	72.5572	hypothetical protein, conserved
PVX_117005	0	0	0	0	0	0	0	0	0	0	18.7175	0	shewanella-like protein phosphatase 1, putative (SHLP1)
PVX_117010	92.3796	64.5278	0	0	0	0	141.453	0	0	32.4518	36.778	0	hypothetical protein, conserved
PVX_117015	0	385.885	0	0	0	1419.67	0	0	0	96.7152	164.604	0	translation initiation factor EF-1, putative
PVX_117020	77.1576	26.9289	0	0	12.2335	0	0	107.141	73.0787	40.6677	15.3578	0	hypothetical protein, conserved
PVX_117025	29.9434	188.09	74.4153	0	18.9863	0	0	41.5758	79.4045	73.6541	53.64	0	splicing factor U2AF large subunit, putative (U2AF2)
PVX_117030	386.092	449.318	0	205.751	102.091	329.41	0	178.754	560.811	519.985	307.409	104.23	RNA helicase-1, putative
PVX_117035	0	0	0	0	0	0	0	0	0	0	0	0	hypothetical protein, conserved
PVX_117040	0	0	0	0	0	0	0	0	77.3628	0	0	0	derlin-1, putative (DER1-1)
PVX_117045	0	0	0	0	0	0	0	0	0	0	0	0	D13 protein, putative
PVX_117050	0	93.6648	0	0	0	0	0	0	0	0	0	0	hypothetical protein, conserved
PVX_117055	0	131.834	0	0	0	0	0	0	71.489	0	37.528	0	hypothetical protein, conserved
PVX_117060	59.6971	48.5971	0	0	3.15282	0	30.3897	13.812	45.2241	45.4556	33.6655	16.0913	hypothetical protein, conserved
PVX_117062	0	11.3614	40.4464	0	15.48	83.2255	0	0	18.5017	28.6077	12.9623	0	conserved Plasmodium protein, unknown function
PVX_117065	74.5195	0	0	0	17.7142	0	0	0	28.2282	13.0937	33.3727	0	TLD domain-containing protein
PVX_117070	0	0	0	0	0	0	0	0	0	0	0	0	hypothetical protein
PVX_117075	0	0	0	1132.36	0	0	542.375	0	133.374	123.243	0	0	hypothetical protein, conserved
PVX_117080	0	0	0	0	0	0	0	0	0	0	0	0	hypothetical protein, conserved
PVX_117085	0	0	0	0	0	0	100.062	0	0	0	13.0319	0	hypothetical protein
PVX_117090	81.4497	56.8852	0	0	25.8552	417.166	124.673	0	0	0	81.0652	131.993	DNA/RNA-binding protein KIN17, putative
PVX_117095	122.132	0	304.312	0	0	0	187.188	169.732	46.297	128.691	121.562	0	50S ribosomal protein L22, apicoplast, putative (L22)
PVX_117100	0	0	160.885	0	0	0	0	89.8296	73.5195	0	25.747	0	1-deoxy-D-xylulose 5-phosphate reductoisomerase, putative (DXR)
PVX_117105	58.2352	0	0	0	0	0	89.0734	161.762	22.0657	102.304	139.101	0	hypothetical protein, conserved
PVX_117110	0	0	0	0	20.6863	0	0	0	0	45.8088	51.9067	0	DNA-3-methyladenine glycosylase, putative
PVX_117115	0	0	0	0	0	0	62.8791	0	0	0	24.5615	0	hypothetical protein, conserved
PVX_117120	0	0	0	0	0	0	0	0	20.4492	18.9689	16.1166	0	rhoptry protein, putative
PVX_117125	0	0	0	0	0	0	49.5953	45.0595	12.2939	11.4032	25.837	0	hypothetical protein, conserved
PVX_117130	0	0	0	0	0	0	0	0	99.946	0	0	0	hypothetical protein, conserved
PVX_117135	0	0	0	0	0	0	0	0	0	0	0	0	hypothetical protein, conserved
PVX_117140	195.504	0	0	0	0	0	0	0	74.1475	0	0	0	hypothetical protein
PVX_117145	25.7999	36.0119	0	0	16.3573	0	0	0	19.5471	27.1986	25.6762	0	transcription factor with AP2 domain(s), putative (ApiAP2)
PVX_117150	99.4992	17.3601	0	0	23.6557	0	0	0	103.653	96.1527	138.631	40.2467	26S proteasome regulatory subunit RPN2, putative (RPN2)
PVX_117155	0	0	0	0	4.35208	0	0	0	0	4.82607	0	0	hypothetical protein
PVX_117160	0	0	0	0	0	0	56.2055	0	27.8619	12.921	29.2768	0	protein phosphatase containing kelch-like domains, putative (PPKL)
PVX_117165	0	0	0	0	0	0	157.243	0	0	0	0	0	hypothetical protein, conserved
PVX_117170	712.378	1244.43	887.416	0	301.79	1826.36	727.812	824.989	1260.17	1084.27	1063.58	770.438	40S ribosomal protein S3, putative
PVX_117175	0	0	0	0	0	0	0	0	0	0	0	0	dynein beta chain, putative
PVX_117180	0	0	0	0	0	0	0	0	0	0	0	0	aspartyl proteinase, putative
PVX_117185	0	0	0	0	0	0	0	0	0	0	27.0367	110.015	hypothetical protein, conserved
PVX_117190	0	0	0	68.9227	0	0	0	0	0	0	4.2956	0	potassium channel, putative
PVX_117192	0	0	0	0	0	0	1891.45	0	0	1252.15	1428.47	0	conserved Plasmodium protein, unknown function
PVX_117195	35.3344	24.6632	0	0	11.2038	0	0	0	13.3863	24.8321	7.03309	0	hypothetical protein, conserved
PVX_117200	0	70.1068	0	0	31.8716	0	0	0	76.0731	70.5049	0	0	hypothetical protein, conserved
PVX_117205	0	0	96.5241	0	49.2527	0	0	0	44.1327	40.9323	38.6444	62.8503	hypothetical protein, conserved
PVX_117210	0	0	357.13	0	45.5403	0	439.389	199.115	108.616	251.536	513.29	0	hypothetical protein, conserved
PVX_117215	46.9595	98.3472	0	0	0	0	0	0	71.1674	33.0001	56.0829	0	ATP-dependent zinc metalloprotease FTSH, putative
PVX_117220	0	0	0	0	0	0	0	0	0	199.393	0	0	hypothetical protein
PVX_117225	283.879	0	235.593	302.819	30.0476	0	0	0	0	0	37.6725	0	vacuolar ATP synthase subunit d, putative,ATP synthase (C/AC39) subunit, putative
PVX_117230	18.3322	38.379	0	0	0	93.7159	0	0	6.94428	25.7691	10.9465	0	Ser/Thr protein phosphatase family protein
PVX_117240	0	21.6431	0	0	0	0	0	0	0	3.63352	12.3473	16.7217	hypothetical protein, conserved
PVX_117245	436.98	0	0	0	0	0	0	0	332.012	153.241	174.049	0	hypothetical protein, conserved
PVX_117250	0	26.7558	0	0	0	0	0	53.2262	0	26.9376	15.2591	0	hypothetical protein, conserved
PVX_117255	0	0	0	0	0	0	60.2452	0	0	0	7.84461	0	hypothetical protein, conserved
PVX_117260	38.4793	0	0	0	0	0	0	0	14.578	13.5209	22.9773	0	hypothetical protein, conserved
PVX_117265	46.0415	32.1411	0	0	29.2052	0	70.3951	0	17.4439	32.3551	45.822	0	YL1 nuclear protein, putative
PVX_117270	0	21.6827	0	0	0	0	0	0	11.7689	0	0	0	hypothetical protein, conserved
PVX_117275	0	0	0	0	0	0	0	875.887	0	219.724	249.855	0	hypothetical protein, conserved
PVX_117280	0	0	0	220.694	21.9007	0	0	0	0	0	0	0	cyclin homologue, putative
PVX_117285	0	0	0	0	0	0	0	0	59.5562	0	0	0	hypothetical protein
PVX_117290	0	0	0	0	0	0	0	0	0	0	9.67198	0	hypothetical protein, conserved,Plasmodium yoelii subtelomeric family PYST-A containing protein

Gene ID / Patient	Patient 1	Patient 2	Patient 3	Patient 4	Patient 5	Patient 6	Patient 7	Patient 8	Patient 9	Patient 10	Patient 11	Patient 12	Gene Description
PVX_117292	0	0	0	0	15.2629	0	0	0	54.6962	0	57.4701	0	DNA-directed RNA polymerase III subunit RPC4, putative
PVX_117295	0	43.3713	0	0	0	0	0	86.2736	23.5366	43.6474	12.3642	0	DNA polymerase alpha subunit, putative
PVX_117300	166.537	0	0	266.388	0	0	127.464	115.677	63.1126	58.505	82.8757	0	replication factor C3, putative
PVX_117310	0	0	13.1701	0	1.68026	0	0	0	0	0	1.0555	0	hypothetical protein
PVX_117315	0	72.215	257.427	0	0	0	0	0	0	0	20.577	0	hypothetical protein, conserved
PVX_117320	44.1778	0	0	0	0	0	67.5416	0	16.7376	0	35.1737	0	hypothetical protein, conserved
PVX_117322	9272.13	4858.61	5223.94	6361.18	3050.6	1131.69	3720.55	7822.64	8451.58	8142.21	7404.95	5728.83	glyceraldehyde-3-phosphate dehydrogenase, putative (GAPDH)
PVX_117325	0	37.4639	133.447	0	17.0224	0	0	0	20.3318	0	53.4057	0	cytochrome c1 precursor, putative
PVX_117330	0	0	0	0	0	0	0	0	0	0	38.8734	0	hypothetical protein, conserved
PVX_117335	0	0	0	0	0	0	0	0	0	0	0	0	hypothetical protein, conserved
PVX_117340	6.73199	0	0	0	2.13307	0	0	0	0	7.09763	1.33991	0	hypothetical protein, conserved
PVX_117345	0	0	0	0	0	0	0	59.739	24.4493	30.2413	12.8465	0	hypothetical protein, conserved
PVX_117350	0	0	0	0	0	0	0	0	0	0	877.137	0	hypothetical protein, conserved
PVX_117356	22.3691	0	0	0	0	0	0	0	16.9474	31.443	0	0	hypothetical protein, conserved
PVX_117357	0	0	0	0	176.108	0	0	0	0	0	0	0	thioredoxin-like protein (TLP1)
PVX_117360	0	17.2546	0	78.9642	7.83729	0	37.7755	34.3265	65.5603	78.1923	49.2101	0	valine--tRNA ligase, putative
PVX_117365	14.5005	0	0	0	0	0	0	0	0	0	2.88615	0	hypothetical protein
PVX_117370	0	0	0	0	0	0	0	0	0	0	0	0	hypothetical protein
PVX_117375	43.9608	92.0633	0	0	0	0	0	0	49.966	61.7864	43.7511	0	splicing factor 3B subunit 2, putative
PVX_117380	0	0	0	0	0	0	0	0	0	72.6038	0	0	meiotic nuclear division protein 1, putative (MND1)
PVX_117385	58.8093	0	0	0	0	0	0	0	0	0	23.412	0	phosphatidylinositol-4-phosphate 5-kinase, putative
PVX_117390	932.694	260.914	465.636	598.503	0	958.43	286.491	259.404	990.401	851.47	631.326	0	40S ribosomal protein S28e, putative
PVX_117395	0	64.1961	0	588.178	0	470.865	0	127.686	0	129.141	146.356	148.981	U3 small nucleolar ribonucleoprotein protein IMP3, putative (IMP3)
PVX_117400	0	0	0	0	0	0	0	0	18.0476	0	0	0	hypothetical protein, conserved
PVX_117405	0	53.9368	192.193	0	24.5138	0	0	0	0	27.1327	15.3735	0	hypothetical protein, conserved
PVX_117410	77.2989	53.9834	0	247.249	24.5351	0	0	0	0	27.1562	61.5469	0	30S ribosomal protein S10, putative
PVX_117415	0	0	0	0	0	0	0	0	21.2362	0	44.6245	0	snRNA-activating protein complex subunit 3, putative
PVX_117420	377.706	197.878	470.19	302.178	149.92	0	722.981	787.155	393.664	464.387	300.744	153.079	60S ribosomal protein L27, putative (RPL27)
PVX_117425	0	0	0	0	0	0	0	0	0	0	0	0	inner membrane complex sub-compartment protein 3, putative (ISP3)
PVX_117430	0	0	0	0	0	0	0	0	0	0	0	0	hypothetical protein
PVX_117435	0	60.7602	0	0	55.2364	0	0	120.854	0	61.1198	69.2654	0	ubiquitin carboxyl-terminal hydrolase isozyme L3, putative (UCHL3)
PVX_117440	1082.28	947.152	2031	870.181	258.869	2787.42	0	1882.86	513.353	1424.45	754.211	881.639	60S ribosomal protein L29, putative
PVX_117445	136.058	0	339.144	0	0	0	0	189.112	0	47.7851	81.2556	0	hypothetical protein, conserved
PVX_117450	0	0	0	0	0	0	0	0	0	0	10.2864	0	kelch domain-containing protein
PVX_117455	0	173.532	0	0	0	0	0	0	47.0684	43.6109	0	0	FYVE and coiled-coil domain-containing protein, putative (FCP)
PVX_117460	0	0	0	0	0	0	0	0	0	0	0	0	hypothetical protein
PVX_117465	0	0	0	0	0	0	0	0	0	0	0	0	hypothetical protein, conserved
PVX_117470	205.082	286.54	0	328.221	32.5672	0	314.125	427.434	233.191	108.058	224.532	166.271	bax inhibitor 1, putative
PVX_117475	243.932	0	0	0	0	0	0	0	46.2338	171.355	242.793	0	hypothetical protein, conserved
PVX_117480	54.7401	0	0	0	17.3535	0	41.8226	0	72.5793	96.1788	125.299	0	hypothetical protein, conserved
PVX_117485	0	0	0	0	0	0	0	0	12.201	45.2683	12.8209	0	hypothetical protein, conserved
PVX_117490	108.773	151.989	0	0	69.1032	0	166.641	151.146	123.687	38.2072	43.3052	0	hypothetical protein, conserved
PVX_117495	0	68.7936	0	0	0	0	0	0	37.3245	0	58.8098	0	OPA3-like protein, putative
PVX_117500	42.5398	0	0	135.935	0	0	0	0	80.5842	59.7897	76.2063	0	hypothetical protein, conserved
PVX_117505	0	28.5938	0	130.889	0	0	62.6197	113.764	15.5191	14.3934	24.4603	0	GTP-binding protein, putative
PVX_117510	128.918	112.476	80.1019	0	40.874	0	49.2556	0	97.678	113.252	141.131	104.314	ATP-dependent RNA helicase DBP5, putative (DBP5)
PVX_117515	0	0	0	0	0	0	0	0	0	64.9566	0	0	hypothetical protein, conserved
PVX_117525	33.446	23.3446	0	0	0	0	0	0	12.6707	23.5053	33.286	0	hypothetical protein, conserved
PVX_117530	0	0	185.147	0	70.8463	0	0	0	28.1963	130.703	59.244	0	exonuclease V, mitochondrial, putative
PVX_117535	0	50.1157	0	76.4491	22.7631	0	0	33.2337	0	25.2346	38.115	0	hypothetical protein, conserved
PVX_117540	0	0	53.9735	0	0	0	0	0	0	0	17.295	0	spindle assembly abnormal protein 4, putative (SAS4)
PVX_117545	0	0	0	0	148.523	0	0	0	0	0	23.2646	0	aminodeoxychorismate lyase, putative (ADCL)
PVX_117550	0	14.6684	0	67.1248	26.6492	0	32.1113	0	47.7729	44.3179	25.1016	0	hypothetical protein, conserved
PVX_117555	667.651	0	0	0	0	0	0	0	508.062	233.868	133.002	0	hypothetical protein, conserved
PVX_117560	0	0	0	0	0	0	8223.06	0	0	1659.9	0	0	hypothetical protein, conserved
PVX_117565	15.9888	33.4856	39.7554	0	0	0	0	22.204	72.6936	33.7074	14.3213	38.8293	cysteine proteinase precursor,vivapain-1
PVX_117570	0	0	0	0	2.6776	0	12.9044	0	0	0	5.04567	0	hypothetical protein, conserved
PVX_117575	0	0	0	0	0	0	0	0	0	0	28.2591	0	hypothetical protein, conserved
PVX_117580	17.7364	37.1313	44.0633	0	5.6214	0	0	24.6237	47.0298	24.9316	38.8326	0	hypothetical protein, conserved
PVX_117585	98.4116	0	122.353	0	15.6074	0	0	68.3343	18.6432	69.1564	39.1772	0	hypothetical protein, conserved
PVX_117590	0	0	0	0	0	0	0	0	0	28.1528	0	0	vacuolar protein sorting-associated protein 4, putative (VPS4)
PVX_117595	19.5406	0	0	0	0	0	14.921	13.5632	11.1024	0	15.5572	0	hypothetical protein, conserved

Gene ID / Patient	Patient 1	Patient 2	Patient 3	Patient 4	Patient 5	Patient 6	Patient 7	Patient 8	Patient 9	Patient 10	Patient 11	Patient 12	Gene Description
PVX_117600	158.887	73.9516	0	0	100.803	0	80.9946	0	60.2009	148.869	52.7103	0	hypothetical protein, conserved
PVX_117605	0	518.001	0	792.885	0	0	0	686.546	561.59	606.176	343.829	1204.99	thioredoxin 1, putative (TRX1)
PVX_117610	0	28.0164	0	0	0	0	0	0	0	0	7.98887	0	hypothetical protein, conserved
PVX_117615	0	0	341.553	0	21.7829	0	105.027	0	208.088	144.697	68.3175	111.199	signal peptide peptidase, putative (SPP)
PVX_117620	0	0	0	0	0	0	295.794	0	73.0292	67.6096	0	0	conserved protein, unknown function
PVX_117625	0	201.727	0	131.917	26.1843	0	0	57.3283	93.8452	72.5311	8.21737	0	V-type H(+)-translocating pyrophosphatase, putative (VP1)
PVX_117630	0	0	230.611	0	0	0	141.813	128.804	0	16.2949	110.771	0	hypothetical protein, conserved
PVX_117635	139.234	0	0	0	0	0	213.515	0	158.357	97.7989	166.304	0	hypothetical protein, conserved
PVX_117640	0	0	31.7313	0	0	0	0	35.4677	0	4.48923	35.5959	0	hypothetical protein, conserved
PVX_117645	0	0	0	0	10.9952	0	0	0	0	0	0	0	hypothetical protein, conserved
PVX_117650	77.8368	0	0	497.945	24.7061	0	0	108.126	0	27.3451	77.469	0	hypothetical protein, conserved
PVX_117655	194.149	0	0	0	36.9395	0	0	107.839	29.4218	54.5764	92.7466	125.701	hypothetical protein, conserved
PVX_117660	0	0	0	0	0	0	0	0	56.3458	0	0	0	serine hydroxymethyltransferase, putative
PVX_117665	0	0	0	0	0	0	0	0	0	0	0	0	transcription factor with AP2 domain(s), putative (ApiAP2)
PVX_117670	79.9936	0	199.08	0	0	0	0	0	0	0	47.7695	0	polyprenol reductase, putative
PVX_117675	0	11.6955	0	0	0	0	0	0	6.34859	0	0	0	LCCL domain-containing protein (CCp2)
PVX_117680	0	0	0	0	0	0	0	0	0	0	16.5972	44.9754	hypothetical protein, conserved
PVX_117685	0	0	0	0	0	0	0	0	0	0	0	0	ferlin, putative
PVX_117690	26.37	55.212	131.056	0	16.7191	0	0	0	19.9791	27.7995	78.7309	0	AP-1 complex subunit gamma, putative
PVX_117695	89.9651	0	0	0	28.5642	0	137.745	124.989	102.287	94.8118	107.45	145.828	hemolysin III, putative (HlyIII)
PVX_117700	0	0	0	0	0	0	0	0	0	0	0	0	Pv-fam-g protein
PVX_117705	0	0	0	0	0	0	0	0	0	40.8895	0	0	methyltransferase, putative
PVX_117710	0	0	0	0	0	0	0	0	0	0	0	0	hypothetical protein
PVX_117715	0	187.722	0	0	0	0	0	0	0	0	106.776	0	protein tyrosine phosphatase, putative (PTP1)
PVX_117720	0	0	0	0	0	0	0	0	0	0	0	0	hypothetical protein
PVX_117725	0	0	0	0	0	0	0	0	0	0	10.8305	0	hypothetical protein
PVX_117730	0	0	0	0	0	0	0	0	0	0	0	0	protein phosphatase 2C, putative
PVX_117735	0	0	0	0	0	0	0	0	0	0	0	0	Pv-fam-g protein
PVX_117740	0	0	0	0	0	0	0	0	0	0	0	0	Pv-fam-g protein
PVX_117745	238.211	83.161	148.124	380.781	151.153	0	546.555	578.984	248.216	564.937	474.16	96.4488	6-phosphogluconate dehydrogenase, decarboxylating, putative
PVX_117750	0	0	0	239.575	47.5477	0	114.632	104.05	141.926	131.578	104.371	121.365	ribosomal protein S11, putative
PVX_117755	147.252	102.929	0	0	0	0	0	0	0	0	29.3139	0	nifU protein, putative
PVX_117760	131.475	229.414	0	105.003	31.264	0	0	45.6389	161.875	92.3983	157.015	53.1926	peptidase, putative
PVX_117765	0	0	0	0	0	0	0	0	0	0	0	0	serine/threonine protein kinase, putative
PVX_117770	19.2925	53.8533	0	61.6086	12.2297	0	29.4722	0	29.2324	47.458	26.8799	0	hypothetical protein, conserved
PVX_117775	0	0	0	0	0	0	0	399.587	0	201.485	114.313	0	tRNA intron endonuclease, putative
PVX_117780	53.4367	37.3072	0	0	16.9511	0	81.7212	0	40.4936	18.7753	31.9095	0	RNA-binding protein, putative
PVX_117785	0	24.959	88.8802	0	0	0	0	0	0	0	14.2348	0	hypothetical protein, conserved
PVX_117790	104.032	48.4086	86.1913	0	21.9904	0	53.0005	48.1509	52.549	134.037	103.534	56.1222	glucose-6-phosphate 1-dehydrogenase, putative
PVX_117795	1277.49	446.162	1362.99	875.954	260.756	467.495	978.018	887.42	899.15	1121.92	1180.66	295.829	hypothetical protein, conserved
PVX_117800	0	13.4459	0	0	0	0	0	0	0	13.5419	7.67004	0	hypothetical protein, conserved
PVX_117805	0	0	0	0	0	0	0	0	0	0	0	0	pyridine nucleotide transhydrogenase, putative
PVX_117810	0	23.532	0	0	10.6876	0	0	0	31.9343	17.7758	16.7799	0	hypothetical protein, conserved
PVX_117812	0	0	0	0	0	0	0	0	0	0	0	0	conserved Plasmodium protein, unknown function
PVX_117815	0	0	0	0	0	0	0	0	0	0	3.85912	0	hypothetical protein, conserved
PVX_117825	0	0	0	0	0	0	0	0	0	0	9.84301	0	dynactin subunit 4, putative
PVX_117830	63.7019	0	0	0	10.0984	162.891	48.6765	44.2253	24.1326	44.7689	0	0	hypothetical protein, conserved
PVX_117835	0	220.391	0	0	0	0	0	0	0	0	31.3796	0	hypothetical protein, conserved
PVX_117840	0	0	458.905	0	0	0	0	0	69.7243	64.5567	73.2053	0	hypothetical protein, conserved
PVX_117845	0	0	295.805	0	37.7237	0	181.953	0	45.0061	0	23.635	192.609	hypothetical protein, conserved
PVX_117850	0	0	0	31.7855	0	0	0	13.8212	18.856	6.99783	3.96329	16.102	hypothetical protein, conserved
PVX_117855	410.749	0	0	0	65.3922	0	0	285.65	77.8962	216.313	40.8877	0	hypothetical protein, conserved
PVX_117860	34.7176	24.2325	0	110.915	11.008	0	0	48.207	39.4577	12.1994	27.6412	0	hypothetical protein, conserved
PVX_117865	0	0	0	0	32.8692	0	0	0	0	0	0	0	hypothetical protein, conserved
PVX_117870	0	0	0	198.875	0	0	0	86.3936	47.1386	0	12.3813	0	aminomethyl transferase domain containing protein
PVX_117875	0	86.6823	0	396.92	0	0	0	86.2137	0	0	12.3556	0	hypothetical protein, conserved
PVX_117880	0	9.74429	0	0	0	0	0	0	0	4.90739	0	0	rhoptry neck protein 2, putative (RON2)
PVX_117885	0	18.3391	0	83.9295	8.33006	0	0	0	49.7718	18.4677	10.4605	0	hypothetical protein, conserved
PVX_117890	32.6409	0	0	0	0	0	0	0	24.7313	11.4698	32.4847	0	sortilin, putative
PVX_117895	0	0	0	0	0	0	0	0	0	0	0	0	protein phosphatase 2b regulatory subunit, putative (CNB)
PVX_117900	0	0	0	0	0	0	0	0	0	0	0	0	LCCL domain-containing protein (LAP5)
PVX_117905	0	229.716	0	0	0	0	0	0	0	57.689	32.7047	0	pre-mRNA-splicing factor CWF18, putative

Gene ID / Patient	Patient 1	Patient 2	Patient 3	Patient 4	Patient 5	Patient 6	Patient 7	Patient 8	Patient 9	Patient 10	Patient 11	Patient 12	Gene Description
PVX_117910	0	34.9242	0	0	7.93156	0	0	0	9.47837	8.79247	14.9405	40.4832	hypothetical protein, conserved
PVX_117915	180.519	0	0	579.089	57.4429	0	0	251.018	0	190.157	143.752	0	hypothetical protein, conserved
PVX_117920	18.3887	51.3297	0	58.7209	5.82825	188.01	0	0	27.8627	25.8485	29.2806	29.747	hypothetical protein, conserved
PVX_117925	1013.53	544.211	387.612	498.216	111.253	199.403	178.765	541.308	649.818	643.786	426.755	315.485	elongation factor 2, putative
PVX_117930	41.5909	0	0	0	13.1896	0	0	0	0	0	107.62	67.3245	hypothetical protein, conserved
PVX_117935	0	0	0	0	0	0	0	0	0	0	0	0	acetyl-CoA acetyltransferase, putative
PVX_117940	0	55.3717	0	0	0	0	242.702	0	90.1375	0	94.6925	0	hypothetical protein, conserved
PVX_117945	0	42.9625	76.49	98.3162	0	0	0	0	11.6596	10.8151	55.1343	0	hypothetical protein, conserved
PVX_117950	52.2816	36.5002	130.012	167.11	49.7528	0	0	0	19.809	36.7391	72.846	0	hypothetical protein, conserved
PVX_117960	18.6105	0	0	0	5.89858	0	0	0	14.0994	0	3.70423	0	hypothetical protein, conserved
PVX_117965	0	0	0	53.6879	10.6575	85.9475	25.6829	23.3421	31.8443	29.5428	16.7326	27.1974	hypothetical protein, conserved
PVX_117970	40.5727	28.3214	0	0	12.8664	0	0	0	30.7427	42.7691	8.07579	0	flavodoxin domain containing protein
PVX_117975	0	0	0	0	0	0	0	0	0	0	28.2448	0	hypothetical protein
PVX_117980	0	102.541	0	0	0	187.852	0	0	41.7414	25.8103	36.5509	118.884	signal recognition particle subunit SRP54, putative (SRP54)
PVX_117985	0	0	0	0	0	0	0	0	0	33.776	38.2797	0	protein kinase, putative
PVX_117990	0	0	0	0	0	0	0	0	22.5052	41.7361	35.4676	0	hypothetical protein, conserved
PVX_117995	0	0	0	0	0	441.751	66.0108	0	32.6821	30.295	60.0816	0	hypothetical protein, conserved
PVX_118000	0	0	0	0	8.14811	0	0	0	9.73703	18.0647	25.5803	0	exosome complex exonuclease RRP6, putative (RRP6)
PVX_118005	0	0	0	0	0	0	0	0	0	0	0	0	hypothetical protein, conserved
PVX_118010	0	0	0	0	0	0	0	0	0	0	0	0	Histone H1, gonadal, putative
PVX_118015	0	0	0	0	0	0	0	0	0	0	7.92489	0	transcription factor with AP2 domain(s), putative (ApiAP2)
PVX_118020	0	0	0	0	0	0	34.9179	31.7311	0	8.03136	4.54901	0	crossover junction endonuclease MUS81, putative (MUS81)
PVX_118025	0	29.5268	105.157	135.163	13.4142	0	64.6646	176.214	48.0762	89.176	33.6774	0	transcription factor IIIb subunit, putative
PVX_118030	0	0	0	0	0	0	0	0	0	573.21	0	0	hypothetical protein, conserved
PVX_118035	0	0	0	0	0	0	0	0	0	0	15.641	0	hypothetical protein, conserved
PVX_118040	0	0	0	0	0	0	0	0	0	0	0	0	gamete egress and sporozoite traversal protein, putative (GEST)
PVX_118045	0	0	0	0	0	0	0	0	0	0	58.2787	0	hypothetical protein
PVX_118050	0	100.123	0	0	0	0	0	0	0	50.3073	28.5161	0	hypothetical protein, conserved
PVX_118055	152.231	53.1561	0	0	0	0	0	105.733	86.5325	53.4812	60.6046	0	hypothetical protein, conserved
PVX_118060	0	133.526	0	0	0	0	0	0	0	67.0332	38.0085	0	hypothetical protein, conserved
PVX_118062	27.8774	0	46.1605	29.6661	5.88912	0	14.1911	0	14.0791	32.6568	29.5927	15.0284	chloroquine resistance marker protein, putative
PVX_118065	35.9014	25.0592	0	0	22.7674	0	0	49.8514	54.4046	50.4611	21.4379	0	ubiquitin-protein ligase, putative
PVX_118070	0	14.2662	0	0	6.4796	0	0	0	0	7.18387	4.06893	0	hypothetical protein, conserved
PVX_118075	0	0	0	0	0	0	0	0	0	0	0	0	hypothetical protein
PVX_118080	53.2579	0	0	0	0	0	40.6892	0	30.263	37.43	15.9008	0	hypothetical protein, conserved
PVX_118090	0	0	0	0	0	0	0	166.323	0	0	71.4741	0	hypothetical protein, conserved
PVX_118095	66.2954	77.1099	109.814	0	0	0	0	92.0429	0	31.0626	21.9924	0	WD domain, G-beta repeat domain containing protein
PVX_118100	19.3929	0	0	0	0	0	0	26.9239	22.0383	13.6299	3.85995	0	multidrug resistance protein 2, putative
PVX_118105	0	0	0	0	5.67769	0	0	0	6.78579	6.29527	17.8278	0	calponin homology domain-containing protein, putative
PVX_118110	0	151.989	0	348.228	0	0	0	0	82.4577	38.2072	216.526	0	hypothetical protein, conserved
PVX_118115	0	0	0	0	0	0	0	0	0	0	0	0	hypothetical protein
PVX_118120	0	0	0	0	0	0	0	0	0	0	0	0	hypothetical protein, conserved
PVX_118125	0	0	0	0	0	0	0	152.631	0	77.1644	65.5957	0	hypothetical protein
PVX_118130	0	0	0	0	0	0	0	0	0	97.9196	166.657	0	mitochondrial ribosomal protein S14 precursor, putative
PVX_118135	48.5123	67.7341	0	155.046	30.7744	0	74.1787	67.3708	36.7607	34.091	57.9375	78.5438	hypothetical protein, conserved
PVX_118140	0	342.057	0	0	0	0	250.28	226.735	0	114.538	32.4663	0	hypothetical protein, conserved
PVX_118145	1094.1	903.274	743.015	1591.72	221.113	1019.41	761.673	1381.98	1394.83	628.905	752.376	322.536	40S ribosomal protein S5, putative
PVX_118150	0	0	0	0	32.1973	0	0	0	0	0	20.1807	0	hypothetical protein, conserved
PVX_118155	214.223	0	0	0	68.2246	0	0	0	81.2575	75.2079	127.95	0	heme detoxification protein, putative (HDP)
PVX_118160	0	0	0	0	0	0	0	0	39.3616	0	62.0177	0	hypothetical protein, conserved
PVX_118162	0	0	0	0	0	0	0	0	0	103.392	58.6626	0	centrin-2, putative (CEN2)
PVX_118165	20.4329	7.12871	25.3758	0	0	0	15.6026	0	34.8281	17.9519	20.3345	16.5231	hypothetical protein, conserved
PVX_118170	0	0	0	0	0	0	0	0	0	0	17.5035	0	pyruvate dehydrogenase E1 component subunit beta, putative
PVX_118175	0	0	0	0	7.95381	0	0	0	0	0	0	0	hypothetical protein, conserved
PVX_118180	502.578	287.054	0	584.043	72.4532	233.757	349.276	253.792	432.757	385.288	354.674	443.8	M17 leucyl aminopeptidase, putative
PVX_118185	0	0	0	0	0	0	0	0	0	0	0	0	hypothetical protein, conserved
PVX_118190	0	135.478	0	0	41.0431	331.08	296.827	269.489	196.052	181.779	141.609	0	ATP-dependent RNA helicase DDX5, putative (DDX5)
PVX_118195	0	0	0	0	0	0	0	0	0	0	0	0	hypothetical protein, conserved
PVX_118200	200.844	140.245	0	214.078	84.9771	0	0	185.98	101.474	117.605	93.2832	0	hypothetical protein, conserved
PVX_118205	0	28.9518	0	0	0	0	0	0	31.4268	29.1469	24.7664	0	hypothetical protein, conserved
PVX_118210	0	0	0	0	0	0	0	0	0	124.216	70.5047	0	hypothetical protein, conserved
PVX_118215	0	0	0	0	0	0	0	455.231	0	0	0	0	hypothetical protein

Gene ID / Patient	Patient 1	Patient 2	Patient 3	Patient 4	Patient 5	Patient 6	Patient 7	Patient 8	Patient 9	Patient 10	Patient 11	Patient 12	Gene Description
PVX_118220	0	25.4473	0	0	11.5601	0	0	50.6233	55.247	76.8631	43.5395	0	serine/threonine kinase-1, putative
PVX_118225	55.5254	0	138.091	0	35.2292	0	0	77.1154	42.077	19.509	77.366	0	hypothetical protein, conserved
PVX_118230	33.6852	0	0	0	0	0	0	46.773	38.2841	5.91833	10.0572	0	RNA helicase, putative
PVX_118235	60.3715	63.2043	75.0182	192.849	38.2802	0	0	83.825	68.6121	106.072	114.157	0	histidine--tRNA ligase, putative
PVX_118240	0	61.0573	0	279.691	0	0	0	121.445	0	30.7091	121.807	0	hypothetical protein, conserved
PVX_118245	0	0	0	0	0	0	0	0	33.689	62.4548	70.7792	144.085	hypothetical protein, conserved
PVX_118255	986.961	1999.42	491.509	0	501.489	0	604.62	1234.17	1720.72	1490.79	864.446	320.039	fructose 1,6-bisphosphate aldolase, putative
PVX_118260	0	0	0	0	0	0	366.389	0	0	0	47.4186	0	hypothetical protein, conserved
PVX_118265	0	0	0	0	0	0	0	0	0	0	0	0	hypothetical protein
PVX_118270	0	0	0	0	0	0	0	0	0	0	4.06893	0	serine/threonine protein kinase, putative
PVX_118275	0	48.6423	0	0	0	0	0	0	26.3958	24.4727	41.5968	0	hypothetical protein, conserved
PVX_118280	0	185.108	0	0	42.0922	0	0	184.068	150.617	46.5131	52.7272	0	1-acyl-sn-glycerol-3-phosphate acyltransferase, putative (LPAAT)
PVX_118285	0	0	0	0	0	0	0	0	0	74.6722	0	0	hypothetical protein, conserved
PVX_118290	0	0	0	0	0	0	0	0	0	0	2.63254	0	hypothetical protein, conserved
PVX_118292	0	0	0	0	0	0	0	0	0	0	0	0	conserved Plasmodium protein, unknown function
PVX_118295	0	40.6547	72.3793	0	9.23344	0	0	0	44.1334	10.2344	34.7824	47.1288	heat shock protein, putative
PVX_118300	49.3959	0	0	0	15.6678	252.751	0	0	37.4305	17.3559	29.4964	0	hypothetical protein, conserved
PVX_118305	143.936	0	0	0	45.7608	0	0	0	54.5704	50.5498	0	0	dephospho-CoA kinase, putative (DPCK)
PVX_118310	0	0	0	53.0596	0	0	0	0	0	0	0	0	gamma-tubulin complex component, putative
PVX_118315	0	23.699	84.391	0	10.7656	0	0	94.2917	64.3153	47.7238	20.2747	0	poly(A)-specific ribonuclease PARN, putative
PVX_118320	72.4421	101.13	0	0	11.4852	0	0	0	68.6114	76.3655	93.7246	58.6234	WD domain, G-beta repeat domain containing protein
PVX_118325	0	0	0	0	0	0	0	367.656	100.242	92.7235	210.398	0	U6 snRNA-associated Sm-like protein LSm5, putative
PVX_118330	56.3647	0	0	0	0	0	0	0	0	19.8038	22.4387	0	hypothetical protein, conserved
PVX_118335	80.4523	0	0	0	0	0	0	0	0	0	32.0273	0	hypothetical protein, conserved
PVX_118340	0	0	57.8516	74.3594	0	0	0	0	0	8.18178	9.26845	0	serine/threonine protein kinase, putative (SRPK2)
PVX_118345	18.9069	6.59626	0	0	5.99117	0	0	26.2466	14.323	13.289	11.2895	15.2888	protein transport protein SEC7, putative (SEC7)
PVX_118350	0	0	0	0	0	0	0	86.3335	0	65.5166	61.8637	0	hypothetical protein, conserved
PVX_118355	0	0	0	0	0	0	0	0	0	0	4.97645	0	hypothetical protein
PVX_118360	11.5613	0	0	0	3.66372	0	0	32.0996	4.37925	0	4.60228	0	TRAP-like protein (TREP)
PVX_118365	0	26.9755	0	0	0	0	0	0	43.923	27.1586	7.69217	0	protein prenyltransferase alpha subunit, putative
PVX_118370	0	0	0	0	7.1881	0	0	31.4894	4.29599	3.98576	15.8018	0	hypothetical protein, conserved
PVX_118375	0	81.0558	0	0	92.0775	297.091	177.568	241.856	87.9763	183.55	127.095	0	methionine-tRNA ligase, putative
PVX_118380	32.3811	0	0	0	0	0	0	0	0	0	25.7809	0	GTP-binding protein, putative
PVX_118385	194.653	0	0	0	61.9623	0	0	270.71	73.8245	136.688	232.516	0	replication factor A protein 3, putative (RPA3)
PVX_118390	86.4874	60.4075	0	0	0	0	0	0	65.5538	91.1483	86.0797	140.177	ADP-ribosylation-like factor, putative
PVX_118395	0	0	0	0	0	0	0	0	0	0	1166.19	0	REX1 DNA repair protein, putative
PVX_118400	120.488	0	0	0	38.2841	617.835	0	0	0	84.6399	47.9702	0	vacuolar-sorting protein SNF7, putative
PVX_118405	71.666	0	0	0	0	0	0	0	0	75.5338	14.2654	0	metalloprotease, putative
PVX_118410	56.047	39.1309	0	0	0	0	0	0	84.9448	19.6922	22.3122	0	hypothetical protein, conserved
PVX_118415	0	0	0	0	0	0	0	0	0	0	11.0045	0	hypothetical protein, conserved
PVX_118420	132.142	30.7482	0	0	41.9083	0	67.3419	122.335	16.6881	61.908	96.4419	0	FACT complex subunit SSRP1, putative (FACT-S)
PVX_118425	0	0	0	0	0	0	0	0	0	0	0	0	serine/threonine protein kinase, putative
PVX_118430	624.109	373.651	0	0	56.6151	0	546.025	247.734	540.637	344.528	496.936	144.517	60S ribosomal protein L10a, putative
PVX_118435	321.411	617.301	0	257.038	127.531	823.068	245.979	446.494	365.409	649.257	703.765	130.211	KS1 protein precursor, putative
PVX_118440	0	0	0	0	0	0	0	0	0	0	0	0	hypothetical protein, conserved
PVX_118445	0	0	0	0	0	0	0	0	0	0	0	0	hypothetical protein, conserved
PVX_118450	0	28.3343	0	129.701	38.6166	0	124.102	0	30.7566	42.7885	96.9535	0	transporter, putative
PVX_118455	42.8453	89.726	0	0	27.1756	0	0	59.4975	48.6978	15.0547	196.148	0	clathrin coat assembly protein AP50, putative
PVX_118460	0	0	0	0	0	0	0	0	0	0	0	0	hypothetical protein, conserved
PVX_118465	0	0	0	0	0	0	0	0	0	0	12.8735	0	allantoicase, putative
PVX_118470	26.7964	0	0	0	0	0	0	0	0	0	0	0	hypothetical protein, conserved
PVX_118475	0	0	101.165	0	0	0	0	0	0	14.3089	8.10453	32.9362	stromal-processing peptidase, putative (SPP)
PVX_118480	0	0	120.691	0	0	0	0	0	0	0	19.323	78.5864	delta-aminolevulinic acid dehydratase, putative,porphobilinogen synthase, putative (PBGS)
PVX_118485	0	19.4898	0	0	0	0	0	0	0	0	38.908	0	hypothetical protein, conserved
PVX_118490	0	0	0	0	0	0	0	0	5.82091	0	12.2345	0	hypothetical protein, conserved
PVX_118495	932.485	723.824	1032.1	0	329.074	531.016	476.114	719.82	1217.38	1128.23	494.992	0	triosephosphate isomerase, putative
PVX_118500	173.183	0	0	0	0	0	0	0	131.343	60.8121	34.4772	0	vesicle-associated membrane protein, putative
PVX_118505	0	0	255.063	0	32.5298	0	156.882	0	77.6413	0	20.3886	0	leucine carboxyl methyltransferase, putative
PVX_118510	0	66.6636	0	0	0	0	0	265.186	36.1695	33.524	170.973	0	hypothetical protein, conserved
PVX_118515	0	0	63.5603	81.697	0	0	0	0	0	0	40.7296	0	histone, putative
PVX_118520	102.777	0	255.944	0	97.9265	0	0	0	155.818	144.409	184.13	0	ubiquinol cytochrome c oxidoreductase, putative
PVX_118525	0	12.7694	0	0	0	0	27.9528	0	20.7945	6.43041	21.8527	0	hypothetical protein, conserved

Gene ID / Patient	Patient 1	Patient 2	Patient 3	Patient 4	Patient 5	Patient 6	Patient 7	Patient 8	Patient 9	Patient 10	Patient 11	Patient 12	Gene Description
PVX_118530	0	0	0	0	0	0	0	70.3641	0	0	65.5672	0	hypothetical protein, conserved
PVX_118535	0	8.77473	31.2362	40.1494	11.9551	0	0	0	9.52651	17.6769	15.0174	0	RNA helicase, putative
PVX_118540	61.8579	0	153.867	0	19.6266	0	0	0	70.3167	0	24.6257	100.189	copper transporter, putative
PVX_118545	2325.97	651.058	2325.44	2241.75	815.206	0	1073.26	1294.4	1323.57	1877.97	1806.05	378.545	thioredoxin peroxidase 1, putative (TPx1)
PVX_118550	68.5575	23.926	0	0	0	0	0	47.5974	25.9725	24.0904	6.82296	0	hypothetical protein
PVX_118560	0	54.3594	0	0	0	0	0	108.126	29.4968	27.3451	61.9752	0	DNA primase small subunit, putative
PVX_118565	0	0	0	0	0	0	0	197.218	0	249.145	84.7344	0	conserved protein, unknown function
PVX_118570	0	20.0074	0	0	0	0	0	0	13.0312	0	2.28219	0	cleavage and polyadenylation specificity factor, putative
PVX_118575	31.969	0	39.7092	0	0	0	0	0	6.05487	0	6.36308	0	metacaspase 2, putative
PVX_118580	55.4222	116.083	137.834	0	52.7455	0	84.7631	76.972	146.996	175.254	121.349	448.743	translocation protein sec62, putative
PVX_118585	0	0	0	0	0	0	0	0	0	0	0	0	50S ribosomal protein L20, putative
PVX_118590	0	0	17.6056	0	0	0	0	39.3615	8.05504	9.96498	7.05458	11.4636	hypothetical protein, conserved
PVX_118595	69.489	0	0	111.001	22.0332	0	0	0	13.1628	24.4177	6.91568	0	DNA repair metallo-beta-lactamase protein, putative
PVX_118600	0	0	0	0	0	0	0	0	0	0	8.68401	0	hypothetical protein, conserved
PVX_118605	0	37.1739	0	0	0	0	0	0	40.3489	130.958	52.9926	0	hypothetical protein, conserved
PVX_118610	52.806	36.8666	0	168.789	16.7508	0	0	146.673	40.0155	111.323	31.5329	0	hypothetical protein, conserved
PVX_118615	43.9824	0	109.348	0	0	0	67.2424	0	0	0	78.7907	0	hypothetical protein, conserved
PVX_118620	171.977	60.0588	428.073	275.111	109.196	0	394.92	238.919	325.878	271.869	393.684	0	proteasome subunit alpha type-1, putative
PVX_118625	0	0	0	0	34.5516	0	0	302.291	41.2288	38.2072	21.6526	0	trailer hitch homolog, putative (CITH)
PVX_118630	0	0	0	0	0	0	0	0	0	0	0	0	hypothetical protein, conserved
PVX_118635	0	15.7926	56.2268	0	0	0	34.5733	0	42.8614	31.8087	27.0249	0	hypothetical protein, conserved
PVX_118640	69.2739	72.5285	0	110.657	0	0	0	96.19	52.488	24.3421	110.308	0	hypothetical protein, conserved
PVX_118645	0	0	0	0	31.1024	0	0	0	0	68.8082	0	158.79	cytochrome c oxidase assembly protein COX11, putative (COX11)
PVX_118648	5.45035	0	0	0	1.72692	0	0	0	0	1.91547	2.16963	0	hypothetical protein, conserved
PVX_118650	0	0	0	0	0	97.8053	0	0	7.24716	0	0	0	LCCL domain-containing protein (CCp1)
PVX_118655	0	29.9087	0	136.912	0	0	0	0	16.2326	30.1095	42.6409	0	bromodomain protein, putative
PVX_118660	67.7592	0	0	0	0	0	0	0	0	0	13.4876	0	tubulin epsilon chain, putative
PVX_118665	0	0	0	0	0	0	0	0	0	0	0	0	hypothetical protein, conserved
PVX_118670	0	41.5528	0	0	0	0	0	0	0	0	11.8461	0	hypothetical protein, conserved
PVX_118675	0	0	0	0	0	0	0	0	0	0	0	0	hypothetical protein
PVX_118680	0	0	0	0	0	0	0	0	0	0	0	0	early transcribed membrane protein (ETRAMP)
PVX_118682	0	0	0	0	0	0	0	0	0	0	0	0	erythrocyte membrane protein 3, putative
PVX_118685	0	231.419	0	0	0	849.776	0	0	62.7537	0	0	0	Plasmodium exported protein, unknown function
PVX_118690	0	89.3744	0	0	0	655.947	0	0	96.9644	0	25.4592	0	40 kDa heat shock protein, putative
PVX_118695	234.56	444.378	0	107.049	53.122	514.131	153.638	139.583	63.4723	105.972	100.045	0	Pv-fam-d protein
PVX_118700	0	0	0	0	0	0	0	0	0	20.7853	11.7756	0	PST-A protein
PVX_118705	0	111.635	0	0	0	0	0	0	121.15	56.1547	31.8179	0	hypothetical protein, conserved
PVX_119205	0	0	0	0	0	0	0	0	0	0	0	0	VIR protein,PIR protein
PVX_119210	0	0	0	0	0	0	0	0	0	0	7.82051	0	variant surface protein Vir22/5/24, putative,PIR protein
PVX_119215	0	0	0	0	0	0	0	0	0	0	19.2017	0	VIR protein,PIR protein
PVX_119220	112.375	353.063	139.741	0	17.825	287.563	0	156.072	255.475	217.158	111.841	0	Plasmodium exported protein, unknown function
PVX_119225	70.1493	293.914	0	0	44.5236	359.166	107.337	194.879	132.911	197.162	125.671	113.644	Plasmodium exported protein, unknown function
PVX_119230	129.255	135.38	0	0	61.5201	0	0	0	122.444	90.8239	38.5926	0	Plasmodium exported protein, unknown function
PVX_119235	0	0	0	0	0	0	0	0	0	0	0	0	Plasmodium exported protein, unknown function
PVX_119240	0	0	0	0	0	0	0	0	36.7978	34.1056	57.9802	0	hypothetical protein, conserved
PVX_119245	90.6833	63.3046	0	0	0	0	0	0	34.3574	79.6585	0	0	splicing regulatory protein, putative
PVX_119250	0	0	0	71.9433	7.14052	115.173	0	156.379	34.1337	47.4969	49.3212	0	serine/threonine protein kinase, putative (SRPK1)
PVX_119255	22.9956	0	57.1376	0	0	0	0	31.9269	8.71107	8.08088	9.15413	0	ATP-dependent transporter, putative
PVX_119260	0	0	0	0	50.0416	0	0	0	298.305	55.2566	156.622	0	hypothetical protein, conserved
PVX_119265	66.2543	138.791	0	0	0	0	202.729	92.0257	125.527	46.5546	26.3761	0	hypothetical protein, conserved
PVX_119270	48.3388	0	60.0561	154.386	7.6615	123.578	36.9281	134.228	27.4673	33.9732	48.107	0	exportin-1, putative
PVX_119275	71.8028	75.1775	0	114.701	0	0	0	49.8514	40.8034	50.4611	78.6055	58.1055	N-ethylmaleimide-sensitive fusion protein, putative
PVX_119280	0	0	0	0	0	0	0	31.0258	8.46523	0	0	0	hypothetical protein, conserved
PVX_119285	27.3868	0	0	87.4766	17.3642	0	0	0	0	19.2475	43.609	0	HAD superfamily protein, putative
PVX_119290	255.784	446.872	0	0	40.644	0	0	0	145.447	224.594	76.3777	0	DNA-directed RNA polymerases I, II, and III 14.4 kDa polypeptide, putative
PVX_119295	0	0	0	0	0	0	0	76.123	20.7678	0	10.9101	0	palmitoyltransferase, putative (DHHC1)
PVX_119300	15.7566	0	0	0	0	0	0	0	5.96851	0	0	0	hypothetical protein, conserved
PVX_119305	262.537	0	0	0	0	0	0	0	99.5268	0	52.2631	213.026	thioredoxin-like redox-active protein, putative
PVX_119310	77.6342	0	386.391	0	24.6417	0	0	0	0	0	92.7209	125.797	lipoamide acyltransferase component of branched-chain alpha-keto acid dehydrogenase complex, putative (BCKDH-E2)
PVX_119315	0	0	0	0	0	0	0	0	0	0	51.4319	0	hypothetical protein, conserved
PVX_119320	0	0	0	0	0	0	0	0	0	0	0	0	phosphatidylethanolamine-binding protein, putative
PVX_119325	0	0	0	0	0	0	0	0	0	0	0	0	inner membrane complex protein 1a, putative (IMC1a)

Gene ID / Patient	Patient 1	Patient 2	Patient 3	Patient 4	Patient 5	Patient 6	Patient 7	Patient 8	Patient 9	Patient 10	Patient 11	Patient 12	Gene Description
PVX_119330	0	0	0	0	0	0	0	0	24.4007	0	0	0	inner membrane complex protein 1e, putative (IMC1e)
PVX_119335	0	0	0	206.345	20.4771	0	0	0	0	22.6732	25.6913	0	EH (for Eps15 Homology) domain containing protein
PVX_119340	0	0	0	0	0	0	0	0	19.182	0	15.1181	0	hypothetical protein, conserved
PVX_119345	607.234	1701.26	1520.79	0	387.63	3130.98	936.11	845.403	1152.36	1704.7	846.363	0	60S ribosomal protein L44, putative (RPL44)
PVX_119350	0	204.307	0	0	0	0	0	406.119	0	102.382	0	0	1-cys-glutaredoxin-like protein-1, putative
PVX_119355	0	0	0	0	0	0	0	0	0	0	0	0	circumsporozoite (CS) protein (CSP)
PVX_119360	0	0	0	0	0	0	0	0	27.7576	0	0	0	hypothetical protein
PVX_119365	29.0964	40.615	0	0	0	0	133.389	0	0	0	23.1656	0	hypothetical protein, conserved
PVX_119370	1144.57	200.373	716.28	0	182.582	0	0	0	217.182	301.262	626.712	0	hypothetical protein, conserved
PVX_119375	92.2843	0	0	0	0	0	0	0	69.9505	32.4183	55.11	0	elongation factor (EF-TS), putative
PVX_119380	16.4438	11.4737	40.8412	0	5.21052	0	25.1115	0	6.22851	8.66841	16.3646	0	hypothetical protein, conserved
PVX_119385	31.3929	0	0	100.284	19.9061	0	0	0	0	44.1251	12.4971	0	hypothetical protein, conserved
PVX_119390	0	135.169	0	0	24.5623	198.107	59.201	0	44.0181	40.826	53.9617	0	hypothetical protein, conserved
PVX_119395	305.044	106.62	0	0	0	0	0	424.058	173.487	160.688	212.543	247.665	hypothetical protein, conserved
PVX_119400	24.7681	17.282	41.0108	0	2.61608	0	0	0	21.8903	11.6059	27.9353	13.3518	hypothetical protein, conserved
PVX_119405	0	0	0	0	0	0	0	0	0	0	50.7242	0	AP endonuclease (DNA-[apurinic or apyrimidinic site] lyase), putative
PVX_119410	268.042	187.33	668.091	1288.09	42.5981	0	205.487	745.103	406.462	329.49	453.551	0	ubiquitin-conjugating enzyme E2, putative
PVX_119415	0	25.1095	0	0	0	0	0	0	13.6285	0	0	0	hypothetical protein, conserved
PVX_119420	0	0	0	0	86.2896	0	416.669	0	0	0	0	440.82	hypothetical protein, conserved
PVX_119425	0	0	0	0	0	0	0	0	0	0	38.5014	0	hypothetical protein, conserved
PVX_119430	0	0	0	0	0	0	0	0	0	0	0	0	hypothetical protein, conserved
PVX_119435	0	48.8707	0	223.807	66.6285	0	107.085	97.2107	106.079	196.7	222.891	0	activator of Hsp90 ATPase, putative (AHA1)
PVX_119440	310.333	108.473	0	0	0	0	476.138	215.711	294.163	490.417	494.237	0	glutaredoxin 1, putative
PVX_119445	0	35.4033	0	0	0	0	0	70.4263	38.4277	17.818	80.7521	0	FAD-dependent glycerol-3-phosphate dehydrogenase, putative
PVX_119450	0	0	0	0	39.7884	0	191.921	348.024	0	87.9535	199.399	0	hypothetical protein, conserved
PVX_119455	0	0	0	0	0	0	0	329.61	89.8757	83.162	47.1674	0	hypothetical protein, conserved
PVX_119460	82.644	28.8448	0	132.039	0	0	0	0	46.966	72.598	32.8998	0	hypothetical protein, conserved
PVX_119465	192.355	134.312	159.5	205.013	40.6897	0	294.27	445.284	315.844	337.906	395.647	103.856	T-complex protein 1, beta subunit, putative
PVX_119470	2287.3	267.276	2870.53	4919.51	853.36	0	1178.3	2124.57	4922.05	3075.61	3416.1	1246.07	40S ribosomal protein S23, putative
PVX_119475	226.705	634.517	0	0	144.444	2332.42	1394.5	1576.92	687.994	1193.77	722.169	0	40S ribosomal protein S12, putative
PVX_119480	496.887	594.829	176.613	454.018	202.744	0	325.851	788.796	645.565	773.09	692.349	459.996	60S ribosomal protein L7, putative
PVX_119485	0	0	0	0	0	0	0	0	22.916	0	12.0383	0	microtubule associated protein EB1, putative
PVX_119490	84.9279	0	0	0	0	0	0	235.969	0	29.8352	67.622	137.643	ATP-dependent Clp protease proteolytic subunit, putative
PVX_119495	74.1603	51.7893	0	0	47.074	0	226.978	0	0	0	73.8095	0	hypothetical protein, conserved
PVX_119500	46.3035	0	0	0	29.3716	0	0	0	52.6297	0	46.0828	0	hypothetical protein, conserved
PVX_119505	18.4342	0	0	0	5.84266	0	0	0	6.9829	0	11.0074	0	hypothetical protein, conserved
PVX_119510	52.1293	0	129.633	0	33.0718	0	159.437	72.3964	39.5025	18.3161	114.139	84.4085	hypothetical protein, conserved
PVX_119515	0	0	0	0	2.56009	0	0	22.4314	3.06027	2.83939	3.21619	0	hypothetical protein, conserved
PVX_119520	66.6987	46.5741	0	0	21.165	0	102.046	277.93	75.8219	46.8668	119.489	216.087	DNA polymerase delta small subunit, putative
PVX_119525	18.9856	0	0	0	0	0	0	0	0	0	7.55777	0	hypothetical protein, conserved
PVX_119530	149.926	34.889	372.805	479.184	31.7035	0	305.677	277.613	113.609	316.069	268.581	80.9156	T-complex protein 1, eta subunit, putative
PVX_119535	0	0	0	0	0	0	0	0	0	0	20.6008	0	hypothetical protein, conserved
PVX_119540	0	0	0	0	106.76	0	0	0	126.883	117.269	0	0	activator of Hsp90 ATPase, putative
PVX_119545	111.674	0	0	0	0	0	0	77.5486	0	58.8554	22.2287	0	pre-mRNA-processing factor 19, putative (PRPF19)
PVX_119550	0	0	0	0	0	0	0	0	0	0	31.0687	0	hypothetical protein, conserved
PVX_119555	0	0	0	0	224.739	0	0	0	265.404	0	0	0	hypothetical protein, conserved
PVX_119560	0	48.2938	57.3145	0	7.31178	0	0	0	52.4281	16.2118	18.3649	0	splicing factor 3B subunit 1, putative
PVX_119565	60.9729	0	0	0	19.3453	0	0	0	46.2068	21.4224	157.777	98.7527	dual specificity protein phosphatase, putative (YVH1)
PVX_119570	0	0	0	0	0	0	0	0	0	0	55.0374	0	hypothetical protein, conserved
PVX_119575	0	0	0	0	0	0	0	0	0	0	0	0	serine/threonine protein kinase 6, putative
PVX_119580	0	0	0	0	0	0	0	0	16.2794	15.0981	25.6582	0	hypothetical protein, conserved
PVX_119585	103.655	108.549	0	165.657	32.88	0	0	71.977	78.5474	163.89	134.11	0	asparagine synthetase [glutamine-hydrolyzing], putative
PVX_119587	2002.95	1879.23	0	0	1075	0	0	932.808	3302.4	3040.28	2261.03	2199.87	60S acidic ribosomal protein P2, putative
PVX_119590	0	51.2789	0	0	23.3048	0	0	0	0	77.3924	43.8496	0	hypothetical protein, conserved
PVX_119595	0	88.6133	0	0	0	0	0	0	96.1394	44.5372	25.2428	0	YTH domain-containing protein, putative
PVX_119600	0	0	0	0	7.13513	0	0	0	0	7.91017	26.8822	0	hypothetical protein, conserved
PVX_119605	0	0	0	0	0	0	0	0	87.5542	0	91.9013	0	hypothetical protein, conserved
PVX_119610	0	0	0	0	0	0	0	0	0	0	0	0	calcium-dependent protein kinase 3, putative
PVX_119615	7.45732	15.6099	37.0425	23.8062	7.08882	0	11.3879	10.3521	11.2985	18.3454	26.717	12.0598	hypothetical protein, conserved
PVX_119620	0	31.6091	56.2695	144.652	7.17848	0	0	0	34.3151	23.8746	45.0757	36.6391	phosphoglycerate mutase, putative
PVX_119625	0	0	0	0	0	0	0	0	10.6687	0	0	0	hypothetical protein, conserved
PVX_119630	145.927	61.0974	36.2501	93.188	46.2468	0	22.2892	121.552	121.607	66.6669	90.0397	47.2075	DEAD box helicase, putative

Gene ID / Patient	Patient 1	Patient 2	Patient 3	Patient 4	Patient 5	Patient 6	Patient 7	Patient 8	Patient 9	Patient 10	Patient 11	Patient 12	Gene Description
PVX_119635	0	73.6634	0	0	33.4904	0	0	0	39.965	74.0749	41.9785	0	eukaryotic translation initiation factor 3 subunit 11, putative
PVX_119640	0	0	0	0	0	0	0	0	0	132.038	74.955	0	trafficking protein particle complex subunit 4, putative (TRAPPC4)
PVX_119645	0	0	0	0	0	0	0	0	0	244.256	138.934	0	hypothetical protein, conserved
PVX_119650	0	0	0	0	0	0	0	0	0	0	0	0	hypothetical protein, conserved
PVX_119655	0	0	0	0	0	0	0	0	0	0	0	0	hypothetical protein, conserved
PVX_119660	0	82.6108	0	0	18.769	302.797	0	0	89.6635	41.5706	70.6538	0	hypothetical protein, conserved
PVX_119665	24.2283	16.9088	0	0	0	0	37.0181	0	27.5343	34.056	57.8691	0	valine--tRNA ligase, putative
PVX_119670	0	0	0	0	9.79825	0	0	42.9115	11.7079	43.4395	49.2114	0	phosphatidylinositol 3- and 4-kinase, putative
PVX_119675	9.80669	0	0	0	0	0	0	13.6137	11.1437	3.44639	11.7114	0	protein kinase domain containing protein
PVX_119680	211.69	296.189	0	679.681	67.4135	1088.48	650.759	0	80.295	297.277	210.727	0	hypothetical protein, conserved
PVX_119685	0	0	0	0	15.1084	0	0	66.151	18.0476	16.7371	0	0	ribophorin I, putative
PVX_119690	0	0	0	0	0	0	0	0	0	0	13.7056	111.542	aspartyl proteinase, putative
PVX_119695	58.8093	82.1219	0	188.01	0	0	89.953	0	44.5665	103.312	152.178	95.2426	hypothetical protein, conserved
PVX_119700	0	0	0	0	0	0	0	0	0	0	11.6144	0	hypothetical protein, conserved
PVX_119705	0	113.811	0	0	51.778	0	0	0	0	57.1648	0	0	hypothetical protein, conserved
PVX_119710	44.7745	0	0	0	42.601	0	0	248.711	84.8188	78.6624	160.42	72.4845	E3 ubiquitin-protein ligase, putative
PVX_119715	0	0	0	0	0	0	0	0	0	9.9244	0	0	hypothetical protein, conserved
PVX_119720	252.536	176.475	0	0	80.2526	0	0	350.974	239.33	310.441	402.174	204.882	26S proteasome regulatory subunit RPN12, putative (RPN12)
PVX_119725	0	0	0	0	0	0	0	96.983	26.4576	0	13.8981	0	glycogen synthase kinase 3, putative (GSK3)
PVX_119735	0	44.4837	0	0	0	0	97.4603	88.4859	0	0	50.7242	103.189	transporter, putative
PVX_119740	662.171	555.324	990.229	0	336.738	679.336	0	736.271	1154.73	697.696	632.726	644.774	60S ribosomal protein L26, putative
PVX_119745	0	87.7791	0	200.974	0	0	0	174.609	71.4531	110.421	125.118	0	hypothetical protein, conserved
PVX_119750	41.533	28.9921	0	265.428	39.5136	212.468	126.985	0	125.882	58.3749	82.6695	0	ubiquitin-protein ligase, putative
PVX_119755	0	0	0	0	166.265	0	267.425	0	198.167	122.334	242.75	283.028	hypothetical protein, conserved
PVX_119760	0	175.088	0	0	0	0	0	0	0	0	199.221	0	hypothetical protein, conserved
PVX_119765	0	38.5511	0	0	0	0	0	153.374	41.8432	0	21.9818	89.4159	hypothetical protein, conserved
PVX_119770	42.6615	0	106.06	272.648	27.0589	0	65.2203	0	16.1629	59.9607	67.9327	69.0596	hypothetical protein, conserved
PVX_119775	51.3222	35.83	382.868	164.039	65.1182	0	313.929	0	311.125	198.358	143.018	83.0997	hypothetical protein, conserved
PVX_119780	54.0825	37.7584	0	0	0	0	82.7106	0	40.9832	38.0043	64.5904	87.5759	fusion protein, putative
PVX_119785	0	0	0	0	15.0124	0	144.743	0	35.8661	83.1545	37.6852	0	hypothetical protein, conserved
PVX_119790	68.4788	0	170.204	0	10.8563	0	0	47.5428	38.9141	36.0941	40.8908	55.413	hypothetical protein, conserved
PVX_119795	0	85.3431	0	0	38.8081	0	0	169.732	0	128.691	121.562	0	co-chaperone p23, putative
PVX_119800	0	422.249	0	0	0	0	0	0	76.3177	0	40.0601	0	hypothetical protein, conserved
PVX_119805	0	0	0	0	0	0	0	0	0	0	16.7623	0	hypothetical protein
PVX_119812	0	0	0	274.055	0	0	0	0	0	0	17.0511	0	DER1-like protein, putative
PVX_119813	25.6226	0	0	0	8.12244	0	0	35.575	48.5318	9.0039	10.1999	41.4575	hypothetical protein, conserved
PVX_119815	59.4737	83.0504	0	0	0	0	90.9712	82.6021	157.746	125.375	59.1912	0	serine/threonine protein phosphatase 6, putative (PPP6)
PVX_119820	295.661	51.6181	0	0	70.3773	0	113.113	205.349	56.0198	181.775	205.984	0	conserved Plasmodium protein, unknown function
PVX_119825	0	134.476	0	0	0	0	0	0	145.924	33.8123	38.3209	0	hypothetical protein, conserved
PVX_119830	0	0	0	0	0	0	0	0	0	0	0	0	hypothetical protein
PVX_120330	0	0	0	0	0	0	0	0	32.1856	0	0	0	variable surface protein Vir14-related
PVX_120335	0	0	0	0	0	0	0	0	0	25.0576	14.1971	0	variable surface protein Vir18-related
PVX_120340	0	54.0769	0	0	0	0	0	0	0	0	0	0	variable surface protein Vir12-related
PVX_120840	0	81.0239	0	0	0	0	0	0	0	40.7306	0	0	variable surface protein Vir8-related
PVX_120845	0	0	0	0	9.0955	0	0	0	7.24207	0	0	0	variable surface protein Vir12-related
PVX_121345	0	0	0	0	0	0	0	0	0	0	0	0	variable surface protein Vir6, putative
PVX_121350	0	0	0	0	0	0	0	0	0	0	0	0	hypothetical protein
PVX_121355	0	0	0	0	0	0	0	0	0	0	0	0	variable surface protein Vir15-like
PVX_121855	0	0	0	0	0	0	0	0	0	0	7.72674	0	variable surface protein Vir18, putative,PIR protein
PVX_121860	0	0	0	0	0	0	0	0	0	0	0	0	hypothetical protein
PVX_121862	0	0	0	0	0	0	0	0	0	0	0	0	VIR protein,PIR protein
PVX_121865	0	0	0	0	0	0	0	0	0	0	0	0	RAD protein (Pv-fam-e)
PVX_121870	0	403.548	0	0	0	0	0	0	0	0	0	0	Pv-fam-d protein
PVX_121875	0	0	0	0	0	0	0	0	83.6643	0	0	0	hypothetical protein
PVX_121876	0	134.766	0	0	0	0	0	0	0	33.8852	19.2017	0	Plasmodium exported protein, unknown function
PVX_121877	0	83.9658	0	0	0	0	0	0	45.5504	42.2063	0	0	Plasmodium exported protein, unknown function
PVX_121878	0	0	0	0	0	0	0	0	0	0	0	0	Plasmodium exported protein, unknown function, pseudogene
PVX_121879	0	0	0	0	0	0	0	0	0	78.0057	0	0	Plasmodium exported protein, unknown function
PVX_121880	174.74	585.442	0	111.652	33.2435	178.746	53.4149	0	119.159	147.364	83.4743	56.561	Plasmodium exported protein, unknown function
PVX_121885	0	0	0	0	0	0	0	0	0	0	0	0	cytoadherence linked asexual protein, CLAG, putative
PVX_121890	295.437	1444.67	0	315.183	218.917	0	301.642	547.313	298.596	484.306	392.065	0	hypothetical protein, conserved
PVX_121895	0	969.009	0	0	294.132	1187.41	0	0	262.663	162.04	183.803	0	Plasmodium exported protein, unknown function

Gene ID / Patient	Patient 1	Patient 2	Patient 3	Patient 4	Patient 5	Patient 6	Patient 7	Patient 8	Patient 9	Patient 10	Patient 11	Patient 12	Gene Description
PVX_121897	0	400.493	0	0	0	0	0	0	54.3081	0	57.0322	0	tryptophan-rich antigen (Pv-fam-a)
PVX_121900	0	0	0	0	0	0	0	0	55.3813	34.2394	9.69831	0	PST-A protein
PVX_121905	0	0	0	0	0	0	0	0	101.139	93.5509	0	0	hypothetical protein
PVX_121910	0	372.27	265.421	0	135.401	0	326.512	148.083	80.7872	336.908	296.999	0	Pv-fam-d protein
PVX_121912	94.6262	0	0	0	0	0	0	0	0	33.2406	0	0	Plasmodium exported protein, unknown function
PVX_121915	0	0	0	0	0	0	0	0	43.9555	40.7306	0	0	Plasmodium exported protein, unknown function
PVX_121920	12.3256	0	0	0	0	0	0	0	0	0	4.90654	0	reticulocyte binding protein 2a (RBP2a)
PVX_121925	0	172.191	0	0	0	0	0	0	0	0	0	0	hypothetical protein, conserved
PVX_121927	0	0	0	0	0	0	0	0	0	0	0	0	conserved Plasmodium protein, unknown function
PVX_121930	0	841.774	0	0	76.5188	0	0	0	274.056	141.143	127.957	0	Plasmodium exported protein, unknown function
PVX_121935	1512.42	1386.61	0	0	330.173	2421.66	868.497	656.656	931.462	1128.99	602.123	306.482	Plasmodium exported protein, unknown function
PVX_121940	0	0	0	0	0	0	0	0	32.7452	0	85.9966	0	hypothetical protein
PVX_121945	0	350.602	0	0	0	0	0	0	81.5368	50.3962	42.8304	0	gap, putative
PVX_121950	0	0	0	0	0	0	0	0	0	0	0	0	early transcribed membrane protein (ETRAMP)
PVX_121955	135.441	0	0	0	0	0	0	0	0	47.5683	26.9622	0	hypothetical protein
PVX_121960	0	0	0	0	0	0	0	0	0	0	35.7936	0	hypothetical protein, conserved
PVX_121965	11.5211	0	0	0	10.9529	0	35.1918	0	0	0	9.17256	18.6339	hypothetical protein, conserved
PVX_121970	53.5646	37.3966	0	0	16.9918	0	81.9172	0	0	131.741	85.2957	0	hypothetical protein, conserved
PVX_121975	0	26.8398	95.5539	0	0	0	0	0	14.5691	13.5157	15.3104	0	DEAD/DEAH box ATP-dependent RNA helicase, putative
PVX_121980	517.484	813.687	644.828	828.828	452.271	1990.69	198.328	539.415	833.738	999.635	901.38	0	40S ribosomal protein S7, putative
PVX_121985	73.0035	50.9568	0	0	0	0	0	0	41.486	25.6524	21.7964	0	hypothetical protein, conserved
PVX_121990	0	0	0	0	60.7671	0	0	265.503	72.4052	67.0332	0	0	hypothetical protein, conserved
PVX_121995	190.916	0	0	0	0	0	0	265.503	72.4052	201.1	266.06	0	Putative N6-DNA-methyltransferase, putative
PVX_122000	0	0	0	0	67.2536	0	0	0	0	148.288	84.0917	0	vesicle-associated membrane protein 714, putative,VAMP714, putative
PVX_122005	0	0	173.442	0	44.2457	0	213.333	0	52.8328	73.4752	27.7528	112.935	hypothetical protein, conserved
PVX_122010	89.4709	20.8152	0	0	0	0	0	41.4095	0	20.9599	29.6808	0	hypothetical protein, conserved
PVX_122015	0	0	0	0	0	0	0	0	13.2506	0	6.9625	0	sodium/hydrogen exchanger 1, putative
PVX_122020	0	0	0	0	69.0191	0	0	0	41.1787	0	21.6263	0	hypothetical protein, conserved
PVX_122025	0	0	0	0	11.008	0	53.0623	0	0	24.3987	48.3722	56.1876	hypothetical protein, conserved
PVX_122030	0	2.35679	0	0	4.28089	0	0	9.37789	1.27943	2.37429	8.7402	5.46206	hypothetical protein, conserved
PVX_122035	0	0	0	0	0	0	0	0	0	0	0	0	hypothetical protein, conserved
PVX_122040	0	0	0	0	0	0	0	45.6557	0	0	6.54469	0	hypothetical protein, conserved
PVX_122045	29.5772	20.6432	147.009	94.4788	28.1309	0	45.1984	41.0673	22.4095	10.3934	35.3227	47.8614	DNA ligase I, putative
PVX_122050	0	0	0	0	0	0	0	0	0	0	0	0	CorA-like Mg2+ transporter protein, putative
PVX_122055	0	120.118	0	275.111	27.2991	0	131.64	0	0	30.2077	17.1167	0	hypothetical protein, conserved
PVX_122060	0	0	0	0	0	0	0	0	0	0	21.0343	0	hypothetical protein, conserved
PVX_122065	0	241.194	286.648	0	73.1127	0	0	159.901	130.848	0	114.528	0	heat shock protein, putative
PVX_122070	0	0	0	0	0	0	0	0	0	0	0	0	hypothetical protein, conserved
PVX_122075	0	0	0	0	0	0	0	0	0	150.922	0	0	cyclin 4, putative
PVX_122077	0	0	0	0	0	0	0	0	0	0	5.00115	0	NLI interacting factor-like phosphatase, putative (NIF1)
PVX_122080	0	39.3033	0	0	0	0	0	0	0	59.3366	33.6157	91.1623	DNA-directed RNA polymerase II 13.3 kDa polypeptide, putative
PVX_122085	0	15.721	0	0	0	0	0	0	8.53343	0	26.9025	36.4453	hypothetical protein, conserved
PVX_122090	0	0	151.249	0	0	0	0	0	0	0	12.1037	0	hypothetical protein, conserved
PVX_122095	0	0	193.87	249.19	24.7276	0	0	216.44	29.5225	54.7377	124.058	0	transcription factor with AP2 domain(s), putative (ApiAP2)
PVX_122100	26.0895	18.2041	0	0	11.0227	0	0	60.362	26.3522	9.16874	24.2329	14.0643	hypothetical protein, conserved
PVX_122105	0	0	102.726	0	13.1042	0	63.1698	0	0	29.0392	57.5747	0	hypothetical protein, conserved
PVX_122110	0	0	0	0	10.2555	0	49.4338	0	49.0155	22.7323	51.5059	52.3459	mitogen-activated protein kinase phosphatase 1, putative (MKP1)
PVX_122115	0	0	0	0	35.9994	0	0	0	0	39.8028	90.23	0	hypothetical protein, conserved
PVX_122120	0	0	0	0	0	0	0	0	0	0	0	0	hypothetical protein, conserved
PVX_122125	0	0	0	0	0	0	0	0	0	0	0	0	hypothetical protein, conserved
PVX_122130	89.1921	62.263	110.876	285.028	28.2873	0	0	61.9297	101.377	94.0186	124.273	72.1952	hypothetical protein, conserved
PVX_122135	0	0	0	0	0	0	0	0	0	0	0	0	hypothetical protein, conserved
PVX_122140	0	0	369.603	0	0	0	0	0	0	52.0558	0	0	hypothetical protein
PVX_122145	72.8918	0	0	0	23.1337	0	0	0	27.6219	0	0	0	hypothetical protein, conserved
PVX_122150	0	0	0	0	0	0	0	0	10.9337	10.142	5.74472	0	hypothetical protein, conserved
PVX_122155	133.001	0	165.437	0	42.2039	0	0	184.736	151.193	70.0913	66.1852	215.444	26S protease regulatory subunit 10B, putative (RPT4)
PVX_122160	0	0	0	23.7173	0	0	0	0	0	0	1.47873	0	hypothetical protein, conserved
PVX_122165	76.4406	53.3834	0	0	0	0	0	106.185	57.9348	80.5642	76.0793	0	hypothetical protein, conserved
PVX_122170	0	0	0	0	42.6163	0	0	93.2694	25.4447	47.1832	26.7323	0	hypothetical protein, conserved
PVX_122175	0	0	0	0	27.7534	0	0	0	33.1293	0	0	0	hypothetical protein, conserved
PVX_122180	103.356	72.1568	0	0	16.3925	0	79.027	143.538	78.3205	0	82.291	83.6763	U1A small nuclear ribonucleoprotein, putative
PVX_122185	92.9553	64.9304	0	0	0	0	142.337	0	70.4594	0	55.5108	150.687	exosome complex component RRP40, putative (RRP40)

Gene ID / Patient	Patient 1	Patient 2	Patient 3	Patient 4	Patient 5	Patient 6	Patient 7	Patient 8	Patient 9	Patient 10	Patient 11	Patient 12	Gene Description
PVX_122190	31.2831	65.503	0	0	9.91822	0	0	0	0	32.9782	31.1334	0	U3 small nucleolar RNA-associated protein 6, putative
PVX_122195	0	0	0	0	0	0	0	54.4391	0	13.7756	39.0169	0	DnaJ protein, putative
PVX_122200	0	37.2404	0	0	0	0	0	74.0802	0	0	21.2349	0	DEAD box helicase, putative
PVX_122205	0	0	0	0	0	0	0	0	0	0	0	0	hypothetical protein, conserved
PVX_122207	0	0	0	0	0	0	0	0	0	0	0	1754.68	conserved Plasmodium protein, unknown function
PVX_122210	49.615	0	0	0	0	0	75.8675	206.709	18.7983	139.463	88.8817	0	DNA-directed RNA polymerase alpha chain, putative
PVX_122215	0	0	0	0	8.01905	0	0	0	9.58288	17.7788	15.1052	0	hypothetical protein, conserved
PVX_122218	84.9279	237.268	0	0	134.808	0	260.023	117.985	193.113	328.187	355.015	0	fork head domain protein, putative
PVX_122222	0	23.5737	0	0	0	0	0	0	0	11.8679	20.1676	54.6594	hypothetical protein, conserved
PVX_122225	0	0	106.979	0	0	0	0	0	0	15.1199	8.56509	69.6581	hypothetical protein, conserved
PVX_122230	0	0	0	0	0	0	0	0	0	0	0	0	hypothetical protein, conserved
PVX_122235	0	0	253.607	0	0	0	0	141.504	0	0	60.8177	0	transcription factor, putative
PVX_122240	37.9652	61.8141	94.3052	80.81	12.0312	0	19.3284	52.705	47.9357	44.4734	42.8202	20.4685	carbamoyl phosphate synthetase, putative
PVX_122245	1169.29	700.749	1666.94	535.65	265.683	857.747	1025.54	696.737	760.081	703.888	831.356	271.352	40S ribosomal protein S27, putative (RPS27)
PVX_122250	11.3594	15.8515	0	0	1.7996	0	0	0	2.15129	3.99214	9.0437	9.18463	hypothetical protein, conserved
PVX_122255	0	0	0	0	0	0	0	0	45.2809	20.9934	11.8936	0	hypothetical protein, conserved
PVX_122260	0	0	0	0	0	0	0	32.4269	8.84749	8.2074	9.29748	0	hypothetical protein, conserved
PVX_122265	0	0	0	0	0	0	0	0	0	0	0	0	hypothetical protein
PVX_122270	0	0	0	0	0	0	0	0	0	0	25.6543	0	tyrosine recombinase, putative (INT)
PVX_122275	0	0	0	0	13.9748	0	0	0	16.7011	23.2396	13.163	0	mRNA-decapping enzyme 2, putative (DCP2)
PVX_122280	96.7101	0	120.234	0	30.6745	0	73.9379	67.1522	36.6415	135.922	19.2499	0	hypothetical protein, conserved
PVX_122285	0	1278.27	1014.15	651.765	129.293	1043.75	0	847.21	1078.16	1425.75	1212.71	330.173	60S ribosomal protein L24, putative
PVX_122290	41.7072	0	103.685	0	52.906	0	63.7594	57.9163	15.8013	58.6196	58.1114	0	protein phosphatase 2C domain containing protein
PVX_122295	33.787	47.1653	0	0	0	0	0	93.8287	12.7999	0	53.8006	0	U4/U6 small nuclear ribonucleoprotein PRP3, putative (PRPF3)
PVX_122300	0	17.1268	0	0	0	0	0	68.1446	0	0	4.88458	0	HORMA domain protein, putative
PVX_122305	0	160.258	0	0	0	0	0	0	0	0	0	0	H/ACA ribonucleoprotein complex subunit 1, putative (GAR1)
PVX_122310	149.715	0	0	0	0	0	0	0	0	52.5779	29.8044	0	ribosomal RNA large subunit methyltransferase J, putative
PVX_122315	40.6903	14.1981	50.5482	0	12.8973	104.013	93.2439	28.2462	23.1207	35.7479	36.4455	0	vacuolar protein sorting-associated protein 18, putative (VPS18)
PVX_122320	43.6818	0	0	0	0	0	0	60.6596	0	30.6972	26.0841	0	hypothetical protein, conserved
PVX_122325	0	0	0	0	0	0	0	0	0	0	91.1955	0	hypothetical protein, conserved
PVX_122330	156.217	54.5494	0	0	49.585	0	119.546	0	59.1996	109.762	46.6437	0	ATP synthase delta chain, mitochondrial, putative
PVX_122335	30.9371	0	0	197.653	0	0	0	0	0	0	30.789	50.0639	hypothetical protein, conserved
PVX_122340	0	0	0	0	0	0	0	0	0	0	26.3246	0	hypothetical protein, conserved
PVX_122345	0	115.07	0	0	0	845.065	0	0	62.4071	0	0	0	zinc finger protein, putative
PVX_122350	0	22.0114	78.3785	0	0	0	48.1957	87.5776	35.8417	11.0818	50.2172	51.035	hypothetical protein, conserved
PVX_122355	0	79.1745	0	0	35.9994	580.942	173.629	0	0	39.8028	45.115	0	conserved protein, unknown function
PVX_122360	0	0	0	0	137.441	0	0	0	0	151.503	42.9585	0	ras-related protein Rab-5B, putative (RAB5b)
PVX_122365	0	117.672	0	0	0	0	0	0	0	59.0982	134.018	0	RNA-binding protein, putative
PVX_122370	0	33.3837	59.4297	0	7.5816	0	0	33.2071	9.06033	25.2144	9.52112	0	hypothetical protein, conserved
PVX_122375	253.61	0	0	406.164	80.5948	0	0	0	96.1394	133.612	25.2428	205.756	30S ribosomal protein S15, putative
PVX_122380	0	0	0	516.286	0	0	0	0	0	0	96.1718	0	protein ISD11, putative (ISD11)
PVX_122385	0	0	0	0	0	0	0	0	0	0	10.0997	0	hypothetical protein, conserved
PVX_122390	0	0	125.037	0	0	0	0	0	0	17.6679	0	0	alternative splicing regulator, putative
PVX_122395	0	57.8872	0	530.302	0	0	0	115.141	0	29.1171	32.9967	0	ATP synthase subunit gamma, mitochondrial, putative
PVX_122405	76.2453	106.494	189.732	0	24.2	0	0	0	144.467	133.931	121.416	0	AP-1 complex subunit mu, putative
PVX_122410	55.4566	77.4368	0	177.275	70.371	0	0	154.039	126.074	38.9696	176.617	179.609	26S protease regulatory subunit 7, putative (RPT1)
PVX_122415	0	44.6205	0	0	0	0	0	0	24.2188	22.4644	12.7247	0	hypothetical protein, conserved
PVX_122420	0	278.074	0	0	0	0	0	0	0	0	52.8054	0	cytochrome c, putative
PVX_122425	810.745	383.969	431.745	462.451	312.107	0	353.976	723.661	778.804	641.014	766.519	140.562	M1-family alanyl aminopeptidase, putative
PVX_122430	0	79.3494	0	0	0	0	57.9211	0	71.7788	13.3149	67.8811	0	vacuolar ATP synthase catalytic subunit A, putative
PVX_122435	0	0	0	0	0	0	0	0	34.0272	31.5406	35.7447	0	malonyl CoA-acyl carrier protein transacylase precursor, putative
PVX_122440	0	0	0	0	8.1716	0	0	0	0	0	5.1308	0	hypothetical protein, conserved
PVX_122445	69.3878	0	0	0	0	0	0	96.381	52.5869	24.3778	27.6237	0	hypothetical protein, conserved
PVX_122450	0	70.1068	0	0	31.8716	0	0	0	76.0731	0	59.931	0	hypothetical protein, conserved
PVX_122455	0	0	0	0	7.94713	0	38.305	0	28.4909	17.6194	14.9698	0	small GTP-binding protein, putative
PVX_122458	0	0	0	0	0	0	0	0	0	0	0	0	conserved Plasmodium protein, unknown function
PVX_122460	0	0	0	0	0	0	125.359	113.77	31.0362	0	130.417	0	2-oxoisovalerate dehydrogenase subunit alpha, mitochondrial, putative (BCKDHA)
PVX_122465	0	0	0	0	0	0	0	34.841	9.50647	0	2.49764	0	hypothetical protein, conserved
PVX_122470	54.9755	115.106	68.3079	87.7993	17.4282	140.558	0	114.496	114.544	77.2739	93.0106	133.433	eukaryotic translation initation factor 4 gamma, putative (EIF4G)
PVX_122475	0	0	0	0	0	0	0	0	0	0	55.3796	0	ubiquitin domain containing protein
PVX_122480	0	0	0	0	0	0	0	0	0	0	0	0	hypothetical protein, conserved
PVX_122485	34.8392	24.3174	0	0	11.0466	0	0	96.7519	52.7946	24.4842	34.6726	56.3847	methionyl-tRNA formyltransferase, putative (MTFMT)

Gene ID / Patient	Patient 1	Patient 2	Patient 3	Patient 4	Patient 5	Patient 6	Patient 7	Patient 8	Patient 9	Patient 10	Patient 11	Patient 12	Gene Description
PVX_122487	0	0	0	0	0	0	0	0	0	0	40.0601	0	peptidyl-prolyl cis-trans isomerase, putative
PVX_122490	0	0	0	112.576	0	0	0	48.9285	26.6988	0	63.1234	0	DEAD box helicase, putative
PVX_122495	0	0	0	0	0	0	0	0	7.63399	0	4.0112	0	hypothetical protein, conserved
PVX_122500	0	0	0	0	0	0	0	0	0	0	5.96663	0	hypothetical protein, conserved
PVX_122505	0	0	0	0	0	0	0	0	34.5827	32.0549	36.3279	0	septum formation protein Maf domain containing protein
PVX_122510	25.9332	0	21.4702	0	2.73916	0	13.2011	0	6.5486	12.1518	5.16167	0	hypothetical protein, conserved
PVX_122515	0	0	0	0	28.4211	0	137.054	0	67.8501	31.446	53.4561	0	hypothetical protein, conserved
PVX_122520	0	0	0	0	7.44816	0	0	32.6228	8.90093	8.25696	4.67681	0	hypothetical protein, conserved
PVX_122525	0	0	110.49	142.018	0	0	0	61.7144	33.6747	31.2306	26.5375	0	hypothetical protein, conserved
PVX_122530	12.0566	0	0	0	3.82072	0	0	33.475	9.13375	0	19.1978	0	telomerase reverse transcriptase, putative (TERT)
PVX_122535	54.9458	76.7232	273.295	0	0	0	0	0	62.4564	19.3054	98.4322	88.9761	hypothetical protein, conserved
PVX_122540	0	0	0	0	0	0	0	0	0	185.528	0	0	hypothetical protein, conserved
PVX_122545	0	0	0	0	31.6581	0	0	0	75.5643	0	39.687	0	cop-coated vesicle membrane protein p24 precursor, putative
PVX_122550	60.6835	0	0	194.012	38.5068	0	0	252.85	114.969	63.9622	60.3954	98.2831	lipoate-protein ligase 1, putative (LipL1)
PVX_122555	0	75.6083	0	173.085	34.3541	0	165.622	75.2013	61.5492	38.0503	118.559	0	hypothetical protein, conserved
PVX_122560	348.333	0	0	0	0	0	0	0	132.038	40.7834	208.023	188.347	ubiquitin-like protein, putative
PVX_122565	0	62.3374	0	0	56.6717	0	0	0	101.471	125.408	35.5308	0	TFIIH basal transcription factor subunit, putative
PVX_122570	0	0	0	0	64.4996	0	0	0	76.8367	142.252	80.6644	0	hypothetical protein, conserved
PVX_122575	99.0237	0	0	0	0	0	0	0	37.5312	0	39.4234	0	serine/threonine protein kinase, putative
PVX_122580	0	0	0	0	0	0	0	0	12.6518	0	13.2945	0	hypothetical protein, conserved
PVX_122585	0	0	0	0	0	0	0	0	0	0	0	0	hypothetical protein, conserved
PVX_122590	250.505	174.959	415.662	534.27	79.5235	0	0	232.003	158.224	146.672	182.837	270.652	zinc finger (CCCH type) protein, putative
PVX_122600	0	92.2536	0	0	0	0	101.065	0	50.0627	0	39.4473	0	hypothetical protein, conserved
PVX_122605	135.724	47.3871	0	217.006	21.5347	347.431	0	94.2602	51.4299	71.5259	81.0485	109.932	phosphatidylinositol synthase, putative (PIS)
PVX_122610	96.775	135.204	0	0	92.1946	0	444.611	0	110.035	33.9951	115.584	0	tRNA (adenine(58)-N(1))-methyltransferase catalytic subunit TRM61, putative (GCD14)
PVX_122615	94.0297	0	234.104	300.905	29.8577	0	0	130.641	35.6371	231.218	74.8699	152.434	transcription factor MYB1, putative (MYB1)
PVX_122620	50.7224	123.898	189.059	0	80.3952	259.352	77.5009	0	105.681	71.2964	65.623	123.103	exportin-1, putative
PVX_122625	0	0	0	0	0	0	0	0	0	0	0	0	protein kinase, putative
PVX_122630	23.1563	0	0	0	7.34017	0	35.379	0	8.77193	0	23.0452	0	inositol polyphosphate kinase, putative (IPK2)
PVX_122635	0	0	0	0	0	0	0	0	0	0	0	0	ADP-ribosylation factor, putative
PVX_122637	0	0	0	0	0	0	0	0	0	0	0	0	conserved Plasmodium protein, unknown function
PVX_122640	0	0	0	0	0	0	0	0	0	0	21.5218	0	hypothetical protein, conserved
PVX_122645	85.4283	0	0	0	9.02764	0	0	39.538	21.5751	30.0195	39.6756	46.0782	pre-mRNA-processing factor 40, putative (PRP40)
PVX_122650	36.324	25.3543	0	0	23.0357	0	0	0	13.7613	38.2912	72.3007	0	choline-phosphate cytidylyltransferase, putative
PVX_122655	0	0	0	0	0	0	80.2807	0	0	0	0	0	hypothetical protein, conserved
PVX_122660	0	92.1442	328.612	0	0	0	0	183.253	49.9836	185.231	131.235	0	protein transport protein SEC20, putative
PVX_122665	0	0	0	97.0139	19.2571	0	0	0	0	21.3439	30.2248	0	hypothetical protein, conserved
PVX_122670	96.5015	134.737	0	0	15.3041	0	0	67.0074	36.5624	50.8609	48.0209	0	U4/U6.U5 tri-snRNP-associated protein 2, putative
PVX_122675	33.5841	23.441	83.4717	0	0	0	0	0	0	11.8011	33.4235	0	DNA replication licensing factor MCM4, putative
PVX_122680	0	16.6576	0	38.1085	11.3475	0	0	16.57	18.0848	12.584	35.6358	38.6103	transcription factor with AP2 domain(s), putative (ApiAP2)
PVX_122685	55.3537	0	0	176.945	35.1201	0	169.316	153.753	83.8936	97.2432	99.1629	89.6375	hypothetical protein, conserved
PVX_122690	0	66.5215	0	0	0	0	0	132.31	144.37	234.169	151.652	154.385	hypothetical protein, conserved
PVX_122695	0	0	0	0	0	0	0	0	0	0	0	0	hypothetical protein, conserved
PVX_122700	0	0	0	0	36.7356	0	0	80.4099	0	40.6834	11.5242	0	hypothetical protein, conserved
PVX_122705	0	0	0	0	0	1650.24	0	445.437	121.429	0	0	0	hypothetical protein, conserved
PVX_122710	1567.98	767.306	782.135	0	249.328	0	240.584	653.946	1724.1	1486.71	811.58	254.638	40S ribosomal protein S19, putative (RPS19)
PVX_122715	0	0	0	0	22.9796	0	0	50.3158	27.4557	0	14.425	0	hypothetical protein, conserved
PVX_122720	0	0	0	0	0	0	0	0	0	0	0	0	hypothetical protein, conserved
PVX_122725	178.139	62.2132	0	0	0	0	136.37	0	67.5123	31.2895	53.1901	144.373	ferredoxin, putative
PVX_122730	0	0	0	0	0	0	0	0	0	0	0	0	glycerol-3-phosphate 1-O-acyltransferase, putative (G3PAT)
PVX_122735	0	14.3647	0	0	0	0	0	0	0	0	0	33.3	hypothetical protein, conserved
PVX_122740	0	65.4721	58.2764	0	0	0	0	0	0	8.24182	37.3459	0	structural maintenance of chromosome 2, putative
PVX_122742	0	0	0	0	0	0	0	0	0	0	0	0	conserved Plasmodium protein, unknown function
PVX_122745	0	70.052	0	0	0	0	0	0	0	17.6283	69.9054	0	hypothetical protein, conserved
PVX_122750	0	0	0	0	0	0	0	0	0	0	7.84116	0	hypothetical protein, conserved
PVX_122755	177.795	99.2803	0	113.606	33.8252	0	0	0	80.8285	74.9699	14.1556	0	translocation protein SEC63, putative (SEC63)
PVX_122760	0	0	0	0	0	0	0	0	0	0	10.2686	0	hypothetical protein, conserved
PVX_122765	42.2985	29.5268	0	0	0	0	0	0	32.0508	14.8627	8.41934	0	hypothetical protein
PVX_122770	88.1814	30.7785	0	0	13.9832	225.569	0	0	16.7046	30.9845	17.5522	0	conserved protein, unknown function
PVX_122775	0	0	0	0	0	0	0	0	0	0	57.9493	67.3245	hypothetical protein, conserved
PVX_122780	53.8022	112.649	0	0	17.056	137.556	82.2108	37.3506	61.1447	37.8125	21.4178	0	N(2),N(2)-dimethylguanosine tRNA methyltransferase, putative
PVX_122785	29.7938	20.7945	0	0	0	0	0	0	11.2868	20.939	53.3721	48.2123	hypothetical protein, conserved

Gene ID / Patient	Patient 1	Patient 2	Patient 3	Patient 4	Patient 5	Patient 6	Patient 7	Patient 8	Patient 9	Patient 10	Patient 11	Patient 12	Gene Description
PVX_122790	0	0	0	0	0	0	0	0	0	0	130.188	0	hypothetical protein, conserved
PVX_122795	0	0	39.5396	0	0	0	0	22.0965	0	0	19.0078	0	hypothetical protein, conserved
PVX_122800	0	0	0	0	0	0	0	52.4841	0	0	22.5697	0	protein phosphatase PP2A regulatory subunit A, putative
PVX_122805	0	0	0	0	0	0	0	0	9.68141	0	0	0	hypothetical protein, conserved
PVX_122810	23.9752	16.7321	0	0	0	0	0	0	54.4931	16.8501	38.1763	0	hypothetical protein, conserved
PVX_122815	0	0	0	310.051	0	0	148.365	0	36.718	34.0318	96.4245	0	ATP-dependent Clp protease adaptor protein ClpS, putative
PVX_122820	0	0	0	0	0	0	0	0	0	39.6825	0	91.4504	TBC domain protein, putative
PVX_122825	40.2986	0	0	0	0	0	0	0	0	0	0	0	hypothetical protein, conserved
PVX_122830	119.682	0	0	0	0	0	183.419	0	45.3675	0	0	0	big signal peptidase, putative
PVX_122835	0	0	0	0	0	0	0	0	0	148.288	0	0	SNARE protein, putative
PVX_122840	67.3006	93.9895	0	0	0	0	0	0	51.0043	94.5792	147.36	0	ras-related protein Rab-11A, putative (RAB11a)
PVX_122845	0	0	0	0	0	0	0	26.2969	0	0	3.77009	0	hypothetical protein, conserved
PVX_122850	0	0	0	0	41.524	0	100.102	181.762	24.7933	91.9524	117.217	0	dihydrolipoyllysine-residue succinyltransferase component of 2-oxoglutarate dehydrogenase complex, putative
PVX_122855	177.3	0	0	0	0	0	0	0	134.47	0	70.5941	288.1	cyclophilin E, putative,peptidyl-prolyl cis-trans isomerase E, putative
PVX_122860	0	0	0	0	0	0	0	0	0	0	0	0	hypothetical protein, conserved
PVX_122865	0	0	0	0	0	0	0	0	0	0	0	0	hypothetical protein, conserved
PVX_122870	128.998	0	0	0	0	0	0	0	0	271.842	77.0384	0	hypothetical protein, conserved
PVX_122875	40.3089	14.0631	0	32.1722	6.38659	0	0	13.9893	11.4512	21.2488	10.0287	16.2979	hypothetical protein, conserved
PVX_122880	0	0	0	0	0	0	0	0	0	0	25.791	0	palmitoyltransferase, putative (DHHC8)
PVX_122885	0	0	0	86.8384	0	0	0	0	0	0	5.41137	0	3',5'-cyclic nucleotide phosphodiesterase, putative
PVX_122890	0	0	0	0	0	0	0	0	0	0	0	0	3',5'-cyclic nucleotide phosphodiesterase, putative
PVX_122895	0	71.7998	0	328.977	0	0	0	0	0	36.1022	143.212	166.654	hypothetical protein, conserved
PVX_122900	0	0	0	0	0	0	0	0	0	0	0	0	hypothetical protein, conserved
PVX_122905	36.6963	25.6143	0	351.729	11.636	0	0	0	41.7072	38.6836	51.1293	0	hypothetical protein, conserved
PVX_122910	0	0	0	0	0	0	292.626	264.937	72.2509	200.672	113.783	0	hypothetical protein, conserved
PVX_122915	161.03	0	0	0	0	0	0	0	0	0	32.0573	0	hypothetical protein, conserved
PVX_122920	17.0989	35.7924	0	27.2935	13.5453	0	0	23.7365	25.9066	18.0274	39.138	0	hypothetical protein, conserved
PVX_122925	0	0	0	0	0	0	0	0	0	0	0	0	hypothetical protein
PVX_122930	0	0	0	358.725	35.5926	0	0	0	84.9372	118.064	156.123	0	histone H2B variant, putative (H2B.Z)
PVX_122935	0	0	112.22	0	0	0	0	0	0	0	0	0	hypothetical protein, conserved
PVX_122940	0	14.6546	0	0	0	0	32.0811	0	0	0	4.17968	0	hypothetical protein, conserved
PVX_122945	31.5256	22.0036	78.3509	0	9.99515	0	0	0	23.886	0	25.0998	102.034	palmitoyltransferase, putative (DHHC4)
PVX_122947	0	0	0	539.698	0	0	0	0	63.8171	0	0	0	calmodulin, putative
PVX_122950	42.9069	29.9517	0	0	0	0	0	59.5831	65.0238	15.0764	59.7831	69.4574	transcription elongation factor, putative
PVX_122955	0	0	0	0	0	0	0	0	44.1026	40.895	11.5842	0	hypothetical protein, conserved
PVX_122956	0	0	0	0	0	0	0	0	0	0	0	0	tRNA Asparagine
PVX_122957	0	0	0	0	0	0	0	0	0	0	0	0	tRNA Leucine
PVX_122958	0	0	0	0	0	0	0	0	0	0	0	0	tRNA Serine
PVX_122960	0	0	0	0	43.3991	0	104.625	189.962	103.646	72.0722	136.113	0	phosphoglycolate phosphatase precursor, putative
PVX_122962	0	0	0	0	0	0	1436.13	0	0	0	0	0	conserved Plasmodium protein, unknown function
PVX_122965	0	0	0	0	6.40929	0	0	0	0	0	8.04959	0	hypothetical protein, conserved
PVX_122970	0	0	0	0	0	0	0	0	0	0	0	0	calcium-dependent protein kinase 3, putative
PVX_122975	0	0	0	0	0	0	0	0	0	0	0	0	hypothetical protein, conserved
PVX_122980	0	0	0	0	0	0	0	0	0	100.101	113.584	0	ATP synthase epsilon chain, mitochondrial , putative
PVX_122985	34.9344	24.3839	0	0	11.0768	0	0	48.5082	66.1736	61.3777	20.8604	0	GTP-binding protein TypA, putative
PVX_122990	0	0	0	0	0	0	0	0	0	0	109.238	900.579	hypothetical protein, conserved
PVX_122995	76.2374	0	94.7544	243.585	12.0874	0	58.2669	105.863	72.2069	13.3943	113.81	61.698	transporter, putative
PVX_123000	227.176	0	141.251	0	18.0176	0	86.8648	78.8781	21.5193	39.9091	56.524	0	cation diffusion facilitator transporter domain containing protein
PVX_123005	0	23.681	0	0	10.7574	0	103.708	0	25.7066	0	47.2718	0	FAD-dependent monooxygenase, putative
PVX_123010	147.087	51.3339	91.4026	0	23.3199	0	0	102.121	83.5857	116.289	117.107	0	importin-beta 2, putative
PVX_123015	0	0	0	0	0	0	0	0	0	0	0	0	hypothetical protein
PVX_123020	0	0	0	108.761	0	0	0	0	0	11.9627	6.77624	0	hypothetical protein, conserved
PVX_123025	215.97	301.774	0	0	34.3008	0	165.43	300.1	81.8603	113.792	64.4875	175.126	selenoprotein, putative (Sel3)
PVX_123030	302.425	211.269	251.029	0	32.0155	0	154.4	0	76.4161	141.645	301.005	0	superoxide dismutase [Fe], putative
PVX_123035	0	0	0	0	0	0	0	0	0	0	0	0	hypothetical protein, conserved
PVX_123040	0	0	0	0	0	0	0	0	0	0	0	0	hypothetical protein, conserved
PVX_123045	0	0	0	0	0	0	0	0	0	0	0	0	hypothetical protein, conserved
PVX_123050	0	0	0	0	16.421	0	0	0	19.6141	0	10.3042	0	phospholipase DDHD1, putative
PVX_123055	27.752	77.4754	0	0	0	0	0	38.5323	63.0791	19.5042	5.52381	0	AAA family ATPase, putative
PVX_123060	2524.94	1137.7	608.137	1302.78	749.799	834.332	1496.08	3168.16	1944.6	2174.65	1896.93	395.98	DNA/RNA-binding protein Alba 1, putative (ALBA1)
PVX_123065	100.808	0	0	0	0	0	0	0	38.208	35.4112	20.067	0	hypothetical protein, conserved
PVX_123070	990.038	790.841	1057.79	1359.63	314.738	725.698	433.809	786.375	1018.79	1043.11	1126.22	459.177	60S ribosomal protein L13, putative

Gene ID / Patient	Patient 1	Patient 2	Patient 3	Patient 4	Patient 5	Patient 6	Patient 7	Patient 8	Patient 9	Patient 10	Patient 11	Patient 12	Gene Description
PVX_123075	638.807	557.674	397.448	255.429	152.08	408.958	488.878	443.703	695.99	476.89	524.527	258.793	40S ribosomal protein S16, putative
PVX_123080	0	71.7998	0	0	0	0	0	0	0	0	20.4589	0	GDP-mannose 4,6-dehydratase, putative (GMD)
PVX_123085	24.5878	0	61.0963	0	0	0	0	0	9.31428	8.6403	9.78794	0	ABC transporter, putative
PVX_123090	0	86.3228	0	0	19.613	0	94.5599	257.569	46.8454	130.309	61.5218	0	translation initiation factor SUI1, putative
PVX_123095	0	0	0	0	0	0	0	0	0	0	0	0	hypothetical protein
PVX_123100	99.7276	243.678	0	0	47.4491	0	0	69.2486	18.8926	105.121	99.2528	80.735	hypothetical protein, conserved
PVX_123105	134.189	187.35	222.417	0	70.9306	228.843	0	186.347	186.415	157.168	222.582	144.824	hypothetical protein, conserved
PVX_123110	153.143	213.899	0	489.833	121.519	1960.62	0	106.367	377.221	538.014	137.177	0	Mitotic apparatus protein p62, putative
PVX_123115	0	48.6044	0	0	22.0884	0	0	0	79.1258	24.4537	83.1289	0	nuclear movement protein, putative
PVX_123120	0	0	0	0	0	0	0	0	0	0	0	0	hypothetical protein, conserved
PVX_123125	0	0	0	0	0	0	0	0	18.2812	0	0	0	hypothetical protein, conserved
PVX_123130	291.625	0	0	0	0	0	0	0	0	51.2082	0	0	hypothetical protein, conserved
PVX_123135	0	0	0	0	0	0	0	101.334	55.2888	25.6293	43.5636	0	hypothetical protein, conserved
PVX_123140	0	0	0	0	0	0	0	0	0	42.8387	24.2793	0	ubiquitin-conjugating enzyme E2, putative
PVX_123145	0	102.789	0	0	15.5627	0	37.5059	68.1633	83.6906	51.7565	87.9465	39.7165	hypothetical protein, conserved
PVX_123150	62.3753	43.5528	0	0	39.582	0	0	0	165.445	109.575	198.654	0	karyopherin alpha, putative
PVX_123155	0	0	0	0	0	0	0	0	29.6516	0	0	0	sporozoite surface protein 3, putative (SSP3)
PVX_123158	0	0	0	0	0	0	0	0	0	0	12.9767	0	trypsin-like serine protease, putative
PVX_123162	134.222	0	0	0	42.6622	0	0	0	50.884	141.421	133.598	0	GTPase, putative
PVX_123165	12.6873	35.4125	63.0306	0	20.1033	0	19.3777	17.6132	48.058	22.2934	35.3537	0	hypothetical protein, conserved
PVX_123170	0	0	0	399.697	59.4975	0	95.6187	0	23.6846	21.9608	12.4419	0	hypothetical protein, conserved
PVX_123175	0	17.3312	0	0	7.8721	0	0	34.479	9.40734	0	9.88572	0	hypothetical protein, conserved
PVX_123180	0	0	0	0	21.0237	0	0	0	0	0	13.188	0	hypothetical protein, conserved
PVX_123185	0	0	0	0	0	0	0	36.1338	29.5764	9.14527	41.4403	0	hypothetical protein, conserved
PVX_123190	0	15.3386	0	0	0	0	0	0	0	0	4.37472	0	hypothetical protein, conserved
PVX_123195	0	27.3896	0	0	0	0	0	0	14.8657	13.7876	7.81022	0	hypothetical protein, conserved
PVX_123200	0	54.0443	192.463	0	0	0	59.1754	0	146.663	81.6168	92.4658	0	conserved protein, unknown function
PVX_123205	0	10.6356	37.8621	0	4.83032	0	0	0	5.77332	5.35616	3.03361	0	CCR4-associated factor 1, putative (CAF1)
PVX_123210	0	19.8997	70.8561	0	0	0	0	0	21.6026	10.0192	28.3758	0	hypothetical protein, conserved
PVX_123215	0	0	0	132.837	0	0	0	0	15.7499	43.8218	16.5493	0	mitochondrial carrier protein, putative
PVX_123220	0	0	84.1992	0	0	0	0	0	0	23.8077	13.4858	0	cullin-1, putative (CUL1)
PVX_123225	0	0	0	0	0	0	0	0	0	0	0	0	hypothetical protein, conserved
PVX_123230	117.612	0	0	250.528	111.888	0	0	0	163.38	137.756	101.444	63.4567	hydroxymethylpterin pyrophosphokinase-dihydropteroate synthetase, putative
PVX_123235	0	0	0	0	0	0	0	0	0	0	155.81	639.553	hypothetical protein, conserved
PVX_123240	104.518	437.713	173.189	333.911	154.653	534.567	53.2484	241.88	303.569	330.537	305.119	56.3847	DEAD/DEAH box helicase, putative
PVX_123245	0	88.2376	0	0	40.1263	0	0	0	47.866	44.3488	0	0	Protein phosphatase 2C containing protein
PVX_123250	48.565	67.8077	0	155.214	46.2117	0	0	0	18.4003	119.448	29.0002	0	aquaporin, putative (AQP2)
PVX_123260	91.1916	95.4894	113.365	0	14.4611	0	0	0	34.55	80.1048	36.3027	73.8157	hypothetical protein, conserved
PVX_123265	93.8494	0	0	0	0	0	95.6158	0	0	32.9782	31.1334	0	hypothetical protein, conserved
PVX_123270	0	0	0	500.895	49.6908	0	0	0	0	109.742	31.1056	0	50S ribosomal protein L33, putative
PVX_123275	119.363	83.405	0	0	75.8512	0	0	0	90.4927	167.7	71.2834	0	acyl-CoA binding protein, putative
PVX_123283	0	33.0742	0	0	7.5113	0	0	0	8.97636	16.6538	0	0	JmjC domain containing protein (JmjC1)
PVX_123290	0	0	0	0	8.78158	0	0	0	10.4937	0	5.51355	0	hypothetical protein, conserved
PVX_123295	54.0499	0	0	0	34.2918	0	0	75.065	0	18.9907	32.2757	0	RuvB-like helicase 1, putative (RUVB1)
PVX_123300	0	0	0	0	0	0	0	0	0	1.91349	3.25108	0	hypothetical protein, conserved
PVX_123305	0	0	0	0	80.8745	0	0	0	0	89.0482	202.045	0	histone acetyltransferase subunit NuA4, putative
PVX_123307	0	0	0	0	11.6169	0	0	50.8721	55.5185	77.2406	51.0456	0	conserved Plasmodium protein, unknown function
PVX_123310	0	33.4373	0	76.5106	15.1876	0	36.6016	66.5208	27.2247	16.8366	9.53641	38.759	hypothetical protein, conserved
PVX_123315	43.9176	0	0	0	13.9283	0	0	0	0	0	34.9665	0	hypothetical protein, conserved
PVX_123320	19.862	0	0	0	6.29543	0	0	0	0	13.9596	27.6733	0	hypothetical protein, conserved
PVX_123325	0	0	0	0	31.1365	0	0	0	37.1608	68.8835	39.0347	0	hypothetical protein, conserved
PVX_123330	0	0	0	0	0	0	0	0	43.7892	40.6046	34.5057	0	glycerol-3-phosphate 1-O-acyltransferase, putative
PVX_123335	0	0	0	488.892	0	0	0	0	115.658	107.126	30.3633	0	conserved protein, unknown function
PVX_123340	65.819	137.791	204.389	157.627	46.9353	0	50.2696	91.3765	155.825	156.129	114.63	0	eukaryotic translation initiation factor 3 subunit 10, putative
PVX_123345	0	0	0	0	0	0	0	0	41.3798	0	65.1956	0	iron-sulfur subunit of succinate dehydrogenase, putative
PVX_123350	0	40.8141	0	0	0	0	0	40.5975	0	20.5491	0	0	bromodomain protein, putative
PVX_123355	137.946	96.4128	343.867	441.988	0	0	211.532	0	52.2971	242.238	54.922	0	hypothetical protein, conserved
PVX_123357	0	0	0	0	0	0	901.703	0	0	0	115.482	0	conserved Plasmodium protein, unknown function
PVX_123360	0	0	0	0	0	0	0	0	0	0	0	0	hypothetical protein, conserved
PVX_123365	0	0	0	0	0	0	0	0	0	0	0	0	integral membrane protein GPR180, putative
PVX_123370	0	0	0	0	0	0	0	0	0	0	0	0	dynein light chain 1, putative
PVX_123375	0	172.191	0	0	0	0	0	0	186.681	0	97.9667	0	RNA polymerase subunit 8c, putative

Gene ID / Patient	Patient 1	Patient 2	Patient 3	Patient 4	Patient 5	Patient 6	Patient 7	Patient 8	Patient 9	Patient 10	Patient 11	Patient 12	Gene Description
PVX_123380	0	88.1075	0	0	10.0057	0	48.2298	43.8197	35.867	22.1793	25.1263	0	bifunctional aminoacyl-tRNA synthetase, putative
PVX_123385	0	20.857	74.2661	0	18.9482	0	45.6667	0	45.2831	42.0037	11.8961	0	hypothetical protein, conserved
PVX_123390	0	0	0	0	36.3222	0	0	0	86.6747	40.1586	45.5185	0	hypothetical protein, conserved
PVX_123395	0	17.2737	0	0	15.6919	0	0	34.3645	9.37612	8.69764	19.7058	40.0462	GPI ethanolamine phosphate transferase 3, putative (PIGO)
PVX_123400	0	62.8393	0	0	28.5642	0	0	124.989	34.0957	0	17.9083	0	SET domain containing protein
PVX_123405	59.2767	124.163	0	0	94.032	0	90.6693	82.3283	134.763	124.96	129.789	96.0009	geranylgeranyl transferase type2 beta subunit, putative
PVX_123407	511.38	0	0	1655.89	0	0	0	0	0	358.53	101.851	0	conserved Plasmodium protein, unknown function
PVX_123410	0	0	0	0	0	0	0	0	0	0	57.1237	0	hypothetical protein, conserved
PVX_123415	0	0	0	0	0	0	0	0	0	0	0	0	adrenodoxin-type ferredoxin, putative
PVX_123420	0	0	0	0	0	0	0	0	74.9793	69.4923	19.6899	0	hypothetical protein, conserved
PVX_123425	0	0	0	0	0	0	0	0	0	0	0	0	hypothetical protein, conserved
PVX_123430	0	0	0	536.276	26.6073	0	0	116.436	31.7633	29.4442	50.0514	135.834	hypothetical protein, conserved
PVX_123435	133.421	0	332.546	0	0	0	0	0	50.5802	46.8596	185.921	0	thioredoxin peroxidase 2, putative
PVX_123440	0	0	0	0	0	0	0	0	0	12.1994	6.91031	0	hypothetical protein, conserved
PVX_123445	0	0	0	0	0	0	0	0	0	0	0	0	cyclophilin, putative
PVX_123450	666.919	116.573	0	534.647	53.0372	0	0	0	379.332	585.481	265.538	270.844	10 kDa chaperonin, putative
PVX_123455	0	38.6226	0	0	17.5492	0	0	0	41.9208	38.8732	55.0564	89.582	hypothetical protein, conserved
PVX_123457	0	0	0	0	0	0	0	0	0	0	0	0	conserved Plasmodium protein, unknown function
PVX_123460	24.608	0	0	0	0	0	0	0	0	8.64742	4.89801	0	hypothetical protein
PVX_123465	0	0	0	0	0	0	0	0	0	0	111.247	0	hypothetical protein, conserved
PVX_123470	0	0	0	0	0	0	0	0	0	0	0	0	hypothetical protein, conserved
PVX_123475	0	29.1138	103.685	0	26.453	0	0	115.833	63.2051	87.9295	41.5081	0	hypothetical protein, conserved
PVX_123480	0	0	0	0	0	0	0	0	56.2389	52.154	29.5453	0	serine--tRNA ligase, putative
PVX_123485	0	0	0	0	0	0	0	0	0	43.9767	0	0	hypothetical protein, conserved
PVX_123490	0	69.4054	0	317.99	0	0	0	0	301.25	69.8008	0	0	glycerol-3-phosphate dehydrogenase, putative
PVX_123495	0	0	0	376.206	0	0	180.035	163.263	89.066	123.795	23.3868	0	signal recognition particle subunit SRP19, putative (SRP19)
PVX_123500	0	30.3006	0	0	13.766	0	0	60.277	16.4452	30.5037	17.2797	0	hypothetical protein, conserved
PVX_123505	159.987	111.734	995.401	255.887	126.96	0	489.754	888.994	454.718	533.947	589.157	777.769	male development gene 1, putative (MDV1)
PVX_123510	0	0	0	0	0	0	0	0	0	0	0	0	cell traversal protein for ookinetes and sporozoites (CelTOS)
PVX_123515	28.7598	0	0	91.8656	9.11764	0	0	0	0	10.1062	11.4488	0	perforin-like protein 2 (PLP2)
PVX_123517	0	0	0	0	0	0	0	0	0	0	0	0	tRNA Proline
PVX_123520	0	0	73.2381	0	9.34298	0	0	0	11.1642	31.0672	17.5974	47.6879	DNA-binding chaperone, putative
PVX_123525	0	0	0	0	132.127	0	0	0	0	0	0	0	hypothetical protein, conserved
PVX_123530	0	0	0	0	0	0	0	0	0	0	0	0	meiotic recombination protein SPO11, putative (SPO11)
PVX_123535	28.1359	58.9106	0	0	0	0	42.9939	0	21.3173	19.7739	11.2004	45.5273	RNA-binding protein, putative
PVX_123540	72.7365	0	90.3987	0	0	0	55.5881	50.4999	27.5561	76.6757	57.9111	0	GTP-binding protein, putative
PVX_123545	0	19.4533	0	0	0	0	42.5918	0	0	0	11.0958	0	hypothetical protein, conserved
PVX_123550	51.6778	72.1568	128.508	0	32.785	0	0	71.769	39.1603	72.6298	51.4319	0	hypothetical protein, conserved
PVX_123555	0	0	0	0	0	0	0	0	0	0	0	0	hypothetical protein, conserved
PVX_123560	0	0	0	0	0	0	0	0	25.9711	0	13.6426	0	hypothetical protein, conserved
PVX_123565	0	0	0	0	0	0	0	0	0	0	31.8851	0	zinc finger protein, putative
PVX_123570	0	58.6485	0	0	0	0	0	116.655	0	0	16.7151	0	hypothetical protein, conserved
PVX_123575	0	0	0	0	0	0	0	0	0	0	0	0	thrombospondin-related apical membrane protein, putative (TRAMP)
PVX_123580	0	0	0	0	0	0	0	95.8604	0	0	0	0	hypothetical protein, conserved
PVX_123585	0	39.4949	0	45.179	0	0	21.6122	0	16.0793	9.94512	5.63267	0	hypothetical protein, conserved
PVX_123590	0	0	0	0	0	0	83.827	76.123	0	19.2581	10.9101	0	AP-3 complex subunit mu, putative
PVX_123592	144.372	0	0	0	22.9094	0	0	100.271	54.7088	50.7213	43.1068	0	triose or hexose phosphate/phosphate translocator, putative
PVX_123595	23.6708	16.5196	0	75.5993	0	0	0	0	0	0	14.1343	0	hypothetical protein, conserved
PVX_123597	0	0	96.4822	0	0	0	0	0	44.1135	27.2764	15.4511	0	arginyl-tRNA synthetase, putative
PVX_123600	0	0	0	0	0	0	0	0	0	0	0	0	hypothetical protein, conserved
PVX_123605	0	0	0	0	0	0	0	0	0	0	0	0	hypothetical protein, conserved
PVX_123610	0	0	0	0	12.7449	0	0	0	15.2263	0	7.99963	0	hypothetical protein, conserved
PVX_123615	0	0	0	0	0	0	0	0	0	7.20049	0	0	formin 2, putative
PVX_123620	47.9022	22.2848	39.6666	0	0	0	0	44.3349	36.2903	72.9468	28.6032	0	clathrin heavy chain, putative
PVX_123625	0	66.9987	0	0	18.2579	98.1622	0	79.9743	43.6416	20.2431	38.2186	31.0625	aminophospholipid-transporting P-ATPase, putative
PVX_123630	0	0	0	0	0	0	0	0	0	0	0	0	raf kinase inhibitor, putative (RKIP)
PVX_123632	0	0	0	0	0	0	0	0	0	0	0	0	conserved Plasmodium protein, unknown function
PVX_123635	0	0	464.662	0	0	0	0	0	0	130.723	37.0596	0	D-ribulose-5-phosphate 3-epimerase, putative
PVX_123640	0	0	0	0	0	0	0	86.2736	0	0	0	0	hypothetical protein, conserved
PVX_123645	41.4944	57.9304	103.156	0	0	0	0	57.6208	15.7207	43.7405	33.0371	0	pre-mRNA splicing factor PRP17, putative
PVX_123650	0	0	0	0	0	0	0	0	0	0	2.76905	0	hypothetical protein, conserved
PVX_123655	0	0	0	0	0	0	0	0	0	0	2.20869	0	hypothetical protein, conserved

Gene ID / Patient	Patient 1	Patient 2	Patient 3	Patient 4	Patient 5	Patient 6	Patient 7	Patient 8	Patient 9	Patient 10	Patient 11	Patient 12	Gene Description
PVX_123660	0	0	0	0	0	265.52	0	72.0605	0	36.4623	10.3281	0	debranching enzyme-associated ribonuclease, putative
PVX_123665	0	73.8376	0	0	33.5698	0	0	0	0	0	42.0776	0	ribosome biogenesis protein TSR3, putative
PVX_123670	0	0	0	0	0	0	0	0	80.691	0	63.5669	0	hypothetical protein, conserved
PVX_123675	0	0	0	0	0	0	0	0	0	0	0	0	hypothetical protein, conserved
PVX_123680	0	0	0	0	0	0	0	0	0	0	0	0	unspecified product
PVX_123682	143.245	300.37	0	0	0	0	0	199.115	0	50.3073	28.5161	0	heterochromatin protein 1, putative (HP1)
PVX_123685	0	0	0	0	0	0	0	43.6801	5.95906	5.52845	9.39361	0	histone-lysine N-methyltransferase, H3 lysine-4 specific, putative (SET10)
PVX_123690	0	0	0	0	0	0	0	0	0	0	0	0	hypothetical protein, conserved
PVX_123695	0	0	0	0	0	1228.9	0	0	90.5963	0	47.5451	0	hypothetical protein, conserved
PVX_123700	0	0	0	0	0	0	0	0	0	0	0	0	hypothetical protein, conserved
PVX_123705	0	0	0	0	0	0	0	0	0	0	0	0	inner membrane complex protein 1h, putative (IMC1h)
PVX_123710	26.4245	0	0	0	0	0	40.3766	0	20.0204	27.857	15.7787	0	hypothetical protein, conserved
PVX_123715	82.1223	0	204.39	0	0	0	125.705	228.168	31.1217	0	130.776	133.086	hypothetical protein, conserved
PVX_123720	33.5338	0	0	107.129	10.6324	0	0	46.5627	0	23.5669	6.67467	0	FbpA domain protein, putative
PVX_123725	0	0	0	0	14.0944	0	0	0	16.8374	0	26.5375	0	hypothetical protein, conserved
PVX_123730	0	0	0	0	0	0	0	0	43.904	13.5734	15.3777	0	hypothetical protein, conserved
PVX_123735	0	47.6402	0	0	21.6498	0	0	0	0	0	0	0	hypothetical protein, conserved
PVX_123740	131.923	46.0588	0	210.917	83.7224	0	0	0	0	162.221	170.686	0	hypothetical protein, conserved
PVX_123745	58.4971	102.069	218.061	93.4281	46.3635	0	0	81.2216	22.1605	20.5558	128.077	0	endoplasmin, putative (GRP94)
PVX_123750	10.6076	7.40167	0	0	10.0842	0	0	0	8.0359	0	10.5565	0	transcription factor with AP2 domain(s), putative (ApiAP2)
PVX_123755	0	0	0	0	98.177	0	0	0	58.5279	162.627	92.1898	0	hypothetical protein, conserved
PVX_123760	8.81991	12.3083	0	0	0	0	0	0	10.0223	6.19924	33.3543	0	transcription factor with AP2 domain(s), putative (AP2-G)
PVX_123765	0	0	0	0	0	0	0	0	0	0	0	0	glideosome-associated protein 45, putative (GAP45)
PVX_123770	0	0	0	0	35.6374	0	0	0	0	0	0	0	hypothetical protein, conserved
PVX_123775	0	51.4056	0	0	0	0	0	0	0	51.7222	14.6526	0	hypothetical protein, conserved
PVX_123780	0	0	0	0	0	0	0	0	0	0	0	0	hypothetical protein, conserved
PVX_123785	0	0	198.902	255.658	0	0	0	222.05	0	56.1547	222.725	0	CAMP-dependent protein kinase regulatory subunit, putative
PVX_123790	0	47.3153	0	0	21.5021	0	0	0	0	23.8059	53.9505	0	nucleus export protein BRR6, putative
PVX_123795	24.7715	0	0	0	7.85248	0	0	34.3931	18.7678	8.70486	4.93055	0	DNA gyrase subunit A, putative (GyrA)
PVX_123800	73.9652	25.8081	45.9398	59.0486	0	0	0	51.3439	49.0318	32.4908	55.2075	0	phospholipid-transporting ATPase, putative
PVX_123805	6.54443	9.13257	0	0	2.07363	0	9.99353	0	9.91536	0	3.90774	0	hypothetical protein, conserved
PVX_123810	0	0	44.5939	0	5.68908	0	0	0	20.3982	6.3079	17.8636	0	hypothetical protein, conserved
PVX_123815	0	0	0	0	45.9835	0	221.835	0	109.67	50.7948	201.549	234.804	hypothetical protein, conserved
PVX_123820	0	43.5647	0	0	19.8108	0	0	0	47.2653	43.793	49.6413	0	mitochondrial carrier protein, putative
PVX_123825	336.595	117.672	0	539.698	160.614	0	0	233.996	127.634	177.295	201.027	273.402	50S ribosomal protein L24, putative
PVX_123830	0	0	0	0	26.5824	0	0	0	95.2007	88.2499	100.009	135.707	GTP cyclohydrolase I, putative (GCH1)
PVX_123835	0	0	0	0	0	0	0	0	0	0	0	0	hypothetical protein, conserved
PVX_123840	0	0	0	0	12.3453	0	0	0	0	27.3594	7.74906	0	hypothetical protein, conserved
PVX_123845	146.186	178.567	0	0	150.65	0	55.8608	152.242	318.449	205.47	269.151	59.1506	polyadenylate-binding protein, putative
PVX_123850	0	65.0864	0	0	14.7854	0	71.2765	0	52.9862	65.5189	46.3949	0	hypothetical protein, conserved
PVX_123855	213.141	0	0	0	0	0	0	0	0	0	106.071	0	histone chaperone ASF1, putative (ASF1)
PVX_123860	0	127.007	0	0	115.588	0	0	0	137.749	127.543	108.471	0	cytochrome c heme lyase, putative
PVX_123865	0	17.3024	0	0	0	0	75.7605	0	9.3917	0	19.7386	40.1128	hypothetical protein, conserved
PVX_123870	0	0	0	214.561	21.2921	0	0	93.1994	25.4256	117.87	40.0685	0	hypothetical protein, conserved
PVX_123875	682.233	0	0	0	0	0	0	0	519.208	0	135.909	0	splicing factor 3B subunit 6, putative (SF3B6)
PVX_123880	0	0	0	0	0	0	0	0	14.3928	13.3522	11.3439	0	hypothetical protein, conserved
PVX_123885	19.3677	0	48.1183	0	12.2774	0	29.5871	0	7.33657	27.2245	53.9692	0	isoleucine--tRNA ligase, putative
PVX_123890	24.8955	52.1239	0	0	15.7836	0	0	0	18.8618	8.74845	44.5971	0	hypothetical protein, conserved
PVX_123895	0	83.2937	0	0	0	0	0	0	45.186	0	0	0	hypothetical protein, conserved
PVX_123900	0	155.008	0	0	0	0	169.957	0	42.0471	38.9646	132.493	0	hypothetical protein, conserved
PVX_123905	140.961	49.2174	0	0	0	0	107.845	97.9002	53.4156	123.808	56.1176	114.182	hypothetical protein, conserved
PVX_123910	92.5553	43.0662	0	0	39.1254	0	47.148	42.8375	35.0631	21.6823	30.7041	0	hypothetical protein, conserved
PVX_123915	0	15.0575	107.218	0	6.83909	0	0	0	16.3467	22.7466	25.7674	0	hypothetical protein, conserved
PVX_123920	138.588	19.3448	137.759	0	43.9351	0	84.7083	38.4846	73.5011	107.14	154.475	44.8499	ubiquitin-activating enzyme E1, putative
PVX_123925	0	38.338	0	0	0	0	0	0	0	0	0	0	hypothetical protein, conserved
PVX_123930	0	0	0	0	0	0	0	0	13.1424	24.3798	20.7149	0	hypothetical protein, conserved
PVX_123935	221.585	77.4077	0	0	140.78	0	169.746	153.955	587.93	466.996	485.205	0	haloacid dehalogenase-like hydrolase, putative (HAD3)
PVX_123940	0	0	0	0	13.2265	0	0	0	0	0	0	0	hypothetical protein
PVX_123945	81.7473	171.28	0	0	51.8996	0	125.13	340.687	247.836	143.591	178.995	0	haloacid dehalogenase-like hydrolase, putative (HAD2)
PVX_123950	37.8447	0	0	0	12.0005	0	0	0	14.3375	13.298	37.6639	0	hypothetical protein, conserved
PVX_123955	0	0	0	0	0	0	0	0	19.3628	26.9423	10.1737	41.3509	hypothetical protein, conserved
PVX_123960	0	0	0	484.743	48.0894	0	232.005	630.705	516.058	106.221	331.173	245.563	proliferating cell nuclear antigen, putative

Gene ID / Patient	Patient 1	Patient 2	Patient 3	Patient 4	Patient 5	Patient 6	Patient 7	Patient 8	Patient 9	Patient 10	Patient 11	Patient 12	Gene Description
PVX_123965	0	0	0	0	0	0	131.192	0	48.7679	90.4583	17.0809	0	WD domain, G-beta repeat domain containing protein
PVX_123970	87.6736	0	0	280.515	0	0	0	243.604	99.6801	92.3979	104.713	0	ataxin-3, putative (ATX3)
PVX_123975	60.9729	0	0	0	0	0	0	0	0	0	12.1367	0	hypothetical protein, conserved
PVX_123980	0	0	0	0	0	0	0	0	0	0	0	0	hypothetical protein, conserved
PVX_123985	0	96.0772	0	0	14.5501	0	70.1421	127.417	121.669	96.717	82.1836	74.2704	ATP-dependent RNA helicase, putative
PVX_123990	0	10.6193	0	0	4.82293	0	0	0	0	0	6.05794	0	potassium channel, putative
PVX_123995	0	0	0	0	0	0	0	42.6024	0	0	0	0	hypothetical protein, conserved
PVX_124000	0	0	0	0	0	0	0	0	13.3546	12.3867	14.0329	0	hypothetical protein, conserved
PVX_124005	11.2616	0	0	0	7.13743	0	17.1994	15.6337	4.26572	0	11.2074	0	hypothetical protein, conserved
PVX_124010	0	0	0	0	0	0	0	0	52.7946	12.2421	34.6726	0	hypothetical protein, conserved
PVX_124015	0	23.5737	83.9447	0	21.4173	0	51.6189	0	12.7951	0	47.0577	0	hypothetical protein, conserved
PVX_124020	0	40.2225	0	0	18.2705	0	44.0331	0	43.6643	10.1257	45.8837	46.6276	histone acetyltransferase, putative
PVX_124025	0	0	0	0	0	0	0	0	54.3143	0	14.2654	0	RNA pseudouridylate synthase, putative
PVX_124030	0	0	0	0	102.805	0	0	224.685	0	340.513	64.3462	0	hypothetical protein, conserved
PVX_124035	75.0937	0	0	0	71.5014	0	0	104.312	85.3701	52.7634	59.7909	0	hypothetical protein, conserved
PVX_124040	0	0	0	0	12.9902	0	0	0	0	0	24.4603	0	hypothetical protein, conserved
PVX_124045	32.0334	0	0	0	0	0	0	0	12.1354	0	25.5041	0	NIMA-related protein kinase (Pfnek-1), putative
PVX_124050	0	42.2645	0	96.7182	47.996	0	0	42.0401	57.3509	85.1158	42.1858	0	hypothetical protein, conserved
PVX_124055	174.322	60.8787	0	0	27.6721	0	0	0	0	30.6194	104.101	0	hypothetical protein, conserved
PVX_124060	0	0	0	0	0	0	0	0	0	0	0	0	merozoite surface protein-9 precursor
PVX_124065	0	40.7741	0	0	9.26059	0	0	40.5578	22.1315	0	46.5128	0	hypothetical protein, conserved
PVX_124070	71.8513	7.16224	0	0	3.25265	0	0	28.4986	19.4399	18.0363	28.6022	33.2017	WD domain, G-beta repeat domain containing protein
PVX_124075	40.4078	0	0	0	12.814	0	0	0	15.3089	14.1984	16.086	0	hypothetical protein, conserved
PVX_124080	17.9103	49.994	0	0	5.67655	0	0	0	0	6.29401	17.8243	0	hypothetical protein, conserved
PVX_124085	107.403	42.827	76.2308	48.9915	38.9011	78.429	70.3085	21.3008	98.8028	53.9197	48.8624	99.2733	multidrug resistance associated protein, putative,ABC transporter, putative
PVX_124090	65.3295	0	0	0	82.8529	0	99.843	90.7112	37.124	91.8251	117.03	52.8622	hypothetical protein, conserved
PVX_124095	815.88	285.359	509.398	654.753	519.543	0	0	567.386	1083.08	787.731	1137.02	663.374	macrophage migration inhibitory factor, putative (MIF)
PVX_124100	250.024	130.932	0	0	39.665	0	191.237	694.53	331.584	285.491	311.047	101.24	T-complex protein 1, gamma subunit, putative
PVX_124105	0	0	0	0	0	0	0	42.8523	23.3835	43.3796	86.0011	99.8858	hypothetical protein, conserved
PVX_124110	0	0	0	0	0	0	0	0	0	110.667	62.799	0	hypothetical protein
PVX_124115	0	0	0	0	0	0	0	62.9337	0	15.9236	9.02053	0	hypothetical protein, conserved
PVX_124120	0	0	0	0	0	0	0	0	0	0	6.49889	0	myosin D, putative
PVX_124130	0	0	0	0	0	0	0	0	0	0	33.3933	67.8931	hypothetical protein, conserved
PVX_124140	0	0	0	0	11.1873	0	0	24.5023	20.0562	24.8087	17.5641	0	hypothetical protein, conserved
PVX_124145	0	0	0	0	0	0	0	0	0	0	0	0	thioredoxin-like associated protein 1, putative (TLAP1)
PVX_124150	32.9898	0	0	105.39	52.2988	0	0	91.6144	24.9957	34.7771	98.4961	0	hypothetical protein, conserved
PVX_124155	0	0	0	0	0	0	0	0	0	0	0	0	hypothetical protein, conserved
PVX_124160	249.37	87.1295	0	399.349	79.2432	0	382.231	0	47.2653	131.379	148.924	0	ATP-dependent protease subunit ClpQ, putative
PVX_124165	62.6811	43.7665	0	0	0	0	0	174.119	23.751	44.0447	87.3372	0	hypothetical protein, conserved
PVX_124170	192.924	134.766	0	0	61.2639	0	0	0	36.5596	135.541	96.0087	0	sun-family protein, putative
PVX_124175	42.8453	29.9087	106.518	0	13.5878	0	0	0	97.3956	75.2737	34.1127	0	protein transport protein SEC13, putative (SEC13)
PVX_124180	0	0	0	180.524	53.7454	0	86.3706	0	0	59.5237	22.4812	0	conserved Plasmodium protein, unknown function
PVX_124185	0	0	0	0	0	0	57.0504	0	14.1402	0	0	0	serine/threonine protein kinase RIO1, putative (RIO1)
PVX_124190	0	0	0	0	0	0	0	0	13.2606	0	13.9341	0	hypothetical protein, conserved
PVX_124195	0	152.92	0	0	139.054	0	167.663	0	248.887	76.8814	21.785	177.489	ras-related protein Rab-2, putative (RAB2)
PVX_124200	76.637	0	0	0	24.3246	0	0	0	29.0419	0	0	0	hypothetical protein, conserved
PVX_124700	0	0	0	0	0	0	0	0	0	0	0	0	VIR protein, pseudogene,PIR protein
PVX_124705	0	0	0	0	0	0	0	0	0	26.5824	0	0	variable surface protein Vir12-related,PIR protein
PVX_124708	0	0	0	0	0	0	0	0	20.8444	38.6582	10.9504	0	VIR protein,PIR protein
PVX_124710	0	0	0	0	0	0	0	0	0	0	0	0	variable surface protein Vir4-related,PIR protein
PVX_124712	0	0	0	0	0	0	0	0	0	0	0	0	VIR protein, pseudogene,PIR protein, pseudogene
PVX_124715	0	0	0	0	0	0	0	0	0	0	0	0	variable surface protein Vir5-related,PIR protein
PVX_124720	0	0	0	0	0	0	0	0	0	0	11.2833	0	variable surface protein Vir12-related,PIR protein
PVX_124725	0	0	0	0	0	0	0	0	0	0	0	0	VIR protein,PIR protein
PVX_125726	0	0	0	0	0	0	0	0	0	0	0	0	hypothetical protein
PVX_125728	0	0	0	0	0	0	0	0	0	0	0	0	tryptophan-rich antigen (Pv-fam-a)
PVX_125730	0	58.7036	0	0	0	0	0	0	31.8529	29.5271	66.9233	136.218	tryptophan-rich antigen (Pv-fam-a)
PVX_125735	0	158.524	0	0	24.0155	0	0	105.107	28.6734	0	15.0615	0	hypothetical protein
PVX_125738	0	0	0	0	0	0	0	0	0	0	0	0	reticulocyte binding protein 1 precursor, putative
PVX_127260	0	0	0	0	0	0	0	0	0	0	0	0	variable surface protein Vir16, putative, truncated
PVX_128260	0	0	0	0	0	0	0	0	0	0	29.5571	0	Pvstp1, truncated, putative
PVX_129260	0	0	0	0	0	0	3467.26	0	0	1420.66	0	0	variable surface protein Vir12, putative

Gene ID / Patient	Patient 1	Patient 2	Patient 3	Patient 4	Patient 5	Patient 6	Patient 7	Patient 8	Patient 9	Patient 10	Patient 11	Patient 12	Gene Description
PVX_130260	0	0	0	0	0	0	552.656	0	0	247.906	0	0	variable surface protein Vir12, truncated, putative
PVX_131260	0	98.3878	0	0	0	0	0	0	106.735	0	28.0226	0	variable surface protein Vir12, truncated, putative
PVX_132260	0	0	0	0	0	0	0	0	0	0	0	0	Pvstp1, truncated, putative
PVX_133260	0	0	0	0	0	0	0	0	0	0	29.5571	0	variable surface protein Vir12, putative, truncated
PVX_134260	0	0	0	0	0	0	0	0	0	0	0	0	variable surface protein Vir12, putative, truncated
PVX_135260	0	0	0	0	0	0	0	0	0	0	0	0	hypothetical protein, truncated
PVX_136260	134.878	94.4287	0	433.769	43.0139	0	415.402	0	51.1803	47.3386	53.7098	0	DNA-directed RNA polymerase 2 subunit, putative, truncated
PVX_137260	0	0	0	0	0	0	0	0	0	0	0	0	dihydrolipoamide S-acetyltransferase, truncated, putative
PVX_138260	1297.4	958.259	461.667	593.403	388.592	190.026	567.937	1313.51	1531.51	1080.7	988.785	541.095	elongation factor 1 alpha, pseudogene, putative
PVX_139260	0	0	0	0	0	0	0	0	0	0	0	0	variable surface protein Vir3, truncated,putative
PVX_140260	0	0	0	186.385	0	0	0	0	0	20.4447	11.5869	0	helicase, putative
PVX_141260	0	0	0	0	0	0	0	0	0	0	0	0	variable surface protein Vir17, putative
PVX_142260	0	0	0	0	0	0	0	0	0	0	0	0	cytochrome c oxidase subunit III, putative
PVX_143260	0	0	0	0	0	0	0	0	0	0	0	0	variable surface protein Vir35, putative
PVX_144260	0	0	0	0	0	0	0	0	0	0	0	0	variable surface protein Vir17, putative, pseudogene
PVX_145260	0	0	0	0	0	0	0	0	0	0	0	0	variable surface protein Vir30, putative
PVX_146260	0	0	0	0	0	0	0	0	0	0	0	0	variable surface protein Vir11, putative
PVX_147260	810.754	142.152	1018.18	0	324.281	1048.31	626.976	0	384.917	284.361	322.909	662.977	ribosomal protein L21e, putative, truncated
PVX_148260	0	0	0	0	0	0	0	0	0	0	0	0	variable surface protein Vir25, truncated, putative
PVX_149260	0	0	0	0	0	0	0	0	0	0	0	0	variable surface prtoein Vir14, truncated, putative
PVX_150260	0	0	0	0	0	0	0	0	0	0	20.1807	0	variable surface protein Vir27, truncated, putative
PVX_151260	0	0	0	0	0	0	0	0	0	0	0	0	Pvstp1, pseudogene, putative
PVX_152260	0	0	0	0	0	0	0	0	0	0	0	0	variable surface protein Vir17, pseudogene, putative
PVX_153260	0	0	0	0	0	0	0	0	0	0	0	0	variable surface protein Vir17, truncated, putative
PVX_154260	0	0	0	0	0	0	0	0	0	0	0	0	variable surface protein Vir23, pseudogene
PVX_155260	0	0	0	0	0	0	0	0	0	0	0	0	variable surface rptoein Vir3, pseudogene, putative
PVX_156260	0	0	0	0	0	0	0	0	0	0	0	0	variable surface proteinVir5, pseudogene, putative
PVX_157260	0	0	0	0	0	0	0	0	0	0	0	0	variable surface protein Vir12, truncated, putative
PVX_158260	0	0	0	0	0	0	0	0	0	0	0	0	variable surface protein Vir12, truncated, putative
PVX_159260	0	0	0	0	0	0	0	0	0	0	0	0	variable surface protein Vir14, pseudogene, putative
PVX_160260	0	0	0	0	0	0	0	0	0	0	0	0	variable surface protein Vir3, truncated, putative
PVX_161260	0	0	0	0	0	0	0	0	0	0	0	0	variable surface protein Vir3, truncated, putative
PVX_162260	0	0	0	0	0	0	0	0	0	0	0	0	variable surface protein Vir5, pseudogene, putative
PVX_163260	0	0	0	0	0	0	0	0	0	0	0	0	variable surface protein Vir35, pseudogene, putative
PVX_163265	0	0	0	0	0	0	0	0	0	0	0	0	variable surface protein Vir30, pseudogene, putative
PVX_164265	0	0	0	0	0	0	0	0	0	0	0	0	variable surface protein Vir21, putative
PVX_165265	0	0	0	0	0	0	0	0	0	0	0	0	Pvstp1, truncated, putative
PVX_166265	0	0	0	0	0	0	0	0	0	0	0	0	Pvstp1, truncated, putative
PVX_167265	0	0	0	0	0	0	0	0	0	0	0	0	variable surface protein Vir15, truncated, putative
PVX_168265	0	0	0	0	0	0	0	0	0	0	0	0	variable surface protein Vir16, pseudogene, putative
PVX_168270	0	0	0	0	0	0	0	0	0	0	0	0	variable surface protein Vir15, pseudogene, putative
PVX_169270	0	0	0	0	0	0	0	0	0	0	0	0	variable surface protein Vir14, putative
PVX_170270	0	78.9742	0	0	0	0	0	0	0	0	0	0	variable surface protein Vir12, truncated, putative
PVX_171270	0	0	0	0	0	0	0	0	0	0	0	0	variable surface protein Vir17, pseudogene, putative
PVX_172270	0	0	0	0	0	0	0	0	0	0	0	0	variable surface protein Vir22, truncated, putative
PVX_173270	0	0	0	0	0	0	0	0	0	0	0	0	variable surface protein Vir23, putative
PVX_174270	0	0	0	0	0	0	0	0	0	0	0	0	variable surface protein Vir6-like, pseudogene
PVX_175270	0	56.9371	0	0	0	0	0	0	0	28.6398	0	0	variable surface protein Vir14, truncated, putative
PVX_176270	0	0	0	0	0	0	0	0	0	0	0	0	variable surface protein Vir8
PVX_176275	0	0	0	0	0	0	0	0	0	0	0	0	variable surface protein Vir9, pseudogene, putative
PVX_177275	0	79.8836	0	0	0	0	0	0	0	0	0	0	variable surface protein Vir7, putative
PVX_178275	0	0	0	0	0	0	0	0	0	0	0	0	variable surface protein Vir11, putative
PVX_179275	0	0	0	0	0	0	0	0	0	0	0	0	variable surface protein Vir C1-29, pseudogene, putative
PVX_180275	0	0	0	0	0	0	0	0	0	0	0	0	variable surface protein Vir26-like, truncated
PVX_181275	0	0	0	0	0	0	0	0	0	0	0	0	variable surface protein Vir17, pseudogene, putative
PVX_182275	0	0	0	0	0	0	0	0	0	0	0	0	variable surface protein Vir11, truncated, putative
PVX_183275	0	0	0	0	0	0	0	0	0	32.4518	0	0	hypothetical protein
PVX_183280	0	55.3717	0	0	0	0	0	0	0	27.8536	47.3463	0	variable surface protein Vir12-related
PVX_184280	0	0	0	0	0	0	0	0	0	0	0	0	variable surface protein Vir5, pseudogene, putative
PVX_184285	0	0	0	0	0	0	0	0	0	0	0	0	variable surface protein Vir4, putative
PVX_185285	0	0	0	0	0	0	0	0	0	0	0	0	variable surface protein Vir2, truncated, putative
PVX_186285	0	0	0	0	0	0	0	0	0	0	0	0	variable surface protein Vir3, truncated,pseudogene

Gene ID / Patient	Patient 1	Patient 2	Patient 3	Patient 4	Patient 5	Patient 6	Patient 7	Patient 8	Patient 9	Patient 10	Patient 11	Patient 12	Gene Description
PVX_186290	0	0	0	0	0	0	0	0	0	0	0	0	variable surface protein Vir15-related, truncated
PVX_187290	0	0	0	0	0	0	0	0	0	0	0	0	circumsporozoite protein, truncated, putative
PVX_188290	0	0	0	0	0	0	0	0	0	0	0	0	circumsporozoite protein, truncated, putative
PVX_191290	33.7071	0	0	107.99	0	0	0	0	29.8166	0	6.71008	0	adrenodoxin reductase, putative
PVX_192290	36.4531	0	0	116.824	0	0	0	0	13.8206	0	7.25682	0	adrenodoxin reductase, putative
PVX_193290	0	30.0062	0	0	0	0	0	0	0	0	0	0	hypothetical protein
PVX_195290	17.6541	36.9758	43.9024	0	0	0	0	24.5179	60.2009	37.2173	15.8131	42.8796	vivapain-1
PVX_196290	30.7298	64.3947	0	0	0	0	0	0	11.6464	10.795	61.1703	0	developmentally regulated GTP-binding protein 1, putative
PVX_198290	0	0	0	0	0	0	0	89.2488	0	11.2878	6.39515	0	RNA helicase, putative
PVX_200290	0	17.3774	0	0	15.8045	0	38.1165	34.5603	9.42678	8.73429	25.9893	40.348	DNA polymerase epsilon subunit B
PVX_201290	0	20.0247	0	0	18.2161	0	43.9389	39.8229	10.8616	10.0615	22.8129	46.508	DNA polymerase epsilon subunit B, truncated, putative
PVX_202290	16.3577	0	0	52.3043	10.381	0	0	0	14.4628	0	16.2801	0	adrenodoxin reductase, putative
PVX_203290	0	32.6691	232.878	0	0	0	0	0	0	32.8586	18.6196	0	PST-A protein, truncated
PVX_206290	0	17.3532	0	0	15.7825	0	38.0634	34.5123	9.41369	8.72217	25.9532	40.2918	DNA polymerase epsilon subunit B, putative
PVX_208290	0	15.3672	0	0	13.9739	0	33.698	30.5638	8.33703	7.72591	22.9867	35.6728	DNA polymerase epsilon subunit B, putative
PVX_209290	0	21.93	0	0	0	0	0	0	14.2832	0	2.5014	0	cleavage and polyadenylation specifity factor protein, putative
PVX_210290	0	0	0	0	0	0	0	0	0	8.95818	30.464	0	citrate synthase, mitochondrial precursor, putative, truncated
PVX_211290	60.163	0	0	0	0	0	0	0	9.12234	0	5.98821	0	citrate synthase, mitochondrial precursor, putative
PVX_212290	0	0	0	0	0	0	0	0	0	0	0	0	ribose 5-phosphate epimerase, putative
PVX_213290	0	0	0	0	0	0	0	0	0	0	0	0	hypothetical protein
PVX_214290	0	0	0	0	0	0	0	0	0	0	9.02839	0	hypothetical protein
PVX_215290	0	0	0	0	0	0	0	0	0	0	9.02839	0	hypothetical protein
PVX_216290	35.2505	0	0	0	0	0	0	0	13.3674	12.3778	7.01769	0	hypothetical protein, conserved
PVX_217290	42.8447	0	0	0	0	0	0	0	16.2515	15.0416	8.52998	0	hypothetical protein
PVX_218290	35.2505	0	0	0	0	0	0	0	13.3674	12.3778	7.01769	0	hypothetical protein
PVX_220290	395.326	345.641	987.117	0	220.237	507.963	607.367	1374.52	1087.03	728.698	708.337	482.06	cyclophilin, putative
PVX_221290	0	31.3155	0	0	0	0	0	0	0	0	8.91989	0	cleavage and polyadenylation specifity factor protein, putative, truncated
PVX_222290	28.9691	60.7011	0	0	0	0	0	0	10.9787	10.1767	57.6651	0	developmentally regulated GTP-binding protein 1, putative
PVX_223290	0	0	0	0	0	0	0	0	0	9.39992	31.9675	0	citrate synthase, mitochondrial precursor, putative, truncated
PVX_224290	78.3988	0	0	0	0	0	0	0	11.8903	0	7.80365	0	citrate synthase, mitochondrial precursor, putative
PVX_225290	0	0	0	0	0	0	0	0	0	10.1426	34.4955	0	citrate synthase, mitochondrial precursor, putative, truncated
PVX_226290	38.001	0	0	0	0	0	0	0	11.5264	0	7.56502	123.41	citrate synthase, mitochondrial precursor, putative
PVX_227290	0	0	0	0	0	0	0	0	0	0	0	0	hypothetical protein
PVX_228290	21.1717	0	0	0	0	0	0	0	6.41883	5.95011	28.6577	0	citrate synthase, mitochondrial precursor, putative
PVX_230290	0	0	0	0	0	0	0	0	0	0	18.3768	0	hypothetical protein, conserved, truncated
PVX_231290	0	0	0	0	0	0	157.441	0	77.7176	71.9395	61.191	0	hypothetical protein
PVX_235290	0	0	0	0	0	0	0	0	0	0	0	0	phosphatidylethanolamine-binding protein, putative
PVX_237290	35.2505	0	0	0	0	0	0	0	13.3674	12.3778	7.01769	0	hypothetical protein
PVX_238290	0	0	0	0	0	0	0	0	0	69.0287	39.2386	0	hypothetical protein
PVX_239290	0	0	0	0	0	0	0	0	97.8038	0	8.55982	0	vivapain-1
PVX_240290	38.1591	79.9859	95.0588	0	0	0	0	53.0272	43.3928	80.4198	15.1922	0	vivapain-1
PVX_241290	0	0	0	0	0	0	0	0	0	0	0	0	variable surface protein Vir12-like
PVX_241295	0	0	0	0	0	0	0	0	0	0	0	0	variable surface protein Vir18, putative
PVX_242295	0	0	0	0	0	0	0	0	0	0	0	0	Pvstp1, truncated, putative
PVX_243295	0	0	0	0	0	0	0	0	0	0	0	0	cytochrome c oxidase subunit III, pseudogene
PVX_244295	0	0	0	0	0	0	0	0	0	0	0	0	cytochrome c oxidase subunit III, truncated, putative
PVX_245295	0	0	0	0	0	0	0	0	0	0	0	0	cytochrome c oxidase subunit III, pseudogene
PVX_246295	0	0	0	0	0	0	0	0	24.5572	15.1679	34.388	0	hypothetical protein, truncated
PVX_248300	0	0	0	0	0	0	0	0	0	0	0	0	variable surface protein Vir29, pseudogene, putative
PVX_249300	0	0	0	0	0	0	0	0	0	17.2776	9.79085	0	hypothetical protein
PVX_250300	53.0327	0	0	0	0	0	81.2396	0	0	0	31.6704	86.0016	ADP/ATP transporter on adenylate translocase, putative
PVX_251300	0	41.8252	0	0	0	0	0	0	0	0	0	0	hypothetical protein
PVX_252300	94.6262	165.247	0	0	30.0476	0	72.4515	197.205	125.522	99.7217	103.599	0	casein kinase II, alpha subunit, putative
PVX_253300	0	0	0	0	15.4159	0	0	67.4483	18.3989	68.2113	9.66337	157.408	syntaxin, putative
PVX_254300	0	36.3598	0	0	16.5304	0	0	0	39.4533	36.5638	41.4413	0	mitochondrial carrier protein, putative
