# Supplementary material for: Plasmodium vivax readiness to transmit: implication for malaria eradication
Source: BMC Syst Biol. 2019 Jan 11;13:5. doi: 10.1186/s12918-018-0669-4 (PMC6330404; doi:10.1186/s12918-018-0669-4)
Supplement: Supplementary file 3 — Supplemental table s2. Table S2. (PDF 753 kb) [file 12918_2018_669_MOESM3_ESM.pdf]

S2 Table

GeneID	Pvalue	BackGroundPre	BackGroundDx
PlvioMp1	1	0	0
PlvioMp2	1	0	0
PlvioMp3	0.359317338	0	0.294451006
PVX_000000	1	0	0
PVX_000005	0.007054102	0	2.083283255
PVX_000010	1.03E-05	0	7.257606641
PVX_000015	0.078805602	0	1.24853291
PVX_000020	1	0	0
PVX_000025	1	0	0
PVX_000525	0.359317338	0	0.189773385
PVX_000530	0.036687443	0	0.55458053
PVX_000535	0.016473156	0	1.624619874
PVX_000540	0.359317338	0	0.179253224
PVX_000545	0.001084333	0	3.106887107
PVX_000550	0.036687443	0	1.121133922
PVX_000555	0.078805602	0	1.08095978
PVX_000560	0.036687443	0	1.061933816
PVX_000565	0.002855585	0	2.228957738
PVX_000570	0.359317338	0	0.347601856
PVX_000575	0.002855585	0	2.288426204
PVX_000580	0.002855585	0	2.936251361
PVX_000585	0.016473156	0	1.749686564
PVX_000590	3.76E-05	0	5.305878424
PVX_000595	0.166224591	0	0.463616188
PVX_000600	0.000383392	0	3.120831128
PVX_000604	0.000383392	0	3.653425844
PVX_000606	0.166224591	0	0.770870781
PVX_000610	3.76E-05	0	4.910105591
PVX_000615	0.036687443	0	1.29249639
PVX_000620	0.000383392	0	3.027364591
PVX_000625	0.001084333	0	2.491165189
PVX_000630	0.002855585	0	2.802119348
PVX_000635	0.166224591	0	0.64389557
PVX_000640	0.016473156	0	1.864254395
PVX_000645	0.359317338	0	0.25180039
PVX_000650	0.000383392	0	2.564190592
PVX_000660	0.036687443	0	0.614144898
PVX_000670	0.078805602	0	0.774528265

GeneID	Pvalue	BackGroundPre	BackGroundDx
PVX_000675	1	0	0
PVX_000677	1	0	0
PVX_000678	1	0	0
PVX_000680	0.036687443	0	1.013930035
PVX_000685	0.036687443	0	1.469892729
PVX_000690	0.036687443	0	1.085083317
PVX_000695	0.166224591	0	0.683013674
PVX_000700	0.166224591	0	0.574261563
PVX_000705	0.002855585	0	2.186469025
PVX_000710	0.036687443	0	0.826264759
PVX_000715	0.007054102	0	1.684346355
PVX_000720	0.001084333	0	3.275800336
PVX_000725	0.016473156	0	1.871770847
PVX_000730	0.002855585	0	2.585196306
PVX_000735	0.002855585	0	1.904912843
PVX_000737	1	0	0
PVX_000738	1	0	0
PVX_000745	0.007054102	0	1.988503394
PVX_000750	1	0	0
PVX_000755	0.001084333	0	3.224349479
PVX_000760	1	0	0
PVX_000765	0.001084333	0	2.548037851
PVX_000770	0.036687443	0	1.282617701
PVX_000775	0.002855585	0	2.685798755
PVX_000780	0.001084333	0	2.590665992
PVX_000785	0.007054102	0	1.903313429
PVX_000790	0.016473156	0	1.420813571
PVX_000795	0.016473156	0	1.197477494
PVX_000800	0.002855585	0	2.298660614
PVX_000805	0.166224591	0	0.560571095
PVX_000810	1	0	0
PVX_000815	1	0	0
PVX_000820	0.036687443	0	1.087612963
PVX_000825	0.036687443	0	1.366489386
PVX_000830	0.036687443	0	1.407871813
PVX_000835	0.166224591	0	0.625055062
PVX_000840	0.078805602	0	0.690649668
PVX_000845	0.359317338	0	0.566468337
PVX_000850	0.016473156	0	1.61743597
PVX_000855	0.359317338	0	0.180323649
PVX_000860	0.359317338	0	0.136841558

GeneID	Pvalue	BackGroundPre	BackGroundDx
PVX_000865	1	0	0
PVX_000870	0.078805602	0	0.934266053
PVX_000875	1	0	0
PVX_000880	0.007054102	0	1.951513464
PVX_000885	0.001084333	0	3.081489466
PVX_000890	0.001084333	0	3.03823361
PVX_000895	0.016473156	0	1.633816426
PVX_000900	0.166224591	0	0.506579383
PVX_000905	0.036687443	0	1.623083508
PVX_000910	0.359317338	0	0.230994355
PVX_000915	0.166224591	0	0.916741746
PVX_000920	0.016473156	0	1.584997651
PVX_000925	0.001084333	0	3.287774609
PVX_000930	0.078805602	0	0.974232461
PVX_000935	0.000125309	0	4.084212927
PVX_000940	0.001084333	0	2.865565577
PVX_000945	0.359317338	0	0.409487725
PVX_000950	0.007054102	0	2.289529178
PVX_000955	3.76E-05	0	3.375892491
PVX_000960	0.036687443	0	1.642497961
PVX_000965	0.016473156	0	1.772665011
PVX_000970	0.000125309	0	3.03566976
PVX_000975	0.007054102	0	1.385783531
PVX_000980	1	0	0
PVX_000985	3.76E-05	0	3.5168559
PVX_000990	0.007054102	0	2.065979027
PVX_000995	1	0	0
PVX_001000	0.359317338	0	0.198877962
PVX_001005	0.359317338	0	0.223460687
PVX_001010	1	0	0
PVX_001015	1	0	0
PVX_001020	1	0	0
PVX_001025	1	0	0
PVX_001030	0.001084333	0	2.931884889
PVX_001035	0.016473156	0	2.001428538
PVX_001040	0.359317338	0	0.082987674
PVX_001045	0.359317338	0	0.364136054
PVX_001050	0.016473156	0	1.172973129
PVX_001055	0.166224591	0	0.537040276
PVX_001060	0.016473156	0	1.601557769
PVX_001065	0.166224591	0	0.664627823

GeneID	Pvalue	BackGroundPre	BackGroundDx
PVX_001070	1	0	0
PVX_001075	1	0	0
PVX_001080	0.016473156	0	0.920696511
PVX_001085	0.166224591	0	0.813128636
PVX_001090	0.007054102	0	1.866470814
PVX_001095	0.036687443	0	1.286330259
PVX_001097	1	0	0
PVX_001100	1	0	0
PVX_001105	0.166224591	0	0.536625831
PVX_001110	1	0	0
PVX_001610	0.359317338	0	0.247467845
PVX_001615	1	0	0
PVX_001620	1	0	0
PVX_001625	0.359317338	0	0.231567237
PVX_001630	0.166224591	0	0.506201227
PVX_001635	0.359317338	0	0.198626719
PVX_001640	0.359317338	0	0.259105897
PVX_001645	0.078805602	0	0.732620485
PVX_001650	0.000383392	0	3.201706754
PVX_001655	0.166224591	0	0.777726414
PVX_001660	0.359317338	0	0.456749419
PVX_001670	1.03E-05	0	5.807207768
PVX_001675	0.359317338	0	0.262984373
PVX_001680	1	0	0
PVX_001685	1.03E-05	0	5.424831532
PVX_001690	0.359317338	0	0.364811559
PVX_001695	1	0	0
PVX_001700	0.359317338	0	0.303063731
PVX_001705	0.166224591	0	0.542117658
PVX_001710	0.016473156	0	2.038998749
PVX_001715	1	0	0
PVX_001720	0.001084333	0	2.804385019
PVX_001725	0.359317338	0	0.279711224
PVX_001730	0.166224591	0	0.684937561
PVX_001735	0.036687443	0	1.358145877
PVX_001740	0.359317338	0	0.294182618
PVX_001745	0.359317338	0	0.49032351
PVX_001750	0.001084333	0	2.911718697
PVX_001752	1	0	0
PVX_001755	0.007054102	0	1.606292267
PVX_001760	0.001084333	0	3.0710045

GeneID	Pvalue	BackGroundPre	BackGroundDx
PVX_001765	0.166224591	0	0.655701136
PVX_001770	0.166224591	0	0.893361333
PVX_001775	0.016473156	0	1.100016013
PVX_001780	0.002855585	0	2.3112826
PVX_001782	0.359317338	0	0.327105083
PVX_001783	0.166224591	0	0.828369528
PVX_001785	1	0	0
PVX_001790	0.002855585	0	1.917767952
PVX_001795	0.078805602	0	1.00629624
PVX_001800	0.078805602	0	0.95992301
PVX_001805	0.001084333	0	2.433030086
PVX_001810	0.000125309	0	2.64748398
PVX_001820	0.166224591	0	0.520189908
PVX_001825	1	0	0
PVX_001830	1	0	0
PVX_001835	0.001084333	0	4.246726787
PVX_001840	0.078805602	0	0.70332244
PVX_001845	0.001084333	0	2.458048409
PVX_001850	0.036687443	0	1.230729728
PVX_001855	0.166224591	0	0.550635031
PVX_001860	0.166224591	0	0.950060272
PVX_001865	0.002855585	0	2.439709902
PVX_001868	1	0	0
PVX_001872	0.359317338	0	0.218127876
PVX_001875	1	0	0
PVX_001880	0.000383392	0	3.569322137
PVX_001885	0.036687443	0	1.378054477
PVX_001890	0.016473156	0	1.336708707
PVX_001895	0.016473156	0	1.29818783
PVX_001900	1	0	0
PVX_001905	0.016473156	0	2.51314681
PVX_001910	0.166224591	0	0.839012603
PVX_001920	0.001084333	0	2.519237181
PVX_001925	0.078805602	0	1.033402563
PVX_001930	0.002855585	0	2.351644819
PVX_001935	0.002855585	0	2.422402832
PVX_001940	0.007054102	0	2.571659516
PVX_001945	0.000383392	0	4.133366376
PVX_001950	0.002855585	0	1.32002964
PVX_001955	0.007054102	0	1.541598346
PVX_001960	0.016473156	0	1.464398458

GeneID	Pvalue	BackGroundPre	BackGroundDx
PVX_001965	0.007054102	0	1.527515469
PVX_001970	0.002855585	0	3.031238966
PVX_001975	0.002855585	0	2.309797594
PVX_001980	0.359317338	0	0.427124381
PVX_002480	1	0	0
PVX_002485	0.002855585	0	2.833464835
PVX_002490	0.359317338	0	0.249466249
PVX_002495	1	0	0
PVX_002500	1	0	0
PVX_002505	0.166224591	0	0.530101828
PVX_002507	0.359317338	0	0.310636143
PVX_002510	1	0	0
PVX_002512	0.078805602	0	1.045816278
PVX_002515	0.166224591	0	0.584760488
PVX_002520	1	0	0
PVX_002525	0.359317338	0	0.28706636
PVX_002530	0.166224591	0	0.669515137
PVX_002535	0.016473156	0	1.915632873
PVX_002550	0.000125309	0	2.800561999
PVX_002555	0.000383392	0	3.752641226
PVX_002560	1	0	0
PVX_002565	0.359317338	0	0.274354699
PVX_002570	0.359317338	0	0.394602024
PVX_002575	0.000125309	0	3.588020519
PVX_002580	0.007054102	0	2.313436078
PVX_002585	0.016473156	0	2.050196508
PVX_002590	3.76E-05	0	6.631237075
PVX_002595	0.078805602	0	1.252808489
PVX_002600	0.078805602	0	0.824040619
PVX_002605	0.007054102	0	2.131599564
PVX_002610	0.002855585	0	1.593964475
PVX_002615	0.007054102	0	2.595036038
PVX_002620	0.078805602	0	0.936912375
PVX_002625	0.078805602	0	1.173646589
PVX_002630	0.007054102	0	1.461895287
PVX_002635	1	0	0
PVX_002640	0.036687443	0	1.428677241
PVX_002645	0.002855585	0	2.62069355
PVX_002650	0.001084333	0	4.341538646
PVX_002655	0.078805602	0	0.936574344
PVX_002660	0.016473156	0	1.306254805

GeneID	Pvalue	BackGroundPre	BackGroundDx
PVX_002665	0.007054102	0	2.047912335
PVX_002670	0.007054102	0	2.518231528
PVX_002675	0.078805602	0	1.368567112
PVX_002680	0.002855585	0	1.941998869
PVX_002685	0.000383392	0	3.676689764
PVX_002690	0.166224591	0	0.427209308
PVX_002695	0.359317338	0	0.267771674
PVX_002700	0.166224591	0	0.399000705
PVX_002705	0.166224591	0	0.407801061
PVX_002710	0.002855585	0	2.371898744
PVX_002715	0.036687443	0	1.050334332
PVX_002720	0.000383392	0	3.311729345
PVX_002725	0.016473156	0	1.334625388
PVX_002730	0.166224591	0	0.682171297
PVX_002735	0.016473156	0	1.433249709
PVX_002740	0.166224591	0	0.505839964
PVX_002745	1	0	0
PVX_002750	0.359317338	0	0.266214919
PVX_002755	0.000125309	0	2.913164046
PVX_002760	0.002855585	0	2.864633612
PVX_002765	0.002855585	0	2.314273898
PVX_002770	0.016473156	0	1.53727639
PVX_002775	0.166224591	0	0.76880267
PVX_002780	0.359317338	0	0.323401599
PVX_002785	0.007054102	0	2.157416187
PVX_002790	1	0	0
PVX_002795	0.016473156	0	1.19643834
PVX_002800	1	0	0
PVX_002805	0.078805602	0	0.602858963
PVX_002810	0.078805602	0	1.191899612
PVX_002815	0.002855585	0	2.325683266
PVX_002820	0.078805602	0	0.632388935
PVX_002825	0.078805602	0	1.018538364
PVX_002830	0.000383392	0	2.9365274
PVX_002835	3.76E-05	0	4.692872299
PVX_002840	0.007054102	0	1.685187697
PVX_002845	0.002855585	0	2.217605118
PVX_002850	0.359317338	0	0.490707044
PVX_002855	0.078805602	0	0.459307018
PVX_002860	0.016473156	0	1.766932505
PVX_002865	0.036687443	0	1.28472965

GeneID	Pvalue	BackGroundPre	BackGroundDx
PVX_002867	0.078805602	0	1.960409
PVX_002870	0.166224591	0	0.935044547
PVX_002875	0.078805602	0	1.059050156
PVX_002880	0.016473156	0	1.94053151
PVX_002885	0.078805602	0	1.036567635
PVX_002890	0.002855585	0	2.274844383
PVX_002895	0.007054102	0	1.981225959
PVX_002900	0.078805602	0	1.196604876
PVX_002905	0.000125309	0	2.232877791
PVX_002910	0.000125309	0	2.426321618
PVX_002915	0.000383392	0	3.776976008
PVX_002920	0.007054102	0	2.236457098
PVX_002925	0.016473156	0	1.149719569
PVX_002930	0.007054102	0	2.239048188
PVX_002935	0.001084333	0	3.118781553
PVX_002940	0.000125309	0	4.342541448
PVX_002945	0.036687443	0	1.104423791
PVX_002950	0.166224591	0	0.758541115
PVX_002955	0.166224591	0	0.371053366
PVX_002960	1	0	0
PVX_002965	0.016473156	0	1.597851733
PVX_002970	0.007054102	0	2.332099956
PVX_002975	0.001084333	0	2.326337237
PVX_003475	0.166224591	0	0.418825877
PVX_003485	0.166224591	0	0.739271502
PVX_003487	0.166224591	0	0.808761653
PVX_003490	0.359317338	0	0.276747518
PVX_003495	1	0	0
PVX_003500	1	0	0
PVX_003505	0.166224591	0	0.498609044
PVX_003510	1	0	0
PVX_003515	0.359317338	0	0.335346659
PVX_003520	1	0	0
PVX_003525	0.166224591	0	0.386370804
PVX_003530	0.166224591	0	0.686742082
PVX_003535	0.359317338	0	0.226758718
PVX_003540	0.078805602	0	0.846059592
PVX_003545	0.000125309	0	4.546495652
PVX_003550	1	0	0
PVX_003555	0.000125309	0	3.546557361
PVX_003560	0.007054102	0	2.146813364

GeneID	Pvalue	BackGroundPre	BackGroundDx
PVX_003565	0.000383392	0	5.558524141
PVX_003570	0.007054102	0	1.639115368
PVX_003575	1	0	0
PVX_003578	0.359317338	0	0.619167775
PVX_003580	0.359317338	0	0.409657691
PVX_003585	0.036687443	0	1.0828871
PVX_003590	0.359317338	0	0.14346278
PVX_003595	0.002855585	0	2.413024667
PVX_003600	0.036687443	0	1.779524928
PVX_003605	0.166224591	0	0.916440339
PVX_003607	1	0	0
PVX_003610	0.078805602	0	0.937863189
PVX_003615	0.002855585	0	2.319180976
PVX_003620	0.001084333	0	2.86253032
PVX_003625	1	0	0
PVX_003630	0.166224591	0	0.492021246
PVX_003635	0.359317338	0	0.215204729
PVX_003640	0.036687443	0	1.508668952
PVX_003645	1	0	0
PVX_003650	1	0	0
PVX_003655	0.078805602	0	0.990678814
PVX_003660	0.166224591	0	0.452894225
PVX_003665	0.007054102	0	2.186178358
PVX_003670	0.359317338	0	0.284591691
PVX_003675	0.166224591	0	0.663364658
PVX_003680	0.078805602	0	0.72031923
PVX_003685	0.166224591	0	0.553227682
PVX_003690	1	0	0
PVX_003695	0.166224591	0	0.464623807
PVX_003700	0.000383392	0	3.282124907
PVX_003705	0.078805602	0	1.536665267
PVX_003710	0.166224591	0	1.049353907
PVX_003715	0.016473156	0	1.558629878
PVX_003720	0.002855585	0	2.68827295
PVX_003725	0.166224591	0	0.913020812
PVX_003730	3.76E-05	0	4.179871532
PVX_003735	0.016473156	0	1.120960917
PVX_003740	0.166224591	0	0.690372942
PVX_003745	1	0	0
PVX_003750	0.001084333	0	1.99298824
PVX_003755	0.007054102	0	1.794410621

GeneID	Pvalue	BackGroundPre	BackGroundDx
PVX_003760	0.359317338	0	0.628893839
PVX_003765	0.016473156	0	1.766509576
PVX_003770	0.359317338	0	0.299325261
PVX_003775	1	0	0
PVX_003780	0.001084333	0	1.905551121
PVX_003785	0.359317338	0	0.488643743
PVX_003790	1	0	0
PVX_003795	1	0	0
PVX_003800	0.007054102	0	1.61992831
PVX_003805	0.359317338	0	0.204026705
PVX_003810	0.359317338	0	0.350093881
PVX_003815	1	0	0
PVX_003820	0.359317338	0	0.283936591
PVX_003825	0.359317338	0	0.290682605
PVX_003830	1	0	0
PVX_003835	0.166224591	0	0.495035633
PVX_003840	0.166224591	0	0.418308469
PVX_003845	0.036687443	0	1.100175036
PVX_003850	1	0	0
PVX_003855	0.007054102	0	1.598568276
PVX_003860	0.007054102	0	2.691173776
PVX_003865	0.002855585	0	1.918904327
PVX_003870	0.002855585	0	1.702644164
PVX_003875	1	0	0
PVX_003880	0.016473156	0	2.203716104
PVX_003885	0.016473156	0	1.732249338
PVX_003890	0.036687443	0	1.448734339
PVX_003895	0.166224591	0	0.807662344
PVX_003900	0.036687443	0	0.876582284
PVX_003905	0.078805602	0	0.486131087
PVX_003910	0.078805602	0	0.984310098
PVX_003915	0.002855585	0	2.894106571
PVX_003920	0.078805602	0	1.078417745
PVX_003925	0.016473156	0	2.004823365
PVX_003930	1	0	0
PVX_003935	0.359317338	0	0.209816104
PVX_003940	0.007054102	0	1.948826373
PVX_003945	1.03E-05	0	5.86393157
PVX_003950	0.016473156	0	2.779847523
PVX_003955	0.000383392	0	5.892066795
PVX_003960	0.001084333	0	2.088573948

GeneID	Pvalue	BackGroundPre	BackGroundDx
PVX_003965	0.016473156	0	1.807099076
PVX_003970	0.166224591	0	0.951140284
PVX_003975	0.016473156	0	1.322006125
PVX_003980	0.359317338	0	0.234403546
PVX_003985	0.007054102	0	1.675837752
PVX_003990	0.359317338	0	0.351924615
PVX_003995	0.016473156	0	2.098581729
PVX_004495	1	0	0
PVX_004503	0.359317338	0	0.177897386
PVX_004505	1	0	0
PVX_004510	0.359317338	0	0.290507743
PVX_004515	0.036687443	0	1.643166798
PVX_004520	0.078805602	0	1.213792969
PVX_004525	0.359317338	0	0.302264521
PVX_004530	0.359317338	0	0.281696818
PVX_004535	0.016473156	0	1.893706874
PVX_004536	1	0	0
PVX_004537	0.359317338	0	0.264114901
PVX_004539	0.007054102	0	2.294521793
PVX_005040	0.166224591	0	0.528431114
PVX_005045	0.359317338	0	0.211537285
PVX_005050	1	0	0
PVX_005055	0.166224591	0	0.506547903
PVX_005057	0.078805602	0	1.099942623
PVX_005058	0.359317338	0	0.293178709
PVX_005060	0.359317338	0	0.43907233
PVX_005065	1	0	0
PVX_005565	1	0	0
PVX_005570	1	0	0
PVX_005575	1	0	0
PVX_005580	1	0	0
PVX_006080	0.359317338	0	0.21138707
PVX_006580	1	0	0
PVX_007080	1	0	0
PVX_007085	1	0	0
PVX_007585	1	0	0
PVX_008085	0.002855585	0	2.811320813
PVX_009090	1	0	0
PVX_009590	1	0	0
PVX_009595	1	0	0
PVX_009600	1	0	0

GeneID	Pvalue	BackGroundPre	BackGroundDx
PVX_010100	1	0	0
PVX_010105	1	0	0
PVX_010605	1	0	0
PVX_010610	1	0	0
PVX_011110	1	0	0
PVX_011610	1	0	0
PVX_011615	1	0	0
PVX_012115	0.166224591	0	0.537441518
PVX_013120	1	0	0
PVX_013620	0.166224591	0	0.535436804
PVX_013625	0.359317338	0	0.249462909
PVX_014125	1	0	0
PVX_014625	1	0	0
PVX_014630	1	0	0
PVX_015130	1	0	0
PVX_015135	1	0	0
PVX_015635	1	0	0
PVX_015640	1	0	0
PVX_016140	1	0	0
PVX_016640	1	0	0
PVX_017140	1	0	0
PVX_017145	1	0	0
PVX_017645	0.359317338	0	0.348072528
PVX_017650	1	0	0
PVX_018150	1	0	0
PVX_018155	1	0	0
PVX_018655	1	0	0
PVX_018660	1	0	0
PVX_019160	1	0	0
PVX_019165	0.166224591	0	0.762799929
PVX_019665	1	0	0
PVX_019670	1	0	0
PVX_020170	1	0	0
PVX_020175	1	0	0
PVX_020675	1	0	0
PVX_020680	0.359317338	0	0.310477665
PVX_021180	1	0	0
PVX_021680	1	0	0
PVX_021685	1	0	0
PVX_022185	0.002855585	0	2.614673855
PVX_022685	0.016473156	0	1.641213015

GeneID	Pvalue	BackGroundPre	BackGroundDx
PVX_023185	1	0	0
PVX_023685	1	0	0
PVX_024185	1	0	0
PVX_024685	0.036687443	0	1.356484351
PVX_024690	1	0	0
PVX_025190	0.359317338	0	0.544797456
PVX_025690	1	0	0
PVX_026190	1	0	0
PVX_026690	1	0	0
PVX_027190	1	0	0
PVX_027690	1	0	0
PVX_028190	1	0	0
PVX_028690	1	0	0
PVX_029190	1	0	0
PVX_029690	1	0	0
PVX_030190	1	0	0
PVX_030690	1	0	0
PVX_031190	1	0	0
PVX_031690	1	0	0
PVX_032190	0.359317338	0	0.256274316
PVX_032690	1	0	0
PVX_033190	1	0	0
PVX_033690	1	0	0
PVX_034190	1	0	0
PVX_034690	0.359317338	0	0.243629498
PVX_035190	1	0	0
PVX_035690	1	0	0
PVX_036190	1	0	0
PVX_036690	0.359317338	0	0.539247739
PVX_037190	1	0	0
PVX_037690	1	0	0
PVX_038190	1	0	0
PVX_038690	1	0	0
PVX_039190	1	0	0
PVX_039690	1	0	0
PVX_040190	1	0	0
PVX_040690	1	0	0
PVX_041190	1	0	0
PVX_041690	1	0	0
PVX_042190	1	0	0
PVX_042690	1	0	0

GeneID	Pvalue	BackGroundPre	BackGroundDx
PVX_043190	0.078805602	0	0.897968983
PVX_043690	1	0	0
PVX_044190	1	0	0
PVX_044690	1	0	0
PVX_045190	0.359317338	0	0.380681442
PVX_045690	1	0	0
PVX_046190	1	0	0
PVX_046690	1	0	0
PVX_047190	1	0	0
PVX_047690	1	0	0
PVX_048190	1	0	0
PVX_048690	1	0	0
PVX_049190	1	0	0
PVX_049690	0.078805602	0	0.92752964
PVX_050190	0.359317338	0	0.306034978
PVX_050690	1	0	0
PVX_051190	1	0	0
PVX_051690	1	0	0
PVX_052190	1	0	0
PVX_052690	0.359317338	0	0.280420131
PVX_053190	1	0	0
PVX_053690	1	0	0
PVX_054190	0.359317338	0	0.297078727
PVX_054690	1	0	0
PVX_055190	1	0	0
PVX_055690	0.359317338	0	0.369395293
PVX_056190	0.166224591	0	0.718797797
PVX_056690	1	0	0
PVX_057190	1	0	0
PVX_057690	1	0	0
PVX_058190	1	0	0
PVX_058690	1	0	0
PVX_059190	1	0	0
PVX_059690	1	0	0
PVX_060190	1	0	0
PVX_060690	1	0	0
PVX_061190	0.359317338	0	0.305443043
PVX_061690	1	0	0
PVX_062190	1	0	0
PVX_062690	0.016473156	0	1.474950508
PVX_063190	1	0	0

GeneID	Pvalue	BackGroundPre	BackGroundDx
PVX_063690	1	0	0
PVX_064190	1	0	0
PVX_064690	1	0	0
PVX_065190	1	0	0
PVX_065690	0.078805602	0	1.06188046
PVX_066190	0.166224591	0	0.619258907
PVX_066690	1	0	0
PVX_067190	1	0	0
PVX_067690	1	0	0
PVX_068190	1	0	0
PVX_068690	1	0	0
PVX_069190	1	0	0
PVX_069690	1	0	0
PVX_070190	1	0	0
PVX_070690	1	0	0
PVX_071190	1	0	0
PVX_071690	1	0	0
PVX_072190	0.359317338	0	0.305923501
PVX_072690	1	0	0
PVX_073190	0.359317338	0	0.32597808
PVX_073690	0.078805602	0	0.909686845
PVX_074190	1	0	0
PVX_074690	0.166224591	0	0.517771658
PVX_074695	1	0	0
PVX_075195	1	0	0
PVX_075695	1	0	0
PVX_076195	0.359317338	0	0.332586304
PVX_076695	1	0	0
PVX_077195	1	0	0
PVX_077695	0.016473156	0	1.203517715
PVX_078195	1	0	0
PVX_078695	1	0	0
PVX_079195	0.359317338	0	0.275532978
PVX_079695	0.001084333	0	2.284110308
PVX_079700	0.166224591	0	0.523896244
PVX_079705	0.036687443	0	1.49618259
PVX_079710	1	0	0
PVX_079715	0.016473156	0	1.327491685
PVX_079720	0.166224591	0	0.502778104
PVX_079725	0.166224591	0	0.754994975
PVX_079730	0.016473156	0	1.346185769

GeneID	Pvalue	BackGroundPre	BackGroundDx
PVX_079735	0.166224591	0	0.601087519
PVX_079740	1	0	0
PVX_079745	0.078805602	0	0.75527417
PVX_079750	0.078805602	0	0.776193126
PVX_079755	0.016473156	0	1.748759389
PVX_079760	0.078805602	0	1.125717502
PVX_079765	0.007054102	0	2.487401054
PVX_079770	0.036687443	0	1.600570318
PVX_079772	0.359317338	0	0.123176197
PVX_079775	0.036687443	0	1.014218453
PVX_079778	1	0	0
PVX_079780	0.036687443	0	1.724641632
PVX_079785	0.007054102	0	1.413027725
PVX_079790	1	0	0
PVX_079795	0.078805602	0	1.177259411
PVX_079800	0.036687443	0	1.406971593
PVX_079805	1	0	0
PVX_079810	0.002855585	0	2.334480438
PVX_079815	0.036687443	0	1.162993452
PVX_079820	0.078805602	0	0.954189571
PVX_079825	1	0	0
PVX_079830	1	0	0
PVX_079835	1	0	0
PVX_079840	0.001084333	0	3.431769044
PVX_079845	0.002855585	0	2.302698036
PVX_079850	0.002855585	0	3.07346689
PVX_079855	0.001084333	0	2.386416595
PVX_079857	1	0	0
PVX_079858	1	0	0
PVX_079860	1	0	0
PVX_079865	0.000125309	0	4.463659174
PVX_079870	0.078805602	0	1.377819259
PVX_079875	0.078805602	0	1.232830999
PVX_079880	0.002855585	0	2.369396062
PVX_079885	0.002855585	0	3.02689952
PVX_079890	0.016473156	0	1.451425994
PVX_079895	0.078805602	0	1.073605765
PVX_079900	0.036687443	0	1.493536005
PVX_079905	0.001084333	0	2.999153357
PVX_079910	0.002855585	0	1.194713703
PVX_079915	1	0	0

GeneID	Pvalue	BackGroundPre	BackGroundDx
PVX_079920	0.016473156	0	1.599743715
PVX_079925	0.001084333	0	2.931110175
PVX_079930	0.001084333	0	2.959235501
PVX_079935	0.078805602	0	0.952126839
PVX_079940	0.007054102	0	2.155024082
PVX_079945	0.016473156	0	1.527334571
PVX_079950	1	0	0
PVX_079955	0.359317338	0	0.272623938
PVX_079960	0.036687443	0	1.504784404
PVX_079965	0.359317338	0	0.305374759
PVX_079970	0.007054102	0	1.537177952
PVX_079975	0.036687443	0	1.291845305
PVX_079980	0.078805602	0	1.069980917
PVX_079985	0.016473156	0	1.119702853
PVX_079990	0.000125309	0	3.739778411
PVX_079995	0.359317338	0	0.157214127
PVX_080000	0.016473156	0	1.645846007
PVX_080005	0.036687443	0	1.508480349
PVX_080010	0.016473156	0	1.428358395
PVX_080015	0.016473156	0	2.194734068
PVX_080020	0.078805602	0	1.231455496
PVX_080025	0.166224591	0	0.931939402
PVX_080030	0.016473156	0	1.43021336
PVX_080035	0.002855585	0	2.593367135
PVX_080040	1	0	0
PVX_080045	0.016473156	0	1.38738484
PVX_080050	1.03E-05	0	4.51881195
PVX_080055	1	0	0
PVX_080060	0.000383392	0	3.111868125
PVX_080065	0.036687443	0	1.084102358
PVX_080070	0.078805602	0	1.01926815
PVX_080075	1	0	0
PVX_080080	0.002855585	0	2.344064708
PVX_080085	0.078805602	0	1.516022015
PVX_080090	0.002855585	0	2.274387298
PVX_080095	0.000383392	0	3.404880501
PVX_080100	0.000125309	0	3.422766359
PVX_080105	0.007054102	0	1.432094641
PVX_080110	0.002855585	0	2.882823296
PVX_080115	0.078805602	0	1.263815457
PVX_080120	0.166224591	0	0.614286024

GeneID	Pvalue	BackGroundPre	BackGroundDx
PVX_080125	0.166224591	0	0.854070772
PVX_080130	0.078805602	0	0.294280623
PVX_080135	0.016473156	0	1.660652422
PVX_080140	0.166224591	0	0.780284896
PVX_080145	1	0	0
PVX_080147	0.359317338	0	0.456705673
PVX_080150	1	0	0
PVX_080155	0.036687443	0	0.982333049
PVX_080160	0.007054102	0	1.70963214
PVX_080165	0.359317338	0	0.44604503
PVX_080170	0.036687443	0	1.311952216
PVX_080175	0.016473156	0	1.427692032
PVX_080180	0.001084333	0	2.178613242
PVX_080185	0.078805602	0	0.874477443
PVX_080190	0.078805602	0	0.713240002
PVX_080195	0.016473156	0	1.594184995
PVX_080200	0.000125309	0	4.362282928
PVX_080205	0.016473156	0	1.028545034
PVX_080210	0.036687443	0	1.265504172
PVX_080215	0.036687443	0	1.522360439
PVX_080220	0.359317338	0	0.322098928
PVX_080225	0.016473156	0	2.103051742
PVX_080230	0.007054102	0	2.553097304
PVX_080235	0.000383392	0	3.230036805
PVX_080240	0.016473156	0	1.607605781
PVX_080245	1.03E-05	0	6.832841883
PVX_080250	0.078805602	0	1.132650219
PVX_080255	0.007054102	0	1.693930198
PVX_080260	0.000383392	0	4.444854351
PVX_080265	0.002855585	0	2.588212802
PVX_080270	0.166224591	0	0.42746136
PVX_080275	1.03E-05	0	6.2481865
PVX_080280	0.078805602	0	0.817030437
PVX_080285	0.007054102	0	2.645317266
PVX_080290	0.359317338	0	0.446320059
PVX_080295	0.002855585	0	2.616995892
PVX_080300	1	0	0
PVX_080305	1	0	0
PVX_080310	1	0	0
PVX_080315	0.002855585	0	1.974659218
PVX_080320	0.166224591	0	0.374086408

GeneID	Pvalue	BackGroundPre	BackGroundDx
PVX_080325	1	0	0
PVX_080330	0.000125309	0	4.169135222
PVX_080335	0.036687443	0	1.592317212
PVX_080340	0.036687443	0	0.975072091
PVX_080345	0.078805602	0	1.340694395
PVX_080350	0.036687443	0	1.435256217
PVX_080355	0.036687443	0	0.973512384
PVX_080360	0.166224591	0	0.880783729
PVX_080365	3.76E-05	0	4.865414286
PVX_080370	1	0	0
PVX_080375	0.359317338	0	0.226765847
PVX_080380	0.001084333	0	2.476793251
PVX_080385	0.007054102	0	2.458388929
PVX_080390	0.166224591	0	0.581713584
PVX_080395	0.166224591	0	0.731086902
PVX_080400	1.03E-05	0	6.734039357
PVX_080405	3.76E-05	0	5.682445558
PVX_080410	0.036687443	0	0.875281964
PVX_080415	0.000125309	0	3.2844556
PVX_080420	0.001084333	0	2.263030638
PVX_080425	1	0	0
PVX_080430	0.002855585	0	2.801687455
PVX_080435	0.016473156	0	1.603207854
PVX_080440	0.000125309	0	5.321336516
PVX_080445	0.016473156	0	1.087919852
PVX_080450	0.000125309	0	3.857959207
PVX_080455	0.078805602	0	1.762032973
PVX_080460	0.078805602	0	0.902674871
PVX_080465	0.166224591	0	0.409559045
PVX_080470	1	0	0
PVX_080475	1	0	0
PVX_080480	0.016473156	0	1.34302353
PVX_080485	0.036687443	0	1.680044343
PVX_080490	1	0	0
PVX_080495	0.016473156	0	1.54340628
PVX_080500	0.007054102	0	2.042898957
PVX_080505	0.016473156	0	1.278231216
PVX_080510	0.166224591	0	0.478604826
PVX_080515	0.036687443	0	1.73306935
PVX_080520	1	0	0
PVX_080523	1	0	0

GeneID	Pvalue	BackGroundPre	BackGroundDx
PVX_080525	0.001084333	0	2.693453536
PVX_080530	0.078805602	0	0.792548423
PVX_080535	0.078805602	0	0.942058487
PVX_080540	0.166224591	0	0.730363957
PVX_080545	0.016473156	0	1.933520472
PVX_080550	0.016473156	0	2.238328255
PVX_080555	0.359317338	0	0.224892785
PVX_080560	0.007054102	0	1.533377619
PVX_080565	0.078805602	0	1.415836508
PVX_080570	0.359317338	0	0.277207051
PVX_080575	1.03E-05	0	5.728578115
PVX_080580	0.002855585	0	1.798310616
PVX_080585	0.359317338	0	0.290652298
PVX_080590	0.359317338	0	0.300986142
PVX_080595	0.359317338	0	0.275057951
PVX_080600	0.078805602	0	0.944606697
PVX_080605	0.359317338	0	0.289368314
PVX_080610	0.078805602	0	1.140174433
PVX_080615	0.078805602	0	0.787711079
PVX_080617	0.359317338	0	0.197383829
PVX_080620	1	0	0
PVX_080625	0.002855585	0	2.558782154
PVX_080630	0.036687443	0	1.199145161
PVX_080635	0.016473156	0	2.145938726
PVX_080640	0.001084333	0	4.311211581
PVX_080645	0.078805602	0	0.879028735
PVX_080650	1.03E-05	0	6.324648175
PVX_080655	0.036687443	0	1.643813966
PVX_080657	0.359317338	0	0.372367561
PVX_080660	0.000125309	0	2.090681456
PVX_080665	0.166224591	0	0.643727351
PVX_080670	0.166224591	0	0.407019965
PVX_080675	0.001084333	0	2.562152784
PVX_080680	0.166224591	0	0.581849506
PVX_080685	0.000125309	0	4.482348433
PVX_080690	1	0	0
PVX_080700	1	0	0
PVX_081200	1	0	0
PVX_081205	0.016473156	0	1.813048794
PVX_081210	0.078805602	0	1.293421265
PVX_081215	0.001084333	0	2.803374827

GeneID	Pvalue	BackGroundPre	BackGroundDx
PVX_081220	0.036687443	0	1.984994095
PVX_081225	0.007054102	0	2.414866202
PVX_081230	0.036687443	0	1.28912221
PVX_081235	0.016473156	0	1.528566435
PVX_081240	0.007054102	0	1.806067279
PVX_081245	0.036687443	0	1.639271232
PVX_081250	0.359317338	0	0.1370304
PVX_081255	0.016473156	0	2.03262134
PVX_081260	0.036687443	0	1.491881371
PVX_081265	0.000125309	0	4.706043621
PVX_081270	0.002855585	0	1.993430257
PVX_081275	0.016473156	0	1.129223372
PVX_081277	0.001084333	0	3.294450784
PVX_081280	0.078805602	0	1.420295216
PVX_081285	0.002855585	0	2.302741753
PVX_081290	0.166224591	0	0.788857287
PVX_081295	0.359317338	0	0.275462707
PVX_081300	0.001084333	0	3.250038937
PVX_081305	0.036687443	0	1.760073275
PVX_081307	0.078805602	0	1.353986706
PVX_081310	0.016473156	0	2.377989441
PVX_081315	0.166224591	0	0.855790763
PVX_081320	0.002855585	0	2.951999599
PVX_081325	0.002855585	0	3.01799745
PVX_081330	0.166224591	0	0.492537977
PVX_081335	0.359317338	0	0.45757729
PVX_081340	0.016473156	0	1.76302025
PVX_081345	0.166224591	0	0.473407476
PVX_081350	0.016473156	0	2.072403031
PVX_081355	0.016473156	0	2.179419955
PVX_081360	0.359317338	0	0.393902512
PVX_081365	1.03E-05	0	3.987626695
PVX_081370	0.016473156	0	1.640189476
PVX_081375	0.000383392	0	3.981060626
PVX_081380	0.036687443	0	1.678665366
PVX_081385	0.002855585	0	2.302798657
PVX_081390	0.007054102	0	2.062728892
PVX_081395	0.166224591	0	0.301669139
PVX_081400	0.001084333	0	2.515809786
PVX_081405	0.002855585	0	2.916837935
PVX_081410	0.166224591	0	0.569320321

GeneID	Pvalue	BackGroundPre	BackGroundDx
PVX_081415	0.166224591	0	0.701563056
PVX_081420	0.166224591	0	1.072978038
PVX_081425	1	0	0
PVX_081430	0.359317338	0	0.310136821
PVX_081435	0.007054102	0	1.856749384
PVX_081440	1	0	0
PVX_081445	1	0	0
PVX_081450	0.036687443	0	1.475121246
PVX_081455	0.002855585	0	2.061336892
PVX_081460	0.359317338	0	0.478096333
PVX_081465	0.166224591	0	0.675679547
PVX_081470	0.016473156	0	1.775850438
PVX_081475	0.001084333	0	3.02740462
PVX_081480	0.078805602	0	0.92755853
PVX_081485	0.001084333	0	2.103808049
PVX_081490	0.001084333	0	2.313998508
PVX_081495	0.036687443	0	1.655874875
PVX_081498	0.166224591	0	0.838830547
PVX_081502	0.166224591	0	0.860899414
PVX_081505	0.000383392	0	3.300738578
PVX_081510	0.036687443	0	1.788313791
PVX_081515	0.016473156	0	1.34856486
PVX_081520	1	0	0
PVX_081525	0.036687443	0	0.923207914
PVX_081530	0.000383392	0	3.243669159
PVX_081535	0.002855585	0	2.73884442
PVX_081540	0.000383392	0	2.046635417
PVX_081550	0.359317338	0	0.27586581
PVX_081555	0.359317338	0	0.234358008
PVX_081560	1	0	0
PVX_081565	0.001084333	0	3.019287256
PVX_081570	0.166224591	0	0.665754703
PVX_081572	0.036687443	0	1.219515689
PVX_081575	0.036687443	0	0.689637314
PVX_081580	0.359317338	0	0.207174107
PVX_081585	0.000125309	0	3.212146072
PVX_081590	0.016473156	0	1.463418722
PVX_081595	0.000383392	0	3.676387184
PVX_081600	0.078805602	0	0.685835276
PVX_081605	1	0	0
PVX_081610	0.000125309	0	3.978069819

GeneID	Pvalue	BackGroundPre	BackGroundDx
PVX_081615	0.078805602	0	0.973799135
PVX_081620	0.036687443	0	1.090541588
PVX_081625	0.001084333	0	1.641370656
PVX_081630	0.000125309	0	2.588129615
PVX_081635	0.016473156	0	1.459081386
PVX_081640	0.016473156	0	2.337469633
PVX_081645	0.166224591	0	1.090393365
PVX_081650	0.007054102	0	2.317407481
PVX_081655	0.007054102	0	1.669702912
PVX_081660	0.007054102	0	1.831039781
PVX_081665	1	0	0
PVX_081670	0.036687443	0	0.99153171
PVX_081675	0.002855585	0	2.831516339
PVX_081680	0.166224591	0	0.652404069
PVX_081685	0.007054102	0	1.679312804
PVX_081690	0.007054102	0	1.671565803
PVX_081695	0.001084333	0	2.378646067
PVX_081700	0.036687443	0	0.946427838
PVX_081705	0.001084333	0	3.32241708
PVX_081707	0.166224591	0	0.265534853
PVX_081710	1	0	0
PVX_081715	0.036687443	0	1.665897276
PVX_081720	0.166224591	0	0.823658273
PVX_081722	1	0	0
PVX_081725	0.000383392	0	2.498710032
PVX_081730	0.359317338	0	0.253680833
PVX_081740	0.016473156	0	1.727827317
PVX_081745	0.001084333	0	2.804456458
PVX_081750	0.016473156	0	2.114273882
PVX_081755	0.166224591	0	0.2697562
PVX_081760	0.036687443	0	1.612487879
PVX_081765	0.002855585	0	2.474922323
PVX_081770	0.002855585	0	2.643571987
PVX_081775	0.002855585	0	2.308825384
PVX_081780	0.036687443	0	1.612749729
PVX_081785	0.007054102	0	1.856326352
PVX_081790	1	0	0
PVX_081792	0.078805602	0	0.430844062
PVX_081795	1	0	0
PVX_081800	0.036687443	0	1.067849206
PVX_081805	0.007054102	0	1.782336591

GeneID	Pvalue	BackGroundPre	BackGroundDx
PVX_081810	0.000383392	0	3.242319682
PVX_081815	1	0	0
PVX_081820	0.002855585	0	1.39589732
PVX_081822	1	0	0
PVX_081824	1	0	0
PVX_081825	1	0	0
PVX_081830	1.03E-05	0	6.182988102
PVX_081832	0.016473156	0	1.826031437
PVX_081835	0.359317338	0	0.318643931
PVX_081840	0.036687443	0	1.502552243
PVX_081845	0.036687443	0	1.271052629
PVX_081847	3.76E-05	0	4.771442969
PVX_081850	1	0	0
PVX_082350	0.078805602	0	0.61088527
PVX_082355	0.000125309	0	4.497496013
PVX_082360	0.166224591	0	0.416439892
PVX_082365	0.078805602	0	1.180045776
PVX_082370	0.016473156	0	1.713229266
PVX_082375	0.016473156	0	1.434928631
PVX_082380	1	0	0
PVX_082385	0.007054102	0	2.234225253
PVX_082390	0.002855585	0	2.730122284
PVX_082395	0.001084333	0	1.943916727
PVX_082400	0.016473156	0	1.257512507
PVX_082405	1	0	0
PVX_082410	0.007054102	0	1.968981898
PVX_082415	1	0	0
PVX_082420	0.001084333	0	1.769169272
PVX_082425	0.001084333	0	2.950542104
PVX_082430	1	0	0
PVX_082435	0.166224591	0	0.522306611
PVX_082437	0.359317338	0	0.42107096
PVX_082440	0.078805602	0	1.271074632
PVX_082445	0.002855585	0	3.063868577
PVX_082450	0.001084333	0	3.441736045
PVX_082455	0.078805602	0	1.099982344
PVX_082460	0.359317338	0	0.33094582
PVX_082465	0.000383392	0	3.20701358
PVX_082470	0.000383392	0	3.597284643
PVX_082475	0.001084333	0	2.405672472
PVX_082480	0.000125309	0	3.842971398

GeneID	Pvalue	BackGroundPre	BackGroundDx
PVX_082485	0.359317338	0	0.220178191
PVX_082490	0.166224591	0	0.436374869
PVX_082495	0.036687443	0	1.513353225
PVX_082500	0.078805602	0	1.22925608
PVX_082505	1	0	0
PVX_082510	0.036687443	0	1.221141007
PVX_082515	0.036687443	0	1.553324568
PVX_082520	3.76E-05	0	4.312916698
PVX_082525	0.000125309	0	5.408594197
PVX_082530	0.036687443	0	1.496169469
PVX_082535	1	0	0
PVX_082540	1	0	0
PVX_082545	0.078805602	0	1.28353896
PVX_082550	3.76E-05	0	3.895991256
PVX_082555	0.007054102	0	2.33685912
PVX_082560	0.001084333	0	2.993660672
PVX_082565	0.036687443	0	1.141254158
PVX_082570	0.007054102	0	2.642649169
PVX_082575	0.001084333	0	2.590932203
PVX_082580	0.016473156	0	1.865339446
PVX_082585	1	0	0
PVX_082590	0.078805602	0	0.57852453
PVX_082595	0.036687443	0	1.845960884
PVX_082600	0.007054102	0	2.658210094
PVX_082605	0.036687443	0	1.572898388
PVX_082610	0.001084333	0	2.336314475
PVX_082615	1	0	0
PVX_082620	0.078805602	0	1.130518031
PVX_082625	0.007054102	0	1.646049281
PVX_082630	0.359317338	0	0.277456316
PVX_082635	0.016473156	0	1.431186734
PVX_082640	0.001084333	0	2.981496162
PVX_082645	0.002855585	0	3.029040224
PVX_082650	1	0	0
PVX_082655	1	0	0
PVX_082660	0.166224591	0	0.821981928
PVX_082665	1	0	0
PVX_082670	0.359317338	0	0.330460172
PVX_082675	1	0	0
PVX_082680	1	0	0
PVX_082685	1	0	0

GeneID	Pvalue	BackGroundPre	BackGroundDx
PVX_082690	1	0	0
PVX_082695	1	0	0
PVX_082700	0.359317338	0	0.370658123
PVX_082710	0.036687443	0	1.605169262
PVX_082715	1	0	0
PVX_082720	0.016473156	0	1.97942847
PVX_082725	0.007054102	0	1.723984793
PVX_082730	0.007054102	0	1.912771254
PVX_082735	1	0	0
PVX_082740	0.166224591	0	0.601285354
PVX_082742	0.078805602	0	1.31203104
PVX_082745	0.001084333	0	3.027607256
PVX_082750	0.036687443	0	1.184343262
PVX_082755	0.016473156	0	1.917023577
PVX_082760	0.078805602	0	1.18850935
PVX_082765	0.016473156	0	1.84381811
PVX_082770	0.007054102	0	2.433607236
PVX_082775	0.016473156	0	1.643849007
PVX_082780	0.036687443	0	0.995050787
PVX_082785	0.036687443	0	1.485270744
PVX_082790	0.002855585	0	2.249412602
PVX_082795	0.001084333	0	2.709218928
PVX_082800	0.036687443	0	1.189770388
PVX_082810	0.036687443	0	0.765987903
PVX_082813	1	0	0
PVX_082815	1	0	0
PVX_082820	0.359317338	0	0.342726778
PVX_082825	0.078805602	0	0.95105852
PVX_082830	0.078805602	0	1.048267682
PVX_082835	0.002855585	0	2.758254693
PVX_082840	0.000125309	0	5.659509992
PVX_082845	3.76E-05	0	5.318486109
PVX_082850	1	0	0
PVX_082855	0.002855585	0	2.27350406
PVX_082860	0.002855585	0	2.330172085
PVX_082865	0.016473156	0	1.623844946
PVX_082870	1	0	0
PVX_082875	1	0	0
PVX_082880	0.036687443	0	1.111180678
PVX_082882	1	0	0
PVX_082884	1	0	0

GeneID	Pvalue	BackGroundPre	BackGroundDx
PVX_082885	0.166224591	0	0.770315144
PVX_082890	0.016473156	0	2.453219137
PVX_082895	0.078805602	0	1.216392024
PVX_082900	0.036687443	0	1.435697431
PVX_082905	0.078805602	0	0.675482723
PVX_082910	0.078805602	0	1.019844308
PVX_082915	0.166224591	0	0.571379271
PVX_082920	1	0	0
PVX_082925	0.007054102	0	2.310736515
PVX_082930	1	0	0
PVX_082935	0.007054102	0	2.011565337
PVX_082937	0.166224591	0	0.71688819
PVX_082938	0.359317338	0	0.200893479
PVX_082945	0.007054102	0	2.258733492
PVX_082950	1	0	0
PVX_082955	0.359317338	0	0.419391401
PVX_082960	0.016473156	0	1.632589936
PVX_082962	1	0	0
PVX_082964	1	0	0
PVX_082965	3.76E-05	0	5.303473389
PVX_082970	0.002855585	0	3.388817311
PVX_082975	1	0	0
PVX_082980	0.036687443	0	1.00419356
PVX_082985	0.359317338	0	0.458048499
PVX_082990	0.359317338	0	0.235234861
PVX_082995	0.166224591	0	0.907514336
PVX_083000	1.03E-05	0	6.648162913
PVX_083005	0.016473156	0	1.730413473
PVX_083010	0.036687443	0	1.139759821
PVX_083015	0.016473156	0	1.92850725
PVX_083020	0.007054102	0	2.160810518
PVX_083025	1	0	0
PVX_083030	3.76E-05	0	3.828318642
PVX_083032	0.359317338	0	0.62761016
PVX_083035	0.016473156	0	1.750894851
PVX_083040	0.001084333	0	1.689372396
PVX_083045	3.76E-05	0	6.227257706
PVX_083050	0.036687443	0	1.145493024
PVX_083055	0.002855585	0	2.394415889
PVX_083060	0.002855585	0	2.206956363
PVX_083065	0.036687443	0	0.948320795

GeneID	Pvalue	BackGroundPre	BackGroundDx
PVX_083070	0.036687443	0	1.633316722
PVX_083075	0.078805602	0	1.059826943
PVX_083080	0.016473156	0	1.514591066
PVX_083085	0.359317338	0	0.081275858
PVX_083090	0.166224591	0	0.666813066
PVX_083095	0.007054102	0	1.658738516
PVX_083100	0.036687443	0	1.055739868
PVX_083105	3.76E-05	0	3.858391849
PVX_083110	0.002855585	0	1.949130465
PVX_083115	0.036687443	0	1.255285948
PVX_083120	0.036687443	0	1.653850181
PVX_083125	0.166224591	0	0.533291627
PVX_083130	0.007054102	0	1.764729981
PVX_083135	0.036687443	0	1.432929926
PVX_083140	0.007054102	0	1.549835687
PVX_083150	0.166224591	0	0.666396482
PVX_083155	0.016473156	0	2.19602086
PVX_083160	0.166224591	0	0.528829747
PVX_083165	0.002855585	0	2.938179286
PVX_083170	0.078805602	0	0.546233866
PVX_083175	0.016473156	0	2.148433413
PVX_083180	1	0	0
PVX_083185	0.001084333	0	3.193834683
PVX_083190	0.000383392	0	3.308514579
PVX_083195	0.166224591	0	0.760990894
PVX_083200	1	0	0
PVX_083205	0.000383392	0	3.949273462
PVX_083210	0.078805602	0	1.179245744
PVX_083215	0.007054102	0	2.579005712
PVX_083220	0.002855585	0	1.525541924
PVX_083225	0.007054102	0	2.098886843
PVX_083230	0.016473156	0	1.915876791
PVX_083235	0.359317338	0	0.250351349
PVX_083240	0.036687443	0	1.124384386
PVX_083245	0.036687443	0	1.510405151
PVX_083250	0.359317338	0	0.291323594
PVX_083255	0.036687443	0	1.122152629
PVX_083260	0.007054102	0	2.110547311
PVX_083262	1	0	0
PVX_083265	0.166224591	0	0.875057363
PVX_083270	1.03E-05	0	6.278241574

GeneID	Pvalue	BackGroundPre	BackGroundDx
PVX_083275	0.359317338	0	0.274471702
PVX_083280	0.001084333	0	3.037953791
PVX_083285	0.078805602	0	1.009059274
PVX_083290	0.007054102	0	1.636780867
PVX_083295	1	0	0
PVX_083300	1	0	0
PVX_083305	0.166224591	0	0.698331047
PVX_083310	0.007054102	0	1.632840775
PVX_083315	0.007054102	0	0.984364102
PVX_083320	0.036687443	0	1.611590341
PVX_083325	0.166224591	0	0.544940255
PVX_083330	0.078805602	0	0.705596721
PVX_083335	0.166224591	0	0.302899117
PVX_083340	0.007054102	0	2.184709319
PVX_083345	0.359317338	0	0.374096933
PVX_083350	0.000125309	0	5.141656104
PVX_083355	0.000383392	0	3.061408971
PVX_083360	0.007054102	0	1.61868624
PVX_083365	0.078805602	0	1.132142475
PVX_083370	0.001084333	0	2.025665592
PVX_083375	0.036687443	0	1.293557475
PVX_083380	0.166224591	0	0.693878573
PVX_083385	0.007054102	0	2.058527192
PVX_083390	1	0	0
PVX_083395	1	0	0
PVX_083400	0.000383392	0	3.317673909
PVX_083405	0.036687443	0	2.165345982
PVX_083410	0.001084333	0	3.356804047
PVX_083415	0.078805602	0	0.891289008
PVX_083420	0.078805602	0	0.786689582
PVX_083425	0.016473156	0	1.980737215
PVX_083430	0.078805602	0	0.964423546
PVX_083435	0.078805602	0	1.129340732
PVX_083440	1	0	0
PVX_083445	0.002855585	0	2.230206485
PVX_083450	0.007054102	0	2.715206882
PVX_083455	0.036687443	0	0.928261602
PVX_083460	0.078805602	0	0.911875764
PVX_083465	3.76E-05	0	5.864579692
PVX_083470	0.078805602	0	0.748075089
PVX_083475	1	0	0

GeneID	Pvalue	BackGroundPre	BackGroundDx
PVX_083480	0.000125309	0	4.383577147
PVX_083485	0.166224591	0	0.600896589
PVX_083490	0.007054102	0	1.421821226
PVX_083495	0.036687443	0	1.046254547
PVX_083500	0.007054102	0	1.708338516
PVX_083505	0.078805602	0	0.829445284
PVX_083510	0.002855585	0	2.080220238
PVX_083515	0.007054102	0	2.265622827
PVX_083520	0.166224591	0	0.515745358
PVX_083530	0.000383392	0	2.23211373
PVX_083535	0.036687443	0	1.231037956
PVX_083545	0.078805602	0	1.13567311
PVX_083550	0.016473156	0	1.504145529
PVX_083555	3.76E-05	0	5.889263961
PVX_083560	1.03E-05	0	6.388231546
PVX_083565	1	0	0
PVX_083570	0.078805602	0	1.128041467
PVX_083575	1	0	0
PVX_083580	1	0	0
PVX_083585	0.359317338	0	0.27046598
PVX_083590	0.166224591	0	0.730145731
PVX_084090	0.359317338	0	0.502648001
PVX_084095	1	0	0
PVX_084100	0.007054102	0	1.955805357
PVX_084105	0.359317338	0	0.308284069
PVX_084110	1	0	0
PVX_084115	0.359317338	0	0.319373306
PVX_084120	0.016473156	0	1.940906756
PVX_084125	0.001084333	0	2.644575902
PVX_084130	0.036687443	0	1.549434
PVX_084135	0.002855585	0	2.54043937
PVX_084160	0.036687443	0	0.546928322
PVX_084165	0.016473156	0	1.624606001
PVX_084170	0.359317338	0	0.259694094
PVX_084175	0.001084333	0	2.302121784
PVX_084180	0.000383392	0	3.445837155
PVX_084185	0.078805602	0	1.696554789
PVX_084190	0.001084333	0	4.178654461
PVX_084195	0.359317338	0	0.154967863
PVX_084200	0.016473156	0	1.721216723
PVX_084205	0.007054102	0	2.688765593

GeneID	Pvalue	BackGroundPre	BackGroundDx
PVX_084210	0.016473156	0	1.771286238
PVX_084215	0.001084333	0	2.599434106
PVX_084220	0.001084333	0	2.733191294
PVX_084225	0.016473156	0	1.846257177
PVX_084230	0.000125309	0	3.641339761
PVX_084235	0.036687443	0	1.69631169
PVX_084240	1	0	0
PVX_084245	0.359317338	0	0.287133612
PVX_084250	0.016473156	0	1.634156418
PVX_084255	0.000125309	0	4.409143208
PVX_084260	0.359317338	0	0.285492188
PVX_084265	0.036687443	0	1.273925833
PVX_084270	0.078805602	0	0.840972027
PVX_084275	0.036687443	0	1.663661073
PVX_084277	1	0	0
PVX_084280	0.000383392	0	2.784342842
PVX_084285	0.036687443	0	1.218610165
PVX_084290	1	0	0
PVX_084295	0.078805602	0	0.710148532
PVX_084300	0.078805602	0	0.74427374
PVX_084305	0.007054102	0	1.654317881
PVX_084310	0.000125309	0	4.446860803
PVX_084315	0.359317338	0	0.319571762
PVX_084320	1	0	0
PVX_084325	0.000125309	0	2.434680664
PVX_084330	0.000383392	0	2.942282821
PVX_084335	0.007054102	0	2.478796551
PVX_084340	0.359317338	0	0.306847458
PVX_084345	3.76E-05	0	4.209337542
PVX_084350	0.166224591	0	0.35703954
PVX_084355	0.036687443	0	1.43514961
PVX_084360	1	0	0
PVX_084365	0.036687443	0	1.151092905
PVX_084370	0.000383392	0	3.627912738
PVX_084375	0.359317338	0	0.322655448
PVX_084380	0.078805602	0	0.62678279
PVX_084385	0.359317338	0	0.101855947
PVX_084390	0.001084333	0	2.694522995
PVX_084395	0.166224591	0	0.415971143
PVX_084400	1	0	0
PVX_084405	1	0	0

GeneID	Pvalue	BackGroundPre	BackGroundDx
PVX_084410	0.359317338	0	0.297590769
PVX_084415	0.166224591	0	0.706800746
PVX_084417	0.007054102	0	2.009194845
PVX_084420	1	0	0
PVX_084425	0.359317338	0	0.3297943
PVX_084430	0.078805602	0	0.823762625
PVX_084435	0.166224591	0	0.733016427
PVX_084440	0.002855585	0	1.531380923
PVX_084445	0.036687443	0	0.65834853
PVX_084450	0.016473156	0	2.211556497
PVX_084455	0.166224591	0	0.582953886
PVX_084460	0.166224591	0	0.427766011
PVX_084462	0.078805602	0	1.653749964
PVX_084465	0.001084333	0	3.099643512
PVX_084470	0.016473156	0	1.104223315
PVX_084475	0.078805602	0	0.658018336
PVX_084480	0.007054102	0	2.65986055
PVX_084485	0.359317338	0	0.191210307
PVX_084490	0.016473156	0	1.865172848
PVX_084495	0.036687443	0	0.867548882
PVX_084500	0.078805602	0	1.013315829
PVX_084505	0.166224591	0	0.366411818
PVX_084510	1	0	0
PVX_084515	0.016473156	0	1.864268616
PVX_084519	0.036687443	0	1.795135737
PVX_084521	0.016473156	0	1.320305476
PVX_084525	0.016473156	0	1.664886241
PVX_084530	0.359317338	0	0.1139559
PVX_084535	0.078805602	0	0.991990404
PVX_084540	0.166224591	0	0.335280819
PVX_084545	0.007054102	0	2.441750752
PVX_084550	1	0	0
PVX_084555	0.078805602	0	0.787086064
PVX_084560	0.016473156	0	1.739878407
PVX_084565	0.007054102	0	1.644751961
PVX_084570	0.078805602	0	0.760312146
PVX_084575	1	0	0
PVX_084580	1	0	0
PVX_084585	1	0	0
PVX_084590	0.002855585	0	2.046753193
PVX_084595	0.036687443	0	0.940864184

GeneID	Pvalue	BackGroundPre	BackGroundDx
PVX_084600	0.000383392	0	3.897224423
PVX_084605	0.007054102	0	2.11175899
PVX_084610	0.078805602	0	0.600189638
PVX_084615	0.016473156	0	1.572029058
PVX_084620	1.03E-05	0	5.652099781
PVX_084625	0.002855585	0	1.874531799
PVX_084630	0.001084333	0	3.415077946
PVX_084635	0.007054102	0	1.984249584
PVX_084640	0.036687443	0	1.625151953
PVX_084645	0.001084333	0	3.150538698
PVX_084650	0.000383392	0	3.961862359
PVX_084655	0.007054102	0	2.256925862
PVX_084660	0.001084333	0	2.582641783
PVX_084665	0.007054102	0	2.135514558
PVX_084670	0.036687443	0	1.592291876
PVX_084675	0.000383392	0	3.395352638
PVX_084680	1	0	0
PVX_084685	0.001084333	0	2.715522727
PVX_084687	0.078805602	0	1.297015195
PVX_084690	0.016473156	0	1.349316306
PVX_084695	0.078805602	0	1.322621741
PVX_084698	0.359317338	0	0.339054503
PVX_084702	0.036687443	0	1.18014441
PVX_084705	0.166224591	0	0.568599725
PVX_084710	0.036687443	0	1.353906146
PVX_084715	0.007054102	0	1.607945855
PVX_084720	0.007054102	0	1.957515121
PVX_084725	0.166224591	0	0.282706033
PVX_084730	0.002855585	0	1.73970468
PVX_084735	0.002855585	0	2.599338979
PVX_084740	0.036687443	0	1.4870889
PVX_084745	0.007054102	0	1.876269557
PVX_084750	0.007054102	0	2.037834423
PVX_084755	0.359317338	0	0.164957844
PVX_084760	0.036687443	0	1.636496471
PVX_084765	0.078805602	0	1.032866104
PVX_084770	0.000383392	0	2.202373422
PVX_084775	0.359317338	0	0.211537285
PVX_084780	0.016473156	0	1.744520598
PVX_084785	0.007054102	0	2.205557088
PVX_084790	0.359317338	0	0.234197675

GeneID	Pvalue	BackGroundPre	BackGroundDx
PVX_084795	0.000383392	0	4.033262584
PVX_084800	1	0	0
PVX_084805	0.002855585	0	2.731464448
PVX_084810	0.001084333	0	1.544022455
PVX_084815	1	0	0
PVX_084820	0.001084333	0	3.751205657
PVX_084825	0.007054102	0	2.496901856
PVX_084830	0.078805602	0	0.989956322
PVX_084835	0.166224591	0	0.631188705
PVX_084837	0.036687443	0	0.730665591
PVX_084840	0.166224591	0	0.307368094
PVX_084845	0.036687443	0	1.190102057
PVX_084850	0.002855585	0	3.41782118
PVX_084855	0.001084333	0	2.280892567
PVX_084860	0.000125309	0	2.225444622
PVX_084870	0.078805602	0	0.812425761
PVX_084875	1	0	0
PVX_084885	0.000383392	0	2.679333144
PVX_084895	0.007054102	0	2.842103206
PVX_084900	1	0	0
PVX_084905	0.002855585	0	2.488946894
PVX_084910	0.078805602	0	1.129110547
PVX_084915	0.036687443	0	0.924762567
PVX_084920	0.016473156	0	1.605806483
PVX_084925	1	0	0
PVX_084930	0.036687443	0	0.951192369
PVX_084935	0.078805602	0	0.692473948
PVX_084940	0.001084333	0	3.13957464
PVX_084945	0.016473156	0	1.919965073
PVX_084950	0.166224591	0	0.472663391
PVX_084955	3.76E-05	0	6.065611216
PVX_084960	0.359317338	0	0.276228215
PVX_084965	0.166224591	0	0.413186239
PVX_084970	0.016473156	0	1.489117879
PVX_084975	0.007054102	0	1.701531251
PVX_084980	1	0	0
PVX_084985	0.166224591	0	0.614400196
PVX_084990	0.002855585	0	2.816468495
PVX_084995	0.078805602	0	1.509786879
PVX_085000	0.078805602	0	0.792912628
PVX_085005	0.001084333	0	2.801191639

GeneID	Pvalue	BackGroundPre	BackGroundDx
PVX_085010	0.007054102	0	1.709204066
PVX_085015	0.007054102	0	2.29545793
PVX_085020	0.166224591	0	0.506466749
PVX_085025	0.002855585	0	1.898941598
PVX_085030	1	0	0
PVX_085035	0.359317338	0	0.258869749
PVX_085040	1	0	0
PVX_085045	0.166224591	0	0.417757891
PVX_085050	0.000125309	0	3.642581741
PVX_085055	0.007054102	0	2.076519068
PVX_085060	0.007054102	0	2.578707339
PVX_085065	0.002855585	0	2.271308511
PVX_085070	0.036687443	0	0.66868626
PVX_085080	1	0	0
PVX_085085	0.359317338	0	0.178846513
PVX_085090	0.007054102	0	1.973977294
PVX_085095	1	0	0
PVX_085100	0.016473156	0	1.284737891
PVX_085105	0.078805602	0	1.196758861
PVX_085110	0.078805602	0	1.105640655
PVX_085115	0.036687443	0	1.411346758
PVX_085120	0.078805602	0	0.585495172
PVX_085125	0.000125309	0	4.630794187
PVX_085130	0.036687443	0	1.233699952
PVX_085135	0.359317338	0	0.307333881
PVX_085140	0.078805602	0	1.109760945
PVX_085145	3.76E-05	0	5.35703222
PVX_085150	0.036687443	0	1.268011654
PVX_085152	1	0	0
PVX_085155	0.002855585	0	2.453028416
PVX_085160	0.001084333	0	2.774368027
PVX_085165	0.001084333	0	2.872149185
PVX_085170	0.016473156	0	1.440351776
PVX_085175	0.002855585	0	2.975018313
PVX_085180	0.036687443	0	0.910010379
PVX_085185	0.078805602	0	0.859861611
PVX_085190	0.000383392	0	4.069018286
PVX_085195	0.016473156	0	1.14298426
PVX_085200	0.002855585	0	2.20329311
PVX_085203	0.007054102	0	1.473749764
PVX_085205	0.007054102	0	1.816931589

GeneID	Pvalue	BackGroundPre	BackGroundDx
PVX_085209	1	0	0
PVX_085211	0.078805602	0	0.714296567
PVX_085215	1.03E-05	0	5.195237421
PVX_085220	0.000125309	0	5.246163256
PVX_085225	3.76E-05	0	5.725149669
PVX_085230	0.016473156	0	1.907822612
PVX_085235	0.001084333	0	2.360324261
PVX_085240	0.002855585	0	2.666269397
PVX_085245	1	0	0
PVX_085250	0.166224591	0	0.828336511
PVX_085255	1	0	0
PVX_085260	0.078805602	0	1.097763954
PVX_085265	0.016473156	0	1.798673837
PVX_085270	0.000125309	0	4.803207715
PVX_085275	3.76E-05	0	6.016953602
PVX_085290	0.002855585	0	1.885147695
PVX_085295	0.000125309	0	3.203456458
PVX_085300	0.166224591	0	0.623575031
PVX_085305	0.359317338	0	0.199825364
PVX_085310	0.078805602	0	0.65234516
PVX_085315	0.359317338	0	0.295191704
PVX_085320	0.016473156	0	1.665845648
PVX_085325	0.078805602	0	0.922686081
PVX_085330	0.000383392	0	3.279396321
PVX_085335	0.359317338	0	0.221378278
PVX_085340	0.007054102	0	2.243531639
PVX_085345	0.001084333	0	2.783844311
PVX_085350	0.166224591	0	0.614670892
PVX_085355	0.016473156	0	1.587228964
PVX_085360	0.000125309	0	3.364521572
PVX_085365	0.036687443	0	1.460995253
PVX_085370	0.359317338	0	0.307005867
PVX_085375	0.007054102	0	2.405243668
PVX_085380	0.166224591	0	0.848637013
PVX_085385	0.007054102	0	2.70294076
PVX_085390	0.359317338	0	0.302059739
PVX_085395	0.016473156	0	1.641115568
PVX_085405	0.078805602	0	1.599484247
PVX_085410	0.078805602	0	1.08489382
PVX_085415	0.359317338	0	0.328664427
PVX_085420	1.03E-05	0	7.626922869

GeneID	Pvalue	BackGroundPre	BackGroundDx
PVX_085425	0.000125309	0	3.65981898
PVX_085430	0.016473156	0	2.302395017
PVX_085435	0.078805602	0	0.781603912
PVX_085440	0.359317338	0	0.316521532
PVX_085445	0.166224591	0	0.457425093
PVX_085450	0.016473156	0	1.708872991
PVX_085455	0.001084333	0	2.399637792
PVX_085460	0.002855585	0	2.350959187
PVX_085465	0.359317338	0	0.321929512
PVX_085470	0.002855585	0	1.705784512
PVX_085475	1	0	0
PVX_085480	0.002855585	0	2.756185487
PVX_085485	0.002855585	0	2.424980274
PVX_085490	0.000125309	0	4.18185074
PVX_085495	3.76E-05	0	4.601773036
PVX_085500	0.036687443	0	1.27144124
PVX_085505	1	0	0
PVX_085510	0.002855585	0	1.956486348
PVX_085515	0.036687443	0	1.59020437
PVX_085520	0.002855585	0	2.504102547
PVX_085525	0.016473156	0	1.435321626
PVX_085530	0.036687443	0	1.114647292
PVX_085535	0.078805602	0	0.783787134
PVX_085540	0.359317338	0	0.371939372
PVX_085545	0.359317338	0	0.258774579
PVX_085550	0.359317338	0	0.15095847
PVX_085555	0.016473156	0	1.783332891
PVX_085560	0.016473156	0	1.915021139
PVX_085565	0.001084333	0	2.49832687
PVX_085567	0.359317338	0	0.302757876
PVX_085570	0.016473156	0	1.142278647
PVX_085575	0.007054102	0	2.163740254
PVX_085580	0.359317338	0	0.155342078
PVX_085585	0.166224591	0	0.464450612
PVX_085590	0.000125309	0	2.094530237
PVX_085595	0.078805602	0	1.042866437
PVX_085600	0.166224591	0	0.601416228
PVX_085605	0.016473156	0	1.891065974
PVX_085610	0.002855585	0	2.043524455
PVX_085615	1	0	0
PVX_085620	1	0	0

GeneID	Pvalue	BackGroundPre	BackGroundDx
PVX_085625	0.000125309	0	4.364604296
PVX_085630	0.036687443	0	1.078349727
PVX_085635	0.359317338	0	0.263637902
PVX_085640	0.078805602	0	0.689672189
PVX_085645	0.007054102	0	1.781469157
PVX_085650	1	0	0
PVX_085655	0.078805602	0	1.284888364
PVX_085660	0.078805602	0	0.976059447
PVX_085665	0.000125309	0	4.004952406
PVX_085670	0.166224591	0	0.367591531
PVX_085675	0.036687443	0	0.857777502
PVX_085680	0.001084333	0	2.466670071
PVX_085685	0.002855585	0	2.747592387
PVX_085690	0.016473156	0	2.135202243
PVX_085695	0.166224591	0	0.592487719
PVX_085700	0.036687443	0	1.631759819
PVX_085705	0.036687443	0	1.178952505
PVX_085710	0.007054102	0	2.491132699
PVX_085715	0.036687443	0	0.944111339
PVX_085720	0.002855585	0	2.743982702
PVX_085725	0.016473156	0	1.055639726
PVX_085730	0.000125309	0	4.803854641
PVX_085735	1.03E-05	0	6.696945944
PVX_085740	0.007054102	0	2.380679194
PVX_085745	0.016473156	0	1.355706086
PVX_085750	0.007054102	0	2.478126935
PVX_085755	0.007054102	0	2.164854629
PVX_085760	0.016473156	0	1.661297202
PVX_085765	0.359317338	0	0.147827483
PVX_085770	0.359317338	0	0.300108463
PVX_085775	0.359317338	0	0.346145801
PVX_085780	0.016473156	0	1.410439703
PVX_085785	0.359317338	0	0.429396638
PVX_085790	1	0	0
PVX_085795	1	0	0
PVX_085800	1	0	0
PVX_085810	0.166224591	0	0.583037926
PVX_085815	0.016473156	0	1.848086154
PVX_085820	0.016473156	0	1.290795787
PVX_085825	0.007054102	0	1.818531345
PVX_085830	0.016473156	0	1.796994101

GeneID	Pvalue	BackGroundPre	BackGroundDx
PVX_085835	0.002855585	0	1.651749364
PVX_085840	0.001084333	0	3.760244938
PVX_085845	0.359317338	0	0.406377057
PVX_085850	0.001084333	0	2.506746585
PVX_085855	0.166224591	0	0.363754965
PVX_085860	0.166224591	0	0.642647609
PVX_085865	0.036687443	0	1.424240147
PVX_085870	0.002855585	0	2.421330556
PVX_085875	0.166224591	0	0.461281013
PVX_085877	0.007054102	0	1.691453521
PVX_085880	0.016473156	0	1.078752489
PVX_085885	0.078805602	0	1.146032893
PVX_085890	0.036687443	0	1.189870743
PVX_085895	0.016473156	0	1.635079059
PVX_085900	0.036687443	0	0.996880972
PVX_085905	0.002855585	0	2.37999569
PVX_085910	1	0	0
PVX_085915	0.036687443	0	1.228807833
PVX_085920	3.76E-05	0	4.755301476
PVX_085925	1	0	0
PVX_085930	0.359317338	0	0.330918015
PVX_085935	0.001084333	0	1.607807365
PVX_085940	0.002855585	0	2.245734508
PVX_085945	1	0	0
PVX_085950	0.007054102	0	2.481570423
PVX_085955	0.002855585	0	2.181966329
PVX_085960	0.036687443	0	1.607284729
PVX_085965	0.016473156	0	1.477548933
PVX_085970	3.76E-05	0	3.801923425
PVX_085975	0.036687443	0	1.220784094
PVX_085977	0.016473156	0	1.31454075
PVX_085980	0.359317338	0	0.377037611
PVX_085985	0.001084333	0	3.17202776
PVX_085990	0.007054102	0	2.580223599
PVX_085995	0.007054102	0	1.737929032
PVX_086000	0.016473156	0	1.73597183
PVX_086005	0.166224591	0	0.740608263
PVX_086010	1	0	0
PVX_086015	3.76E-05	0	2.372260289
PVX_086020	3.76E-05	0	6.254312741
PVX_086022	0.078805602	0	1.326196838

GeneID	Pvalue	BackGroundPre	BackGroundDx
PVX_086025	0.001084333	0	2.485050957
PVX_086030	1	0	0
PVX_086035	0.036687443	0	1.038576029
PVX_086040	0.166224591	0	0.74351249
PVX_086045	1	0	0
PVX_086050	0.166224591	0	0.310681502
PVX_086055	0.078805602	0	1.433991583
PVX_086060	0.166224591	0	0.954447733
PVX_086065	0.001084333	0	3.309406424
PVX_086070	0.359317338	0	0.282831784
PVX_086075	0.001084333	0	3.346665605
PVX_086080	0.166224591	0	0.436532103
PVX_086085	0.036687443	0	1.285615169
PVX_086090	0.078805602	0	1.077017517
PVX_086095	0.036687443	0	1.576316784
PVX_086100	0.007054102	0	1.399643789
PVX_086105	0.036687443	0	1.098239982
PVX_086110	0.002855585	0	2.466789295
PVX_086115	0.036687443	0	1.24314759
PVX_086120	1.03E-05	0	3.759867075
PVX_086125	0.007054102	0	1.60884847
PVX_086130	0.007054102	0	2.171260758
PVX_086135	0.359317338	0	0.178797387
PVX_086140	0.007054102	0	1.935911115
PVX_086145	0.002855585	0	2.273390481
PVX_086150	0.359317338	0	0.234077223
PVX_086155	0.001084333	0	3.294866647
PVX_086160	0.000383392	0	3.115432055
PVX_086165	0.007054102	0	1.65369259
PVX_086170	0.002855585	0	2.167744045
PVX_086175	1	0	0
PVX_086180	0.007054102	0	1.710900308
PVX_086185	0.001084333	0	2.899047451
PVX_086190	0.036687443	0	1.604660074
PVX_086195	0.001084333	0	2.857686387
PVX_086200	1	0	0
PVX_086205	0.359317338	0	0.340803488
PVX_086210	0.002855585	0	2.519034326
PVX_086215	0.166224591	0	0.703224753
PVX_086220	0.166224591	0	0.733016427
PVX_086225	1	0	0

GeneID	Pvalue	BackGroundPre	BackGroundDx
PVX_086230	0.036687443	0	1.713631883
PVX_086235	0.007054102	0	2.24435494
PVX_086240	0.000125309	0	4.514951341
PVX_086245	0.359317338	0	0.133902209
PVX_086250	0.166224591	0	0.545752207
PVX_086255	1	0	0
PVX_086260	0.359317338	0	0.340407641
PVX_086265	0.359317338	0	0.197919665
PVX_086268	1	0	0
PVX_086272	0.359317338	0	0.255238172
PVX_086275	0.359317338	0	0.276147311
PVX_086280	1	0	0
PVX_086285	0.166224591	0	0.494564014
PVX_086290	0.007054102	0	1.389495665
PVX_086295	0.016473156	0	1.741017234
PVX_086305	0.000125309	0	5.362347749
PVX_086310	1	0	0
PVX_086315	0.000383392	0	3.206041309
PVX_086320	1	0	0
PVX_086325	0.036687443	0	0.956782166
PVX_086330	0.000383392	0	3.440774568
PVX_086335	0.166224591	0	0.617629572
PVX_086340	0.002855585	0	2.527188004
PVX_086345	1	0	0
PVX_086350	1	0	0
PVX_086845	1	0	0
PVX_086850	1	0	0
PVX_086855	0.036687443	0	1.008943521
PVX_086860	1	0	0
PVX_086863	1	0	0
PVX_086865	1	0	0
PVX_086870	1	0	0
PVX_086875	1	0	0
PVX_086880	1	0	0
PVX_086890	0.016473156	0	1.63330088
PVX_086893	0.359317338	0	0.238654721
PVX_086895	1	0	0
PVX_086900	0.007054102	0	2.140513377
PVX_086903	1	0	0
PVX_086905	1.03E-05	0	6.0784156
PVX_086910	0.166224591	0	0.731257785

GeneID	Pvalue	BackGroundPre	BackGroundDx
PVX_086915	0.007054102	0	2.620491448
PVX_086920	1	0	0
PVX_086925	0.166224591	0	0.640795936
PVX_086930	0.359317338	0	0.266126416
PVX_086935	0.359317338	0	0.412280738
PVX_086940	0.002855585	0	2.22903699
PVX_086945	1	0	0
PVX_086950	1	0	0
PVX_086955	0.166224591	0	0.888086898
PVX_086960	0.078805602	0	0.694555648
PVX_086962	0.359317338	0	0.510898395
PVX_086965	0.016473156	0	2.33314499
PVX_086970	0.359317338	0	0.270784399
PVX_086975	0.359317338	0	0.260022886
PVX_086980	0.002855585	0	2.737979624
PVX_086985	0.359317338	0	0.372677604
PVX_086990	0.002855585	0	3.033133493
PVX_086995	0.007054102	0	2.561886235
PVX_087000	0.078805602	0	1.411810781
PVX_087005	0.078805602	0	1.213505233
PVX_087010	0.002855585	0	2.364998335
PVX_087015	0.078805602	0	0.848552056
PVX_087020	0.036687443	0	1.505436698
PVX_087025	0.016473156	0	1.780982766
PVX_087030	0.002855585	0	2.968352331
PVX_087035	0.000383392	0	3.699993497
PVX_087040	0.016473156	0	1.228850981
PVX_087042	0.078805602	0	1.238535984
PVX_087044	0.166224591	0	0.542536908
PVX_087045	0.007054102	0	2.421021563
PVX_087050	0.078805602	0	0.74687258
PVX_087055	0.078805602	0	0.834554385
PVX_087060	1	0	0
PVX_087065	0.000383392	0	3.051072455
PVX_087070	0.016473156	0	1.315882245
PVX_087075	0.036687443	0	1.553107676
PVX_087080	0.166224591	0	0.664924182
PVX_087085	0.016473156	0	1.461571244
PVX_087090	0.000383392	0	3.460460005
PVX_087095	3.76E-05	0	5.244021956
PVX_087105	0.078805602	0	0.79244934

GeneID	Pvalue	BackGroundPre	BackGroundDx
PVX_087107	0.359317338	0	0.423421891
PVX_087110	0.359317338	0	0.284035654
PVX_087115	0.000383392	0	3.894987666
PVX_087120	0.001084333	0	2.508046508
PVX_087125	1	0	0
PVX_087130	0.016473156	0	1.64428897
PVX_087135	0.000383392	0	3.666122957
PVX_087140	0.002855585	0	2.577143097
PVX_087143	0.016473156	0	2.186982947
PVX_087145	0.001084333	0	2.603065436
PVX_087150	0.078805602	0	1.07564624
PVX_087155	0.036687443	0	0.991253132
PVX_087160	0.166224591	0	0.694954064
PVX_087165	1	0	0
PVX_087665	0.000383392	0	3.538337648
PVX_087670	0.002855585	0	3.152731093
PVX_087675	3.76E-05	0	4.582990228
PVX_087680	1	0	0
PVX_087682	1	0	0
PVX_087684	1	0	0
PVX_087685	0.007054102	0	2.208149062
PVX_087690	0.166224591	0	0.406822053
PVX_087695	0.359317338	0	0.436689586
PVX_087700	0.036687443	0	1.045281124
PVX_087705	0.078805602	0	1.29999572
PVX_087710	0.007054102	0	2.019316481
PVX_087715	0.166224591	0	0.301947008
PVX_087720	1	0	0
PVX_087725	0.016473156	0	2.154943426
PVX_087730	0.078805602	0	0.653190784
PVX_087735	0.000383392	0	2.178609891
PVX_087740	0.007054102	0	1.411519072
PVX_087745	0.166224591	0	0.238407657
PVX_087750	0.007054102	0	1.77722346
PVX_087755	0.078805602	0	0.685976008
PVX_087760	0.016473156	0	1.32907076
PVX_087765	0.166224591	0	0.455079199
PVX_087775	0.001084333	0	1.558868213
PVX_087780	0.036687443	0	0.921111429
PVX_087785	0.078805602	0	0.640761439
PVX_087790	0.007054102	0	2.202244837

GeneID	Pvalue	BackGroundPre	BackGroundDx
PVX_087795	0.000383392	0	2.586620175
PVX_087800	0.078805602	0	0.746715918
PVX_087802	0.078805602	0	0.83236246
PVX_087805	0.078805602	0	0.814713107
PVX_087810	0.016473156	0	1.547098929
PVX_087815	0.002855585	0	1.558350619
PVX_087820	0.007054102	0	1.818155667
PVX_087825	0.036687443	0	2.809236279
PVX_087830	1	0	0
PVX_087835	0.001084333	0	3.675491454
PVX_087840	3.76E-05	0	3.798294378
PVX_087845	0.002855585	0	1.945467855
PVX_087851	0.166224591	0	0.965431751
PVX_087855	0.359317338	0	0.398870099
PVX_087860	0.000383392	0	5.439211849
PVX_087865	0.036687443	0	1.089926373
PVX_087870	0.078805602	0	1.023898225
PVX_087875	1	0	0
PVX_087876	1	0	0
PVX_087877	1	0	0
PVX_087878	1	0	0
PVX_087879	1	0	0
PVX_087880	0.016473156	0	1.084731415
PVX_087885	0.359317338	0	0.235132762
PVX_087895	0.002855585	0	2.374110584
PVX_087900	0.036687443	0	0.881489747
PVX_087905	0.078805602	0	1.122298678
PVX_087910	0.016473156	0	1.530738616
PVX_087915	0.002855585	0	2.148338327
PVX_087920	0.001084333	0	3.642944293
PVX_087925	0.166224591	0	0.580494694
PVX_087930	0.007054102	0	2.277435673
PVX_087935	0.166224591	0	1.151782902
PVX_087940	0.166224591	0	0.380726655
PVX_087945	0.000383392	0	3.46550786
PVX_087950	1.03E-05	0	7.118567536
PVX_087955	0.002855585	0	2.15968508
PVX_087960	0.359317338	0	0.274978055
PVX_087965	0.016473156	0	1.78265418
PVX_087970	1.03E-05	0	5.349212726
PVX_087975	0.016473156	0	2.034437937

GeneID	Pvalue	BackGroundPre	BackGroundDx
PVX_087980	0.016473156	0	1.741922738
PVX_087985	0.002855585	0	2.101474096
PVX_087990	0.166224591	0	0.570525031
PVX_087995	0.002855585	0	1.609333094
PVX_088000	0.000383392	0	2.579567641
PVX_088005	0.166224591	0	0.762275047
PVX_088007	0.002855585	0	2.071842221
PVX_088010	0.002855585	0	2.241927679
PVX_088015	0.036687443	0	1.412733806
PVX_088020	0.359317338	0	0.393391817
PVX_088025	0.036687443	0	0.778873089
PVX_088035	0.036687443	0	0.846182278
PVX_088040	0.036687443	0	1.204355116
PVX_088045	0.359317338	0	0.114192906
PVX_088050	0.036687443	0	1.543063716
PVX_088055	0.036687443	0	1.431803982
PVX_088060	0.016473156	0	2.001624178
PVX_088065	1.03E-05	0	6.304718076
PVX_088070	0.002855585	0	2.79680668
PVX_088075	0.002855585	0	2.397546786
PVX_088080	0.166224591	0	0.827391211
PVX_088085	0.007054102	0	1.492797172
PVX_088090	0.007054102	0	2.738418035
PVX_088095	0.078805602	0	0.729348128
PVX_088100	0.036687443	0	1.370228751
PVX_088105	0.001084333	0	3.023121368
PVX_088110	0.036687443	0	1.358331463
PVX_088115	0.002855585	0	3.364963849
PVX_088120	0.001084333	0	2.726828222
PVX_088125	1	0	0
PVX_088130	0.007054102	0	1.661530306
PVX_088140	0.036687443	0	1.270894525
PVX_088145	0.002855585	0	2.899153382
PVX_088150	0.002855585	0	2.823733605
PVX_088155	0.359317338	0	0.21801833
PVX_088165	0.001084333	0	2.612348319
PVX_088170	0.002855585	0	3.154959352
PVX_088175	0.002855585	0	2.967900559
PVX_088180	0.007054102	0	2.73137485
PVX_088185	0.002855585	0	2.568796209
PVX_088190	0.007054102	0	1.389272735

GeneID	Pvalue	BackGroundPre	BackGroundDx
PVX_088195	0.036687443	0	1.439006303
PVX_088200	0.007054102	0	1.982185008
PVX_088205	0.002855585	0	2.517223172
PVX_088210	0.166224591	0	0.622568847
PVX_088215	0.002855585	0	1.81740072
PVX_088220	0.002855585	0	2.260265794
PVX_088225	0.002855585	0	2.273356427
PVX_088230	0.359317338	0	0.380283058
PVX_088235	0.016473156	0	1.374177969
PVX_088240	0.359317338	0	0.254535075
PVX_088245	0.359317338	0	0.32534097
PVX_088250	0.016473156	0	1.441626709
PVX_088254	0.007054102	0	1.95511001
PVX_088256	1	0	0
PVX_088265	0.166224591	0	0.576832584
PVX_088270	0.007054102	0	2.104960662
PVX_088275	0.016473156	0	1.112476308
PVX_088280	0.036687443	0	1.441068285
PVX_088775	1	0	0
PVX_088780	0.359317338	0	0.280587006
PVX_088790	0.359317338	0	0.350097001
PVX_088795	0.166224591	0	0.563926955
PVX_088797	1	0	0
PVX_088798	1	0	0
PVX_088800	1	0	0
PVX_088805	1	0	0
PVX_088810	1	0	0
PVX_088815	0.036687443	0	1.338406387
PVX_088820	0.007054102	0	2.23831195
PVX_088825	1	0	0
PVX_088830	0.000383392	0	4.043368189
PVX_088835	0.359317338	0	0.423861872
PVX_088840	0.078805602	0	1.071430233
PVX_088845	1	0	0
PVX_088850	0.166224591	0	0.598898359
PVX_088855	0.001084333	0	3.7798988
PVX_088860	0.359317338	0	0.343048375
PVX_088865	0.001084333	0	4.256544482
PVX_088870	1	0	0
PVX_088875	1	0	0
PVX_088880	0.036687443	0	1.376439285

GeneID	Pvalue	BackGroundPre	BackGroundDx
PVX_088885	0.166224591	0	0.762236037
PVX_088890	0.036687443	0	1.527576666
PVX_088895	0.016473156	0	2.085838961
PVX_088905	0.007054102	0	1.378203855
PVX_088907	1	0	0
PVX_088910	1	0	0
PVX_088915	1	0	0
PVX_088920	1	0	0
PVX_088930	0.036687443	0	1.372355332
PVX_088935	0.007054102	0	1.906917049
PVX_088940	0.016473156	0	1.348432665
PVX_088945	0.016473156	0	1.371354641
PVX_088950	0.016473156	0	1.536708351
PVX_088955	1	0	0
PVX_088960	3.76E-05	0	5.274602363
PVX_088965	0.000125309	0	2.970193998
PVX_088970	1	0	0
PVX_088975	0.001084333	0	2.45625555
PVX_088985	0.000383392	0	3.499229002
PVX_088990	0.359317338	0	0.521809896
PVX_088995	0.036687443	0	1.146828453
PVX_089000	0.359317338	0	0.203375881
PVX_089010	0.036687443	0	0.807700648
PVX_089015	0.002855585	0	1.962876921
PVX_089020	1	0	0
PVX_089025	1.03E-05	0	6.845406967
PVX_089030	0.002855585	0	2.792616898
PVX_089035	0.002855585	0	2.441407747
PVX_089040	0.002855585	0	2.934000321
PVX_089045	0.078805602	0	0.785831481
PVX_089050	0.036687443	0	1.349684549
PVX_089055	0.000125309	0	2.396687787
PVX_089060	0.016473156	0	1.246924003
PVX_089065	0.016473156	0	1.551783269
PVX_089070	0.359317338	0	0.259683013
PVX_089075	1	0	0
PVX_089080	0.359317338	0	0.467605726
PVX_089085	0.002855585	0	2.203174924
PVX_089087	0.166224591	0	0.894009314
PVX_089090	0.016473156	0	2.182218626
PVX_089095	0.359317338	0	0.37958679

GeneID	Pvalue	BackGroundPre	BackGroundDx
PVX_089105	1	0	0
PVX_089110	0.002855585	0	1.629905139
PVX_089115	0.036687443	0	1.0930611
PVX_089120	0.007054102	0	1.38246486
PVX_089125	0.016473156	0	1.645756003
PVX_089130	0.078805602	0	0.94857843
PVX_089135	0.036687443	0	1.466682848
PVX_089140	0.007054102	0	2.109103952
PVX_089145	0.016473156	0	1.352802669
PVX_089150	0.000383392	0	2.846643557
PVX_089155	0.036687443	0	1.277193853
PVX_089160	0.359317338	0	0.226765847
PVX_089165	0.007054102	0	2.377085741
PVX_089170	0.001084333	0	3.124511318
PVX_089175	0.007054102	0	2.880548217
PVX_089180	0.166224591	0	0.646084421
PVX_089185	1	0	0
PVX_089195	0.359317338	0	0.447645753
PVX_089200	0.002855585	0	2.02985704
PVX_089205	0.016473156	0	1.992757645
PVX_089210	0.036687443	0	1.489591666
PVX_089215	0.078805602	0	0.748409261
PVX_089220	0.166224591	0	0.468205775
PVX_089225	0.000125309	0	4.194958123
PVX_089230	1	0	0
PVX_089235	0.001084333	0	2.889647704
PVX_089240	1	0	0
PVX_089245	0.166224591	0	0.542255273
PVX_089250	0.002855585	0	3.152468233
PVX_089255	0.078805602	0	0.663087951
PVX_089260	0.036687443	0	1.565754552
PVX_089265	0.016473156	0	1.547331452
PVX_089270	0.007054102	0	2.567420697
PVX_089275	0.036687443	0	1.777093736
PVX_089280	3.76E-05	0	6.108499345
PVX_089285	0.007054102	0	1.679922017
PVX_089290	0.166224591	0	0.549232378
PVX_089292	0.166224591	0	0.927021927
PVX_089295	0.007054102	0	1.946626674
PVX_089300	0.359317338	0	0.421915086
PVX_089305	0.166224591	0	0.626546968

GeneID	Pvalue	BackGroundPre	BackGroundDx
PVX_089310	0.359317338	0	0.265441185
PVX_089315	0.016473156	0	1.346652743
PVX_089320	0.001084333	0	2.525110457
PVX_089325	0.002855585	0	2.174790691
PVX_089330	0.359317338	0	0.223186975
PVX_089335	0.002855585	0	1.983802861
PVX_089340	0.166224591	0	0.541102973
PVX_089345	0.036687443	0	1.180763511
PVX_089355	0.016473156	0	1.508748006
PVX_089360	0.016473156	0	1.686922131
PVX_089365	0.007054102	0	1.619996759
PVX_089370	0.007054102	0	2.478221001
PVX_089375	0.359317338	0	0.138516444
PVX_089380	1	0	0
PVX_089385	1	0	0
PVX_089390	0.078805602	0	1.081123401
PVX_089395	1	0	0
PVX_089400	0.007054102	0	1.672619599
PVX_089405	0.359317338	0	0.199637274
PVX_089410	0.359317338	0	0.328351319
PVX_089415	0.007054102	0	1.434952777
PVX_089425	3.76E-05	0	6.46291587
PVX_089430	0.007054102	0	2.065192296
PVX_089435	0.007054102	0	2.039469688
PVX_089440	1	0	0
PVX_089445	0.016473156	0	1.809521985
PVX_089450	1	0	0
PVX_089455	0.166224591	0	0.700465225
PVX_089460	1	0	0
PVX_089465	0.166224591	0	0.595863819
PVX_089467	1	0	0
PVX_089470	0.166224591	0	0.632378828
PVX_089473	0.359317338	0	0.372546994
PVX_089475	1	0	0
PVX_089480	0.002855585	0	2.188995075
PVX_089485	0.007054102	0	2.075152235
PVX_089490	0.007054102	0	2.374960043
PVX_089495	0.078805602	0	1.403658883
PVX_089500	1	0	0
PVX_089505	0.002855585	0	3.112771358
PVX_089510	1	0	0

GeneID	Pvalue	BackGroundPre	BackGroundDx
PVX_089515	0.078805602	0	0.947964927
PVX_089520	0.359317338	0	0.431953758
PVX_089525	1	0	0
PVX_089530	0.359317338	0	0.196786011
PVX_089535	0.166224591	0	0.420811188
PVX_089540	0.002855585	0	3.230069203
PVX_089542	0.036687443	0	1.338903887
PVX_089545	0.359317338	0	0.297313039
PVX_089550	0.078805602	0	0.866625665
PVX_089555	0.016473156	0	1.396638202
PVX_089560	0.007054102	0	2.144052484
PVX_089565	0.036687443	0	1.365192859
PVX_089570	0.359317338	0	0.258299723
PVX_089575	0.166224591	0	0.792537979
PVX_089580	0.001084333	0	3.224410763
PVX_089585	0.359317338	0	0.51226456
PVX_089590	0.036687443	0	1.518076581
PVX_089592	0.359317338	0	0.177824814
PVX_089595	0.036687443	0	1.617064288
PVX_089600	0.078805602	0	1.107157017
PVX_089605	0.002855585	0	2.70213403
PVX_089610	0.359317338	0	0.200148219
PVX_089615	0.016473156	0	1.193196739
PVX_089620	0.036687443	0	1.347054767
PVX_089625	0.000383392	0	4.006218891
PVX_089630	0.166224591	0	0.61396945
PVX_089632	0.016473156	0	2.239506056
PVX_089635	0.016473156	0	2.126032907
PVX_089640	0.000125309	0	4.462351908
PVX_089645	0.016473156	0	1.435480424
PVX_089650	0.078805602	0	0.983537377
PVX_089655	0.002855585	0	2.295866996
PVX_089660	0.016473156	0	1.598833432
PVX_089665	0.078805602	0	0.637664593
PVX_089667	1	0	0
PVX_089670	1	0	0
PVX_089675	0.036687443	0	1.313221069
PVX_089680	0.166224591	0	0.663520119
PVX_089685	1	0	0
PVX_089690	0.036687443	0	1.260884348
PVX_089695	1	0	0

GeneID	Pvalue	BackGroundPre	BackGroundDx
PVX_089700	1	0	0
PVX_089705	0.007054102	0	2.326139389
PVX_089710	0.036687443	0	1.361496748
PVX_089715	0.016473156	0	1.266238049
PVX_089720	0.166224591	0	0.544025146
PVX_089725	0.007054102	0	2.502039436
PVX_089730	0.036687443	0	1.539058816
PVX_089735	0.000383392	0	3.434913919
PVX_089740	0.016473156	0	1.923267066
PVX_089745	0.166224591	0	0.510497436
PVX_089750	3.76E-05	0	5.718818206
PVX_089755	0.078805602	0	1.005190139
PVX_089760	0.166224591	0	0.575121698
PVX_089765	0.001084333	0	3.348723754
PVX_089770	0.359317338	0	0.296018599
PVX_089775	0.016473156	0	1.909390155
PVX_089780	0.078805602	0	1.399940729
PVX_089785	1	0	0
PVX_089790	0.036687443	0	1.206014826
PVX_089795	0.359317338	0	0.318417515
PVX_089800	1	0	0
PVX_089805	0.036687443	0	1.691007735
PVX_089810	0.007054102	0	2.672131184
PVX_089815	1	0	0
PVX_089820	1	0	0
PVX_089825	0.359317338	0	0.31062091
PVX_089830	0.166224591	0	0.824510767
PVX_089835	1	0	0
PVX_089840	0.016473156	0	2.132791819
PVX_089845	0.078805602	0	1.114230318
PVX_089850	0.002855585	0	2.554769725
PVX_089852	1	0	0
PVX_089855	1	0	0
PVX_089860	0.359317338	0	0.347074426
PVX_089863	0.359317338	0	0.403837624
PVX_089865	0.036687443	0	1.461010088
PVX_089867	0.359317338	0	0.347074426
PVX_089870	0.359317338	0	0.403837624
PVX_089875	1	0	0
PVX_089880	0.078805602	0	0.862569963
PVX_089885	0.359317338	0	0.404688623

GeneID	Pvalue	BackGroundPre	BackGroundDx
PVX_089890	1	0	0
PVX_089895	0.007054102	0	1.848240695
PVX_089900	0.036687443	0	1.49131314
PVX_089905	0.166224591	0	0.668404743
PVX_089910	0.001084333	0	2.284533235
PVX_089915	0.036687443	0	1.488223168
PVX_089920	0.016473156	0	1.699395047
PVX_089925	0.166224591	0	1.132814591
PVX_089930	0.016473156	0	2.188541663
PVX_089935	0.001084333	0	2.088531389
PVX_089940	0.359317338	0	0.344004846
PVX_089945	1	0	0
PVX_089950	0.001084333	0	2.922744497
PVX_089955	0.001084333	0	2.811987557
PVX_089960	0.036687443	0	0.649928946
PVX_089970	0.000383392	0	3.504259987
PVX_089972	0.359317338	0	0.173803937
PVX_089975	0.359317338	0	0.452251164
PVX_089980	0.016473156	0	1.534966957
PVX_089985	0.016473156	0	1.328294479
PVX_089990	0.007054102	0	1.033739447
PVX_089995	0.036687443	0	0.976145981
PVX_090000	0.000125309	0	4.161471752
PVX_090005	0.007054102	0	1.986841147
PVX_090010	0.166224591	0	0.736405476
PVX_090015	0.002855585	0	3.33525646
PVX_090020	0.359317338	0	0.127850827
PVX_090025	0.007054102	0	1.992245721
PVX_090030	0.359317338	0	0.308652265
PVX_090035	0.359317338	0	0.21631599
PVX_090040	0.359317338	0	0.272040316
PVX_090045	0.036687443	0	1.465767026
PVX_090050	0.016473156	0	2.424335101
PVX_090055	0.078805602	0	0.870512201
PVX_090060	0.007054102	0	1.366838569
PVX_090065	0.359317338	0	0.212142637
PVX_090070	1.03E-05	0	5.297587901
PVX_090075	0.166224591	0	0.55391035
PVX_090080	0.016473156	0	2.035698361
PVX_090085	0.000383392	0	1.687482444
PVX_090090	0.036687443	0	0.88825318

GeneID	Pvalue	BackGroundPre	BackGroundDx
PVX_090095	3.76E-05	0	4.044251439
PVX_090100	0.359317338	0	0.352184791
PVX_090105	0.166224591	0	0.738629733
PVX_090110	0.002855585	0	1.6998585
PVX_090115	0.002855585	0	2.861763645
PVX_090120	1	0	0
PVX_090125	0.036687443	0	1.258049798
PVX_090130	0.359317338	0	0.2159934
PVX_090135	1	0	0
PVX_090137	1	0	0
PVX_090140	0.166224591	0	0.829866091
PVX_090145	0.078805602	0	0.907348779
PVX_090150	0.000383392	0	2.515190664
PVX_090155	0.000383392	0	3.764037812
PVX_090160	1.03E-05	0	6.539043714
PVX_090165	0.000383392	0	2.381581869
PVX_090170	0.166224591	0	0.840365045
PVX_090175	0.007054102	0	2.39328855
PVX_090180	0.359317338	0	0.286905585
PVX_090185	0.078805602	0	1.130864342
PVX_090190	0.016473156	0	1.856944397
PVX_090195	0.166224591	0	0.775007527
PVX_090200	0.078805602	0	1.192073525
PVX_090205	1	0	0
PVX_090210	1	0	0
PVX_090215	1	0	0
PVX_090220	0.001084333	0	2.279008582
PVX_090225	0.166224591	0	0.74199989
PVX_090230	1.03E-05	0	6.982030315
PVX_090235	0.359317338	0	0.339919975
PVX_090240	1	0	0
PVX_090245	1	0	0
PVX_090250	0.002855585	0	2.80622386
PVX_090255	0.000125309	0	4.68643346
PVX_090260	0.359317338	0	0.390042462
PVX_090265	0.000383392	0	3.65011112
PVX_090270	0.166224591	0	0.786299632
PVX_090275	0.078805602	0	0.947279415
PVX_090280	0.078805602	0	1.076072392
PVX_090285	0.078805602	0	0.68833334
PVX_090290	0.359317338	0	0.176637579

GeneID	Pvalue	BackGroundPre	BackGroundDx
PVX_090293	0.359317338	0	0.364107784
PVX_090295	1	0	0
PVX_090300	0.359317338	0	0.359198913
PVX_090305	0.016473156	0	1.53943302
PVX_090310	1	0	0
PVX_090315	0.359317338	0	0.32694506
PVX_090320	0.359317338	0	0.248458881
PVX_090325	0.166224591	0	0.317904123
PVX_090330	1	0	0
PVX_090335	0.359317338	0	0.365028873
PVX_090830	0.166224591	0	0.732893575
PVX_090835	1	0	0
PVX_090840	1	0	0
PVX_090845	0.000125309	0	5.239800137
PVX_090847	1	0	0
PVX_090848	1	0	0
PVX_090850	0.078805602	0	0.814654844
PVX_090860	1	0	0
PVX_090865	0.007054102	0	1.980912301
PVX_090870	0.001084333	0	3.227954065
PVX_090876	0.002855585	0	1.682414812
PVX_090878	0.359317338	0	0.665080357
PVX_090880	0.000383392	0	2.960241891
PVX_090885	0.036687443	0	1.353616323
PVX_090890	0.002855585	0	1.995626041
PVX_090895	0.166224591	0	0.41554439
PVX_090900	3.76E-05	0	5.152751198
PVX_090905	0.359317338	0	0.205751197
PVX_090910	1	0	0
PVX_090915	0.016473156	0	2.394481046
PVX_090920	0.078805602	0	0.999360274
PVX_090925	1	0	0
PVX_090930	0.078805602	0	1.220791837
PVX_090935	0.001084333	0	3.857233881
PVX_090940	1	0	0
PVX_090945	0.359317338	0	0.282133026
PVX_090950	1.03E-05	0	6.755757547
PVX_090955	0.166224591	0	0.760382805
PVX_090960	0.016473156	0	1.770869307
PVX_090965	0.001084333	0	3.140981727
PVX_090970	0.016473156	0	1.761835883

GeneID	Pvalue	BackGroundPre	BackGroundDx
PVX_090972	0.359317338	0	0.434132498
PVX_090975	0.002855585	0	2.604280275
PVX_090980	0.016473156	0	1.69013367
PVX_090985	0.002855585	0	2.742020741
PVX_090990	0.166224591	0	0.618328312
PVX_090995	0.359317338	0	0.326199017
PVX_091000	0.001084333	0	2.143087072
PVX_091005	0.001084333	0	2.560841625
PVX_091010	0.036687443	0	1.085321586
PVX_091015	0.016473156	0	1.255915177
PVX_091020	1	0	0
PVX_091025	0.036687443	0	2.055009618
PVX_091030	0.000383392	0	3.202157407
PVX_091035	0.036687443	0	1.45181711
PVX_091040	1.03E-05	0	4.101859167
PVX_091045	0.036687443	0	1.691085736
PVX_091050	0.016473156	0	2.177669615
PVX_091055	0.036687443	0	1.171136939
PVX_091060	0.002855585	0	2.442621728
PVX_091065	0.007054102	0	1.549040492
PVX_091075	1	0	0
PVX_091080	0.001084333	0	2.6464996
PVX_091085	0.359317338	0	0.174676292
PVX_091090	0.016473156	0	1.960890922
PVX_091092	0.078805602	0	1.434436783
PVX_091095	0.001084333	0	3.193905295
PVX_091100	0.007054102	0	2.487428117
PVX_091105	3.76E-05	0	5.094364781
PVX_091110	0.001084333	0	3.259104213
PVX_091115	0.359317338	0	0.410028819
PVX_091120	0.036687443	0	1.39911496
PVX_091136	0.166224591	0	0.496977008
PVX_091137	0.359317338	0	0.285527952
PVX_091140	0.002855585	0	2.472311931
PVX_091145	3.76E-05	0	5.954106202
PVX_091150	0.166224591	0	0.820150496
PVX_091155	0.359317338	0	0.248985928
PVX_091160	0.000383392	0	2.532816558
PVX_091165	0.078805602	0	1.09985978
PVX_091170	3.76E-05	0	3.974490119
PVX_091175	0.002855585	0	2.026181194

GeneID	Pvalue	BackGroundPre	BackGroundDx
PVX_091180	0.078805602	0	0.999176329
PVX_091185	1	0	0
PVX_091190	1	0	0
PVX_091195	0.016473156	0	1.728350449
PVX_091200	0.036687443	0	1.232911548
PVX_091205	0.007054102	0	2.637567059
PVX_091210	0.078805602	0	1.00654364
PVX_091215	0.166224591	0	0.764644639
PVX_091220	0.078805602	0	1.368921342
PVX_091225	1	0	0
PVX_091230	1	0	0
PVX_091235	0.016473156	0	1.37507077
PVX_091240	0.007054102	0	2.50800055
PVX_091245	0.359317338	0	0.317178715
PVX_091250	0.078805602	0	1.297724741
PVX_091255	0.359317338	0	0.216948195
PVX_091260	0.036687443	0	1.431977813
PVX_091265	0.016473156	0	1.408847451
PVX_091270	0.078805602	0	1.244908471
PVX_091275	3.76E-05	0	4.764883323
PVX_091280	0.016473156	0	1.364557418
PVX_091285	0.078805602	0	0.792600478
PVX_091290	0.166224591	0	0.578537306
PVX_091295	0.359317338	0	0.253886208
PVX_091300	0.359317338	0	0.060736469
PVX_091305	0.078805602	0	1.142536137
PVX_091307	0.166224591	0	1.180034765
PVX_091310	0.007054102	0	2.473204109
PVX_091315	0.002855585	0	2.874145561
PVX_091320	0.016473156	0	1.625199062
PVX_091325	0.166224591	0	0.606228598
PVX_091330	0.000383392	0	3.761905631
PVX_091335	0.078805602	0	0.826033712
PVX_091340	0.166224591	0	0.610693904
PVX_091345	1	0	0
PVX_091350	0.359317338	0	0.294987138
PVX_091355	0.002855585	0	2.267809934
PVX_091360	0.036687443	0	1.182198043
PVX_091365	0.166224591	0	0.682479377
PVX_091370	1	0	0
PVX_091375	0.001084333	0	3.010270993

GeneID	Pvalue	BackGroundPre	BackGroundDx
PVX_091380	0.359317338	0	0.301562444
PVX_091385	0.359317338	0	0.199024107
PVX_091390	0.016473156	0	2.149326396
PVX_091395	1	0	0
PVX_091400	1	0	0
PVX_091405	0.007054102	0	2.261023886
PVX_091410	0.000125309	0	4.21432814
PVX_091415	0.000125309	0	4.300044746
PVX_091420	0.166224591	0	0.697221242
PVX_091425	0.000383392	0	4.31760171
PVX_091430	0.359317338	0	0.275057951
PVX_091434	0.359317338	0	0.237572843
PVX_091436	0.001084333	0	2.280091767
PVX_091440	0.036687443	0	1.811340993
PVX_091445	0.078805602	0	1.429668389
PVX_091450	0.000383392	0	3.595655563
PVX_091455	0.036687443	0	1.504734434
PVX_091460	0.036687443	0	1.135753003
PVX_091465	0.007054102	0	2.205855065
PVX_091470	0.007054102	0	2.213554334
PVX_091475	1	0	0
PVX_091480	0.007054102	0	1.887820133
PVX_091485	3.76E-05	0	4.155431938
PVX_091490	0.078805602	0	0.801528806
PVX_091495	0.002855585	0	3.809899957
PVX_091500	0.002855585	0	2.982097314
PVX_091505	0.007054102	0	1.89513946
PVX_091510	0.359317338	0	0.342636868
PVX_091515	0.001084333	0	4.37751601
PVX_091520	1	0	0
PVX_091525	0.036687443	0	1.32100259
PVX_091530	0.016473156	0	1.068754434
PVX_091535	0.359317338	0	0.365360437
PVX_091540	0.078805602	0	1.302677514
PVX_091545	0.000383392	0	3.464077272
PVX_091550	0.002855585	0	2.420928677
PVX_091555	0.078805602	0	1.182203223
PVX_091560	0.000383392	0	3.564123029
PVX_091565	0.016473156	0	1.501382715
PVX_091570	1	0	0
PVX_091575	1	0	0

GeneID	Pvalue	BackGroundPre	BackGroundDx
PVX_091580	1	0	0
PVX_091585	0.007054102	0	2.117492695
PVX_091590	0.007054102	0	2.146832084
PVX_091595	0.078805602	0	1.153775526
PVX_091600	0.001084333	0	3.225317617
PVX_091605	3.76E-05	0	3.548968115
PVX_091610	0.001084333	0	3.0854626
PVX_091615	0.002855585	0	2.032441087
PVX_091620	1	0	0
PVX_091625	0.078805602	0	1.164326779
PVX_091630	0.016473156	0	0.965966042
PVX_091635	0.002855585	0	2.493500062
PVX_091640	3.76E-05	0	6.486364862
PVX_091645	1	0	0
PVX_091650	0.166224591	0	0.718735505
PVX_091652	0.007054102	0	2.314220778
PVX_091655	3.76E-05	0	3.989718697
PVX_091660	0.359317338	0	0.129460638
PVX_091662	0.166224591	0	0.400746861
PVX_091664	1	0	0
PVX_091665	1	0	0
PVX_091670	0.359317338	0	0.379318952
PVX_091675	0.007054102	0	1.516850423
PVX_091680	0.166224591	0	0.737222144
PVX_091685	1	0	0
PVX_091690	1	0	0
PVX_091695	0.359317338	0	0.273348469
PVX_091700	0.000125309	0	4.2057804
PVX_091705	0.000125309	0	3.57157852
PVX_091710	1	0	0
PVX_091715	0.359317338	0	0.313707697
PVX_091720	0.166224591	0	0.591788037
PVX_091725	0.359317338	0	0.275984357
PVX_091730	0.036687443	0	1.05856283
PVX_091735	0.078805602	0	0.857332642
PVX_091740	0.078805602	0	0.571156195
PVX_091745	0.166224591	0	0.405084885
PVX_091750	0.359317338	0	0.22149226
PVX_091752	0.078805602	0	1.044572902
PVX_091755	0.078805602	0	0.834394428
PVX_091760	0.036687443	0	0.414740772

GeneID	Pvalue	BackGroundPre	BackGroundDx
PVX_091765	0.002855585	0	1.965273738
PVX_091770	0.078805602	0	0.690438621
PVX_091775	0.166224591	0	0.463577564
PVX_091780	0.001084333	0	3.031501667
PVX_091785	0.001084333	0	2.900541195
PVX_091790	0.007054102	0	1.761460355
PVX_091795	0.001084333	0	2.774542854
PVX_091800	0.166224591	0	1.149472781
PVX_091805	0.016473156	0	1.430238354
PVX_091810	0.016473156	0	1.883912766
PVX_091815	0.007054102	0	2.314860221
PVX_091820	0.016473156	0	1.156475446
PVX_091825	0.016473156	0	1.745067971
PVX_091830	0.166224591	0	0.642105765
PVX_091835	0.166224591	0	1.018856758
PVX_091840	1	0	0
PVX_091845	0.036687443	0	1.305954112
PVX_091850	0.016473156	0	2.037068149
PVX_091855	0.007054102	0	2.21139452
PVX_091860	0.000125309	0	3.326866614
PVX_091865	3.76E-05	0	5.857072982
PVX_091870	0.359317338	0	0.329947519
PVX_091875	0.007054102	0	2.154510742
PVX_091880	0.078805602	0	1.175875744
PVX_091885	0.000383392	0	2.666184471
PVX_091890	0.016473156	0	1.722185144
PVX_091895	0.078805602	0	1.088005837
PVX_091900	0.359317338	0	0.520096704
PVX_091905	0.078805602	0	0.834127418
PVX_091910	0.359317338	0	0.213060922
PVX_091915	0.016473156	0	2.178122531
PVX_091920	3.76E-05	0	4.368574911
PVX_091922	0.036687443	0	1.022439044
PVX_091925	1.03E-05	0	6.988998108
PVX_091930	0.166224591	0	0.634483674
PVX_091935	0.007054102	0	2.349381642
PVX_091940	0.001084333	0	2.496210009
PVX_091945	1	0	0
PVX_091950	0.166224591	0	0.460106832
PVX_091955	0.166224591	0	0.526634918
PVX_091960	0.166224591	0	1.133632822

GeneID	Pvalue	BackGroundPre	BackGroundDx
PVX_091965	0.007054102	0	2.637795606
PVX_091970	0.000383392	0	3.743711315
PVX_091975	0.078805602	0	1.317215685
PVX_091980	0.036687443	0	1.304553894
PVX_091985	0.078805602	0	0.889291943
PVX_091990	0.016473156	0	1.708967925
PVX_091992	0.036687443	0	1.655956682
PVX_091995	1	0	0
PVX_092000	0.007054102	0	1.692307313
PVX_092005	0.359317338	0	0.290022276
PVX_092010	0.001084333	0	2.066469204
PVX_092015	1	0	0
PVX_092025	0.002855585	0	3.025634469
PVX_092030	0.036687443	0	2.138834677
PVX_092035	0.078805602	0	0.496473183
PVX_092040	0.001084333	0	3.187105176
PVX_092045	0.002855585	0	2.119161595
PVX_092050	0.166224591	0	0.525759436
PVX_092055	0.359317338	0	0.27828215
PVX_092060	1	0	0
PVX_092065	0.000383392	0	4.085190244
PVX_092070	1.03E-05	0	5.855544038
PVX_092075	0.000383392	0	3.877584904
PVX_092080	1	0	0
PVX_092085	0.007054102	0	2.279602095
PVX_092090	0.016473156	0	1.313777762
PVX_092095	0.166224591	0	0.579222301
PVX_092100	0.166224591	0	0.712409019
PVX_092105	0.002855585	0	2.539198743
PVX_092110	0.016473156	0	1.739965509
PVX_092115	0.000383392	0	5.915222234
PVX_092120	1.03E-05	0	6.406632037
PVX_092125	0.002855585	0	2.962752872
PVX_092130	0.078805602	0	0.758698354
PVX_092135	0.078805602	0	0.95558997
PVX_092140	0.002855585	0	1.732043898
PVX_092145	1	0	0
PVX_092150	0.036687443	0	0.86048253
PVX_092155	0.078805602	0	1.195109795
PVX_092160	0.359317338	0	0.221830365
PVX_092165	0.166224591	0	0.719936844

GeneID	Pvalue	BackGroundPre	BackGroundDx
PVX_092170	0.166224591	0	0.534912085
PVX_092175	0.016473156	0	1.146814027
PVX_092180	1	0	0
PVX_092185	0.166224591	0	0.380869885
PVX_092190	1	0	0
PVX_092195	0.016473156	0	1.490665332
PVX_092200	0.078805602	0	1.068993794
PVX_092205	0.002855585	0	2.068979868
PVX_092210	1	0	0
PVX_092215	3.76E-05	0	4.519659851
PVX_092220	0.078805602	0	1.134665776
PVX_092225	0.001084333	0	2.360244426
PVX_092230	0.166224591	0	0.511972047
PVX_092235	0.016473156	0	1.542095555
PVX_092240	0.016473156	0	2.013020422
PVX_092245	0.166224591	0	0.672882158
PVX_092250	0.078805602	0	1.028419399
PVX_092260	0.000383392	0	2.624108077
PVX_092265	0.002855585	0	1.75729156
PVX_092270	0.359317338	0	0.227194611
PVX_092275	1	0	0
PVX_092280	0.007054102	0	2.075899826
PVX_092285	0.078805602	0	0.892298888
PVX_092290	0.007054102	0	1.662583226
PVX_092300	0.007054102	0	1.707692471
PVX_092305	0.007054102	0	1.342131755
PVX_092310	3.76E-05	0	4.24904162
PVX_092315	0.001084333	0	3.033062485
PVX_092320	0.001084333	0	2.063830433
PVX_092325	0.078805602	0	0.4113988
PVX_092330	0.359317338	0	0.336008949
PVX_092335	0.036687443	0	0.866443682
PVX_092340	1	0	0
PVX_092345	0.016473156	0	1.41566809
PVX_092350	0.001084333	0	2.950409264
PVX_092355	1	0	0
PVX_092360	1	0	0
PVX_092365	0.001084333	0	3.207523639
PVX_092370	0.359317338	0	0.16952906
PVX_092375	0.007054102	0	2.06157574
PVX_092380	1	0	0

GeneID	Pvalue	BackGroundPre	BackGroundDx
PVX_092385	0.000383392	0	3.920854471
PVX_092390	0.002855585	0	2.657917628
PVX_092395	0.002855585	0	1.801379489
PVX_092400	0.166224591	0	1.23568903
PVX_092405	0.359317338	0	0.405193407
PVX_092410	0.001084333	0	3.15238878
PVX_092415	0.166224591	0	0.216326984
PVX_092420	0.166224591	0	0.539831889
PVX_092425	1	0	0
PVX_092430	0.002855585	0	2.252590777
PVX_092435	3.76E-05	0	3.872793443
PVX_092440	0.001084333	0	2.600646586
PVX_092445	0.036687443	0	0.885943209
PVX_092450	0.359317338	0	0.24201056
PVX_092455	0.359317338	0	0.357487015
PVX_092460	0.078805602	0	0.570014557
PVX_092465	0.016473156	0	1.755568317
PVX_092470	0.166224591	0	0.683827984
PVX_092475	1	0	0
PVX_092480	0.000125309	0	3.738804814
PVX_092485	0.007054102	0	2.148949346
PVX_092490	0.166224591	0	0.877826274
PVX_092495	1	0	0
PVX_092500	1	0	0
PVX_092505	1	0	0
PVX_092510	0.359317338	0	0.183901809
PVX_092515	0.359317338	0	0.093124138
PVX_092520	0.016473156	0	2.224494652
PVX_092525	1	0	0
PVX_092530	0.078805602	0	0.926612606
PVX_092535	0.359317338	0	0.11625734
PVX_092540	0.000125309	0	3.844884438
PVX_092545	0.078805602	0	1.001497329
PVX_092550	0.016473156	0	1.194194398
PVX_092555	0.007054102	0	1.494298864
PVX_092560	1	0	0
PVX_092565	0.078805602	0	1.118400698
PVX_092570	0.002855585	0	1.708579869
PVX_092575	0.359317338	0	0.258329762
PVX_092580	0.359317338	0	0.199130129
PVX_092585	0.016473156	0	1.252153531

GeneID	Pvalue	BackGroundPre	BackGroundDx
PVX_092590	0.016473156	0	1.083429779
PVX_092595	0.036687443	0	1.530334314
PVX_092600	0.016473156	0	2.10211737
PVX_092605	0.000383392	0	3.447250738
PVX_092610	0.036687443	0	1.568300252
PVX_092615	1	0	0
PVX_092620	1	0	0
PVX_092625	0.359317338	0	0.222102764
PVX_092630	0.000125309	0	2.797412254
PVX_092635	0.002855585	0	2.334095557
PVX_092640	0.166224591	0	0.401701709
PVX_092650	1	0	0
PVX_092655	0.359317338	0	0.098410778
PVX_092660	0.166224591	0	0.918140394
PVX_092665	1	0	0
PVX_092670	0.359317338	0	0.326544646
PVX_092675	0.007054102	0	2.531825069
PVX_092680	0.359317338	0	0.274005501
PVX_092685	0.078805602	0	1.096746166
PVX_092690	0.002855585	0	2.627831827
PVX_092695	1	0	0
PVX_092700	0.359317338	0	0.307678884
PVX_092705	1	0	0
PVX_092710	0.000125309	0	2.644995044
PVX_092715	0.359317338	0	0.394823437
PVX_092720	0.166224591	0	0.295683405
PVX_092725	0.007054102	0	2.168415705
PVX_092730	0.000125309	0	5.111240272
PVX_092735	0.000125309	0	5.263875876
PVX_092740	0.036687443	0	1.090202711
PVX_092745	0.001084333	0	3.002210879
PVX_092750	0.359317338	0	0.349148753
PVX_092755	0.007054102	0	2.077223402
PVX_092760	0.359317338	0	0.11980419
PVX_092765	0.007054102	0	2.232276148
PVX_092770	0.016473156	0	1.852727934
PVX_092775	0.001084333	0	3.460577365
PVX_092780	0.359317338	0	0.274235981
PVX_092785	0.359317338	0	0.245684234
PVX_092790	0.359317338	0	0.183800869
PVX_092795	0.016473156	0	1.787809382

GeneID	Pvalue	BackGroundPre	BackGroundDx
PVX_092800	0.036687443	0	1.250052668
PVX_092805	3.76E-05	0	5.95442617
PVX_092810	0.078805602	0	1.242742473
PVX_092815	1	0	0
PVX_092820	1.03E-05	0	7.746088812
PVX_092825	0.036687443	0	0.873190864
PVX_092830	0.007054102	0	2.060287307
PVX_092835	0.036687443	0	1.43683629
PVX_092840	0.078805602	0	1.035840204
PVX_092845	0.359317338	0	0.241288233
PVX_092850	0.036687443	0	1.649073891
PVX_092855	1	0	0
PVX_092860	0.000383392	0	3.245117116
PVX_092865	1	0	0
PVX_092870	0.078805602	0	1.298562796
PVX_092875	0.002855585	0	2.463600145
PVX_092880	0.007054102	0	1.908397453
PVX_092885	0.078805602	0	0.776116829
PVX_092890	0.166224591	0	0.697749165
PVX_092895	0.016473156	0	1.521210642
PVX_092897	1	0	0
PVX_092900	0.016473156	0	1.735551516
PVX_092905	1	0	0
PVX_092910	0.036687443	0	1.737854777
PVX_092915	1	0	0
PVX_092920	1	0	0
PVX_092925	0.078805602	0	0.785510139
PVX_092930	1	0	0
PVX_092935	0.359317338	0	0.094626887
PVX_092940	0.016473156	0	1.765566869
PVX_092945	0.359317338	0	0.086794195
PVX_092947	1	0	0
PVX_092950	0.359317338	0	0.240195432
PVX_092955	0.359317338	0	0.137457741
PVX_092958	0.078805602	0	1.961325546
PVX_092962	0.016473156	0	1.375315117
PVX_092965	0.016473156	0	1.607898718
PVX_092970	0.036687443	0	1.36232234
PVX_092975	0.359317338	0	0.119746976
PVX_092980	0.166224591	0	0.719134284
PVX_092985	0.016473156	0	1.320281341

GeneID	Pvalue	BackGroundPre	BackGroundDx
PVX_092990	0.000125309	0	3.546262994
PVX_092995	0.002855585	0	3.028428781
PVX_093495	1	0	0
PVX_093500	1	0	0
PVX_093505	1	0	0
PVX_093510	0.000125309	0	3.63696869
PVX_093515	0.007054102	0	2.208133517
PVX_093520	0.007054102	0	2.675175228
PVX_093525	0.166224591	0	0.267940365
PVX_093530	0.007054102	0	1.913269021
PVX_093535	0.016473156	0	1.011731133
PVX_093540	0.007054102	0	1.853171226
PVX_093545	0.007054102	0	2.030242222
PVX_093550	0.016473156	0	1.619267103
PVX_093555	3.76E-05	0	5.188985455
PVX_093560	0.007054102	0	2.2074767
PVX_093565	0.016473156	0	1.212085152
PVX_093570	1	0	0
PVX_093575	0.036687443	0	1.010099453
PVX_093580	0.007054102	0	2.068542501
PVX_093585	0.166224591	0	0.568541773
PVX_093590	0.000383392	0	3.076652566
PVX_093595	0.007054102	0	2.202532586
PVX_093600	0.359317338	0	0.288523851
PVX_093605	0.016473156	0	1.697348839
PVX_093607	0.166224591	0	0.694313767
PVX_093610	0.359317338	0	0.237596439
PVX_093615	0.036687443	0	1.058381133
PVX_093620	0.036687443	0	1.411293952
PVX_093625	0.001084333	0	2.523722557
PVX_093630	0.001084333	0	3.821223316
PVX_093635	0.007054102	0	1.198124236
PVX_093640	0.007054102	0	1.745511269
PVX_093645	0.002855585	0	1.481678397
PVX_093650	0.036687443	0	1.151336516
PVX_093655	0.078805602	0	0.605047097
PVX_093660	0.007054102	0	2.262625081
PVX_093665	0.002855585	0	2.449620494
PVX_093670	1	0	0
PVX_093675	1	0	0
PVX_093680	1.42E-05	0.300125289	6.794770629

GeneID	Pvalue	BackGroundPre	BackGroundDx
PVX_093682	1	0	0
PVX_093685	1.03E-05	0	5.710927703
PVX_093695	0.078805602	0	1.214206163
PVX_093700	0.078805602	0	1.099057258
PVX_093705	0.036687443	0	1.365826711
PVX_093710	0.359317338	0	0.384417944
PVX_093715	0.359317338	0	0.313604098
PVX_093720	1	0	0
PVX_093725	1	0	0
PVX_093730	0.359317338	0	0.315373186
PVX_093735	1	0	0
PVX_094230	0.078805602	0	0.407094425
PVX_094235	1	0	0
PVX_094240	1	0	0
PVX_094243	1	0	0
PVX_094245	0.359317338	0	0.241889463
PVX_094247	1	0	0
PVX_094250	1	0	0
PVX_094255	0.359317338	0	0.136178194
PVX_094260	0.359317338	0	0.351828337
PVX_094265	0.078805602	0	0.516220549
PVX_094270	0.359317338	0	0.367246723
PVX_094275	0.000125309	0	4.309018959
PVX_094277	0.002855585	0	2.514301586
PVX_094280	0.007054102	0	2.219668911
PVX_094285	0.007054102	0	2.345579629
PVX_094290	0.016473156	0	1.895967785
PVX_094295	0.078805602	0	1.166714401
PVX_094300	3.76E-05	0	3.860194854
PVX_094303	3.76E-05	0	5.011809792
PVX_094305	0.359317338	0	0.281108206
PVX_094310	1	0	0
PVX_094315	0.001084333	0	3.563958997
PVX_094325	0.166224591	0	0.774418315
PVX_094330	0.016473156	0	1.616978778
PVX_094335	0.016473156	0	1.519886951
PVX_094340	0.007054102	0	1.5435507
PVX_094345	0.036687443	0	1.691122503
PVX_094350	0.036687443	0	0.838561738
PVX_094355	1	0	0
PVX_094360	0.078805602	0	1.268275916

GeneID	Pvalue	BackGroundPre	BackGroundDx
PVX_094365	0.359317338	0	0.33707562
PVX_094370	0.359317338	0	0.218017721
PVX_094375	0.001084333	0	3.946822144
PVX_094380	0.359317338	0	0.290607199
PVX_094385	0.002855585	0	1.9847855
PVX_094390	0.001084333	0	2.31727607
PVX_094395	0.359317338	0	0.290221974
PVX_094400	0.000383392	0	4.601195065
PVX_094405	0.002855585	0	1.504005352
PVX_094410	0.359317338	0	0.155160878
PVX_094415	0.000125309	0	4.049251814
PVX_094420	0.166224591	0	0.626171121
PVX_094425	0.359317338	0	0.286500411
PVX_094430	0.359317338	0	0.284832958
PVX_094435	0.078805602	0	0.934379266
PVX_094440	0.078805602	0	1.005975331
PVX_094445	0.002855585	0	2.256577415
PVX_094450	0.016473156	0	1.452831829
PVX_094455	0.359317338	0	0.345162889
PVX_094460	1	0	0
PVX_094462	1	0	0
PVX_094465	0.016473156	0	1.315019393
PVX_094470	0.078805602	0	0.982451002
PVX_094480	0.166224591	0	0.528099122
PVX_094485	1	0	0
PVX_094490	0.078805602	0	1.066950526
PVX_094495	1	0	0
PVX_094500	1	0	0
PVX_094505	0.007054102	0	2.48365692
PVX_094510	1	0	0
PVX_094515	0.166224591	0	0.517649329
PVX_094520	0.359317338	0	0.48540113
PVX_094525	0.016473156	0	2.174543065
PVX_094530	0.000383392	0	2.610349997
PVX_094535	1.03E-05	0	6.043139051
PVX_094540	0.359317338	0	0.310691038
PVX_094545	0.078805602	0	0.951793449
PVX_094550	0.359317338	0	0.352546152
PVX_094555	1	0	0
PVX_094560	1	0	0
PVX_094565	0.007054102	0	2.295133448

GeneID	Pvalue	BackGroundPre	BackGroundDx
PVX_094570	0.078805602	0	1.332568011
PVX_094575	0.016473156	0	2.489120829
PVX_094580	0.007054102	0	1.513722431
PVX_094585	0.166224591	0	0.552903699
PVX_094590	0.002855585	0	2.820703012
PVX_094595	0.007054102	0	1.417845776
PVX_094600	0.000383392	0	2.326802184
PVX_094605	0.036687443	0	1.630439689
PVX_094610	0.359317338	0	0.357474564
PVX_094615	0.000125309	0	4.229578195
PVX_094620	0.078805602	0	0.751738135
PVX_094625	1	0	0
PVX_094635	0.000125309	0	4.236891681
PVX_094640	0.007054102	0	2.212443495
PVX_094645	0.000125309	0	4.596915738
PVX_094650	1	0	0
PVX_094655	0.078805602	0	1.027263768
PVX_094660	0.002855585	0	2.850374138
PVX_094665	0.016473156	0	1.601679762
PVX_094670	1	0	0
PVX_094675	0.001084333	0	2.565019708
PVX_094680	0.016473156	0	1.780042657
PVX_094685	0.007054102	0	2.227374662
PVX_094690	0.016473156	0	1.835131343
PVX_094695	0.166224591	0	0.594394053
PVX_094700	1	0	0
PVX_094705	0.078805602	0	0.735009603
PVX_094710	0.166224591	0	0.345609788
PVX_094715	0.002855585	0	2.377403902
PVX_094720	0.036687443	0	1.449931066
PVX_094725	0.166224591	0	0.60161119
PVX_094730	0.002855585	0	1.874581611
PVX_094735	0.016473156	0	2.245396568
PVX_094740	1	0	0
PVX_094745	1	0	0
PVX_094750	0.000125309	0	3.965566489
PVX_094755	0.007054102	0	2.050788036
PVX_094760	0.002855585	0	2.81007929
PVX_094765	0.036687443	0	1.500139882
PVX_094770	1	0	0
PVX_094775	0.166224591	0	0.593593777

GeneID	Pvalue	BackGroundPre	BackGroundDx
PVX_094780	0.078805602	0	1.012063657
PVX_094785	0.078805602	0	1.038546796
PVX_094790	0.000383392	0	4.433848193
PVX_094795	0.166224591	0	0.807638043
PVX_094800	0.078805602	0	0.801418903
PVX_094805	0.002855585	0	2.515484156
PVX_094810	0.000125309	0	3.518771143
PVX_094815	0.078805602	0	1.061655671
PVX_094820	0.078805602	0	0.772726182
PVX_094825	0.078805602	0	1.027388658
PVX_094830	1	0	0
PVX_094835	3.76E-05	0	4.210853962
PVX_094840	3.76E-05	0	5.895607161
PVX_094845	0.016473156	0	1.471077862
PVX_094850	0.001084333	0	2.943626387
PVX_094855	0.002855585	0	1.807890467
PVX_094860	0.007054102	0	1.994169929
PVX_094865	3.76E-05	0	4.062733236
PVX_094870	0.166224591	0	0.544844499
PVX_094875	0.002855585	0	1.940043402
PVX_094880	0.036687443	0	0.949110061
PVX_094885	0.000125309	0	3.622389217
PVX_094890	1	0	0
PVX_094895	0.007054102	0	1.514945076
PVX_094900	0.166224591	0	0.404521041
PVX_094902	0.359317338	0	0.371485607
PVX_094905	1	0	0
PVX_094910	0.016473156	0	1.310562323
PVX_094915	1	0	0
PVX_094920	0.166224591	0	0.630033816
PVX_094925	0.166224591	0	0.44782788
PVX_094930	0.002855585	0	1.919687935
PVX_094935	0.078805602	0	1.050183751
PVX_094940	0.078805602	0	0.580035432
PVX_094945	0.000125309	0	2.895488927
PVX_094950	0.001084333	0	3.25936136
PVX_094955	1	0	0
PVX_094965	1	0	0
PVX_094970	0.078805602	0	1.145388207
PVX_094975	0.166224591	0	0.657834821
PVX_094980	0.001084333	0	3.047089466

GeneID	Pvalue	BackGroundPre	BackGroundDx
PVX_094985	0.002855585	0	2.783016677
PVX_094990	0.002855585	0	2.181110558
PVX_094995	0.166224591	0	0.681746083
PVX_095000	0.007054102	0	2.332082318
PVX_095005	0.078805602	0	0.938988793
PVX_095010	0.016473156	0	1.840971178
PVX_095015	1.03E-05	0	8.062592305
PVX_095020	1	0	0
PVX_095025	0.078805602	0	1.008511356
PVX_095030	0.000383392	0	3.571356864
PVX_095035	0.166224591	0	0.429281342
PVX_095040	0.002855585	0	1.980705225
PVX_095045	0.000125309	0	3.536741435
PVX_095050	0.000383392	0	3.440258561
PVX_095055	0.166224591	0	0.355818522
PVX_095060	0.036687443	0	1.192481235
PVX_095065	0.166224591	0	0.512022294
PVX_095070	0.036687443	0	1.23710345
PVX_095075	0.016473156	0	2.34904773
PVX_095080	1.03E-05	0	7.056169106
PVX_095085	1	0	0
PVX_095095	0.036687443	0	0.662004558
PVX_095100	0.359317338	0	0.183392652
PVX_095105	0.016473156	0	1.891684062
PVX_095115	0.016473156	0	1.3890464
PVX_095120	0.016473156	0	1.675173555
PVX_095125	1	0	0
PVX_095130	0.036687443	0	1.348803282
PVX_095135	3.76E-05	0	5.764390871
PVX_095140	0.007054102	0	1.540073927
PVX_095145	0.007054102	0	1.310820091
PVX_095150	0.002855585	0	1.847174016
PVX_095155	0.016473156	0	1.195949376
PVX_095160	0.000383392	0	3.287167226
PVX_095165	0.359317338	0	0.320196123
PVX_095170	0.007054102	0	1.854203258
PVX_095175	0.036687443	0	1.320728569
PVX_095180	0.016473156	0	1.35110392
PVX_095185	0.078805602	0	0.895080231
PVX_095190	0.000125309	0	5.14460088
PVX_095195	0.036687443	0	1.276504959

GeneID	Pvalue	BackGroundPre	BackGroundDx
PVX_095200	0.016473156	0	1.71258868
PVX_095205	0.000125309	0	4.10878804
PVX_095210	0.002855585	0	2.876664665
PVX_095215	0.166224591	0	0.336002124
PVX_095220	3.76E-05	0	4.512898951
PVX_095225	1	0	0
PVX_095230	0.007054102	0	2.182281767
PVX_095235	0.000383392	0	3.968600607
PVX_095240	0.036687443	0	1.454634475
PVX_095245	0.078805602	0	0.894070929
PVX_095250	0.002855585	0	1.706657951
PVX_095255	0.016473156	0	2.735298065
PVX_095260	0.007054102	0	2.036499257
PVX_095265	0.001084333	0	2.111441555
PVX_095270	0.078805602	0	0.909813928
PVX_095275	0.002855585	0	2.441475159
PVX_095280	0.036687443	0	1.599115982
PVX_095285	0.359317338	0	0.206095508
PVX_095290	0.002855585	0	2.719017437
PVX_095295	0.036687443	0	1.489945285
PVX_095300	0.036687443	0	1.126237735
PVX_095305	0.166224591	0	0.195378852
PVX_095310	1	0	0
PVX_095315	0.002855585	0	2.113425886
PVX_095320	3.76E-05	0	3.161078624
PVX_095325	0.007054102	0	2.354911455
PVX_095330	0.166224591	0	0.595988007
PVX_095335	0.166224591	0	0.505799136
PVX_095340	0.016473156	0	1.967161707
PVX_095345	0.002855585	0	1.753315668
PVX_095350	3.76E-05	0	5.902491709
PVX_095355	0.166224591	0	0.550755711
PVX_095360	0.166224591	0	0.669820151
PVX_095365	0.000383392	0	2.148489708
PVX_095370	0.016473156	0	1.380507623
PVX_095375	0.078805602	0	0.872414776
PVX_095380	0.000125309	0	4.588570419
PVX_095385	0.016473156	0	1.710697082
PVX_095390	0.000125309	0	5.84289657
PVX_095395	1	0	0
PVX_095400	0.016473156	0	2.030704749

GeneID	Pvalue	BackGroundPre	BackGroundDx
PVX_095405	0.002855585	0	2.784228209
PVX_095410	0.001084333	0	3.129905123
PVX_095415	0.016473156	0	1.687908352
PVX_095420	3.76E-05	0	4.462576811
PVX_095425	0.036687443	0	1.191951454
PVX_095430	0.007054102	0	2.509965831
PVX_095435	1	0	0
PVX_095440	0.166224591	0	0.699943864
PVX_095445	0.016473156	0	1.682739838
PVX_095450	0.359317338	0	0.205756842
PVX_095452	0.007054102	0	1.547596793
PVX_095455	0.036687443	0	1.559788244
PVX_095460	0.016473156	0	2.09510432
PVX_095470	0.007054102	0	1.616901865
PVX_095475	1	0	0
PVX_095480	0.001084333	0	3.784250345
PVX_095485	0.001084333	0	4.158281692
PVX_095490	0.166224591	0	0.458639451
PVX_095495	1	0	0
PVX_095990	0.359317338	0	0.320196123
PVX_095995	1	0	0
PVX_095997	0.036687443	0	1.133882908
PVX_096000	1	0	0
PVX_096001	1	0	0
PVX_096001a	0.166224591	0	0.543594588
PVX_096003	1	0	0
PVX_096004	1	0	0
PVX_096005	0.166224591	0	0.607344652
PVX_096007	1	0	0
PVX_096010	0.016473156	0	1.831949645
PVX_096015	0.036687443	0	1.730069437
PVX_096020	0.016473156	0	2.000293189
PVX_096030	0.007054102	0	2.695435077
PVX_096035	0.166224591	0	0.589954628
PVX_096040	1	0	0
PVX_096045	0.001084333	0	4.17068054
PVX_096050	0.007054102	0	2.280589801
PVX_096055	0.078805602	0	1.030069073
PVX_096060	0.078805602	0	1.220051484
PVX_096065	0.016473156	0	1.976065525
PVX_096070	0.000383392	0	4.102871594

GeneID	Pvalue	BackGroundPre	BackGroundDx
PVX_096071	1	0	0
PVX_096075	0.036687443	0	1.198701946
PVX_096080	0.007054102	0	2.558572516
PVX_096085	0.166224591	0	0.458423671
PVX_096090	0.166224591	0	0.459595295
PVX_096095	1	0	0
PVX_096105	1	0	0
PVX_096110	0.016473156	0	0.883610385
PVX_096115	0.078805602	0	0.612982859
PVX_096120	1	0	0
PVX_096125	0.078805602	0	1.304273007
PVX_096130	0.016473156	0	1.600575364
PVX_096135	0.036687443	0	1.366615505
PVX_096140	0.016473156	0	1.39097794
PVX_096145	0.036687443	0	0.914859314
PVX_096150	0.001084333	0	2.232769464
PVX_096155	0.007054102	0	1.487439
PVX_096165	0.007054102	0	1.921195587
PVX_096170	0.016473156	0	2.498870224
PVX_096175	0.359317338	0	0.352101243
PVX_096180	0.002855585	0	2.006842786
PVX_096185	1	0	0
PVX_096195	1	0	0
PVX_096200	0.359317338	0	0.243396289
PVX_096205	0.166224591	0	0.558993642
PVX_096210	0.166224591	0	0.752960806
PVX_096215	0.359317338	0	0.265852108
PVX_096220	0.016473156	0	2.366754161
PVX_096225	0.002855585	0	2.686973486
PVX_096230	0.036687443	0	1.550688684
PVX_096235	0.000383392	0	3.488595612
PVX_096240	0.000383392	0	2.862484156
PVX_096245	0.166224591	0	0.612687159
PVX_096250	0.001084333	0	3.014939986
PVX_096252	0.001084333	0	3.448687926
PVX_096253	0.359317338	0	0.543751494
PVX_096255	0.000383392	0	4.106291222
PVX_096260	1	0	0
PVX_096265	0.000125309	0	5.651958782
PVX_096268	0.036687443	0	1.301848754
PVX_096271	0.007054102	0	2.541734522

GeneID	Pvalue	BackGroundPre	BackGroundDx
PVX_096273	0.002855585	0	2.161225631
PVX_096275	0.016473156	0	1.439304362
PVX_096280	0.000125309	0	4.813003299
PVX_096285	0.001084333	0	2.033847002
PVX_096289	0.078805602	0	1.290852293
PVX_096292	0.007054102	0	2.510995948
PVX_096295	0.016473156	0	1.314863861
PVX_096300	0.002855585	0	3.233833791
PVX_096302	0.016473156	0	2.397511277
PVX_096305	0.001084333	0	2.923373313
PVX_096307	1	0	0
PVX_096310	0.359317338	0	0.345735142
PVX_096315	0.036687443	0	1.580088039
PVX_096320	0.002855585	0	2.957669832
PVX_096325	0.002855585	0	1.524533861
PVX_096330	0.007054102	0	2.049260864
PVX_096335	0.000125309	0	5.257979575
PVX_096340	1.03E-05	0	6.93670559
PVX_096345	0.002855585	0	2.360442652
PVX_096350	0.002855585	0	2.172315178
PVX_096355	0.016473156	0	1.664101264
PVX_096360	0.359317338	0	0.325709572
PVX_096365	0.078805602	0	1.348851033
PVX_096370	0.001084333	0	3.655051889
PVX_096380	0.016473156	0	2.252090721
PVX_096385	0.359317338	0	0.282855283
PVX_096390	1	0	0
PVX_096395	0.359317338	0	0.2213104
PVX_096400	0.078805602	0	1.097797271
PVX_096405	0.078805602	0	1.169750737
PVX_096410	1	0	0
PVX_096910	1	0	0
PVX_096920	0.359317338	0	0.268705017
PVX_096925	1	0	0
PVX_096930	0.359317338	0	0.381198591
PVX_096935	1	0	0
PVX_096937	0.359317338	0	0.282336205
PVX_096938	1	0	0
PVX_096940	1	0	0
PVX_096945	0.359317338	0	0.506951212
PVX_096950	0.000383392	0	3.717213786

GeneID	Pvalue	BackGroundPre	BackGroundDx
PVX_096955	0.000125309	0	4.505976065
PVX_096960	1	0	0
PVX_096965	1	0	0
PVX_096970	0.036687443	0	1.441912517
PVX_096975	3.76E-05	0	4.811133136
PVX_096980	0.002855585	0	2.93702268
PVX_096985	0.078805602	0	1.0220985
PVX_096987	0.001084333	0	3.548135498
PVX_096990	0.166224591	0	0.501958672
PVX_096992	0.016473156	0	2.076381343
PVX_096995	0.000125309	0	4.258976099
PVX_097000	0.036687443	0	1.072247877
PVX_097002	1	0	0
PVX_097005	0.166224591	0	0.740121521
PVX_097010	0.078805602	0	1.092774093
PVX_097015	0.016473156	0	1.406031309
PVX_097025	0.007054102	0	1.376654009
PVX_097525	1	0	0
PVX_097530	1	0	0
PVX_097540	0.036687443	0	1.105436216
PVX_097542	1	0	0
PVX_097545	0.166224591	0	0.594601202
PVX_097550	0.078805602	0	0.775570904
PVX_097555	1	0	0
PVX_097557	0.000125309	0	3.771351495
PVX_097560	0.036687443	0	1.296023472
PVX_097565	0.002855585	0	2.760712269
PVX_097567	0.016473156	0	1.97873593
PVX_097570	0.166224591	0	0.525056922
PVX_097575	0.003138348	0.205539965	2.279009527
PVX_097577	0.078805602	0	1.000467515
PVX_097580	0.166224591	0	0.743596083
PVX_097583	0.000125309	0	6.107103813
PVX_097585	0.166224591	0	0.771316254
PVX_097590	1	0	0
PVX_097592	1	0	0
PVX_097595	0.359317338	0	0.322976835
PVX_097600	0.007054102	0	2.46288982
PVX_097605	0.016473156	0	1.4433963
PVX_097610	0.007054102	0	2.238084281
PVX_097615	0.078805602	0	0.854431147

GeneID	Pvalue	BackGroundPre	BackGroundDx
PVX_097620	1	0	0
PVX_097625	1.03E-05	0	5.351226511
PVX_097630	1	0	0
PVX_097635	1	0	0
PVX_097640	0.016473156	0	1.683493265
PVX_097645	0.007054102	0	2.610929771
PVX_097650	1	0	0
PVX_097655	0.166224591	0	0.672399186
PVX_097660	0.001084333	0	2.732081615
PVX_097665	0.036687443	0	1.084936822
PVX_097670	0.359317338	0	0.276953736
PVX_097675	0.078805602	0	0.794567565
PVX_097680	1	0	0
PVX_097685	0.359317338	0	0.237235513
PVX_097690	0.166224591	0	0.398569523
PVX_097695	0.359317338	0	0.126575939
PVX_097700	1	0	0
PVX_097705	0.359317338	0	0.125953995
PVX_097710	0.359317338	0	0.306885202
PVX_097715	0.078805602	0	1.181666531
PVX_097720	0.359317338	0	0.192760116
PVX_097725	0.359317338	0	0.248959046
PVX_097730	0.036687443	0	1.458315348
PVX_097735	0.007054102	0	2.415742191
PVX_097740	1.03E-05	0	5.305454999
PVX_097745	0.078805602	0	1.257471571
PVX_097750	0.359317338	0	0.282629531
PVX_097755	0.359317338	0	0.184002902
PVX_097760	0.000125309	0	5.430130463
PVX_097765	0.007054102	0	2.03989024
PVX_097770	0.000383392	0	2.614657236
PVX_097772	0.359317338	0	0.408601116
PVX_097775	0.016473156	0	1.323624628
PVX_097780	1	0	0
PVX_097785	0.036687443	0	1.128509011
PVX_097787	1	0	0
PVX_097790	0.359317338	0	0.391750625
PVX_097795	0.007054102	0	1.665352104
PVX_097800	0.001084333	0	1.443846021
PVX_097805	0.359317338	0	0.248542098
PVX_097810	0.036687443	0	0.784287479

GeneID	Pvalue	BackGroundPre	BackGroundDx
PVX_097815	0.007054102	0	1.254670879
PVX_097820	1	0	0
PVX_097825	0.036687443	0	1.229210345
PVX_097830	0.000383392	0	3.224008006
PVX_097835	0.002855585	0	2.057734809
PVX_097840	0.078805602	0	0.971533094
PVX_097845	0.036687443	0	1.258439135
PVX_097850	0.036687443	0	1.424021505
PVX_097852	0.078805602	0	1.47895747
PVX_097855	0.078805602	0	1.2593618
PVX_097860	0.000125309	0	4.297330007
PVX_097865	0.002855585	0	3.04965904
PVX_097870	0.166224591	0	0.562976253
PVX_097875	1	0	0
PVX_097885	0.007054102	0	1.202727517
PVX_097890	1	0	0
PVX_097895	0.078805602	0	0.69702318
PVX_097900	0.002855585	0	2.099561817
PVX_097905	0.016473156	0	1.646489642
PVX_097910	0.359317338	0	0.368759372
PVX_097915	3.76E-05	0	5.41768376
PVX_097920	1	0	0
PVX_097925	1	0	0
PVX_097930	0.359317338	0	0.330197821
PVX_097935	1	0	0
PVX_097940	0.078805602	0	0.793135514
PVX_097945	3.76E-05	0	4.537610592
PVX_097950	0.001084333	0	2.43201281
PVX_097955	0.002855585	0	2.931699816
PVX_097960	0.016473156	0	1.967375233
PVX_097965	1	0	0
PVX_097970	0.078805602	0	1.200608208
PVX_097975	0.016473156	0	1.927389108
PVX_097980	1	0	0
PVX_097985	1	0	0
PVX_097990	0.036687443	0	1.668055339
PVX_097995	0.016473156	0	1.394649331
PVX_098000	0.078805602	0	1.014072591
PVX_098005	1	0	0
PVX_098010	0.016473156	0	0.943296931
PVX_098015	0.036687443	0	1.488034099

GeneID	Pvalue	BackGroundPre	BackGroundDx
PVX_098020	0.166224591	0	0.505054334
PVX_098022	0.359317338	0	0.482679711
PVX_098023	0.166224591	0	0.915562103
PVX_098025	0.000125309	0	2.894467718
PVX_098030	1	0	0
PVX_098035	0.036687443	0	1.283031149
PVX_098040	0.001084333	0	3.063098086
PVX_098045	0.359317338	0	0.350152277
PVX_098050	0.002855585	0	2.152488876
PVX_098055	0.007054102	0	2.122549706
PVX_098060	0.359317338	0	0.270127441
PVX_098065	0.007054102	0	1.464094409
PVX_098070	0.002855585	0	2.238960566
PVX_098075	0.166224591	0	0.653776849
PVX_098080	0.036687443	0	1.311095321
PVX_098085	0.078805602	0	0.911334036
PVX_098582	1	0	0
PVX_098585	0.359317338	0	0.095514957
PVX_098590	1	0	0
PVX_098595	0.016473156	0	2.174647516
PVX_098600	0.078805602	0	1.101479127
PVX_098605	0.016473156	0	1.967137941
PVX_098610	0.166224591	0	0.306096285
PVX_098615	0.000125309	0	2.667216285
PVX_098620	0.002855585	0	1.940624046
PVX_098625	1	0	0
PVX_098630	0.007054102	0	1.963990544
PVX_098635	1	0	0
PVX_098640	1.03E-05	0	5.873905522
PVX_098645	0.007054102	0	2.608266194
PVX_098650	0.007054102	0	1.579826548
PVX_098655	0.166224591	0	0.642934897
PVX_098660	0.359317338	0	0.312914873
PVX_098665	0.078805602	0	1.163788061
PVX_098670	0.078805602	0	1.318699736
PVX_098675	0.002855585	0	1.797748815
PVX_098680	0.359317338	0	0.382898619
PVX_098685	0.000383392	0	3.285078517
PVX_098690	0.002855585	0	2.012104224
PVX_098695	0.016473156	0	1.986341705
PVX_098700	0.007054102	0	1.562412431

GeneID	Pvalue	BackGroundPre	BackGroundDx
PVX_098705	0.036687443	0	1.104083861
PVX_098710	1	0	0
PVX_098712	0.359317338	0	0.358074798
PVX_098715	1	0	0
PVX_098720	1	0	0
PVX_098725	0.007054102	0	1.85324284
PVX_098730	0.016473156	0	1.339151812
PVX_098735	0.016473156	0	1.707998665
PVX_098740	0.078805602	0	0.467802109
PVX_098745	0.007054102	0	1.301272803
PVX_098750	0.359317338	0	0.321778688
PVX_098755	0.166224591	0	0.654039484
PVX_098760	0.016473156	0	1.808830753
PVX_098765	1	0	0
PVX_098770	1	0	0
PVX_098775	0.002855585	0	2.407777853
PVX_098780	0.002855585	0	2.435956767
PVX_098784	0.078805602	0	1.587524212
PVX_098786	0.036687443	0	1.563886464
PVX_098795	0.036687443	0	1.769694294
PVX_098800	0.007054102	0	1.58097958
PVX_098805	0.036687443	0	0.947667301
PVX_098810	0.036687443	0	1.552347779
PVX_098815	0.000383392	0	3.178562516
PVX_098820	0.359317338	0	0.340725447
PVX_098825	0.001084333	0	2.843302329
PVX_098830	0.000125309	0	4.129061781
PVX_098835	0.036687443	0	1.68671613
PVX_098840	0.078805602	0	1.078187256
PVX_098845	0.078805602	0	0.938775527
PVX_098847	0.078805602	0	0.805327961
PVX_098850	0.078805602	0	0.593709152
PVX_098855	0.002855585	0	2.545230235
PVX_098860	0.166224591	0	0.677724863
PVX_098865	0.359317338	0	0.116996454
PVX_098870	0.036687443	0	1.390263614
PVX_098875	0.078805602	0	1.128625189
PVX_098880	0.007054102	0	2.050193968
PVX_098885	0.036687443	0	1.020095845
PVX_098890	0.007054102	0	1.919448081
PVX_098895	0.359317338	0	0.204922991

GeneID	Pvalue	BackGroundPre	BackGroundDx
PVX_098900	1	0	0
PVX_098905	0.036687443	0	1.854514011
PVX_098910	0.007054102	0	1.616583122
PVX_098915	0.359317338	0	0.414407657
PVX_098920	0.016473156	0	1.467029876
PVX_098925	0.036687443	0	0.944946822
PVX_098930	0.359317338	0	0.446439167
PVX_098935	0.007054102	0	1.548125991
PVX_098940	0.359317338	0	0.195215282
PVX_098945	0.000125309	0	3.230732161
PVX_098950	0.000125309	0	2.59301774
PVX_098955	0.016473156	0	1.53519315
PVX_098960	0.036687443	0	1.153764681
PVX_098965	0.036687443	0	0.95828175
PVX_098970	0.016473156	0	2.082621987
PVX_098975	0.359317338	0	0.363951918
PVX_098980	0.078805602	0	0.942591417
PVX_098985	0.016473156	0	1.86305537
PVX_098990	1	0	0
PVX_098995	1	0	0
PVX_099000	0.359317338	0	0.281949147
PVX_099005	0.359317338	0	0.087313709
PVX_099010	0.016473156	0	1.350665256
PVX_099015	0.359317338	0	0.308496751
PVX_099020	0.007054102	0	2.098735446
PVX_099025	0.002855585	0	2.388372393
PVX_099030	0.166224591	0	0.546658688
PVX_099035	0.000383392	0	3.941866265
PVX_099040	0.036687443	0	1.185609469
PVX_099045	0.036687443	0	1.198278383
PVX_099050	1	0	0
PVX_099055	0.036687443	0	1.291467814
PVX_099060	0.016473156	0	1.517293421
PVX_099065	0.359317338	0	0.155225333
PVX_099070	0.166224591	0	1.125356153
PVX_099075	0.016473156	0	1.59956324
PVX_099080	0.002855585	0	2.957793416
PVX_099085	0.001084333	0	3.295562622
PVX_099090	1	0	0
PVX_099095	3.76E-05	0	5.291695161
PVX_099100	0.036687443	0	1.206222235

GeneID	Pvalue	BackGroundPre	BackGroundDx
PVX_099105	0.359317338	0	0.360417094
PVX_099107	0.078805602	0	0.901797726
PVX_099110	0.016473156	0	1.312909894
PVX_099115	0.007054102	0	1.824943163
PVX_099117	1	0	0
PVX_099120	0.016473156	0	1.312011235
PVX_099125	0.002855585	0	2.187488341
PVX_099130	0.078805602	0	0.628664156
PVX_099135	0.007054102	0	1.96823417
PVX_099140	0.016473156	0	1.352621211
PVX_099145	0.078805602	0	1.137052824
PVX_099150	0.166224591	0	0.416304212
PVX_099155	0.359317338	0	0.483004053
PVX_099160	0.166224591	0	0.678650474
PVX_099165	1	0	0
PVX_099170	1	0	0
PVX_099175	0.001084333	0	2.496468733
PVX_099180	0.166224591	0	0.624713057
PVX_099185	0.078805602	0	1.422665678
PVX_099190	0.036687443	0	1.488347707
PVX_099195	0.016473156	0	1.682169766
PVX_099200	0.000125309	0	3.547786975
PVX_099205	0.166224591	0	0.478434719
PVX_099210	0.036687443	0	1.809460519
PVX_099215	0.078805602	0	0.952833874
PVX_099220	1	0	0
PVX_099225	0.166224591	0	0.704864794
PVX_099230	0.359317338	0	0.166650582
PVX_099235	0.166224591	0	0.680463237
PVX_099240	0.016473156	0	1.475855912
PVX_099245	1	0	0
PVX_099247	0.002855585	0	1.839599527
PVX_099250	0.016473156	0	1.956071905
PVX_099255	0.007054102	0	1.652274397
PVX_099257	0.359317338	0	0.362379166
PVX_099263	0.359317338	0	0.685653811
PVX_099265	0.359317338	0	0.354465309
PVX_099270	0.036687443	0	0.88245093
PVX_099275	0.002855585	0	2.35352474
PVX_099280	1	0	0
PVX_099285	1	0	0

GeneID	Pvalue	BackGroundPre	BackGroundDx
PVX_099290	1	0	0
PVX_099295	0.001084333	0	2.619700635
PVX_099300	0.007054102	0	2.038592626
PVX_099305	0.166224591	0	0.608451464
PVX_099310	0.016473156	0	2.137121677
PVX_099315	1.03E-05	0	6.24636989
PVX_099320	0.036687443	0	1.398436488
PVX_099325	0.016473156	0	2.359957828
PVX_099330	0.078805602	0	1.164327618
PVX_099335	0.001084333	0	3.854051708
PVX_099340	0.078805602	0	0.682238821
PVX_099345	0.016473156	0	1.547609184
PVX_099350	1	0	0
PVX_099355	0.007054102	0	2.013507949
PVX_099360	0.000125309	0	3.455246952
PVX_099365	1.03E-05	0	6.02233149
PVX_099370	0.002855585	0	2.586817196
PVX_099375	1	0	0
PVX_099380	1	0	0
PVX_099385	0.007054102	0	1.934974287
PVX_099390	0.001084333	0	2.90361071
PVX_099395	0.002855585	0	2.564042156
PVX_099400	0.036687443	0	1.442876933
PVX_099405	0.016473156	0	1.358149298
PVX_099410	0.359317338	0	0.137222028
PVX_099415	0.078805602	0	0.970952978
PVX_099420	0.016473156	0	1.896183161
PVX_099425	0.002855585	0	2.186601239
PVX_099430	1	0	0
PVX_099435	0.001084333	0	2.098945613
PVX_099440	0.359317338	0	0.414382846
PVX_099445	0.007054102	0	1.671631179
PVX_099450	1	0	0
PVX_099455	0.000125309	0	4.22642081
PVX_099460	0.016473156	0	1.916233922
PVX_099465	0.000125309	0	4.651803471
PVX_099470	0.359317338	0	0.169448055
PVX_099475	0.078805602	0	1.196924346
PVX_099480	0.000383392	0	2.511479978
PVX_099485	0.166224591	0	0.683063974
PVX_099490	0.036687443	0	1.516868544

GeneID	Pvalue	BackGroundPre	BackGroundDx
PVX_099495	0.007054102	0	2.119769317
PVX_099500	0.007054102	0	1.322684031
PVX_099505	0.016473156	0	1.613114755
PVX_099507	0.036687443	0	1.300560292
PVX_099510	0.001084333	0	3.036136385
PVX_099515	0.359317338	0	0.179504964
PVX_099520	0.000125309	0	3.503107038
PVX_099525	3.76E-05	0	5.822286303
PVX_099528	1	0	0
PVX_099530	0.036687443	0	1.047670197
PVX_099535	1.03E-05	0	7.268627144
PVX_099540	0.002855585	0	2.46531703
PVX_099545	0.002855585	0	2.599204815
PVX_099550	0.001084333	0	1.708254887
PVX_099555	1	0	0
PVX_099560	0.036687443	0	1.462630299
PVX_099565	0.078805602	0	1.166925692
PVX_099570	0.016473156	0	1.324859173
PVX_099575	0.359317338	0	0.185745149
PVX_099580	0.007054102	0	1.832649861
PVX_099585	1	0	0
PVX_099590	0.007054102	0	2.312205965
PVX_099595	0.359317338	0	0.318085715
PVX_099600	0.000383392	0	3.572337094
PVX_099605	0.007054102	0	2.615804554
PVX_099610	0.359317338	0	0.626220142
PVX_099615	0.078805602	0	0.712600629
PVX_099620	0.036687443	0	1.186793774
PVX_099625	0.036687443	0	0.856401652
PVX_099630	0.016473156	0	1.740413509
PVX_099635	0.002855585	0	2.140161442
PVX_099640	0.359317338	0	0.34687863
PVX_099645	0.078805602	0	0.481975027
PVX_099650	0.001084333	0	2.791596938
PVX_099655	1	0	0
PVX_099660	1	0	0
PVX_099665	1	0	0
PVX_099670	1	0	0
PVX_099675	0.036687443	0	1.299545042
PVX_099680	0.016473156	0	1.671414776
PVX_099685	0.007054102	0	1.629054365

GeneID	Pvalue	BackGroundPre	BackGroundDx
PVX_099690	0.016473156	0	2.367218515
PVX_099695	0.078805602	0	1.150016598
PVX_099700	0.002855585	0	2.490650233
PVX_099705	0.016473156	0	1.161838247
PVX_099710	0.000383392	0	3.947883183
PVX_099715	0.002855585	0	2.015163091
PVX_099725	0.001084333	0	1.980734141
PVX_099735	0.359317338	0	0.406397384
PVX_099740	0.036687443	0	1.030388116
PVX_099745	0.036687443	0	1.220757951
PVX_099750	0.016473156	0	1.36983782
PVX_099755	1	0	0
PVX_099760	0.359317338	0	0.201783966
PVX_099765	3.76E-05	0	3.87750983
PVX_099770	0.016473156	0	1.288431765
PVX_099780	0.078805602	0	1.149305641
PVX_099785	0.036687443	0	1.750171398
PVX_099790	0.001084333	0	3.900325471
PVX_099800	0.166224591	0	0.446139172
PVX_099805	0.036687443	0	1.380649678
PVX_099810	0.359317338	0	0.178659504
PVX_099815	1	0	0
PVX_099820	0.002855585	0	2.370367826
PVX_099825	0.001084333	0	3.410925275
PVX_099830	0.007054102	0	2.180904064
PVX_099835	0.166224591	0	1.124226764
PVX_099840	0.007054102	0	2.258362984
PVX_099845	0.078805602	0	1.202587788
PVX_099850	0.359317338	0	0.301068234
PVX_099855	0.166224591	0	0.572984576
PVX_099860	0.166224591	0	0.694392565
PVX_099870	0.036687443	0	1.525367015
PVX_099875	1	0	0
PVX_099880	1	0	0
PVX_099885	0.007054102	0	1.369750151
PVX_099890	0.016473156	0	1.679479299
PVX_099895	0.078805602	0	1.289162897
PVX_099900	0.166224591	0	0.712668745
PVX_099905	0.036687443	0	1.364585659
PVX_099910	0.166224591	0	0.600076313
PVX_099915	0.000125309	0	4.181068702

GeneID	Pvalue	BackGroundPre	BackGroundDx
PVX_099920	0.166224591	0	0.576407367
PVX_099930	0.007054102	0	1.592035658
PVX_099935	1	0	0
PVX_099940	0.359317338	0	0.415091385
PVX_099944	0.007054102	0	2.301506189
PVX_099946	0.359317338	0	0.434190304
PVX_099955	1	0	0
PVX_099960	0.016473156	0	1.53242451
PVX_099965	0.016473156	0	1.401793368
PVX_099970	1	0	0
PVX_099975	1	0	0
PVX_099980	0.166224591	0	0.417909345
PVX_099985	0.000383392	0	2.907593551
PVX_099990	0.007054102	0	1.424613641
PVX_099995	0.007054102	0	1.917862933
PVX_100000	0.078805602	0	1.005027269
PVX_100005	0.016473156	0	0.847541144
PVX_100010	0.359317338	0	0.303770736
PVX_100510	1	0	0
PVX_100515	0.166224591	0	0.26785542
PVX_100520	0.359317338	0	0.439735676
PVX_100525	0.078805602	0	0.601818858
PVX_100530	0.036687443	0	1.667112466
PVX_100535	0.016473156	0	1.204087102
PVX_100540	0.001084333	0	2.437788625
PVX_100545	0.016473156	0	1.779145265
PVX_100550	0.000383392	0	3.481287385
PVX_100555	0.001084333	0	2.892246756
PVX_100560	0.000383392	0	3.618715512
PVX_100565	0.359317338	0	0.223128001
PVX_100570	0.359317338	0	0.17700061
PVX_100575	0.359317338	0	0.411415131
PVX_100580	0.036687443	0	1.121697458
PVX_100590	0.359317338	0	0.503399388
PVX_100595	0.007054102	0	2.00582634
PVX_100600	0.001084333	0	2.790150825
PVX_100610	0.001084333	0	3.43701995
PVX_100620	0.359317338	0	0.171908904
PVX_100625	0.016473156	0	1.26050331
PVX_100630	0.007054102	0	2.210306784
PVX_100635	3.76E-05	0	2.838653577

GeneID	Pvalue	BackGroundPre	BackGroundDx
PVX_100640	0.036687443	0	1.47494592
PVX_100645	0.001084333	0	3.428741579
PVX_100650	0.007054102	0	1.559382662
PVX_100655	0.078805602	0	1.02526591
PVX_100657	0.002855585	0	1.570904032
PVX_100660	0.036687443	0	0.890944825
PVX_100665	0.000383392	0	2.30697162
PVX_100670	1	0	0
PVX_100675	0.002855585	0	2.737025793
PVX_100680	0.007054102	0	1.810244165
PVX_100685	0.166224591	0	0.584237302
PVX_100690	3.76E-05	0	3.668516976
PVX_100695	0.001084333	0	3.649265335
PVX_100700	1	0	0
PVX_100705	0.001084333	0	3.977485042
PVX_100710	0.166224591	0	0.526314516
PVX_100715	0.000125309	0	3.151651601
PVX_100720	0.007054102	0	1.966486995
PVX_100725	0.016473156	0	1.443036421
PVX_100730	0.000125309	0	4.022175316
PVX_100735	0.001084333	0	3.082528975
PVX_100740	0.036687443	0	0.815479251
PVX_100745	0.001084333	0	2.664560156
PVX_100750	0.036687443	0	1.541107415
PVX_100755	0.016473156	0	1.793847371
PVX_100760	0.359317338	0	0.262026697
PVX_100765	0.016473156	0	1.767248269
PVX_100770	0.007054102	0	2.195861402
PVX_100775	1	0	0
PVX_100780	1	0	0
PVX_100785	0.016473156	0	1.499732589
PVX_100790	0.078805602	0	0.975770698
PVX_100795	0.036687443	0	1.554430487
PVX_100800	0.007054102	0	1.868714868
PVX_100805	0.000125309	0	2.668956485
PVX_100810	0.002855585	0	2.156010105
PVX_100815	0.166224591	0	0.585436813
PVX_100820	0.002855585	0	2.60659861
PVX_100825	0.007054102	0	1.536472362
PVX_100830	0.001084333	0	2.891387912
PVX_100835	0.000383392	0	3.832958823

GeneID	Pvalue	BackGroundPre	BackGroundDx
PVX_100840	0.078805602	0	1.122565194
PVX_100845	0.007054102	0	2.048930128
PVX_100850	1	0	0
PVX_100855	0.000383392	0	3.558685524
PVX_100860	1	0	0
PVX_100865	0.002855585	0	1.87232319
PVX_100870	0.359317338	0	0.271603823
PVX_100875	0.036687443	0	0.871043465
PVX_100880	0.078805602	0	0.897723864
PVX_100885	0.001084333	0	2.822695079
PVX_100890	0.000383392	0	3.399296532
PVX_100895	1	0	0
PVX_100900	0.036687443	0	1.050713659
PVX_100905	0.002855585	0	2.838497497
PVX_100910	0.000383392	0	2.565039486
PVX_100915	0.166224591	0	0.58983348
PVX_100920	1	0	0
PVX_100925	0.002855585	0	2.224326911
PVX_100930	0.007054102	0	2.038380428
PVX_100935	0.001084333	0	2.764978811
PVX_100940	0.078805602	0	0.594528306
PVX_100945	0.359317338	0	0.219267096
PVX_100950	0.078805602	0	0.865691176
PVX_100955	0.007054102	0	2.343708686
PVX_100960	0.016473156	0	1.034436838
PVX_100965	0.002855585	0	2.407274422
PVX_100970	1	0	0
PVX_100975	0.016473156	0	1.64434147
PVX_100980	0.000383392	0	3.337793098
PVX_100985	0.002855585	0	2.757035173
PVX_100990	0.002855585	0	1.998940148
PVX_100995	0.016473156	0	1.754411913
PVX_101000	0.359317338	0	0.215091269
PVX_101005	0.359317338	0	0.72160676
PVX_101010	0.000125309	0	3.81385263
PVX_101015	0.016473156	0	1.976192513
PVX_101020	0.016473156	0	1.846483122
PVX_101025	0.007054102	0	2.336376713
PVX_101030	0.078805602	0	0.889165427
PVX_101035	1.03E-05	0	6.49517517
PVX_101040	0.001084333	0	3.58849748

GeneID	Pvalue	BackGroundPre	BackGroundDx
PVX_101045	0.016473156	0	2.285731842
PVX_101050	0.016473156	0	1.861685949
PVX_101055	0.036687443	0	1.264588441
PVX_101060	0.002855585	0	2.73943221
PVX_101065	1	0	0
PVX_101070	0.359317338	0	0.236075128
PVX_101075	0.359317338	0	0.359411258
PVX_101080	0.002855585	0	2.85382153
PVX_101085	1	0	0
PVX_101090	0.002855585	0	2.825511028
PVX_101095	0.359317338	0	0.213857244
PVX_101100	0.016473156	0	1.529361839
PVX_101105	0.000383392	0	2.869454537
PVX_101110	0.000383392	0	3.230997172
PVX_101115	0.016473156	0	1.566899691
PVX_101120	0.166224591	0	0.787999277
PVX_101125	0.359317338	0	0.41306419
PVX_101130	0.036687443	0	1.806851414
PVX_101135	0.000125309	0	4.042443826
PVX_101140	0.166224591	0	0.440887583
PVX_101145	0.016473156	0	1.773154319
PVX_101150	0.166224591	0	0.515892481
PVX_101155	0.036687443	0	1.182944396
PVX_101160	0.078805602	0	1.314359799
PVX_101165	0.016473156	0	1.879014598
PVX_101170	0.002855585	0	2.752200367
PVX_101175	0.166224591	0	0.314044926
PVX_101180	0.078805602	0	1.102337896
PVX_101185	0.078805602	0	1.031502606
PVX_101190	0.007054102	0	2.179179373
PVX_101195	0.166224591	0	0.610450168
PVX_101200	0.001084333	0	3.369617563
PVX_101205	0.359317338	0	0.181824152
PVX_101210	0.359317338	0	0.204682492
PVX_101215	0.166224591	0	0.787191811
PVX_101220	0.166224591	0	0.753372343
PVX_101225	0.016473156	0	1.716207911
PVX_101230	0.078805602	0	1.032658974
PVX_101235	0.036687443	0	1.437573086
PVX_101240	0.078805602	0	0.730771764
PVX_101245	0.166224591	0	0.665823786

GeneID	Pvalue	BackGroundPre	BackGroundDx
PVX_101250	0.036687443	0	1.218276961
PVX_101255	0.036687443	0	1.012387125
PVX_101257	0.078805602	0	0.884105961
PVX_101260	3.76E-05	0	4.798648133
PVX_101265	0.007054102	0	1.897439294
PVX_101269	0.016473156	0	1.474576489
PVX_101271	1	0	0
PVX_101275	1	0	0
PVX_101280	1	0	0
PVX_101285	0.036687443	0	1.515820097
PVX_101290	0.166224591	0	0.92418111
PVX_101295	0.016473156	0	1.886191459
PVX_101300	0.078805602	0	0.944363249
PVX_101305	0.078805602	0	1.148485239
PVX_101310	1	0	0
PVX_101315	0.000125309	0	4.747999712
PVX_101320	0.001084333	0	2.710045538
PVX_101325	0.078805602	0	0.54405432
PVX_101330	1	0	0
PVX_101335	0.000125309	0	4.471806501
PVX_101340	0.036687443	0	1.242093628
PVX_101345	0.007054102	0	2.374733346
PVX_101350	1	0	0
PVX_101355	0.007054102	0	1.800886608
PVX_101360	0.078805602	0	1.453849462
PVX_101362	0.036687443	0	1.66360947
PVX_101365	0.359317338	0	0.194844474
PVX_101370	1	0	0
PVX_101375	1	0	0
PVX_101380	0.166224591	0	0.971387955
PVX_101385	0.001084333	0	1.958607895
PVX_101390	0.016473156	0	1.508031306
PVX_101395	0.000125309	0	4.314483335
PVX_101400	0.166224591	0	0.395866758
PVX_101405	0.007054102	0	1.767160739
PVX_101410	0.078805602	0	1.152679463
PVX_101415	0.359317338	0	0.238641409
PVX_101420	0.002855585	0	2.915513659
PVX_101425	0.007054102	0	2.254953976
PVX_101430	0.078805602	0	1.095209481
PVX_101435	0.007054102	0	1.666024646

GeneID	Pvalue	BackGroundPre	BackGroundDx
PVX_101440	0.078805602	0	0.764932807
PVX_101442	1	0	0
PVX_101445	0.036687443	0	1.207951378
PVX_101450	1	0	0
PVX_101455	0.007054102	0	2.474009993
PVX_101460	0.359317338	0	0.231965873
PVX_101465	0.036687443	0	1.22954665
PVX_101470	0.007054102	0	1.732037032
PVX_101475	0.007054102	0	1.858286222
PVX_101480	0.016473156	0	1.472508689
PVX_101482	1	0	0
PVX_101484	1	0	0
PVX_101485	0.166224591	0	0.285399395
PVX_101490	0.007054102	0	3.138010061
PVX_101495	0.166224591	0	0.33562056
PVX_101500	0.000383392	0	2.441001115
PVX_101503	0.359317338	0	0.425615067
PVX_101505	1	0	0
PVX_101510	0.007054102	0	1.911220952
PVX_101515	0.000383392	0	4.090813594
PVX_101520	3.76E-05	0	5.447953689
PVX_101525	0.078805602	0	1.153426308
PVX_101530	0.002855585	0	2.503697404
PVX_101535	0.166224591	0	0.625972859
PVX_101540	0.166224591	0	0.744139278
PVX_101545	0.359317338	0	0.314821522
PVX_101550	0.359317338	0	0.4713679
PVX_101555	0.007054102	0	1.747074966
PVX_101560	0.078805602	0	1.002335399
PVX_101562	1	0	0
PVX_101565	1	0	0
PVX_101570	1	0	0
PVX_101575	0.036687443	0	1.167284803
PVX_101580	0.002855585	0	2.86006649
PVX_101585	1	0	0
PVX_101590	1	0	0
PVX_101592	0.036687443	0	1.817859602
PVX_101595	0.016473156	0	2.555302023
PVX_101600	0.078805602	0	0.844011651
PVX_101605	0.359317338	0	0.45802936
PVX_101610	0.000125309	0	4.34472305

GeneID	Pvalue	BackGroundPre	BackGroundDx
PVX_101615	1	0	0
PVX_101617	0.359317338	0	0.281707596
PVX_101620	0.078805602	0	0.781040506
PVX_101625	1	0	0
PVX_101630	1	0	0
PVX_102125	1	0	0
PVX_102130	1	0	0
PVX_102630	1	0	0
PVX_102635	0.359317338	0	0.422948337
PVX_102640	1	0	0
PVX_102645	1	0	0
PVX_103145	1	0	0
PVX_103150	0.078805602	0	0.815267553
PVX_103155	0.166224591	0	0.600887799
PVX_103160	1	0	0
PVX_103660	0.007054102	0	2.329968667
PVX_103665	1	0	0
PVX_103670	1	0	0
PVX_103675	1	0	0
PVX_103680	1	0	0
PVX_104180	0.359317338	0	0.270259312
PVX_104185	1	0	0
PVX_104190	1	0	0
PVX_104690	1	0	0
PVX_104695	1	0	0
PVX_104700	0.359317338	0	0.228644384
PVX_105200	0.359317338	0	0.26060613
PVX_105205	1	0	0
PVX_105700	1	0	0
PVX_105705	1	0	0
PVX_105710	1	0	0
PVX_106210	1	0	0
PVX_106215	1	0	0
PVX_106220	1	0	0
PVX_106720	1	0	0
PVX_106725	1	0	0
PVX_106730	1	0	0
PVX_107230	1	0	0
PVX_107235	1	0	0
PVX_107735	0.078805602	0	1.023602774
PVX_107740	0.078805602	0	1.072094719

GeneID	Pvalue	BackGroundPre	BackGroundDx
PVX_107745	0.036687443	0	1.219528023
PVX_107750	1	0	0
PVX_107755	1	0	0
PVX_108255	1	0	0
PVX_108260	1	0	0
PVX_108760	1	0	0
PVX_108765	1	0	0
PVX_108770	0.078805602	0	0.838188458
PVX_108775	1	0	0
PVX_109275	0.359317338	0	0.319607761
PVX_109280	0.016473156	0	1.971310179
PVX_109778	1	0	0
PVX_109780	1	0	0
PVX_109785	1	0	0
PVX_109790	0.359317338	0	0.329736631
PVX_109795	1	0	0
PVX_110295	1	0	0
PVX_110300	1	0	0
PVX_110805	1	0	0
PVX_110810	1	0	0
PVX_110815	1	0	0
PVX_110820	1	0	0
PVX_110822	0.359317338	0	0.338653963
PVX_110825	0.002855585	0	2.166266571
PVX_110830	0.036687443	0	1.562687809
PVX_110832	0.016473156	0	2.046539209
PVX_110834	0.359317338	0	0.326548289
PVX_110835	3.76E-05	0	5.264115342
PVX_110840	0.359317338	0	0.445714823
PVX_110845	0.078805602	0	1.370628642
PVX_110845a	1	0	0
PVX_110850	0.036687443	0	1.233116415
PVX_110855	0.000383392	0	3.579587704
PVX_110860	0.359317338	0	0.359922904
PVX_110865	0.359317338	0	0.322605466
PVX_110870	0.016473156	0	2.256606912
PVX_110880	0.078805602	0	0.914934816
PVX_110885	0.359317338	0	0.189261739
PVX_110890	0.359317338	0	0.383448334
PVX_110895	0.036687443	0	1.422679113
PVX_110900	0.359317338	0	0.193901263

GeneID	Pvalue	BackGroundPre	BackGroundDx
PVX_110905	0.002855585	0	2.192381602
PVX_110910	0.001084333	0	1.870438355
PVX_110915	0.002855585	0	1.857279093
PVX_110920	0.359317338	0	0.300676165
PVX_110925	0.001084333	0	3.352506488
PVX_110930	0.036687443	0	1.594136137
PVX_110935	1	0	0
PVX_110940	0.000125309	0	3.946323853
PVX_110945	1	0	0
PVX_110950	1	0	0
PVX_110955	1	0	0
PVX_110960	0.078805602	0	0.767959148
PVX_110965	1	0	0
PVX_110970	1	0	0
PVX_110975	0.007054102	0	1.495544029
PVX_110980	0.000125309	0	3.929361387
PVX_110985	0.078805602	0	1.079912859
PVX_110990	1	0	0
PVX_110995	0.036687443	0	1.420256716
PVX_111000	0.007054102	0	1.65970353
PVX_111005	0.007054102	0	1.771920205
PVX_111010	0.359317338	0	0.259171705
PVX_111015	0.359317338	0	0.480964849
PVX_111020	0.359317338	0	0.362310633
PVX_111025	0.016473156	0	1.688169741
PVX_111030	0.016473156	0	2.400337186
PVX_111035	1	0	0
PVX_111040	0.002855585	0	2.740738894
PVX_111045	0.000125309	0	3.353834762
PVX_111050	0.001084333	0	2.905641165
PVX_111055	0.000125309	0	4.686753461
PVX_111060	0.166224591	0	0.742071675
PVX_111065	0.359317338	0	0.222237182
PVX_111070	3.76E-05	0	4.589233161
PVX_111075	0.016473156	0	1.518955634
PVX_111080	0.002855585	0	2.200504652
PVX_111085	0.007054102	0	2.110021768
PVX_111090	0.016473156	0	1.570275989
PVX_111095	0.007054102	0	2.248142649
PVX_111100	0.359317338	0	0.390414918
PVX_111105	0.359317338	0	0.23216602

GeneID	Pvalue	BackGroundPre	BackGroundDx
PVX_111110	0.002855585	0	2.246204908
PVX_111115	0.166224591	0	0.596325323
PVX_111120	0.007054102	0	1.484090609
PVX_111125	0.016473156	0	1.25569769
PVX_111130	1	0	0
PVX_111135	0.166224591	0	0.572740932
PVX_111140	1	0	0
PVX_111145	0.359317338	0	0.315354818
PVX_111150	0.036687443	0	1.150435691
PVX_111155	0.036687443	0	1.030202752
PVX_111160	1	0	0
PVX_111165	1	0	0
PVX_111170	1	0	0
PVX_111175	0.166224591	0	0.686305934
PVX_111180	1	0	0
PVX_111185	0.016473156	0	1.814344875
PVX_111190	0.016473156	0	2.136034148
PVX_111195	0.000383392	0	3.182755152
PVX_111200	0.002855585	0	2.998515611
PVX_111205	1	0	0
PVX_111210	0.166224591	0	0.442739942
PVX_111215	0.166224591	0	0.510308953
PVX_111220	0.001084333	0	2.051499969
PVX_111225	0.078805602	0	1.090710406
PVX_111230	0.166224591	0	0.44697583
PVX_111235	0.002855585	0	2.403864039
PVX_111240	1	0	0
PVX_111245	0.000383392	0	4.830352036
PVX_111250	0.078805602	0	1.05264552
PVX_111255	0.000125309	0	3.540185393
PVX_111260	0.166224591	0	0.501422158
PVX_111265	0.036687443	0	1.386363798
PVX_111270	0.016473156	0	1.247097579
PVX_111275	0.359317338	0	0.210316565
PVX_111280	1	0	0
PVX_111285	0.166224591	0	0.801083775
PVX_111290	0.359317338	0	0.247714259
PVX_111292	1	0	0
PVX_111295	0.007054102	0	1.959413078
PVX_111300	0.001084333	0	3.094834499
PVX_111305	0.078805602	0	1.134377986

GeneID	Pvalue	BackGroundPre	BackGroundDx
PVX_111310	1	0	0
PVX_111315	0.007054102	0	2.155585287
PVX_111320	0.002855585	0	2.154441802
PVX_111325	1	0	0
PVX_111330	1.03E-05	0	6.421665556
PVX_111335	0.359317338	0	0.389265218
PVX_111340	0.036687443	0	1.400365431
PVX_111345	0.078805602	0	1.537838556
PVX_111350	0.016473156	0	1.921160314
PVX_111355	0.001084333	0	2.919754221
PVX_111360	1	0	0
PVX_111365	0.002855585	0	2.271388175
PVX_111370	0.359317338	0	0.160265199
PVX_111375	1	0	0
PVX_111380	3.76E-05	0	6.191215073
PVX_111385	1	0	0
PVX_111387	1	0	0
PVX_111390	1	0	0
PVX_111395	0.166224591	0	0.396894244
PVX_111400	0.078805602	0	0.857255057
PVX_111405	1	0	0
PVX_111410	0.007054102	0	2.189235864
PVX_111415	0.359317338	0	0.144879632
PVX_111420	1	0	0
PVX_111425	0.016473156	0	2.065445033
PVX_111430	0.359317338	0	0.535078937
PVX_111435	0.001084333	0	1.364576073
PVX_111440	0.166224591	0	0.5117699
PVX_111445	0.002855585	0	2.450402372
PVX_111450	0.166224591	0	0.667085408
PVX_111455	0.001084333	0	2.628035166
PVX_111460	0.359317338	0	0.223591227
PVX_111465	0.007054102	0	1.686854218
PVX_111470	1.03E-05	0	5.039532079
PVX_111475	0.036687443	0	1.284015053
PVX_111480	0.016473156	0	1.651265187
PVX_111485	0.007054102	0	2.679008513
PVX_111490	0.016473156	0	1.714960706
PVX_111495	0.002855585	0	2.98297278
PVX_111500	0.359317338	0	0.433842863
PVX_111510	0.359317338	0	0.181544433

GeneID	Pvalue	BackGroundPre	BackGroundDx
PVX_111515	0.007054102	0	1.938955073
PVX_111520	1.03E-05	0	3.946651643
PVX_111525	1	0	0
PVX_111530	1	0	0
PVX_111535	1	0	0
PVX_111540	0.001084333	0	4.547456804
PVX_111545	0.016473156	0	2.070916955
PVX_111550	0.078805602	0	1.65069333
PVX_111555	0.002855585	0	2.536601768
PVX_111560	0.007054102	0	0.941261109
PVX_111565	0.166224591	0	0.722192026
PVX_111570	0.016473156	0	1.82547888
PVX_111575	0.002855585	0	2.313889589
PVX_111580	0.359317338	0	0.282195357
PVX_111590	0.002855585	0	2.139157424
PVX_111595	0.016473156	0	1.036674445
PVX_111600	0.002855585	0	2.573717814
PVX_112100	1	0	0
PVX_112105	0.007054102	0	2.142430291
PVX_112110	0.000383392	0	3.644598131
PVX_112115	1	0	0
PVX_112120	1	0	0
PVX_112125	1	0	0
PVX_112625	0.359317338	0	0.364102298
PVX_112630	0.078805602	0	1.092573856
PVX_112635	0.359317338	0	0.360534238
PVX_112640	1	0	0
PVX_112645	1	0	0
PVX_112650	0.078805602	0	1.334853062
PVX_112655	0.016473156	0	1.615075354
PVX_112660	0.036687443	0	1.412862881
PVX_112665	0.007054102	0	2.533652402
PVX_112670	0.000232007	0.35781602	4.68884563
PVX_112675	0.007054102	0	1.866888734
PVX_112680	3.76E-05	0	4.670046774
PVX_112685	0.007054102	0	2.077400284
PVX_112690	1.03E-05	0	5.672277268
PVX_112695	0.359317338	0	0.351300007
PVX_112700	0.036687443	0	1.344352214
PVX_112705	0.036687443	0	1.413919363
PVX_112710	0.166224591	0	0.615309956

GeneID	Pvalue	BackGroundPre	BackGroundDx
PVX_112715	1	0	0
PVX_112720	1	0	0
PVX_113220	0.359317338	0	0.297572486
PVX_113225	0.078805602	0	1.259800713
PVX_113230	0.078805602	0	0.998598264
PVX_113235	3.76E-05	0	5.361361217
PVX_113240	0.007054102	0	2.261662833
PVX_113245	0.000125309	0	4.81277779
PVX_113250	0.016473156	0	2.042296954
PVX_113255	0.078805602	0	1.248841104
PVX_113260	0.166224591	0	0.452659637
PVX_113265	0.036687443	0	0.940671184
PVX_113270	0.007054102	0	1.558658874
PVX_113275	0.036687443	0	1.575916866
PVX_113280	1	0	0
PVX_113285	0.036687443	0	1.17810604
PVX_113290	0.078805602	0	1.075425391
PVX_113295	0.016473156	0	1.333605107
PVX_113300	0.078805602	0	0.984944822
PVX_113305	0.078805602	0	0.929920409
PVX_113310	0.001084333	0	2.78366713
PVX_113315	0.166224591	0	0.6183428
PVX_113320	0.001084333	0	3.116104631
PVX_113325	0.016473156	0	1.526373234
PVX_113330	0.002855585	0	2.595232579
PVX_113335	0.001084333	0	2.863858196
PVX_113340	0.002855585	0	2.53372668
PVX_113345	0.001084333	0	1.850565124
PVX_113350	0.036687443	0	1.265326132
PVX_113355	0.036687443	0	0.86271362
PVX_113360	1	0	0
PVX_113365	0.007054102	0	1.499443696
PVX_113370	0.359317338	0	0.166142105
PVX_113375	0.166224591	0	0.683124261
PVX_113380	0.001084333	0	2.908747649
PVX_113385	0.078805602	0	0.968744165
PVX_113390	3.76E-05	0	3.229642199
PVX_113395	0.166224591	0	0.497190986
PVX_113400	0.166224591	0	0.70648518
PVX_113405	0.016473156	0	1.326554581
PVX_113410	0.359317338	0	0.401208229

GeneID	Pvalue	BackGroundPre	BackGroundDx
PVX_113415	0.078805602	0	1.174633996
PVX_113420	0.000383392	0	3.346916317
PVX_113430	0.359317338	0	0.4258407
PVX_113435	0.166224591	0	0.585347149
PVX_113440	0.078805602	0	1.427161218
PVX_113445	0.036687443	0	1.355677918
PVX_113450	0.166224591	0	0.337002592
PVX_113455	1	0	0
PVX_113460	0.036687443	0	0.961089213
PVX_113465	0.001084333	0	3.434414527
PVX_113470	0.016473156	0	1.412526399
PVX_113475	0.002855585	0	2.467601756
PVX_113480	0.359317338	0	0.515924653
PVX_113485	0.359317338	0	0.498946552
PVX_113490	0.359317338	0	0.386106778
PVX_113495	0.000125309	0	3.994536714
PVX_113500	0.007054102	0	1.59761446
PVX_113505	0.001084333	0	2.714511886
PVX_113510	0.036687443	0	1.698119386
PVX_113515	3.76E-05	0	3.950011001
PVX_113520	1	0	0
PVX_113525	0.078805602	0	0.953850649
PVX_113530	0.078805602	0	1.039695019
PVX_113535	0.000383392	0	3.240904855
PVX_113540	0.007054102	0	2.034483341
PVX_113545	1	0	0
PVX_113550	1	0	0
PVX_113555	0.007054102	0	2.051616535
PVX_113560	0.002855585	0	2.262407497
PVX_113565	0.166224591	0	0.677208312
PVX_113567	0.016473156	0	1.479690652
PVX_113570	0.007054102	0	2.579538695
PVX_113574	0.078805602	0	0.636532692
PVX_113576	0.007054102	0	2.088230218
PVX_113580	0.078805602	0	1.416165318
PVX_113585	0.000383392	0	4.537480997
PVX_113590	1	0	0
PVX_113595	3.76E-05	0	5.086034423
PVX_113600	0.007054102	0	1.984145266
PVX_113605	0.007054102	0	1.992599383
PVX_113610	0.000125309	0	2.280511624

GeneID	Pvalue	BackGroundPre	BackGroundDx
PVX_113615	0.078805602	0	1.041575784
PVX_113617	0.007054102	0	1.938876946
PVX_113620	0.036687443	0	1.139409327
PVX_113625	0.359317338	0	0.242127834
PVX_113630	1	0	0
PVX_113635	0.007054102	0	1.389110224
PVX_113640	0.359317338	0	0.271183375
PVX_113645	0.007054102	0	1.478365212
PVX_113650	0.016473156	0	1.893319444
PVX_113655	0.001084333	0	3.170369923
PVX_113660	0.001084333	0	3.033253192
PVX_113665	0.001084333	0	3.764611332
PVX_113670	0.016473156	0	1.852267548
PVX_113675	0.000125309	0	3.889621317
PVX_113680	0.002855585	0	2.248173084
PVX_113685	0.166224591	0	0.592841589
PVX_113690	1	0	0
PVX_113695	0.007054102	0	2.030891218
PVX_113705	0.036687443	0	1.568681214
PVX_113710	0.166224591	0	0.479594678
PVX_113720	1	0	0
PVX_113725	0.359317338	0	0.335389407
PVX_113725a	0.002855585	0	5.146977119
PVX_113731	0.000383392	0	2.059256474
PVX_113735	0.359317338	0	0.37472429
PVX_113740	0.000383392	0	3.685593958
PVX_113745	0.359317338	0	0.408219211
PVX_113750	3.76E-05	0	4.60196289
PVX_113755	0.359317338	0	0.164543203
PVX_113757	0.359317338	0	0.441690037
PVX_113760	0.036687443	0	1.654975886
PVX_113765	1	0	0
PVX_113770	0.166224591	0	0.733686366
PVX_113775	0.359317338	0	0.385443808
PVX_113780	0.036687443	0	1.3491401
PVX_113785	0.000125309	0	4.017820281
PVX_113790	3.76E-05	0	4.59083342
PVX_113795	0.359317338	0	0.425955798
PVX_113797	1	0	0
PVX_113800	0.078805602	0	0.569252099
PVX_113805	0.359317338	0	0.317245204

GeneID	Pvalue	BackGroundPre	BackGroundDx
PVX_113810	0.007054102	0	1.918744202
PVX_113815	1	0	0
PVX_113820	0.001084333	0	2.892275546
PVX_113825	3.76E-05	0	2.707112077
PVX_113830	0.036687443	0	0.698145925
PVX_113835	0.078805602	0	0.892382237
PVX_113844	0.359317338	0	0.223114253
PVX_113846	0.007054102	0	1.672033272
PVX_113850	0.002855585	0	2.171315171
PVX_113855	0.166224591	0	0.834466139
PVX_113860	3.76E-05	0	6.197077321
PVX_113865	0.078805602	0	0.804949137
PVX_113870	1	0	0
PVX_113875	0.036687443	0	1.515289277
PVX_113880	0.007054102	0	1.633950545
PVX_113885	0.359317338	0	0.391593742
PVX_113890	1	0	0
PVX_113895	0.359317338	0	0.247714259
PVX_113900	0.002855585	0	2.396542224
PVX_113905	0.001084333	0	1.923923499
PVX_113910	1	0	0
PVX_113915	0.000125309	0	2.710408759
PVX_113920	0.016473156	0	2.233081234
PVX_113925	0.002855585	0	1.956624831
PVX_113930	0.016473156	0	1.039281365
PVX_113935	0.359317338	0	0.305374759
PVX_113940	0.166224591	0	0.762275047
PVX_113945	0.002855585	0	2.155209228
PVX_113950	0.016473156	0	2.005175374
PVX_113955	0.016473156	0	1.34101494
PVX_113960	1	0	0
PVX_113965	1	0	0
PVX_113970	0.016473156	0	1.618327324
PVX_113975	0.359317338	0	0.23317484
PVX_113980	0.016473156	0	1.762095636
PVX_113985	0.359317338	0	0.19527403
PVX_113990	0.001084333	0	3.166398913
PVX_113995	0.036687443	0	1.086990125
PVX_114000	0.002855585	0	2.936013178
PVX_114005	0.359317338	0	0.494895546
PVX_114010	0.078805602	0	1.386148745

GeneID	Pvalue	BackGroundPre	BackGroundDx
PVX_114015	0.036687443	0	2.053993337
PVX_114020	0.166224591	0	0.687427389
PVX_114025	0.036687443	0	1.274137243
PVX_114030	0.036687443	0	1.112598886
PVX_114035	3.76E-05	0	5.367462965
PVX_114040	1.03E-05	0	6.911397382
PVX_114045	0.078805602	0	0.864167217
PVX_114050	0.001084333	0	3.324838559
PVX_114055	0.000125309	0	4.150888416
PVX_114060	0.007054102	0	2.694061167
PVX_114065	0.007054102	0	1.724395723
PVX_114070	0.078805602	0	1.025147854
PVX_114075	0.001084333	0	2.285372149
PVX_114080	0.359317338	0	0.400360356
PVX_114085	0.016473156	0	1.452927183
PVX_114090	0.036687443	0	0.623620372
PVX_114095	3.76E-05	0	4.957207443
PVX_114100	0.000125309	0	3.364465155
PVX_114105	0.036687443	0	1.172236235
PVX_114110	0.078805602	0	0.709755291
PVX_114115	0.036687443	0	0.901098299
PVX_114120	0.166224591	0	0.77837626
PVX_114125	0.016473156	0	1.849634134
PVX_114130	0.036687443	0	1.543360847
PVX_114135	1	0	0
PVX_114140	0.166224591	0	0.557799066
PVX_114145	1	0	0
PVX_114150	0.016473156	0	1.603114891
PVX_114155	0.166224591	0	0.717574613
PVX_114160	0.007054102	0	2.152323469
PVX_114163	1	0	0
PVX_114165	1	0	0
PVX_114167	1	0	0
PVX_114170	0.016473156	0	1.598009268
PVX_114175	0.036687443	0	0.978778058
PVX_114180	0.002855585	0	3.216270932
PVX_114185	0.001084333	0	2.950430412
PVX_114190	1	0	0
PVX_114195	0.016473156	0	1.816342261
PVX_114197	1	0	0
PVX_114200	0.016473156	0	1.361348332

GeneID	Pvalue	BackGroundPre	BackGroundDx
PVX_114205	0.000125309	0	4.600756918
PVX_114210	3.76E-05	0	4.294060502
PVX_114215	1	0	0
PVX_114220	0.359317338	0	0.180557958
PVX_114225	0.016473156	0	1.547966319
PVX_114230	0.016473156	0	1.4925086
PVX_114235	0.016473156	0	2.243674665
PVX_114240	0.166224591	0	0.664605455
PVX_114245	0.166224591	0	0.631597327
PVX_114250	0.036687443	0	1.319387327
PVX_114255	0.002855585	0	2.263382155
PVX_114260	0.036687443	0	0.655866595
PVX_114265	0.166224591	0	0.56398474
PVX_114270	0.000383392	0	3.137221231
PVX_114275	0.036687443	0	1.304662955
PVX_114280	0.007054102	0	1.958107093
PVX_114285	1	0	0
PVX_114290	0.007054102	0	2.448075244
PVX_114295	0.000383392	0	3.178438811
PVX_114300	0.016473156	0	1.438292258
PVX_114305	0.007054102	0	1.607524056
PVX_114310	0.078805602	0	1.110232732
PVX_114315	0.001084333	0	3.432701245
PVX_114320	0.359317338	0	0.341218016
PVX_114325	0.007054102	0	2.185907955
PVX_114330	0.359317338	0	0.330945349
PVX_114335	0.016473156	0	1.563834137
PVX_114337	0.359317338	0	0.503033505
PVX_114340	0.016473156	0	1.062383013
PVX_114344	1	0	0
PVX_114346	0.359317338	0	0.18017005
PVX_114350	0.359317338	0	0.235234861
PVX_114355	0.016473156	0	1.647276364
PVX_114360	1	0	0
PVX_114365	0.359317338	0	0.288524635
PVX_114370	0.016473156	0	1.419220414
PVX_114375	0.359317338	0	0.34225994
PVX_114380	0.078805602	0	0.932030498
PVX_114385	0.166224591	0	0.713120171
PVX_114390	0.078805602	0	1.358441349
PVX_114395	0.036687443	0	1.089497717

GeneID	Pvalue	BackGroundPre	BackGroundDx
PVX_114400	1	0	0
PVX_114405	0.000125309	0	3.251158608
PVX_114410	0.036687443	0	1.094951471
PVX_114415	0.002855585	0	2.699042638
PVX_114420	0.166224591	0	0.557708608
PVX_114425	1	0	0
PVX_114430	0.166224591	0	0.396894244
PVX_114435	0.007054102	0	2.633464829
PVX_114440	0.016473156	0	1.8122778
PVX_114445	3.76E-05	0	5.853808965
PVX_114450	0.016473156	0	1.686916958
PVX_114455	1	0	0
PVX_114460	0.036687443	0	0.903655982
PVX_114465	0.359317338	0	0.424575753
PVX_114470	0.001084333	0	2.511166198
PVX_114475	0.359317338	0	0.329415696
PVX_114480	3.76E-05	0	5.722999676
PVX_114485	0.166224591	0	0.744865731
PVX_114490	0.000125309	0	3.160497908
PVX_114495	0.166224591	0	0.455211221
PVX_114500	0.002855585	0	2.246111381
PVX_114505	0.359317338	0	0.470872174
PVX_114510	0.166224591	0	0.139094446
PVX_114512	0.007054102	0	1.270188892
PVX_114515	0.000383392	0	4.103802341
PVX_114520	1	0	0
PVX_114525	0.166224591	0	0.682001299
PVX_114530	0.359317338	0	0.295374591
PVX_114535	0.002855585	0	2.459619151
PVX_114540	0.036687443	0	1.37433731
PVX_114545	0.016473156	0	1.207971624
PVX_114550	0.166224591	0	0.66659251
PVX_114555	0.000383392	0	3.088232503
PVX_114560	0.007054102	0	2.282284233
PVX_114565	0.002855585	0	2.595481627
PVX_114570	0.007054102	0	1.999014003
PVX_114575	0.001084333	0	2.707181805
PVX_114580	0.002855585	0	2.317805722
PVX_114585	0.000383392	0	1.727414702
PVX_114590	0.002855585	0	2.154952477
PVX_114595	0.166224591	0	0.721135395

GeneID	Pvalue	BackGroundPre	BackGroundDx
PVX_114600	0.359317338	0	0.270264518
PVX_114605	1	0	0
PVX_114610	0.359317338	0	0.416461379
PVX_114615	0.078805602	0	0.517205755
PVX_114620	0.359317338	0	0.346944119
PVX_114625	0.016473156	0	1.482651091
PVX_114630	0.007054102	0	1.892085664
PVX_114635	0.036687443	0	1.316727816
PVX_114640	1	0	0
PVX_114645	0.016473156	0	1.624822534
PVX_114650	0.078805602	0	0.9779272
PVX_114655	0.016473156	0	0.988669015
PVX_114660	0.007054102	0	2.009705454
PVX_114665	0.001084333	0	2.974332333
PVX_114670	0.002855585	0	2.691085297
PVX_114675	0.002855585	0	2.831836967
PVX_114680	3.76E-05	0	4.754604512
PVX_114685	3.76E-05	0	4.926383351
PVX_114690	0.166224591	0	0.615153325
PVX_114695	0.078805602	0	0.755387154
PVX_114700	0.001084333	0	2.7577975
PVX_114705	0.016473156	0	1.795139061
PVX_114710	0.000383392	0	3.719006254
PVX_114715	0.000383392	0	3.584426585
PVX_114720	1	0	0
PVX_114725	1	0	0
PVX_114730	0.166224591	0	0.573622059
PVX_114735	0.001084333	0	2.211172865
PVX_114740	0.166224591	0	0.465458282
PVX_114745	0.016473156	0	1.461478794
PVX_114747	1	0	0
PVX_114750	0.001084333	0	2.987137638
PVX_114755	0.359317338	0	0.285804065
PVX_114760	0.036687443	0	1.000650497
PVX_114765	1	0	0
PVX_114770	0.078805602	0	1.297921963
PVX_114775	0.036687443	0	1.44121098
PVX_114780	0.359317338	0	0.344230675
PVX_114785	0.359317338	0	0.266445135
PVX_114790	0.016473156	0	1.806974127
PVX_114795	1	0	0

GeneID	Pvalue	BackGroundPre	BackGroundDx
PVX_114800	0.166224591	0	0.375180841
PVX_114805	1	0	0
PVX_114810	0.359317338	0	0.26471655
PVX_114815	0.359317338	0	0.364126667
PVX_114820	0.359317338	0	0.076573946
PVX_114825	0.036687443	0	1.607872206
PVX_114830	1.03E-05	0	6.700151157
PVX_114832	1.03E-05	0	6.672358949
PVX_114835	0.036687443	0	1.062612428
PVX_114840	1	0	0
PVX_114845	3.76E-05	0	3.739291164
PVX_114850	0.016473156	0	1.106914705
PVX_114855	0.166224591	0	0.777146641
PVX_114860	0.000383392	0	2.61626413
PVX_114865	0.000383392	0	3.687109084
PVX_114870	0.007054102	0	2.365076642
PVX_114875	0.016473156	0	1.189162439
PVX_114880	0.002855585	0	2.256474035
PVX_114885	0.016473156	0	1.282025368
PVX_114890	0.016473156	0	1.420100469
PVX_114892	1	0	0
PVX_114895	0.002855585	0	2.142660156
PVX_114900	0.078805602	0	0.831607082
PVX_114905	0.359317338	0	0.353885291
PVX_114910	3.76E-05	0	6.125748519
PVX_114920	0.001084333	0	3.120274796
PVX_114922	0.000125309	0	2.49835925
PVX_114925	0.078805602	0	0.866774073
PVX_114930	0.078805602	0	1.054399085
PVX_114935	0.007054102	0	1.907143131
PVX_114940	0.001084333	0	3.092032486
PVX_114945	0.359317338	0	0.37164006
PVX_114950	0.002855585	0	1.758668632
PVX_114955	0.166224591	0	0.282275076
PVX_114960	0.359317338	0	0.449446232
PVX_114965	0.016473156	0	1.329462903
PVX_114970	0.078805602	0	1.183487624
PVX_114975	0.016473156	0	1.905941054
PVX_114977	0.036687443	0	1.583710925
PVX_114980	0.036687443	0	1.396705307
PVX_114985	0.016473156	0	1.698259473

GeneID	Pvalue	BackGroundPre	BackGroundDx
PVX_114990	1	0	0
PVX_114995	0.078805602	0	0.786383024
PVX_115000	3.76E-05	0	4.468399556
PVX_115005	0.002855585	0	2.791156613
PVX_115010	0.016473156	0	1.511944827
PVX_115015	0.000383392	0	3.117552105
PVX_115020	0.002855585	0	1.856454676
PVX_115025	1	0	0
PVX_115030	1	0	0
PVX_115035	1	0	0
PVX_115040	0.166224591	0	0.931633771
PVX_115045	0.002855585	0	3.280061159
PVX_115050	0.166224591	0	0.285996581
PVX_115055	0.001084333	0	3.303113541
PVX_115060	0.166224591	0	0.83181995
PVX_115063	1	0	0
PVX_115065	0.007054102	0	2.0848828
PVX_115070	0.166224591	0	0.665366024
PVX_115075	0.000383392	0	2.685996041
PVX_115080	0.166224591	0	0.495291856
PVX_115085	0.166224591	0	0.870800656
PVX_115090	0.078805602	0	0.76398423
PVX_115095	0.359317338	0	0.111665936
PVX_115100	0.359317338	0	0.186561728
PVX_115105	0.078805602	0	0.724402151
PVX_115110	0.007054102	0	1.608522335
PVX_115115	0.036687443	0	1.484785309
PVX_115120	0.036687443	0	1.145548035
PVX_115125	0.007054102	0	2.080739998
PVX_115130	0.016473156	0	1.717096142
PVX_115135	0.002855585	0	2.741273044
PVX_115140	0.016473156	0	1.603797532
PVX_115145	0.359317338	0	0.24811202
PVX_115155	3.76E-05	0	5.049567613
PVX_115160	0.007054102	0	1.510894445
PVX_115165	1	0	0
PVX_115170	0.007054102	0	1.396619562
PVX_115175	0.002855585	0	1.913285484
PVX_115180	0.001084333	0	1.989221581
PVX_115185	0.166224591	0	0.575034662
PVX_115190	0.166224591	0	0.578738641

GeneID	Pvalue	BackGroundPre	BackGroundDx
PVX_115195	0.359317338	0	0.305630962
PVX_115200	0.007054102	0	2.400370676
PVX_115205	0.078805602	0	1.169301276
PVX_115210	0.016473156	0	1.549980223
PVX_115215	0.007054102	0	2.376990876
PVX_115220	0.016473156	0	2.601052824
PVX_115225	0.036687443	0	1.217976264
PVX_115230	0.036687443	0	1.116662234
PVX_115235	0.001084333	0	2.967942765
PVX_115240	0.078805602	0	0.91495881
PVX_115245	0.036687443	0	1.436027929
PVX_115250	0.359317338	0	0.289001733
PVX_115255	0.000383392	0	4.384953464
PVX_115260	0.166224591	0	1.129132406
PVX_115265	1	0	0
PVX_115270	0.036687443	0	1.650847907
PVX_115275	0.000383392	0	2.102391003
PVX_115280	0.001084333	0	2.748283832
PVX_115285	0.001084333	0	3.572818848
PVX_115290	0.007054102	0	2.038448758
PVX_115295	0.001084333	0	2.884520909
PVX_115300	0.166224591	0	0.55391035
PVX_115305	0.000125309	0	3.382647329
PVX_115310	0.016473156	0	2.221294706
PVX_115315	0.016473156	0	1.711921916
PVX_115320	1	0	0
PVX_115325	0.166224591	0	0.62264657
PVX_115330	0.036687443	0	1.041780524
PVX_115335	0.166224591	0	0.492648137
PVX_115340	0.078805602	0	1.185827887
PVX_115345	0.00037728	0.301955713	3.37246387
PVX_115350	1	0	0
PVX_115355	0.166224591	0	0.814009538
PVX_115360	1	0	0
PVX_115365	0.002855585	0	2.835740248
PVX_115370	0.002855585	0	2.528692
PVX_115375	0.002855585	0	2.956239571
PVX_115380	0.002855585	0	2.4779964
PVX_115385	0.016473156	0	1.700683384
PVX_115390	0.078805602	0	1.52039599
PVX_115395	0.016473156	0	1.423299085

GeneID	Pvalue	BackGroundPre	BackGroundDx
PVX_115400	0.016473156	0	1.331847242
PVX_115405	0.007054102	0	1.973603256
PVX_115410	0.036687443	0	1.197789959
PVX_115415	0.016473156	0	1.740305957
PVX_115420	0.166224591	0	0.435079577
PVX_115425	0.359317338	0	0.407751229
PVX_115430	0.002855585	0	1.292383962
PVX_115435	0.007054102	0	1.857578441
PVX_115440	0.007054102	0	1.880564995
PVX_115445	0.359317338	0	0.373191571
PVX_115446	1	0	0
PVX_115447	1	0	0
PVX_115448	1	0	0
PVX_115449	1	0	0
PVX_115449a	1	0	0
PVX_115450	7.86E-05	0.401900215	5.516390637
PVX_115455	0.359317338	0	0.332595081
PVX_115460	0.001084333	0	3.16322597
PVX_115465	0.007054102	0	2.101454179
PVX_115470	0.000125309	0	4.108222126
PVX_115475	0.359317338	0	0.290237592
PVX_115480	1	0	0
PVX_115490	1	0	0
PVX_115985	0.359317338	0	0.25629817
PVX_115990	1	0	0
PVX_116485	1	0	0
PVX_116490	0.007054102	0	2.010441406
PVX_116495	0.359317338	0	0.283015403
PVX_116500	0.036687443	0	1.720853727
PVX_116505	0.359317338	0	0.211536626
PVX_116510	0.016473156	0	1.904109687
PVX_116515	0.359317338	0	0.133664082
PVX_116520	0.078805602	0	0.78835302
PVX_116525	0.359317338	0	0.411233135
PVX_116530	0.007054102	0	1.743927617
PVX_116535	0.036687443	0	0.970732561
PVX_116540	0.016473156	0	1.850215596
PVX_116545	0.166224591	0	0.578567198
PVX_116550	0.007054102	0	1.048120536
PVX_116555	1	0	0
PVX_116557	0.002855585	0	2.731631016

GeneID	Pvalue	BackGroundPre	BackGroundDx
PVX_116560	0.001084333	0	2.774645022
PVX_116565	1	0	0
PVX_116570	1	0	0
PVX_116575	1	0	0
PVX_116580	0.078805602	0	1.093478304
PVX_116582	0.078805602	0	0.982198079
PVX_116585	0.359317338	0	0.194548556
PVX_116590	0.359317338	0	0.170677084
PVX_116595	0.036687443	0	1.6391412
PVX_116600	0.078805602	0	1.150714793
PVX_116603	0.036687443	0	1.206770197
PVX_116604	0.036687443	0	0.873780377
PVX_116610	0.000383392	0	3.997533972
PVX_116615	0.078805602	0	1.269372079
PVX_116620	0.000125309	0	4.862426331
PVX_116625	0.078805602	0	1.313429917
PVX_116630	1.03E-05	0	7.973390684
PVX_116635	0.000125309	0	4.117674937
PVX_116640	0.359317338	0	0.467061277
PVX_116645	0.166224591	0	0.452822329
PVX_116650	0.016473156	0	1.223818404
PVX_116652	0.078805602	0	1.254668285
PVX_116655	0.036687443	0	0.704729629
PVX_116660	0.002855585	0	2.567421266
PVX_116665	0.166224591	0	0.708485999
PVX_116670	0.001084333	0	2.507231809
PVX_116675	0.002855585	0	2.775078588
PVX_116680	0.007054102	0	1.931966788
PVX_116685	1	0	0
PVX_116690	0.001084333	0	2.846710693
PVX_116695	0.036687443	0	1.296117383
PVX_116700	1.03E-05	0	7.051506216
PVX_116705	0.078805602	0	1.030095767
PVX_116710	0.002855585	0	2.976322928
PVX_116715	3.76E-05	0	6.159365832
PVX_116720	0.359317338	0	0.456382771
PVX_116725	1	0	0
PVX_116730	1	0	0
PVX_116735	0.359317338	0	0.253886208
PVX_116740	1	0	0
PVX_116745	0.002855585	0	2.286697193

GeneID	Pvalue	BackGroundPre	BackGroundDx
PVX_116750	1	0	0
PVX_116755	0.166224591	0	0.415452348
PVX_116760	0.007054102	0	1.270237689
PVX_116765	0.166224591	0	0.352414801
PVX_116770	0.036687443	0	1.422460764
PVX_116775	0.000383392	0	3.132733299
PVX_116780	0.007054102	0	2.304023218
PVX_116785	0.007054102	0	1.87983972
PVX_116790	0.036687443	0	1.297478357
PVX_116795	1	0	0
PVX_116800	0.078805602	0	1.272523846
PVX_116805	0.007054102	0	1.984831117
PVX_116810	0.359317338	0	0.219164445
PVX_116815	1	0	0
PVX_116820	0.007054102	0	2.526136111
PVX_116825	0.036687443	0	1.367745289
PVX_116830	0.016473156	0	1.597971964
PVX_116835	0.001084333	0	3.796036432
PVX_116840	0.036687443	0	1.244350024
PVX_116845	0.002855585	0	2.208701631
PVX_116850	0.036687443	0	1.269253839
PVX_116855	0.359317338	0	0.360134411
PVX_116860	0.166224591	0	0.593995378
PVX_116865	0.001084333	0	2.799473671
PVX_116870	0.016473156	0	1.179588729
PVX_116875	0.007054102	0	2.603948193
PVX_116880	0.036687443	0	1.782267394
PVX_116885	0.359317338	0	0.266709693
PVX_116890	0.359317338	0	0.299481896
PVX_116892	0.359317338	0	0.280438849
PVX_116894	1	0	0
PVX_116895	1	0	0
PVX_116900	0.166224591	0	0.640473814
PVX_116905	0.036687443	0	1.360611746
PVX_116910	0.166224591	0	0.502310877
PVX_116915	0.078805602	0	1.196765557
PVX_116920	0.036687443	0	1.318260364
PVX_116925	0.002855585	0	3.272900802
PVX_116930	0.016473156	0	1.495063214
PVX_116935	0.000383392	0	2.945462675
PVX_116940	0.078805602	0	1.102552375

GeneID	Pvalue	BackGroundPre	BackGroundDx
PVX_116945	0.166224591	0	0.507631377
PVX_116950	0.359317338	0	0.331071536
PVX_116955	0.166224591	0	0.600288034
PVX_116960	0.036687443	0	1.670165514
PVX_116965	0.016473156	0	1.295507306
PVX_116967	0.078805602	0	1.420736835
PVX_116970	0.078805602	0	0.802893013
PVX_116975	0.016473156	0	2.163130923
PVX_116980	0.036687443	0	1.507213767
PVX_116985	0.166224591	0	0.216439549
PVX_116990	0.166224591	0	0.494027415
PVX_116995	1	0	0
PVX_117000	0.007054102	0	2.257539252
PVX_117005	0.359317338	0	0.248458881
PVX_117010	0.016473156	0	1.734999336
PVX_117015	0.036687443	0	1.909055657
PVX_117020	0.002855585	0	2.148677988
PVX_117025	0.001084333	0	2.703702321
PVX_117030	0.000125309	0	4.666844101
PVX_117035	1	0	0
PVX_117040	0.359317338	0	0.363445777
PVX_117045	1	0	0
PVX_117050	0.359317338	0	0.379195186
PVX_117055	0.078805602	0	1.068660025
PVX_117060	0.000383392	0	2.469320519
PVX_117062	0.002855585	0	1.872460161
PVX_117065	0.016473156	0	1.400983169
PVX_117070	1	0	0
PVX_117075	0.036687443	0	1.921133994
PVX_117080	1	0	0
PVX_117085	0.166224591	0	0.604755625
PVX_117090	0.002855585	0	2.660706829
PVX_117095	0.002855585	0	2.870195524
PVX_117100	0.036687443	0	1.432779549
PVX_117105	0.007054102	0	2.199401352
PVX_117110	0.078805602	0	0.907606827
PVX_117115	0.166224591	0	0.616506629
PVX_117120	0.078805602	0	0.741659347
PVX_117125	0.016473156	0	1.34573617
PVX_117130	0.359317338	0	0.38454881
PVX_117135	1	0	0

GeneID	Pvalue	BackGroundPre	BackGroundDx
PVX_117140	0.166224591	0	0.800011303
PVX_117145	0.007054102	0	1.616617894
PVX_117150	0.002855585	0	2.384237178
PVX_117155	0.166224591	0	0.286652329
PVX_117160	0.036687443	0	1.121079389
PVX_117165	0.359317338	0	0.422010986
PVX_117170	3.76E-05	0	6.230526931
PVX_117175	1	0	0
PVX_117180	1	0	0
PVX_117185	0.166224591	0	0.670264974
PVX_117190	0.166224591	0	0.492855553
PVX_117192	0.078805602	0	1.828675188
PVX_117195	0.007054102	0	1.44508616
PVX_117200	0.036687443	0	1.364276028
PVX_117205	0.007054102	0	1.989943187
PVX_117210	0.002855585	0	3.131590813
PVX_117215	0.016473156	0	1.693236181
PVX_117220	0.359317338	0	0.441690037
PVX_117225	0.016473156	0	1.993790691
PVX_117230	0.007054102	0	1.585502119
PVX_117240	0.036687443	0	0.843273
PVX_117245	0.036687443	0	1.841161168
PVX_117250	0.036687443	0	1.119602935
PVX_117255	0.166224591	0	0.524557809
PVX_117260	0.036687443	0	1.022861057
PVX_117265	0.002855585	0	2.108028774
PVX_117270	0.166224591	0	0.472384587
PVX_117275	0.078805602	0	1.474847189
PVX_117280	0.166224591	0	0.711038794
PVX_117285	0.359317338	0	0.341964322
PVX_117290	0.359317338	0	0.197301801
PVX_117292	0.078805602	0	0.906442823
PVX_117295	0.016473156	0	1.487765193
PVX_117300	0.002855585	0	2.750083362
PVX_117310	0.078805602	0	0.363130587
PVX_117315	0.078805602	0	1.076636792
PVX_117320	0.036687443	0	1.208505474
PVX_117322	1.03E-05	0	8.566344518
PVX_117325	0.016473156	0	1.54159779
PVX_117330	0.359317338	0	0.307142453
PVX_117335	1	0	0

GeneID	Pvalue	BackGroundPre	BackGroundDx
PVX_117340	0.036687443	0	0.510755293
PVX_117345	0.036687443	0	1.117753972
PVX_117350	0.359317338	0	0.564816885
PVX_117356	0.078805602	0	0.793195383
PVX_117357	0.359317338	0	0.431396643
PVX_117360	0.001084333	0	2.431133498
PVX_117365	0.166224591	0	0.341524269
PVX_117370	1	0	0
PVX_117375	0.016473156	0	1.684257014
PVX_117380	0.359317338	0	0.358224721
PVX_117385	0.166224591	0	0.607186332
PVX_117390	0.000125309	0	5.261270236
PVX_117395	0.002855585	0	3.036847898
PVX_117400	0.359317338	0	0.245578426
PVX_117405	0.016473156	0	1.553474115
PVX_117410	0.007054102	0	2.049644615
PVX_117415	0.166224591	0	0.576847204
PVX_117420	3.76E-05	0	5.353182632
PVX_117425	1	0	0
PVX_117430	1	0	0
PVX_117435	0.016473156	0	1.778082605
PVX_117440	3.76E-05	0	6.348203455
PVX_117445	0.016473156	0	2.024553586
PVX_117450	0.359317338	0	0.201966538
PVX_117455	0.078805602	0	1.069392611
PVX_117460	1	0	0
PVX_117465	1	0	0
PVX_117470	0.000125309	0	4.399926254
PVX_117475	0.036687443	0	1.666830514
PVX_117480	0.007054102	0	2.033429545
PVX_117485	0.078805602	0	0.753410965
PVX_117490	0.001084333	0	3.034265291
PVX_117495	0.078805602	0	0.998566768
PVX_117500	0.016473156	0	1.795726572
PVX_117505	0.002855585	0	2.161741922
PVX_117510	0.000383392	0	3.382496035
PVX_117515	0.359317338	0	0.349083079
PVX_117525	0.016473156	0	1.340048694
PVX_117530	0.016473156	0	1.82117168
PVX_117535	0.007054102	0	1.826553526
PVX_117540	0.166224591	0	0.576123254

GeneID	Pvalue	BackGroundPre	BackGroundDx
PVX_117545	0.166224591	0	0.68303906
PVX_117550	0.002855585	0	2.062944351
PVX_117555	0.036687443	0	1.924559204
PVX_117560	0.166224591	0	1.369161182
PVX_117565	0.001084333	0	2.290477074
PVX_117570	0.078805602	0	0.477817335
PVX_117575	0.359317338	0	0.28134922
PVX_117580	0.001084333	0	2.193780496
PVX_117585	0.002855585	0	2.282072084
PVX_117590	0.359317338	0	0.281045914
PVX_117595	0.016473156	0	1.147397087
PVX_117600	0.002855585	0	2.627377593
PVX_117605	0.002855585	0	3.761787309
PVX_117610	0.166224591	0	0.463654028
PVX_117615	0.002855585	0	2.742448854
PVX_117620	0.078805602	0	1.185494198
PVX_117625	0.002855585	0	2.3867797
PVX_117630	0.016473156	0	1.903290395
PVX_117635	0.016473156	0	2.091311507
PVX_117640	0.036687443	0	1.032290246
PVX_117645	0.359317338	0	0.207042214
PVX_117650	0.007054102	0	2.18552209
PVX_117655	0.002855585	0	2.534580781
PVX_117660	0.359317338	0	0.337424967
PVX_117665	1	0	0
PVX_117670	0.078805602	0	1.131682712
PVX_117675	0.166224591	0	0.377979672
PVX_117680	0.166224591	0	0.557987189
PVX_117685	1	0	0
PVX_117690	0.002855585	0	2.156582413
PVX_117695	0.001084333	0	3.045110071
PVX_117700	1	0	0
PVX_117705	0.359317338	0	0.311252933
PVX_117710	1	0	0
PVX_117715	0.166224591	0	0.826694169
PVX_117720	1	0	0
PVX_117725	0.359317338	0	0.205890079
PVX_117730	1	0	0
PVX_117735	1	0	0
PVX_117740	1	0	0
PVX_117745	3.76E-05	0	5.096069537

GeneID	Pvalue	BackGroundPre	BackGroundDx
PVX_117750	0.001084333	0	3.173701823
PVX_117755	0.078805602	0	1.087852321
PVX_117760	0.000383392	0	3.415934713
PVX_117765	1	0	0
PVX_117770	0.001084333	0	2.314051091
PVX_117775	0.078805602	0	1.337603913
PVX_117780	0.002855585	0	2.095794833
PVX_117785	0.078805602	0	0.873214861
PVX_117790	0.000125309	0	3.468525872
PVX_117795	1.03E-05	0	6.603166138
PVX_117800	0.078805602	0	0.625609848
PVX_117805	1	0	0
PVX_117810	0.016473156	0	1.246971577
PVX_117812	1	0	0
PVX_117815	0.359317338	0	0.131738113
PVX_117825	0.359317338	0	0.198626719
PVX_117830	0.002855585	0	2.203396031
PVX_117835	0.166224591	0	0.739788247
PVX_117840	0.036687443	0	1.57329668
PVX_117845	0.007054102	0	2.238128276
PVX_117850	0.007054102	0	1.307915472
PVX_117855	0.007054102	0	2.446559957
PVX_117860	0.001084333	0	2.294866637
PVX_117865	0.359317338	0	0.293542171
PVX_117870	0.036687443	0	1.353004732
PVX_117875	0.036687443	0	1.460022353
PVX_117880	0.166224591	0	0.345881547
PVX_117885	0.007054102	0	1.581016334
PVX_117890	0.036687443	0	1.066487423
PVX_117895	1	0	0
PVX_117900	1	0	0
PVX_117905	0.078805602	0	1.085923094
PVX_117910	0.007054102	0	1.408006721
PVX_117915	0.007054102	0	2.615850801
PVX_117920	0.000383392	0	2.638655385
PVX_117925	1.42E-05	0.331698529	5.972543249
PVX_117930	0.036687443	0	1.276356157
PVX_117935	1	0	0
PVX_117940	0.036687443	0	1.550118559
PVX_117945	0.007054102	0	1.813944652
PVX_117950	0.001084333	0	2.707906307

GeneID	Pvalue	BackGroundPre	BackGroundDx
PVX_117960	0.036687443	0	0.764208154
PVX_117965	0.000383392	0	2.543722392
PVX_117970	0.007054102	0	1.598119272
PVX_117975	0.359317338	0	0.281308482
PVX_117980	0.007054102	0	2.111425618
PVX_117985	0.166224591	0	0.601636278
PVX_117990	0.078805602	0	0.875724155
PVX_117995	0.016473156	0	1.780874741
PVX_118000	0.036687443	0	0.901271218
PVX_118005	1	0	0
PVX_118010	1	0	0
PVX_118015	0.359317338	0	0.182403667
PVX_118020	0.036687443	0	0.915323664
PVX_118025	0.000383392	0	3.080727862
PVX_118030	0.359317338	0	0.529416265
PVX_118035	0.359317338	0	0.234322461
PVX_118040	1	0	0
PVX_118045	0.359317338	0	0.340187504
PVX_118050	0.078805602	0	0.994925543
PVX_118055	0.007054102	0	2.190395831
PVX_118060	0.078805602	0	1.065461073
PVX_118062	0.000383392	0	2.309589601
PVX_118065	0.002855585	0	2.086006934
PVX_118070	0.036687443	0	0.70525962
PVX_118075	1	0	0
PVX_118080	0.016473156	0	1.47021878
PVX_118090	0.166224591	0	0.783596278
PVX_118095	0.007054102	0	2.034248038
PVX_118100	0.016473156	0	1.145493611
PVX_118105	0.036687443	0	0.739469424
PVX_118110	0.016473156	0	2.030134144
PVX_118115	1	0	0
PVX_118120	1	0	0
PVX_118125	0.078805602	0	1.132667328
PVX_118130	0.166224591	0	0.809685635
PVX_118135	0.000125309	0	3.402340921
PVX_118140	0.016473156	0	2.087732132
PVX_118145	1.03E-05	0	6.67347106
PVX_118150	0.166224591	0	0.546296578
PVX_118155	0.016473156	0	1.934314619
PVX_118160	0.166224591	0	0.653441206

GeneID	Pvalue	BackGroundPre	BackGroundDx
PVX_118162	0.166224591	0	0.728071534
PVX_118165	0.001084333	0	1.973901482
PVX_118170	0.359317338	0	0.243163325
PVX_118175	0.359317338	0	0.182673262
PVX_118180	3.76E-05	0	5.284303939
PVX_118185	1	0	0
PVX_118190	0.001084333	0	3.43406924
PVX_118195	1	0	0
PVX_118200	0.001084333	0	3.272148772
PVX_118205	0.036687443	0	1.127810673
PVX_118210	0.166224591	0	0.758316952
PVX_118215	0.359317338	0	0.510249938
PVX_118220	0.007054102	0	1.827561135
PVX_118225	0.002855585	0	2.33859286
PVX_118230	0.016473156	0	1.285070815
PVX_118235	0.000383392	0	3.304259604
PVX_118240	0.016473156	0	1.903349354
PVX_118245	0.036687443	0	1.412305416
PVX_118255	0.000125309	0	5.644556454
PVX_118260	0.166224591	0	0.815525439
PVX_118265	1	0	0
PVX_118270	0.359317338	0	0.135260812
PVX_118275	0.036687443	0	1.183718286
PVX_118280	0.007054102	0	2.256389578
PVX_118285	0.359317338	0	0.360534238
PVX_118290	0.359317338	0	0.107494344
PVX_118292	1	0	0
PVX_118295	0.002855585	0	2.002554736
PVX_118300	0.007054102	0	1.853846093
PVX_118305	0.036687443	0	1.398461377
PVX_118310	0.359317338	0	0.332507262
PVX_118315	0.002855585	0	2.149919892
PVX_118320	0.002855585	0	2.389814911
PVX_118325	0.036687443	0	1.701789102
PVX_118330	0.078805602	0	0.853246108
PVX_118335	0.166224591	0	0.658112672
PVX_118340	0.036687443	0	1.078632072
PVX_118345	0.001084333	0	1.746363922
PVX_118350	0.078805602	0	1.067346213
PVX_118355	0.359317338	0	0.148985562
PVX_118360	0.016473156	0	0.914639824

GeneID	Pvalue	BackGroundPre	BackGroundDx
PVX_118365	0.036687443	0	1.053046206
PVX_118370	0.016473156	0	0.973218131
PVX_118375	0.001084333	0	3.3228943
PVX_118380	0.166224591	0	0.566306568
PVX_118385	0.007054102	0	2.476416697
PVX_118390	0.007054102	0	2.227272904
PVX_118395	0.359317338	0	0.588529536
PVX_118400	0.016473156	0	1.936653106
PVX_118405	0.078805602	0	0.945766242
PVX_118410	0.016473156	0	1.530705081
PVX_118415	0.359317338	0	0.207106798
PVX_118420	0.001084333	0	2.72846388
PVX_118425	1	0	0
PVX_118430	0.000383392	0	4.2974182
PVX_118435	3.76E-05	0	5.396257333
PVX_118440	1	0	0
PVX_118445	1	0	0
PVX_118450	0.002855585	0	2.381834338
PVX_118455	0.002855585	0	2.307957244
PVX_118460	1	0	0
PVX_118465	0.359317338	0	0.219165045
PVX_118470	0.359317338	0	0.277075543
PVX_118475	0.036687443	0	1.090689825
PVX_118480	0.078805602	0	1.015840127
PVX_118485	0.166224591	0	0.558875334
PVX_118490	0.166224591	0	0.375234996
PVX_118495	0.000125309	0	5.460869735
PVX_118500	0.036687443	0	1.47820774
PVX_118505	0.016473156	0	1.795621758
PVX_118510	0.016473156	0	1.84193537
PVX_118515	0.078805602	0	1.026166142
PVX_118520	0.007054102	0	2.503431193
PVX_118525	0.016473156	0	1.183701968
PVX_118530	0.166224591	0	0.705500575
PVX_118535	0.002855585	0	1.673870779
PVX_118540	0.007054102	0	2.028145095
PVX_118545	3.76E-05	0	6.559698631
PVX_118550	0.007054102	0	1.659666779
PVX_118560	0.016473156	0	1.634263882
PVX_118565	0.078805602	0	1.271888528
PVX_118570	0.078805602	0	0.572889088

GeneID	Pvalue	BackGroundPre	BackGroundDx
PVX_118575	0.036687443	0	0.929351527
PVX_118580	0.000125309	0	3.967157387
PVX_118585	1	0	0
PVX_118590	0.007054102	0	1.319035026
PVX_118595	0.007054102	0	1.672150943
PVX_118600	0.359317338	0	0.189206339
PVX_118605	0.036687443	0	1.352960712
PVX_118610	0.001084333	0	2.711918539
PVX_118615	0.036687443	0	1.426031945
PVX_118620	0.000125309	0	4.540655368
PVX_118625	0.016473156	0	1.65149295
PVX_118630	1	0	0
PVX_118635	0.007054102	0	1.753698148
PVX_118640	0.002855585	0	2.480533213
PVX_118645	0.078805602	0	1.065711894
PVX_118648	0.036687443	0	0.424246089
PVX_118650	0.166224591	0	0.558585012
PVX_118655	0.016473156	0	1.534830372
PVX_118660	0.166224591	0	0.575325305
PVX_118665	1	0	0
PVX_118670	0.166224591	0	0.525315493
PVX_118675	1	0	0
PVX_118680	1	0	0
PVX_118682	1	0	0
PVX_118685	0.078805602	0	1.362476489
PVX_118690	0.036687443	0	1.570981038
PVX_118695	0.000125309	0	4.160032463
PVX_118700	0.166224591	0	0.469064378
PVX_118705	0.036687443	0	1.422178169
PVX_119205	1	0	0
PVX_119210	0.359317338	0	0.181423308
PVX_119215	0.359317338	0	0.250480563
PVX_119220	0.000383392	0	3.738510926
PVX_119225	0.000125309	0	4.115724098
PVX_119230	0.007054102	0	2.244560811
PVX_119235	1	0	0
PVX_119240	0.078805602	0	0.938984446
PVX_119245	0.036687443	0	1.386475276
PVX_119250	0.002855585	0	2.296613415
PVX_119255	0.007054102	0	1.461024262
PVX_119260	0.036687443	0	1.56035227

GeneID	Pvalue	BackGroundPre	BackGroundDx
PVX_119265	0.002855585	0	2.584191709
PVX_119270	0.000125309	0	3.281685867
PVX_119275	0.001084333	0	2.785912376
PVX_119280	0.166224591	0	0.476180576
PVX_119285	0.016473156	0	1.462086036
PVX_119290	0.007054102	0	2.511333265
PVX_119295	0.078805602	0	0.825268345
PVX_119300	0.166224591	0	0.396682803
PVX_119305	0.036687443	0	1.627163285
PVX_119310	0.016473156	0	1.912614147
PVX_119315	0.359317338	0	0.329959599
PVX_119320	1	0	0
PVX_119325	1	0	0
PVX_119330	0.359317338	0	0.269564728
PVX_119335	0.036687443	0	1.237504383
PVX_119340	0.166224591	0	0.482061165
PVX_119345	0.000125309	0	5.834004476
PVX_119350	0.078805602	0	1.331003568
PVX_119355	1	0	0
PVX_119360	0.359317338	0	0.279908507
PVX_119365	0.036687443	0	1.268211246
PVX_119370	0.002855585	0	3.472970789
PVX_119375	0.036687443	0	1.361170998
PVX_119380	0.001084333	0	1.775536593
PVX_119385	0.016473156	0	1.462318515
PVX_119390	0.002855585	0	2.424480506
PVX_119395	0.002855585	0	3.131835386
PVX_119400	0.001084333	0	1.906015619
PVX_119405	0.359317338	0	0.328827146
PVX_119410	0.000125309	0	4.845885336
PVX_119415	0.166224591	0	0.495439243
PVX_119420	0.078805602	0	1.382901928
PVX_119425	0.359317338	0	0.306361343
PVX_119430	1	0	0
PVX_119435	0.001084333	0	3.181684622
PVX_119440	0.002855585	0	3.33939739
PVX_119445	0.016473156	0	1.573024962
PVX_119450	0.016473156	0	2.051186945
PVX_119455	0.036687443	0	1.551488169
PVX_119460	0.007054102	0	2.033811836
PVX_119465	3.76E-05	0	4.859767595

GeneID	Pvalue	BackGroundPre	BackGroundDx
PVX_119470	3.76E-05	0	6.922878292
PVX_119475	0.000383392	0	4.952235544
PVX_119480	3.76E-05	0	5.623058249
PVX_119485	0.166224591	0	0.478536573
PVX_119490	0.016473156	0	1.875884189
PVX_119495	0.016473156	0	1.825238933
PVX_119500	0.036687443	0	1.258675252
PVX_119505	0.036687443	0	0.787752985
PVX_119510	0.000383392	0	3.133604591
PVX_119515	0.016473156	0	0.717446473
PVX_119520	0.000383392	0	3.318647904
PVX_119525	0.166224591	0	0.428487638
PVX_119530	3.76E-05	0	4.670119094
PVX_119535	0.359317338	0	0.256060863
PVX_119540	0.078805602	0	1.191998667
PVX_119545	0.036687443	0	1.360461373
PVX_119550	0.359317338	0	0.28899004
PVX_119555	0.166224591	0	0.917032785
PVX_119560	0.007054102	0	1.65572642
PVX_119565	0.007054102	0	1.98119503
PVX_119570	0.359317338	0	0.33550161
PVX_119575	1	0	0
PVX_119580	0.078805602	0	0.742609424
PVX_119585	0.001084333	0	3.055305757
PVX_119587	0.001084333	0	5.042139059
PVX_119590	0.036687443	0	1.276025697
PVX_119595	0.036687443	0	1.346464337
PVX_119600	0.078805602	0	0.634281128
PVX_119605	0.166224591	0	0.751262703
PVX_119610	1	0	0
PVX_119615	3.76E-05	0	2.516245945
PVX_119620	0.001084333	0	2.404297754
PVX_119625	0.359317338	0	0.204742504
PVX_119630	3.76E-05	0	3.875324086
PVX_119635	0.016473156	0	1.637131279
PVX_119640	0.166224591	0	0.768397988
PVX_119645	0.166224591	0	0.870289454
PVX_119650	1	0	0
PVX_119655	1	0	0
PVX_119660	0.007054102	0	2.138067698
PVX_119665	0.007054102	0	1.727890912

GeneID	Pvalue	BackGroundPre	BackGroundDx
PVX_119670	0.016473156	0	1.367846249
PVX_119675	0.016473156	0	0.966127259
PVX_119680	0.000383392	0	4.227283618
PVX_119685	0.036687443	0	1.067407024
PVX_119690	0.166224591	0	0.617629572
PVX_119695	0.001084333	0	3.02738701
PVX_119700	0.359317338	0	0.211236585
PVX_119705	0.078805602	0	1.064388508
PVX_119710	0.002855585	0	2.610852622
PVX_119715	0.359317338	0	0.199249902
PVX_119720	0.001084333	0	3.627112502
PVX_119725	0.078805602	0	0.883222557
PVX_119735	0.016473156	0	1.791101736
PVX_119740	0.000125309	0	5.432409298
PVX_119745	0.007054102	0	2.399668619
PVX_119750	0.000383392	0	3.333946637
PVX_119755	0.007054102	0	2.663867221
PVX_119760	0.166224591	0	0.872533802
PVX_119765	0.016473156	0	1.676136784
PVX_119770	0.000383392	0	3.085370677
PVX_119775	0.000125309	0	4.083704379
PVX_119780	0.002855585	0	2.346814648
PVX_119785	0.016473156	0	1.620884345
PVX_119790	0.001084333	0	2.567261349
PVX_119795	0.016473156	0	1.913022175
PVX_119800	0.078805602	0	1.175910034
PVX_119805	0.359317338	0	0.239756519
PVX_119812	0.166224591	0	0.709181475
PVX_119813	0.002855585	0	1.788488422
PVX_119815	0.002855585	0	2.623771215
PVX_119820	0.001084333	0	3.214557016
PVX_119825	0.036687443	0	1.426703075
PVX_119830	1	0	0
PVX_120330	0.359317338	0	0.291843004
PVX_120335	0.166224591	0	0.498451174
PVX_120340	0.359317338	0	0.334060866
PVX_120840	0.166224591	0	0.678187112
PVX_120845	0.166224591	0	0.368445109
PVX_121345	1	0	0
PVX_121350	1	0	0
PVX_121355	1	0	0

GeneID	Pvalue	BackGroundPre	BackGroundDx
PVX_121855	0.359317338	0	0.180532656
PVX_121860	1	0	0
PVX_121862	1	0	0
PVX_121865	1	0	0
PVX_121870	0.359317338	0	0.500230866
PVX_121875	0.359317338	0	0.369891169
PVX_121876	0.078805602	0	0.955730187
PVX_121877	0.078805602	0	1.004064228
PVX_121878	1	0	0
PVX_121879	0.359317338	0	0.364126667
PVX_121880	0.000125309	0	3.938903294
PVX_121885	1	0	0
PVX_121890	0.000383392	0	4.499860053
PVX_121895	0.007054102	0	2.961088113
PVX_121897	0.078805602	0	1.172425625
PVX_121900	0.078805602	0	0.830365675
PVX_121905	0.166224591	0	0.764622745
PVX_121910	0.001084333	0	3.595249275
PVX_121912	0.166224591	0	0.674488243
PVX_121915	0.166224591	0	0.628075649
PVX_121920	0.166224591	0	0.363812267
PVX_121925	0.359317338	0	0.429532919
PVX_121927	1	0	0
PVX_121930	0.016473156	0	2.21004222
PVX_121935	0.000125309	0	5.617304561
PVX_121940	0.166224591	0	0.665392269
PVX_121945	0.036687443	0	1.499636332
PVX_121950	1	0	0
PVX_121955	0.078805602	0	1.010809745
PVX_121960	0.359317338	0	0.30044366
PVX_121965	0.016473156	0	1.157847774
PVX_121970	0.007054102	0	2.025107707
PVX_121975	0.016473156	0	1.3424075
PVX_121980	3.76E-05	0	5.990543882
PVX_121985	0.016473156	0	1.534431786
PVX_121990	0.036687443	0	1.518728913
PVX_121995	0.016473156	0	2.169556169
PVX_122000	0.078805602	0	1.139403084
PVX_122005	0.002855585	0	2.560995268
PVX_122010	0.016473156	0	1.487321895
PVX_122015	0.166224591	0	0.394295169

GeneID	Pvalue	BackGroundPre	BackGroundDx
PVX_122020	0.078805602	0	0.925816364
PVX_122025	0.016473156	0	1.471344398
PVX_122030	0.002855585	0	0.949756461
PVX_122035	1	0	0
PVX_122040	0.166224591	0	0.488636593
PVX_122045	3.76E-05	0	3.338562116
PVX_122050	1	0	0
PVX_122055	0.007054102	0	2.082127996
PVX_122060	0.359317338	0	0.257716694
PVX_122065	0.007054102	0	2.514084618
PVX_122070	1	0	0
PVX_122075	0.359317338	0	0.418613936
PVX_122077	0.359317338	0	0.14932926
PVX_122080	0.036687443	0	1.322019227
PVX_122085	0.036687443	0	1.00192229
PVX_122090	0.166224591	0	0.633200998
PVX_122095	0.002855585	0	2.640994925
PVX_122100	0.001084333	0	2.035537413
PVX_122105	0.016473156	0	1.576880349
PVX_122110	0.007054102	0	1.779867592
PVX_122115	0.078805602	0	0.986086349
PVX_122120	1	0	0
PVX_122125	1	0	0
PVX_122130	0.000125309	0	3.737354621
PVX_122135	1	0	0
PVX_122140	0.166224591	0	0.823872967
PVX_122145	0.078805602	0	0.903365269
PVX_122150	0.078805602	0	0.566570672
PVX_122155	0.001084333	0	3.156385038
PVX_122160	0.166224591	0	0.342937478
PVX_122165	0.007054102	0	2.153565285
PVX_122170	0.016473156	0	1.566187629
PVX_122175	0.166224591	0	0.574076023
PVX_122180	0.001084333	0	2.865585973
PVX_122185	0.007054102	0	2.251833141
PVX_122190	0.016473156	0	1.421486585
PVX_122195	0.078805602	0	0.86646368
PVX_122200	0.078805602	0	0.922009379
PVX_122205	1	0	0
PVX_122207	0.359317338	0	0.62255096
PVX_122210	0.007054102	0	2.169291979

GeneID	Pvalue	BackGroundPre	BackGroundDx
PVX_122215	0.036687443	0	0.855870615
PVX_122218	0.001084333	0	3.510149338
PVX_122222	0.036687443	0	1.069011274
PVX_122225	0.036687443	0	1.164830338
PVX_122230	1	0	0
PVX_122235	0.078805602	0	1.218606748
PVX_122240	3.76E-05	0	3.406793355
PVX_122245	1.03E-05	0	6.555894513
PVX_122250	0.002855585	0	1.145986249
PVX_122255	0.078805602	0	0.790183568
PVX_122260	0.036687443	0	0.86237369
PVX_122265	1	0	0
PVX_122270	0.359317338	0	0.273579207
PVX_122275	0.036687443	0	0.951551347
PVX_122280	0.001084333	0	2.744109257
PVX_122285	0.000125309	0	5.522805938
PVX_122290	0.001084333	0	2.635685521
PVX_122295	0.016473156	0	1.550359766
PVX_122300	0.078805602	0	0.74216057
PVX_122305	0.359317338	0	0.423583797
PVX_122310	0.078805602	0	1.035348737
PVX_122315	0.000125309	0	3.000950725
PVX_122320	0.036687443	0	1.223030865
PVX_122325	0.359317338	0	0.37699261
PVX_122330	0.002855585	0	2.538282176
PVX_122335	0.036687443	0	1.34562717
PVX_122340	0.359317338	0	0.27564895
PVX_122345	0.078805602	0	1.303697124
PVX_122350	0.001084333	0	2.489685421
PVX_122355	0.007054102	0	2.255335892
PVX_122360	0.078805602	0	1.145072876
PVX_122365	0.078805602	0	1.148145948
PVX_122370	0.002855585	0	1.770784203
PVX_122375	0.002855585	0	2.835682433
PVX_122380	0.166224591	0	0.902089705
PVX_122385	0.359317338	0	0.200576507
PVX_122390	0.166224591	0	0.646948415
PVX_122395	0.016473156	0	1.836426325
PVX_122405	0.002855585	0	2.682856023
PVX_122410	0.000383392	0	3.483292818
PVX_122415	0.036687443	0	1.068552369

GeneID	Pvalue	BackGroundPre	BackGroundDx
PVX_122420	0.166224591	0	0.801404234
PVX_122425	3.76E-05	0	5.648754269
PVX_122430	0.016473156	0	1.636974281
PVX_122435	0.078805602	0	0.886883953
PVX_122440	0.166224591	0	0.335786417
PVX_122445	0.016473156	0	1.616838794
PVX_122450	0.036687443	0	1.350940703
PVX_122455	0.016473156	0	1.245139148
PVX_122458	1	0	0
PVX_122460	0.036687443	0	1.493941598
PVX_122465	0.078805602	0	0.598597684
PVX_122470	3.76E-05	0	4.056210162
PVX_122475	0.359317338	0	0.336008949
PVX_122480	1	0	0
PVX_122485	0.001084333	0	2.394095135
PVX_122487	0.359317338	0	0.309586404
PVX_122490	0.036687443	0	1.343771887
PVX_122495	0.166224591	0	0.313948512
PVX_122500	0.359317338	0	0.161760967
PVX_122505	0.078805602	0	0.890814201
PVX_122510	0.002855585	0	1.399499893
PVX_122515	0.016473156	0	1.668188395
PVX_122520	0.016473156	0	0.991962244
PVX_122525	0.007054102	0	2.012497652
PVX_122530	0.016473156	0	1.08365805
PVX_122535	0.002855585	0	2.521000111
PVX_122540	0.359317338	0	0.435715113
PVX_122545	0.078805602	0	0.960844371
PVX_122550	0.001084333	0	3.020894389
PVX_122555	0.001084333	0	2.92478198
PVX_122560	0.016473156	0	2.08876543
PVX_122565	0.016473156	0	1.772541077
PVX_122570	0.036687443	0	1.491990047
PVX_122575	0.078805602	0	0.996357024
PVX_122580	0.166224591	0	0.439478852
PVX_122585	1	0	0
PVX_122590	0.000125309	0	4.478019822
PVX_122600	0.036687443	0	1.399498893
PVX_122605	0.000125309	0	3.755539792
PVX_122610	0.002855585	0	2.762885367
PVX_122615	0.000383392	0	3.537068599

GeneID	Pvalue	BackGroundPre	BackGroundDx
PVX_122620	0.000125309	0	3.859663584
PVX_122625	1	0	0
PVX_122630	0.016473156	0	1.196589157
PVX_122635	1	0	0
PVX_122637	1	0	0
PVX_122640	0.359317338	0	0.259540311
PVX_122645	0.002855585	0	2.047983523
PVX_122650	0.007054102	0	1.727365654
PVX_122655	0.359317338	0	0.366492383
PVX_122660	0.007054102	0	2.465953945
PVX_122665	0.036687443	0	1.178448388
PVX_122670	0.002855585	0	2.330702959
PVX_122675	0.016473156	0	1.438695269
PVX_122680	0.001084333	0	2.062921047
PVX_122685	0.000383392	0	3.426830699
PVX_122690	0.007054102	0	2.468231388
PVX_122695	1	0	0
PVX_122700	0.036687443	0	1.190655548
PVX_122705	0.078805602	0	1.52650928
PVX_122710	0.000125309	0	5.430185553
PVX_122715	0.036687443	0	1.099961686
PVX_122720	1	0	0
PVX_122725	0.002855585	0	2.577575806
PVX_122730	1	0	0
PVX_122735	0.166224591	0	0.522268169
PVX_122740	0.036687443	0	1.179115001
PVX_122742	1	0	0
PVX_122745	0.078805602	0	0.954120044
PVX_122750	0.359317338	0	0.181618174
PVX_122755	0.002855585	0	2.461615291
PVX_122760	0.359317338	0	0.201835008
PVX_122765	0.016473156	0	1.307625351
PVX_122770	0.002855585	0	2.111592154
PVX_122775	0.166224591	0	0.691745514
PVX_122780	0.000383392	0	2.960650684
PVX_122785	0.007054102	0	1.666479763
PVX_122790	0.359317338	0	0.406385951
PVX_122795	0.078805602	0	0.819840214
PVX_122800	0.166224591	0	0.594945534
PVX_122805	0.359317338	0	0.197375404
PVX_122810	0.016473156	0	1.388299972

GeneID	Pvalue	BackGroundPre	BackGroundDx
PVX_122815	0.016473156	0	1.875985103
PVX_122820	0.166224591	0	0.686039192
PVX_122825	0.359317338	0	0.31006905
PVX_122830	0.078805602	0	1.153914009
PVX_122835	0.359317338	0	0.417156444
PVX_122840	0.016473156	0	1.857384024
PVX_122845	0.166224591	0	0.405761526
PVX_122850	0.007054102	0	2.177425626
PVX_122855	0.036687443	0	1.669166921
PVX_122860	1	0	0
PVX_122865	1	0	0
PVX_122870	0.078805602	0	1.236134413
PVX_122875	0.000383392	0	2.126436013
PVX_122880	0.359317338	0	0.274005501
PVX_122885	0.166224591	0	0.527797645
PVX_122890	1	0	0
PVX_122895	0.016473156	0	1.982799933
PVX_122900	1	0	0
PVX_122905	0.002855585	0	2.225191104
PVX_122910	0.016473156	0	2.134095236
PVX_122915	0.166224591	0	0.715501994
PVX_122920	0.001084333	0	2.138324795
PVX_122925	1	0	0
PVX_122930	0.016473156	0	1.981291065
PVX_122935	0.359317338	0	0.394111069
PVX_122940	0.078805602	0	0.657872517
PVX_122945	0.002855585	0	2.041692028
PVX_122947	0.166224591	0	0.872035863
PVX_122950	0.002855585	0	2.220683591
PVX_122955	0.078805602	0	0.83971237
PVX_122956	1	0	0
PVX_122957	1	0	0
PVX_122958	1	0	0
PVX_122960	0.007054102	0	2.297335447
PVX_122962	0.359317338	0	0.605866946
PVX_122965	0.166224591	0	0.350454506
PVX_122970	1	0	0
PVX_122975	1	0	0
PVX_122980	0.166224591	0	0.779785683
PVX_122985	0.002855585	0	2.052866639
PVX_122990	0.166224591	0	0.958899111

GeneID	Pvalue	BackGroundPre	BackGroundDx
PVX_122995	0.000125309	0	3.464600994
PVX_123000	0.001084333	0	2.755608644
PVX_123005	0.016473156	0	1.456954195
PVX_123010	0.001084333	0	2.940232883
PVX_123015	1	0	0
PVX_123020	0.078805602	0	0.775954517
PVX_123025	0.000383392	0	3.66600045
PVX_123030	0.001084333	0	3.347125826
PVX_123035	1	0	0
PVX_123040	1	0	0
PVX_123045	1	0	0
PVX_123050	0.078805602	0	0.692402168
PVX_123055	0.007054102	0	1.704568096
PVX_123060	1.03E-05	0	7.164112637
PVX_123065	0.036687443	0	1.244546146
PVX_123070	1.03E-05	0	6.656510573
PVX_123075	1.03E-05	0	6.016681524
PVX_123080	0.166224591	0	0.612821057
PVX_123085	0.016473156	0	1.195726041
PVX_123090	0.002855585	0	2.54095775
PVX_123095	1	0	0
PVX_123100	0.001084333	0	2.909251842
PVX_123105	0.000125309	0	4.265769857
PVX_123110	0.000383392	0	4.235329456
PVX_123115	0.016473156	0	1.591351586
PVX_123120	1	0	0
PVX_123125	0.359317338	0	0.246594211
PVX_123130	0.166224591	0	0.802844291
PVX_123135	0.036687443	0	1.311472254
PVX_123140	0.166224591	0	0.584208572
PVX_123145	0.001084333	0	2.661346259
PVX_123150	0.007054102	0	2.230501553
PVX_123155	0.359317338	0	0.285223739
PVX_123158	0.359317338	0	0.219782638
PVX_123162	0.016473156	0	1.874457591
PVX_123165	0.000383392	0	2.499412711
PVX_123170	0.007054102	0	1.967077046
PVX_123175	0.016473156	0	1.115868349
PVX_123180	0.166224591	0	0.47870964
PVX_123185	0.036687443	0	1.09165517
PVX_123190	0.166224591	0	0.372936407

GeneID	Pvalue	BackGroundPre	BackGroundDx
PVX_123195	0.036687443	0	0.914990319
PVX_123200	0.007054102	0	2.276435793
PVX_123205	0.007054102	0	1.086186523
PVX_123210	0.016473156	0	1.351022972
PVX_123215	0.036687443	0	1.198560302
PVX_123220	0.078805602	0	0.860776249
PVX_123225	1	0	0
PVX_123230	0.002855585	0	2.821667996
PVX_123235	0.166224591	0	0.959780562
PVX_123240	1.03E-05	0	5.319184801
PVX_123245	0.036687443	0	1.325951322
PVX_123250	0.002855585	0	2.349848325
PVX_123260	0.001084333	0	2.705983352
PVX_123265	0.036687443	0	1.343220649
PVX_123270	0.036687443	0	1.526697418
PVX_123275	0.007054102	0	2.291082132
PVX_123283	0.036687443	0	0.903425379
PVX_123290	0.078805602	0	0.549682059
PVX_123295	0.016473156	0	1.533631124
PVX_123300	0.166224591	0	0.209710395
PVX_123305	0.078805602	0	1.184913358
PVX_123307	0.016473156	0	1.569191269
PVX_123310	0.000383392	0	2.56436716
PVX_123315	0.078805602	0	0.840889699
PVX_123320	0.036687443	0	0.923874755
PVX_123325	0.036687443	0	1.254031451
PVX_123330	0.078805602	0	0.924989252
PVX_123335	0.036687443	0	1.590197264
PVX_123340	0.000125309	0	3.893486153
PVX_123345	0.166224591	0	0.661607152
PVX_123350	0.078805602	0	0.877633977
PVX_123355	0.001084333	0	3.35855977
PVX_123357	0.166224591	0	0.963594196
PVX_123360	1	0	0
PVX_123365	1	0	0
PVX_123370	1	0	0
PVX_123375	0.078805602	0	1.248660181
PVX_123380	0.002855585	0	2.05007689
PVX_123385	0.002855585	0	2.032892401
PVX_123390	0.036687443	0	1.304208723
PVX_123395	0.002855585	0	1.720216385

GeneID	Pvalue	BackGroundPre	BackGroundDx
PVX_123400	0.016473156	0	1.57306728
PVX_123405	0.000383392	0	3.468244389
PVX_123407	0.036687443	0	2.014153594
PVX_123410	0.359317338	0	0.338547792
PVX_123415	1	0	0
PVX_123420	0.078805602	0	0.967967506
PVX_123425	1	0	0
PVX_123430	0.002855585	0	2.510607636
PVX_123435	0.016473156	0	1.979404427
PVX_123440	0.166224591	0	0.387361529
PVX_123445	1	0	0
PVX_123450	0.001084333	0	3.754192034
PVX_123455	0.007054102	0	1.881458429
PVX_123457	1	0	0
PVX_123460	0.078805602	0	0.607017528
PVX_123465	0.359317338	0	0.393391817
PVX_123470	1	0	0
PVX_123475	0.002855585	0	2.377398467
PVX_123480	0.078805602	0	0.95330316
PVX_123485	0.359317338	0	0.317178715
PVX_123490	0.036687443	0	1.665879542
PVX_123495	0.007054102	0	2.396183981
PVX_123500	0.007054102	0	1.622194147
PVX_123505	3.76E-05	0	5.461680094
PVX_123510	1	0	0
PVX_123515	0.016473156	0	1.263976663
PVX_123517	1	0	0
PVX_123520	0.007054102	0	1.618197397
PVX_123525	0.359317338	0	0.40760863
PVX_123530	1	0	0
PVX_123535	0.002855585	0	1.977453588
PVX_123540	0.002855585	0	2.381129194
PVX_123545	0.078805602	0	0.773822632
PVX_123550	0.001084333	0	2.739941065
PVX_123555	1	0	0
PVX_123560	0.166224591	0	0.498225084
PVX_123565	0.359317338	0	0.291084972
PVX_123570	0.078805602	0	0.977553583
PVX_123575	1	0	0
PVX_123580	0.359317338	0	0.381105897
PVX_123585	0.007054102	0	1.481246592

GeneID	Pvalue	BackGroundPre	BackGroundDx
PVX_123590	0.036687443	0	1.189329744
PVX_123592	0.007054102	0	2.043665794
PVX_123595	0.036687443	0	1.093707687
PVX_123597	0.036687443	0	1.210939268
PVX_123600	1	0	0
PVX_123605	1	0	0
PVX_123610	0.078805602	0	0.633707058
PVX_123615	0.359317338	0	0.175349492
PVX_123620	0.002855585	0	2.155589089
PVX_123625	0.001084333	0	2.513319601
PVX_123630	1	0	0
PVX_123632	1	0	0
PVX_123635	0.078805602	0	1.221942886
PVX_123640	0.359317338	0	0.372420668
PVX_123645	0.002855585	0	2.223973871
PVX_123650	0.359317338	0	0.110568582
PVX_123655	0.359317338	0	0.09715523
PVX_123660	0.036687443	0	1.327279884
PVX_123665	0.078805602	0	0.968442003
PVX_123670	0.166224591	0	0.714220478
PVX_123675	1	0	0
PVX_123680	1	0	0
PVX_123682	0.016473156	0	1.941792759
PVX_123685	0.036687443	0	0.829744447
PVX_123690	1	0	0
PVX_123695	0.078805602	0	1.292881024
PVX_123700	1	0	0
PVX_123705	1	0	0
PVX_123710	0.016473156	0	1.355175708
PVX_123715	0.002855585	0	2.772554771
PVX_123720	0.007054102	0	1.648369331
PVX_123725	0.078805602	0	0.742597492
PVX_123730	0.078805602	0	0.77330379
PVX_123735	0.166224591	0	0.583716781
PVX_123740	0.007054102	0	2.398126246
PVX_123745	0.000383392	0	3.166607282
PVX_123750	0.016473156	0	0.9695054
PVX_123755	0.036687443	0	1.526298247
PVX_123760	0.016473156	0	1.065285251
PVX_123765	1	0	0
PVX_123770	0.359317338	0	0.300089131

GeneID	Pvalue	BackGroundPre	BackGroundDx
PVX_123775	0.078805602	0	0.889557259
PVX_123780	1	0	0
PVX_123785	0.016473156	0	2.142428909
PVX_123790	0.036687443	0	1.184072602
PVX_123795	0.007054102	0	1.336106822
PVX_123800	0.001084333	0	2.580046953
PVX_123805	0.007054102	0	0.98647627
PVX_123810	0.016473156	0	1.142478932
PVX_123815	0.007054102	0	2.390337784
PVX_123820	0.016473156	0	1.536328863
PVX_123825	0.000383392	0	4.03327139
PVX_123830	0.016473156	0	1.825675366
PVX_123835	1	0	0
PVX_123840	0.078805602	0	0.675422423
PVX_123845	0.000383392	0	3.755752543
PVX_123850	0.007054102	0	1.93960639
PVX_123855	0.166224591	0	0.836677237
PVX_123860	0.016473156	0	2.007943417
PVX_123865	0.016473156	0	1.3614205
PVX_123870	0.007054102	0	2.0658746
PVX_123875	0.078805602	0	1.475031762
PVX_123880	0.078805602	0	0.659247094
PVX_123885	0.002855585	0	1.865203247
PVX_123890	0.007054102	0	1.594404088
PVX_123895	0.166224591	0	0.688915321
PVX_123900	0.016473156	0	1.877971443
PVX_123905	0.001084333	0	2.98091694
PVX_123910	0.001084333	0	2.4861938
PVX_123915	0.007054102	0	1.568956073
PVX_123920	0.000125309	0	3.556869779
PVX_123925	0.359317338	0	0.306015914
PVX_123930	0.078805602	0	0.746760802
PVX_123935	0.001084333	0	3.634851417
PVX_123940	0.359317338	0	0.221258869
PVX_123945	0.001084333	0	3.324012387
PVX_123950	0.016473156	0	1.272490524
PVX_123955	0.036687443	0	1.041950321
PVX_123960	0.001084333	0	3.684593822
PVX_123965	0.036687443	0	1.350197073
PVX_123970	0.007054102	0	2.452861118
PVX_123975	0.166224591	0	0.558508919

GeneID	Pvalue	BackGroundPre	BackGroundDx
PVX_123980	1	0	0
PVX_123985	0.001084333	0	2.881103172
PVX_123990	0.078805602	0	0.514052025
PVX_123995	0.359317338	0	0.314592683
PVX_124000	0.078805602	0	0.664047771
PVX_124005	0.007054102	0	1.206585536
PVX_124010	0.078805602	0	0.845246427
PVX_124015	0.007054102	0	1.767775117
PVX_124020	0.002855585	0	2.033706016
PVX_124025	0.166224591	0	0.561551691
PVX_124030	0.036687443	0	1.67289495
PVX_124035	0.007054102	0	2.15193111
PVX_124040	0.166224591	0	0.489623122
PVX_124045	0.078805602	0	0.779177457
PVX_124050	0.002855585	0	2.357580882
PVX_124055	0.016473156	0	1.729701456
PVX_124060	1	0	0
PVX_124065	0.016473156	0	1.399155548
PVX_124070	0.001084333	0	2.008648728
PVX_124075	0.016473156	0	1.225026032
PVX_124080	0.016473156	0	1.14101818
PVX_124085	1.03E-05	0	4.106991476
PVX_124090	0.001084333	0	2.890403085
PVX_124095	0.000125309	0	5.406349123
PVX_124100	0.000383392	0	3.979306874
PVX_124105	0.016473156	0	1.653951974
PVX_124110	0.166224591	0	0.739271562
PVX_124115	0.078805602	0	0.774265975
PVX_124120	0.359317338	0	0.167896251
PVX_124130	0.166224591	0	0.647534817
PVX_124140	0.016473156	0	1.246524948
PVX_124145	1	0	0
PVX_124150	0.002855585	0	2.344407344
PVX_124155	1	0	0
PVX_124160	0.001084333	0	3.341691181
PVX_124165	0.007054102	0	2.051541349
PVX_124170	0.007054102	0	2.28559463
PVX_124175	0.002855585	0	2.254286352
PVX_124180	0.016473156	0	1.744456266
PVX_124185	0.166224591	0	0.564888755
PVX_124190	0.166224591	0	0.446762307

GeneID	Pvalue	BackGroundPre	BackGroundDx
PVX_124195	0.002855585	0	2.774432422
PVX_124200	0.078805602	0	0.915534454
PVX_124700	1	0	0
PVX_124705	0.359317338	0	0.276431491
PVX_124708	0.078805602	0	0.770417257
PVX_124710	1	0	0
PVX_124712	1	0	0
PVX_124715	1	0	0
PVX_124720	0.359317338	0	0.209020051
PVX_124725	1	0	0
PVX_125726	1	0	0
PVX_125728	1	0	0
PVX_125730	0.016473156	0	1.678333099
PVX_125735	0.016473156	0	1.593567847
PVX_125738	1	0	0
PVX_127260	1	0	0
PVX_128260	0.359317338	0	0.284966422
PVX_129260	0.166224591	0	1.284249066
PVX_130260	0.166224591	0	0.986134906
PVX_131260	0.078805602	0	1.053898226
PVX_132260	1	0	0
PVX_133260	0.359317338	0	0.284966422
PVX_134260	1	0	0
PVX_135260	1	0	0
PVX_136260	0.001084333	0	3.099673882
PVX_137260	1	0	0
PVX_138260	1.03E-05	0	6.567999429
PVX_139260	1	0	0
PVX_140260	0.078805602	0	0.902608287
PVX_141260	1	0	0
PVX_142260	1	0	0
PVX_143260	1	0	0
PVX_144260	1	0	0
PVX_145260	1	0	0
PVX_146260	1	0	0
PVX_147260	0.000125309	0	5.138418746
PVX_148260	1	0	0
PVX_149260	1	0	0
PVX_150260	0.359317338	0	0.254424199
PVX_151260	1	0	0
PVX_152260	1	0	0

GeneID	Pvalue	BackGroundPre	BackGroundDx
PVX_153260	1	0	0
PVX_154260	1	0	0
PVX_155260	1	0	0
PVX_156260	1	0	0
PVX_157260	1	0	0
PVX_158260	1	0	0
PVX_159260	1	0	0
PVX_160260	1	0	0
PVX_161260	1	0	0
PVX_162260	1	0	0
PVX_163260	1	0	0
PVX_163265	1	0	0
PVX_164265	1	0	0
PVX_165265	1	0	0
PVX_166265	1	0	0
PVX_167265	1	0	0
PVX_168265	1	0	0
PVX_168270	1	0	0
PVX_169270	1	0	0
PVX_170270	0.359317338	0	0.365142007
PVX_171270	1	0	0
PVX_172270	1	0	0
PVX_173270	1	0	0
PVX_174270	1	0	0
PVX_175270	0.166224591	0	0.620706333
PVX_176270	1	0	0
PVX_176275	1	0	0
PVX_177275	0.359317338	0	0.366084257
PVX_178275	1	0	0
PVX_179275	1	0	0
PVX_180275	1	0	0
PVX_181275	1	0	0
PVX_182275	1	0	0
PVX_183275	0.359317338	0	0.2925088
PVX_183280	0.078805602	0	0.939382643
PVX_184280	1	0	0
PVX_184285	1	0	0
PVX_185285	1	0	0
PVX_186285	1	0	0
PVX_186290	1	0	0
PVX_187290	1	0	0

GeneID	Pvalue	BackGroundPre	BackGroundDx
PVX_188290	1	0	0
PVX_191290	0.036687443	0	1.14239854
PVX_192290	0.036687443	0	1.099944925
PVX_193290	0.359317338	0	0.286182265
PVX_195290	0.001084333	0	2.330662045
PVX_196290	0.016473156	0	1.397718601
PVX_198290	0.078805602	0	0.751000132
PVX_200290	0.001084333	0	2.050669943
PVX_201290	0.001084333	0	2.118657989
PVX_202290	0.016473156	0	1.237499267
PVX_203290	0.036687443	0	1.289175184
PVX_206290	0.001084333	0	2.049791817
PVX_208290	0.001084333	0	1.973341732
PVX_209290	0.078805602	0	0.592696931
PVX_210290	0.166224591	0	0.47893653
PVX_211290	0.078805602	0	0.697709311
PVX_212290	1	0	0
PVX_213290	1	0	0
PVX_214290	0.359317338	0	0.192118339
PVX_215290	0.359317338	0	0.192118339
PVX_216290	0.036687443	0	0.910888491
PVX_217290	0.036687443	0	0.971515076
PVX_218290	0.036687443	0	0.910888491
PVX_220290	3.76E-05	0	5.86037746
PVX_221290	0.166224591	0	0.480840735
PVX_222290	0.016473156	0	1.374272912
PVX_223290	0.166224591	0	0.486443362
PVX_224290	0.078805602	0	0.758843729
PVX_225290	0.166224591	0	0.498348461
PVX_226290	0.036687443	0	1.096891228
PVX_227290	1	0	0
PVX_228290	0.036687443	0	0.869276445
PVX_230290	0.359317338	0	0.247006373
PVX_231290	0.036687443	0	1.487590806
PVX_235290	1	0	0
PVX_237290	0.036687443	0	0.910888491
PVX_238290	0.166224591	0	0.661977658
PVX_239290	0.166224591	0	0.57089208
PVX_240290	0.002855585	0	2.299465121
PVX_241290	1	0	0
PVX_241295	1	0	0

GeneID	Pvalue	BackGroundPre	BackGroundDx
PVX_242295	1	0	0
PVX_243295	1	0	0
PVX_244295	1	0	0
PVX_245295	1	0	0
PVX_246295	0.078805602	0	0.799193304
PVX_248300	1	0	0
PVX_249300	0.166224591	0	0.440364568
PVX_250300	0.036687443	0	1.362635195
PVX_251300	0.359317338	0	0.313093893
PVX_252300	0.001084333	0	3.066523469
PVX_253300	0.007054102	0	1.804891507
PVX_254300	0.016473156	0	1.463236897
