# Supplementary material for: Plasmodium vivax readiness to transmit: implication for malaria eradication
Source: BMC Syst Biol. 2019 Jan 11;13:5. doi: 10.1186/s12918-018-0669-4 (PMC6330404; doi:10.1186/s12918-018-0669-4)
Supplement: Supplementary file 4 — Supplemental table s3. Table S3. (PDF 35 kb) [file 12918_2018_669_MOESM4_ESM.pdf]

S3 Table

Gene ID	Ortholog Pfalciparum	Protein Marker candidates (Surface Exposed Markers)	Transcript Marker candidates (Most	Gene Description	Number of patients with Gene Expression	Protein Length	TM Domains	SignalP Scores	Gene Expression in FPKM											
									Low				Medium				High			
									Patient 1	Patient 2	Patient 3	Patient 4	Patient 5	Patient 6	Patient 7	Patient 8	Patient 9	Patient 10	Patient 11	Patient 12
PVX_081315	PF3D7_0109400		Early	tubulin-specific chaperone a, putative	2	168	0		0	0	421.478	0	0	0	0	0	0	0	67.2619	0
PVX_088175	PF3D7_0807400		Marker	hypothetical protein, conserved	7	208	0		0	68.8695	0	631.062	93.9254	0	150.988	136.979	186.828	0	176.624	0
PVX_093660	PF3D7_0801600		Male Early	hypothetical protein, conserved	6	125	1		0	100.284	0	0	45.6136	0	0	398.868	108.79	50.3879	57.1237	0
PVX_090185	PF3D7_0422900		Male Late	hypothetical protein, conserved	3	248	0		0	0	0	0	52.3516	0	0	0	124.814	115.59	0	0
PVX_114130	PF3D7_0620100		Early	hypothetical protein, conserved	4	128	0		0	0	0	0	0	0	244.897	221.875	60.5131	0	31.7714	0
PVX_111495	PF3D7_1024300		Early	hypothetical protein, conserved	7	143	0		0	85.9303	306.41	0	78.151	0	0	683.599	93.2306	86.3833	293.755	0
PVX_080590	PF3D7_0513000	Late Marker	Late	hypothetical protein, conserved	1	271	0	SP	0	0	0	0	0	0	0	0	0	0	36.0339	0
PVX_117165	PF3D7_1466000	Marker	Marker	hypothetical protein, conserved	1	313	0	SP	0	0	0	0	0	0	157.243	0	0	0	0	0
PVX_081290	PF3D7_0110000	Marker	Marker	hypothetical protein	2	234	0	SP	178.36	0	0	0	0	0	0	0	0	0	71.0163	0
PVX_085785	PF3D7_1413300		Marker	hypothetical protein	1	74	1		0	0	0	0	171.908	0	0	0	0	0	0	0
PVX_099745	PF3D7_0926400		Marker	transporter, putative	3	550	13		0	62.9853	0	0	14.3078	0	0	62.6481	0	0	35.9184	0
PVX_079940	PF3D7_0526100		Marker	hypothetical protein, conserved	6	364	3	SP	0	0	0	321.208	31.8716	0	0	139.439	76.0731	35.2524	39.954	0
PVX_001035	PF3D7_0404200		Marker	hypothetical protein, conserved	5	176	0		273.048	0	0	0	87.0847	0	0	0	207.224	95.8312	54.3655	0
PVX_091595	PF3D7_1119200		Marker	conserved protein, unknown function	3	176	0		0	191.17	0	0	0	0	0	0	0	95.8312	54.3655	0
PVX_003620	PF3D7_0203800		Marker	hypothetical protein, conserved	6	553	0		61.3491	0	152.599	0	19.4649	0	93.8454	170.417	116.23	86.218	24.4232	0
PVX_116975	PF3D7_1469800		Marker	hypothetical protein, conserved	5	171	0		0	400.746	0	0	182.582	0	0	0	217.182	100.421	113.948	0
PVX_091045	PF3D7_0816800		Marker	DNA repair protein RAD51, putative (RAD51)	4	350	0		0	0	0	674.262	100.353	0	0	0	0	110.982	83.8581	0
PVX_000930	PF3D7_0406200	Early Marker	Marker	sexual stage antigen s16, putative	3	140	2	SP	0	0	0	0	0	0	0	0	48.6911	45.1121	51.1377	0
PVX_087130	PF3D7_0931500	Marker	Marker	hypothetical protein, conserved	3	212	1	SP	150.313	0	0	0	23.8539	0	0	0	56.9612	105.615	14.9603	0
PVX_098835	PF3D7_0907800	Marker	Marker	ribosomal protein L35, putative	4	162	0	SP	0	0	0	0	199.966	0	0	436.056	0	109.894	62.3593	0
PVX_095330	PF3D7_0318000	Marker	Marker	hypothetical protein, conserved	1	216	0	SP	0	0	0	0	43.8499	0	0	0	0	0	27.461	0
PVX_080680	PF3D7_0511100	Marker	Marker	hypothetical protein, conserved	1	121	0	SP	0	0	0	0	0	0	0	0	43.8418	0	23.024	0
PVX_092700	PF3D7_1141900		Marker	inner membrane complex protein 1b, putative (IMC1b)	1	516	0		0	39.1309	0	0	0	0	0	0	0	0	0	0
PVX_000915	PF3D7_0406600		Marker	hypothetical protein, conserved	2	144	0		386.15	0	0	0	0	0	0	0	0	0	153.793	0
PVX_119435	PF3D7_0306200		Marker	activator of Hsp90 ATPase, putative (AHA1)	8	373	0		0	48.8707	0	223.807	66.6285	0	107.085	97.2107	106.079	196.7	222.891	0
PVX_095430	PF3D7_0316100		Marker	hypothetical protein, conserved	6	324	0		115.961	0	0	0	0	0	177.694	0	131.867	122.192	92.3348	376.205
PVX_084687	PF3D7_1437100		Marker	conserved Plasmodium protein, unknown function	3	164	2	SP	0	0	0	0	0	0	0	427.061	0	215.275	61.0764	0
PVX_114900	PF3D7_1358600		Marker	hypothetical protein, conserved	2	965	0		0	0	0	82.0019	0	0	0	0	0	0	5.11019	41.5408
PVX_122487	PF3D7_1313300		Marker	peptidyl-prolyl cis-trans isomerase, putative	1	215	0		0	0	0	0	0	0	0	0	0	0	40.0601	0
PVX_085300	PF3D7_1423600		Male Late	calcium-dependent protein kinase, putative	1	284	0		0	0	0	0	0	0	0	81.3578	0	20.5813	0	0
PVX_091115	PF3D7_1108800		Male Late	hypothetical protein, conserved	1	150	0		0	0	0	0	0	0	0	0	136.05	0	0	0
PVX_116610	PF3D7_1325300		Male Late	hypothetical protein	9	177	0		264.868	92.554	330.076	0	84.1844	0	0	184.068	150.617	604.67	263.636	214.925
PVX_089735	PF3D7_0415600			adenylate kinase 1, putative	9	231	0		416.851	72.8044	0	0	33.0995	0	159.632	144.803	78.9985	36.6064	62.2343	168.99
PVX_113530	PF3D7_0607300			uroporphyrinogen III decarboxylase, putative (UROD)	3	422	1	SP	0	0	0	0	0	0	0	116.655	31.823	0	66.8605	0
PVX_081307	PF3D7_0109600			cold-shock protein, putative	3	144	0		0	0	0	0	0	0	0	538.042	0	135.45	153.793	0
PVX_088230	PF3D7_0806400			glycosyltransferase, putative	1	186	0		0	0	0	0	0	0	0	0	94.9087	0	0	0
PVX_093600	PF3D7_0802900		Female Early	hypothetical protein, conserved	1	136	0		0	0	0	0	0	0	0	0	0	0	30.8898	0
PVX_079725	PF3D7_0530800	Late Male Marker	Male Late	hypothetical protein, conserved	1	250	0	SP	0	0	321.481	0	0	0	0	0	0	0	25.6795	0
PVX_118405	PF3D7_1441700		Male Late	metalloprotease, putative	2	483	0		71.666	0	0	0	0	0	0	0	0	75.5338	14.2654	0
PVX_079980	PF3D7_0525300		Male Late	hypothetical protein, conserved	2	523	1		65.383	0	0	209.064	0	0	0	0	0	0	26.0292	0
PVX_123505	PF3D7_1216500	Early Male Marker		male development gene 1, putative (MDV1)	11	288	0	SP	159.987	111.734	995.401	255.887	126.96	0	489.754	888.994	454.718	533.947	589.157	777.769
PVX_098695	PF3D7_0905000			hypothetical protein, conserved	5	282	0		0	96.8615	0	444.05	0	0	0	0	52.5403	48.6724	193.12	0
PVX_099465	PF3D7_0921000.1			ubiquitin-conjugating enzyme E2, putative	10	163	0		180.519	378.71	0	579.089	114.886	927.329	554.382	251.018	205.371	380.315	35.938	0
PVX_085000	PF3D7_1430800			hypothetical protein, conserved	1	268	0		0	43.4014	0	0	0	0	0	0	0	21.8389	12.3727	0
PVX_118045	PF3D7_1448900	Late Male Marker		hypothetical protein	1	74	1		0	0	0	0	0	0	0	0	0	0	58.2787	0
PVX_083570	PF3D7_0424000	Male Marker?		Plasmodium exported protein, unknown function	2	300	1		94.7264	397.014	0	0	0	0	0	0	0	0	18.8562	0
