# Supplementary material for: Plasmodium vivax readiness to transmit: implication for malaria eradication
Source: BMC Syst Biol. 2019 Jan 11;13:5. doi: 10.1186/s12918-018-0669-4 (PMC6330404; doi:10.1186/s12918-018-0669-4)
Supplement: Supplementary file 5 — Supplemental methods. Text S1. Full details of the mathematical models of P. falciparum and P. vivax and the calculation of R0. (PDF 365 kb) [file 12918_2018_669_MOESM5_ESM.pdf]

Plasmodium vivax Readiness to Transmit: Implication for Malaria Eradication

S1 Text: Mathematical Models for *P. falciparum* and *P. vivax*

Swamy Rakesh Adapa, Rachel A. Taylor, Chengqi Wang, Richard Thomson-Luque, Leah R. Johnson and Rays H. Y. Jiang

We provide full details of the model equations for both *P. falciparum* and *P. vivax* that are analysed in the main text. We begin with *P. falciparum* as this is the simpler model and is similar to many models that have already been used to study malaria [10, 12].

S1.1 *P. falciparum*

The model put forward for *P. falciparum* is based upon the Ross-MacDonald model [8, 9, 11] developed specifically for malaria but has since been used for many vector-borne diseases. It is a compartmental model of differential equations, i.e. both hosts (humans) and vectors (mosquitoes) are split into different compartments determined by their infection status and we analyze how the dynamics of each compartment changes over time. For *P. falciparum* humans are either Susceptible, $S(t)$, Exposed, $E(t)$, Asymptomatic, $A(t)$, Infected, $I(t)$, or Removed, $R(t)$. A proportion of hosts will show symptoms and are labeled as Infected; the remaining proportion are Asymptomatic but are still able to transmit the disease. Removed hosts are those which have temporary immunity after a recent bout of infection. However, they lose this immunity and move back to being Susceptible to *P. falciparum* after a number of days. Mosquitoes are classified as being Susceptible, $S_M(t)$, Exposed, $E_M(t)$, or Infected, $I_M(T)$. In both cases, Exposed implies that the host or vector has been infected with the disease but is not able to transmit yet. That is, the pathogen is currently proceeding through the internal stages within the host or mosquito.

All hosts are born Susceptible. These Susceptible hosts progress to the Exposed class (Figure S1.1), due to a bite from an Infected vector (with some probability of successful transmission, see Equations (1)-(8)). Once Exposed, a host becomes either Asymptomatic or Infected after a fixed delay to account for the intrinsic incubation period, that is the number of days it takes for *P. falciparum* to reach the blood stage. Whilst all hosts have a natural death rate which includes death by natural causes and other factors unrelated to malaria infection, Infected hosts can also die due to *P. falciparum*. Otherwise, Asymptomatic and Infected hosts clear the infection and move to a Removed class, which indicates temporary immunity from *P. falciparum*. However, this lasts only for a short time, at which point they move back to the Susceptible class.

Similarly, all vectors are born Susceptible. These Susceptible vectors progress to the Exposed class after successful contact with an Infected host. They remain in the Exposed class for the extrinsic incubation period, the duration of time for *P. falciparum* to move from the mid-gut of the mosquito to the salivary glands. They progress to the Infected class and remain so for the

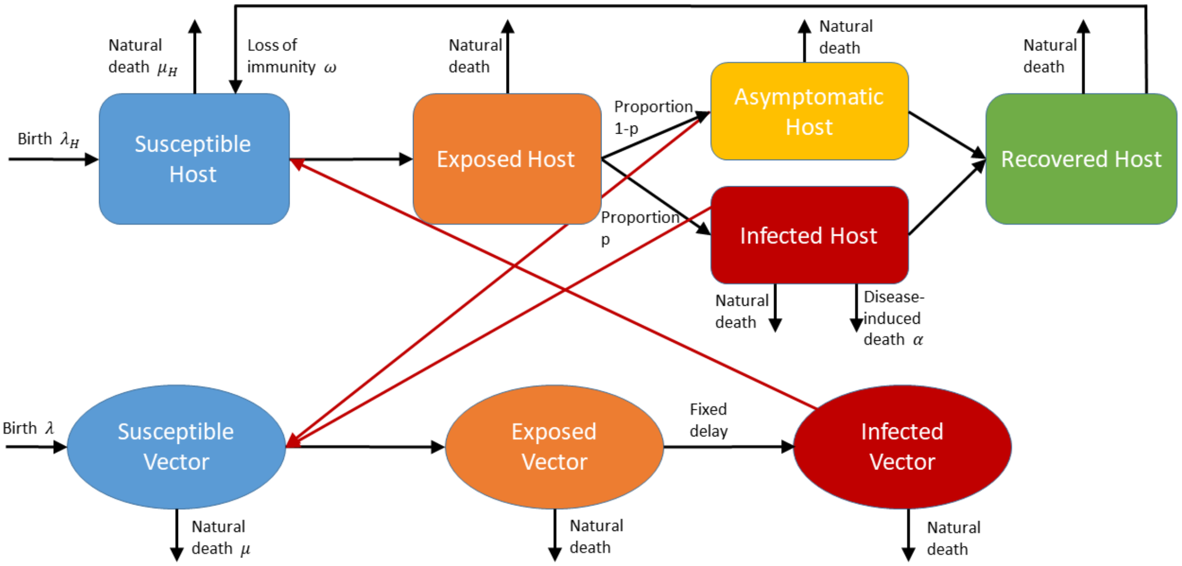

Figure S1.1: A schematic for the *P. falciparum* model. Hosts are represented through the rectangles, vectors in the circles. The processes of how to move between the different compartments are shown with black arrows. The red arrows show the role of interactions between hosts and vectors leading to disease. There are two infectious classes – one which shows symptoms at some point (Infected Host) and one which does not show symptoms (Asymptomatic Host).

remainder of their lifespan. We assume the mosquitoes do not suffer any side-affects from infection by *P. falciparum* nor does it alter their behavior.

We assume that contact between mosquitoes and humans is frequency-dependent, which implies that the number of bites from mosquitoes is not dependent on the density of humans [1]. This is the usual assumption for vector-borne diseases as mosquitoes, and other vectors, will not increase their bite rate because there are more hosts present. This leads to the following model equations:

$$\frac{dS}{dt} = \lambda_H - \frac{ab}{H} I_M S - \mu_H S + wR \tag{1}$$

$$\frac{dE}{dt} = \frac{ab}{H} I_M S - \frac{ab}{H} I_M(t-\tau) S(t-\tau) e^{-\mu_H \tau} - \mu_H E \tag{2}$$

$$\frac{dA}{dt} = (1-p) \frac{ab}{H} I_M(t-\tau) S(t-\tau) e^{-\mu_H \tau} - rA - \mu_H A \tag{3}$$

$$\frac{dI}{dt} = p \frac{ab}{H} I_M(t-\tau) S(t-\tau) e^{-\mu_H \tau} - \alpha I - rI - \mu_H I \tag{4}$$

$$\frac{dR}{dt} = r(A+I) - \mu_H R - wR \tag{5}$$

$$\frac{dS_M}{dt} = \lambda M - (\lambda - \mu) \frac{M^2}{K} - \frac{ac}{H}(A+I) S_M - \mu S_M \tag{6}$$

$$\frac{dE_M}{dt} = \frac{ac}{H} I S_M - \frac{ace^{-\mu EIP}}{H} (A(t-EIP) + I(t-EIP)) S_M(t-EIP) - \mu E_M \tag{7}$$

$$\frac{dI_M}{dt} = \frac{ace^{-\mu EIP}}{H} (A(t-EIP) + I(t-EIP)) S_M(t-EIP) - \mu I_M \tag{8}$$

The parameters of the model are the following: λ_H and μ_H are the birth rate and death rate for humans, respectively; a is the bite rate of mosquitoes; b is the probability that a bite from an infected mosquito on a susceptible host results in successful transmission; c is the probability that a bite from a susceptible mosquito on an infected host results in successful transmission; τ is the time delay for hosts to move from the exposed to the infected class; p is the proportion of hosts that show symptoms when they are infectious; α is the rate of disease-induced death; r is the rate of recovery from the disease; w is the rate that immunity is loss after infection; λ and μ are the birth and death rates of mosquitoes, respectively; K is a carrying capacity for the mosquitoes; EIP is the time delay for the extrinsic incubation period, before mosquitoes move from the exposed to the infected class; lastly $H = S + E + I + R$ is the total host population size and $M = S_M + E_M + I_M$ is the total mosquito population size. For parameter definitions and values, see Table S1.1.

The exponential terms, $e^{-\mu_H \tau}$ and $e^{-\mu EIP}$ account for the fact that natural death of the host or mosquito may occur during the time it is exposed. Thus, not all hosts or vectors that enter the exposed class will progress to the infected class; rather the total is discounted by the exponential term.

We can calculate R_0, the basic reproductive number, which is a widely used criterion to determine the intensity of a disease and it's likelihood to spread. It represents the number of secondary hosts that will be infected when one infected host is introduced into a naïve population. This definition leads to the following criterion: if $R_0 > 1$ disease prevalence is expected to increase in the population; otherwise the number of infected hosts is expected to decline. The above model for *P. falciparum* leads to the following equation for R_0:

$$R_0 = \left(\frac{Ma^2bc}{H\mu} e^{-\mu EIP} e^{-\mu_H \tau} \left(\frac{p}{\alpha + r + \mu_H} + \frac{1-p}{r + \mu_H} \right) \right)^{\frac{1}{2}} \tag{9}$$

This equation can be understood by considering how infection would propagate through the populations of vectors and hosts. It is composed of the ratio of mosquitoes to hosts (M/H); the

number of mosquitoes that would bite the infected host and become infected (ac); the proportion of those mosquitoes that survive to become infectious ($e^{-\mu EIP}$); the lifespan of the mosquito ($\frac{1}{\mu}$); the number of hosts those mosquitoes bite and infect (ab); the proportion of those hosts that survive to become infectious ($e^{-\mu_H \tau}$); the length of time a host is infectious, which is $\frac{1}{\alpha+r+\mu_H}$ for a proportion p of hosts, and $\frac{1}{r+\mu_H}$ for the remaining proportion $1-p$. We will outline the calculations to produce R_0 for the *P. vivax* model below. The same procedure can be used to calculate equation (9) for the *P. falciparum* model.

S1.2 *P. vivax*

We adapt the model for *P. falciparum* to include specific details of *P. vivax*, with a focus on the new information found experimentally. *P. vivax* is significantly different from *P. falciparum*, both because there are believed to be more asymptomatic cases and the high chance of relapses of the disease from 2-3 weeks to months and even years later, depending on whether the strain is tropical or temperate [15]. Asymptomatic cases for *P. falciparum* have been seen, perhaps due to continuous exposure to the disease in regions of high endemicity, but studies have suggested that submicroscopic cases will occur much more frequently for *P. vivax*, with up to 89% of submicroscopic infections being asymptomatic [2, 6]. Relapses of *P. vivax* occur due to the presence of hypnozoites in the liver which will either die over time, or be released into the blood stream, re-infecting the host. The process that causes release of hypnozoites from the liver is unclear. We include two classes of Recovered hosts in our model (Figure S1.2), those with hypnozoites and the possibility of relapse, and those without. We do not keep track of the number of hypnozoites in the liver and thus how many times an individual host can relapse. Instead once they relapse, we treat them as any other infected host with probabilities of being recovered with or without hypnozoites. However, the probability of recovering with hypnozoites is calibrated by the average number of relapses each host is likely to have.

Similar to *P. falciparum*, we include both an asymptomatic compartment and an infected compartment. Those in the infected compartment do not necessarily show symptoms from the beginning of their infection, rather it indicates those hosts which have symptoms at some point, and therefore are likely to seek care and be logged in the health-care system as a *P. vivax* case. We set the proportion of hosts that show symptoms to be a proportion of those in the *P. falciparum* model in order to compare the role of asymptomatic hosts on disease transmission of both *P. falciparum* and *P. vivax*. Of the hosts that relapse in the *P. vivax* model, the same proportion will develop symptoms as for the initial infection.

From our computational experiments, we found that it is possible for human hosts to develop sexual stages much quicker in *P. vivax* than was previously thought possible. We include this reduction in length of the intrinsic incubation period by varying how long it takes for hosts to become infectious, and correspondingly we change the average length of time infectious (such that the total length of time exposed and infectious remains constant).

With these changes to the model, we produce a new schematic to show disease progression for hosts and vectors (Figure S1.2). As before, all hosts are born Susceptible. They progress to Exposed after a successful bite from an infectious vector. The length of time a host is in this class is once again given by a time delay, but we will vary the length of the delay to explore the effects of our experimental results. A proportion k_3p of the hosts will move into the Infected host class, while the remainder progress to the Asymptomatic compartment. Asymptomatic and Infected hosts will transmit the disease and will recover from the disease with the same probability but

only Infected hosts can die due to the disease. The likelihood of hosts recovering with or without hypnozoites is the same for both Infected and Asymptomatic hosts.

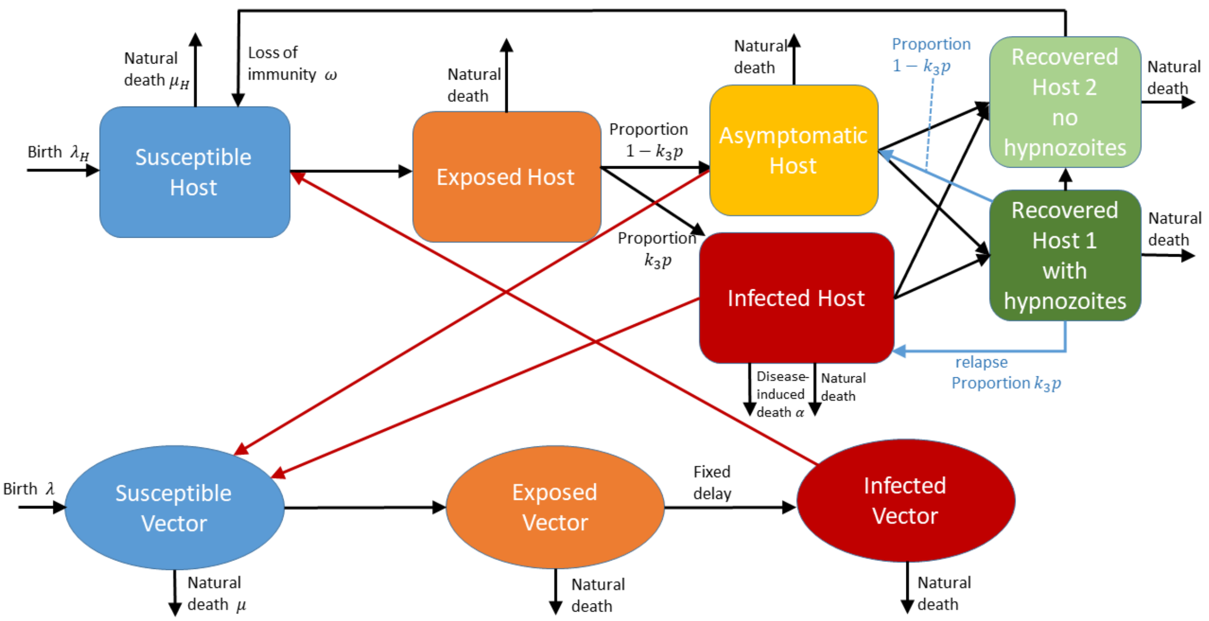

Figure S1.2: A schematic for the *P. vivax* model. Hosts are represented through the rectangles, vectors in the circles. The processes of how to move between the different compartments are shown with black arrows. The red arrows show the role of interactions between hosts and vectors leading to disease. After infection, a proportion have hypnozoites in their liver which can either cause relapse, or the hypnozoites die, in which case they move to Recovered Host 2 class before loss of immunity leads to movement back to the Susceptible class. Relapse can either be back into the Infected class or the Asymptomatic class, with the same proportion as for an initial infection.

The equations for the P. vivax model are:

$$\frac{dS}{dt} = \lambda_H - \frac{ab}{H} I_M S + wR_2 - \mu_H S \tag{10}$$

$$\frac{dE}{dt} = \frac{ab}{H} I_M S - \frac{ab}{H} I_M(t-\tau_1)S(t-\tau_1)e^{-\mu_H \tau_1} - \mu_H E \tag{11}$$

$$\frac{dA}{dt} = \frac{(1-k_3p)ab}{H} I_M(t-\tau_1)S(t-\tau_1)e^{-\mu_H \tau_1} + (1-k_3p)\nu R_1 - r_1 A - \mu_H A \tag{12}$$

$$\frac{dI}{dt} = \frac{k_3pab}{H} I_M(t-\tau_1)S(t-\tau_1)e^{-\mu_H \tau_1} + k_3p\nu R_1 - k_1\alpha I - r_1 I - \mu_H I \tag{13}$$

$$\frac{dR_1}{dt} = k_2 r_1(A+I) - \nu R_1 - \eta R_1 - \mu_H R_1 \tag{14}$$

$$\frac{dR_2}{dt} = (1-k_2)r_1(A+I) + \eta R_1 - wR_2 - \mu_H R_2 \tag{15}$$

$$\frac{dS_M}{dt} = \lambda M - (\lambda - \mu)\frac{M^2}{K} - \frac{ac}{H}(A+I)S_M - \mu S_M \tag{16}$$

$$\frac{dE_M}{dt} = \frac{ac}{H}(A+I)S_M - \frac{ac}{H}(A(t-EIP) + I(t-EIP))S_M(t-EIP)e^{-\mu EIP} - \mu E_M \tag{17}$$

$$\frac{dI_M}{dt} = \frac{ac}{H}(A(t-EIP) + I(t-EIP))S_M(t-EIP)e^{-\mu EIP} - \mu I_M \tag{18}$$

The new host compartments, as discussed above, are Recovered with hypnozoites, $R_1(t)$, and Recovered without hypnozoites, $R_2(t)$. Parameters with the same name as in Equations (1)-(8) are defined in the same way and have the same value. The new parameters are: τ_1 is the length of the exposed time delay; k_3, a proportionality constant relating the proportion of hosts that show symptoms in the *P. vivax* model to the *P. falciparum* model; k_1, a proportionality constant relating death caused by *P. vivax* to *P. falciparum*; r_1, the rate of recovery in *P. vivax*; k_2, the proportion of hosts leaving the Infected/Asymptomatic stages that have hypnozoites in their liver, and thus can potentially relapse; η, the rate of death of hypnozoites in the liver and thus movement from one Removed class to the second; and ν, the rate of relapse caused by hypnozoites. Parameter values and definitions are given in Table S1.1.

S1.2.1 Calculating R_0

We go through the procedure to calculate R_0 for this model. It is based upon the work of [4, 5] which introduces the idea of next-generation matrices. Some good examples of using this method are provided in [14, 17] with specific examples for vector-borne diseases. A further example, specifically for delay-differential equations, is in [1]. The next-generation matrix approach involves calculating R_0 as the largest eigenvalue of the matrix FV^{-1} in which F is defined as the transmission matrix and V is defined as the transition matrix. We outline this process in more detail: first we focus only on those classes which are considered infectious. For our model (10)-(18) for *P. vivax*, the infectious classes are (10), (11), (12), (13), (16) and (17), i.e. Exposed host, Asymptomatic host, Infected host, Recovered host with hynpnozoites, Exposed vector and Infected vector. For both matrices, the rows and columns will be delimited by these classes in the order just stated. The transmission matrix F is a matrix indicating new infections arising in each of the population

classes, moving from columns to rows. Explicitly, it outlines how a host or vector in a column class can lead to a new infection in a vector or host in the row classes. For our system, we have the following matrix for F:

$$F = \begin{pmatrix} 0 & 0 & 0 & 0 & 0 & \frac{ab}{H}S \\ 0 & 0 & 0 & 0 & 0 & 0 \\ 0 & 0 & 0 & 0 & 0 & 0 \\ 0 & 0 & 0 & 0 & 0 & 0 \\ 0 & \frac{ac}{H}S_M & \frac{ac}{H}S_M & 0 & 0 & 0 \\ 0 & 0 & 0 & 0 & 0 & 0 \end{pmatrix} \tag{19}$$

For example, the term in the first row is the rate of new infections that arise from an infected mosquito that go into the E class. Those on the 5th row represent the rate of new infections arising in the E_M class from both the A and I classes.

The matrix V is a transition matrix, outlining other movement between classes not caused by new infectious contact between mosquito and host. Once again, we have each row and column representing equations (10)-(13), (16) and (17) in that order. Of particular note is that movement out of a class is considered positive, whilst movement into a class is negative. This produces the following transition matrix:

$$V = \begin{pmatrix} 1 & 0 & 0 & 0 & 0 & 0 \\ -(1-k_3p)e^{-\mu_H\tau_1} & r_1+\mu_H & 0 & -(1-k_3p)\nu & 0 & 0 \\ -k_3pe^{-\mu_H\tau_1} & 0 & k_1\alpha+r_1+\mu_H & -k_3p\nu & 0 & 0 \\ 0 & -k_2r_1 & -k_2r_1 & \nu+\eta+\mu_H & 0 & 0 \\ 0 & 0 & 0 & 0 & 1 & 0 \\ 0 & 0 & 0 & 0 & -e^{-\mu EIP} & \mu \end{pmatrix} \tag{20}$$

How to treat movement between classes when there is a fixed delay is not intuitive. We handle this by noting that all hosts or vectors leave the exposed class, either through time delayed movement to an infected class, or through death. As there is a time delay, it is not important to know the rate at which hosts or vectors leave the class. Thus, we have 1's in (row, column) = $(1,1)$ and $(5,5)$ in V indicating that all hosts/vectors leave these exposed classes. But we need to consider what proportion of the exposed class progresses to the infected class rather than dying: these are the exponential terms in $(2,1)$, $(3,1)$ and $(6,5)$, with the appropriate negative signs to indicate movement into the class.

The next step is to calculate V^{-1}. First we calculate the determinant of the matrix:

$$\begin{aligned} \det(V) =& \mu(r_1+\mu_H)\left((k_1\alpha+r_1+\mu_H)(\nu+\eta+\mu_H) - k_3p\nu k_2r_1\right) - \mu(k_1\alpha+r_1+\mu_H)(1-k_3p)\nu k_2r_1 \\ =& \mu((r_1+\mu_H)G_1 - (k_1\alpha+r_1+\mu_H)G_2) \end{aligned}$$

where we introduce G_1 and G_2 to simplify the presentation. Proceeding through the steps of calculating the inverse, which can be done by hand or using a suitable computer program, produces the following:

$$V^{-1} = \frac{1}{\det V} \begin{pmatrix} \det V & 0 & 0 & 0 & 0 & 0 \\ e^{-\mu_H \tau_1}((1-k_3p)G_1 + k_3pG_2) & \mu G_1 & \mu G_2 & (k_1\alpha + r_1 + \mu_H)(1-k_3p)\nu\mu & 0 & 0 \\ k_3pe^{-\mu_H\tau_1}(r_1+\mu_H)(\nu+\eta+\mu_H)\mu & \mu G_2 \frac{k_3p}{1-k_3p} & (r_1+\mu_H)(\nu+\eta+\mu_H)\mu - \mu G_2 & (r_1+\mu_H)k_3p\nu\mu & 0 & 0 \\ e^{-\mu_H\tau_1}\mu\frac{G_2}{\nu}\left((k_1\alpha + r_1 + \mu_H) + (r_1+\mu_H)\frac{k_3p}{1-k_3p}\right) & (k_1\alpha + r_1 + \mu_H)k_2r_1\mu & (r_1+\mu_H)k_2r_1\mu & (k_1\alpha + r_1+\mu_H)(r_1+\mu_H)\mu & 0 & 0 \\ 0 & 0 & 0 & 0 & \det V & 0 \\ 0 & 0 & 0 & 0 & \frac{\det V}{\mu}e^{-\mu EIP} & \frac{\det V}{\mu} \end{pmatrix} \tag{21}$$

Next we calculate FV^{-1} in order to compute its eigenvalues. As the matrix is mostly zeroes but some terms are quite long, we separately display the components of the matrix which are non-zero using (row, column) notation:

$$\begin{aligned}
(5,1) =& \frac{ac\mu}{\det V}\frac{S_M}{H}e^{-\mu_H\tau_1}\left[(1-k_3p)\left(G_1 + G_2\frac{k_3p}{1-k_3p}\right) + k_3p(r_1+\mu_H)(\nu+\eta+\mu_H)\right] \\
(5,2) =& \frac{ac\mu}{\det V}\frac{S_M}{H}\left(G_1 + G_2\frac{k_3p}{1-k_3p}\right) \\
(5,3) =& \frac{ac\mu}{\det V}\frac{S_M}{H}(r_1+\mu_H)(\nu+\eta+\mu_H) \\
(5,4) =& \frac{ac\mu}{\det V}\frac{S_M}{H}\left((k_1\alpha + r_1+\mu_H)(1-k_3p)\nu + (r_1+\mu_H)k_3p\nu\right) \\
(1,5) =& \frac{abS}{H}\frac{e^{-\mu EIP}}{\mu} \\
(1,6) =& \frac{abS}{H}\frac{1}{\mu}
\end{aligned}$$

One important aspect to note is that for calculating R_0 we assume that there is only one infected host in an otherwise susceptible population of both hosts and vectors. Therefore, we will assume that $S \approx H$ and $S_M = M$, the total vector population.

The final step is to calculate the eigenvalues of this matrix FV^{-1}. There are 4 zero eigenvalues and a double root of a quadratic. As R_0 is the largest positive eigenvalue, we take the positive root. With some simplification, we end with:

$$R_0 = \left\{a^2bc\frac{M}{H}\frac{e^{-\mu EIP}}{\mu}e^{-\mu_H\tau_1}\left[\frac{(1-k_3p)(k_1\alpha+r_1+\mu_H) + k_3p(r_1+\mu_H)}{(k_1\alpha+r_1+\mu_H)(r_1+\mu_H) - ((1-k_3p)(k_1\alpha+r_1+\mu_H)+k_3p(r_1+\mu_H))\left(\frac{\nu k_2 r_1}{\nu+\eta+\mu_H}\right)}\right]\right\}^{\frac{1}{2}} \tag{22}$$

Although this equation seems complicated, it can be reasonably well understood by thinking in terms of how the disease progresses through the vector and host populations. Most of the terms

in (9) are present in (22); the difference results from a more complicated length of time infectious. To understand it more clearly, we can consider some of the components separately:

$$\frac{(1-k_3p)(k_1\alpha+r_1+\mu_H)+k_3p(r_1+\mu_H)}{(k_1\alpha+r_1+\mu_H)(r_1+\mu_H)} = k_3p\frac{1}{k_1\alpha+r_1+\mu_H}+(1-k_3p)\frac{1}{r_1+\mu_H} \tag{23}$$

The right hand side has a more intuitive meaning than the left: a proportion k_3p go into the infected class for an average length of time infectious given by $\frac{1}{k_1\alpha+r_1+\mu_H}$, while a proportion $1-k_3p$ move into the asymptomatic class for an average length of time $\frac{1}{r_1+\mu_H}$. This term within (22) is regulated by those hosts that relapse: $\frac{\nu}{\nu+\eta+\mu_H}$ is the proportion of recovered hosts with hypnozoites that relapse, and k_2r_1 is the rate at which infected and asymptomatic hosts recover with hypnozoites.

S1.2.2 The role of Asymptomatic Hosts

Above, in the *P. vivax* model, we assumed that a proportion k_3p of infected hosts had symptoms at some point, whilst the remaining proportion $1-k_3p$ were asymptomatic. It is unclear how many asymptomatic hosts there could be, therefore we vary the parameters p and k_3 to assess the effect of asymptomatics on R_0. However, this is assuming that we take into account that asymptomatics exist, but do not know in what proportion. We ask now, what happens when you do not take into account transmission by asymptomatics at all? To do this we use similar equations to (10)-(18) but we no longer think of the Asymptomatic host $A(t)$ as able to transmit infection. However, exposed hosts can still move into the asymptomatic class with the same proportion $1-k_3p$. When calculating R_0 using the procedure outlined above, the $A(t)$ equation will not be considered an infectious class. This leads to the following equation for R_0:

$$R_0 = \left(a^2bc\frac{M}{H}\frac{e^{-\mu EIP}}{\mu}k_3pe^{-\mu_H\tau_1}\frac{1}{k_1\alpha+r_1+\mu_H-k_3p\left(\frac{\nu k_2r_1}{\nu+\eta+\mu_H}\right)}\right)^{\frac{1}{2}} \tag{24}$$

We can compare this R_0 to the R_0 for *P. vivax* and *P. falciparum* above and assess how much information is lost by not taking into account the asymptomatic compartment.

S1.3 Parameter Values

We outline our choice of parameter values for the *P. falciparum* and *P. vivax* models (Table S1.1). We only consider those parameters that are different between the R_0 equations (9) and (22). We assume that the transmission rates between hosts and mosquitoes and the length of time for the mosquito to become infectious will be the same for *P. falciparum* and *P. vivax*.

The host death rate is given by assuming an average lifespan of 71.5 years [13]. The disease-induced death rate for *P. falciparum* is found by assuming 1 million deaths per year to an estimated 350-500 million cases [18]. *P. vivax* has an estimated 13.8 million cases per year with death caused by *P. vivax* in the range 1400 - 14900 [18]. Therefore, the disease-induced death rate of *P. vivax* should be between 0.0001-0.001 and hence k_1, the proportionality constant relating the two disease-induced death rates, will be between 0.05-0.5.

To determine what proportion of hosts leaving the infectious classes will recover with hypnozoites (parameter k_2), we use the fact that on average a host will have 2.1 relapses [16]. Using the

Parameter	Definition	Value	Reference
μ_H	Host death rate	3.84×10^{-5}	[13]
α	Disease-induced death rate for *P. falciparum*	0.002	[18]
k_1	Proportional rate of disease death for *P. vivax*	0.25	[18]
r	Recovery rate for *P. falciparum*	1/60	[3]
r_1	Recovery rate for *P. vivax*	$1/(60+\epsilon)$	-
τ	Length of exposed period for *P. falciparum*	14	[7]
τ_1	Length of exposed period for *P. vivax*	$\tau - \epsilon$	-
ϵ	Reduction in length of exposed period for *P. vivax*	0–7 (3.5)	-
p	Proportion of infected *P. falciparum* hosts with symptoms	0–1 (1)	-
k_3	Proportional rate of having symptoms in *P. vivax* model	0–1 (1)	-
k_2	Proportion of recovering hosts with hypnozoites	0.68	[16]
ν	Hypnozoite relapse rate	1/72	[16]
η	Hypnozoite death rate in liver	1/223	[16]

Table S1.1: Parameter definitions with the values used to calculate R_0 for *P. falciparum* (Eqn (9)) and for *P. vivax* (Eqn (22)). For p, k_3 and ϵ, their range is given with a base value given in parentheses. All rates are in days

concept of Bernoulli trials, the host essentially has an average of 2.1 failures (clearing the infection with hypnozoites) until it has a success (clearing the infection without hypnozoites). This leads to a probability of 0.68 for a failure, i.e. clearing with hypnozoites.

We introduce a new parameter, ϵ, to describe the reduction in length of the intrinsic incubation period, and hence the increase in length until average recovery time with *P. vivax*. A larger ϵ indicates a bigger difference in incubation length between *P. falciparum* and *P. vivax* which corresponds to the disease transmitting sooner for *P. vivax*. This affects both parameters τ and r as seen in Table S1.1, producing τ_1 and r_1. Parameters ϵ, p and k_3 vary over a range therefore we set a baseline value for them, for p and k_3 this is 1 to indicate the baseline assumption is that there are no asymptomatic hosts for either *P. falciparum* or *P. vivax*.

S1.4 Methods

We use Matlab to compare the values of R_0 for *P. falciparum* and *P. vivax*. As we are looking at the relative values of R_0 we need only consider those terms within each R_0 which are different. We treat the value of R_0 for *P. falciparum* as a baseline, setting it's value equal to 1 when $p = 1$ and determining how much R_0 for *P. vivax* differs. We focus on the change in the length of the intrinsic incubation period which correspondingly changes the length of time infectious, but also consider the role of asymptomatic hosts in spreading infection and of relapses. To do this, we perform a sensitivity analysis for R_0, in which all parameters are varied by 10% and the resultant change in R_0 is plotted. For parameters p, k_3 and ϵ, we vary the parameters over their whole range rather than by 10%.

References

[1] R. M. Anderson and R. M. May. *Infectious diseases of humans: dynamics and control*, volume 28. Wiley Online Library, 1991.

[2] Q. Cheng, J. Cunningham, and M. L. Gatton. Systematic review of sub-microscopic P. vivax infections: prevalence and determining factors. *PLoS Negl Trop Dis*, 9(1):e3413, 2015.

[3] W. E. Collins, G. M. Jeffery, and J. M. Roberts. A retrospective examination of anemia during infection of humans with Plasmodium *vivax*. *The American journal of tropical medicine and hygiene*, 68(4):410–412, 2003.

[4] O. Diekmann and J. A. P. Heesterbeek. *Mathematical epidemiology of infectious diseases: model building, analysis and interpretation*, volume 5. John Wiley & Sons, 2000.

[5] O. Diekmann, J. Heesterbeek, and M. Roberts. The construction of next-generation matrices for compartmental epidemic models. *Journal of the Royal Society Interface*, page rsif20090386, 2009.

[6] R. E. Howes, K. E. Battle, K. N. Mendis, D. L. Smith, R. E. Cibulskis, J. K. Baird, and S. I. Hay. Global epidemiology of Plasmodium vivax. *The American Journal of Tropical Medicine and Hygiene*, 95(6 Suppl):15–34, 2016.

[7] D. H. Kerlin and M. L. Gatton. A simulation model of the within-host dynamics of Plasmodium *vivax* infection. *Malaria journal*, 14(1):1–9, 2015.

[8] G. Macdonald. The analysis of equilibrium in malaria. *Tropical diseases bulletin*, 49(9): 813–829, 1952.

[9] G. Macdonald. Epidemiologic models in studies of vector-borne diseases: The R.E Dyer lecture. *Public health reports*, 76(9):753, 1961.

[10] R. C. Reiner, T. A. Perkins, C. M. Barker, T. Niu, L. F. Chaves, A. M. Ellis, D. B. George, A. Le Menach, J. R. Pulliam, D. Bisanzio, et al. A systematic review of mathematical models of mosquito-borne pathogen transmission: 1970–2010. *Journal of The Royal Society Interface*, 10(81):20120921, 2013.

[11] R. Ross. The prevention of malaria. 1911.

[12] D. L. Smith, K. E. Battle, S. I. Hay, C. M. Barker, T. W. Scott, and F. E. McKenzie. Ross, macdonald, and a theory for the dynamics and control of mosquito-transmitted pathogens. *PLoS pathog*, 8(4):e1002588, 2012.

[13] United Nations Department of Economic and Social Affairs. United Nations World Population Prospects: 2015 revision. 2015.

[14] P. Van den Driessche and J. Watmough. Reproduction numbers and sub-threshold endemic equilibria for compartmental models of disease transmission. *Mathematical biosciences*, 180 (1):29–48, 2002.

[15] M. T. White, S. Karl, K. E. Battle, S. I. Hay, I. Mueller, and A. C. Ghani. Modelling the contribution of the hypnozoite reservoir to Plasmodium *vivax* transmission. *Elife*, 3:e04692, 2014.

[16] M. T. White, G. Shirreff, S. Karl, A. C. Ghani, and I. Mueller. Variation in relapse frequency and the transmission potential of Plasmodium *vivax* malaria. In *Proc. R. Soc. B*, volume 283, page 20160048. The Royal Society, 2016.

[17] M. J. Wonham, T. de Camino-Beck, and M. A. Lewis. An epidemiological model for west nile virus: invasion analysis and control applications. *Proceedings of the Royal Society of London B: Biological Sciences*, 271(1538):501–507, 2004.

[18] World Health Organisation. World Malaria Report. 2015.
